# Effects of Xiao Chengqi Formula on Slow Transit Constipation by Assessing Gut Microbiota and Metabolomics Analysis *in vitro* and *in vivo*

**DOI:** 10.3389/fphar.2022.864598

**Published:** 2022-06-08

**Authors:** Qian Zhou, Di Zhang, Heng Zhang, Xingyang Wan, Bang Hu, Qi Zou, Dan Su, Hui Peng, Dandan Huang, Donglin Ren

**Affiliations:** ^1^ Department of Coloproctology, The Sixth Affiliated Hospital, Sun Yat-sen University, Guangzhou, China; ^2^ Guangdong Provincial Key Laboratory of Colorectal and Pelvic Floor Diseases, The Sixth Affiliated Hospital, Sun Yat-sen University, Guangzhou, China; ^3^ Guangdong Institute of Gastroenterology, The Sixth Affiliated Hospital, Sun Yat-sen University, Guangzhou, China

**Keywords:** Xiao Chengqi formula, traditional Chinese medicine, slow transit constipation, butyl aminobenzene, interstitial cells of cajal, interleukin-21 receptor

## Abstract

The Xiao Chengqi (XCQ) formula is a newly constituted traditional Chinese medicine prescription in the treatment of intestinal motility deficiency and is effective in patients with slow transit constipation (STC). XCQ formula was reconstructed based on a “Chengqi” decoction. Astragali Radix, Angelicae Sinensis Radix, and cooked ground Salviae Miltiorrhizae Radix et Rhizoma were added to the prescription to enhance. An STC rat model was constructed and treated with the formula to understand the detailed mechanism by which XCQ promotes intestinal peristalsis. The effects of the XCQ formula on intestinal microflora and metabolic levels and the possible molecular mechanism of its regulation were explored using 16S rDNA sequencing, metabolomics sequencing, and tissue RNA sequencing. The results showed a significant decrease in the abundance of *Roseburia* spp. in the feces of STC rats, a significant decrease in the content of butyl aminobenzene (BAB) in feces, and an increase in the number of interstitial cells of Cajal (ICC) in the colon of STC rats. Furthermore, *in vitro* and *in vivo* experiments revealed that BAB could activate IL-21R on the ICC surface, upregulate the phosphorylation of the downstream molecules STAT3 and ERK, and inhibit loperamide-induced ICC apoptosis. Therefore, the XCQ formula can improve the defecation status of patients with STC by protecting ICC activity, promoting the colonization of *Roseburia* spp*.* to promote peristalsis*,* and increasing the BAB content after metabolism.

## Introduction

Chronic constipation is a common anorectal disease with a high worldwide incidence. Its incidence is rapidly increasing and has been correlated with age, dietary habits, psychosocial factors, changes in the ecological environment, and the accelerating pace of modern life (Aziz et al., 2020). The prevalence of constipation in the adult population in China is 3.19%–11.6% (Aziz et al., 2020). A Finland survey conducted on elderly individuals who had been hospitalized for a long time showed a constipation prevalence as high as 79% (Suzanne et al., 2011). Slow transit constipation (STC) is the most common type of chronic constipation, accounting for 46%–74% of chronic constipation (Christopher et al., 2020). Although there is a prolonged transmission of colonic contents, there are no abnormalities in pelvic floor structure and function. Current STC treatment modalities include high fibre diet, exercise, lifestyle habits, drugs, biofeedback, and psychological treatment intervention. However, because of the long treatment period and scope of drug dependence, it does not have a significant curative effect in most patients. Biofeedback is effective in only 8% of patients with STC (Adil and Brian, 2020). The lengthy treatment process takes an enormous toll on patients’ finances and general quality of life. Thus, it is important to explore the pathogenesis and develop more effective treatment methods for STC to provide an experimental basis for clinical diagnosis and treatment.

The etiology of STC is complex, and its pathogenesis has not been fully delineated. Presently, it is believed that intestinal nervous system lesions, Cajal stromal cells, smooth muscle, and changes in intestinal neurotransmitters are important factors that induce STC (Charles et al., 2001; Wedel et al., 2002; Gary and Jill, 2013). The interstitial cells of Cajal (ICC) are a special type of interstitial cell in the submucosal and muscular layer of the gastrointestinal tract. They can produce physiological slow waves, promote the spread of electrical activity, and control the contraction and peristalsis of smooth muscles in the gastrointestinal tract. Their number and phenotypic abnormalities are considered key factors in constipation. Studies have shown that the number of ICC in the tissues of patients with intestinal dysfunction is lower than that in the control group, and their ultrastructure is damaged (Lyford et al., 2002; Iino et al., 2020). Moreover, many studies have shown that the STC intestinal autoimmune status and inflammatory environment are changed; however, the specific underlying mechanism is unclear.

Graded treatment of STC was employed according to established guidelines. Initially, lifestyle adjustment and biofeedback were performed, and drug treatment was selected for those who found it difficult to make and adhere to lifestyle adjustments. Drug treatment for STC mainly includes volumetric laxatives, osmotic laxatives, irritant laxatives, and intestinal motility promoters. Although these drugs have good short-term efficacy, their long-term use can lead to drug dependence and resistance, which may further aggravate the disease (Adil and Brian, 2020). Surgical treatment, mainly total colectomy or subtotal colectomy, can be used in patients with no improvement after more conservative treatments (Knowles et al., 2017). However, the surgical treatment also causes several problems, such as increased frequency of defecation and decreased anal control, which have acute negative effects on the quality of life of patients. Therefore, great care is necessary for the selection of surgical treatment. The treatment of constipation using traditional Chinese medicine (TCM) has a long history and unique advantages. Many studies have shown that for the treatment of chronic constipation, the combination of Chinese and Western medicine confers significant benefits over Western medicine alone (Linda et al., 2016). Recently, with increasing research on the mechanism of TCM treatment for constipation, increasing evidence has shown that TCM can promote defecation by improving the composition of intestinal microbiota, producing related metabolites, increasing intestinal mucosal secretion, modulating water reabsorption, alleviating intestinal inflammatory responses, and promoting intestinal peristalsis (Xu et al., 20192019). TCM hospitals have reported many anecdotal cases in which TCM compounds achieved good efficacy in the treatment of chronic constipation, but they cannot be widely promoted because their mechanism of action is still unclear.

The Xiao Chengqi (XCQ) formula is the representative recipe for “Treatise on Febrile Diseases.” As per TCM, it is used to lighten Yangming, clear dryness, and remove Pi. We used the XCQ formula based on a small Chengqi decoction and added Astragali Radix, Angelicae Sinensis Radix, and cooked ground Salviae Miltiorrhizae Radix et Rhizoma into the prescription to enhance the treatment of constipation. In a previous study, the application of these constituents led to a reduction in intestinal gas production in postoperative patients treated for colonic anastomosis. The results showed that using this formulation in patients with colon cancer with impaired bowel function significantly shortened the postoperative recovery time compared to conventional treatment and did not increase the risk of anastomotic fistula in mouse models. However, the specific mechanisms underlying the promotion of intestinal peristalsis remain unclear.

To address these questions, in the present study, we established an STC rat model and administered the XCQ formula to observe its efficacy in improving intestinal peristalsis. Through 16S rDNA sequencing and metabolomics, we aimed to explore the influence of the XCQ formula on the intestinal flora. We hypothesized that the intestinal tissue cells and their mechanism of action may be affected by the XCQ formula through RNA-seq of the colon tissue. Finally, we sought to verify this hypothesis in the primary ICC of the colon to clarify the potential molecular mechanism of the promotion of intestinal peristalsis by the XCQ formula. These results provide a solid theoretical basis for the recombination and development of TCM prescriptions in clinical applications for STC.

## Materials and Methods

### Preparation of the XCQ Formula

The composition of the formula was as follows: Rhei Radix et Rhizoma (Rhizomes of *Rheum officinale* Baill, Polygonaceae) 15 g, Magnolia Officinalis Cortex (Barks of *Magnolia officinalis Rehd.et* Wils, Magnoliaceae) 15 g, Aurantii Fructus Immaturus (Fruitlet of *Citrus aurantium* L, Rutaceae) 15 g, Astragali Radix (Roots of *Astragalus membranaceus* Bge, Leguminosae) 20 g, Angelicae Sinensis Radix (Roots of *Angelica sinensis* Diels, Umbelliferae) 15 g, Rehmanniae Radix (Roots of *Rehmannia glutinosa* Libosch, Umbelliferae) 15 g, and Salviae Miltiorrhizae Radix et Rhizoma (Rhizomes of *Salvia miltiorrhiza* Bge, Labiatae) 15 g. To the prescribed amount of Astragali Radix and Rhei Radix et Rhizoma, 100 ml of boiling water was added, decocted for 10 min, and strained, and the filtrate was reserved. Rehmanniae Radix*,* Salviae Miltiorrhizae Radix et Rhizoma*,* Magnolia Officinalis Cortex*,* Angelicae Sinensis Radix*,* and Aurantii Fructus Immaturus were prepared by prescription, and 400 ml of water was added, soaked for 2 h, and decocted twice, 1 h after the first boiling and 0.5 h after the second boiling. The decoction and filtrate of Rhei Radix et Rhizoma were combined, filtered, and concentrated to a relative density of 1.15 (39–40°C), and then cooled naturally.

### Reagents

PD98059 (MedChemExpress, Shanghai, China), STATTIC (MedChemExpress), and MK-2206 2HCl (Selleck, Houston, TX, United States) were dissolved in DMSO separately to obtain a 10 mM solution, which was stored at −20°C. Loperamide hydrochloride (MedChemExpress) was dissolved in DMSO to prepare a 10 mM stock solution and stored at −20°C. Antibodies against poly ADP-ribose polymerase (PARP) (Cat. #9532), caspase-3 (Cat. #9662), cleaved-caspase-3 (Cat. #9664), caspase-9 (Cat. #9508), cleaved-caspase-9 (Cat. #9505 and #9507), Bcl-2 (Cat. #15071), Bax (Cat. #2772), cyclin D1 (Cat. #55506), p21 (Cat. #2947), STAT3 (Cat. #12640) and phospho-STAT3 (Cat. #9145), ERK (Cat. #4695) and phospho-ERK (Cat. #4370), and AKT (Cat. #4691) and phospho-AKT phospho-AKT (Cat. #4060), CD117 (Cat. #3074) were obtained from Cell Signaling Technology (Beverly, MA, United States). Bcl-2 (Cat. ab196495), Alexa Fluor^®^ 488 (Cat. ab150113), and Alexa Fluor^®^ 555 (Cat. ab150078) were obtained from Abcam (Cambridge, MA, United States). Interleukin-21 receptor (IL-21R) antibody (Cat. PA5-19982), and CD117 (Cat. 14-1172-82) were obtained from Invitrogen (Camarillo, CA, United States). β-Actin (Cat. 20536-1-AP), anti-mouse immunoglobulin G (Cat. SA00001-1), and anti-rabbit immunoglobulin G horseradish peroxidase-conjugated secondary antibodies (Cat. SA00001-2) were obtained from Proteintech Group (Chicago, IL, United States).

### High-Performance Liquid Chromatography Tandem Mass Spectrometry

All samples were stored at 4°C. Moreover, 100 µl of each sample was transferred into 2-ml centrifuge tubes (samples with a sample size < 50 µl were treated with half equivalents of the experimental system, but the resolution system remained unchanged). Methanol (400 µl at −20°C) was added to each tube, vortexed for 60 s, and centrifuged at 4°C for 10 min at 12,000 rpm. The supernatant was transferred from each sample into another 2-ml centrifuge tube. The samples were concentrated to dryness under a vacuum. The samples were dissolved in 4 ppm 2-chlorobenzalanine (80%) methanol solution (150 µl), and the supernatant was filtered through a 0.22-µm membrane to obtain the prepared samples for HPLC. Chromatographic separation was performed using an Acquity UPLC^®^ HSS T3 (150 × 2.1 mm, 1.8 µm, Waters) column maintained at 40°C. The autosampler temperature was maintained at 8°C. Gradient elution of analytes was performed with 0.1% formic acid in water (A2) and 0.1% formic acid in acetonitrile (B2) or 5 mM ammonium formate in water (A3) and acetonitrile (B3) at a flow rate of 0.25 ml/min. Each sample (2 μl) was injected after equilibration. An increasing linear gradient of solvent B (v/v) was used as follows: 0–1 min, 2% B2/B3; 1–9 min, 2%–50% B2/B3; 9–12 min, 50%–98% B2/B3; 12–13.5 min, 98% B2/B3; 13.5–14 min, 98%–2% B2/B3; and 14–20 min, 2% B2-positive model (14–17 min, 2% B3-negative model). The chemical compounds used in this study were obtained from Chengdu Must Bio-Technology Co., Ltd., including rhein, emodin, salvianolic acid B, ferulic Acid, astragaloside IV, astragaloside II, magnolol, rehmannia D, and synephrine. All compounds were tested separately and in mixtures, and the retention time (RT) and mass-to-charge ratio (*m*/*z*) of each compound were recorded. The XCQ formula was tested under the same conditions to determine the presence of related compounds by comparing the RT and *m*/*z* of each peak.

### Animals and Measurements

Twenty-four male Sprague-Dawley rats (age, 6–8 weeks) were obtained from the Laboratory Animal Center, Sun Yat-sen University [Certificate No. SCXK (Guangdong) 2016-0029], and randomly divided into blank, model, and experimental groups. Ethical permission for the conduct of the study was provided by Animal Ethical and Welfare Committee of Guangzhou Forevergen Biosciences (ICUC-AEWC-F1910013, Gaungzhou, China). An STC model was established by intragastric administration of 10 mg/kg loperamide once daily for 7 days in both the model and experimental groups. The mental state, body weight, water intake, feed intake, defecation weight, and fecal moisture content of the rats were observed and recorded. After the model was successfully constructed, the experimental group was administered the XCQ formula 6.83 g/kg (converted according to the body surface area of human and rat) by gavage to observe the physical signs and defecation of the rats and evaluate whether the XCQ formula could improve the defecation of STC rats. C-kit immunofluorescence staining was used to label and identify Cajal cells in the rat colon tissue. The localization and quantification of the two antibodies were determined with an excitation filter under a fluorescence microscope, and ICC expression in the rat colon was determined by overlapping fluorescence.

### Intestinal Propelling Rate of Ink Detection

The intestinal propelling rate of ink was used to evaluating intestinal movement of rats. After fasting for 12 h on the last day of the animal experiment, the rats in each group were given 0.2 g/ml carbon powder by gavage. Thirty minutes later, rats were anesthetized and were sacrificed by abdominal aorta bleeding. The abdominal cavity was dissected and the intestine from the pylorus to the ileocecal part was taken and placed on white paper. The intestinal propelling rate of ink in each group was calculated using the following formula:

$$Intestinal\;propelling\;rate\;=\;\frac{Toner\;travelling\;distance\;}{Total\;intestinal\;length}\;\times 100$$

### Tissue Histological and Immunohistological Study

The rats were then sacrificed and dissected. The proximal colon (1 cm) was collected and fixed in 10% formalin. The colon tissue of rats was dehydrated with graded alcohol and embedded in paraffin. The section thickness was 3–5 μm, and the section was placed on a glass slide. After dewaxing, the sections were stained with hematoxylin and eosin (HE) and sealed with neutral resin. The glass slide was covered, and the pathological structure of the colon was examined. Paraffin sections were baked at 60°C for 1 h, followed by sequential removal of xylene and ethanol. Then, 1% Triton X-100 was added to permeabilize the samples at room temperature for 20 min, and nonspecific binding sites were blocked with 5% goat serum at 25°C for 1 h. Samples were incubated with 3% H_2_O_2_ at room temperature for 15 min to block endogenous peroxidase and then soaked in phosphate-buffered saline (PBS) three times. The specimens were incubated overnight with the primary antibody at 4°C and then incubated with the corresponding secondary antibody for 1 h. The color-rendering target protein of DAB was observed under a microscope to control the color-rendering time. The cells were restained with hematoxylin, and staining was stopped with 1% hydrochloric acid in ethanol. The cells were dehydrated using 75%, 85%, 95%, and anhydrous ethanol. Neutral gum and a small amount of xylene were added to the slides, which were then placed on a ventilation cupboard to dry overnight. Samples were observed and photographed using an Olympus microscope.

### Cell Immunohistochemical Staining

A cell immunofluorescence assay was performed after stable cell growth. The supernatant was discarded and fixed with methanol for 30 min at room temperature, and sheep serum was added for blocking at room temperature for 1 h. The C-kit and IL-21R monoclonal antibodies (1:100) were added overnight at 4°C. The corresponding fluorescent secondary antibodies (1:200) were selected for incubation at room temperature for 1 h and images were recorded (Olympus Corp., Tokyo, Japan).

### Gut Microbiota Analysis

After fecal genomic DNA was extracted, the purity and integrity of the DNA were tested, and the V3–V4 region of the 16S rDNA gene was detected. The sequencing results were analyzed using FastQC software v.0.11.2 to evaluate the quality of the original sequencing data. Sequences without chimerism at the paired ends were linked and clustered into operational taxon units (OTU) with 100% similarity. Alpha and beta diversities were calculated to reflect the richness and abundance of each group of bacteria. LEfSe analysis was used to select the dominant bacteria with significant differences between each group and display them in a cladogram. The KEGG database was used to predict gene content and generate metagenomic functional sequencing tables, and the signal transduction routes that might change were selected for verification.

### Metabolomics Analysis

Fecal and blood metabolomics were measured using the Acquity UPLC system (Waters, Milford, MA, United States) and the Q-Trap 5500 mass spectrometer system (AB Sciex, Framingham, MA, United States). Separation and acquisition were performed using an LC (Nexera X2)-MS (TQ8050) system (Shimadzu City, Japan). The mass spectrum data were analyzed using Chroma TOF software (V 4.3×, LECO) and the LECO-Fiehn RTX5 database. Data were processed to perform peak search and alignment, and matched with the compound spectra database through its primary and secondary spectra to identify the compound we found. The datasets were analyzed via pattern recognition methods using MetaboAnalyst (version 3.2.0). Auto-scaling aims to make each variable comparable to the other. Partial least-squares discrimination analysis (PLS-DA) was used to calculate variable importance for the projection (VIP) scores, the *p*-value was evaluated using the Welch’s t-test, and the BH method was used to calculate the adjusted *p*-value to filter the metabolites. All analyses were performed using R software (version 3.6.0). The metabolites of blood and fecal samples were compared under the following screening conditions: *p* < 0.05 and fold change ≥2 or ≤0.5. The screened differential metabolites were analyzed using principal component analysis (PCA), and the metabolites in fecal samples were enriched using KEGG. MetaboAnalystR (version 3.2.0) was used for enrichment analysis and visualization of the enrichment pathway.

### Tissue RNA-Seq Analysis

RNA was extracted from the colon of freshly frozen rats, and RNA integrity was assessed. After library construction and quality inspection, Illumina sequencing was performed, and 150-bp paired-end readings were generated. After removing low-quality reads with joints and N, the reference genome was compared, and the FPKM of each gene was calculated according to the length of the gene to map the readings of the gene. The DESeq2 software was used to analyze the difference between the two groups, and the Benjamini-Hochberg method was used to adjust the *p*-values to log2 fold change to identify significant differences in the expression of the threshold. The differences between genes were identified by gene ontology (GO), and KEGG enrichment analysis was used to identify the signaling pathways involved.

### Rat ICC Extraction

Rats in the control group fasted for 12 h. Under aseptic conditions, 3 cm of the proximal colon was removed, the mesocolon and blood vessels were stripped, and the mucosal layer of the colon was stripped. The muscle strips were repeatedly rinsed three to four times with D-Hank’s solution containing antibiotics. The muscle strips were cut into pieces, and type I collagenase (1.3 mg/ml) was added for digestion at 37°C for 45 min. The cells were transferred into a 15-ml centrifuge tube, centrifuged at 1,500 rpm for 3 min, and resuspended in 10 ml D-Hank’s solution. The cells were centrifuged at 1,500 rpm for 3 min and resuspended in a 10 ml DMEM/F12 1:1 medium. Large tissues and cell clumps were successively removed through 200 and 100 mesh screens, and the cells were suspended in DMEM/F12 1:1 medium containing 10% FBS. Cells were seeded in 6-well plates and treated with TC. The samples were cultured in an incubator at 37°C and 5% CO_2_, and the morphological characteristics were observed during the growth period.

### Human ICC Extraction

The full-thickness tissue of the non-cancerous end of the human colon was removed, and the mucosal layer was stripped off and rinsed repeatedly with D-Hank’s solution containing antibiotics. The muscle strips were cut and type III collagenase (2 mg/ml) was added. The strips were digested at 37°C for 60 min and centrifuged at 1,500 rpm for 3 min. The cells were resuspended in 10 ml M199. Cells were successively screened to remove clumps, and the cells were suspended in an M199 medium containing 10% FBS and 5 ng/ml stem cell factor (SCF). Cells were seeded in 6-well plates and treated with TC. Cells were cultured in an incubator at 37°C and 5% CO_2_. Cells of 10^5^ cells were cultured and mixed with CD117 magnetic beads (Miltec, 130-019-332, Exxon Beijing Technology Co., Ltd.). The c-kit-positive interstitial cells of the Cajal (ICC) were screened by magnetic column separation.

### Cell Viability

The effect of XCQ formula on cell viability was tested using the Cell Counting Kit-8 (CCK-8) assay (Nanjing KeyGen Biotech Co., Ltd., Nanjing, Jiangsu, China). Briefly, cells were seeded into 96-well plates (5 × 10^3^ cells/well) and treated with Butamben or Loperamide. After incubation for 12, 24, 48, or 72 h, 20 µl of CCK-8 solution was added to each well. Absorbance was measured using a 96-well plate reader (Thermo Fisher Scientific, Waltham, MA, United States) at a wavelength of 450 nm after incubation for 4 h.

### Cell Cycle Analysis

ICC cells were incubated with Butamben or Loperamide for the indicated times, after which the cells were collected, washed, and fixed with 66% cold ethanol at 4°C overnight. After the 66% ethanol was removed, cells were washed with PBS and stained with propidium iodide (BD Biosciences, Franklin Lakes, NJ, United States). Cell cycle distribution analysis was performed using a BD FACSCanto II flow cytometer (Franklin Lakes, NJ, United States).

### Cell Apoptosis Analysis

An annexin V-FITC/PI dual staining kit (Nanjing KeyGen Biotech Co., Ltd., Nanjing, China) was used to detect cell apoptosis. Cells were treated with or without specific drugs for the indicated times, after which the cells were collected, washed, and suspended in 500 µL of binding buffer, and mixed with 5 µL of annexin V-FITC and 5 µL of propidium iodide. The solutions were incubated in the dark at room temperature for 15 min before analysis. Cell apoptosis rates were measured using a BD FACSCanto II flow cytometer. Annexin V-FITC-positive and annexin V-FITC-plus PI-positive cells were reported as undergoing apoptosis.

### Western Blot Analysis

Cells were lysed in lysis buffer supplemented with protease and phosphatase inhibitors (Nanjing KeyGen Biotech Co., Ltd.). The Bio-Rad assay kit (Bio-Rad Laboratories, Hercules, CA, United States) was used to determine protein concentrations. Protein electrophoresis was performed on 8%, 10%, and 15% SDS polyacrylamide gels, and proteins were transferred to PVDF membranes, blocked with 5% non-fat dried milk at room temperature for 2 h, and then probed with the appropriate primary antibody overnight at 4°C. After the membranes were washed with 0.1% TBS-T and probed with the corresponding secondary antibody at room temperature for 2 h, protein bands were observed via enhanced chemiluminescence detection.

### Statistical Analyses

All reported results are representative of three independent experiments, and the data are expressed as mean ± standard deviation. For statistical analyses, a one-way analysis of variance followed by Tukey’s test was performed using GraphPad Prism (San Diego, CA, United States). Differences were considered statistically significant at a *p*-value < 0.05.

## Results

### HPLC Chromatogram of the XCQ Formula

**FIGURE 1:**
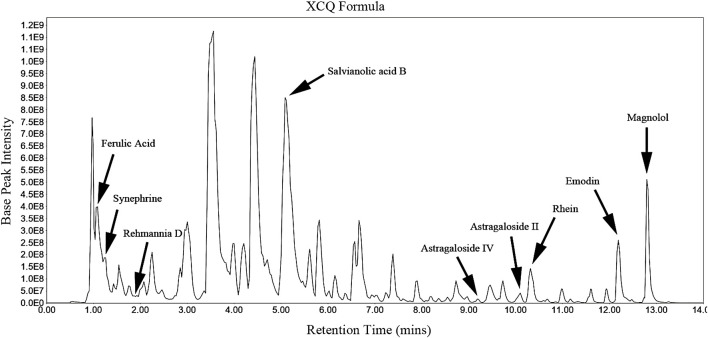
HPLC chromatogram of the characteristics of the Xiao Chengqi (XCQ) formula: The chromatogram of bioactive active compounds derived from the XCQ formula, including Rhein, emodin, salvianolic Acid B, ferulic acid, astragaloside IV, astragaloside II, magnolol, Rehmannia D and synephrine.

The HPLC chromatogram of the XCQ formula is shown in Figure 1, and the characteristics of the compounds are shown as the RT and *m*/*z*. The XCQ formula contains a wide range of chemical compounds, including the main components of seven medicinal materials in the XCQ formula, such as rhein (RT, 1.11 min; *m*/*z*, 197.8074), emodin (RT, 12.19 min; *m*/*z*, 269.0456), salvianolic acid B (RT, 5.14 min; *m*/*z*, 717.1445), ferulic acid (RT, 10.33 min; *m*/*z*, 283.0246), astragaloside IV (RT, 9.2 min; *m*/*z*, 829.4572), astragaloside II (RT, 10.09 min; *m*/*z*, 871.4666), magnolol (RT, 12.8 min; *m*/*z*, 265.1231), rehmannia D (RT, 1 min; *m*/*z*, 731.2247), and synephrine (RT, 1.12 min; *m*/*z*, 197.8074).

### XCQ Formula Improved Defecation in SD Rats With the STC Model

**FIGURE 2:**
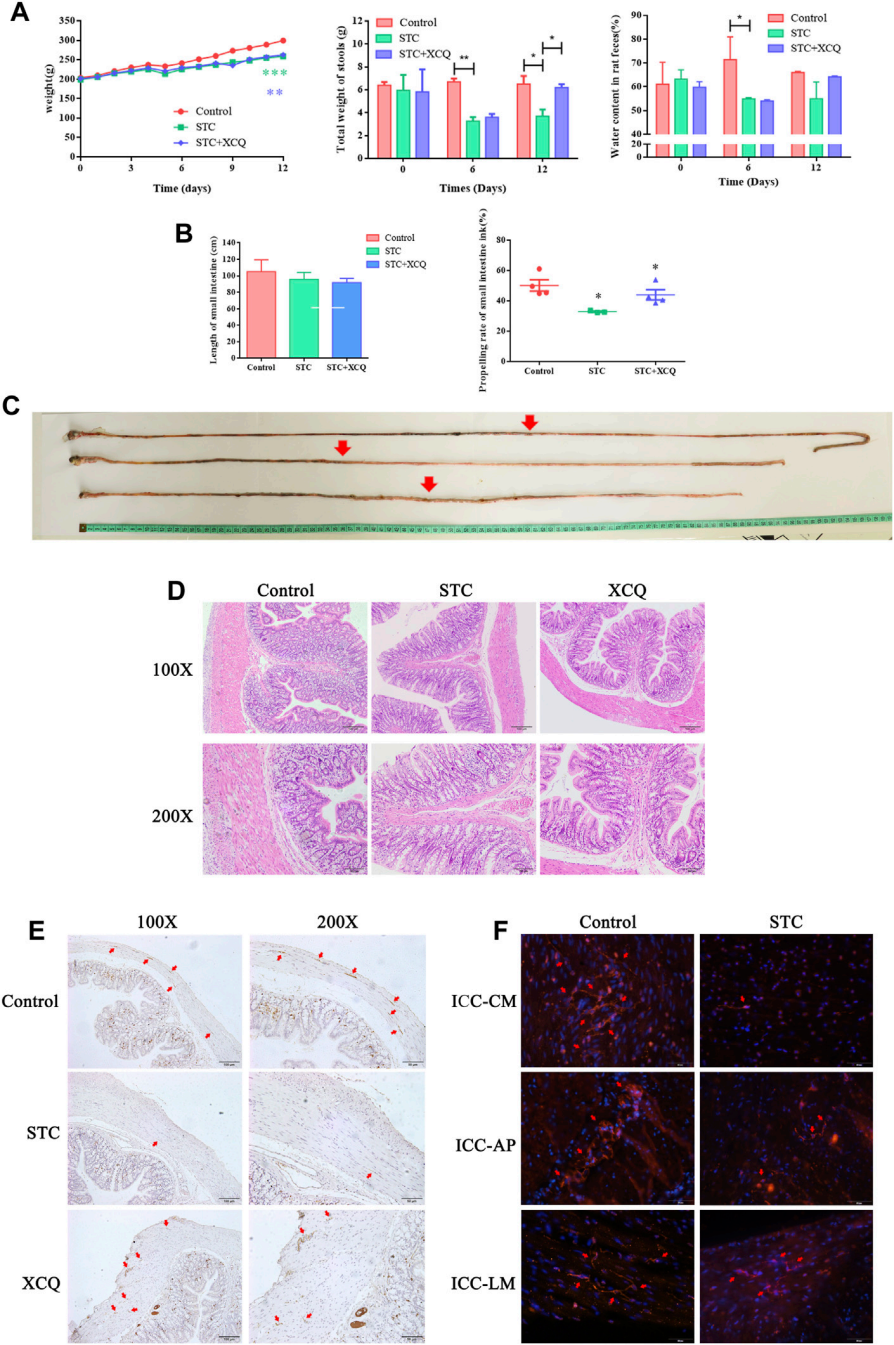
Xiao Chengqi (XCQ) formula can improve defecation in slow transit constipation (STC) rats. **(A)** The physical signs of rats in each group include stable weight growth, and the weight growth rate of rats in the normal control group is faster. The data of each group are compared with the control group. The fecal weight and water content in the STC group are significantly lower than those in the control group, and the defecation weight in the XCQ group is significantly higher than that in the STC group, difference between Control and STC groups was showed as green asterisk, difference between Control and XCQ groups was showed as purple asterisk, *p < 0.05, **p < 0.005; **(B)** The small intestine propulsion rate decreased in the STC group; **(C)** The intestinal propelling length of ink in each group; **(D)** HE staining of three groups of rat colon; **(E)** The expression of c-kit in muscle layer in the XCQ formula group was significantly higher than that in the STC group, the expression of c-kit was marked as red arrow; **(F)** expression changes in c-kit in the colon tissue of STC patients, the expression of c-kit was marked as red arrow.

As shown in Figures 2A–C, loperamide can induce SD rats to significantly reduce defecation moisture content and fecal weight, whereas the XCQ formula can increase the defecation weight of STC rats. It could be observed that the intestinal propelling rate decreased significantly in the STC group, and the STC rat model was established successfully (Figure 2C). Moreover, the XCQ formula can improve loperidol-induced transport dysfunction.

### XCQ Formula Improved c-kit Expression in SD Rats With the STC Model

HE staining showed that the XCQ formula reduced inflammatory cell infiltration in the colon tissue of STC rats and reduced edema and thickening of the muscular mucosa (Figure 2D). Tissue immunofluorescence staining showed that the XCQ formula reversed the loperamide-induced reduction of ICC in the colon of STC rats (Figure 2E). Full-thickness distal colon tissue of surgical specimens from patients with colon tumors without a history of obstruction was used as the control group, and full-thickness expanded colon tissue from patients with STC was used as the STC group. The expression of c-kit in paraffin sections from both patients was detected by tissue immunofluorescence staining, indicating that c-kit expression in the STC group was generally lower than that in the control group. The c-kit expression (ICC-AP) in the surrounding nerve plexus was significantly reduced (Figure 2F).

### XCQ Formula Can Increase the Abundance of *Roseburia* spp. in STC Rat Feces

**FIGURE 3:**
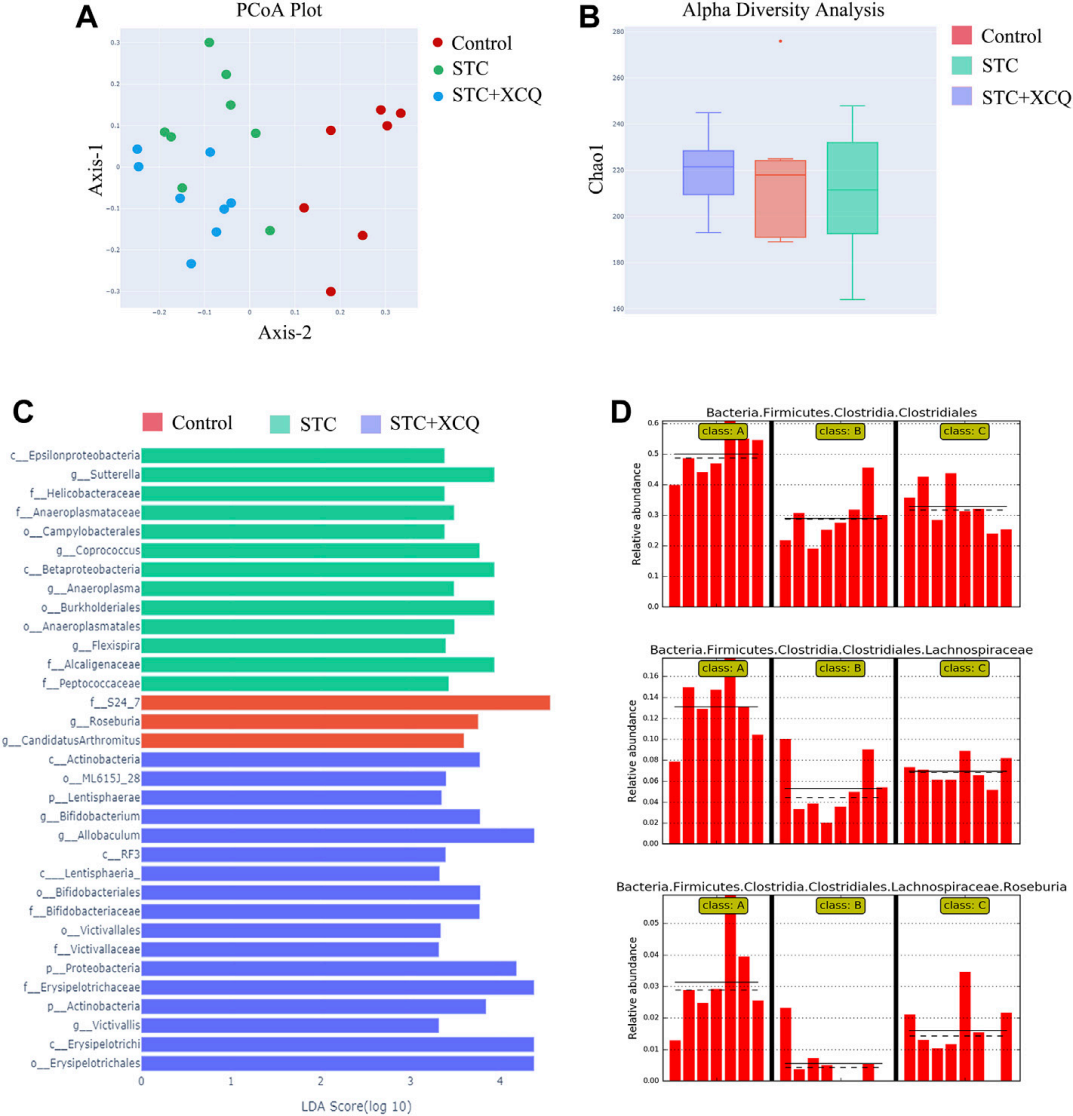
The effect of Xiao Chengqi formula on intestinal flora of slow transit constipation (STC) rats. **(A)** PCoA scatter plot showed that there were significant differences in intestinal flora abundance among the three groups, and the flora abundance of the STC group was significantly lower than that of the control group; **(B)** Chao1 index showed that there was no significant difference in community richness among the feces of rats in the blank group, STC group, and experimental group; **(C)** LEfSe analysis showed that *Roseburia* spp. were the representative bacteria with significant differences among the three groups; **(D)** Based on the order, family and species levels, it can be seen that the abundance of *Roseburia* spp. in the STC group decreased significantly, and the abundance increased significantly after XCQ treatment.

To explore the potential involvement of the gut microbiome in the effects of STC treatment with the XCQ formula, we analyzed the community structures of the gut microbiota in loperamide-induced STC rats treated with the XCQ formula using 16S rRNA microbial profiling analysis. A total of 2,537 OTUs were identified in the abovementioned five groups, including 1,029 OTUs in the control group, 1,082 OTUs in the STC group, and 1,098 OTUs in the XCQ group. Among these, 282 OTUs were identified in both the control and STC groups, and 304 OTUs in the control group were also identified in the XCQ group. Through beta diversity analysis using PCoA based on the unweighted UniFrac index, we showed that the control group was closer to XCQ than the STC group, indicating the high similarity in microbiota community structure between the XCQ and control groups (Figure 3A). The rarefaction curves flattened out in correlation with the increases in Chao1 species indices, indicating preferable sequencing depth and high coverage of species. Alpha diversity analysis showed that microbial abundances in the STC and control groups were not significantly different (Figure 3B). We analyzed the alterations in gut microbiota profiles induced by XCQ treatment in STC rats. At the phylum level, compared with the control group, the abundance of *Actinobacteria.*, *Lentisphaerae.*, and *Proteobacteria* in the STC group were significantly increased, whereas the abundance of *Cyanobacteria* was significantly decreased, showing no significant difference between the experimental and STC groups. Meanwhile, at the class level, compared to the control group, in the STC group, *Betaproteobacteria* and *Epsilonproteobacteria* abundance significantly increased in the STC group. Moreover, the abundances of *C0D-2*, *Clostridia*, and *Mollicutes* significantly decreased. Compared with the STC group, the abundance of *Clostridia* and *Mollicutes* in the model group significantly increased, while the abundance of *Gammaproteobacteria* and *RF3* significantly decreased (Figure 3C). Moreover, the relative abundance of *Roseburia* spp*.* was also lower in the STC group than in the control group but was elevated by XCQ treatment (Figure 3D). These results indicate that changes in the gut microbiota community structure during STC pathogenesis can potentially be reversed by XCQ treatment.

### Effects of the XCQ Formula on the Metabolites Assessed Using STC Rat Feces

**FIGURE 4:**
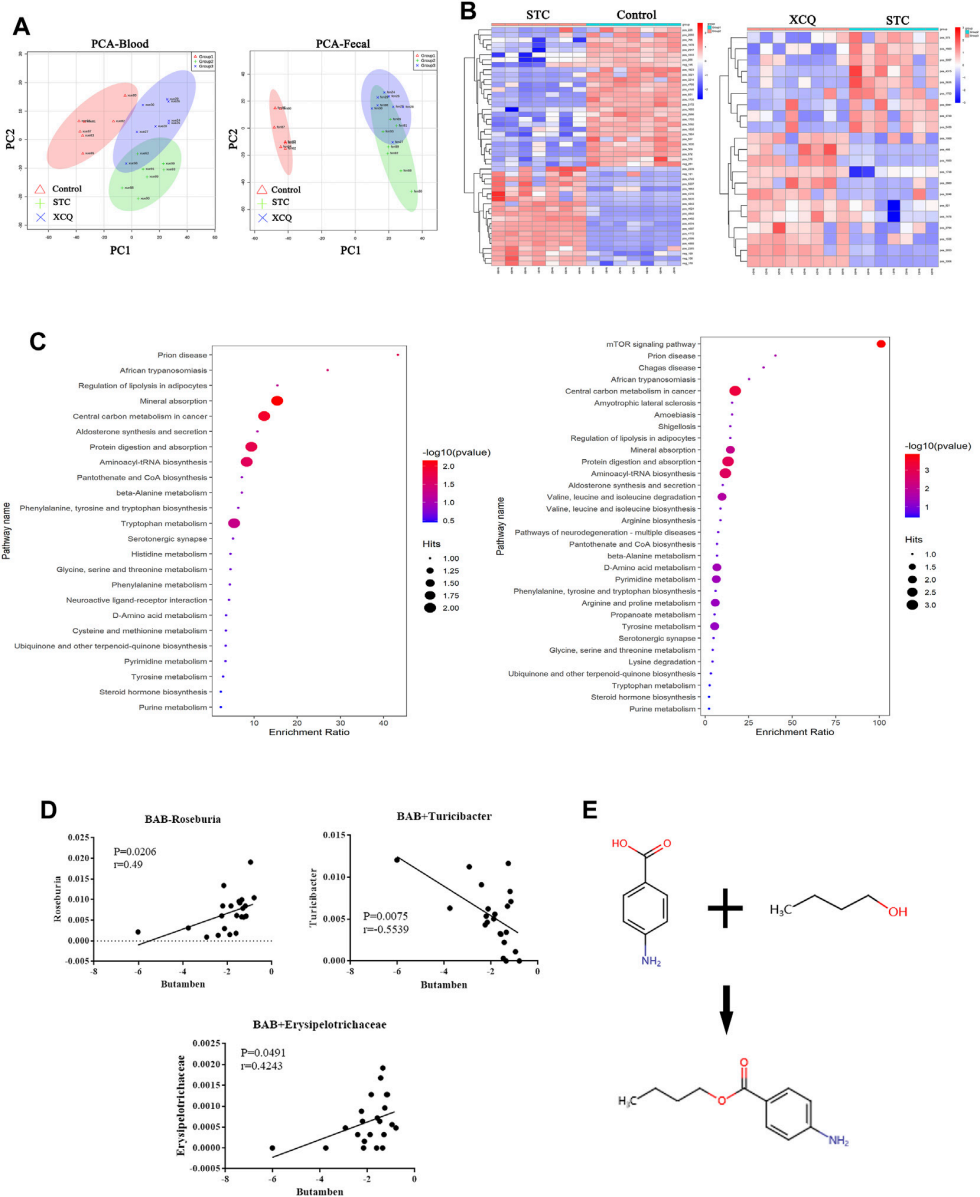
The effect of the Xiao Chengqi (XCQ) formula on metabolites in slow transit constipation (STC) rats. **(A)** PCA analysis of component differences between groups showed that the metabolites in feces (left) and serum (right) of STC rats were significantly different from those in the control group, and the use of the XCQ formula could significantly affect the metabolism of STC rats; **(B)** from the metabolite heat map (left is the control group and STC group, right is the STC group and experimental group), it can be seen that the contents of n-methyltyramine, butyl aminophenyl and 5-deoxy-5-methylthioadenosine were significantly different among the three groups; **(C)** The KEGG enrichment analysis showed as bubble chart between each two groups; **(D)**The relationship between BAB and the abundance of *Roseburia* spp*.* (Pearson correlation = 0.501); **(E)** The compound structure of BAB.

Samples from the control, STC, and XCQ groups were characterized and compared. A total of 1016 molecular features in fecal samples and 851 molecular features in blood samples were obtained and subjected to statistical analysis using MetaboAnalyst (version 3.2.0). PCA revealed that XCQ could affect metabolism in rats (Figure 4A). Features with a *p*-value < 0.05 and fold change ≥2 or ≤0.5 were considered the most significant metabolites and were visualized through a heatmap (Figure 4B). A total of 93 significant metabolites were identified in both the control and STC groups, and 21 significant metabolites in the XCQ group were identified in the STC group. Among the differential metabolites, N-methyltyramine, butyl aminobenzene, and 5-deoxy-5-methionine in the STC group were significantly decreased, whereas the XCQ formula significantly upregulated the levels of these metabolites.

To identify biological patterns based on metabolomics data in feces, MetaboAnalyst R (version 3.2.0) was used to perform pathway analysis using the KEGG metabolic library. The perturbed metabolic pathways in the fecal samples were shown in Figure 4C. Mineral absorption, central carbon metabolism in cancer, protein digestion and absorption, prion disease, and aminoacyl-tRNA biosynthesis were significantly enriched in STC rats compared with controls. L-tryptophan, L-methionine, and tryptamine levels were increased in protein digestion and absorption and aminoacyl-tRNA biosynthesis. Tryptamine and L-tryptophan were also enriched in phenylalanine, tyrosine, and tryptophan biosynthesis and neuroactive ligand-receptor interactions.

Furthermore, we analyzed the correlation between the metabolites and differential flora and found that the content of BAB, a fecal metabolite, was positively correlated with the relative abundance of *Roseburia* spp. (Figure 4D). BAB is an amino acid ester formed by formal condensation of the carboxyl group of 4-aminobenzoic acid with the hydroxyl group of butane-1-alcohol (Figure 4E). Para-aminobenzoic acid, an essential nutrient for some flora, can promote the growth of intestinal flora and maintain tissue activity through its antioxidant effect. Therefore, we believe that aminobutyl may promote the colonization of *Roseburia* spp. and underlie the intestinal peristalsis-promoting effects of the XCQ formula on STC in rats.

### RNA-Sequencing

**FIGURE 5:**
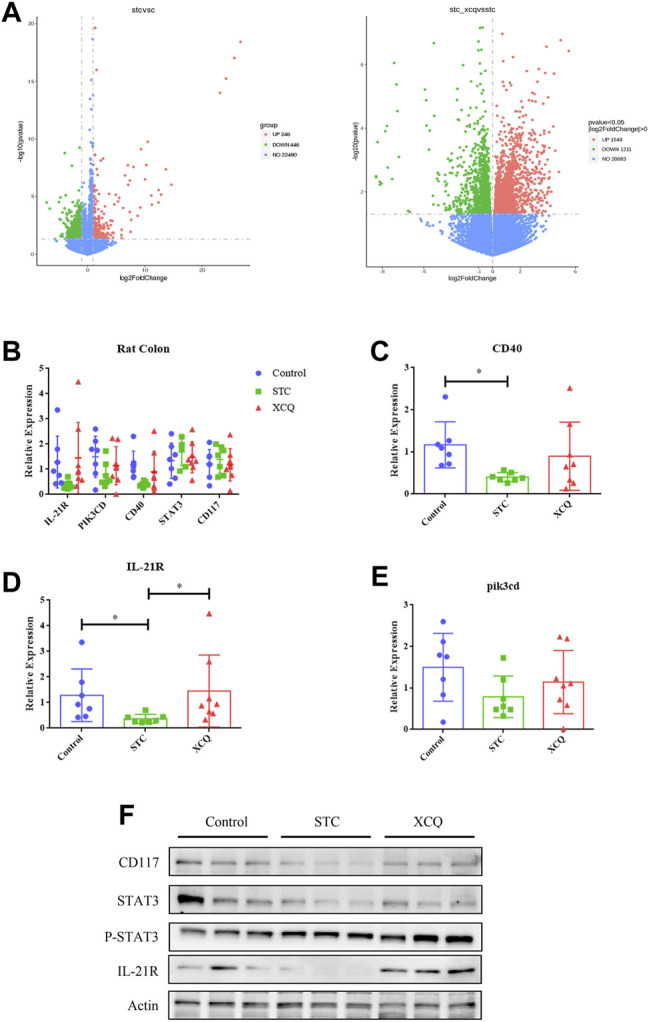
The effect of the Xiao Chengqi formula on slow transit constipation (STC) rats at the colonic transcriptome level. **(A)** Gene expression among the three groups: STC group versus the control group (left) and experimental group versus the control group (right), the descending gene (green), ascending gene (red) and no significant difference gene (blue); **(B)** the expression of IL-21R, CD40, and pik3cd was significantly different among the three groups, *p < 0.05; **(C–E)** The protein expression levels of CD117, IL-21R and their downstream STAT3 activation between groups; **(F)** the expression of CD117, IL-21 and its downstream STAT3 activation in rat colon.

The RNA profiles of the colon tissues of rats in the control, STC, and XCQ groups were determined using RNA-seq. A total of 24,956 genes were identified, which were expressed in at least one sample. The number of expressed genes was 23,085 in the control group, 23,238 in the STC group, and 23,510 in the XCQ group. To determine the differentially expressed genes (DEGs), a *p*-value < 0.05, which was detected by pairwise comparisons between the model and control, XCQ, and STC groups, was used as the screening criterion for gene expression in the three groups. Overall, 1,238 upregulated and 1,246 downregulated DEGs were identified in the STC vs control groups and 1,549 upregulated and 1,212 downregulated DEGs were identified in the XCQ vs STC groups (Figure 5A). Based on the criteria of log|FC| > 1 and *p*adj < 0.05, 2,760 genes, including 145 upregulated and 102 downregulated genes, were identified between the STC and XCQ groups. We obtained differences in the expression of IL-21R, PIK3CD, CD40, and other genes through tissue RNA-seq comparison. The interactions between the DEGs were analyzed using functional enrichment with the KEGG pathway, and GO enrichment analysis showed that the DEGs were mainly associated with signal transduction, receptor, and ligand connection between cells (including IL-6/IL-6R, IL-21/IL-21R) and various signal-related pathways, such as the NF-kappa B signaling and TGF-β signaling pathways. Furthermore, we validated the changes in CD117, IL-21R, and their downstream molecules at the protein level (Figures 5B–F). Therefore, we believe that the regulatory effect of XCQ on CD117 expression and intestinal peristalsis may be related to the inflammatory environment, such as IL-21R.

### Loperamide Can Induce Apoptosis of ICC in Rat Colon

**FIGURE 6:**
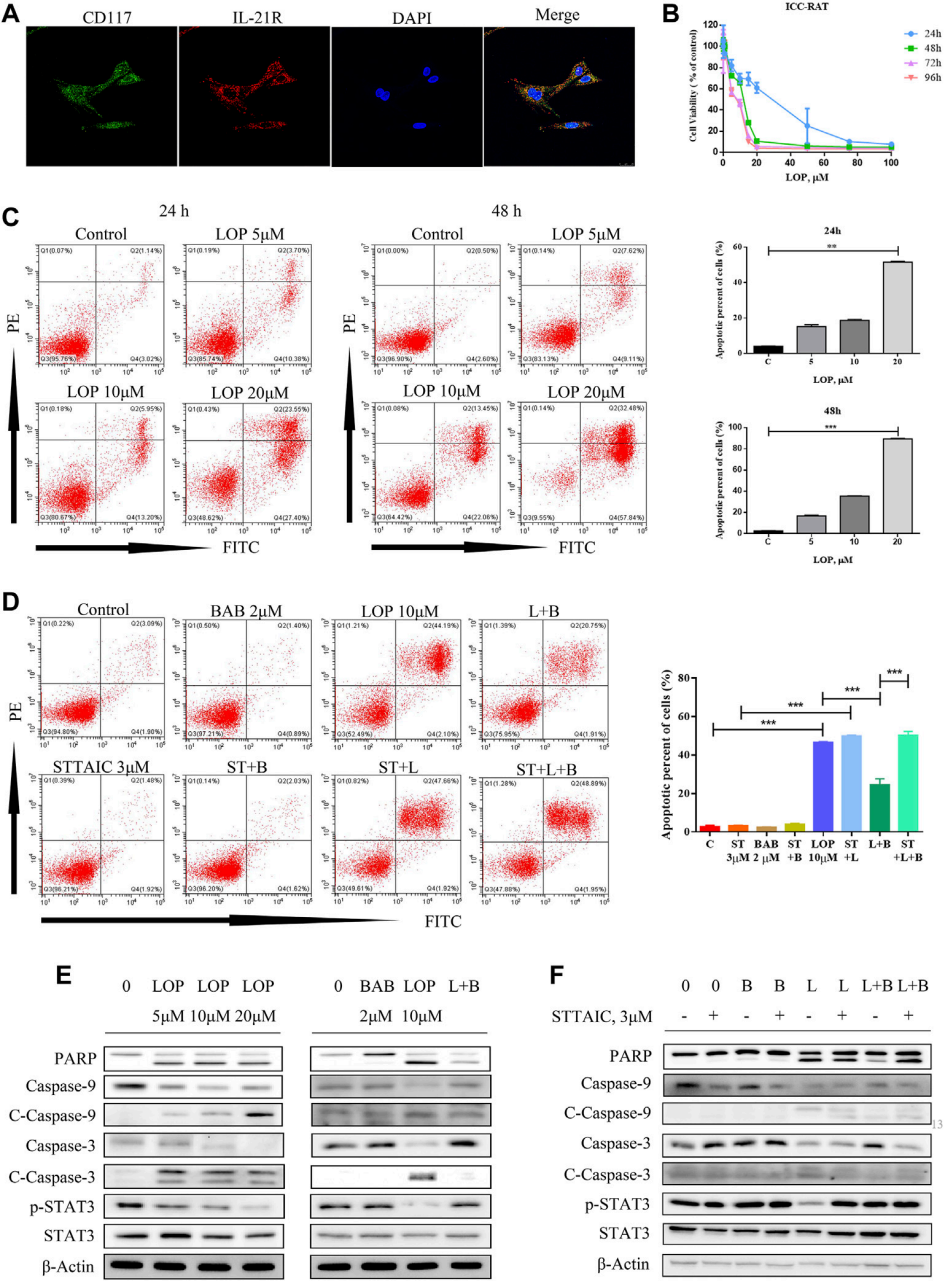
The effect of loperamide on interstitial cells of Cajal (ICC) in rats. **(A)** Immunofluorescence was used to identify the expression of IL-21R (red) and c-kit (green) in ICC; **(B)** when cells were treated with different concentrations of loperamide, it can be seen that loperamide can induce ICC apoptosis in a time-concentration dependent manner, and the IC50 at 48 h is about 12 μM; **(C)** flow cytometry was used to detect apoptosis; **(D)** flow cytometry was used to detect the effect of butyl aminobenzene (BAB) on loperamide induced apoptosis; **(E)** simulated cells were treated with different concentrations of BAB; **(F)** Western blotting was used to detect caspase pathway-related proteins. *p < 0.05, **p < 0.005, ***p < 0.001.

To clarify its regulatory mechanism, we extracted primary colonic ICC from rats and identified the expression of IL-21R in ICC using immunofluorescence (Figure 6A). The cells were treated with loperamide at different concentrations, which induced ICC apoptosis in a time–dose-dependent manner, with an IC50 of approximately 12 µM at 48 h (Figures 6B,C).

### BAB Showed an Anti-Apoptosis Effect in the ICC of Rats

To clarify the influence of BAB, the post-metabolite screened in the previous experiment, on ICC, rats were treated with different loperamide concentrations to simulate the ICC model, indicating that loperamide can induce time–dose-dependent apoptosis of ICC in rats. To observe the effect on cell survival, 2 μM BAB was added 24 h after modeling. The annexin V-FITC/PI staining test and WB results showed that BAB can protect ICC activity and inhibit the caspase-dependent apoptotic response induced by ICC (Figures 6D–F). Further more, the anti-apoptotic effect of BAB was abolished with the usage of STTAIC, which indicated that BAB could modulate STAT3 pathways to achieve the protective effect on ICCs (Figures 6D,F).

### BAB Inhibits Loperamide-Induced Apoptosis in the Human Colon ICC

**FIGURE 7:**
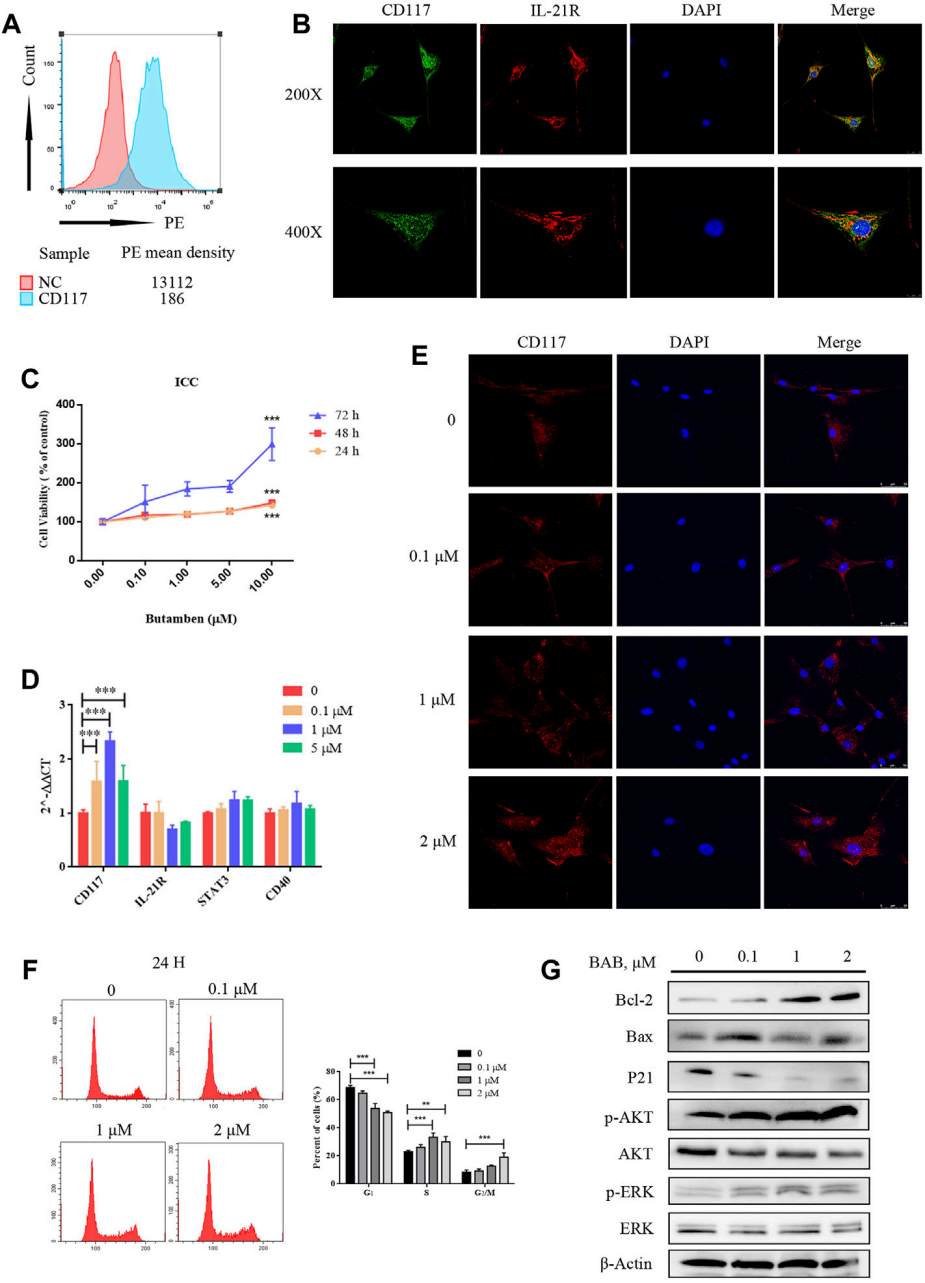
The effect of BAB on human colonic interstitial cells of Cajal (ICC). **(A)** Flow cytometry showed the expression of c-kit in pre purified (red) and post purified (blue) cells; **(B)** CCK-8 showed that loperamide could also induce concentration-dependent apoptosis in ICC; **(C)** immunofluorescence identified the expression of IL-21R (red) and c-kit (green) in ICC; **(D)** qPCR detected the expression changes of related genes; **(E)** Cell immunofluorescence staining showed the expression of c-kit in human colonic ICC after treatment with different concentrations of BAB; **(F)** Cell cycle changes after treatment with BAB, the statistical analysis is listed on the right; **(G)** The expression of cell cycle-related proteins and the activation of Akt and ERK pathways were detected by Western blotting. **p < 0.01, ***p < 0.001.

Human colon ICCs were extracted and purified using magnetic beads, and the expression of c-kit and IL-21R in cells was verified by flow cytometry (Figure 7A). The expression of IL-21R in the cells was verified by immunofluorescence (Figure 7B). The CCK-8 results showed that the number of ICCs increased after treatment with 0–10 μM BAB for 72 h (Figure 7C). qPCR, flow cytometry, and caspase pathway protein levels were detected in cells treated with loperamide in a concentration–time-dependent manner. The addition of BAB inhibited further apoptosis of ICC to some extent (Figures 7D–G).

### BAB Protects the Survival of ICC in the Human Colon by Interfering With IL-21R Activity on the Cell Surface and Regulating Downstream Signaling Pathways

**FIGURE 8:**
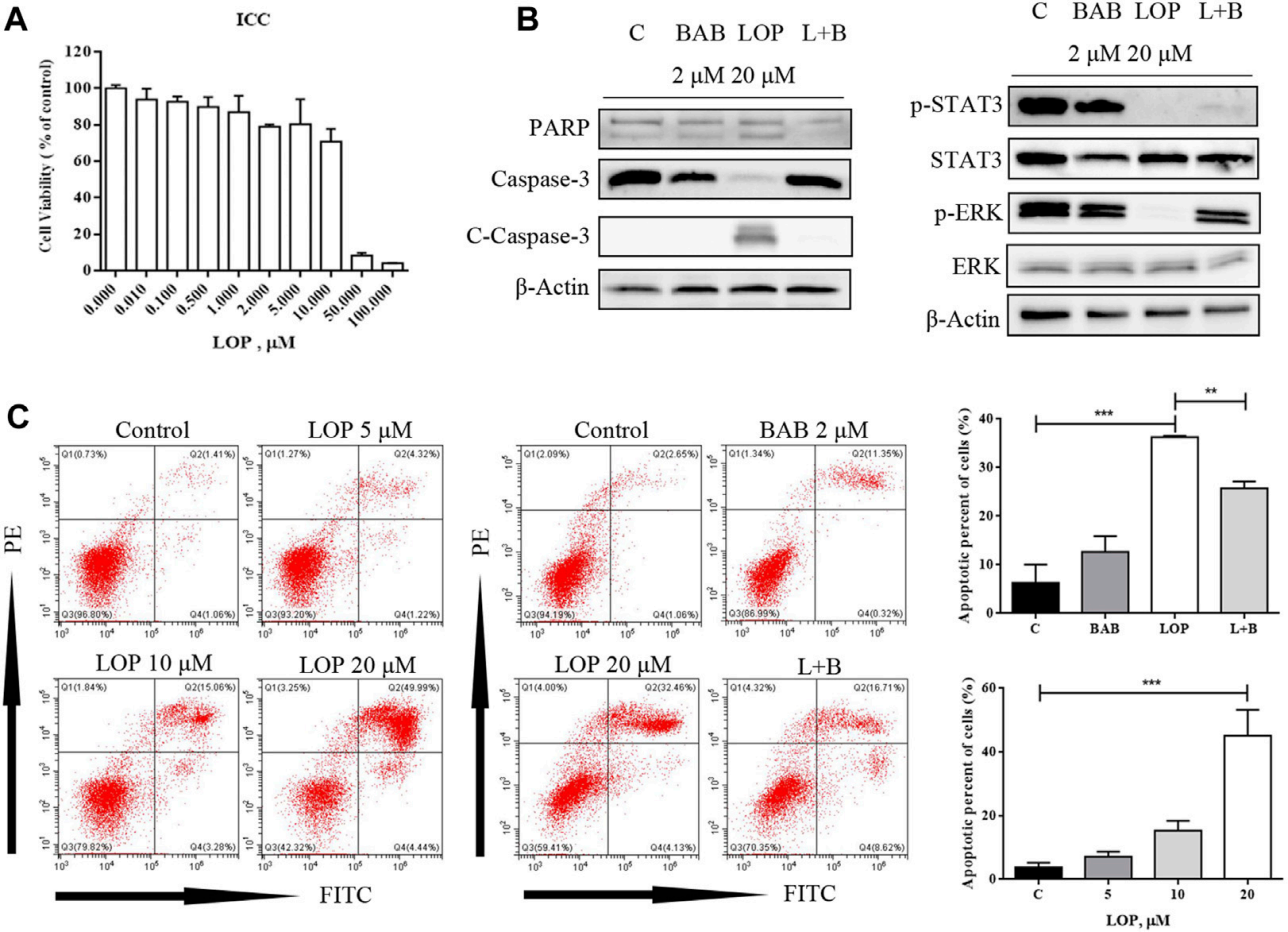
BAB can reverse the apoptosis of interstitial cells of Cajal (ICC) induced by loperamide. **(A)** CCK-8 method showed that loperamide could also induce concentration-dependent apoptosis in ICC at 24 h; **(B)** the expression of apoptosis-related caspase pathway proteins and the activation of STAT3 and ERK were detected by Western blotting; **(C)** apoptosis was detected by flow cytometry. **p < 0.005, ***p < 0.001.

**FIGURE 9:**
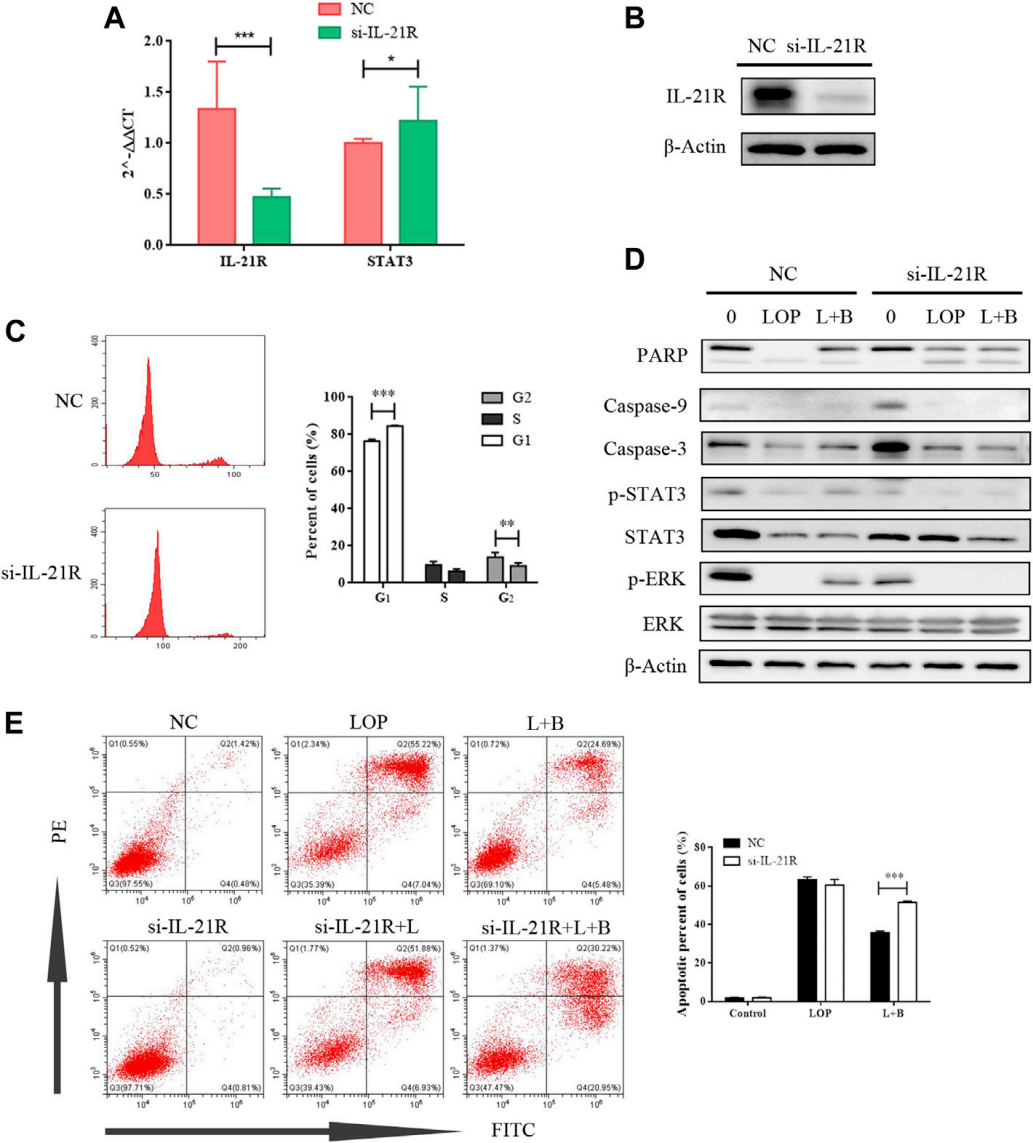
The cell activity of interstitial cells of Cajal can be inhibited by knocking down IL-21R. **(A)** QPCR showed that IL-21R was knocked down at the transcriptional level; **(B)** the expression of IL-21R on the cell surface decreased after IL-21R siRNA transfection; **(C)** the changes in the cell cycle were detected by flow cytometry. *p < 0.01, ***p < 0.001. **(D)** the WB images of caspase pathway and the phosphorylation level of STAT and ERK. **(E)** apoptosis was detected by flow cytometry. **p < 0.005, ***p < 0.001.

According to a literature review, both the JAK/STAT3 and MEK/ERK pathways can be regulated by IL-21R. In this study, the expression of IL-21R in ICC was previously verified. After treatment with LOP, the cell viability significantly decreased in a concentration-dependent manner (Figure 8A). WB images showed that the caspase pathway was activated and p-STAT3 and p-ERK were both upregulated (Figure 8B). It seems that both the STAT3 and ERK pathways were regulated. Moreover, it was found that BAB reduced the apoptosis-induced effect of LOP in human ICCs (Figure 8C). Thus, we hope to further clarify the underlying mechanism of BAB in protecting ICCs and whether the mechanism by which BAB protects ICC is related to IL-21R and STAT3 and ERK pathways. We knocked down IL-21R and verified this by quantitative real-time PCR and WB analysis (Figures 9A,B). The results showed that the cells were even slower to proliferate, and the cells were blocked in the G_1_/S phase (Figure 9C). WB images showed that p-STAT3 and p-ERK were also inhibited at the protein level after IL-21R silencing, BAB lost its protective effect on ICC, the images of PARP, caspase-3, and caspase-9 were almost the same as those in the LOP group after BAB treatment, and the apoptotic rate was not reduced (Figures 9D,E). Therefore, we suggest that aminophenyl affects cell survival by activating IL-21R.

### BAB Can Reverse LOP-Induced ICC Apoptosis Through Activation of the ERK/STAT3 Signaling Pathway

**FIGURE 10:**
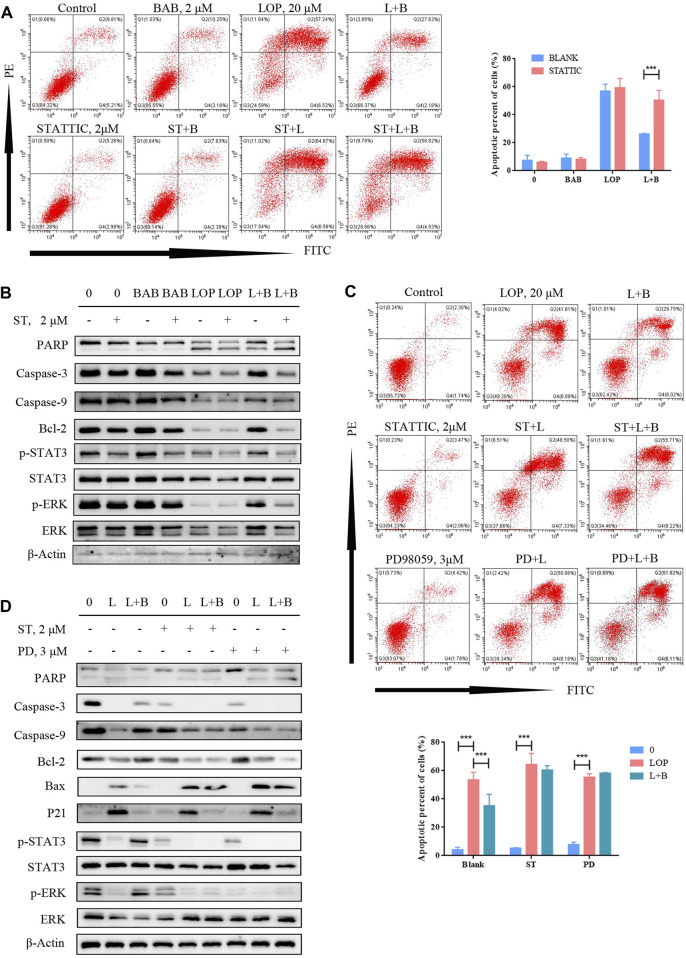
BAB can reverse lop induced interstitial cells of Cajal apoptosis. **(A)** Flow cytometry was used to detect the apoptosis after STAT3 was inhibited and activated; **(B)** Western blot was used to detect the apoptosis after STAT3 was inhibited and activated; **(C)** flow cytometry was used to detect the apoptosis after STAT3 or ERK was inhibited; statistical analyses were shown below; **(D)** PD98059 and STATTIC were used to inhibit the activate of STAT3 and ERK. The expression of proteins associated with caspase pathway, cell proliferation and the activation of STAT3 and ERK were detected by WB.

At the protein level, we treated the cells with STAT3 inhibitors to inhibit STAT3 phosphorylation, and the anti-apoptotic effect of BAB was abolished (Figure 10A). While p-STAT3 was significantly inhibited, the caspase pathway was still activated, and p-ERK expression was not significantly reduced by BAB treatment (Figure 10B). Furthermore, when ERK phosphorylation was inhibited and apoptosis was not reduced, while p-STAT3 expression was significantly decreased (Figures 10C,D). This indicates that ERK is upstream of STAT3 in IL-21R regulated cell survival and BAB activates the ERK/STAT3 signaling pathway through the intervention of IL-21R on the surface of ICCs and reverses the apoptosis of ICCs induced by loperamide.

## Discussion

Chronic constipation is a clinically common anorectal condition. Currently, the prevalence of constipation in the United States is approximately 16%, of which 46%–74% are STC. STC is characterized by prolonged colon passage, decreased motility, and normal pelvic floor function and is more common in older adults (Christopher et al., 2020). The etiology of STC is complex, and its pathogenesis has not yet been fully defined.

The academic theory of TCM is quite different from that of Western medicine. The bioactive components in TCM are considered mainly to be compounds with high molecular weight. Their bioavailability and drug properties cannot be absorbed and utilized by the human body as they would be digested. Thus, it is difficult to reconcile the clinical effectiveness of TCM from the perspective of Western pharmacology. Therefore, we explored the mechanism of the XCQ formula in the treatment of chronic constipation through both a holistic view of TCM and detailed pathological changes from Western medicine theory. From the perspective of Western medicine, the intestinal flora and immune system may clarify the holistic view of TCM and connect both theories.

Recently, the intestinal microenvironment has attracted much attention in the study of the pathogenesis of gastrointestinal motility disorders, such as STC, in which changes in the structure of intestinal flora and related metabolites play a vital role (Eamonn and Robin, 2016). Many studies have shown that the digestive ability of intestinal flora for dietary fiber and interaction between fiber and microorganisms can change the shape, weight, and colonic transport capacity of feces, while excessive short-chain fatty acids and lack of bile acids can lead to decreased peristalsis and colonic secretion (Tigchelaar et al., 2016). Moreover, during the interaction between intestinal flora and intestinal contents, the number of well-known probiotics, such as bifidobacteria and lactic acid bacteria, significantly decreased in some patients with chronic constipation (Konstantinos et al., 2014). In patients with chronic constipation, compositional changes in the intestinal flora are independent risk factors for constipation. *Bacteroides* account for the majority of colonic flora in patients with constipation, while the proportion of Chlamydomonas decreases, and colonic transport function is significantly related to the diversity of intestinal flora, short-chain fatty acids in feces, and plasma acetate content (Kang et al., 2015).

Increasing evidence has shown that TCM can improve defecation by improving the composition of intestinal microbiota, producing useful metabolites, increasing the secretion of the intestinal mucosa, reducing water reabsorption, and promoting intestinal peristalsis (Xu et al., 2019). TCM compounds used by domestic TCM hospitals often achieve a good curative effect in the treatment of chronic constipation anecdotally, but this has not been popularized owing to the lack of clarity in its mechanism.

It is believed that histopathological changes, such as intestinal nervous system lesions, ICC, smooth muscle, and changes in intestinal neurotransmitters, are important factors that induce STC. Studies have found that, with age, there are fewer intestinal intermuscular ganglia and more neurodegenerative changes, which may cause dysfunction in colonic dynamics. Moreover, various gastrointestinal neurotransmitters, such as abnormal secretion of vasoactive peptides and abnormal expression of serotonin receptors, may be associated with the incidence of STC (Gary and Jill, 2013). The smooth muscle is the final effector of gastrointestinal activities. In patients with STC, colonic smooth muscle can have lesions of varying degrees, including smooth muscle fiber atrophy and fibrosis of varying degrees, which will lead to a decline in the contractile function of smooth muscle (Charles et al., 2001). Cajal stromal cells, known as pacing cells of the gastrointestinal tract, can regulate the contraction of the intestinal smooth muscle. The number and phenotypic abnormalities of Cajal cells are closely related to the occurrence and development of STC. The c-kit (CD117) receptor is widely expressed in the cytoplasm and membrane of ICC and regulates MAPK, PI3K/Akt, Wnt, and other signaling pathways by binding to its natural ligand SCF, which determines the differentiation and function of ICC (Gibbons et al., 2007). The amount of ICC in the colon tissue is often reflected in the expression level of the c-kit protein (Goran et al., 2010).

The XCQ formula was reformed based on the XCQ decoction, a representative prescription of “Treatise on Febrile Diseases.” In this study, we added *Astragalus*, *Angelica*, *Rehmannia glutinosa*, and *Salvia miltiorrhiza* based on the XCQ decoction, making it more suitable for patients with STC. In previous studies, the XCQ formula was used in patients with colon anastomosis, and the results showed that the recovery time of intestinal function in patients with colon cancer after nasal feeding using the XCQ formula was significantly shorter than that in patients who received conventional postoperative treatment. Based on anecdotal clinical application experience, the XCQ formula can significantly improve defecation in patients with STC. To date, the detailed mechanism of action of the formula and the involvement of microflora have not been clarified. To solve these problems, an STC rat model was established using loperamide intravagination at the beginning of the investigation and treated with the XCQ formula (Figure 2). The results showed that the defecation volume and fecal moisture content of STC rats treated with the XCQ formula were significantly increased compared to those in the model group. The ink carbon propulsion test showed that the intestinal transport function of the experimental group was significantly improved. The XCQ formula increased the number of ICC in the intestinal tissue of rats in the STC model group. These results indicate that the XCQ formula significantly improved defecation in STC rats, which is consistent with the treatment results of clinical patients with STC.

To further explore the mechanism of the XCQ formula, we used 16S rDNA sequencing technology to sequence the DNA extracted from rat feces. The abundance of various bacteria, including Firmicutes, Clostridia, Lachnospiraceae, and *Roseburia* spp., in the feces of the STC group, was significantly reduced compared to that of the normal control group. Simultaneously, fecal metabolomics detection revealed that the content of BAB significantly increased in the STC group and was positively correlated with changes in the abundance of *Roseburia* spp. Therefore, we speculated that the XCQ formula could promote the colonization of *Roseburia* spp. in STC rats and the metabolism of folic acid, benzoic acid, and purine and increase the generation of BAB after metabolism. BAB is an essential nutrient for some flora, promoting their growth and maintaining tissue activity through antioxidant effects (Alicja et al., 2002; Krátký et al., 2019). Therefore, we believe that BAB may promote colonization of *Roseburia* spp. and promote bowel movement (Muhammad et al., 2020).

Moreover, the RNA-seq comparison of rat colon tissue showed significant differences in the expression of IL-21R, PIK3CD, CD40, and other genes, and GO and KEGG function prediction showed that metabolites had a significant influence on the signal transduction, receptor, and ligand connection between cells. Furthermore, we verified changes in c-kit, IL-21R, and their downstream molecules at the protein level. Therefore, we believe that the regulatory effect of the XCQ formula on c-kit expression and intestinal peristalsis may be related to the inflammatory environment, such as IL-21R. IL-21R is mainly expressed in immune cells (T cells, B cells, NK cells, and macrophages) and non-immune cells (dendritic cells, fibroblasts, and intestinal epithelial cells) (Devangi et al., 2004). IL-21 can promote cell proliferation and differentiation in tumors and inflammatory diseases, such as intestinal chronic diseases, type I diabetes, and rheumatoid arthritis. Its α/γ chain can bind to IL-21 to form a complex that activates JAK1 and JAK3 and their downstream STAT3, STAT1, STAT5A, and STAT5B and the PI3K/Akt signaling pathway to regulate cell growth (Tania et al., 2003; Jennifer et al., 2009; Palani and Mahaboobkhan, 2019). Studies have shown that IL-21R activation can inhibit Th1 and activate Th2, Th17, and Treg responses, which can help alleviate DSS-induced acute colitis, and IL-21R may be a new target for the treatment of inflammatory bowel disease and other enterodynamic disorders (Ian et al., 2007; Wang et al., 2016). However, in the treatment of constipation, few studies have been conducted on the relationship between ICC and the inflammatory environment. Therefore, a new target for the effective treatment of STC may be identified.

Based on previous studies, BAB was found to be a differential metabolite of the XCQ formula, which could effectively improve bowel movement in patients with constipation. BAB is an amino acid ester formed by the formal condensation of the carboxyl group of 4-aminobenzoic acid (PABA) and the hydroxyl group of butane-1-alcohol. PABA is an essential nutrient for some flora. It can promote the growth of intestinal flora and maintain tissue activity through antioxidation. Mass spectrometry showed that PABA is an effective component of the XCQ formula. Butane-1-alcohol can be produced in small amounts by human and mouse intestinal microorganisms, such as polyphycococcus, lactic acid bacteria, *Escherichia coli*, and *Clostridium*. Animal experiments showed that XCQ could promote the colonization of intestinal *Clostridium*, especially *Trichospirillum*, a butyric acid-producing bacterium, or increase the content of the intestinal metabolite butane-1-alcohol. However, PABA was not identified in the three groups of fecal metabolites during the metabolomic detection of feces, but the content of BAB, the product of PABA, was significantly different. We speculated that PABA in XCQ could act on the intestinal tissue and promote the proliferation of intestinal ICC after synthesizing a certain amount of BAB in the intestine and intestinal flora.

To clarify the mechanism of action of BAB, a metabolite of the XCQ formula, we extracted the primary colon ICC from rats, tested the expression of IL-21R in ICC by cellular immunofluorescence, structured the STC model with loperamide, and treated cells with BAB. Finally, the underlying mechanism was verified in human primary colon ICC to validate our conclusions across different species. The results showed that loperamide can induce caspase-dependent apoptosis of ICC in the rat and human colon, whereas aminobutyl at a sufficient concentration can inhibit further apoptosis of ICC and play a protective role. WB analysis and flow cytometry showed that BAB alleviated loperamide-induced ICC apoptosis by promoting STAT3 and ERK phosphorylation. Moreover, when IL-21R was knocked down from the ICC surface, the antiapoptotic effect of BAB on ICC was abolished. Indeed, the apoptosis of ICC induced by loperamide could not be inhibited by IL-21R silencing, and STAT3 and ERK downstream molecules of IL-21R could not be activated. When STATTIC was used, the inhibitor of STAT3 and PD98059, an inhibitor of the ERK pathway, was used to treat cells and inhibit the activation of STAT3 and ERK, the protective effect of BAB on ICC was also abolished, and the reduction of p-STAT3 expression was observed, while PD98059 inhibited ERK phosphorylation. However, STATTIC treatment did not inhibit p-ERK expression. ERK is upstream of STAT3 in the protective mechanism of aminobutyl against ICC. Therefore, we believe that BAB can activate the downstream ERK/STAT3 signaling pathway, promote ICC proliferation, and reverse loperamide-induced ICC cell apoptosis by interfering with IL-21R activity on the surface of ICC. Thus, the XCQ formula promotes colonization of *Roseburia* spp. and increases the content of BAB after metabolism, which protects ICC activity, thus improving STC symptoms.

In this study, we used the guidance of TCM theory to formulate a slow-transit constipation rat model to study the mechanism of the XCQ formula in STC treatment. We found that intestinal flora and metabolic changes play important roles in the pathogenesis and treatment of STC. Our results provide a sound theoretical basis for the rational and effective use of TCM in the clinical setting.

However, this study had some limitations. As mentioned above, the composition of TCM is complex. Thus, the causal relationship between the XCQ formula and its metabolites and *Roseburia* spp., the effect of other metabolites on intestinal motility, and the role of IL-21 release in the alteration of the intestinal inflammatory environment in STC patients warrant further experimental investigation. Furthermore, the detailed source of BAB and its relationship with *Roseburia* spp. have not been clarified and need to be further verified. For instance, STC sterile rat model should be constructed and treated with XCQ formula, *Roseburia* spp., and BAB, respectively. The colonic transport function, histological changes, and CD117 expression of rats of rat colon should be observed to determine the therapeutic effect of STC, and the intestinal flora and major metabolites should be detected to further investigate the association between XCQ formula, *Roseburia* spp., and BAB. Moreover, it is necessary to further develop the beneficial effect of dominant flora and metabolites of the XCQ formula on STC in clinical use, determine the differences in the intestinal flora and immune status of patients with STC treated with XCQ formula, and determine the impact of the formula on the internal environment in patients with STC.

## Conclusion

This study focused on the pathological changes in ICC in a rat STC model and the treatment mechanism of the XCQ formula, a TCM prescription. The XCQ formula restored colonic dynamic indices, colonic lesions, and the number of ICC in STC rats. Combined with the results of previous studies, we speculate that the XCQ formula can promote the colonization of *Roseburia* spp., increase the production of the metabolite BAB, protect the activity of ICC, and thus improve the symptoms of STC.

## Data Availability

The datasets presented in this study can be found in online repositories. The names of the repository/repositories and accession number(s) can be found below: https://www.ncbi.nlm.nih.gov/, PRJNA798229.

## Abbreviations

BAB, butyl aminobenzene; GO, gene ontology; ICC, interstitial cells of Cajal; IL-21R, Interleukin-21 Receptor; OUT, operational taxon units; PBS, phosphate-buffered saline; STC, slow transit constipation; SCF, stem cell factor; TCM, traditional Chinese medicine; XCQ, Xiao Chengqi.
