# Corrigendum: Effects of Xiao Chengqi formula on slow transit constipation by assessing gut microbiota and metabolomics analysis *in vitro* and *in vivo*

**DOI:** 10.3389/fphar.2023.1256600

**Published:** 2023-10-31

**Authors:** Qian Zhou, Di Zhang, Heng Zhang, Xingyang Wan, Bang Hu, Qi Zou, Dan Su, Hui Peng, Dandan Huang, Donglin Ren

**Affiliations:** ^1^ Department of Coloproctology, The Sixth Affiliated Hospital, Sun Yat-sen University, Guangzhou, China; ^2^ Guangdong Provincial Key Laboratory of Colorectal and Pelvic Floor Diseases, The Sixth Affiliated Hospital, Sun Yat-sen University, Guangzhou, China; ^3^ Guangdong Institute of Gastroenterology, The Sixth Affiliated Hospital, Sun Yat-sen University, Guangzhou, China

**Keywords:** Xiao Chengqi formula, traditional Chinese medicine, slow transit constipation, butyl aminobenzene, interstitial cells of cajal, interleukin-21 receptor

In the published article, there was an error in Figure 10 as published. In Figure 10C, the PD+L+B apoptosis figure was misplaced with ST+L+B figure while drawing. The corrected Figure 10 and its caption appear below.

**FIGURE 10:**
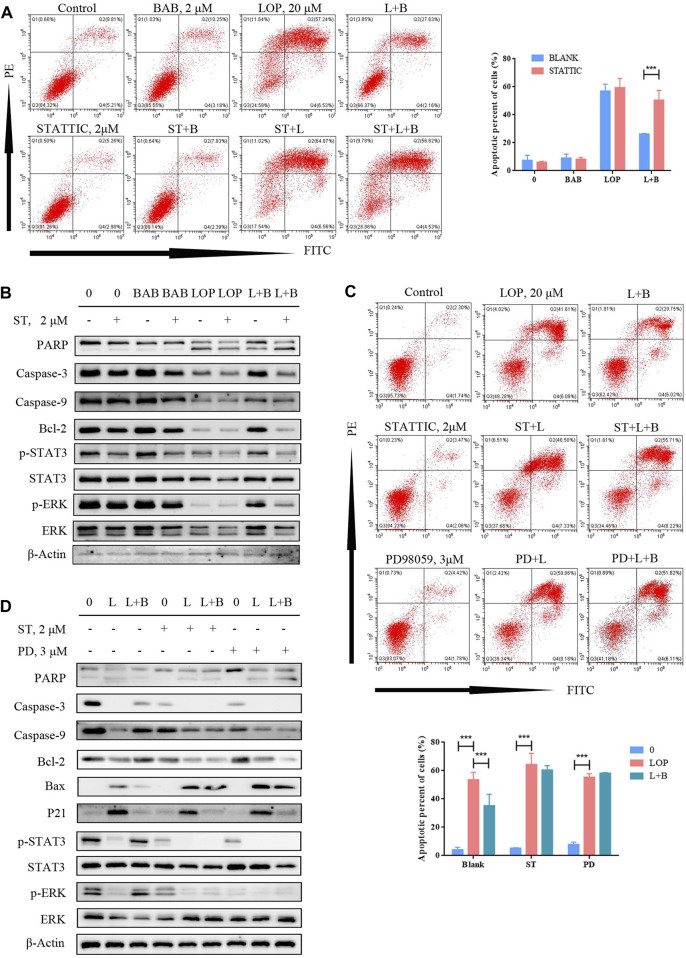
BAB can reverse lop induced interstitial cells of Cajal apoptosis. **(A)** Flow cytometry was used to detect the apoptosis after STAT3 was inhibited and activated; **(B)** Western blot was used to detect the apoptosis after STAT3 was inhibited and activated; **(C)** flow cytometry was used to detect the apoptosis after STAT3 or ERK was inhibited; statistical analyses were shown below; **(D)** PD98059 and STATTIC were used to inhibit the activate of STAT3 and ERK. The expression of proteins associated with caspase pathway, cell proliferation and the activation of STAT3 and ERK were detected by WB.

Additionally, the “TSN” and “AGS and HGC-27 cells” words showed in the **Materials and Methods** section were incorrect.

A correction has been made to **Materials and Methods**, *Cell Viability*, Paragraph 1. This sentence previously stated:

“The effect of TSN on cell viability was tested using the Cell Counting Kit-8 (CCK-8) assay (Nanjing KeyGen Biotech Co., Ltd., Nanjing, Jiangsu, China). Briefly, cells were seeded into 96- well plates (3 × 103 cells/well) and treated with TSN (0.5–2 µM).”

The corrected sentence appears below:

“The effect of XCQ formula on cell viability was tested using the Cell Counting Kit-8 (CCK-8) assay (Nanjing KeyGen Biotech Co., Ltd., Nanjing, Jiangsu, China). Briefly, cells were seeded into 96-well plates (5 × 10^3^ cells/well) and treated with Butamben or Loperamide.”

A correction has been made to **Materials and Methods**, *Cell Cycle Analysis*, Paragraph 1. This sentence previously stated:

“AGS and HGC-27 cells were incubated with TSN for the indicated times, after which the cells were collected, washed, and fixed with 66% cold ethanol at 4°C overnight.”

The corrected sentence appears below:

“ICC cells were incubated with Butamben or Loperamide for the indicated times, after which the cells were collected, washed, and fixed with 66% cold ethanol at 4°C overnight.”

A correction has been made to **Materials and Methods**, *Cell Apoptosis Analysis*, Paragraph 1. This sentence previously stated:

“Cells were treated with or without TSN for the indicated times, after which the cells were collected, washed, and suspended in 500 µL of binding buffer, and mixed with 5 µL of annexin V-FITC and 5 µL of propidium iodide.”

The corrected sentence appears below:

“Cells were treated with or without specific drugs for the indicated times, after which the cells were collected, washed, and suspended in 500 µL of binding buffer, and mixed with 5 µL of annexin V-FITC and 5 µL of propidium iodide.”

Lastly, the figure insertion was mislabeled in Results, RNA-Sequencing section.

A correction has been made to the **Results** section, subsection *RNA-Sequencing*, Paragraph 1. This sentence previously stated:

“Furthermore, we validated the changes in CD117, IL-21R, and their downstream molecules at the protein level (**Figures 5B–E**).”

The corrected sentence appears below:

“Furthermore, we validated the changes in CD117, IL-21R, and their downstream molecules at the protein level (**Figures 5B–F**).”

The authors apologize for these errors and state that this does not change the scientific conclusions of the article in any way. The original article has been updated.

