# Supplementary figures and images for: Effects of Xiao Chengqi Formula on Slow Transit Constipation by Assessing Gut Microbiota and Metabolomics Analysis in vitro and in vivo (part 1 of 3)
Source: Front Pharmacol. 2022 Jun 8;13:864598. doi: 10.3389/fphar.2022.864598 (PMC9237644; doi:10.3389/fphar.2022.864598)

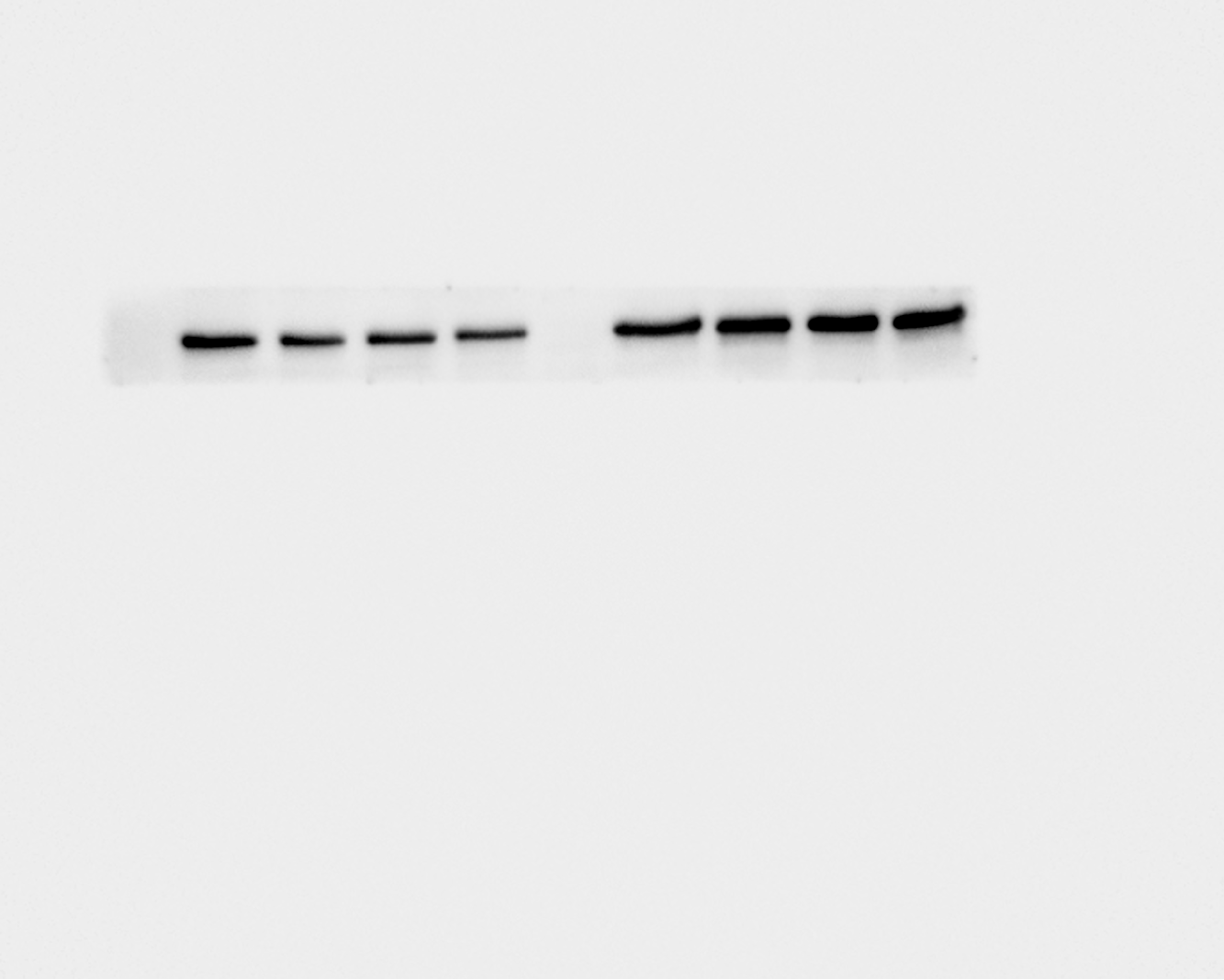

Supplement: Supplementary file 1 [file DataSheet3.ZIP › homo-LOP+b/actin_4(Chemiluminescence).tif]

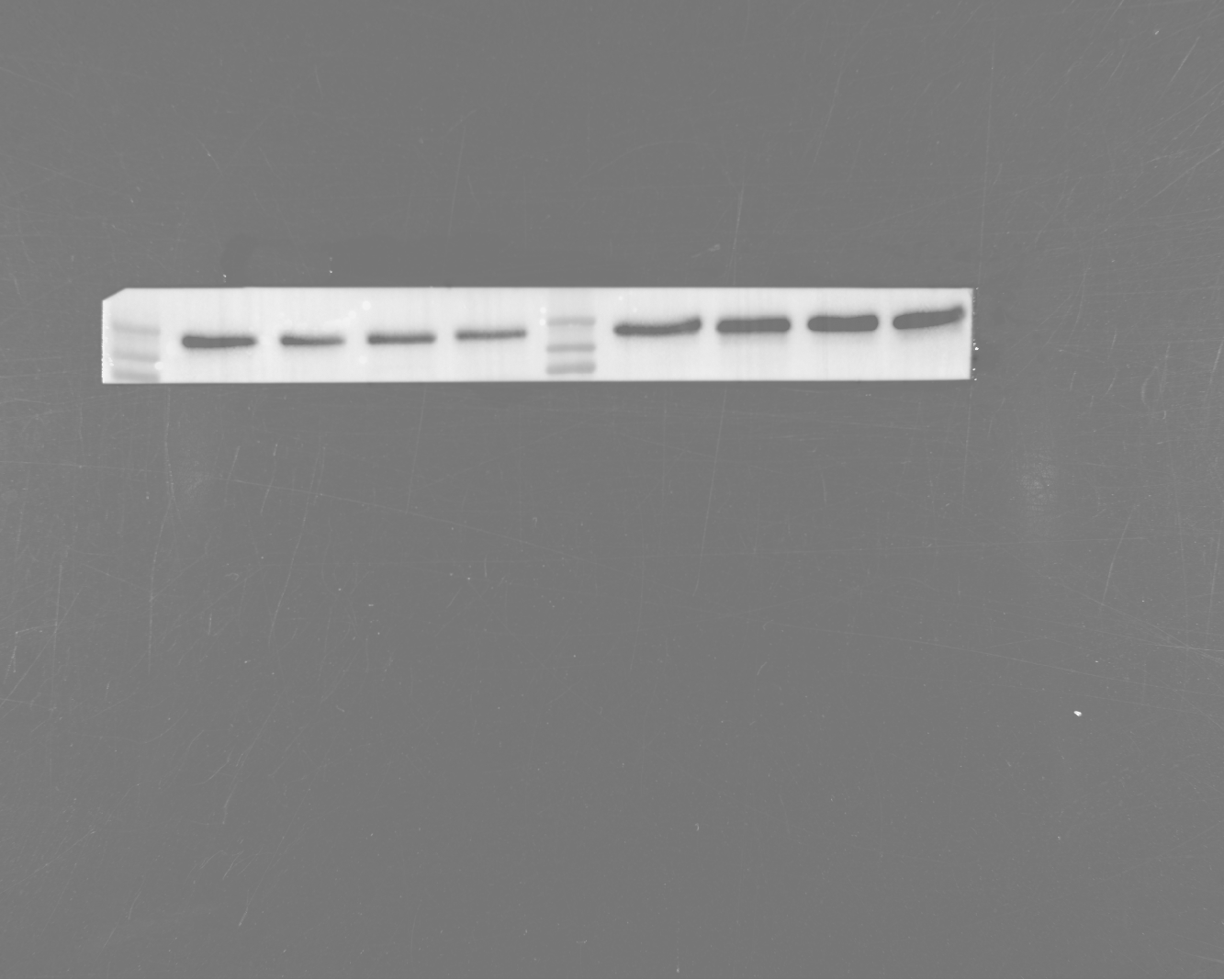

Supplement: Supplementary file 1 [file DataSheet3.ZIP › homo-LOP+b/actin_4(Composite).tif]

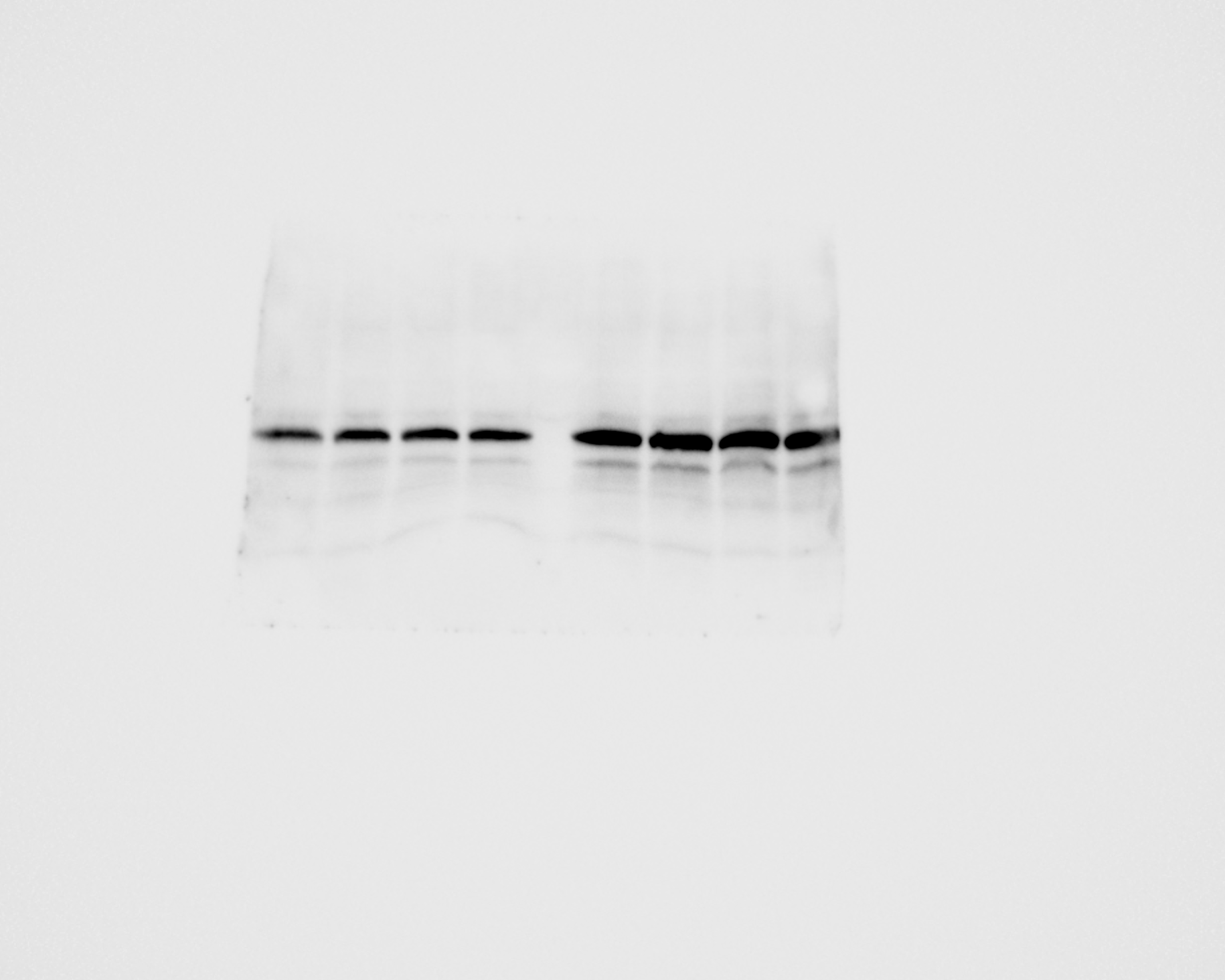

Supplement: Supplementary file 1 [file DataSheet3.ZIP › homo-LOP+b/anorectal 2020-07-20 15h40m12s(Chemiluminescence).tif]

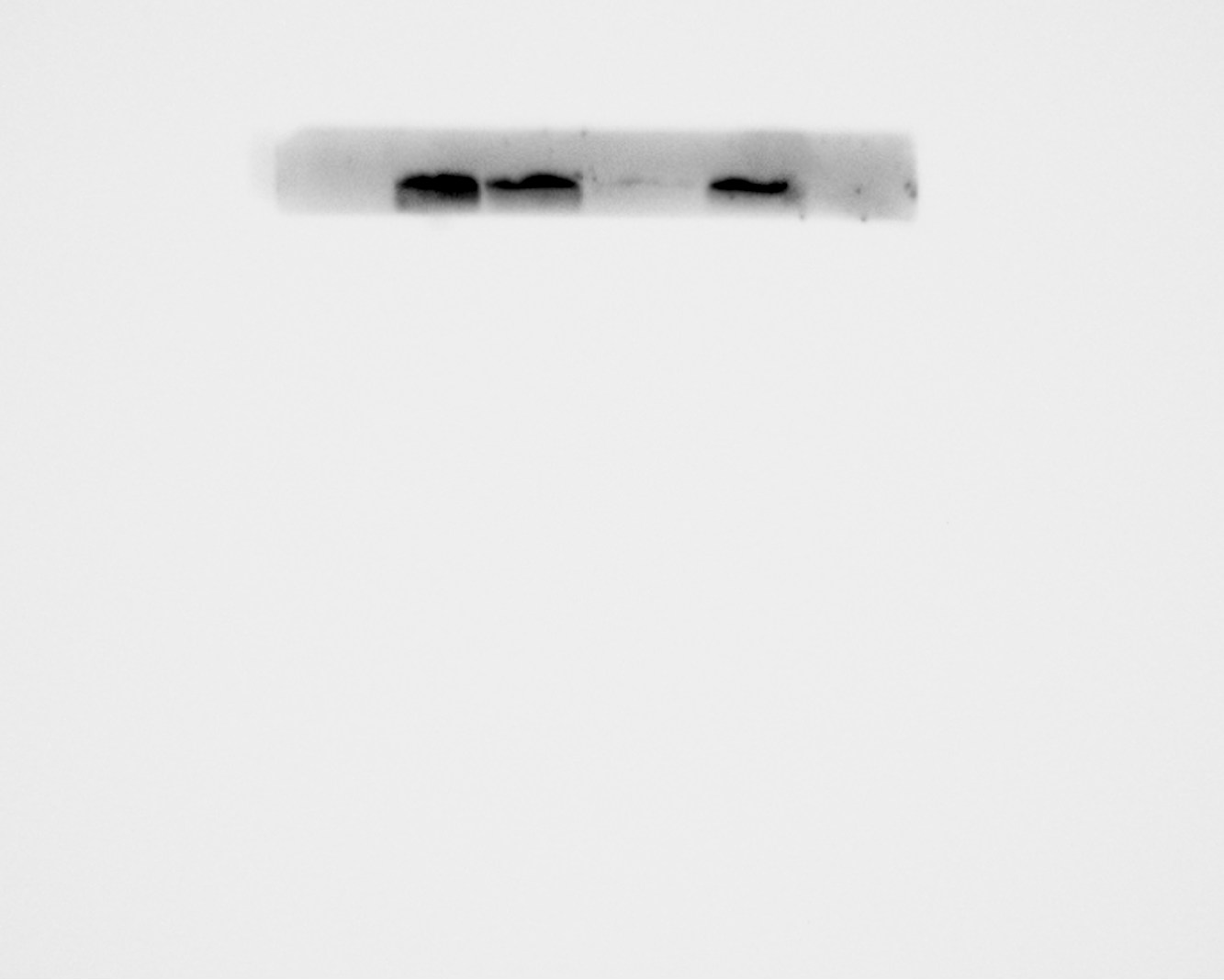

Supplement: Supplementary file 1 [file DataSheet3.ZIP › homo-LOP+b/anorectal 2020-10-07 15h34m51s(Chemiluminescence).tif]

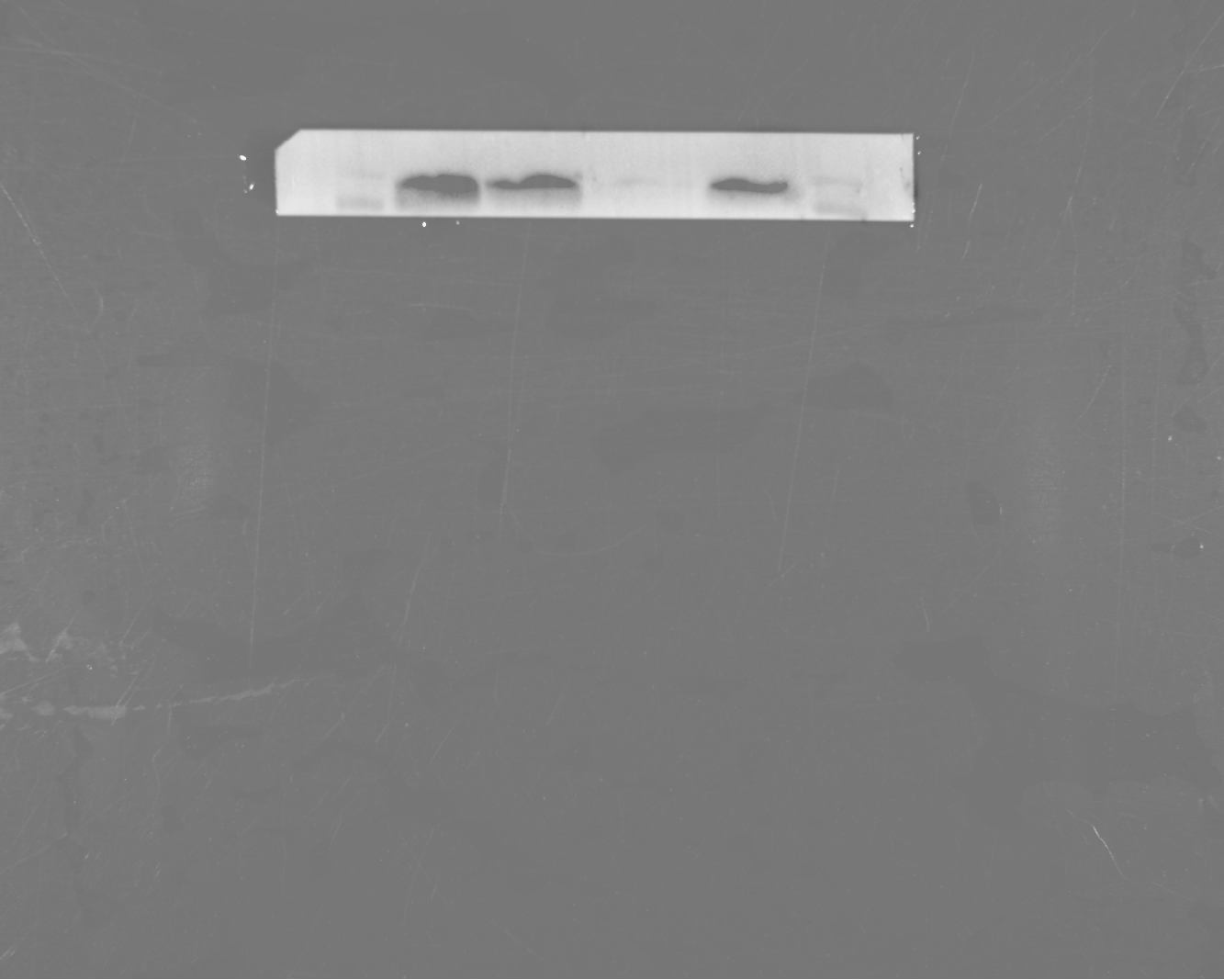

Supplement: Supplementary file 1 [file DataSheet3.ZIP › homo-LOP+b/anorectal 2020-10-07 15h34m51s(Composite).tif]

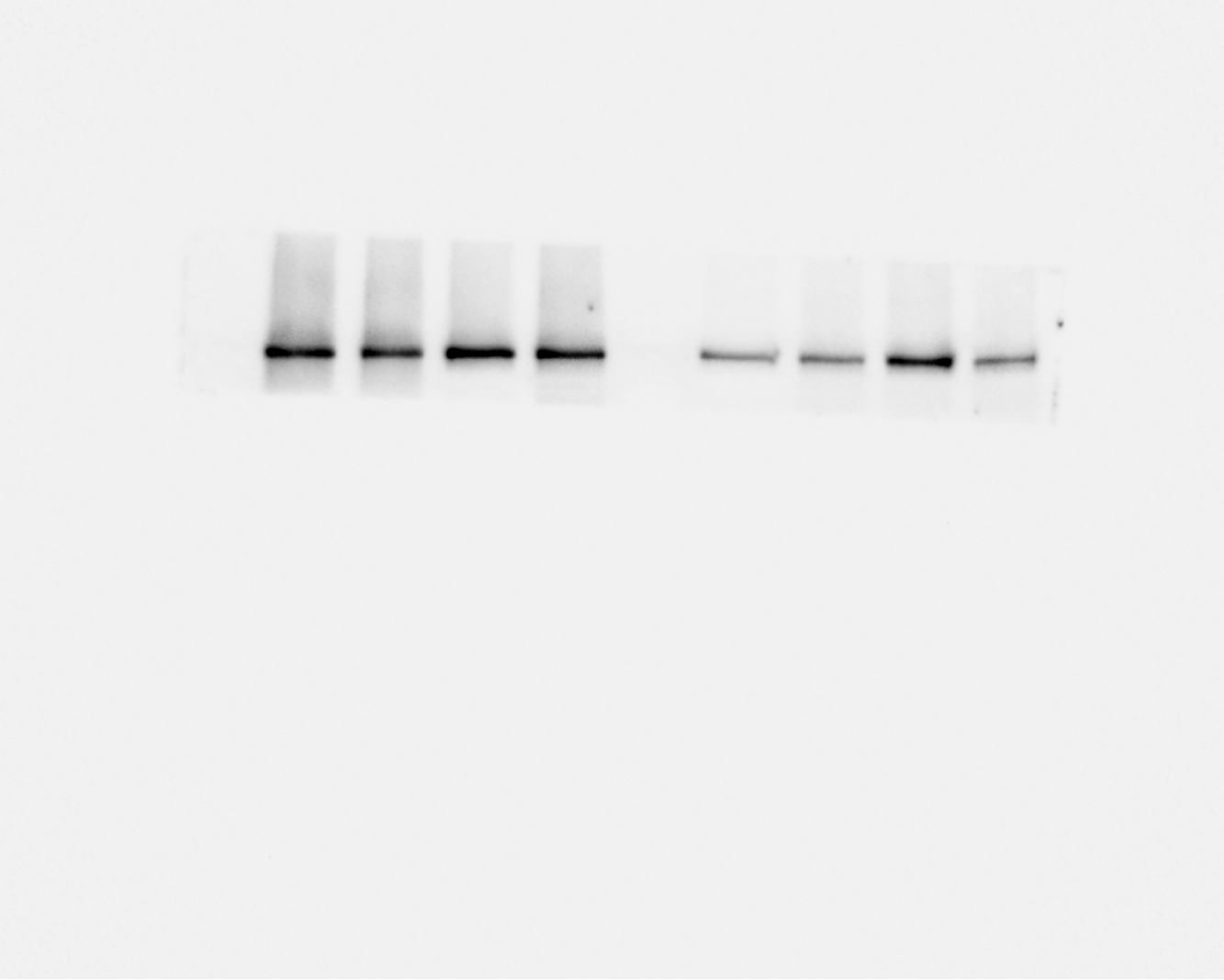

Supplement: Supplementary file 1 [file DataSheet3.ZIP › homo-LOP+b/anorectal 2020-10-13 13h57m20s(Chemiluminescence).tif]

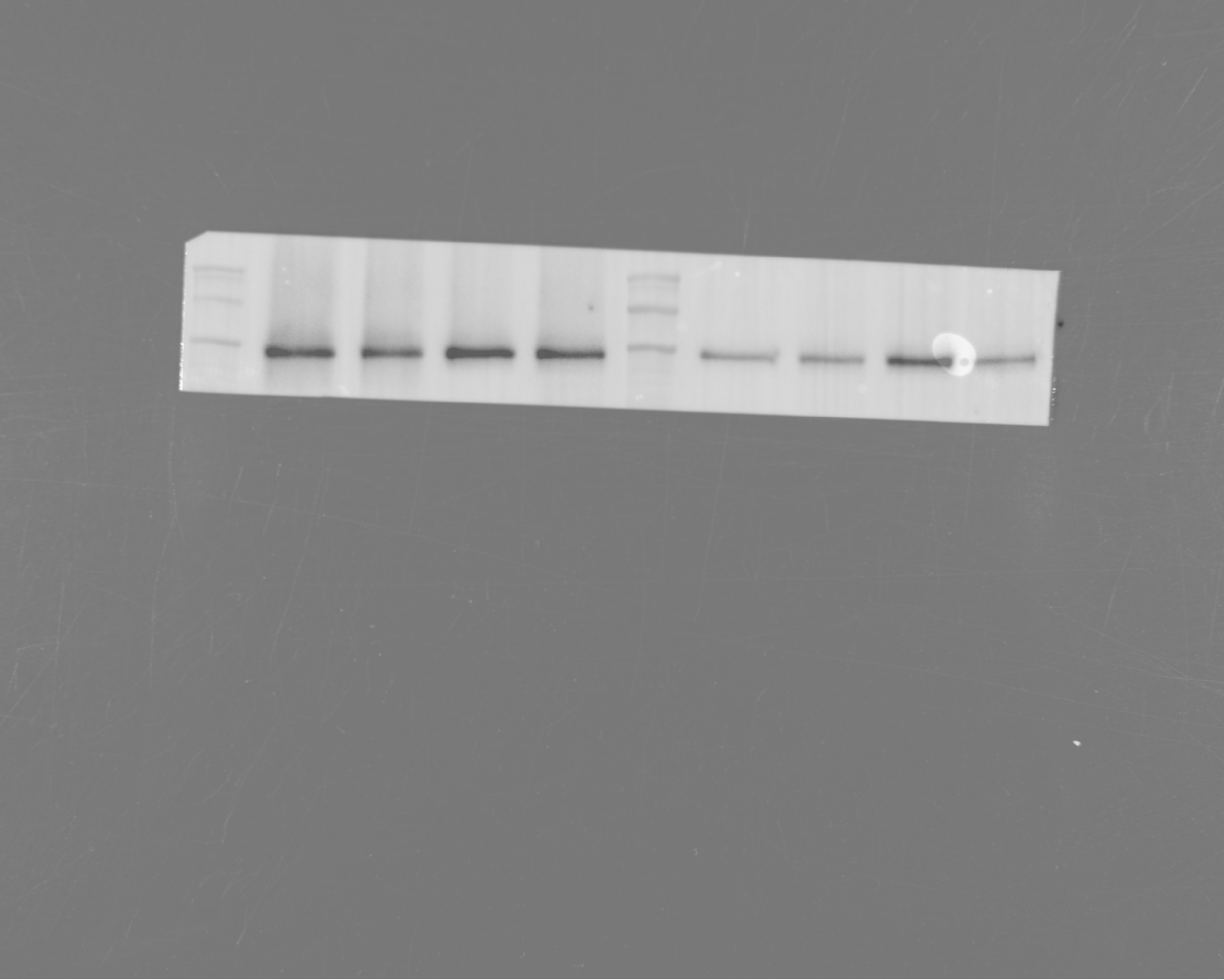

Supplement: Supplementary file 1 [file DataSheet3.ZIP › homo-LOP+b/anorectal 2020-10-13 13h57m20s(Composite).tif]

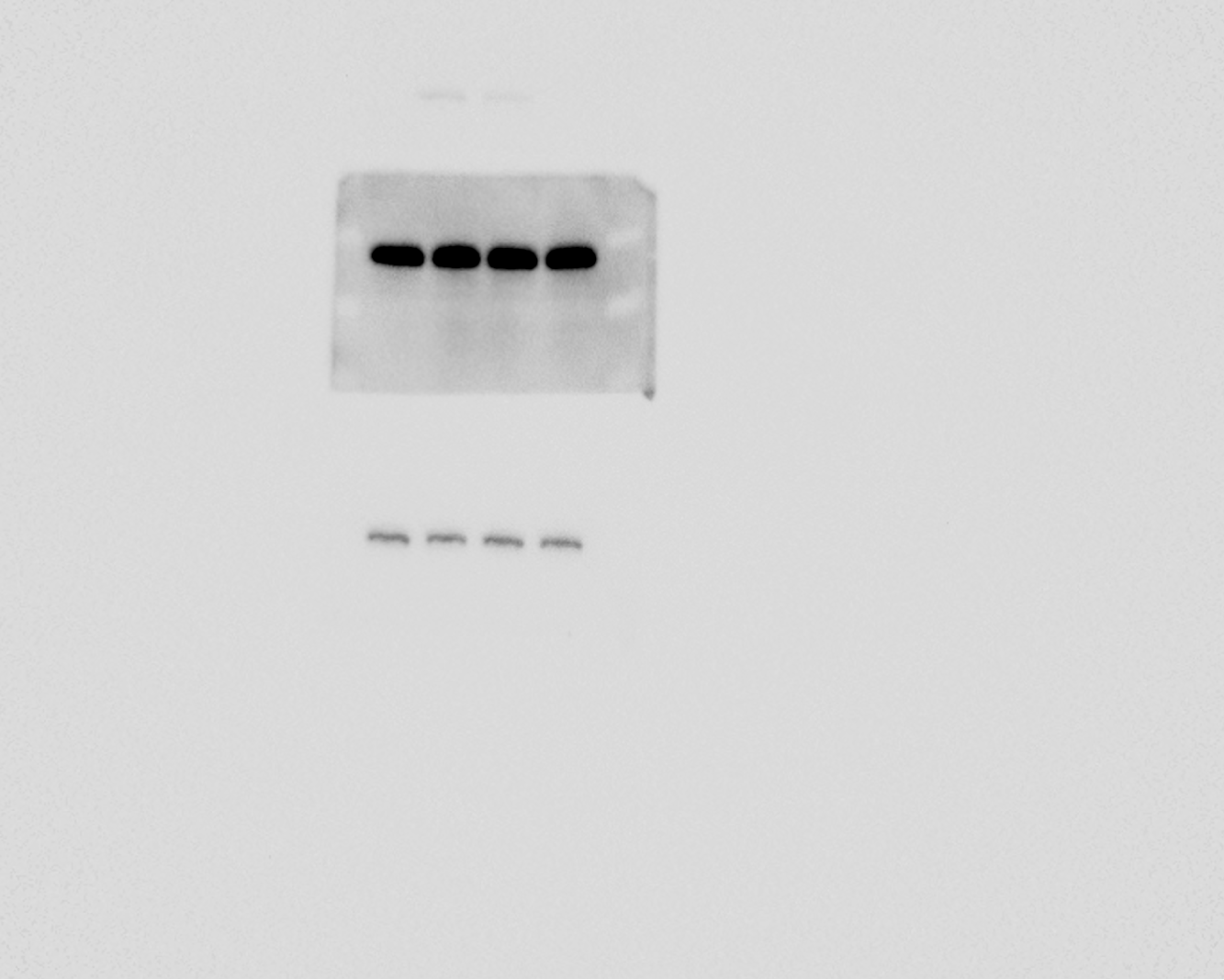

Supplement: Supplementary file 1 [file DataSheet3.ZIP › homo-LOP+b/anorectal 2021-01-29 11h01m17s(Chemiluminescence).tif]

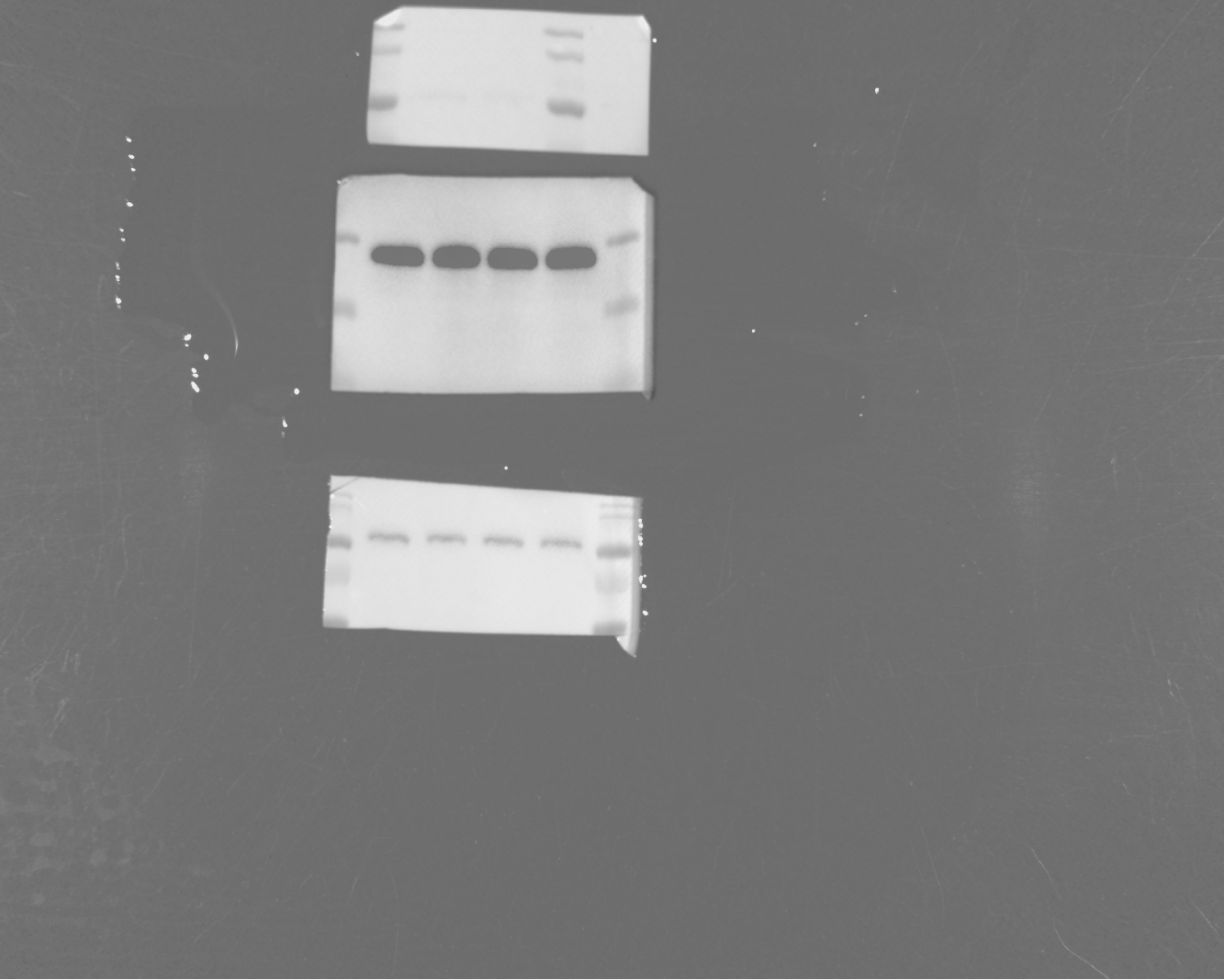

Supplement: Supplementary file 1 [file DataSheet3.ZIP › homo-LOP+b/anorectal 2021-01-29 11h01m17s(Composite).tif]

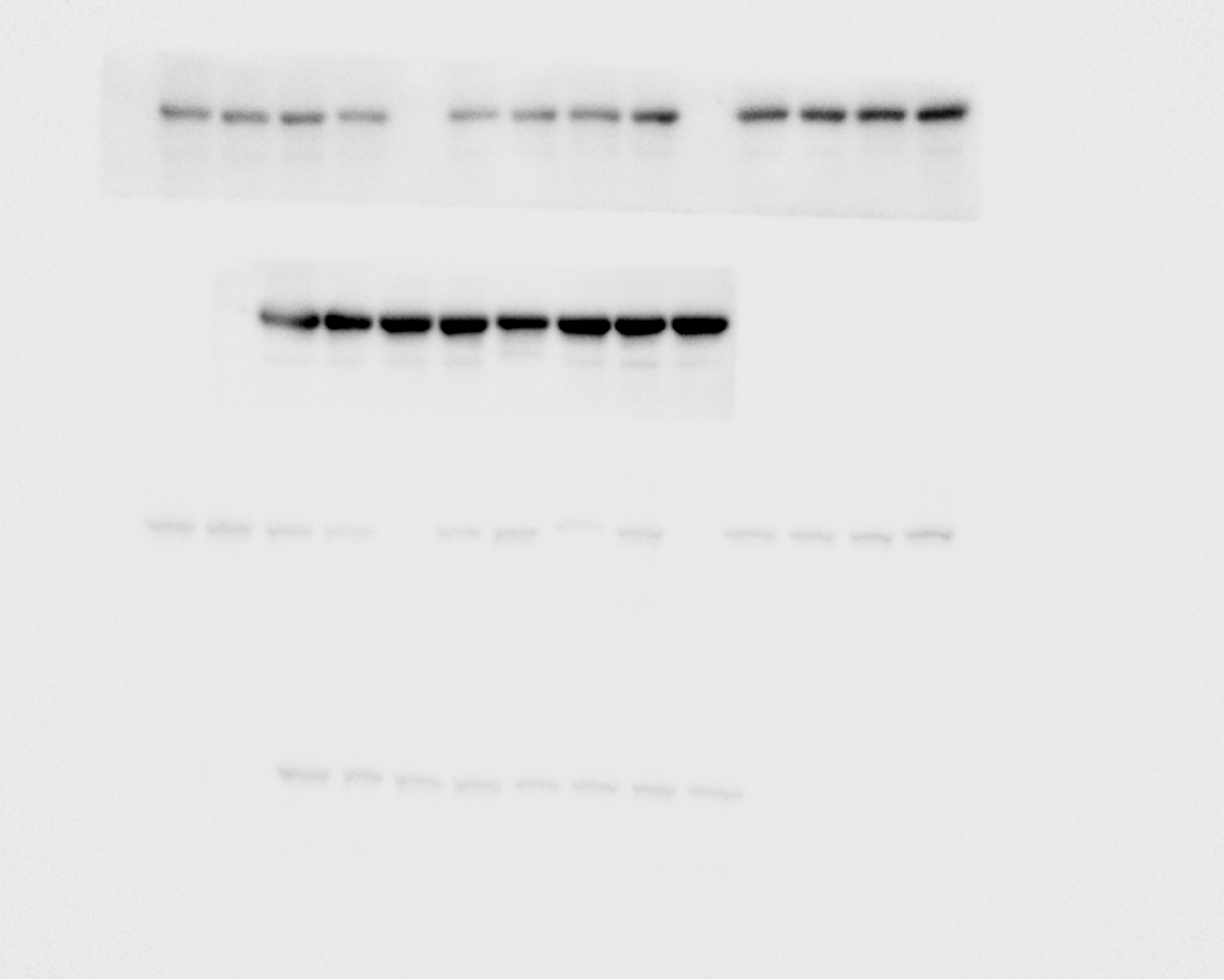

Supplement: Supplementary file 1 [file DataSheet3.ZIP › homo-LOP+b/anorectal 2021-01-29 11h06m44s(Chemiluminescence).tif]

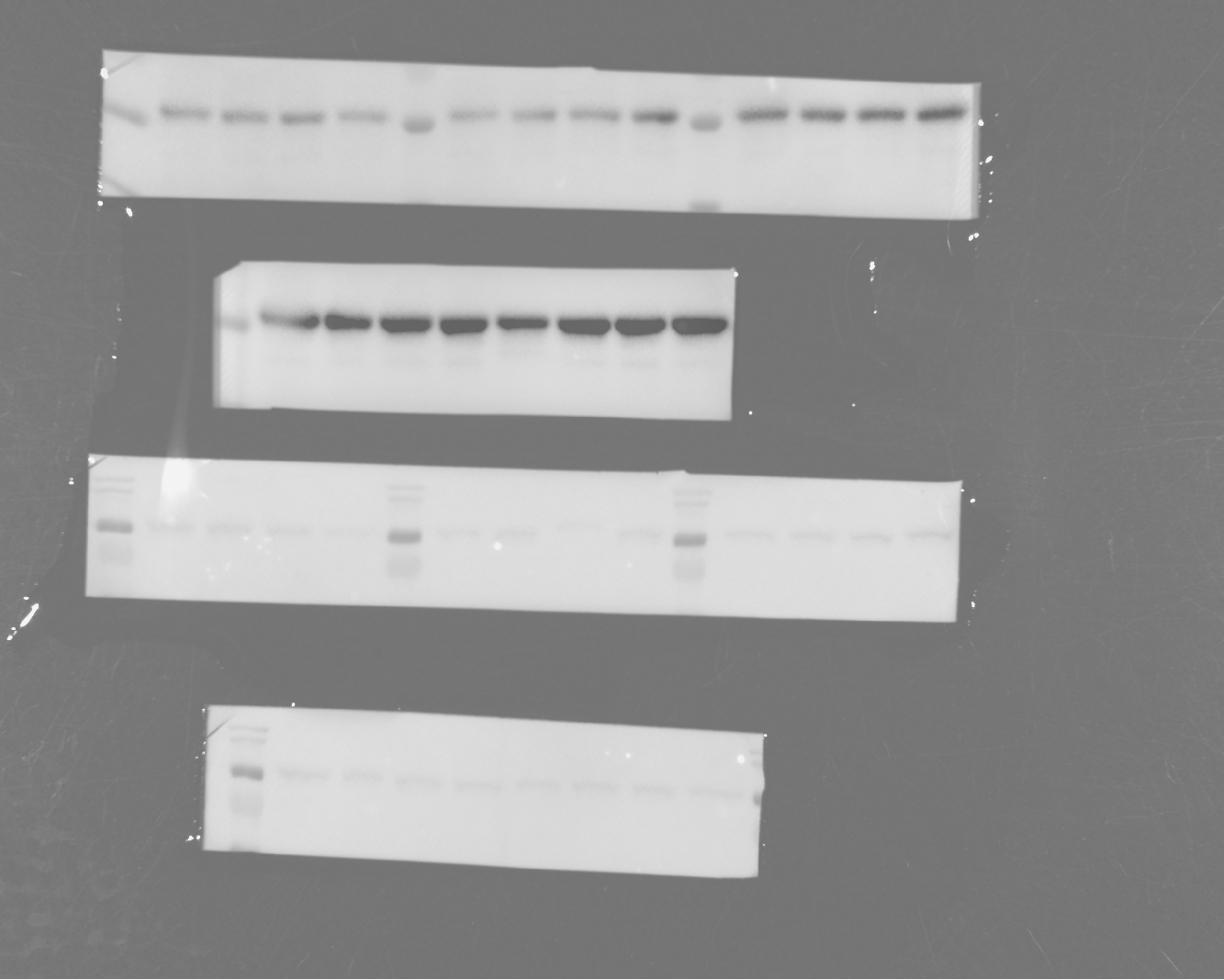

Supplement: Supplementary file 1 [file DataSheet3.ZIP › homo-LOP+b/anorectal 2021-01-29 11h06m44s(Composite).tif]

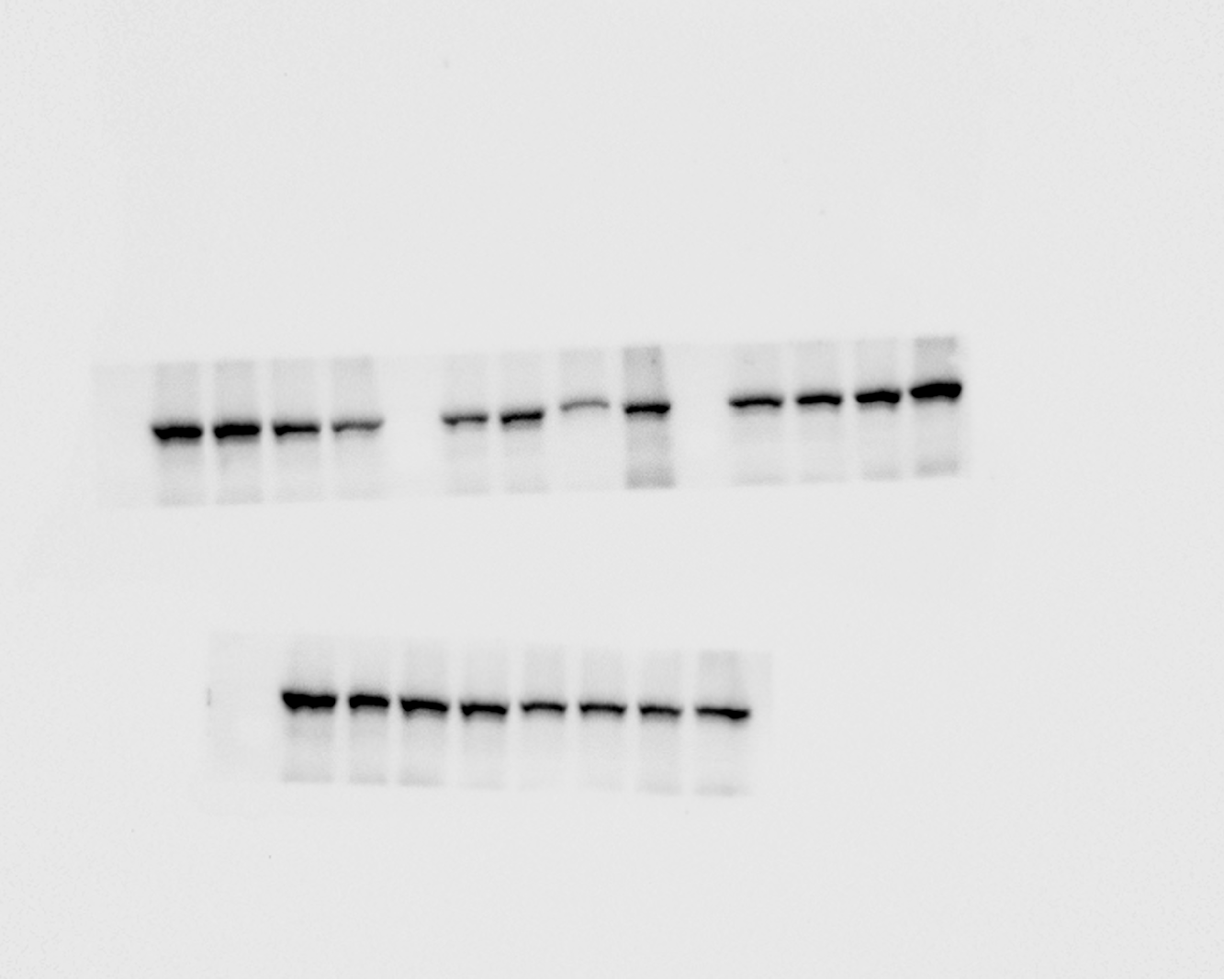

Supplement: Supplementary file 1 [file DataSheet3.ZIP › homo-LOP+b/anorectal 2021-01-29 11h08m36s(Chemiluminescence).tif]

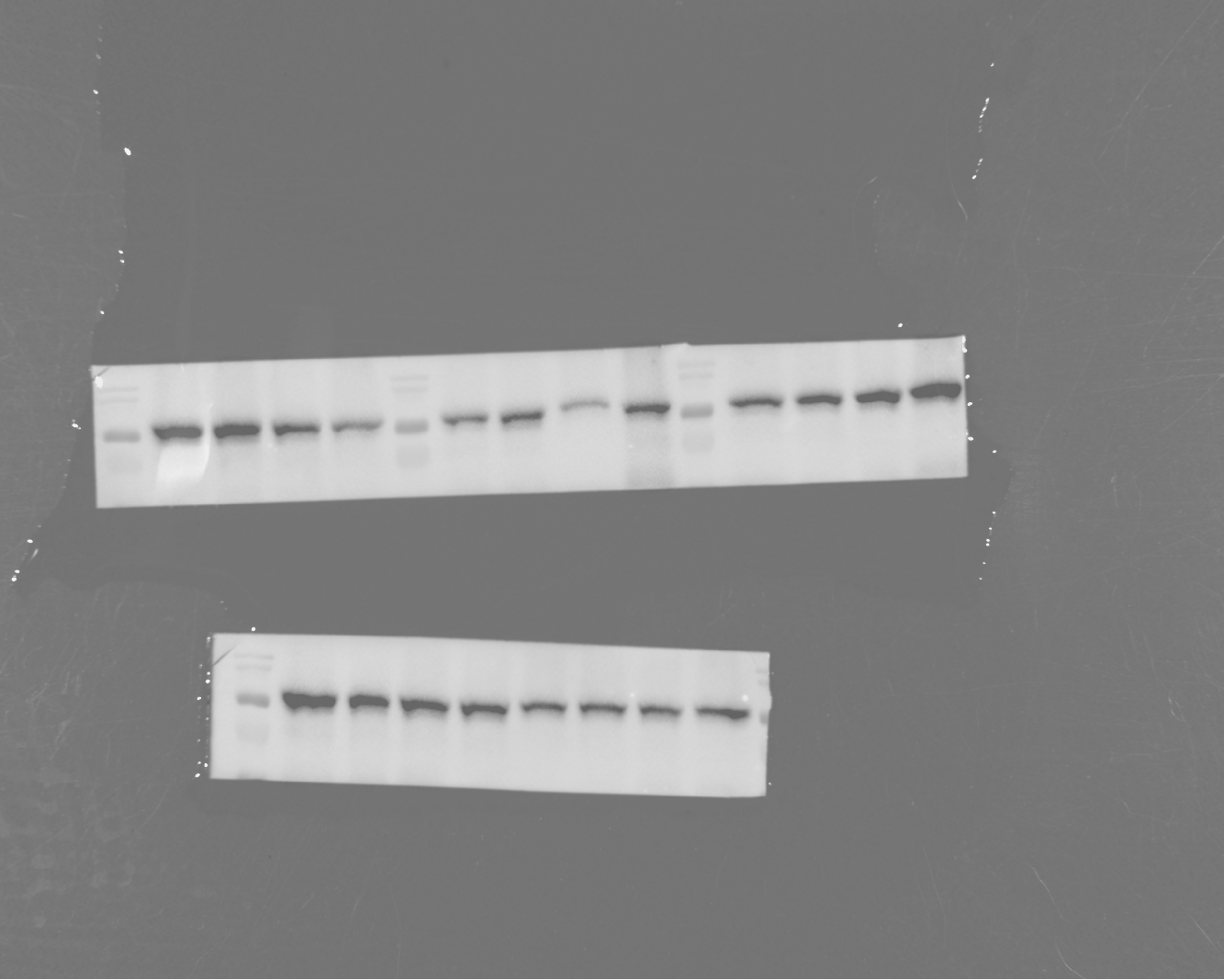

Supplement: Supplementary file 1 [file DataSheet3.ZIP › homo-LOP+b/anorectal 2021-01-29 11h08m36s(Composite).tif]

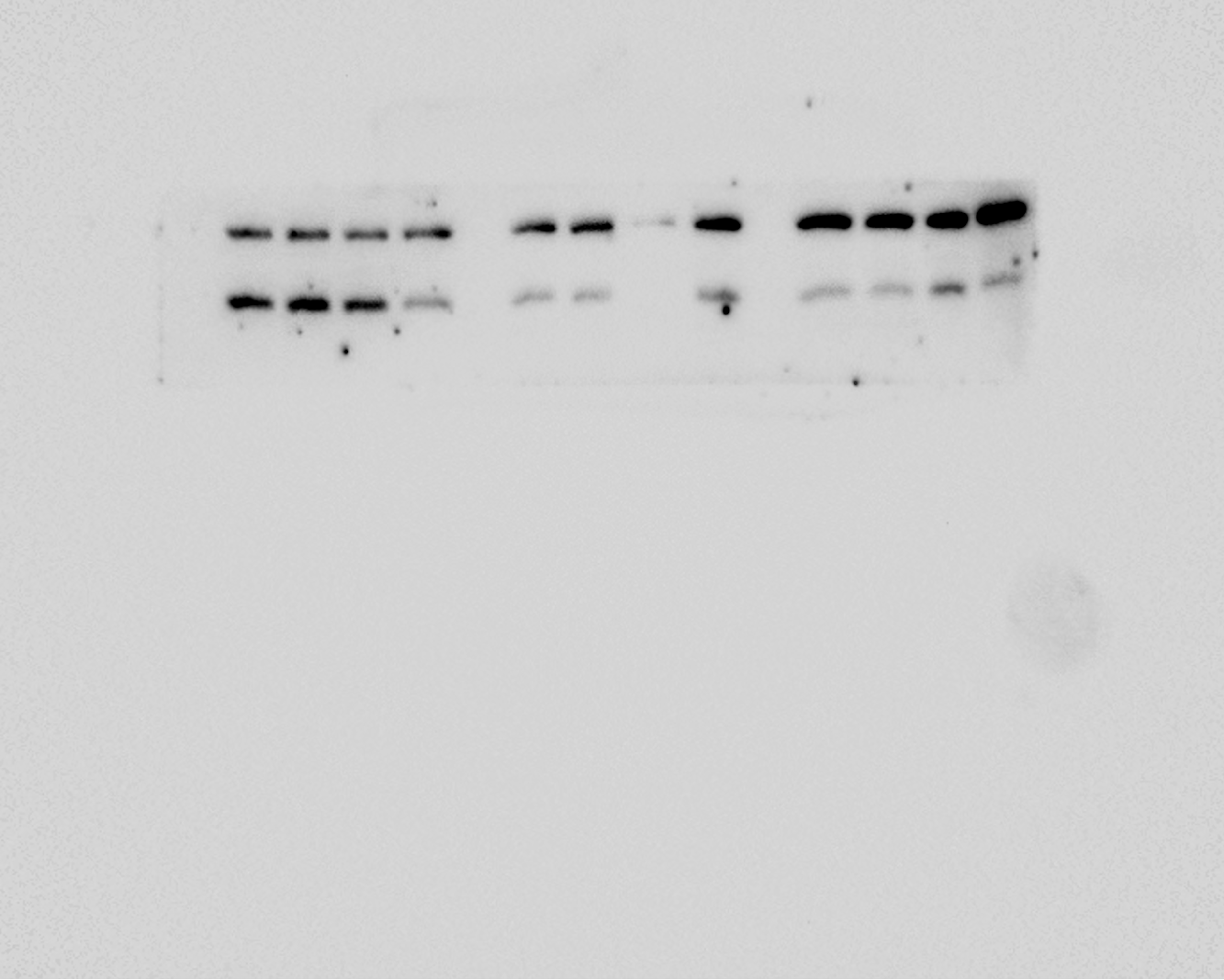

Supplement: Supplementary file 1 [file DataSheet3.ZIP › homo-LOP+b/anorectal 2021-01-30 20h51m54s(Chemiluminescence).tif]

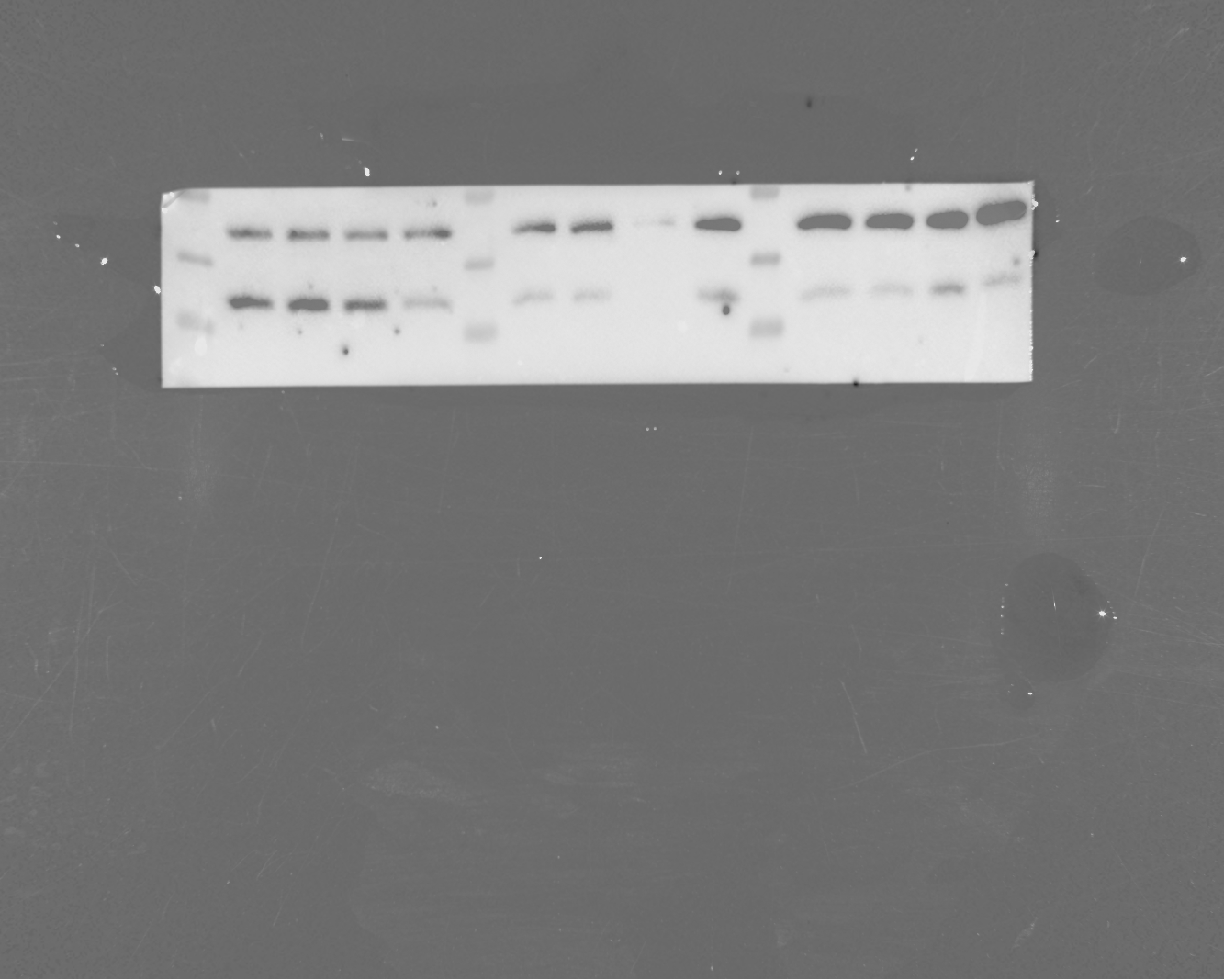

Supplement: Supplementary file 1 [file DataSheet3.ZIP › homo-LOP+b/anorectal 2021-01-30 20h51m54s(Composite).tif]

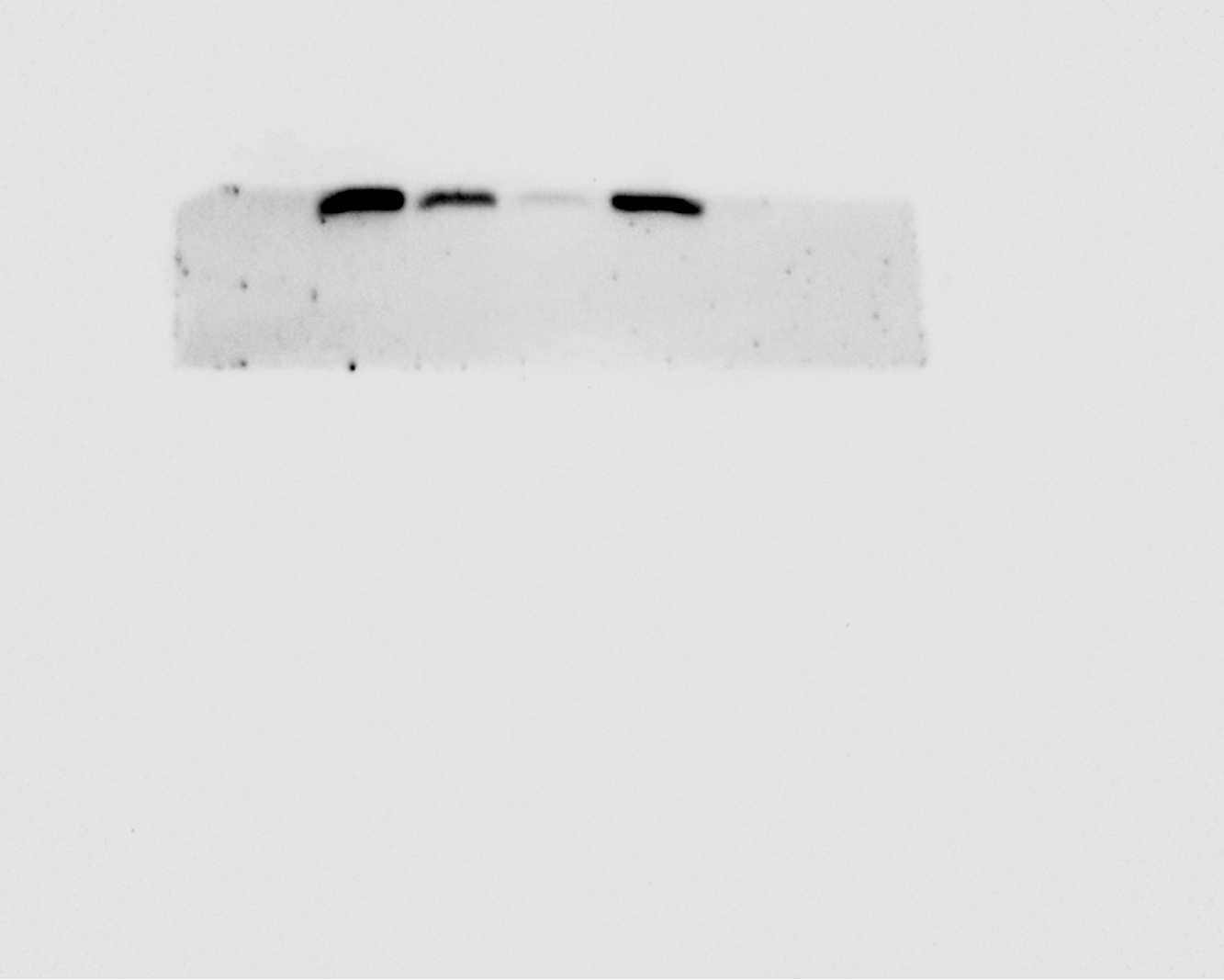

Supplement: Supplementary file 1 [file DataSheet3.ZIP › homo-LOP+b/cas3_3(Chemiluminescence).tif]

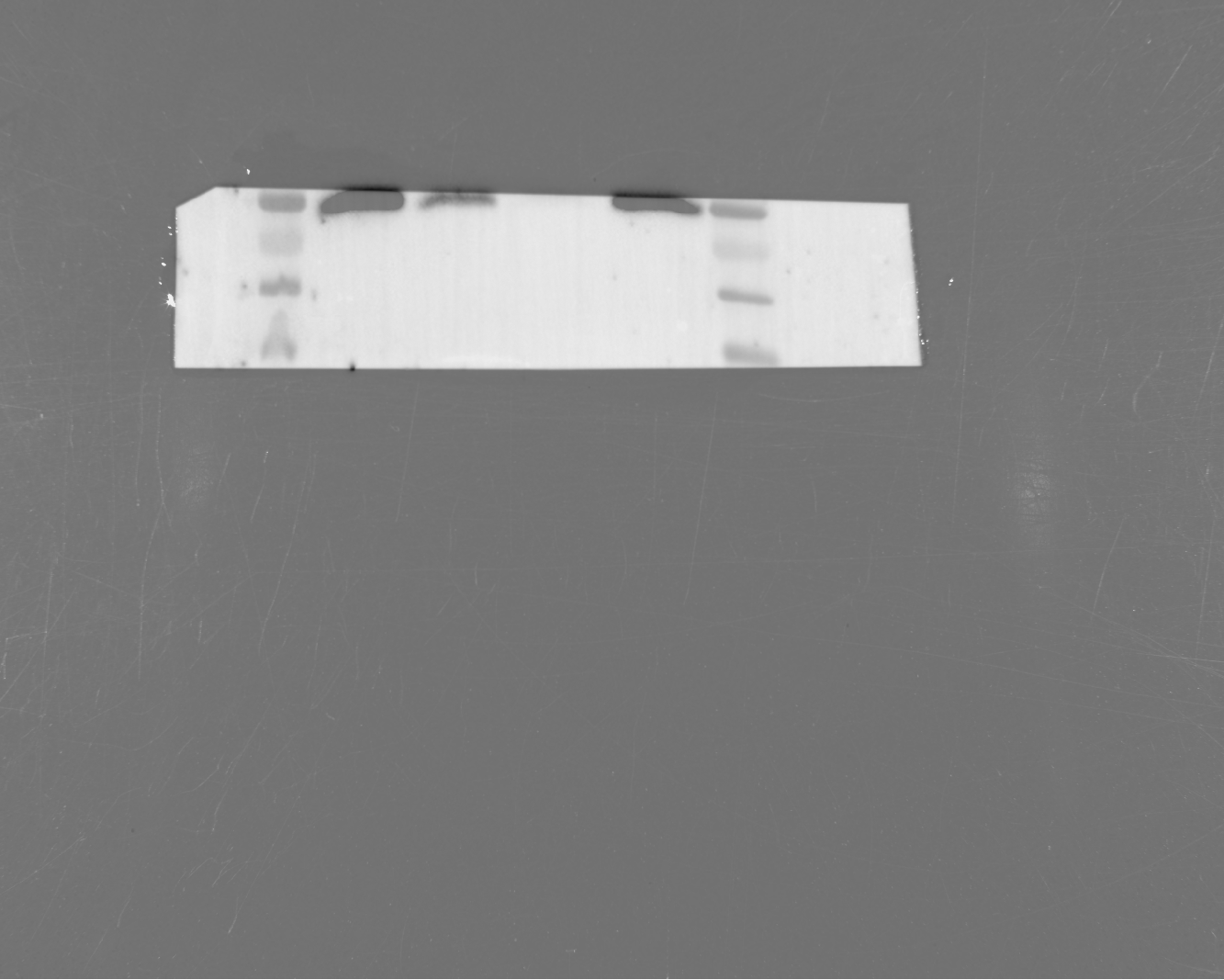

Supplement: Supplementary file 1 [file DataSheet3.ZIP › homo-LOP+b/cas3_3(Composite).tif]

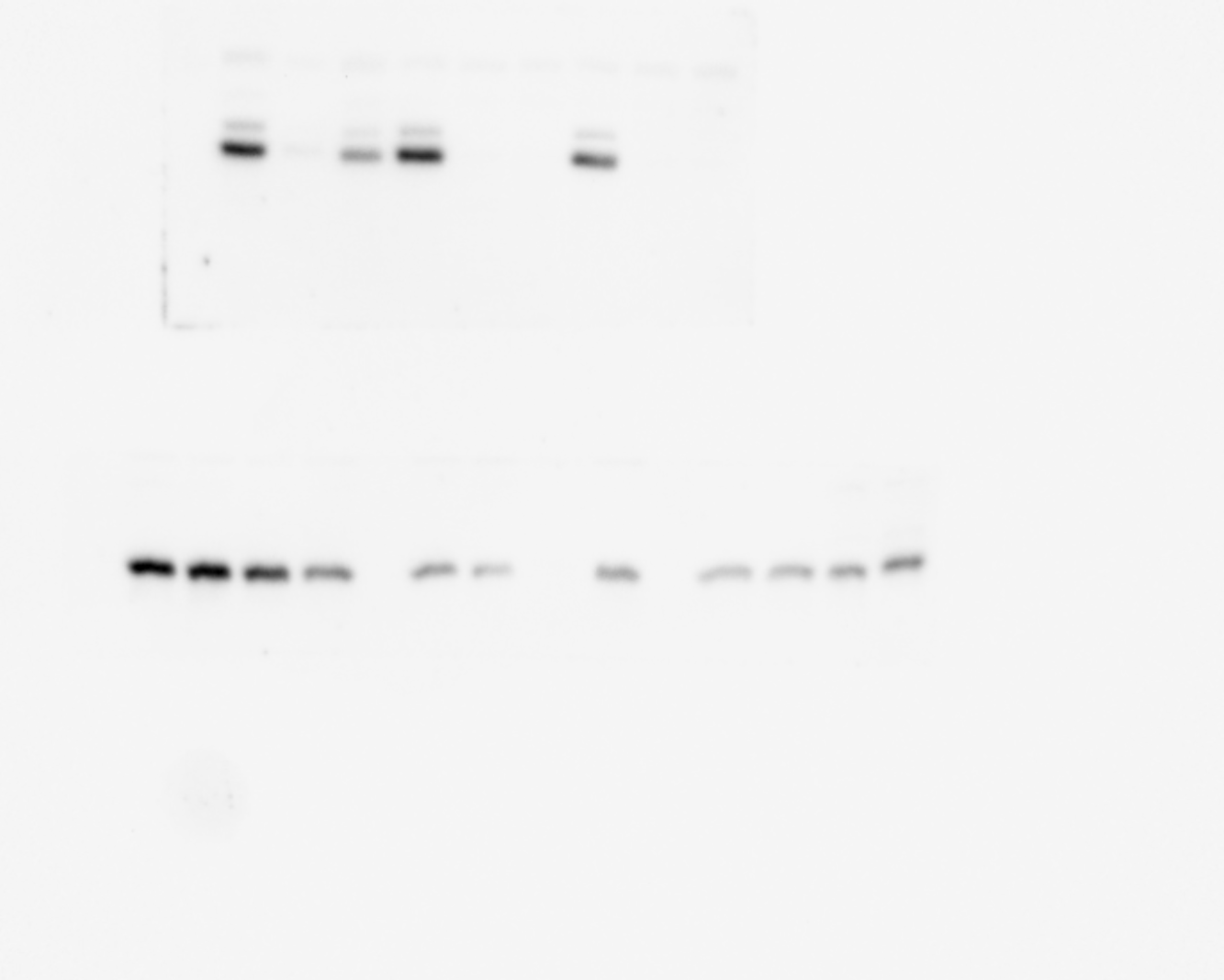

Supplement: Supplementary file 1 [file DataSheet3.ZIP › homo-LOP+b/cas3-lop-st_1(Chemiluminescence).tif]

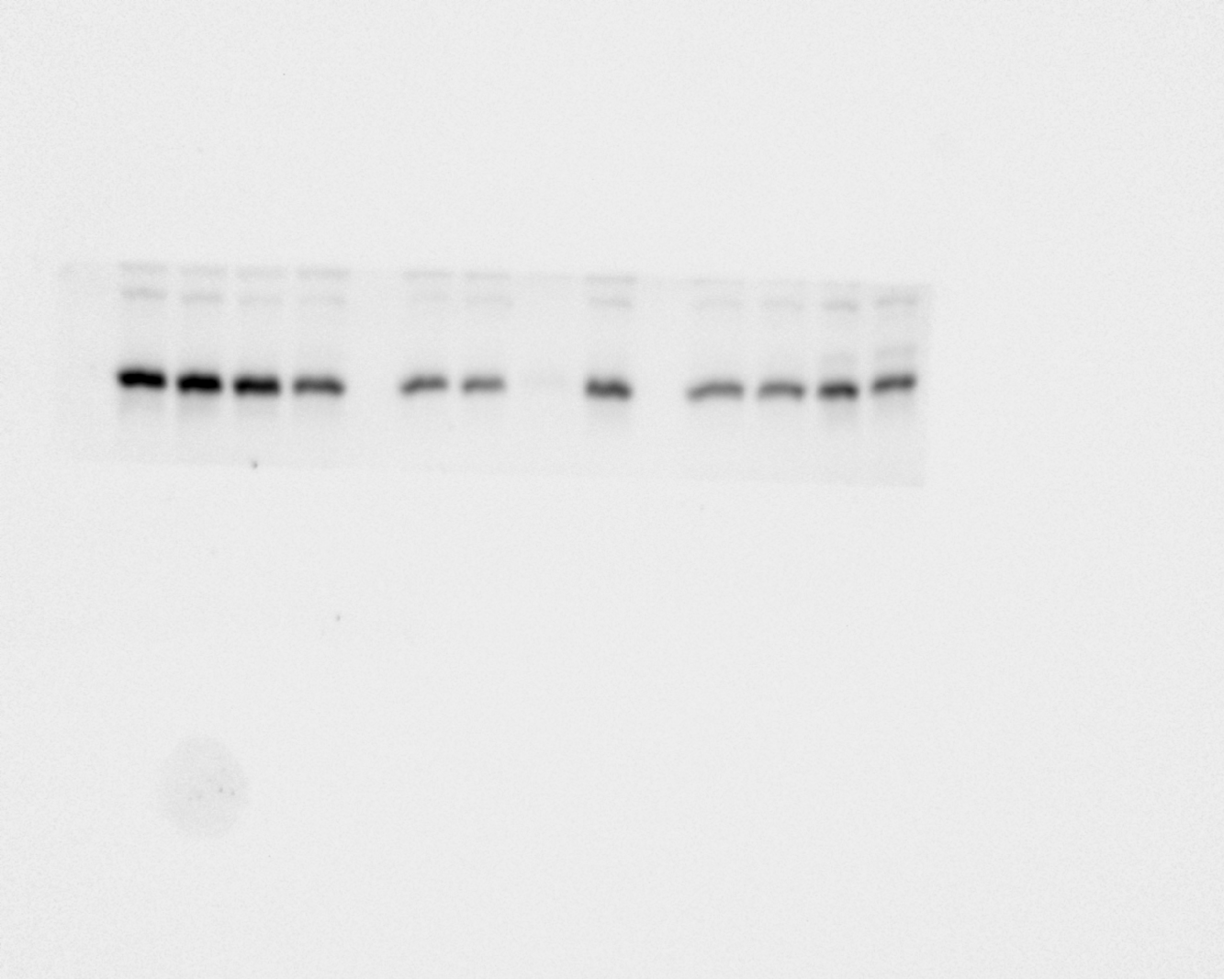

Supplement: Supplementary file 1 [file DataSheet3.ZIP › homo-LOP+b/cas3-lop-st_5(Chemiluminescence).tif]

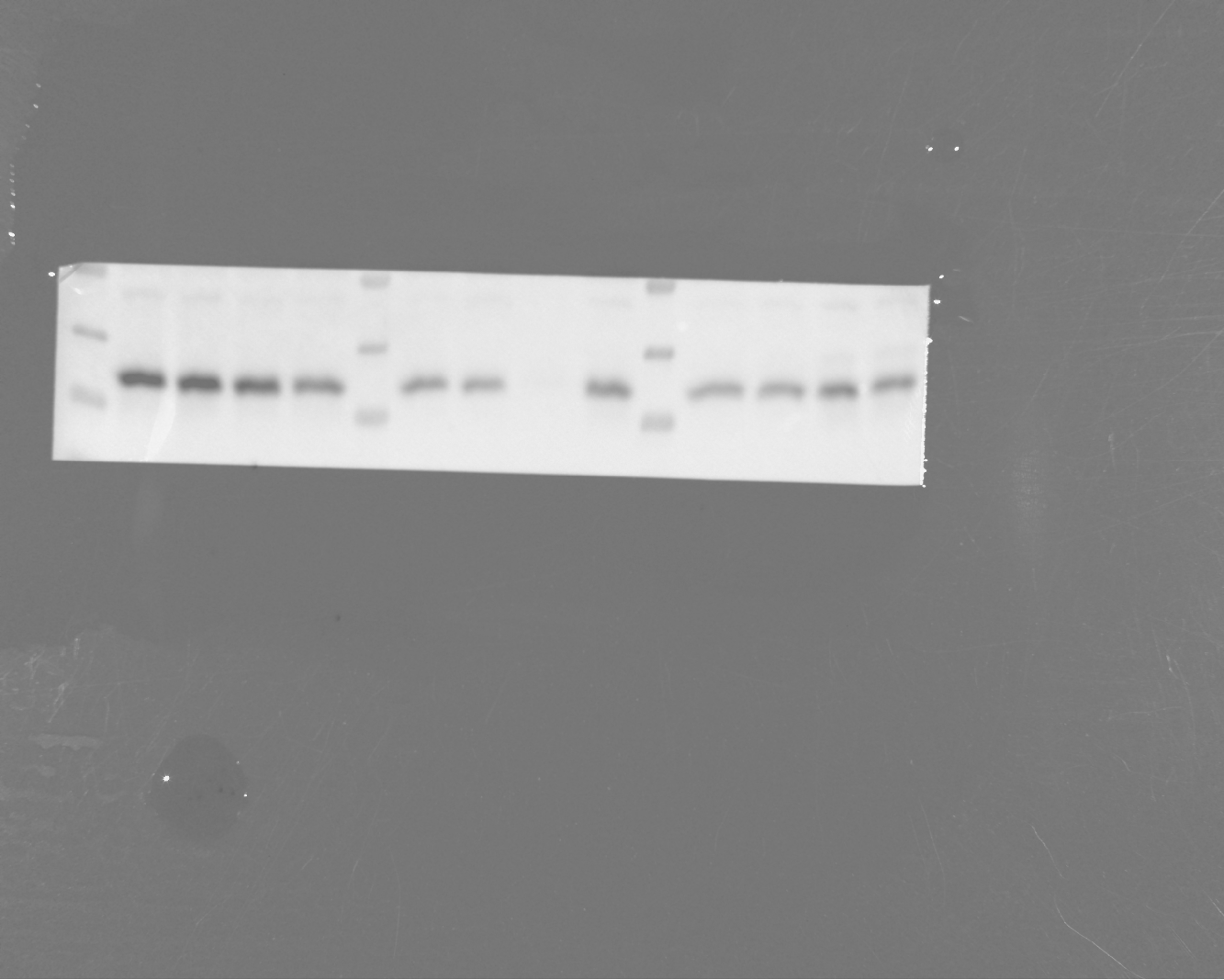

Supplement: Supplementary file 1 [file DataSheet3.ZIP › homo-LOP+b/cas3-lop-st_5(Composite).tif]

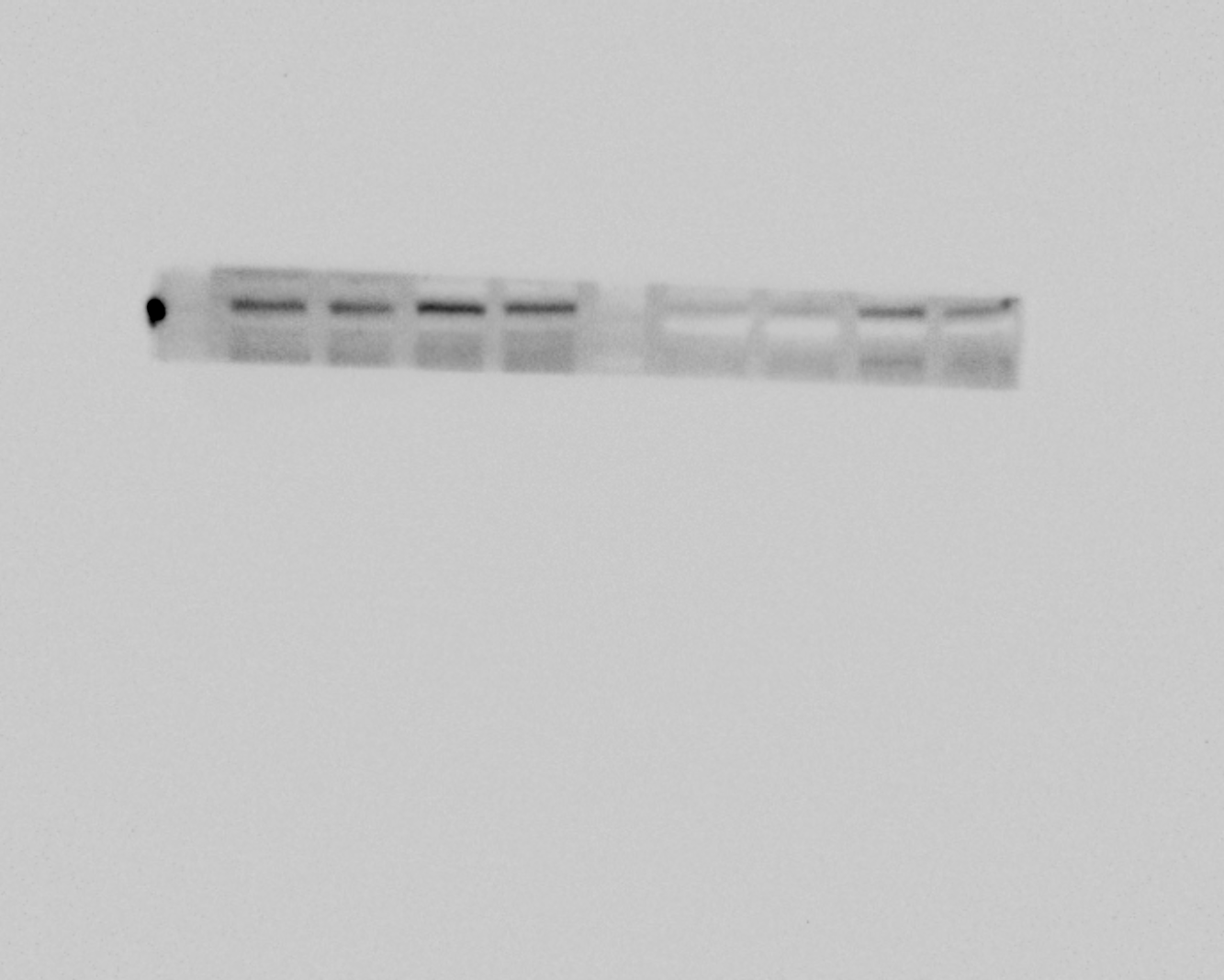

Supplement: Supplementary file 1 [file DataSheet3.ZIP › homo-LOP+b/cas9_2(Chemiluminescence).tif]

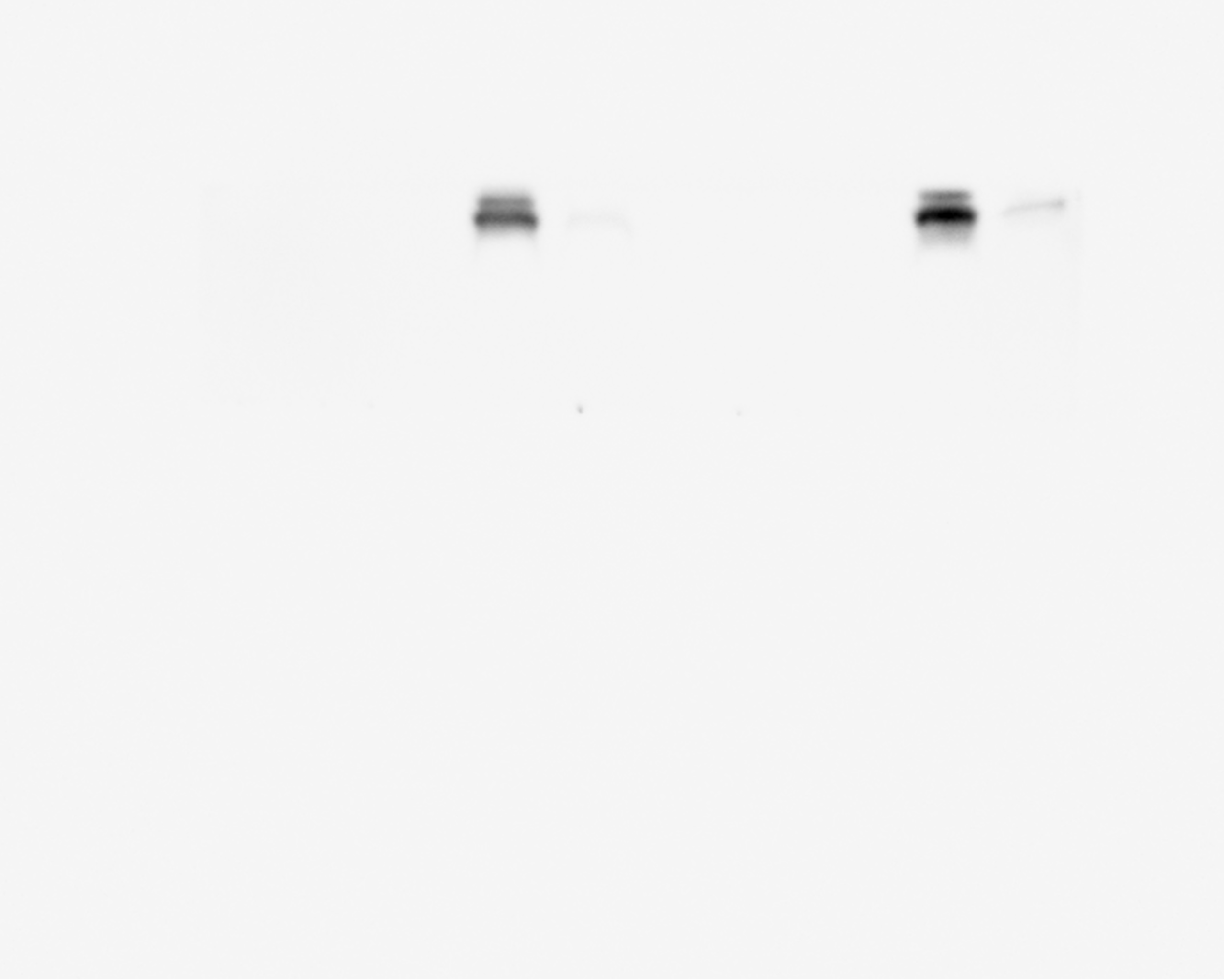

Supplement: Supplementary file 1 [file DataSheet3.ZIP › homo-LOP+b/cc3 (1).tif]

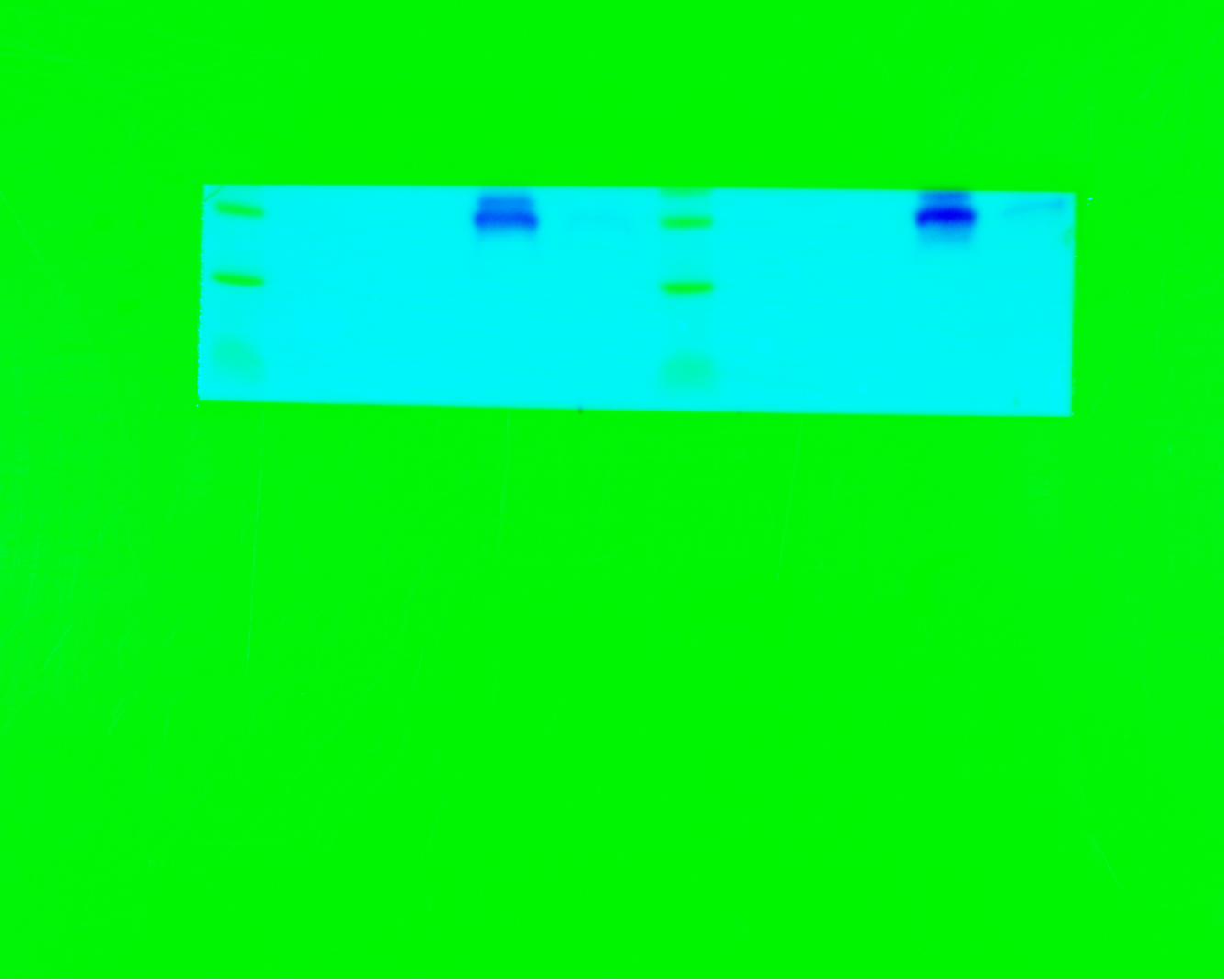

Supplement: Supplementary file 1 [file DataSheet3.ZIP › homo-LOP+b/cc3 (3).tif]

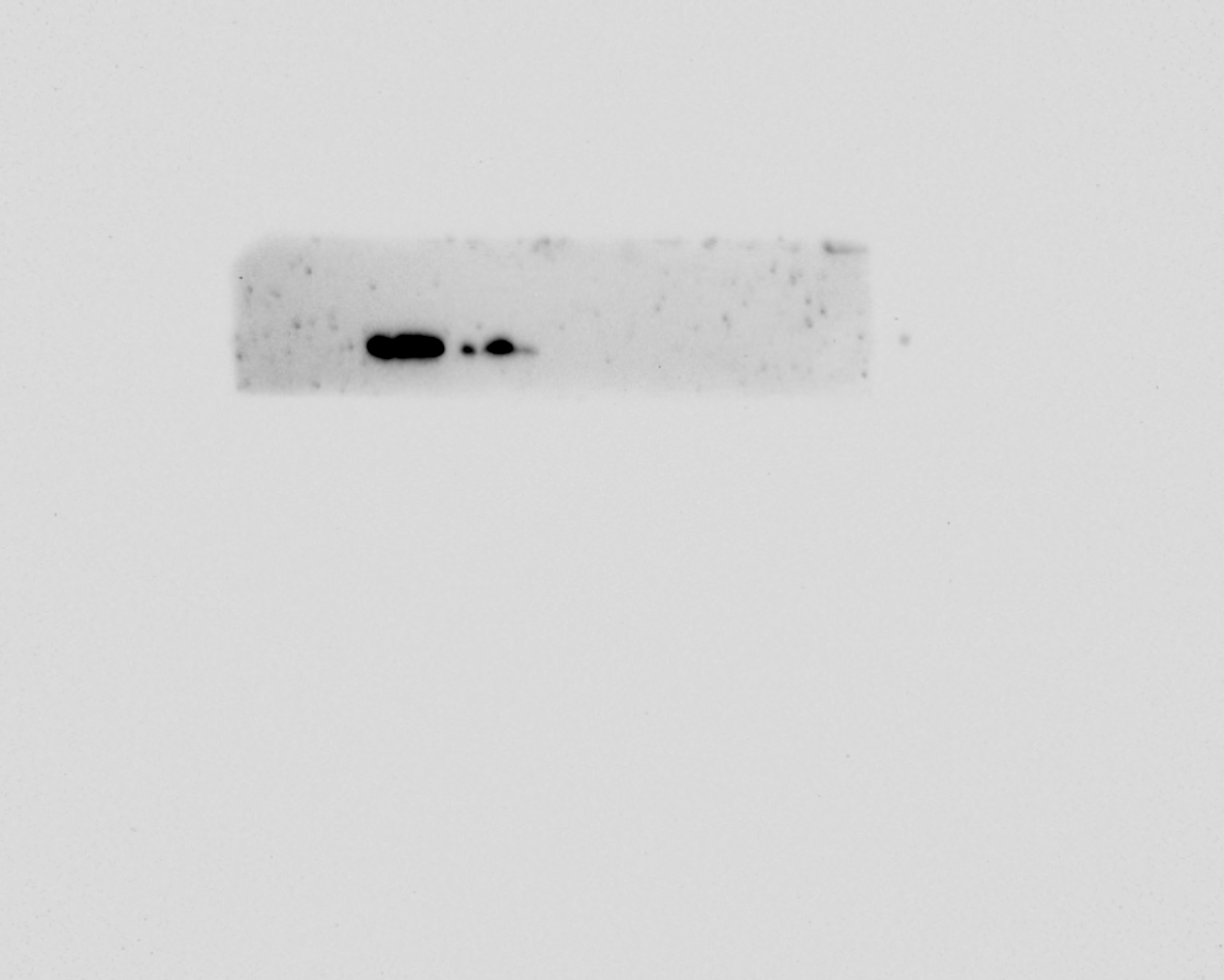

Supplement: Supplementary file 1 [file DataSheet3.ZIP › homo-LOP+b/lopbabpstat3_1(Chemiluminescence).tif]

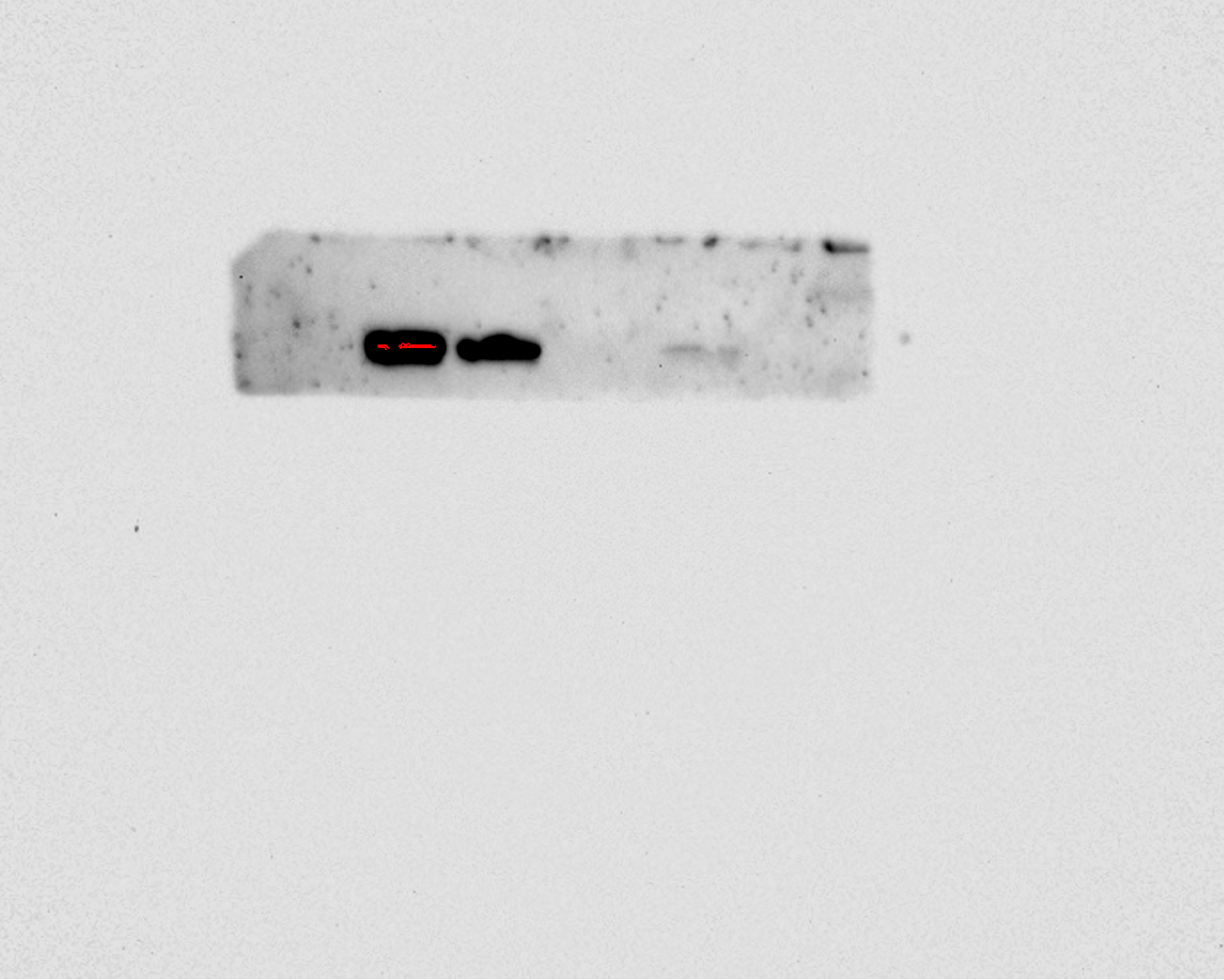

Supplement: Supplementary file 1 [file DataSheet3.ZIP › homo-LOP+b/lopbabpstat3_8(Chemiluminescence).tif]

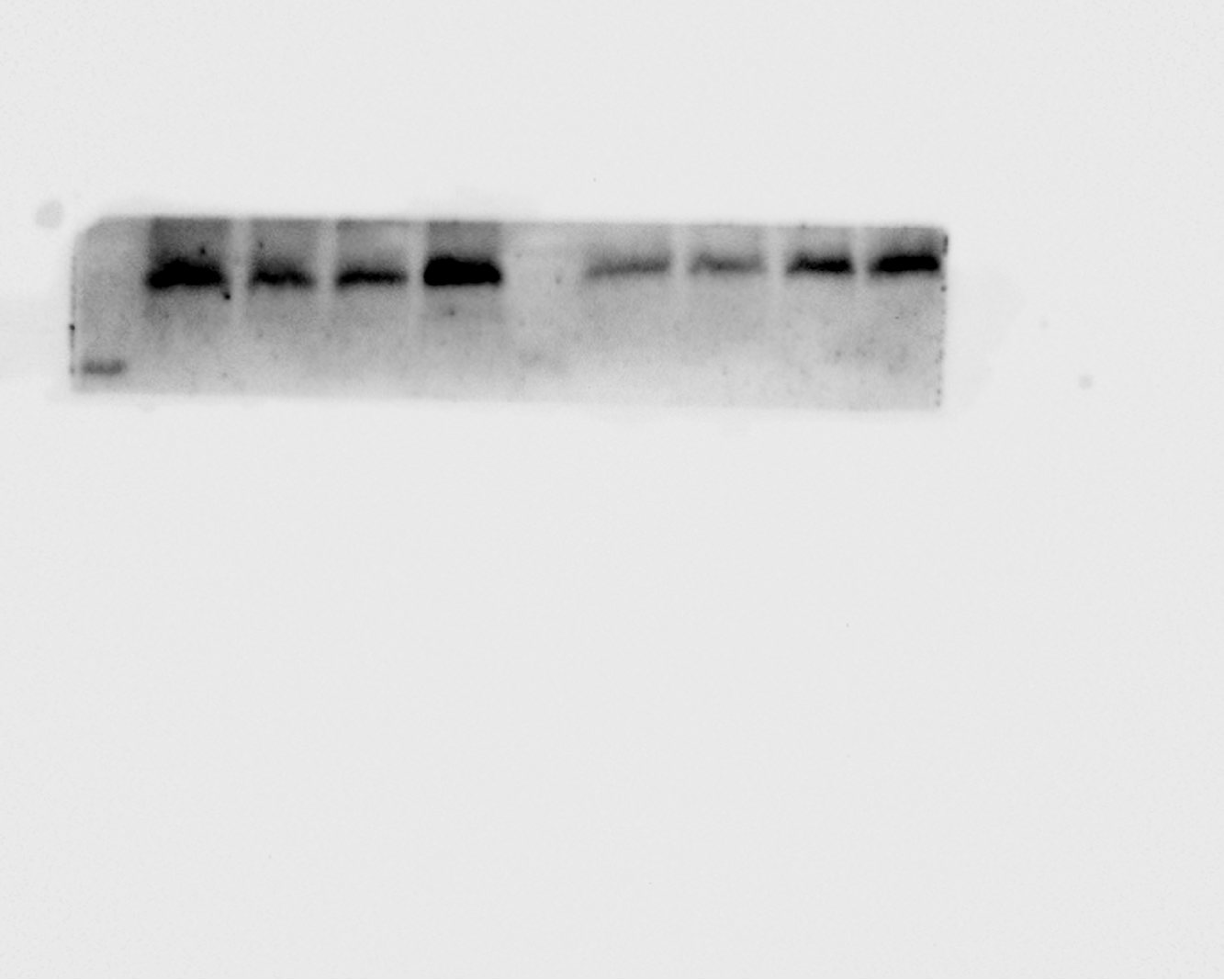

Supplement: Supplementary file 1 [file DataSheet3.ZIP › homo-LOP+b/p21_3(Chemiluminescence).tif]

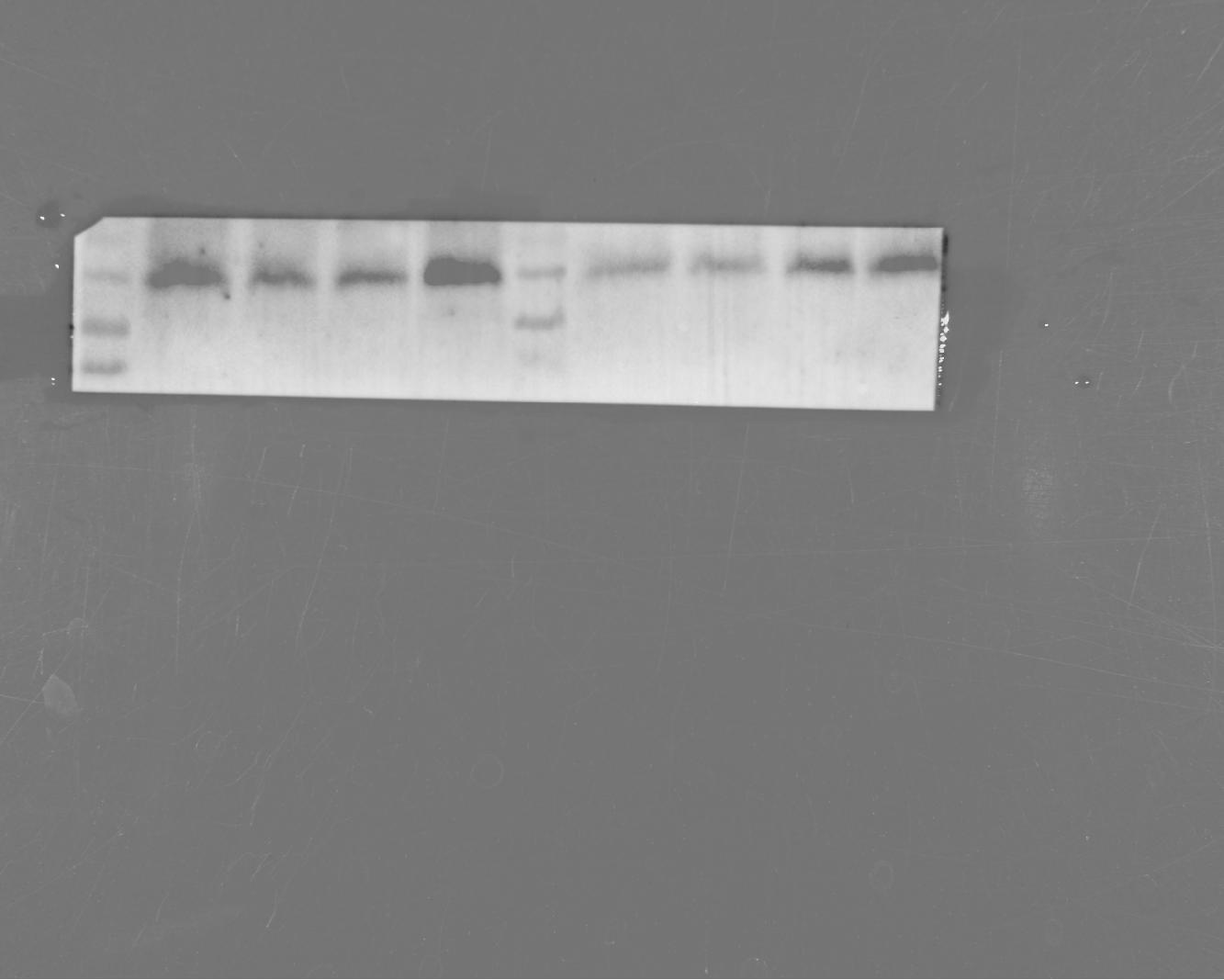

Supplement: Supplementary file 1 [file DataSheet3.ZIP › homo-LOP+b/p21_3(Composite).tif]

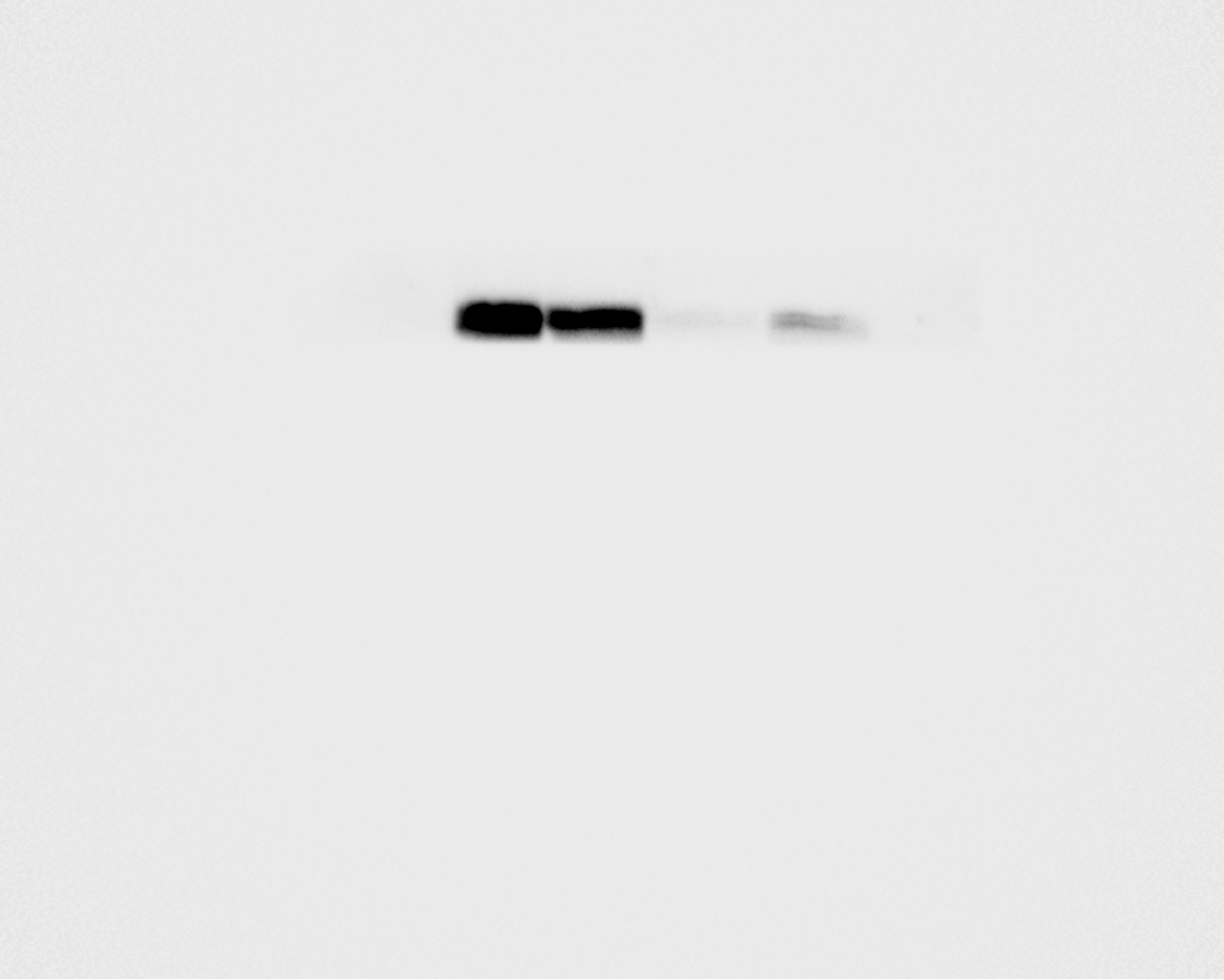

Supplement: Supplementary file 1 [file DataSheet3.ZIP › homo-LOP+b/perk_3(Chemiluminescence).tif]

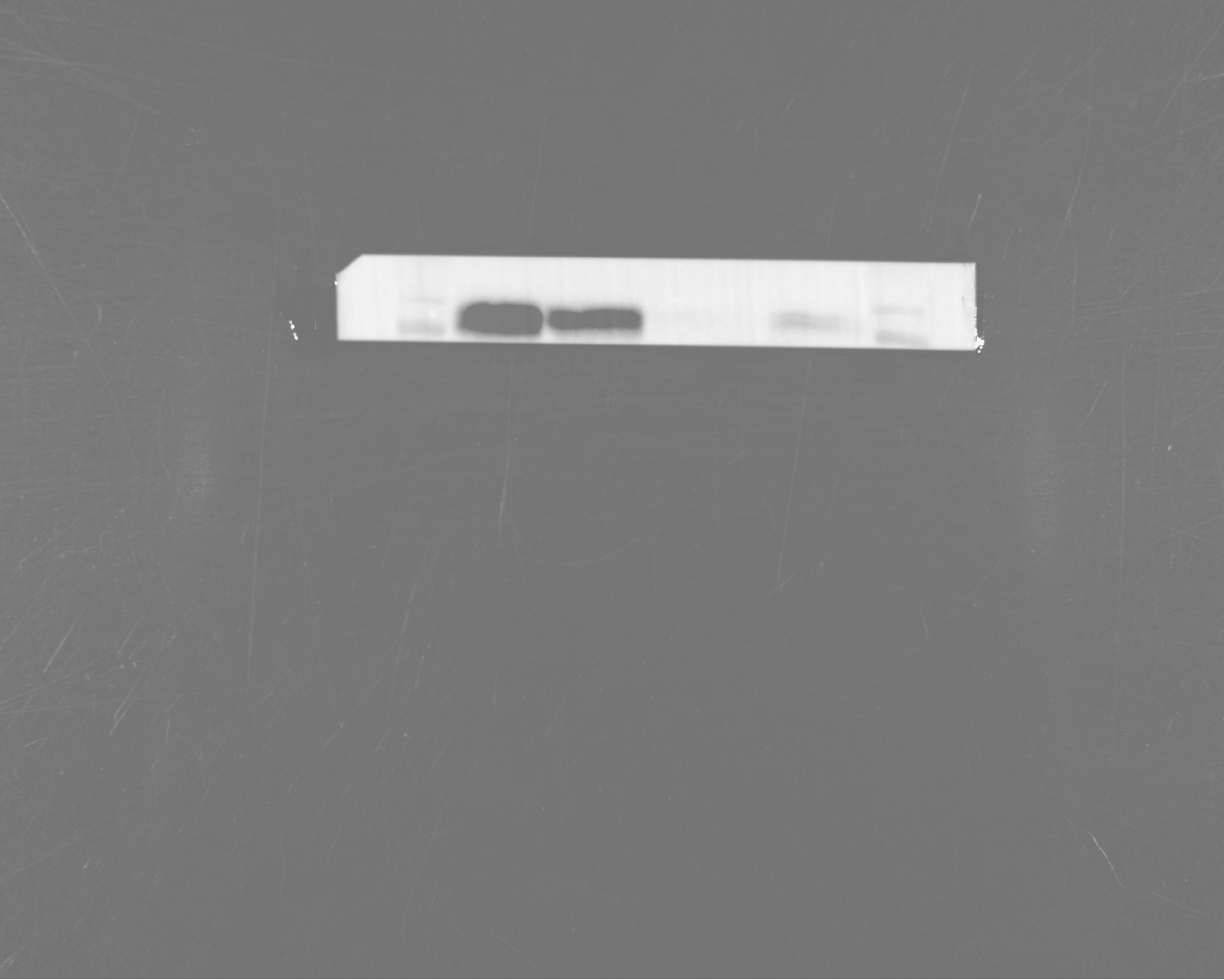

Supplement: Supplementary file 1 [file DataSheet3.ZIP › homo-LOP+b/perk_3(Composite).tif]

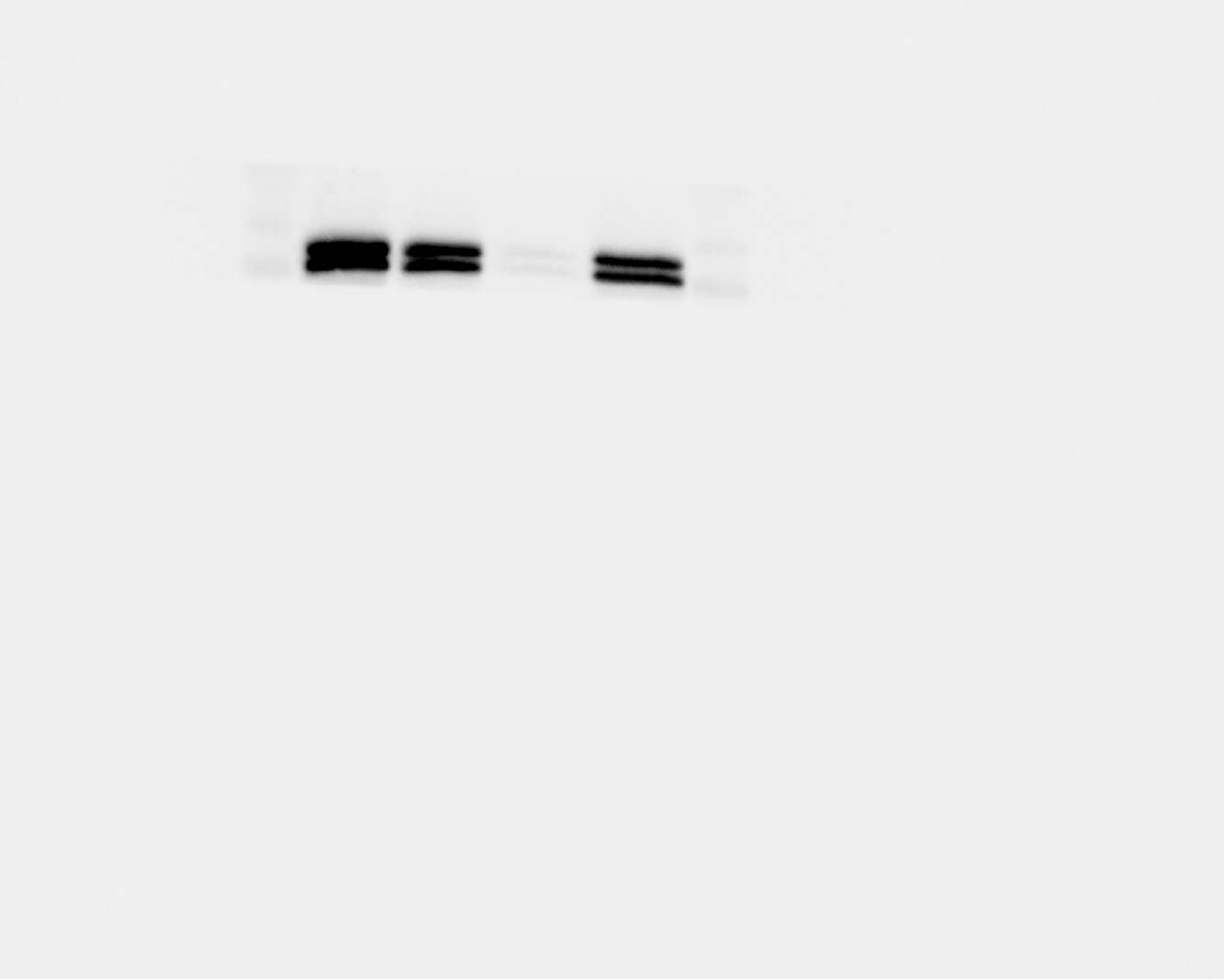

Supplement: Supplementary file 1 [file DataSheet3.ZIP › homo-LOP+b/perkicclop_3(Chemiluminescence).tif]

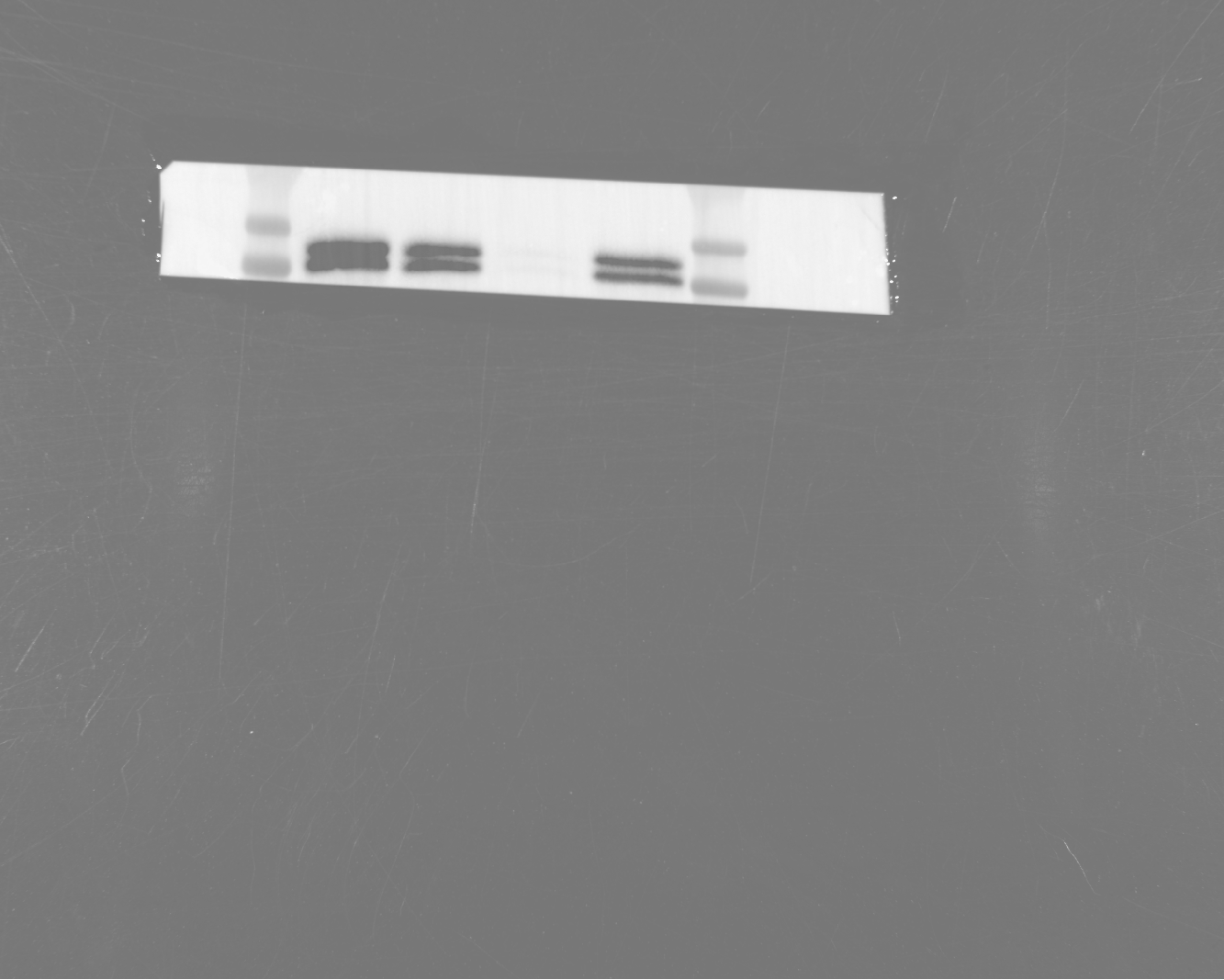

Supplement: Supplementary file 1 [file DataSheet3.ZIP › homo-LOP+b/perkicclop_3(Composite).tif]

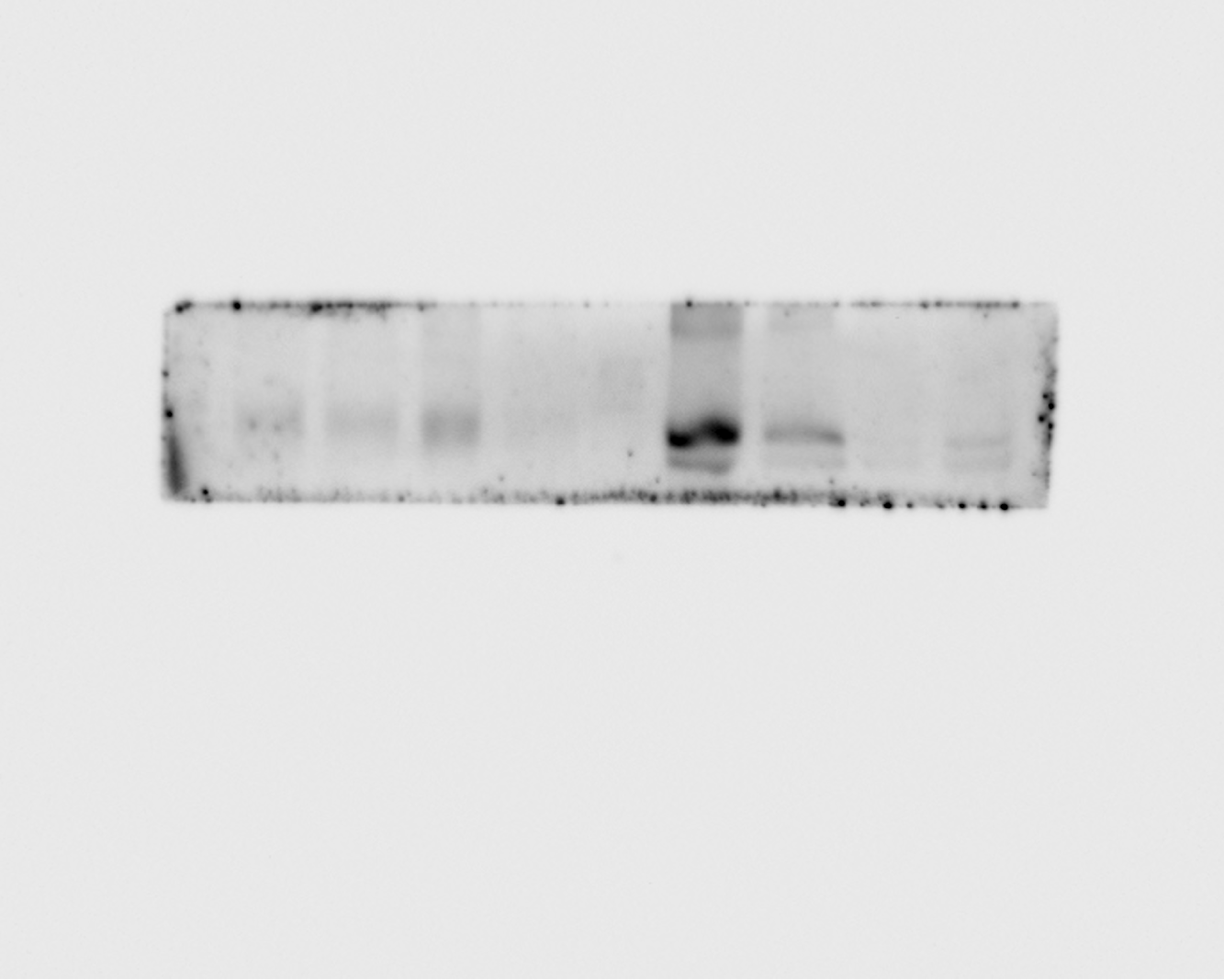

Supplement: Supplementary file 1 [file DataSheet3.ZIP › homo-LOP+b/ppstat31016_6(Chemiluminescence).tif]

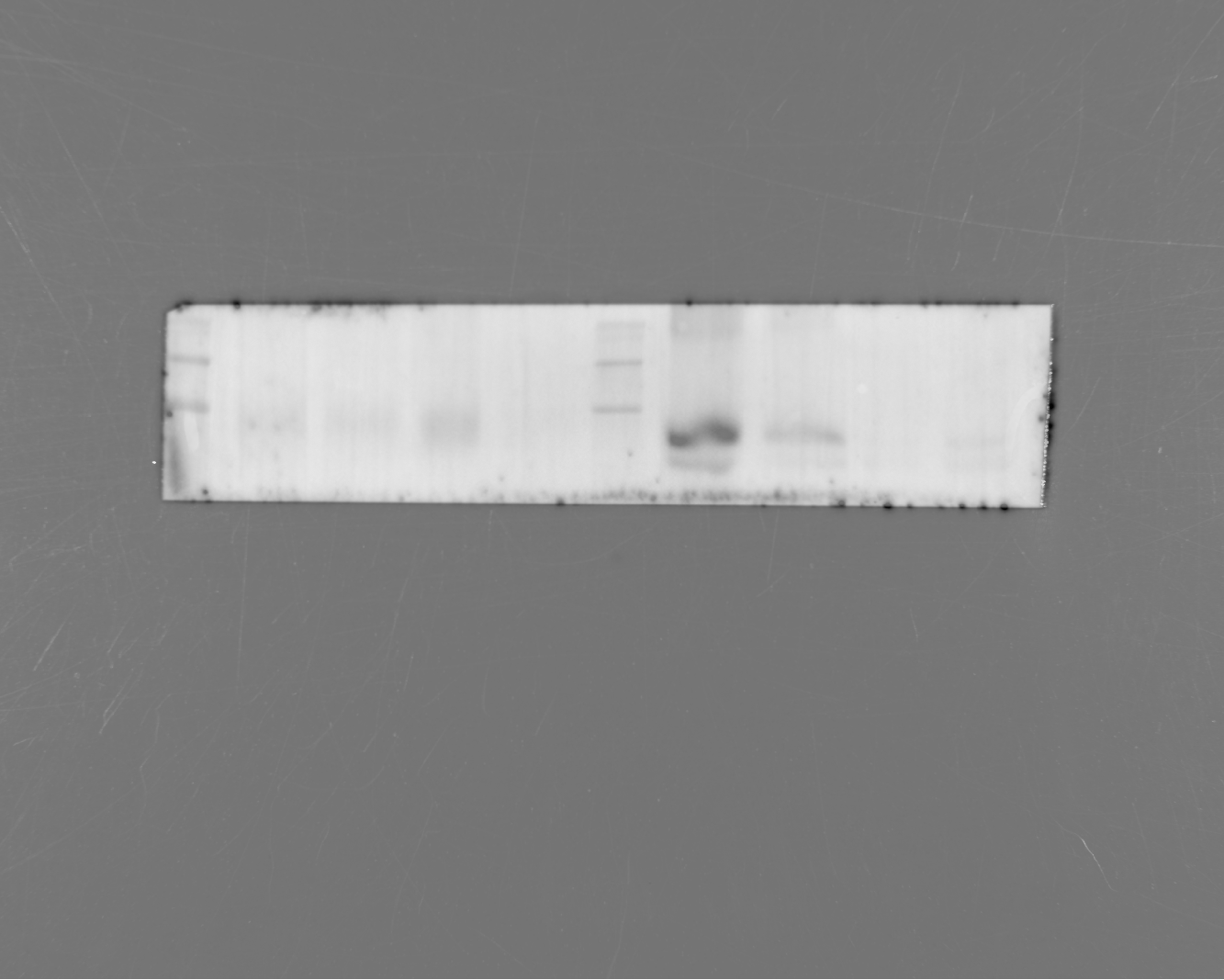

Supplement: Supplementary file 1 [file DataSheet3.ZIP › homo-LOP+b/ppstat31016_6(Composite).tif]

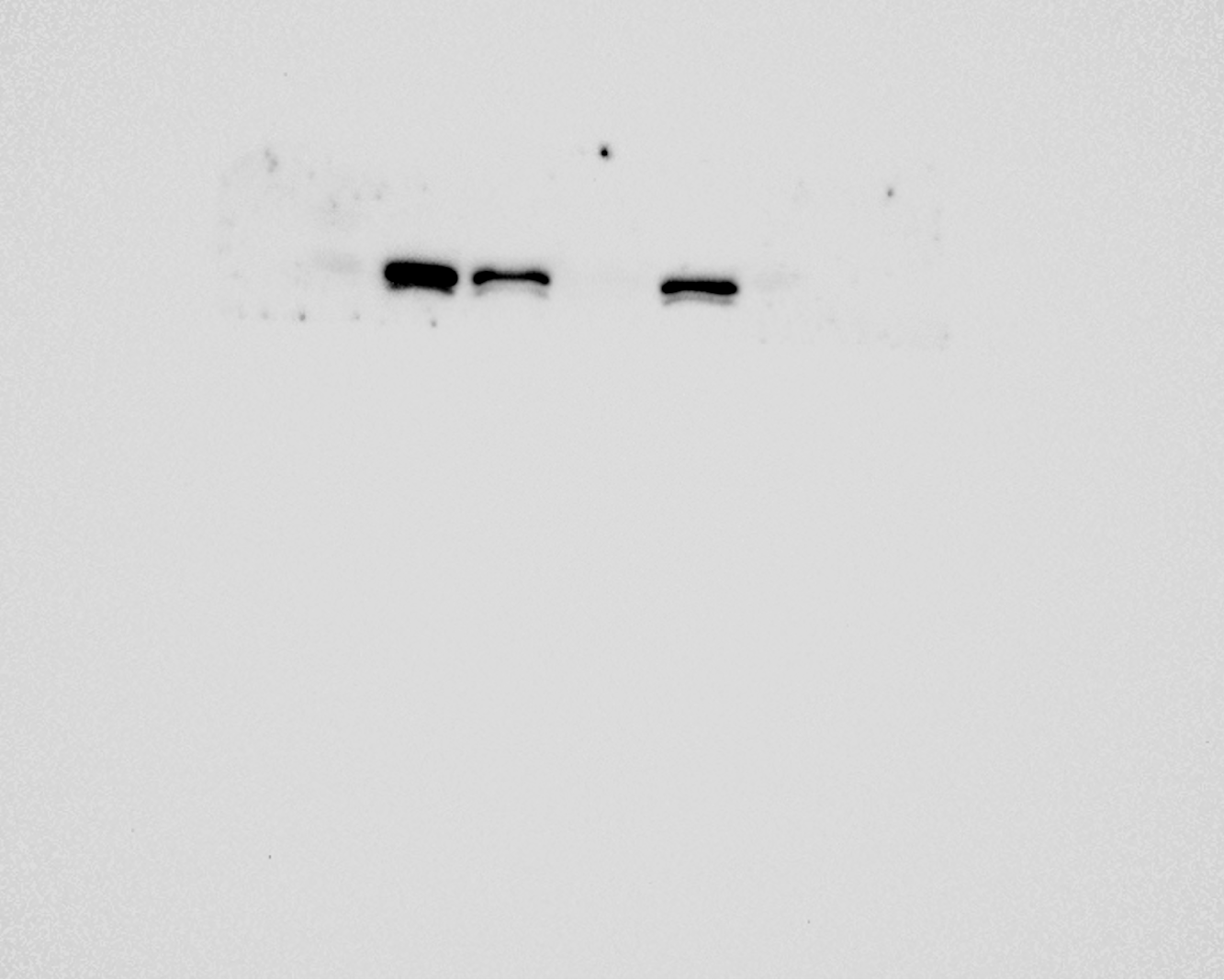

Supplement: Supplementary file 1 [file DataSheet3.ZIP › homo-LOP+b/pstat3_3(Chemiluminescence).tif]

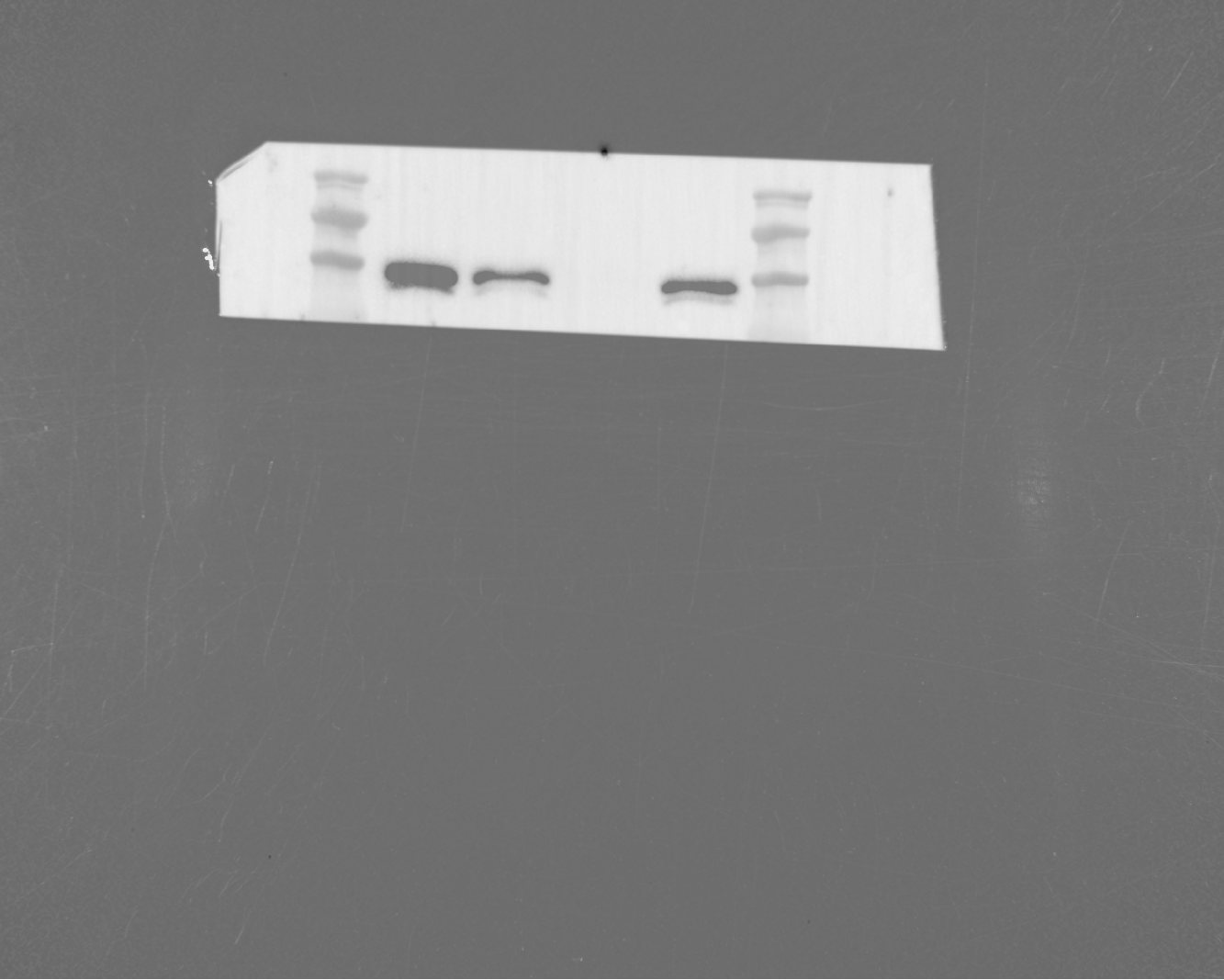

Supplement: Supplementary file 1 [file DataSheet3.ZIP › homo-LOP+b/pstat3_3(Composite).tif]

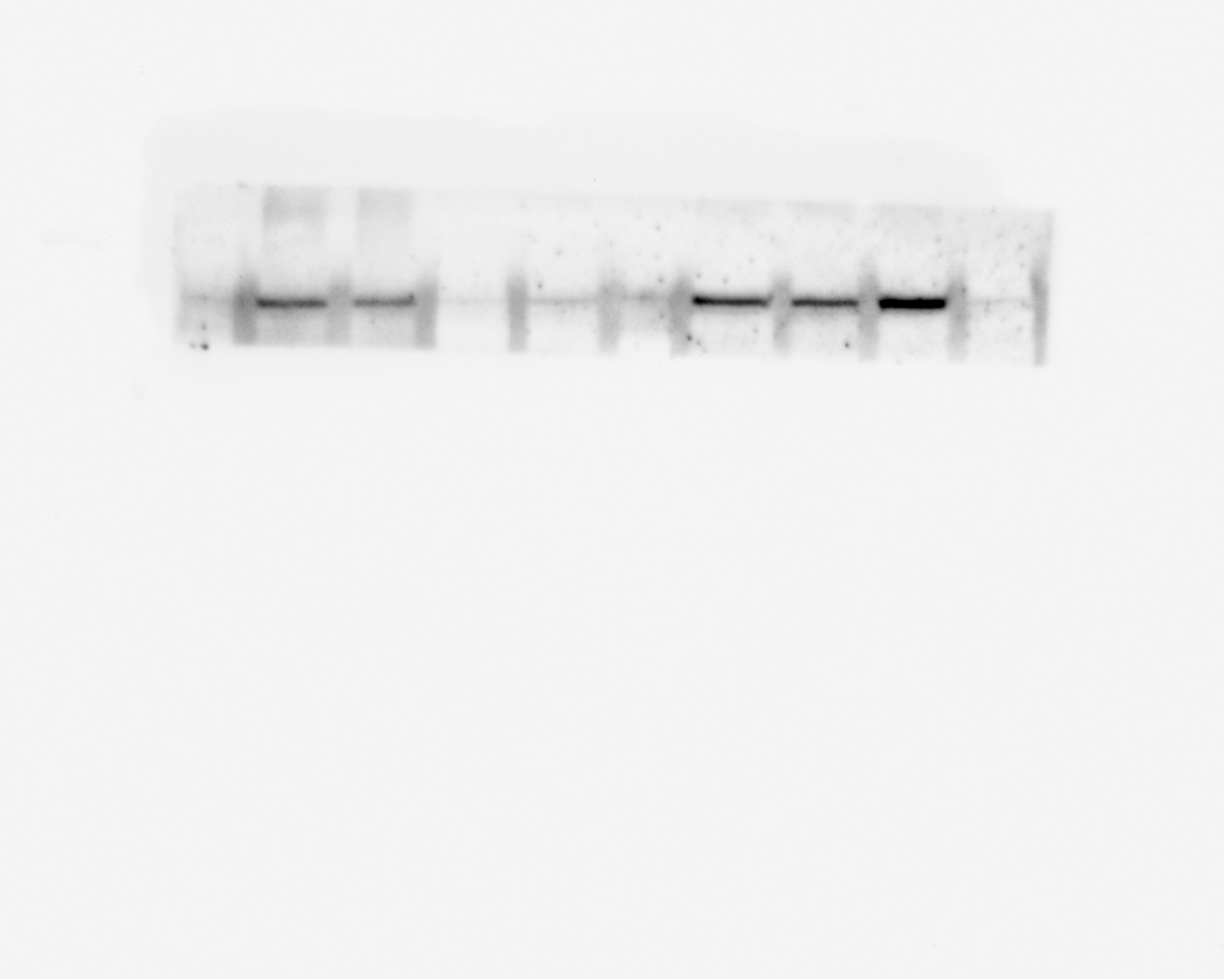

Supplement: Supplementary file 1 [file DataSheet3.ZIP › homo-LOP+b/pstat3-2_3(Chemiluminescence).tif]

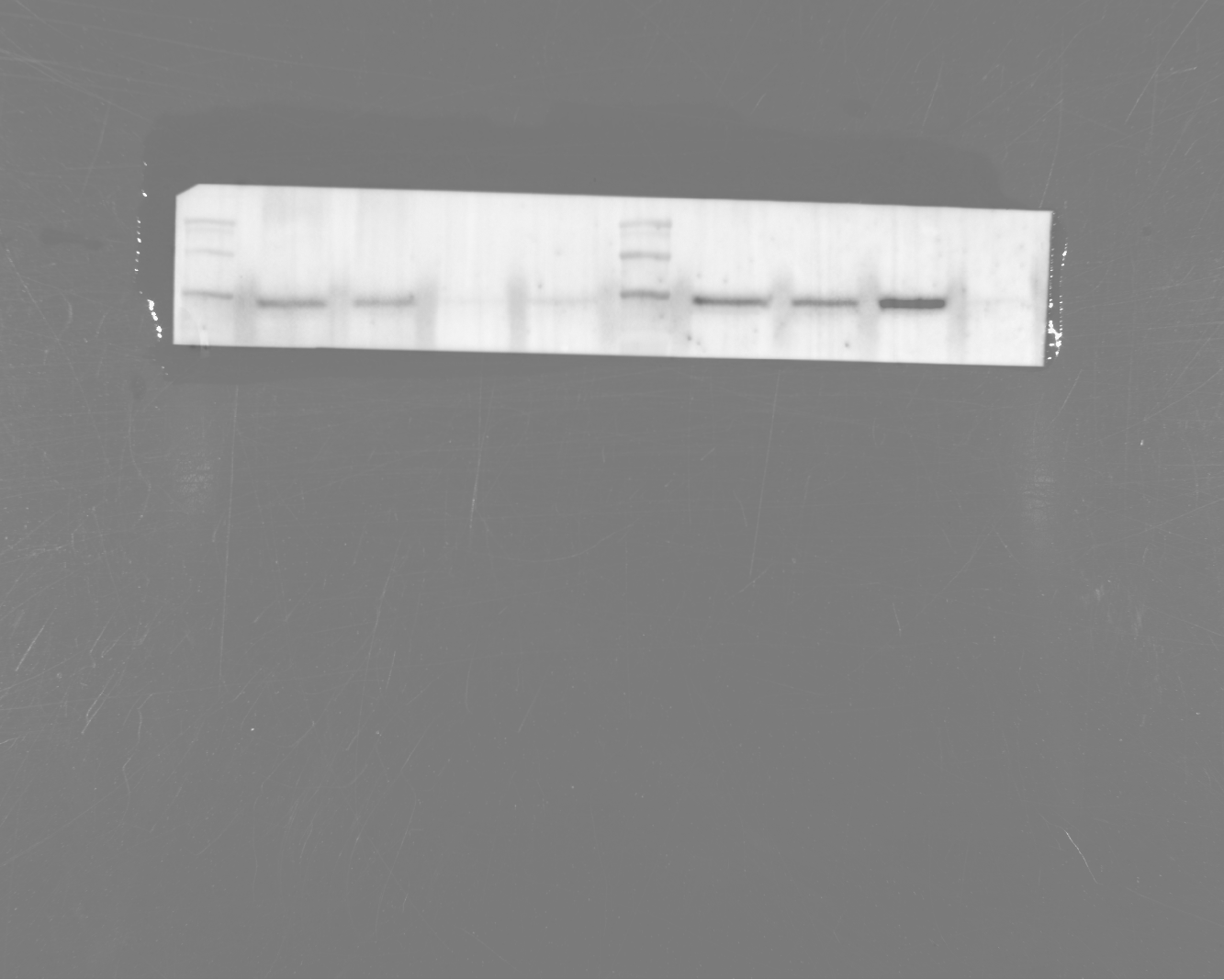

Supplement: Supplementary file 1 [file DataSheet3.ZIP › homo-LOP+b/pstat3-2_3(Composite).tif]

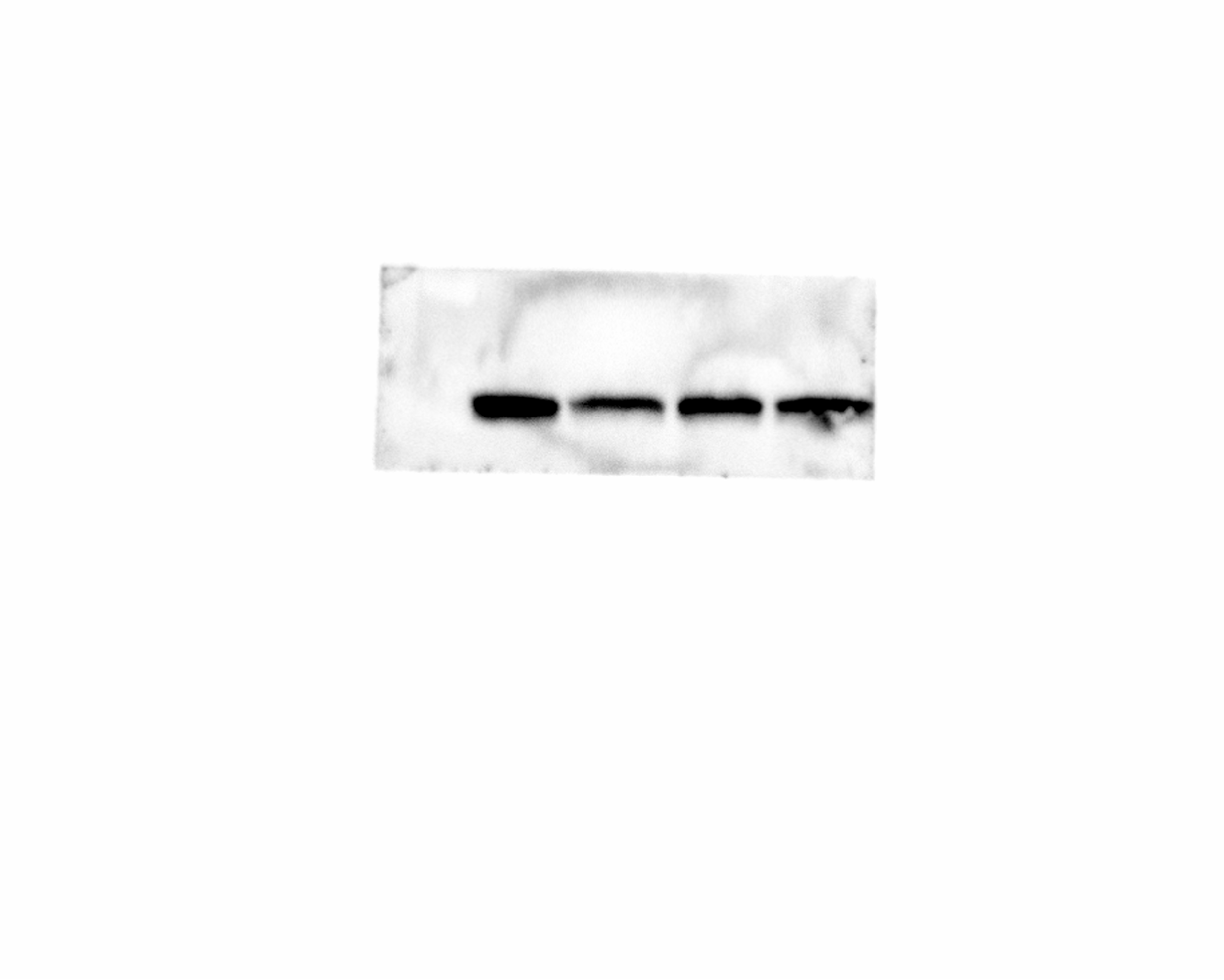

Supplement: Supplementary file 1 [file DataSheet3.ZIP › homo-LOP+b/stat3 (1).tif]

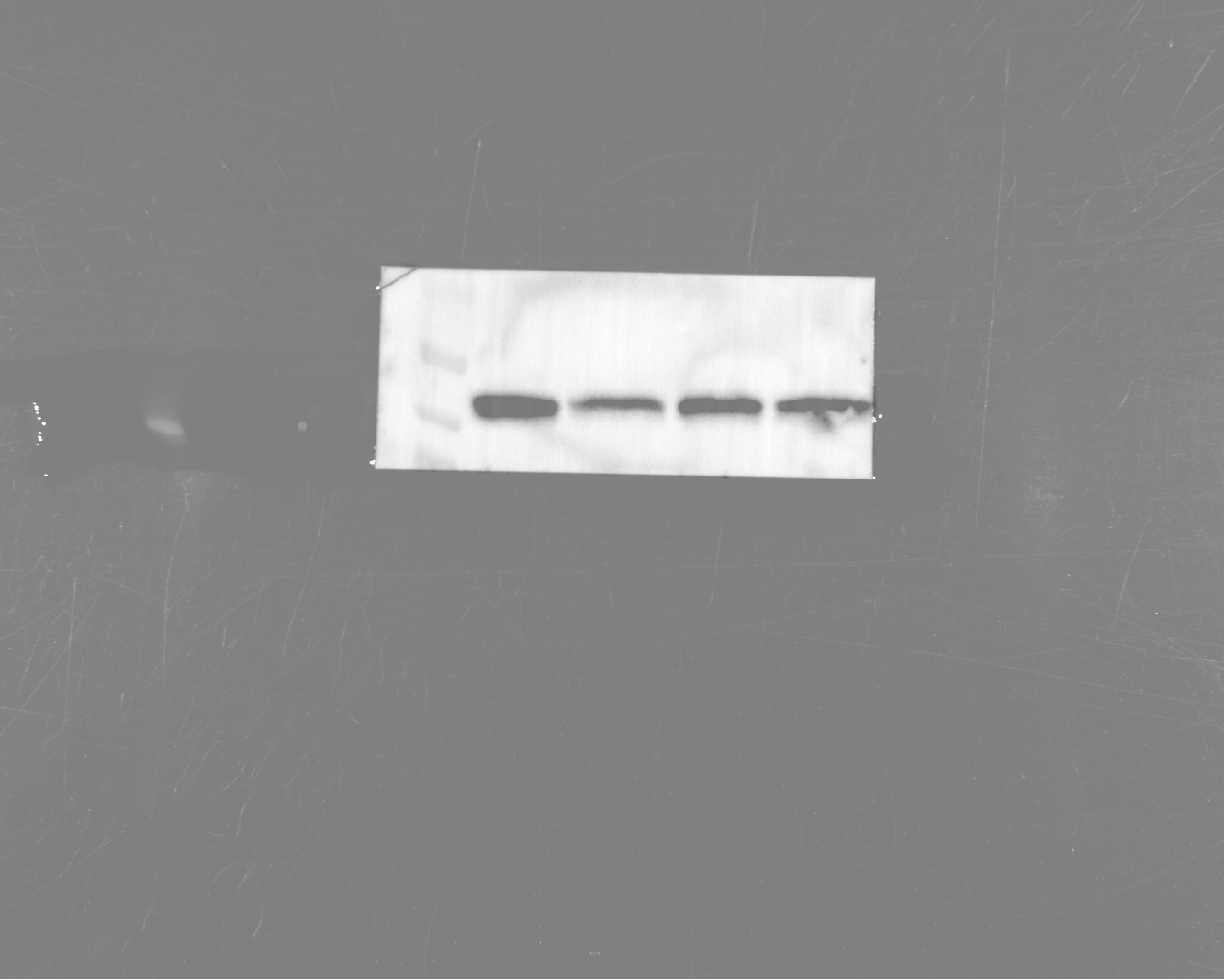

Supplement: Supplementary file 1 [file DataSheet3.ZIP › homo-LOP+b/stat3 (3).tif]

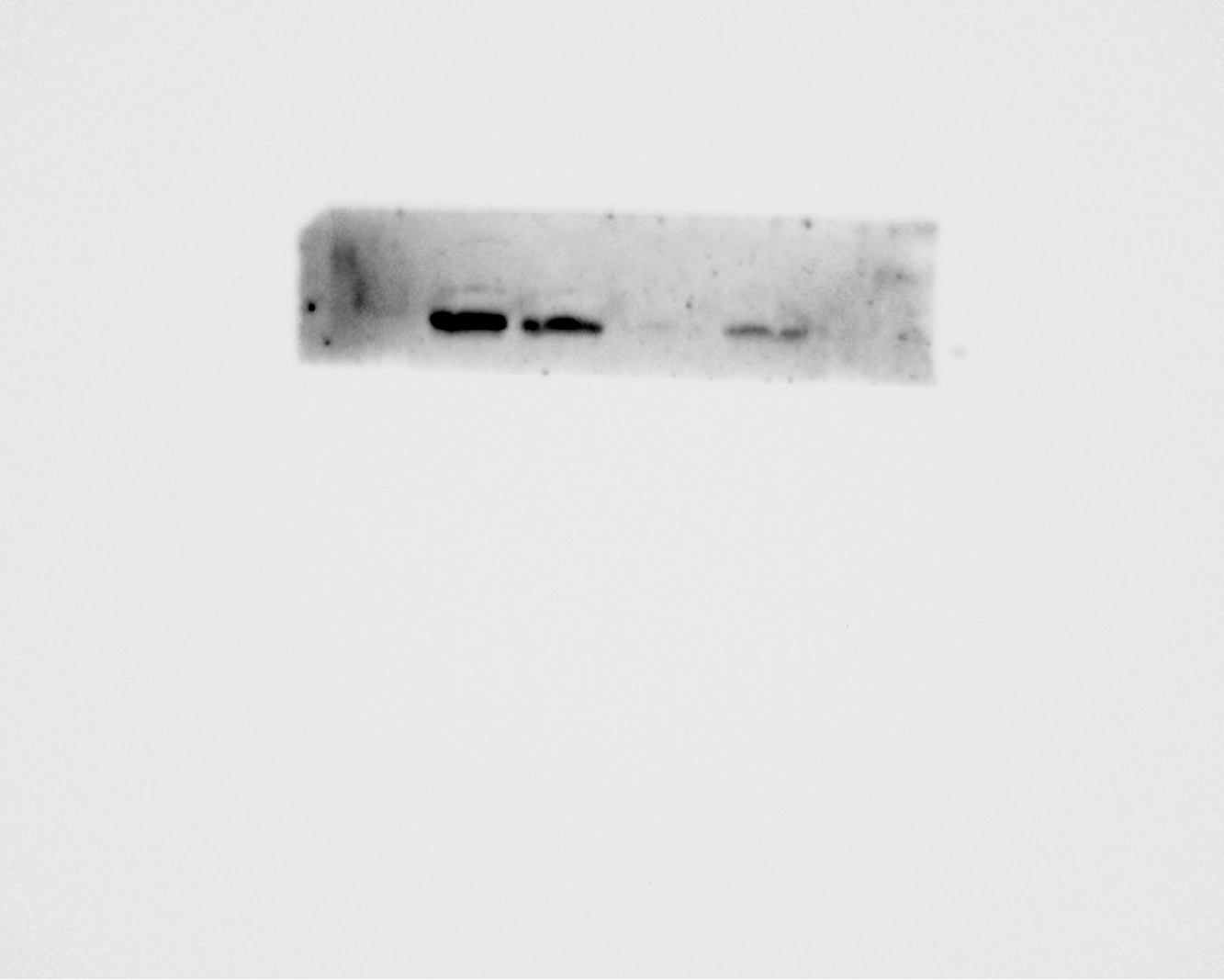

Supplement: Supplementary file 1 [file DataSheet3.ZIP › homo-LOP+b/stat3_3(Chemiluminescence).tif]

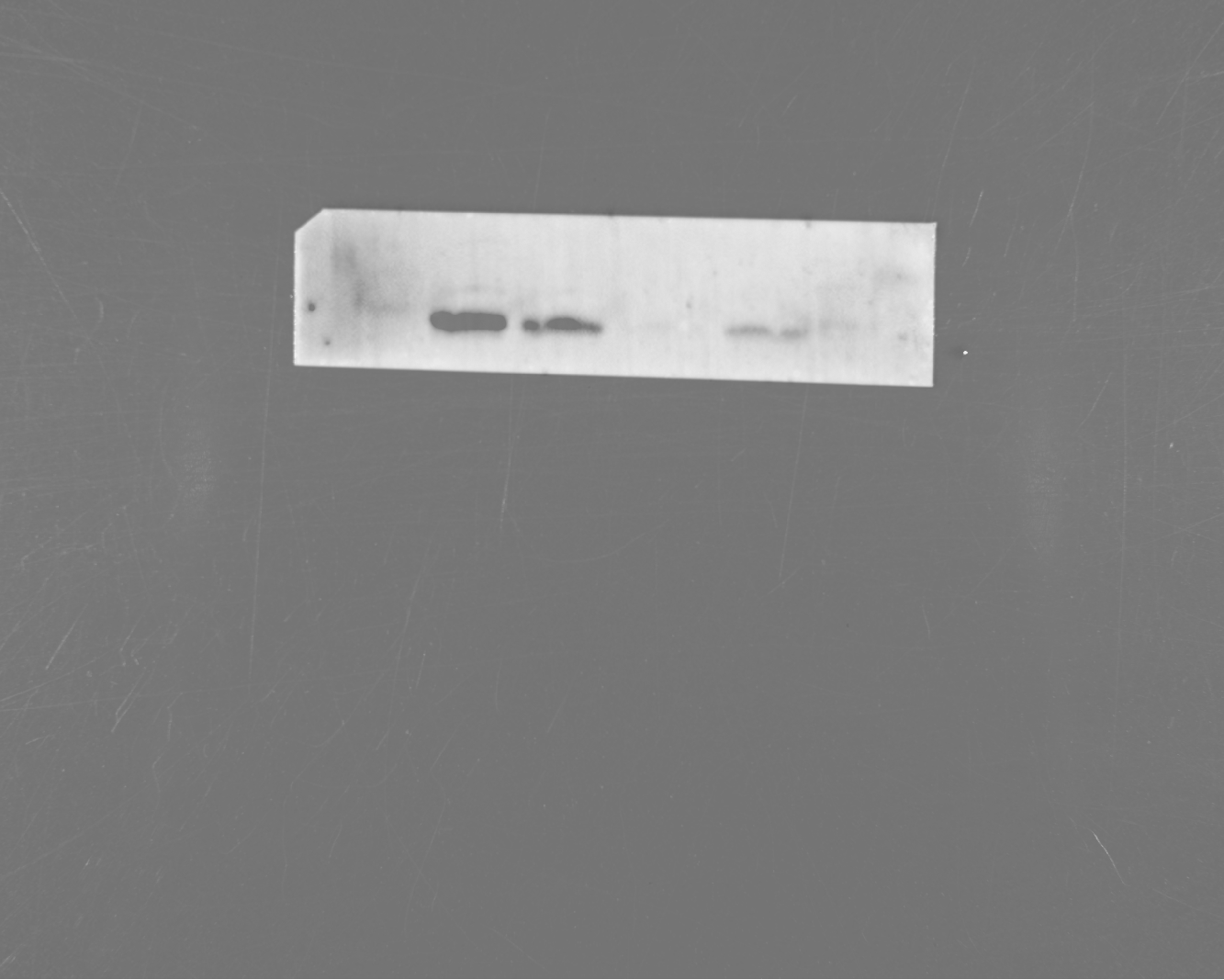

Supplement: Supplementary file 1 [file DataSheet3.ZIP › homo-LOP+b/stat3_3(Composite).tif]

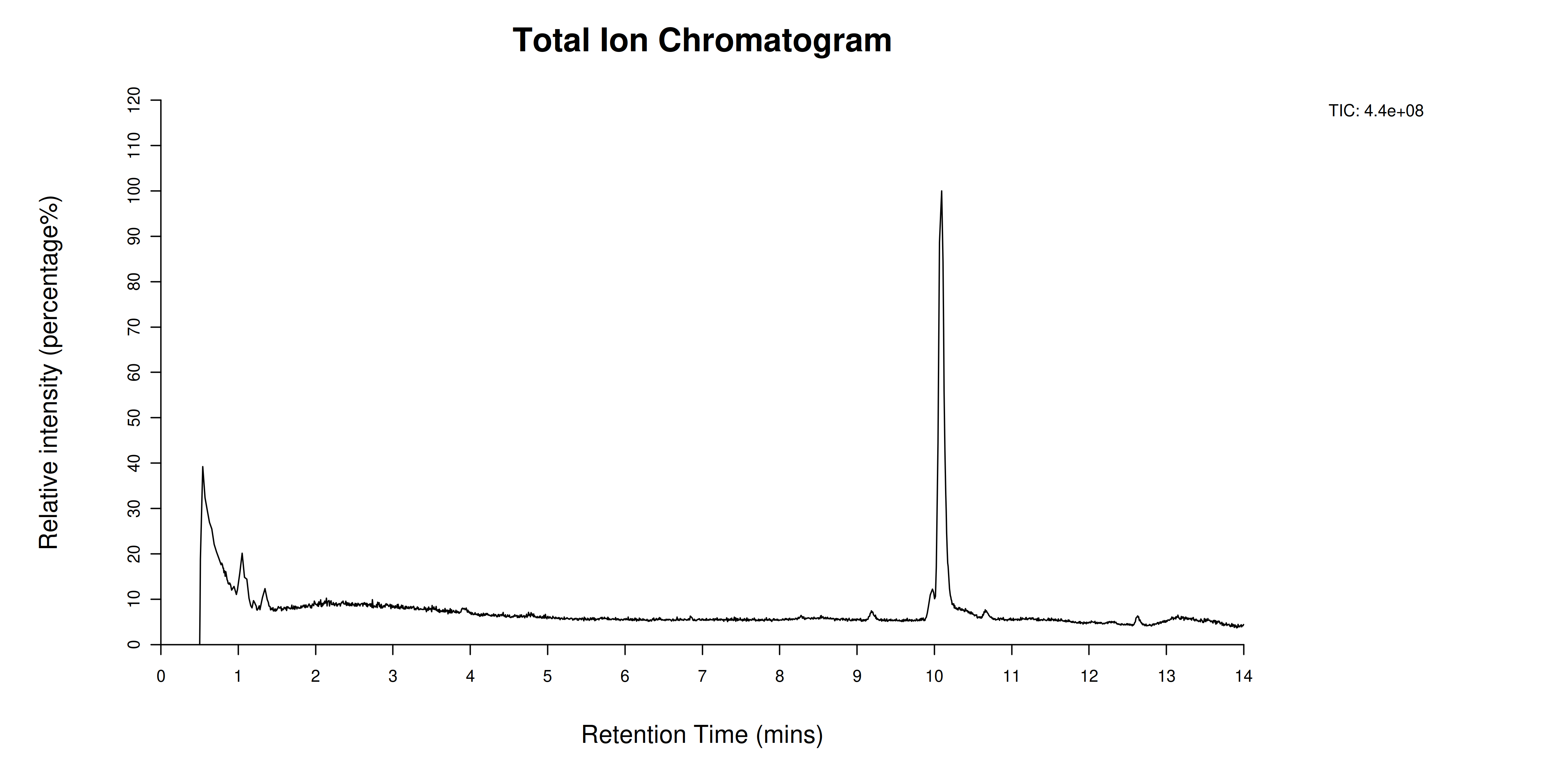

Supplement: Supplementary file 2 [file DataSheet11.ZIP › Astragaloside II.png]

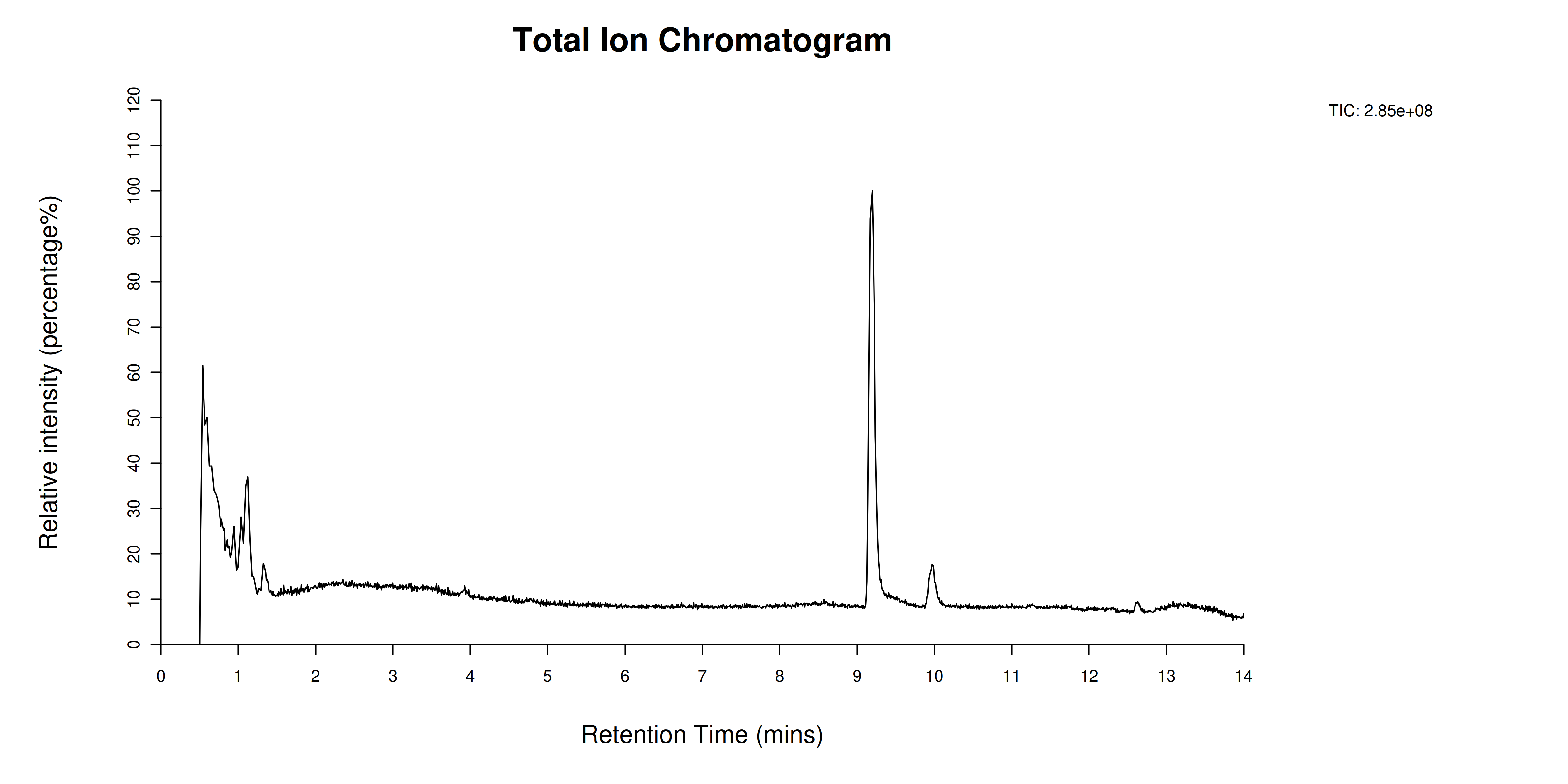

Supplement: Supplementary file 2 [file DataSheet11.ZIP › Astragaloside IV.png]

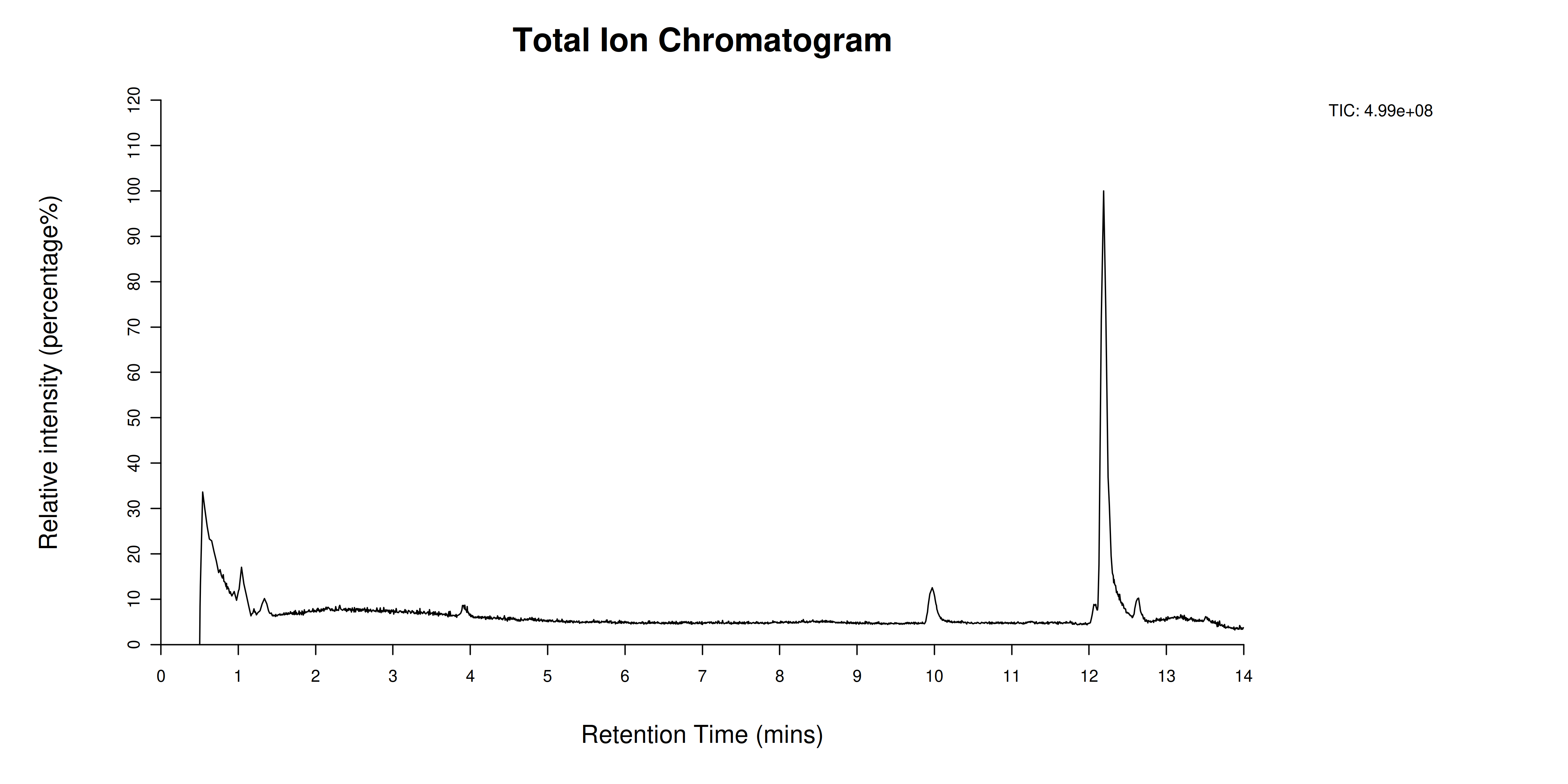

Supplement: Supplementary file 2 [file DataSheet11.ZIP › Emodin.png]

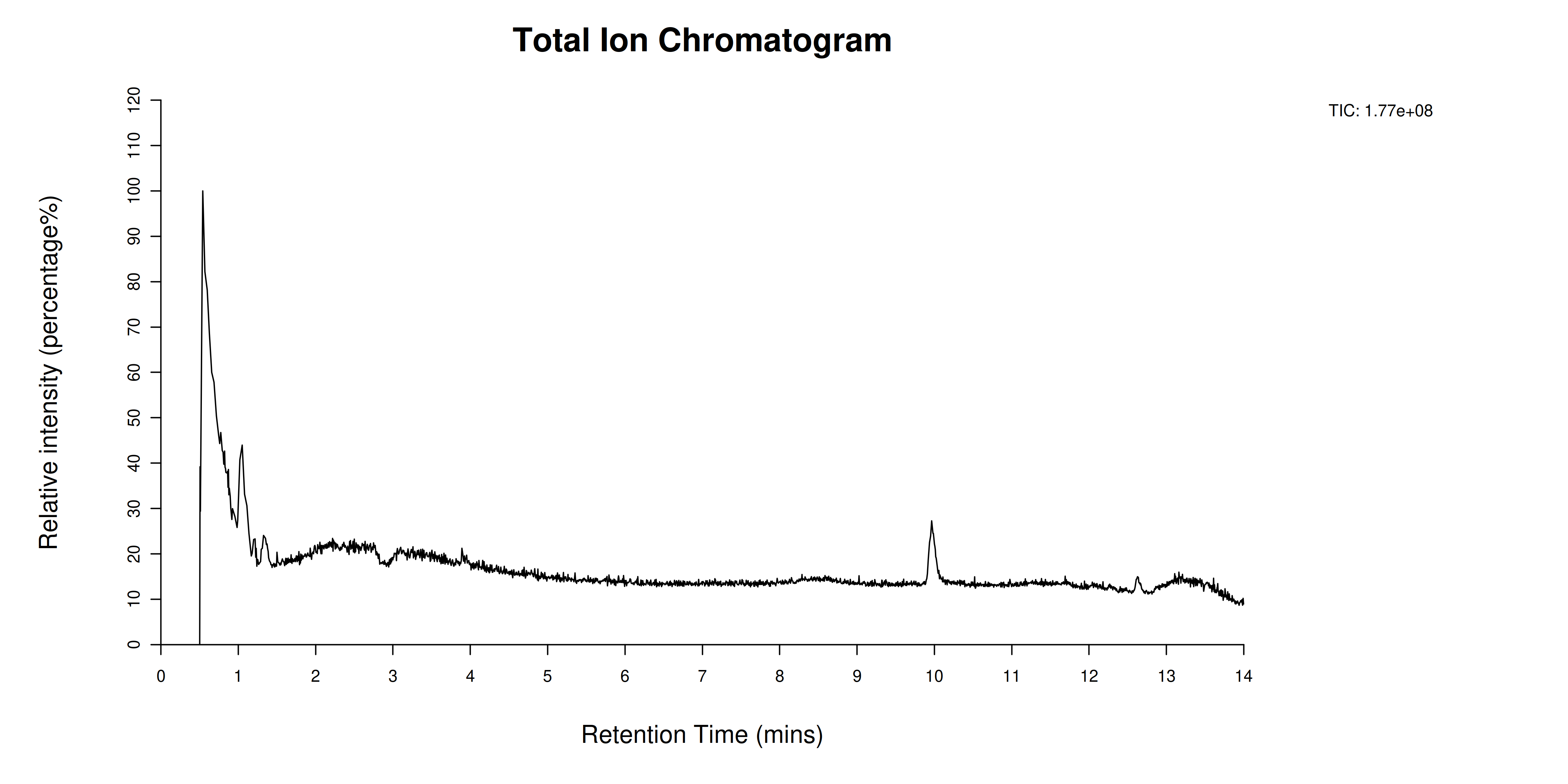

Supplement: Supplementary file 2 [file DataSheet11.ZIP › Ferulic Acid.png]

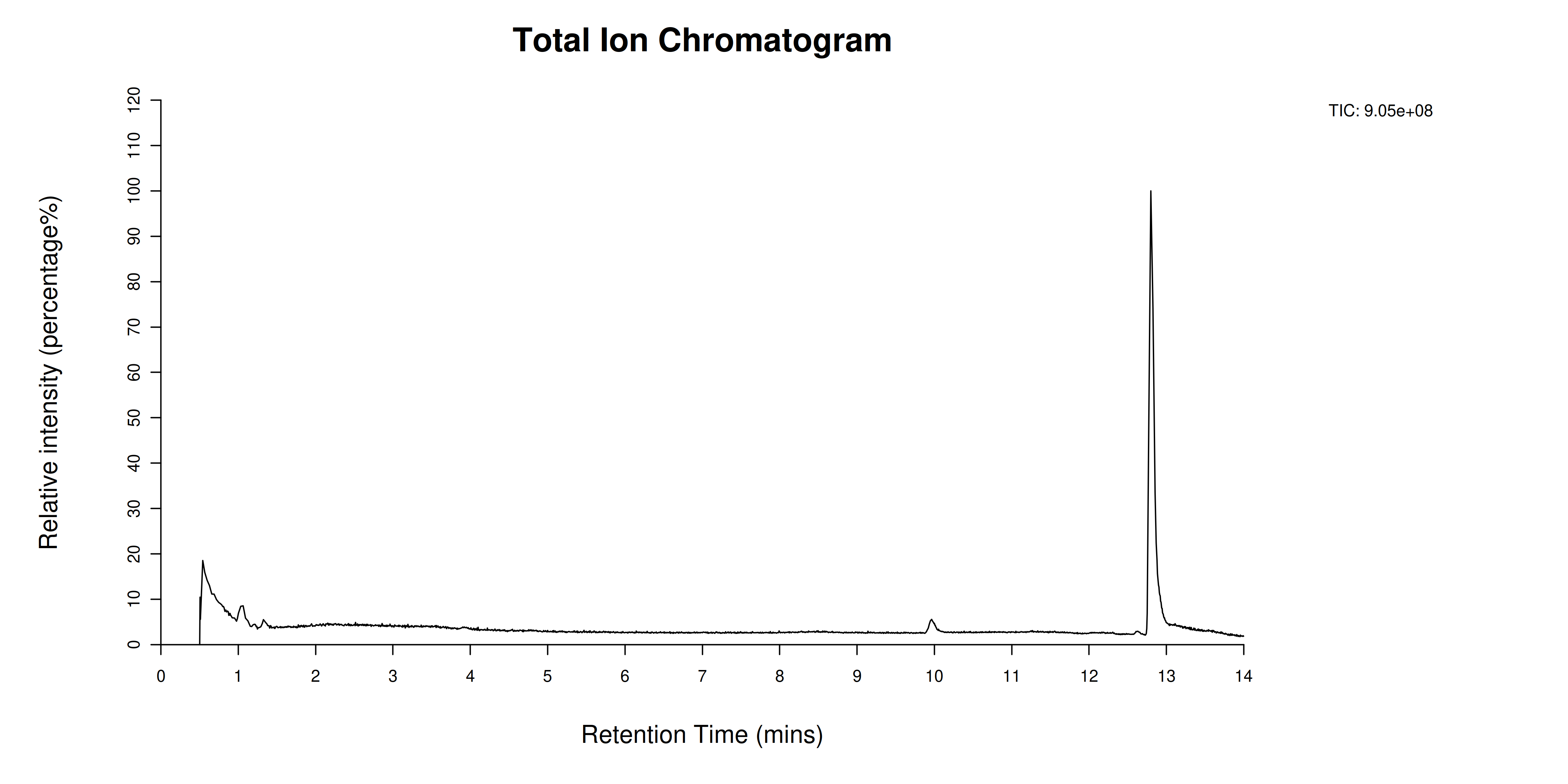

Supplement: Supplementary file 2 [file DataSheet11.ZIP › Magnolol.png]

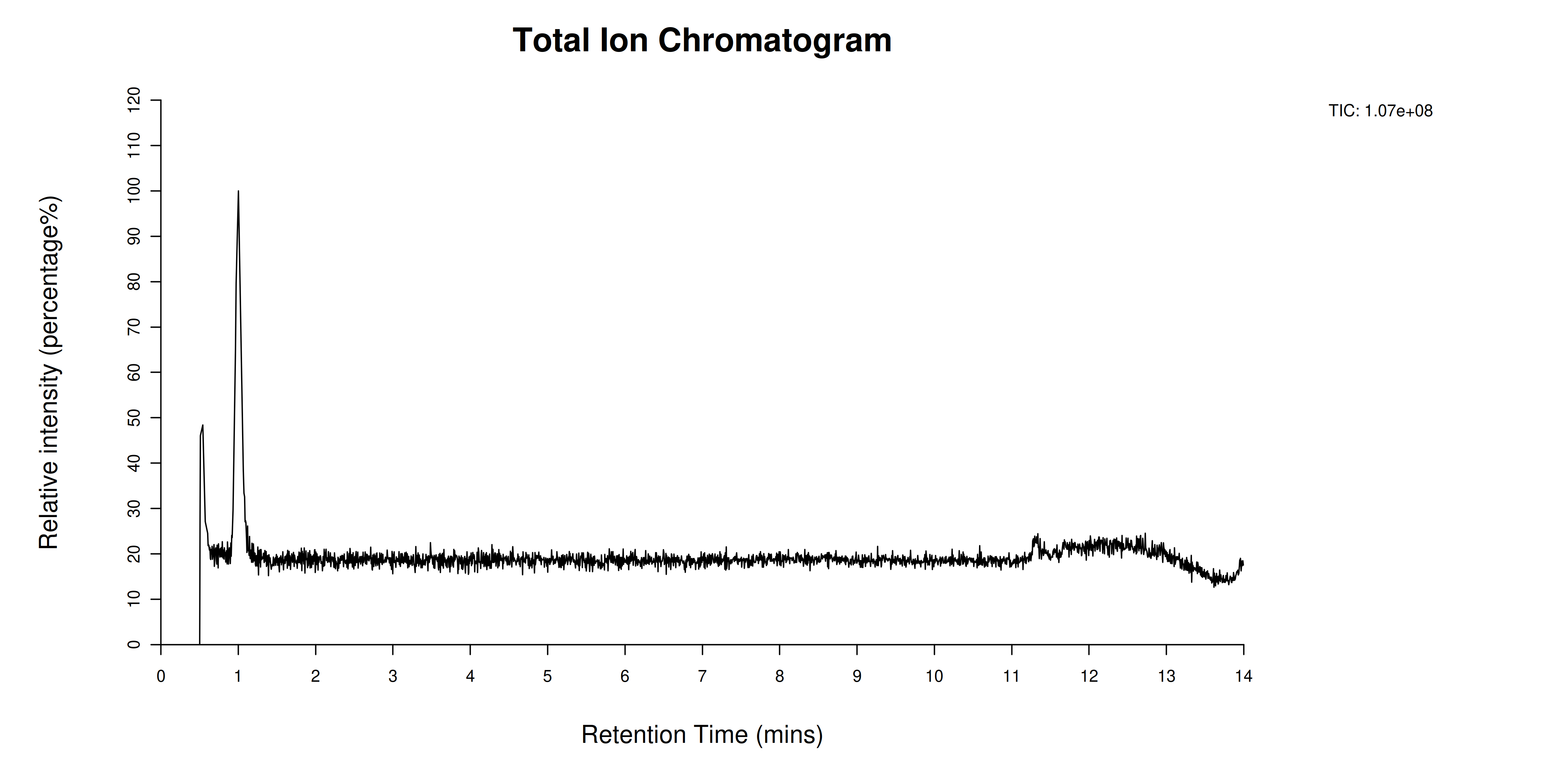

Supplement: Supplementary file 2 [file DataSheet11.ZIP › Rehmannia D.png]

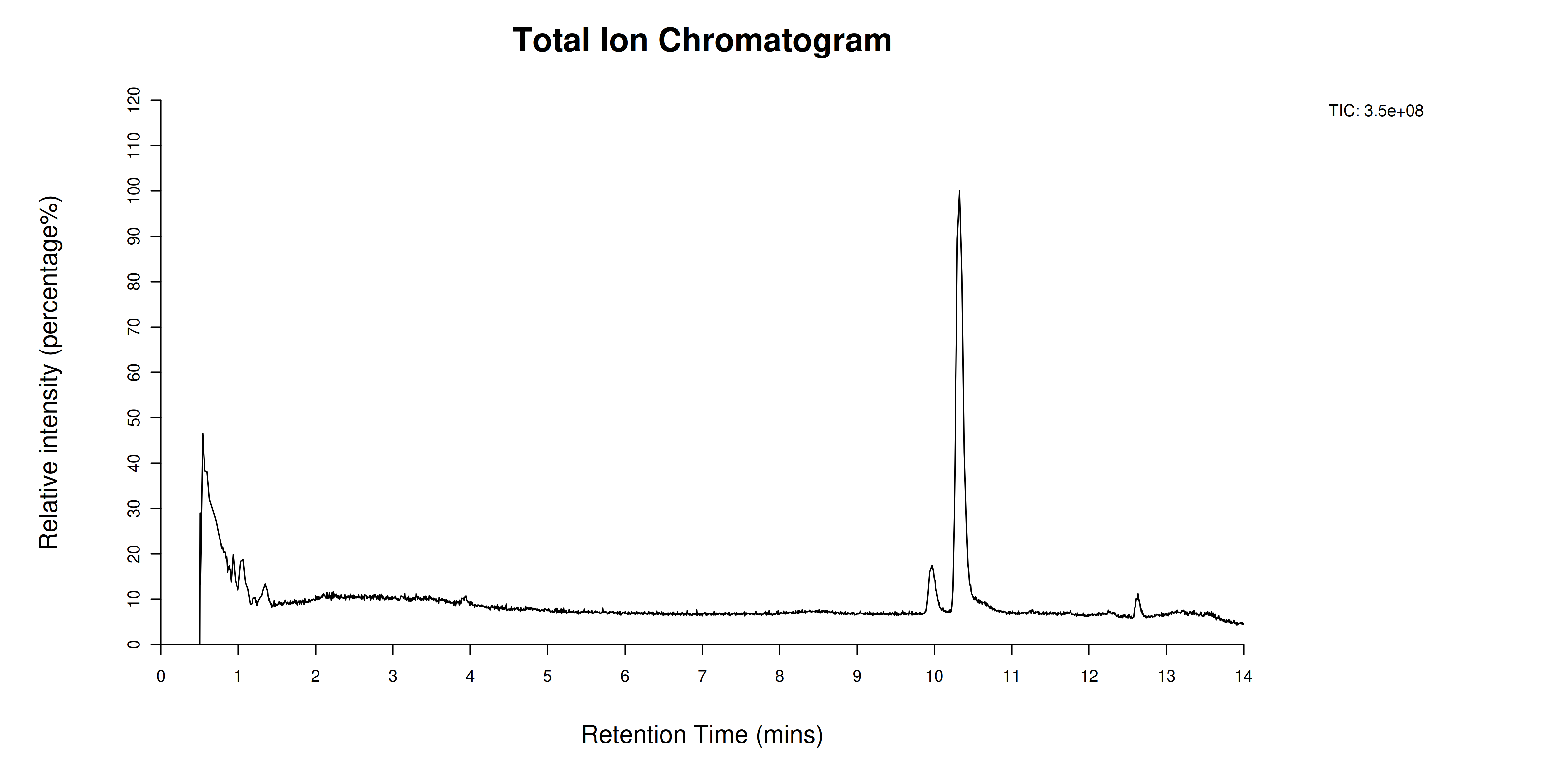

Supplement: Supplementary file 2 [file DataSheet11.ZIP › Rhein.png]

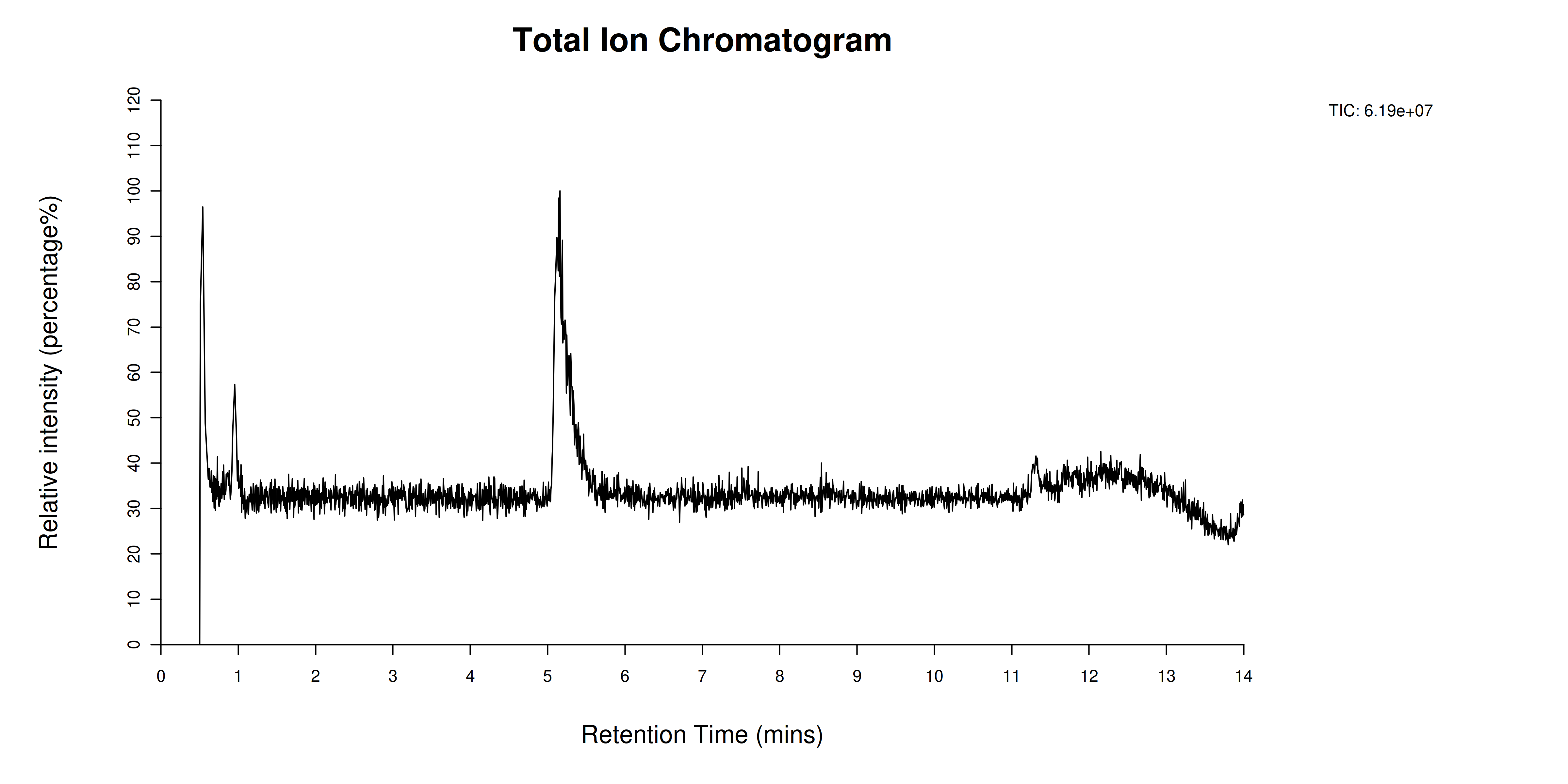

Supplement: Supplementary file 2 [file DataSheet11.ZIP › Salvianolic Acid B.png]

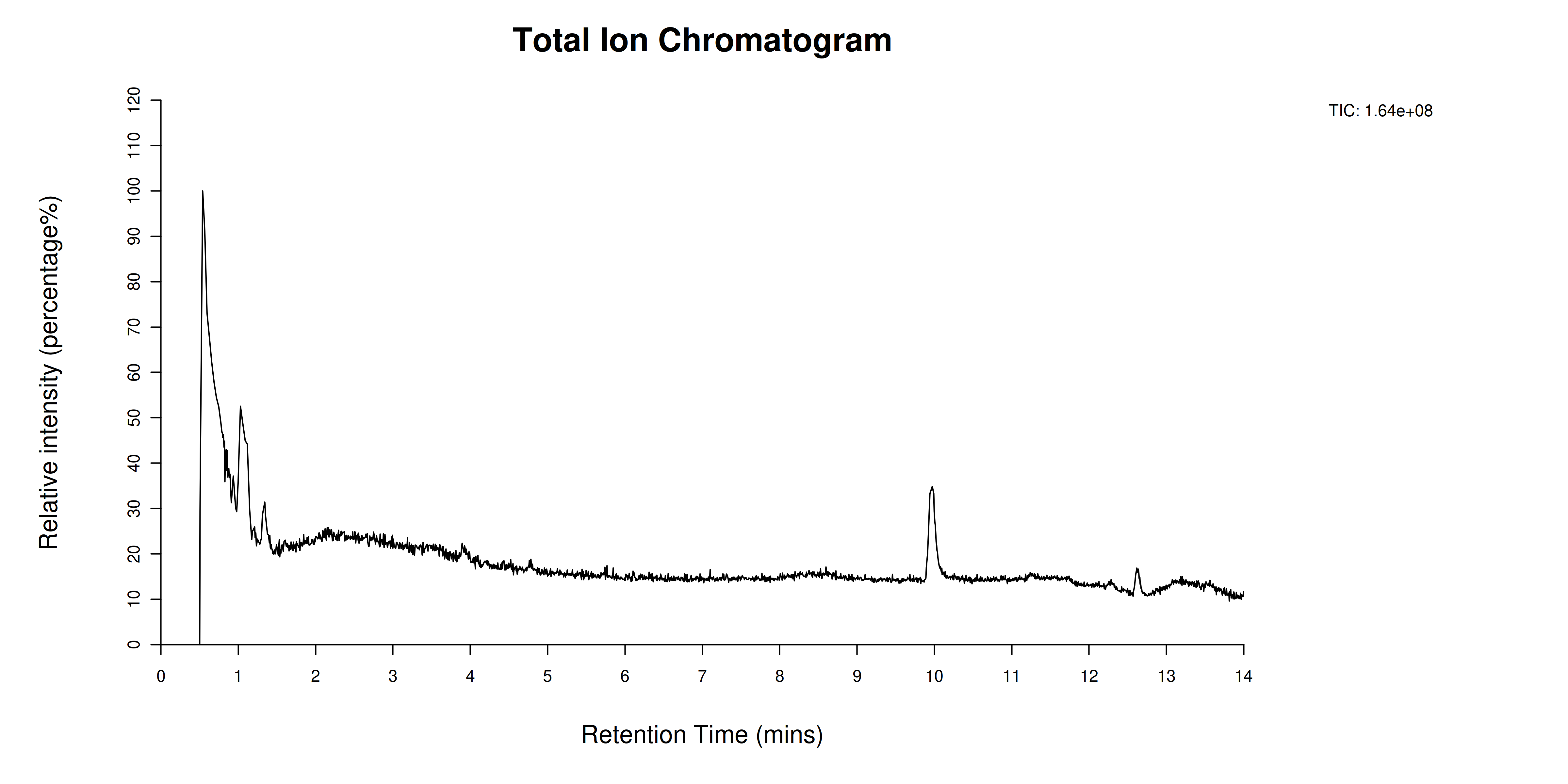

Supplement: Supplementary file 2 [file DataSheet11.ZIP › Synephrine.png]

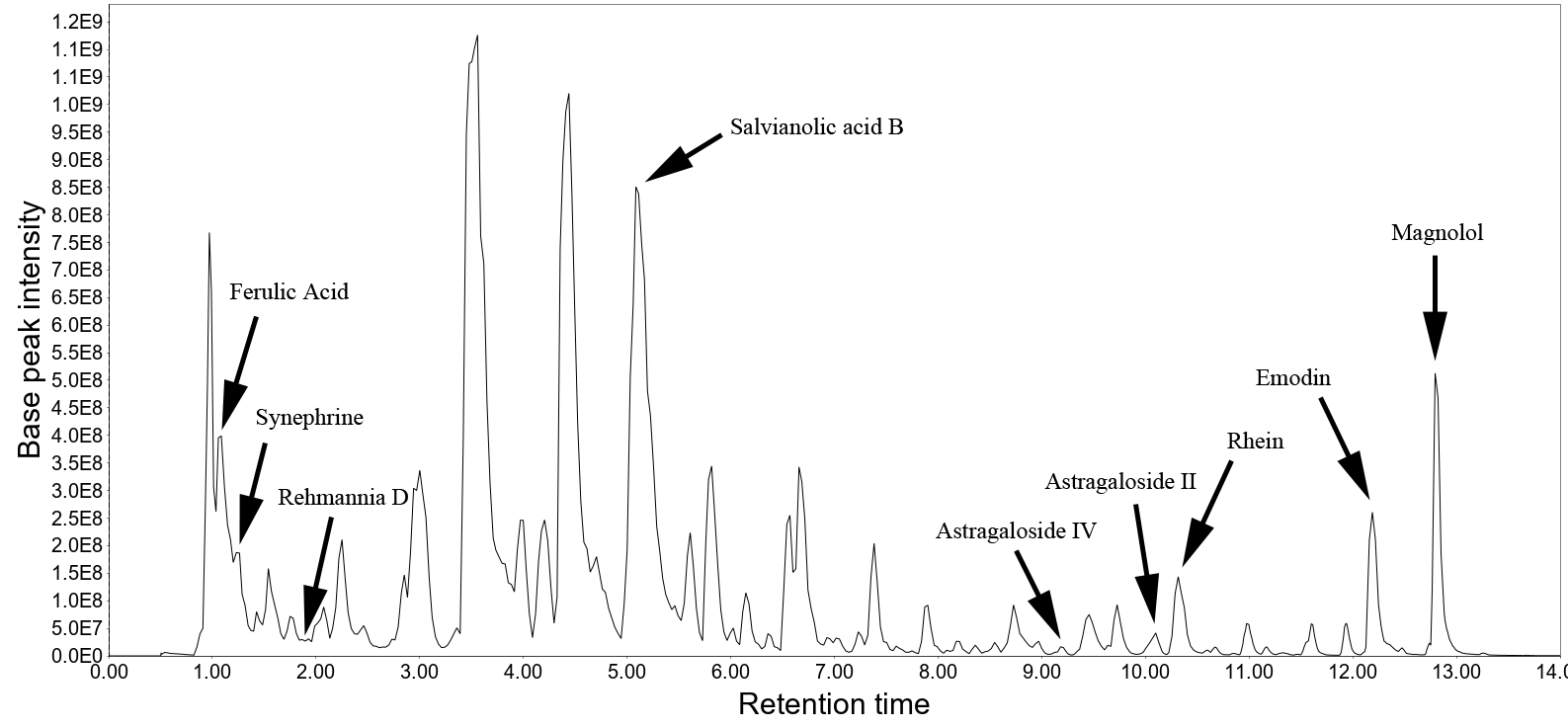

Supplement: Supplementary file 2 [file DataSheet11.ZIP › XCQ.tif]

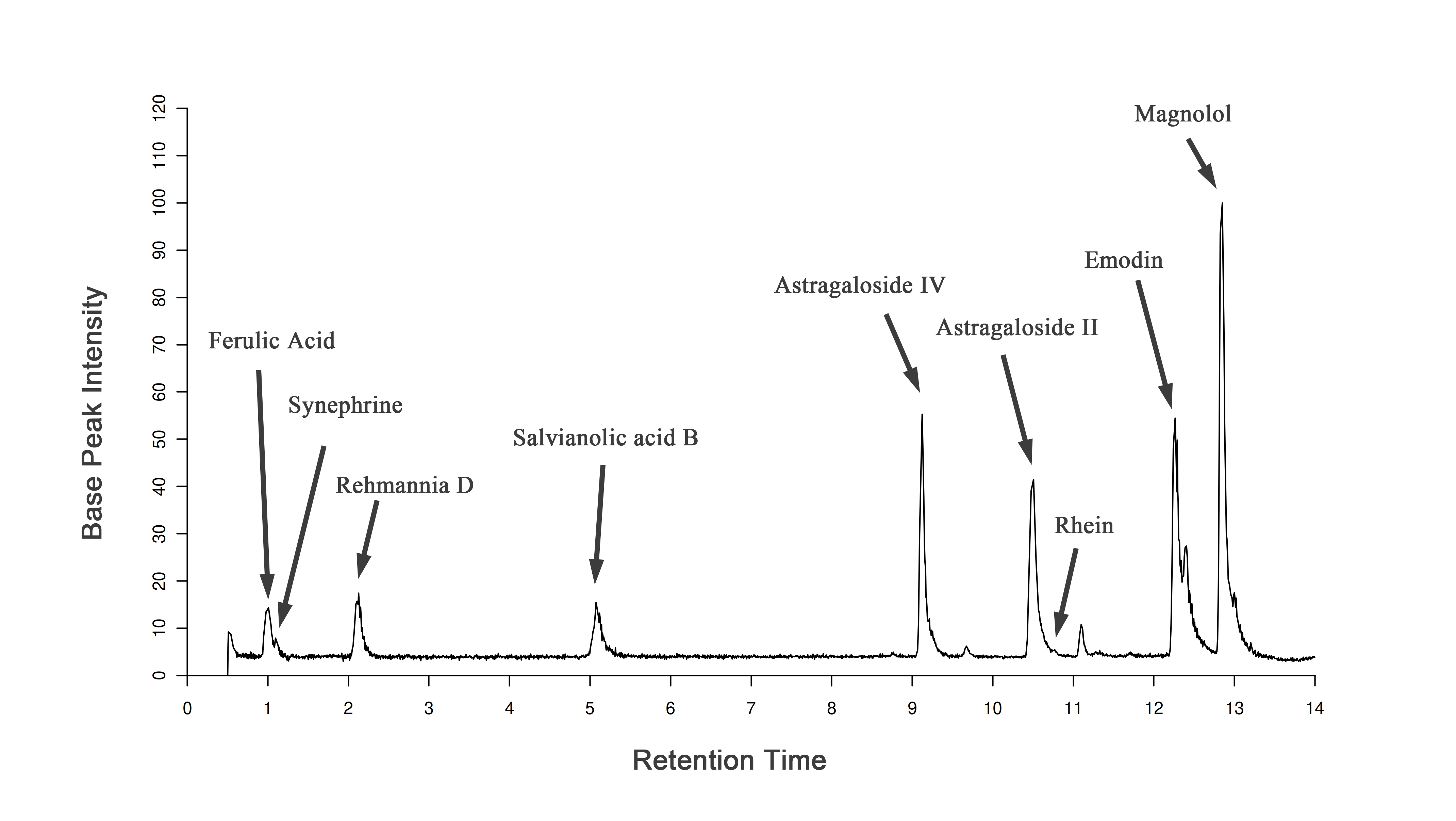

Supplement: Supplementary file 2 [file DataSheet11.ZIP › ╗∞▒Ω.jpg]

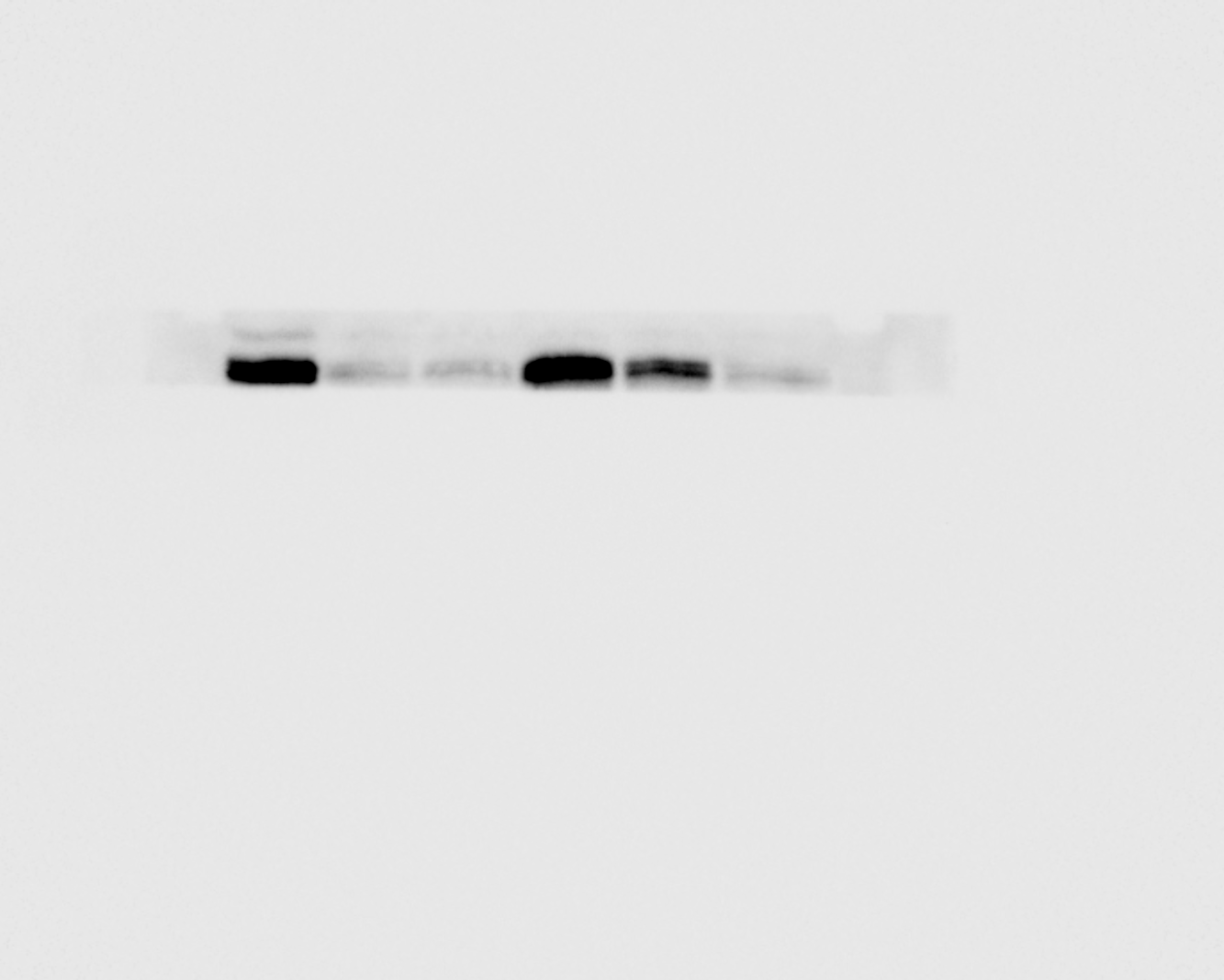

Supplement: Supplementary file 3 [file DataSheet8.ZIP › perk(Chemiluminescence).tif]

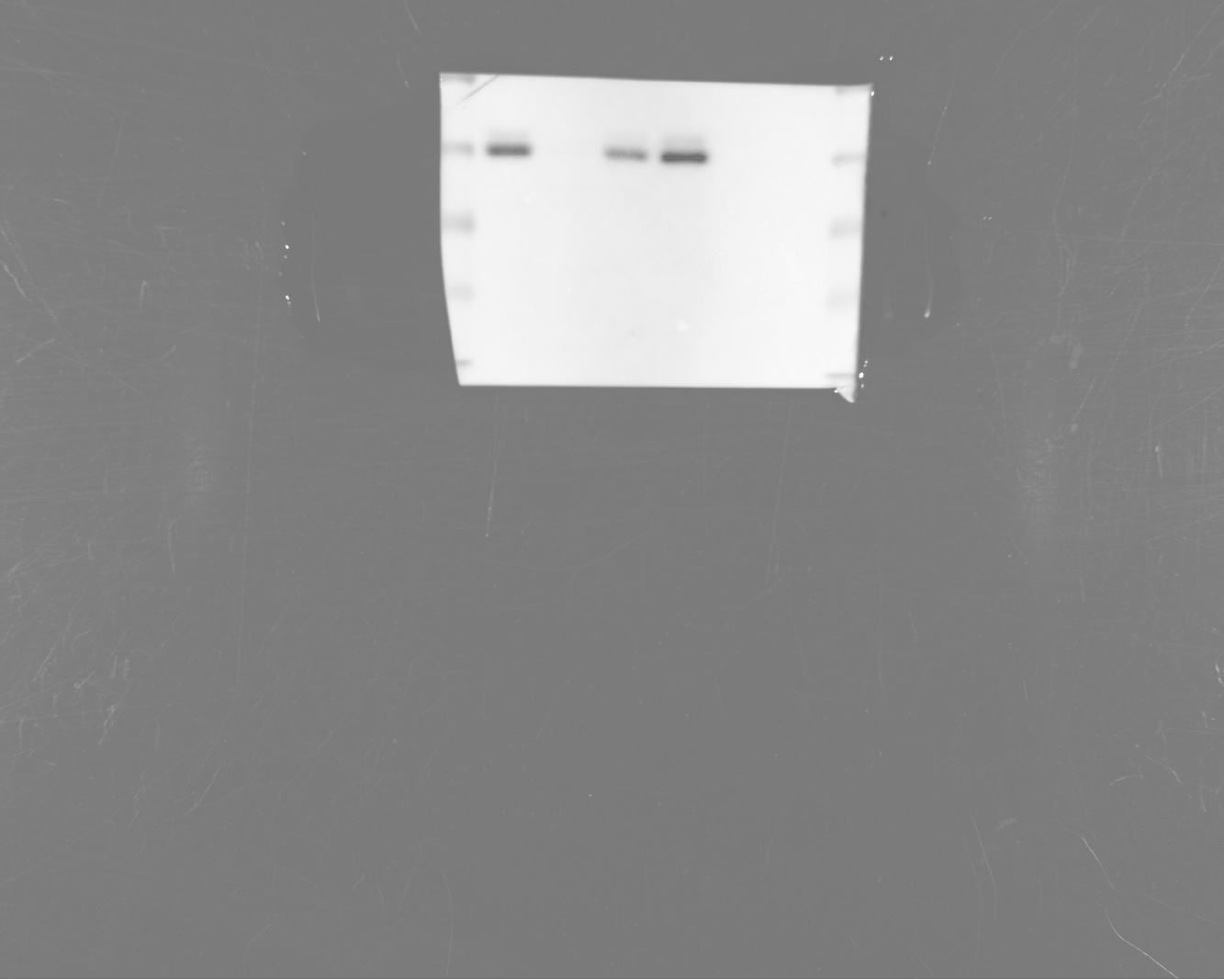

Supplement: Supplementary file 3 [file DataSheet8.ZIP › perk_3(Composite) (1).tif]

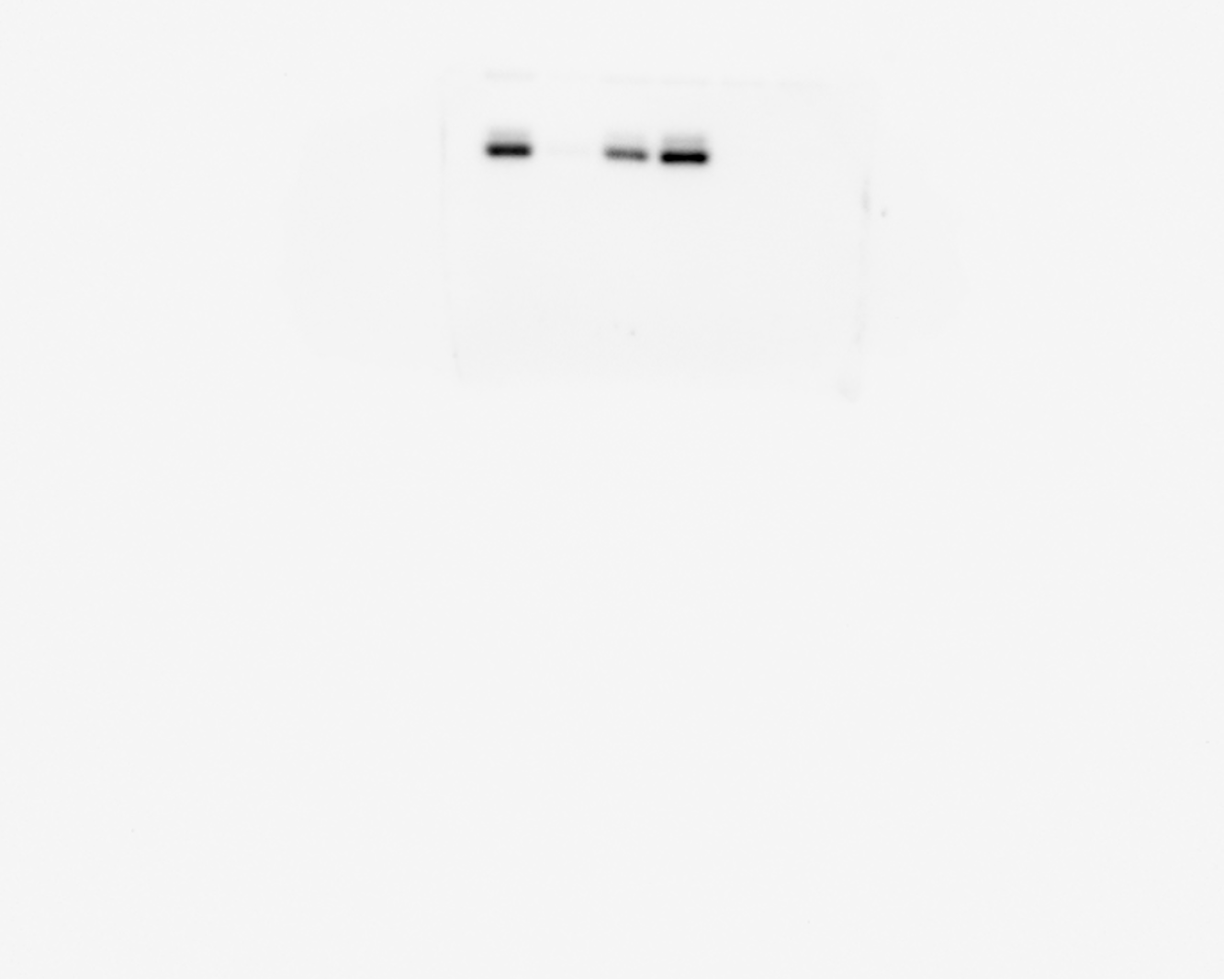

Supplement: Supplementary file 3 [file DataSheet8.ZIP › perk_3(Composite) (2).tif]

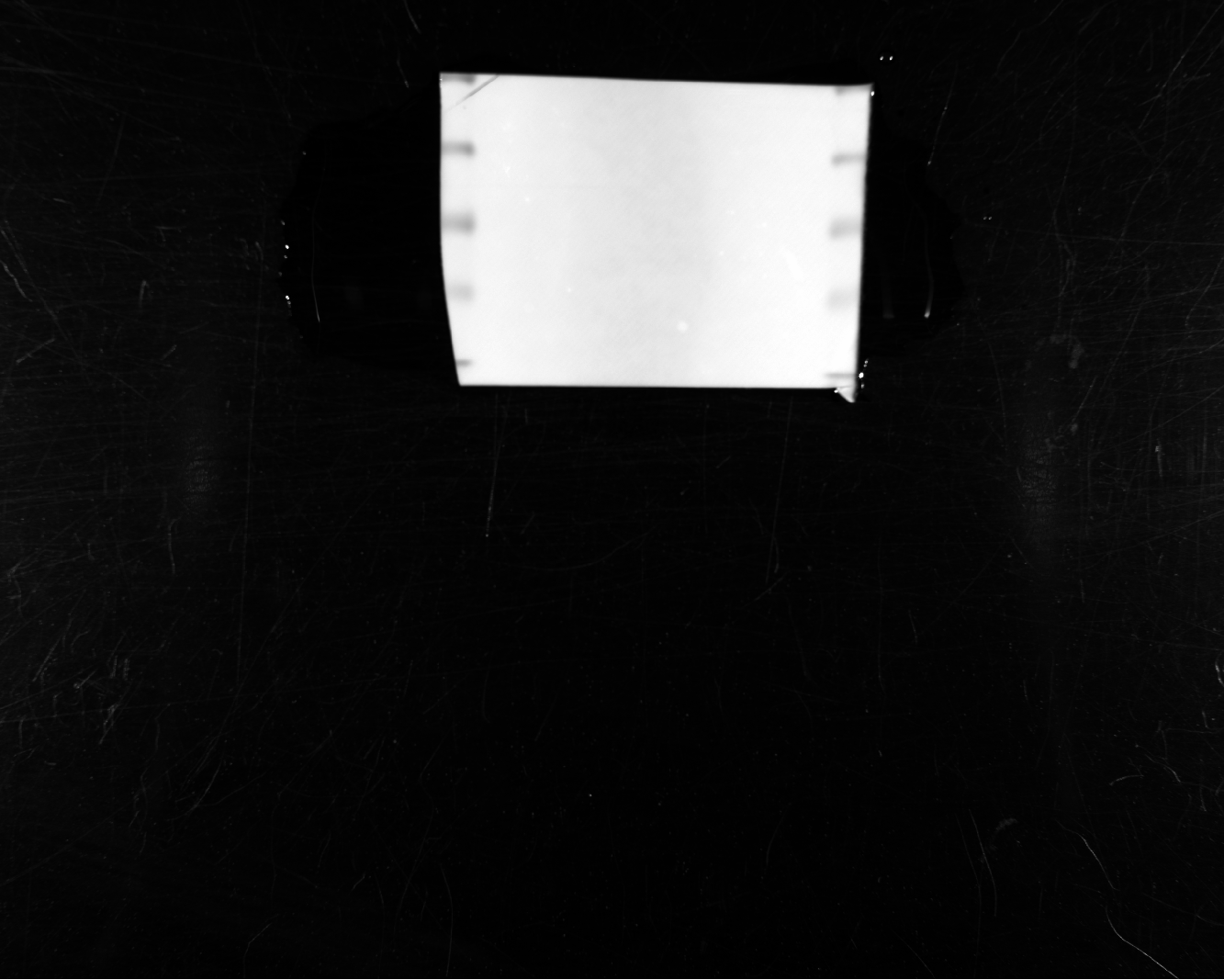

Supplement: Supplementary file 3 [file DataSheet8.ZIP › perk_3(Composite) (3).tif]

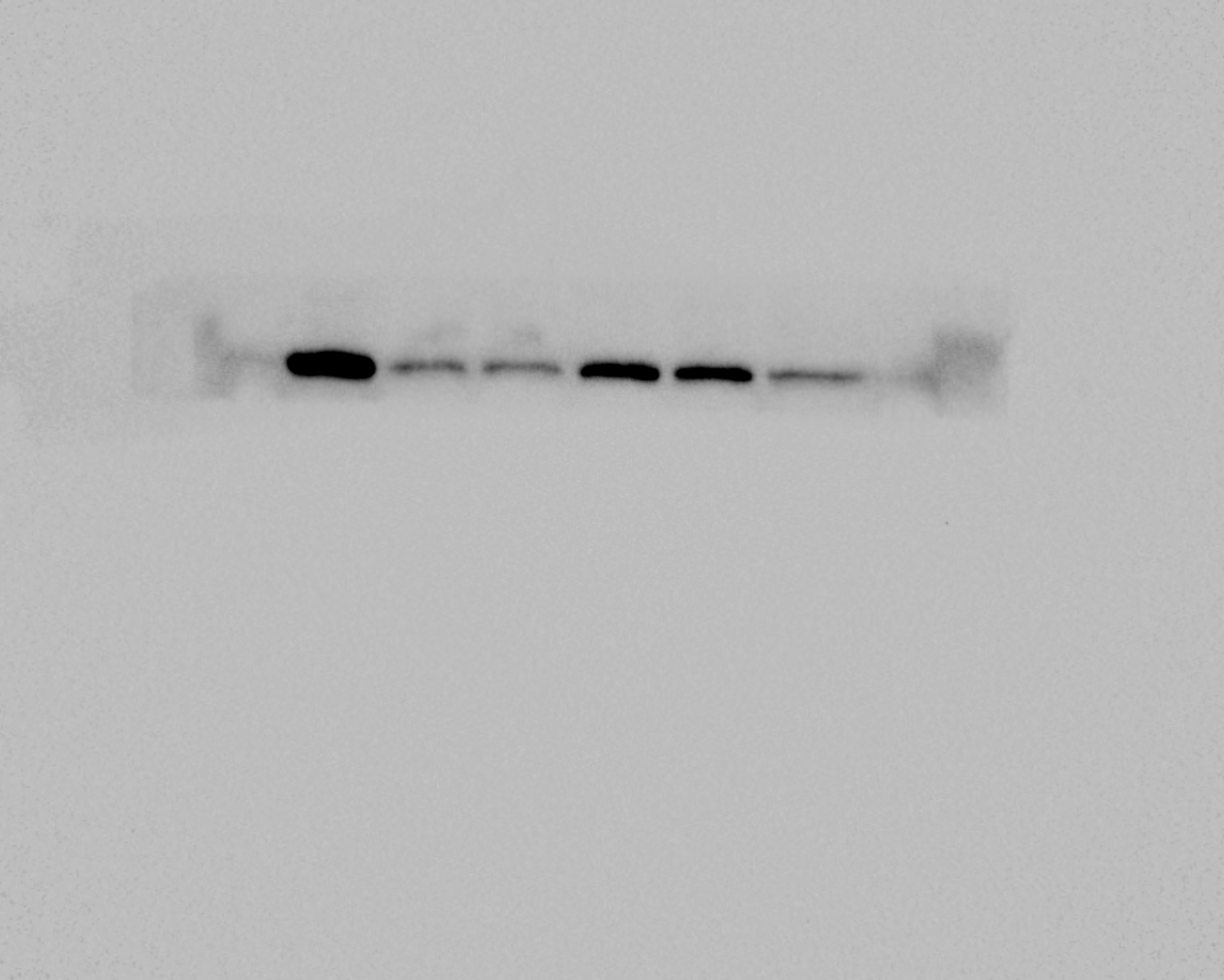

Supplement: Supplementary file 3 [file DataSheet8.ZIP › ppstat3_1(Chemiluminescence).tif]

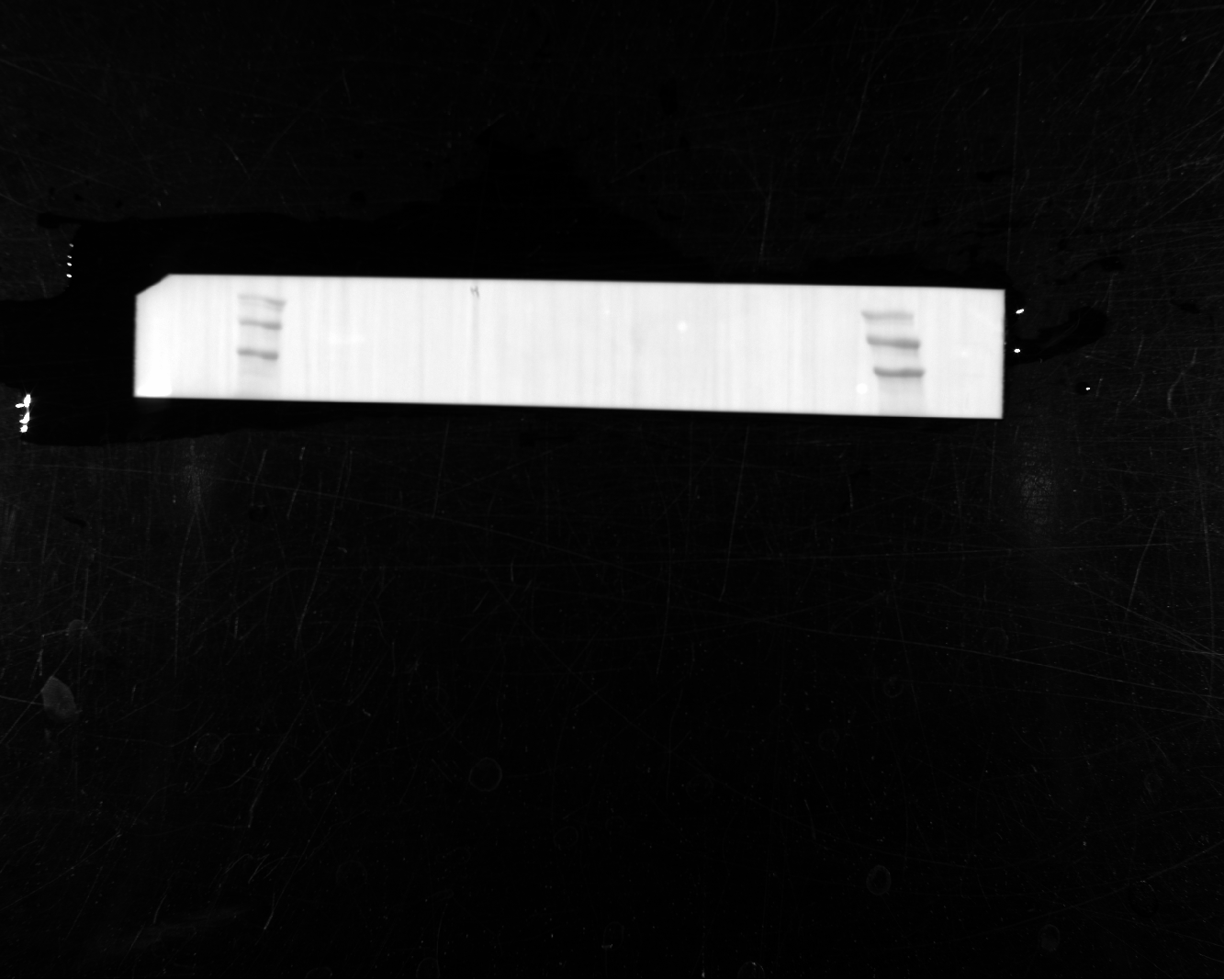

Supplement: Supplementary file 3 [file DataSheet8.ZIP › ppstat3_2(Colorimetric).tif]

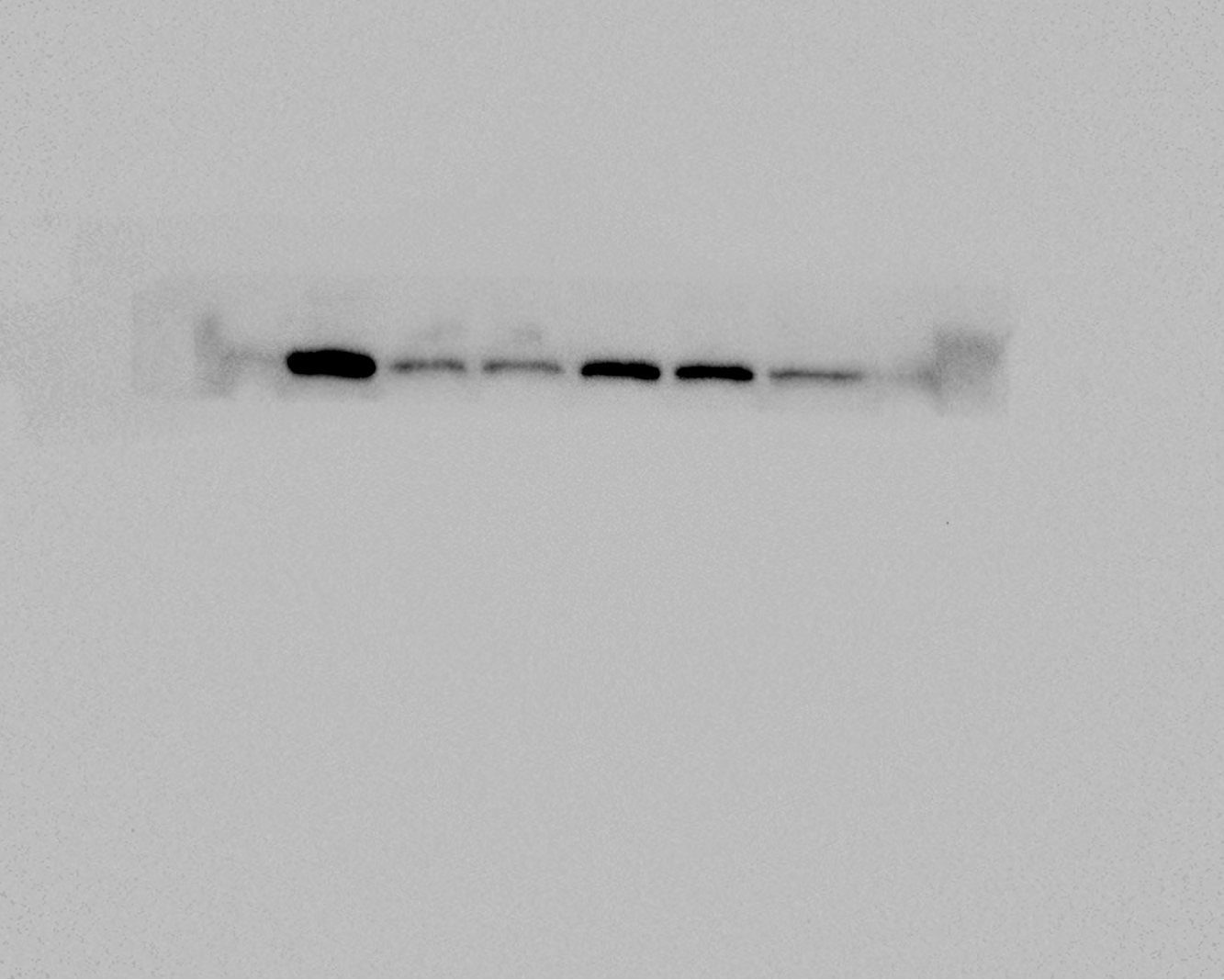

Supplement: Supplementary file 3 [file DataSheet8.ZIP › ppstat3_3(Chemiluminescence).tif]

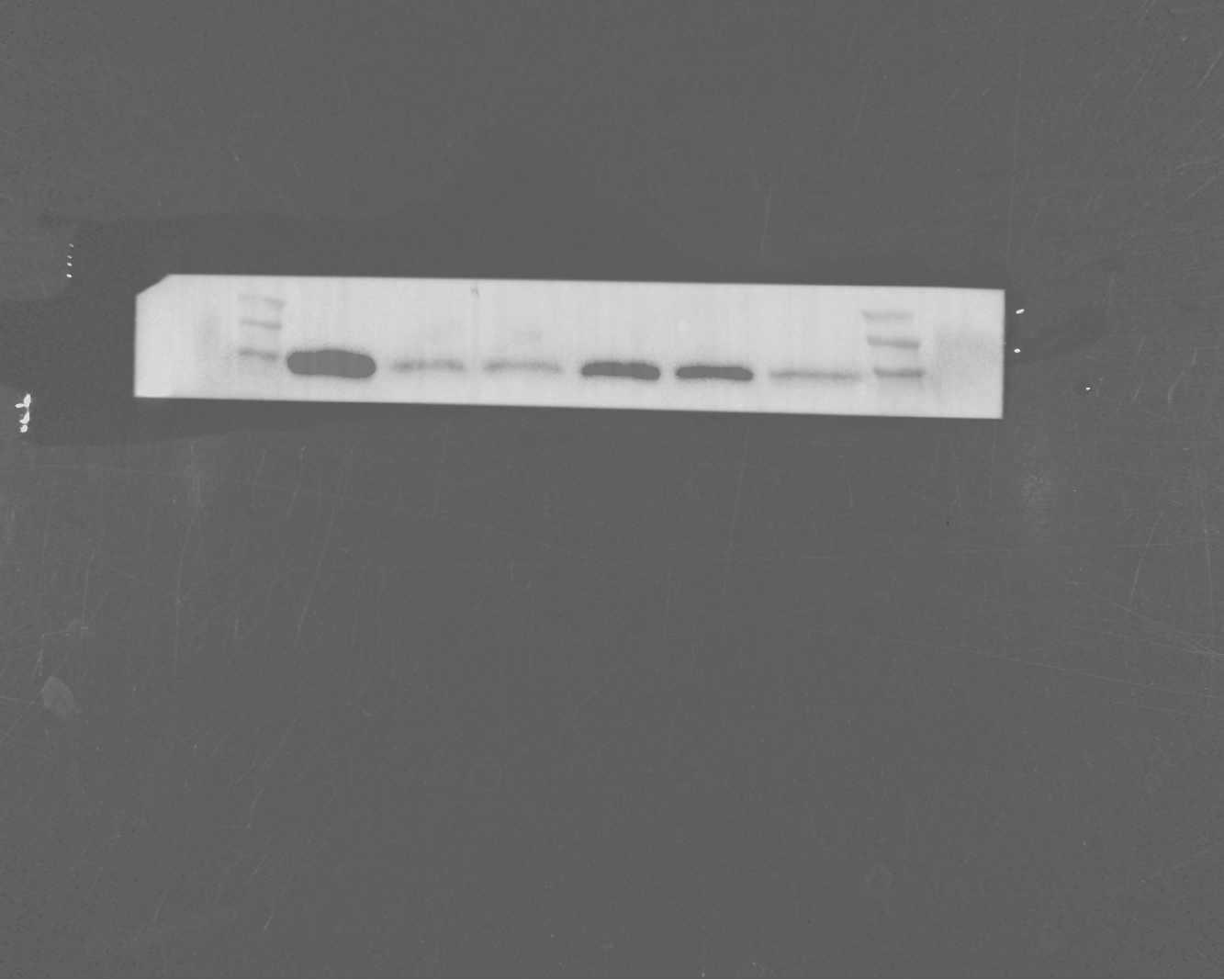

Supplement: Supplementary file 3 [file DataSheet8.ZIP › ppstat3_3(Composite).tif]

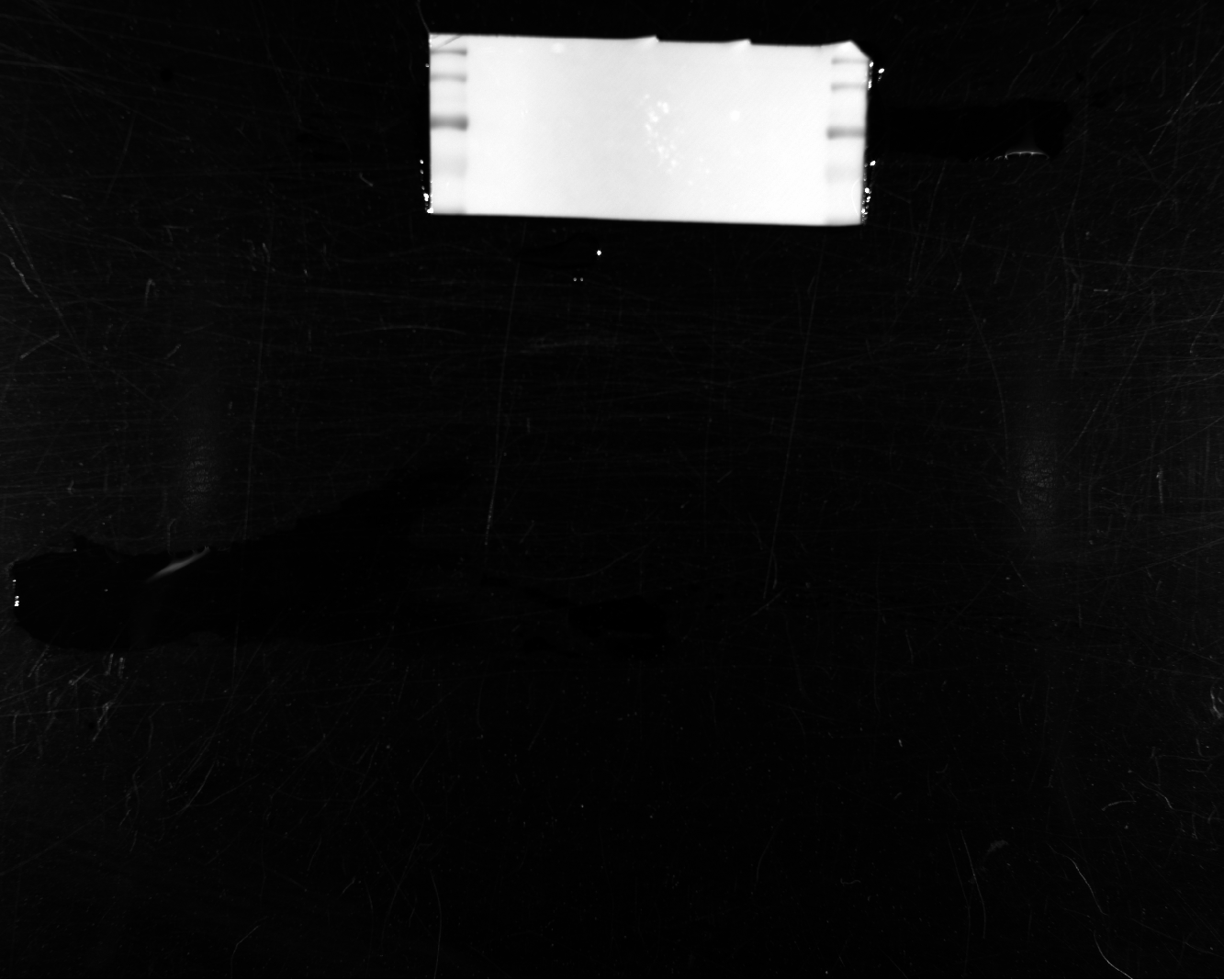

Supplement: Supplementary file 3 [file DataSheet8.ZIP › pst_4(Colorimetric) (1).tif]

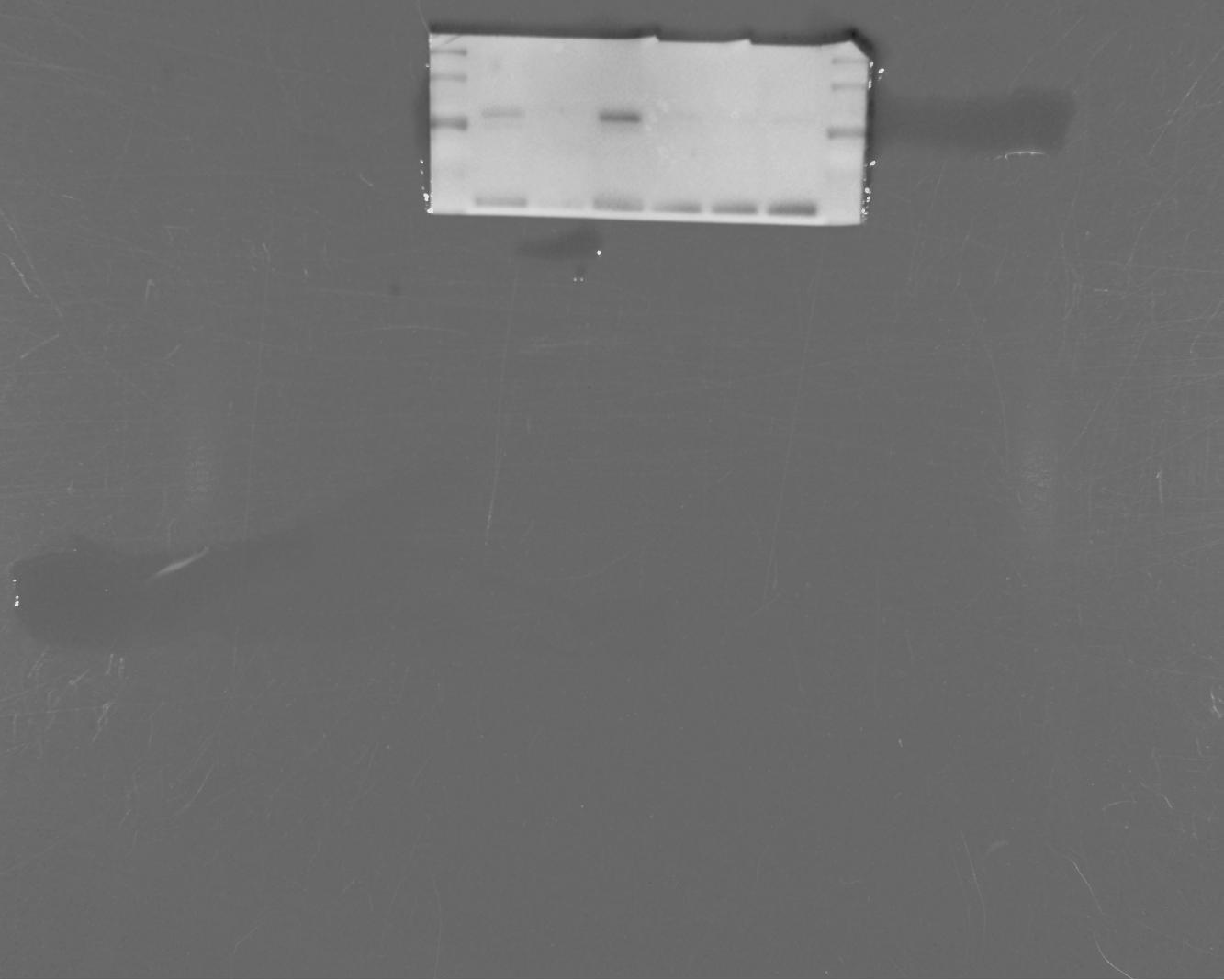

Supplement: Supplementary file 3 [file DataSheet8.ZIP › pst_4(Colorimetric) (2).tif]

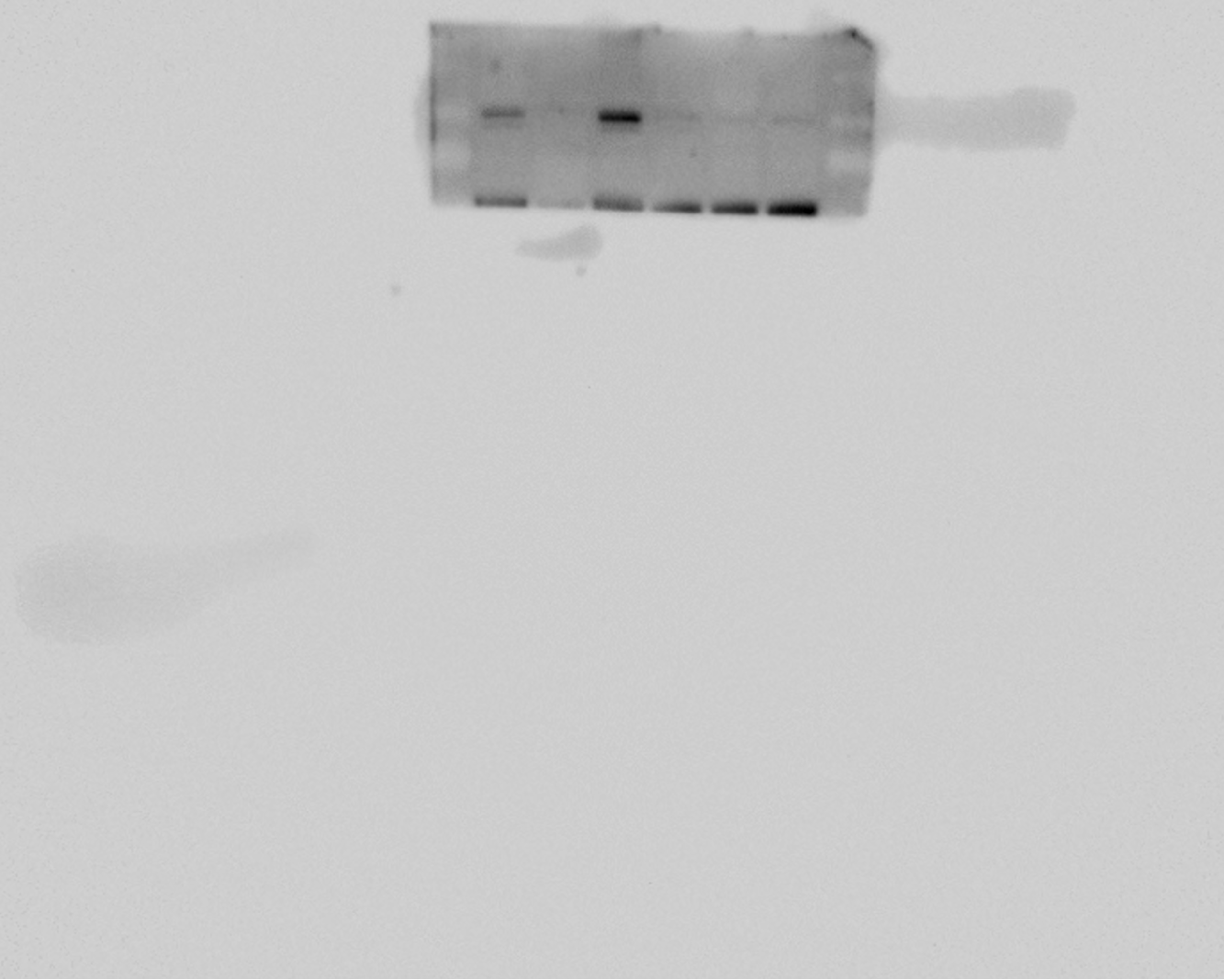

Supplement: Supplementary file 3 [file DataSheet8.ZIP › pst_4(Colorimetric) (3).tif]

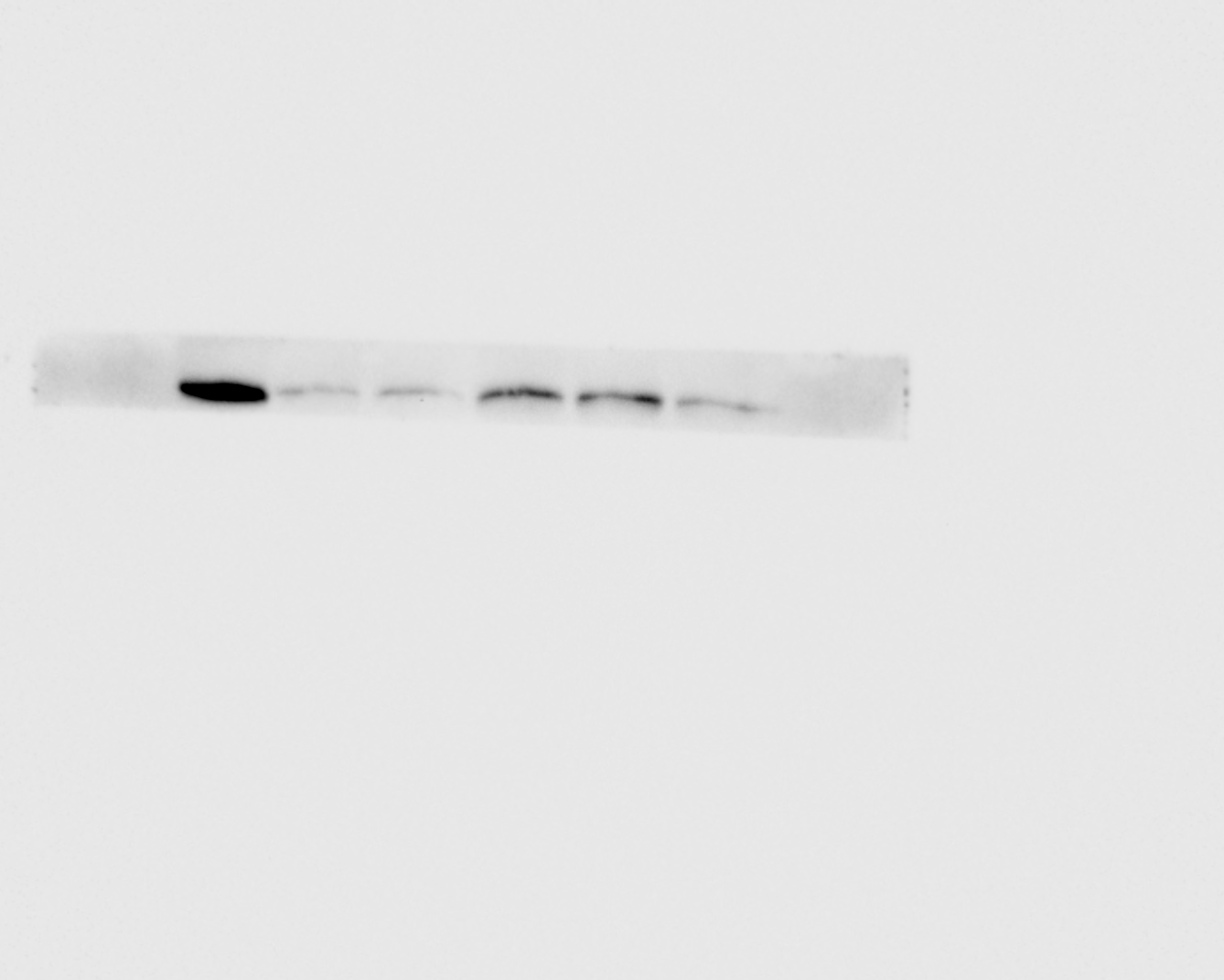

Supplement: Supplementary file 3 [file DataSheet8.ZIP › p-stat3.tif]

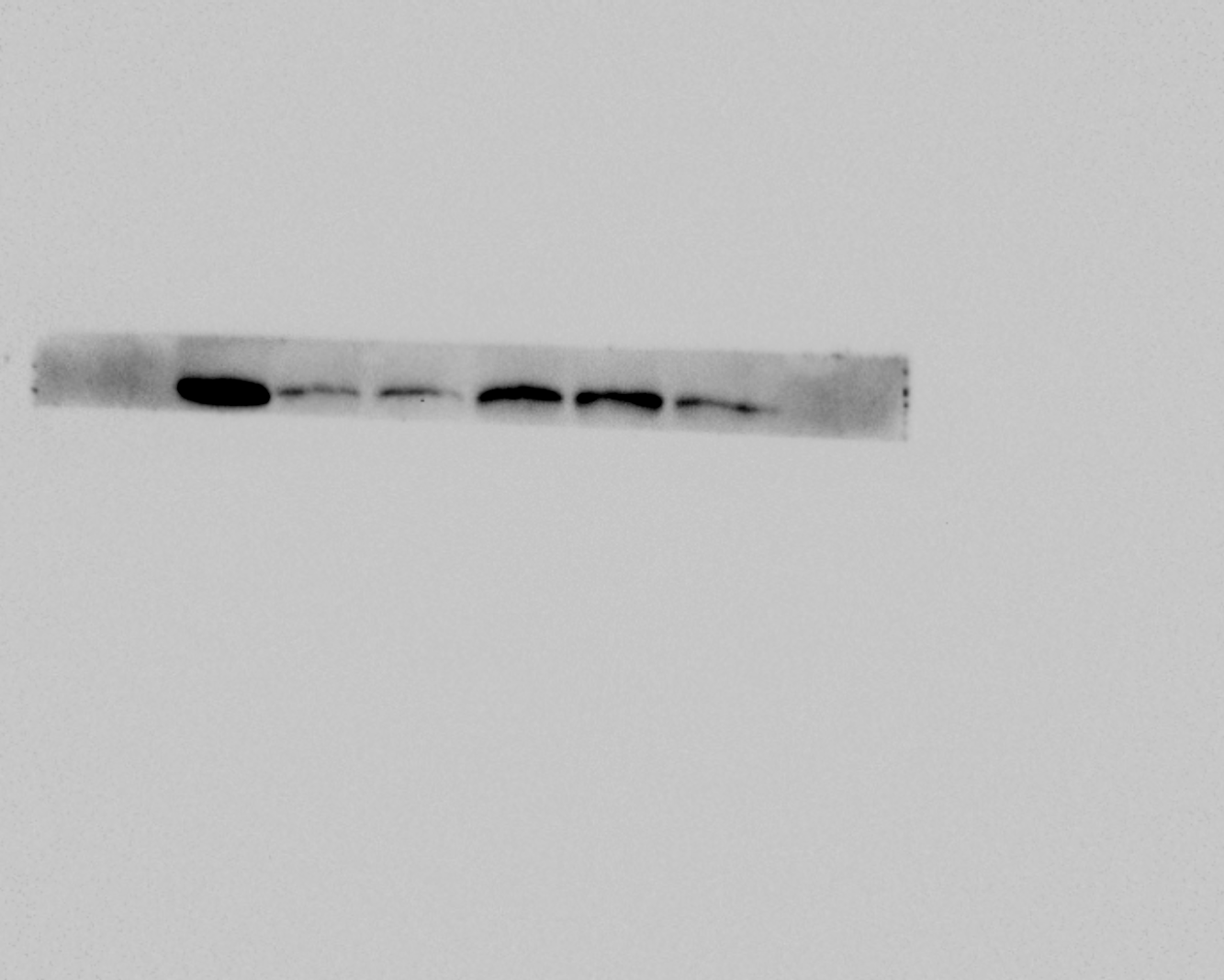

Supplement: Supplementary file 3 [file DataSheet8.ZIP › p-stat3-1.tif]

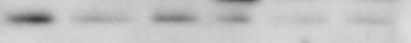

Supplement: Supplementary file 3 [file DataSheet8.ZIP › pstat3-2.tif]

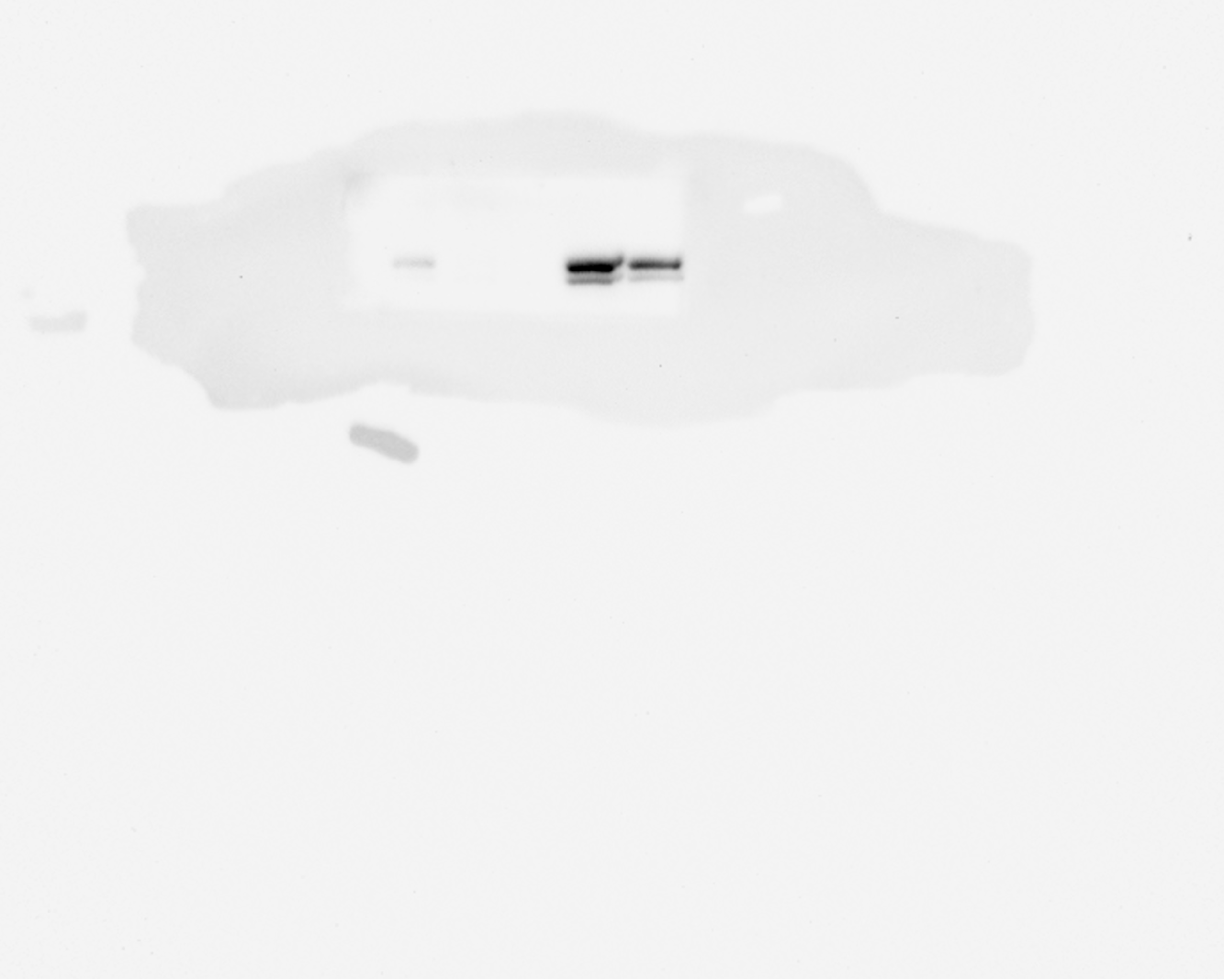

Supplement: Supplementary file 4 [file DataSheet9.ZIP › si-360-2/pst-si-2_3(Chemiluminescence).tif]

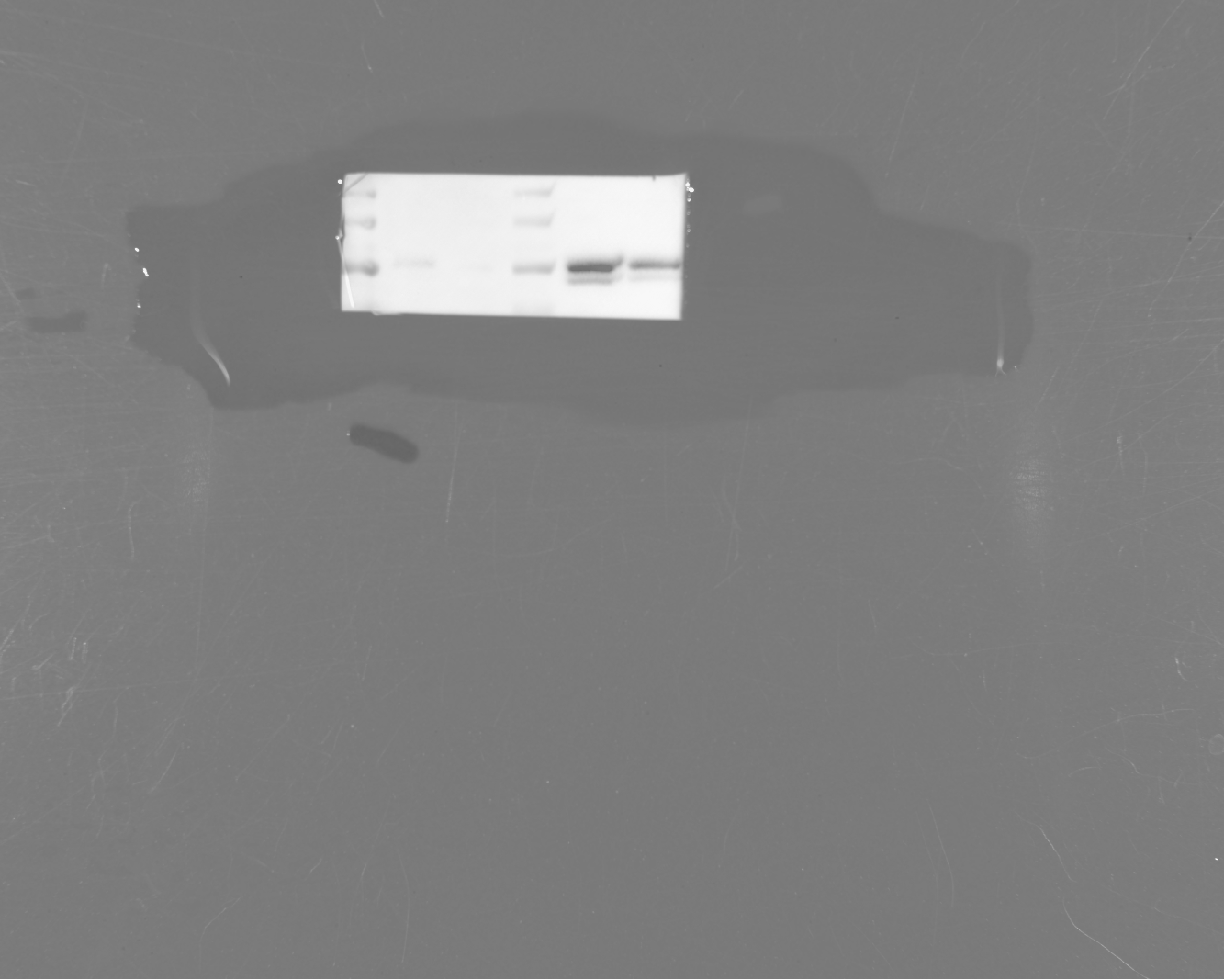

Supplement: Supplementary file 4 [file DataSheet9.ZIP › si-360-2/pst-si-2_3(Composite).tif]

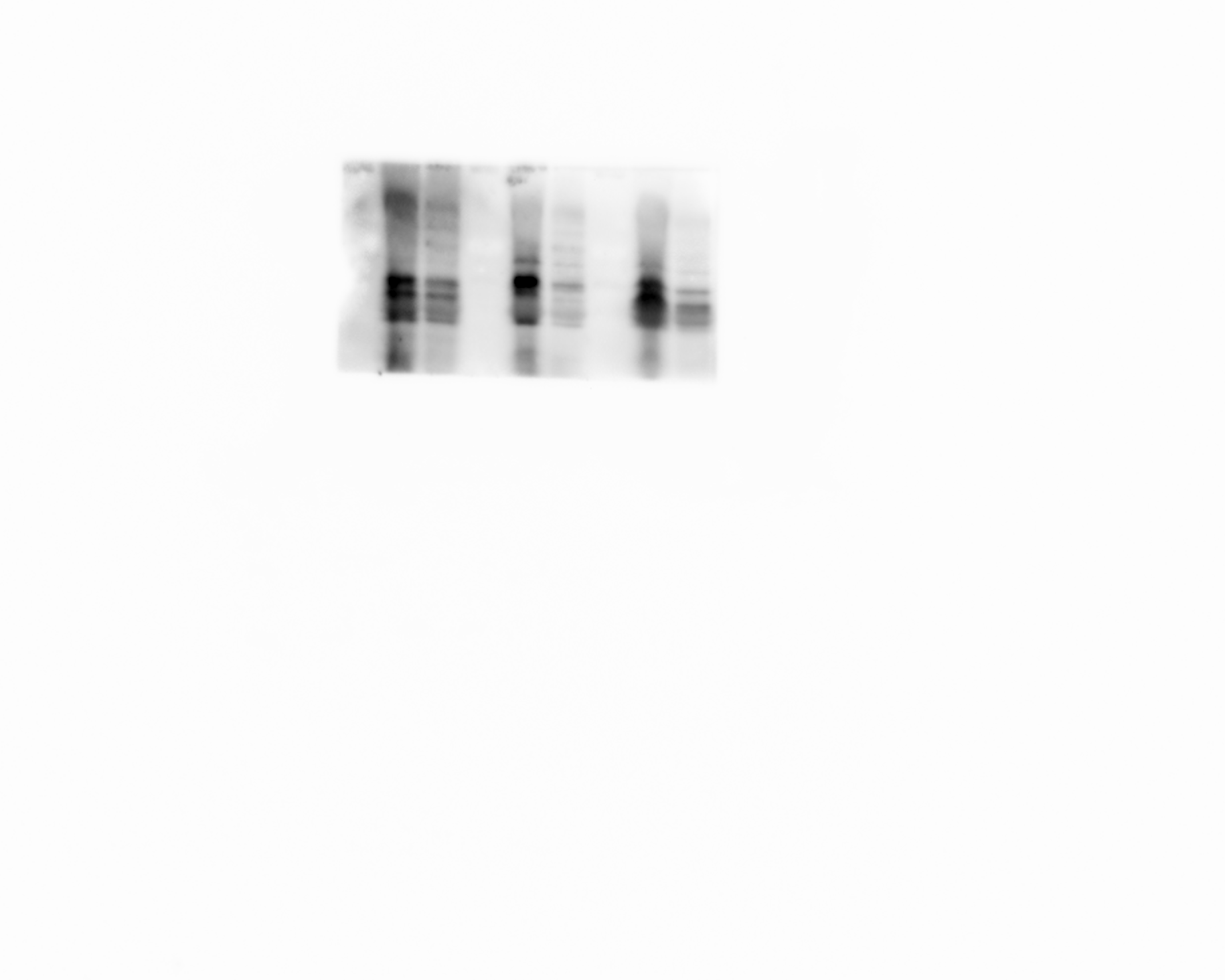

Supplement: Supplementary file 4 [file DataSheet9.ZIP › si-360-2/si-360_7(Chemiluminescence).tif]

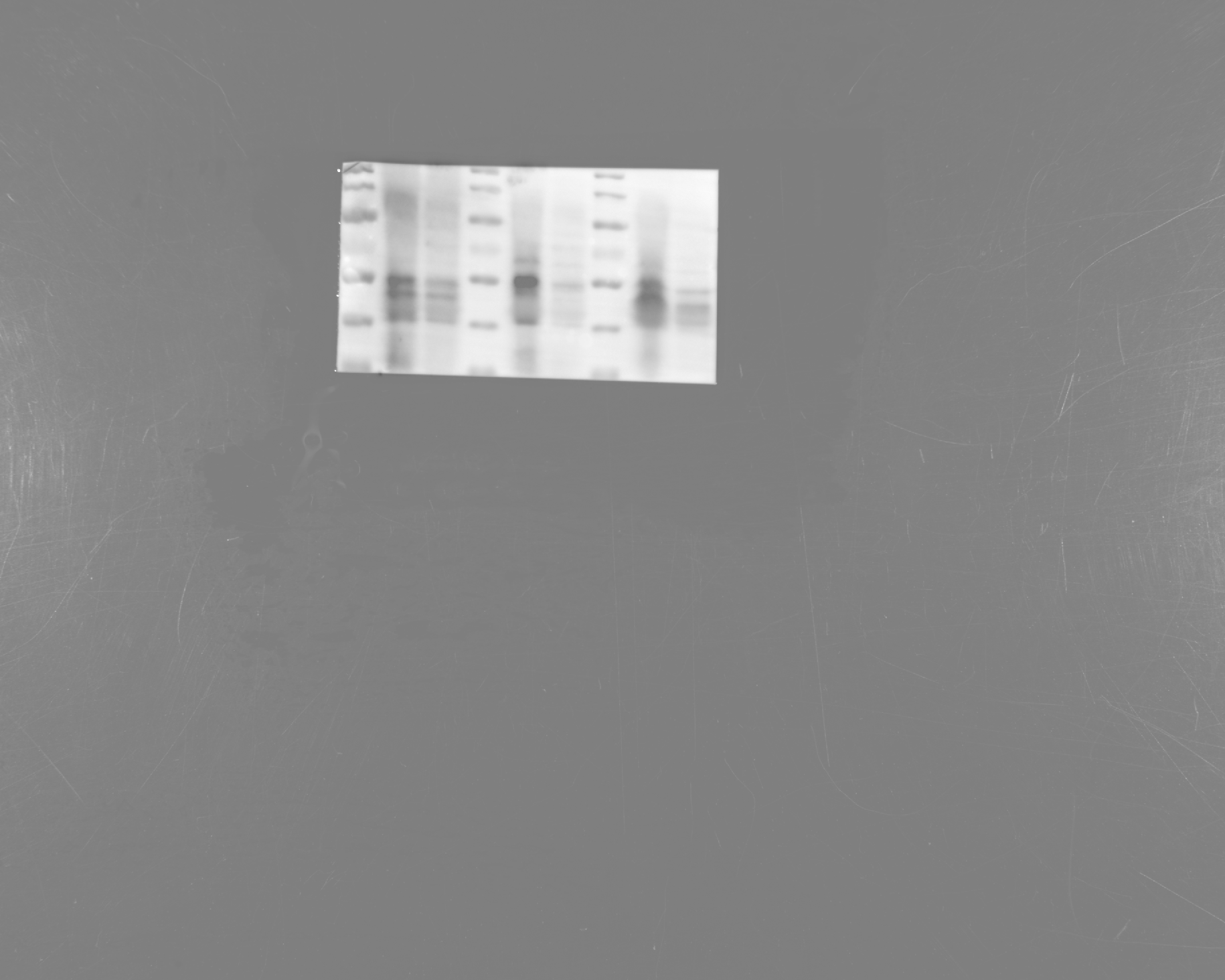

Supplement: Supplementary file 4 [file DataSheet9.ZIP › si-360-2/si-360_7(Composite).tif]

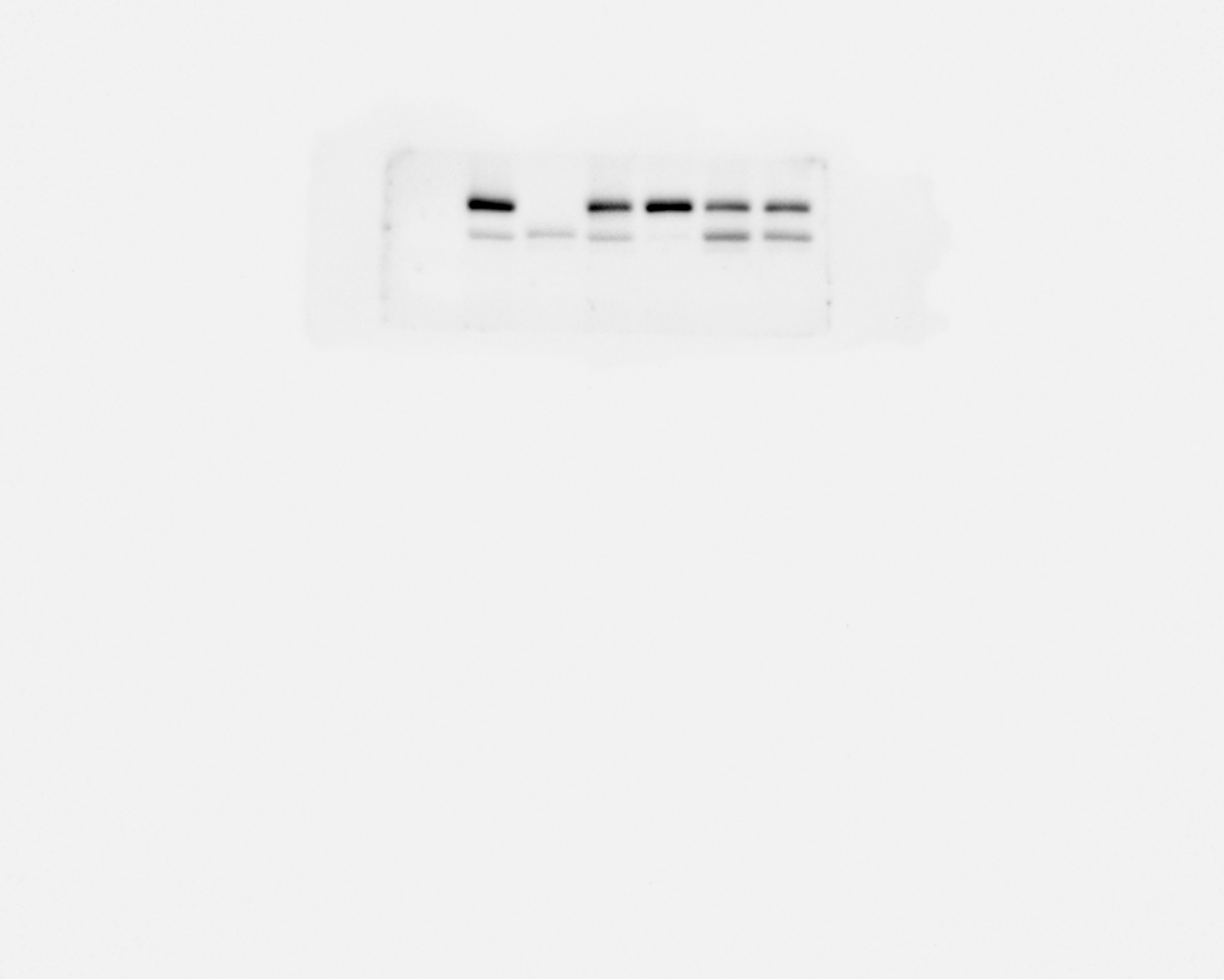

Supplement: Supplementary file 4 [file DataSheet9.ZIP › si-360-2/si-parp_3(Chemiluminescence).tif]

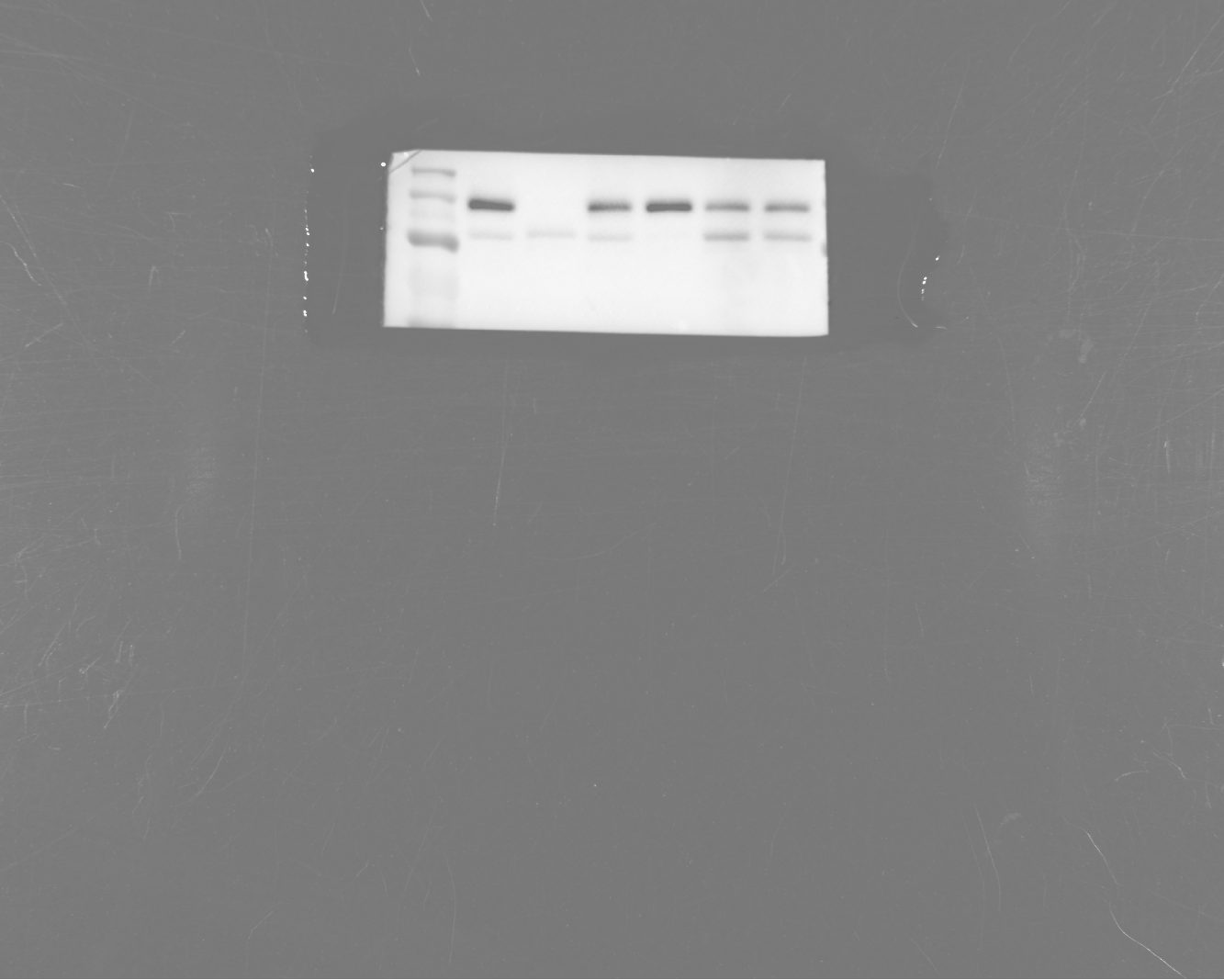

Supplement: Supplementary file 4 [file DataSheet9.ZIP › si-360-2/si-parp_3(Composite).tif]

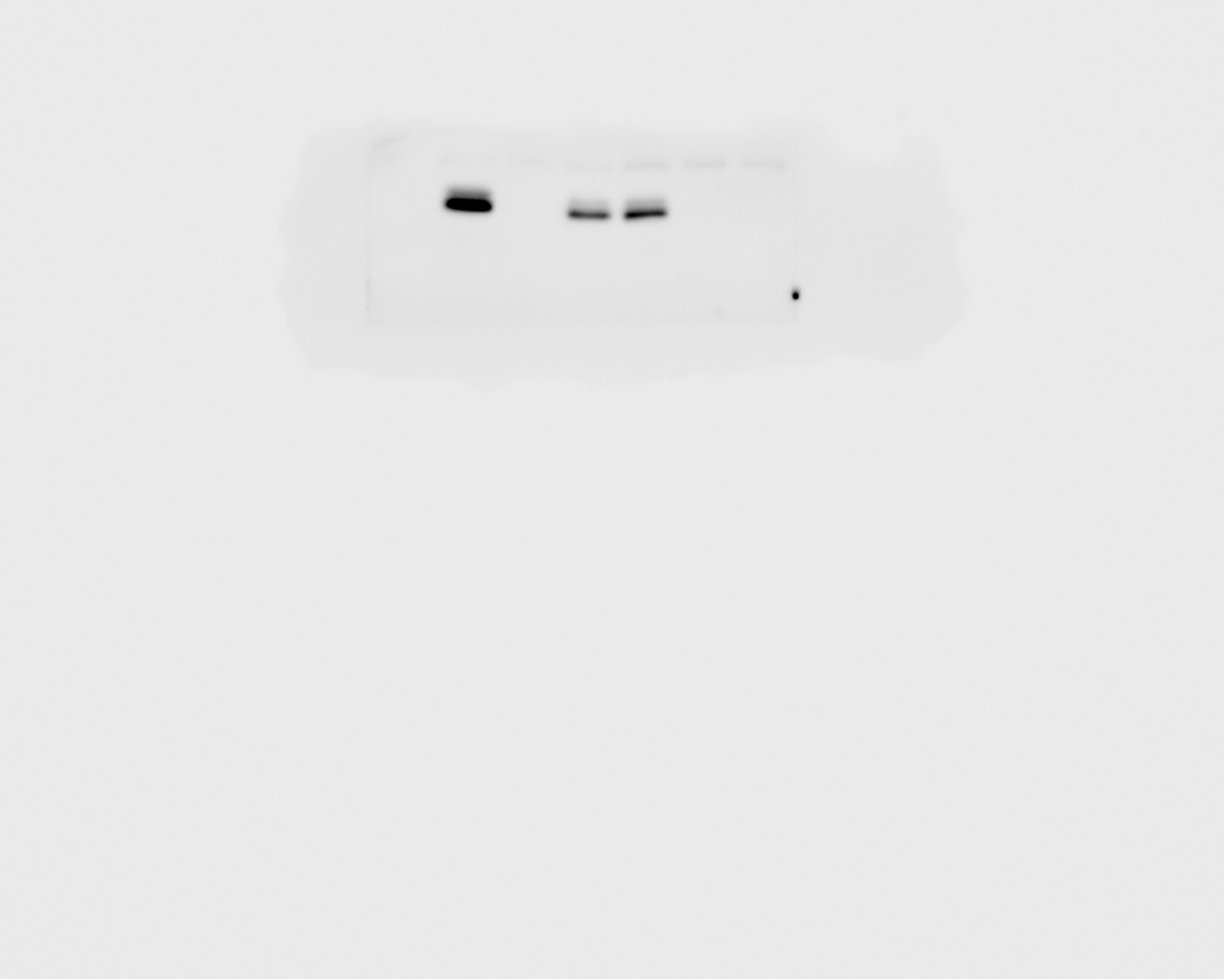

Supplement: Supplementary file 4 [file DataSheet9.ZIP › si-360-2/si-perk_4(Chemiluminescence).tif]

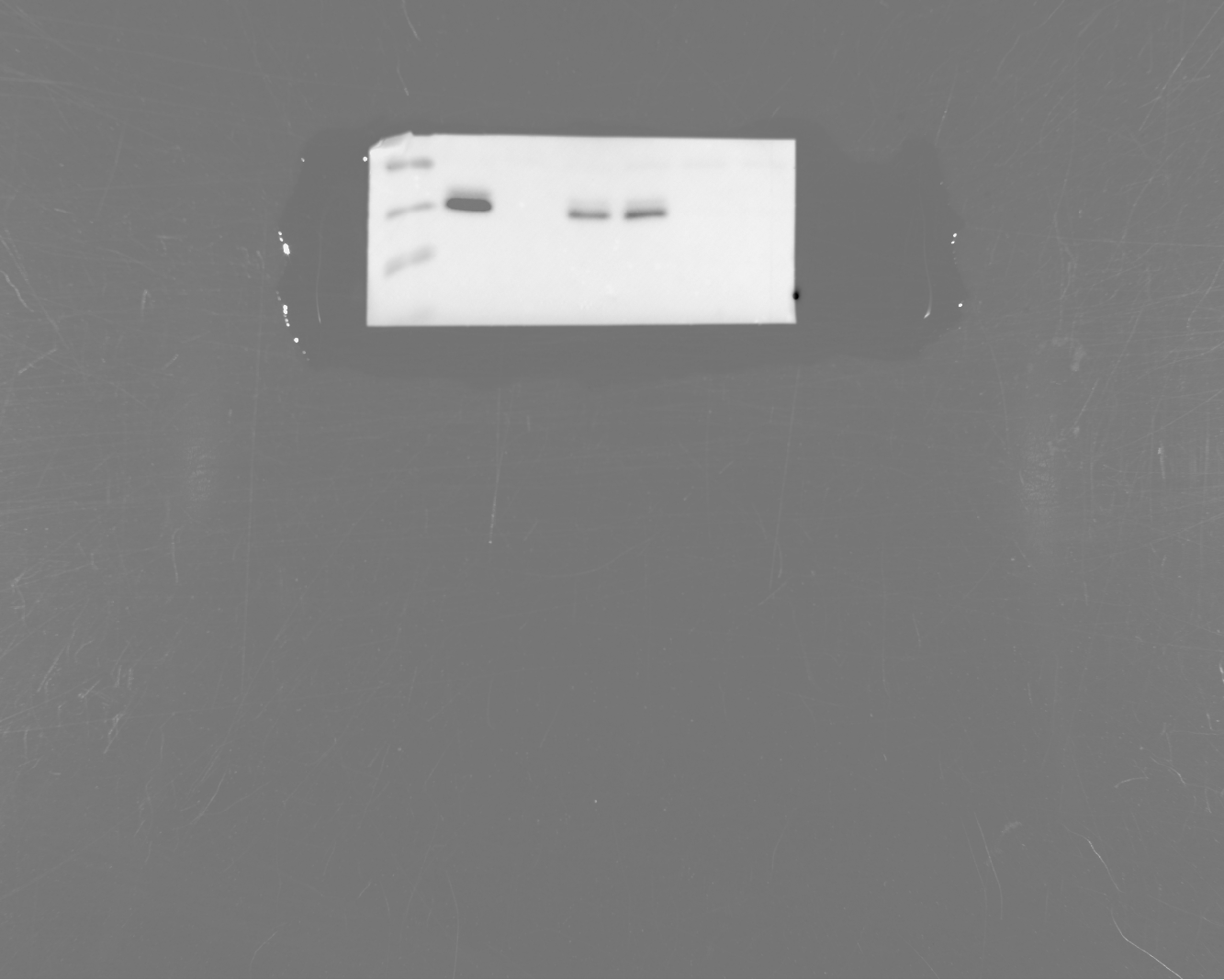

Supplement: Supplementary file 4 [file DataSheet9.ZIP › si-360-2/si-perk_4(Composite).tif]

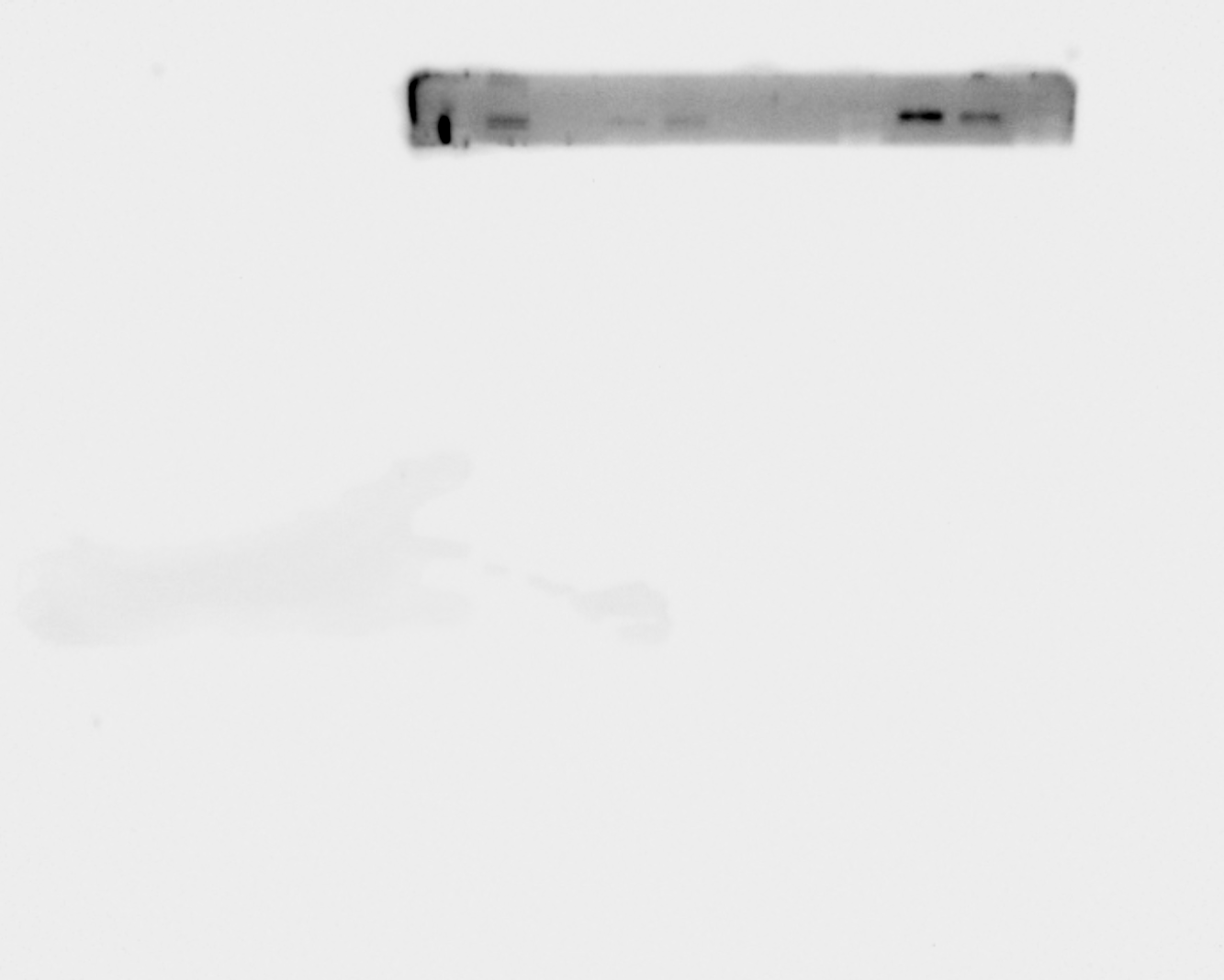

Supplement: Supplementary file 4 [file DataSheet9.ZIP › si-360-2/si-pst-ncsi_2(Chemiluminescence).tif]

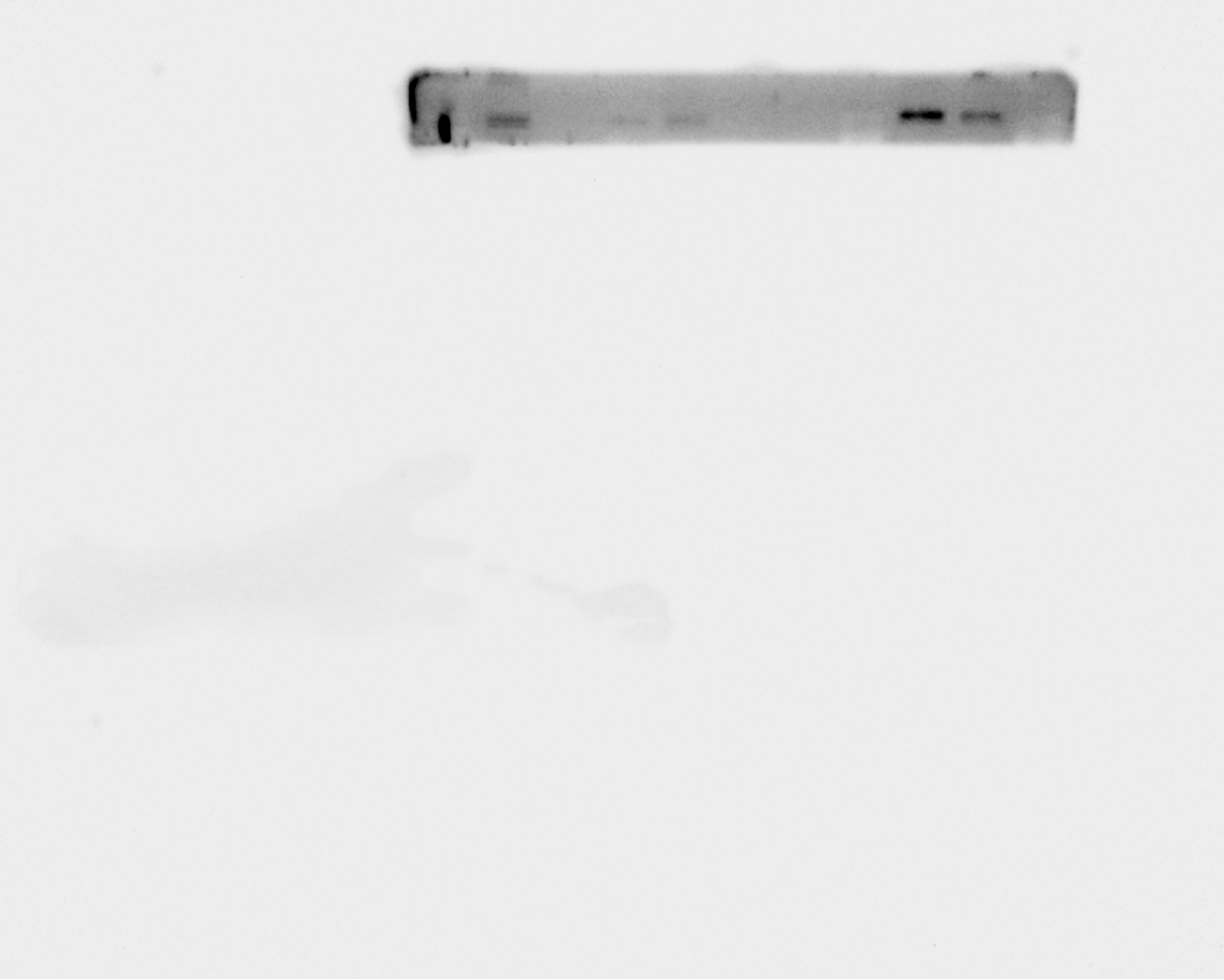

Supplement: Supplementary file 4 [file DataSheet9.ZIP › si-360-2/si-pst-ncsi_4(Chemiluminescence).tif]

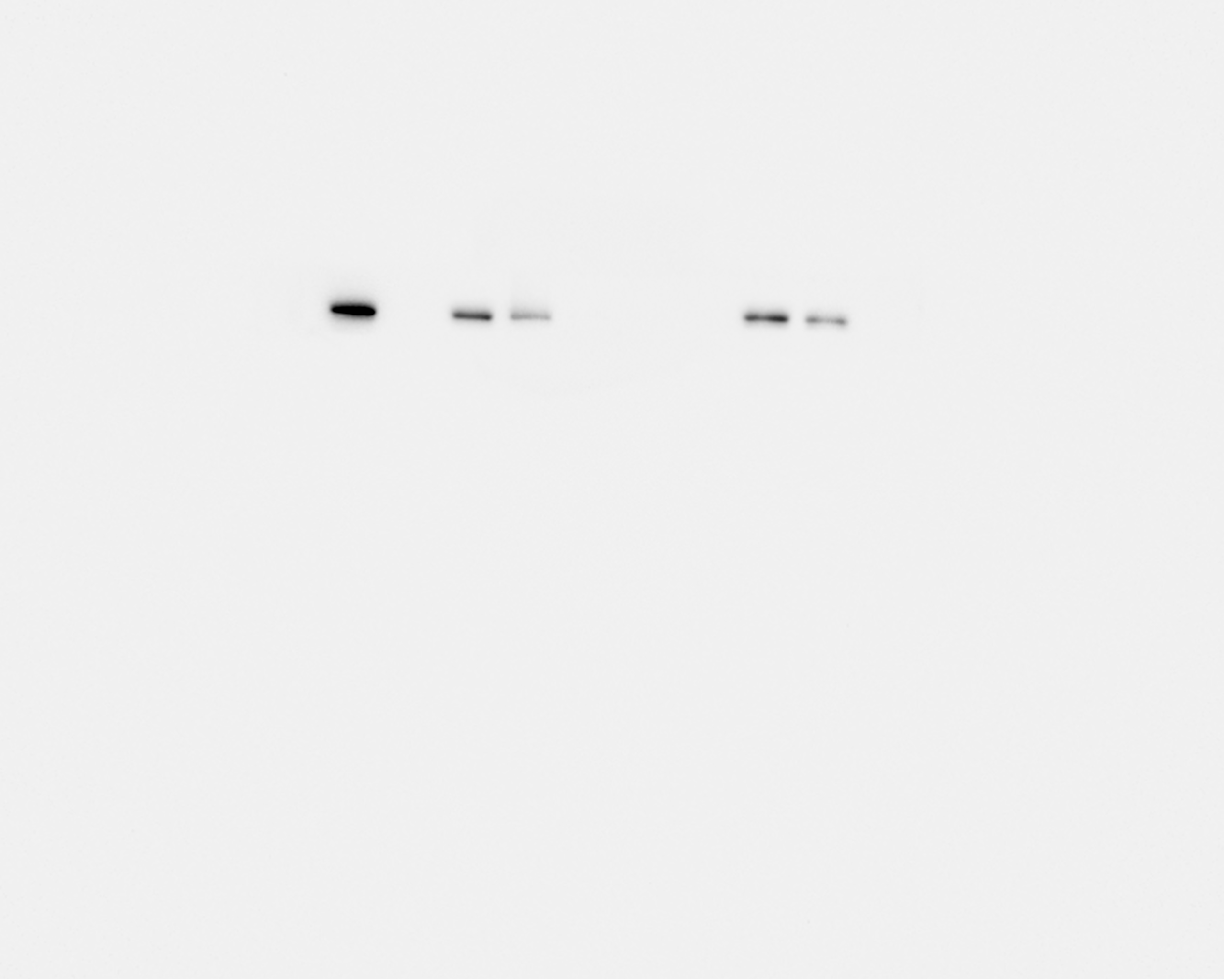

Supplement: Supplementary file 4 [file DataSheet9.ZIP › si-360-2/si-st_3(Chemiluminescence).tif]

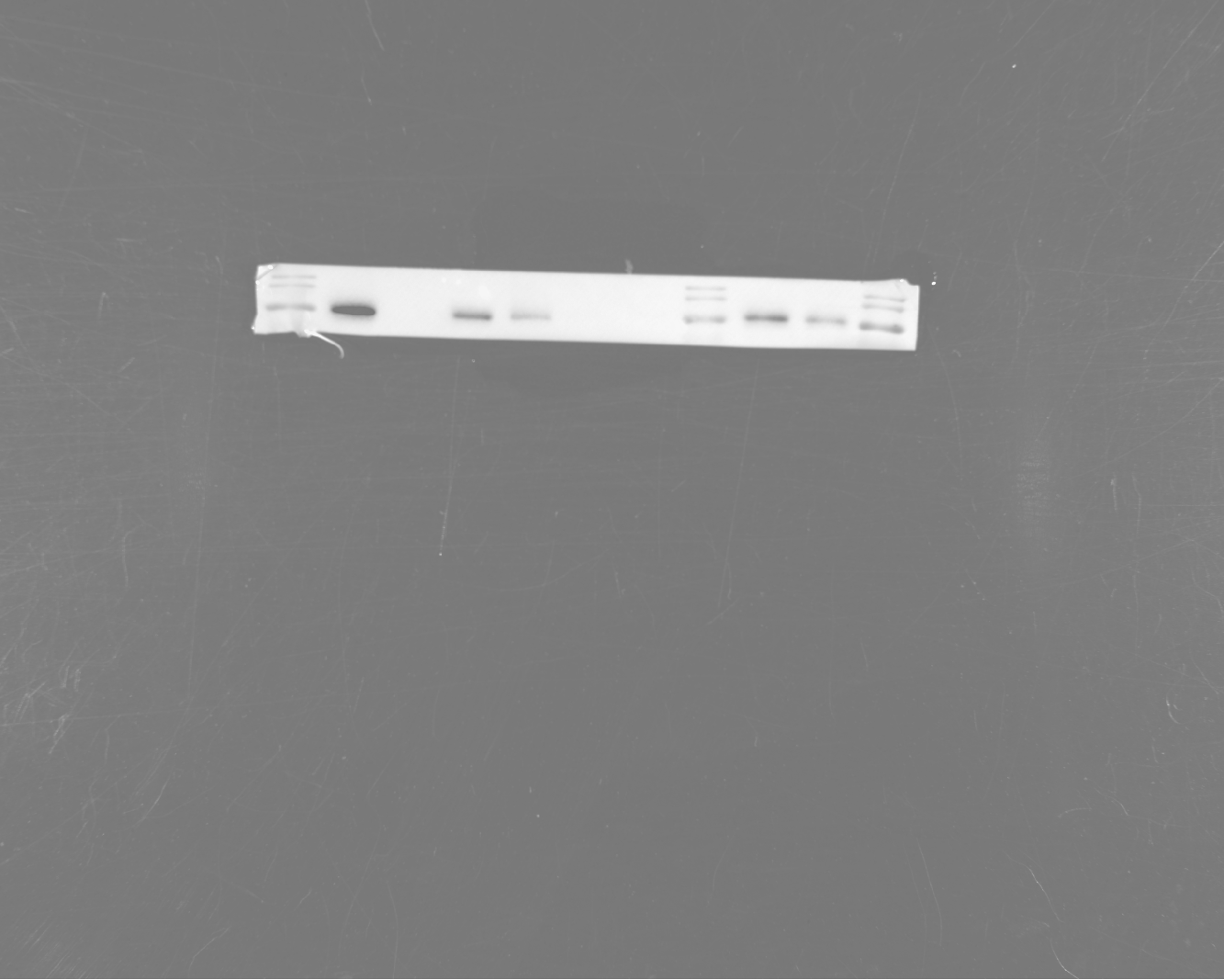

Supplement: Supplementary file 4 [file DataSheet9.ZIP › si-360-2/si-st_3(Composite).tif]

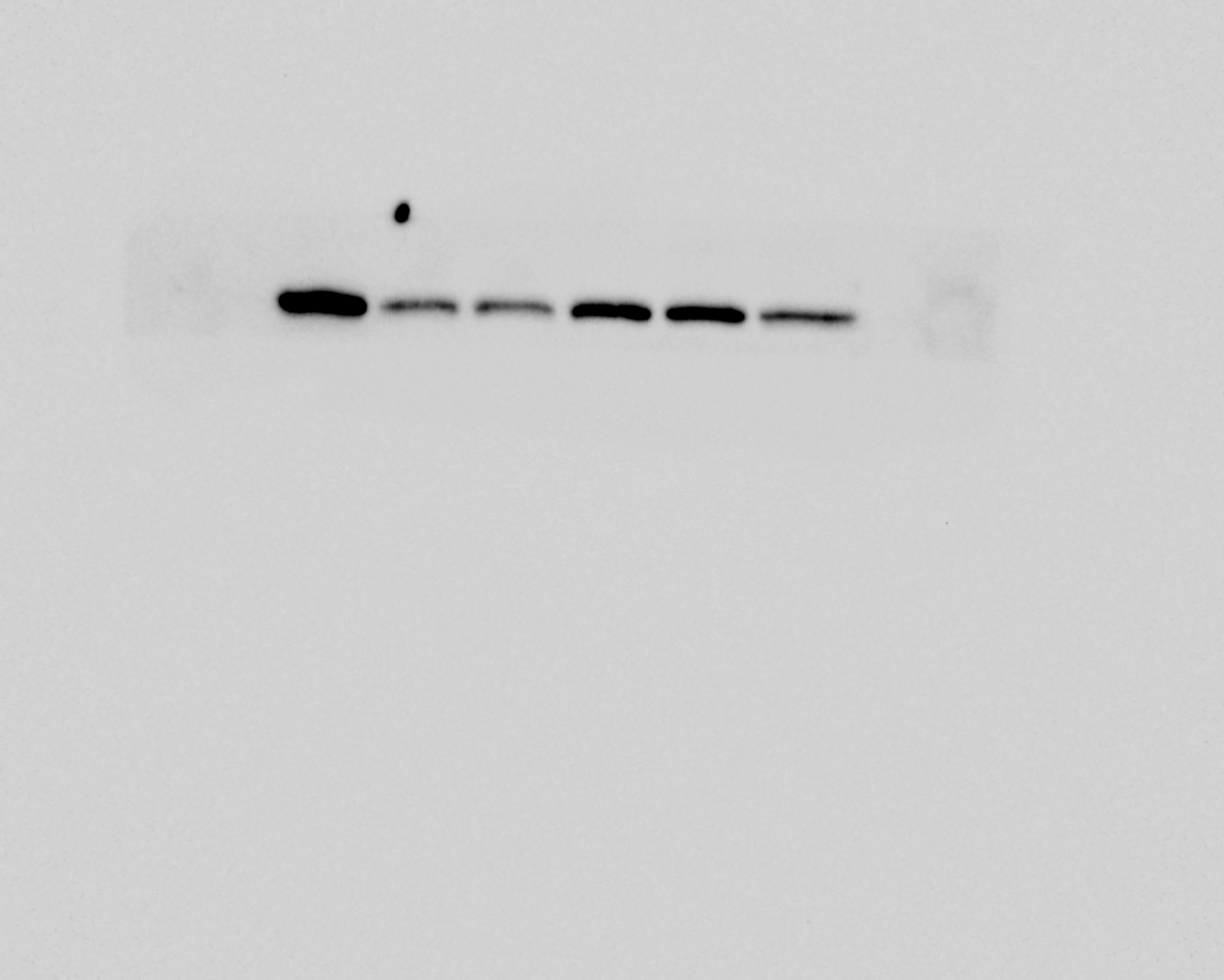

Supplement: Supplementary file 4 [file DataSheet9.ZIP › si-360-2/stat3ncsi_1(Chemiluminescence).tif]

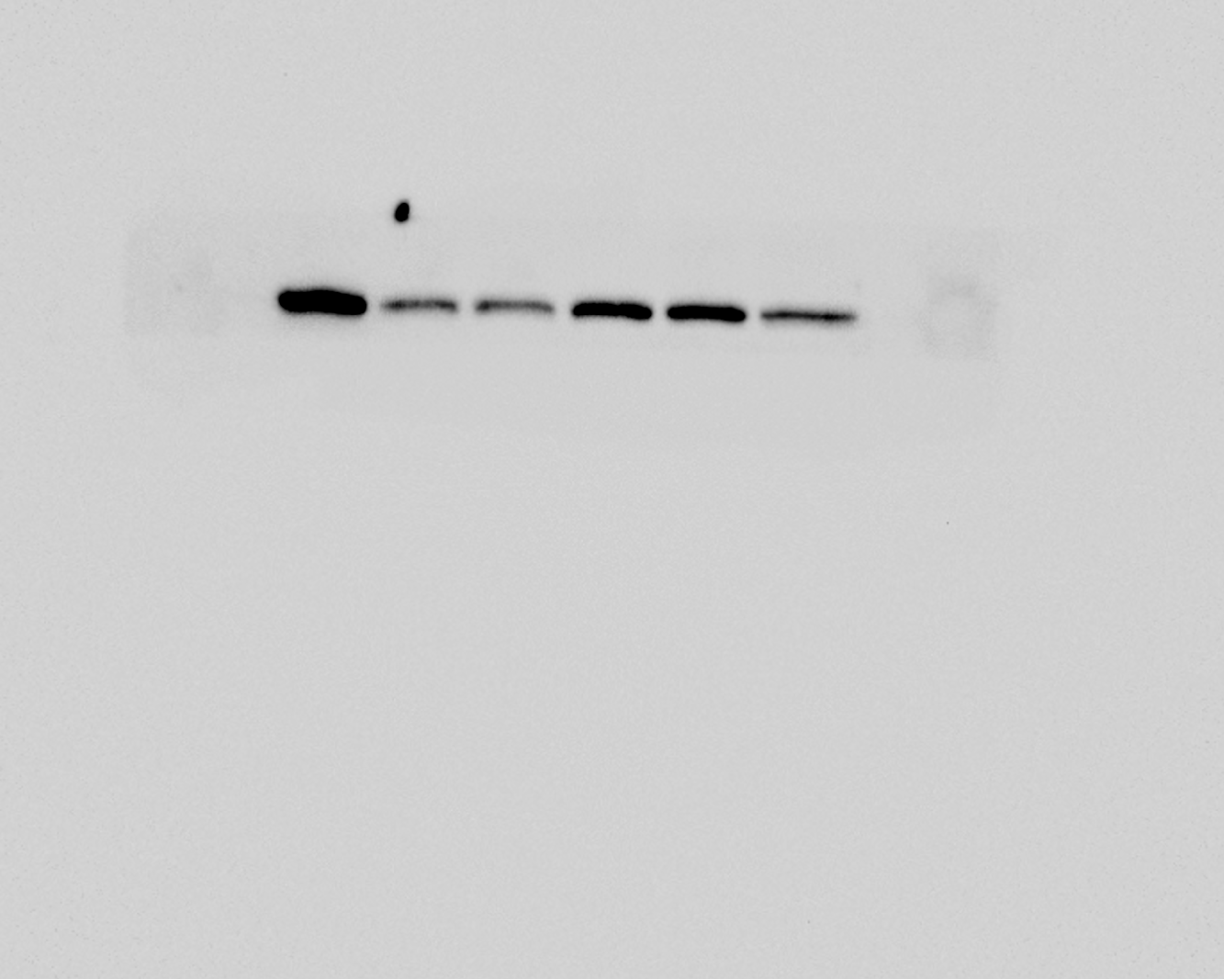

Supplement: Supplementary file 4 [file DataSheet9.ZIP › si-360-2/stat3ncsi_3(Chemiluminescence).tif]

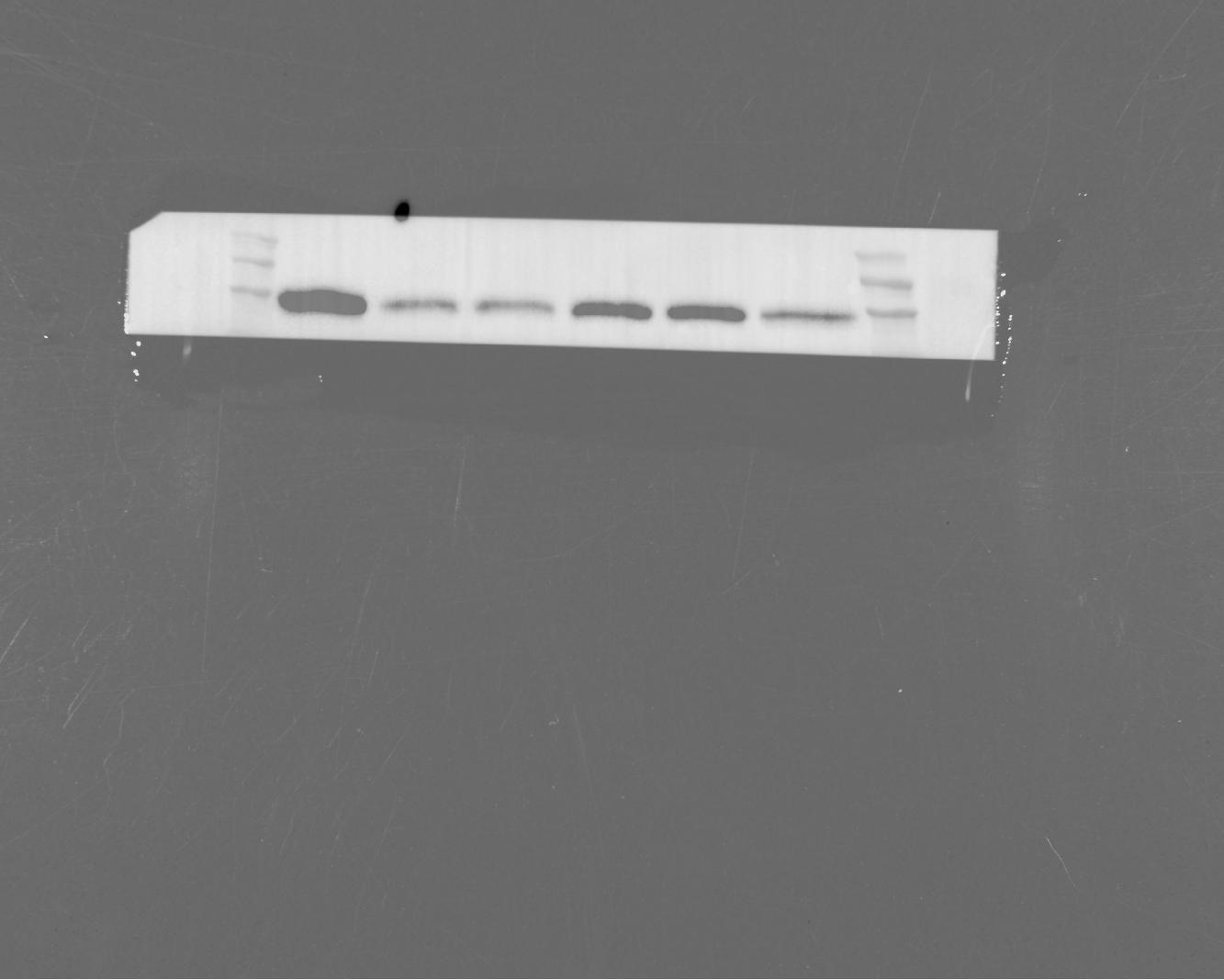

Supplement: Supplementary file 4 [file DataSheet9.ZIP › si-360-2/stat3ncsi_3(Composite).tif]

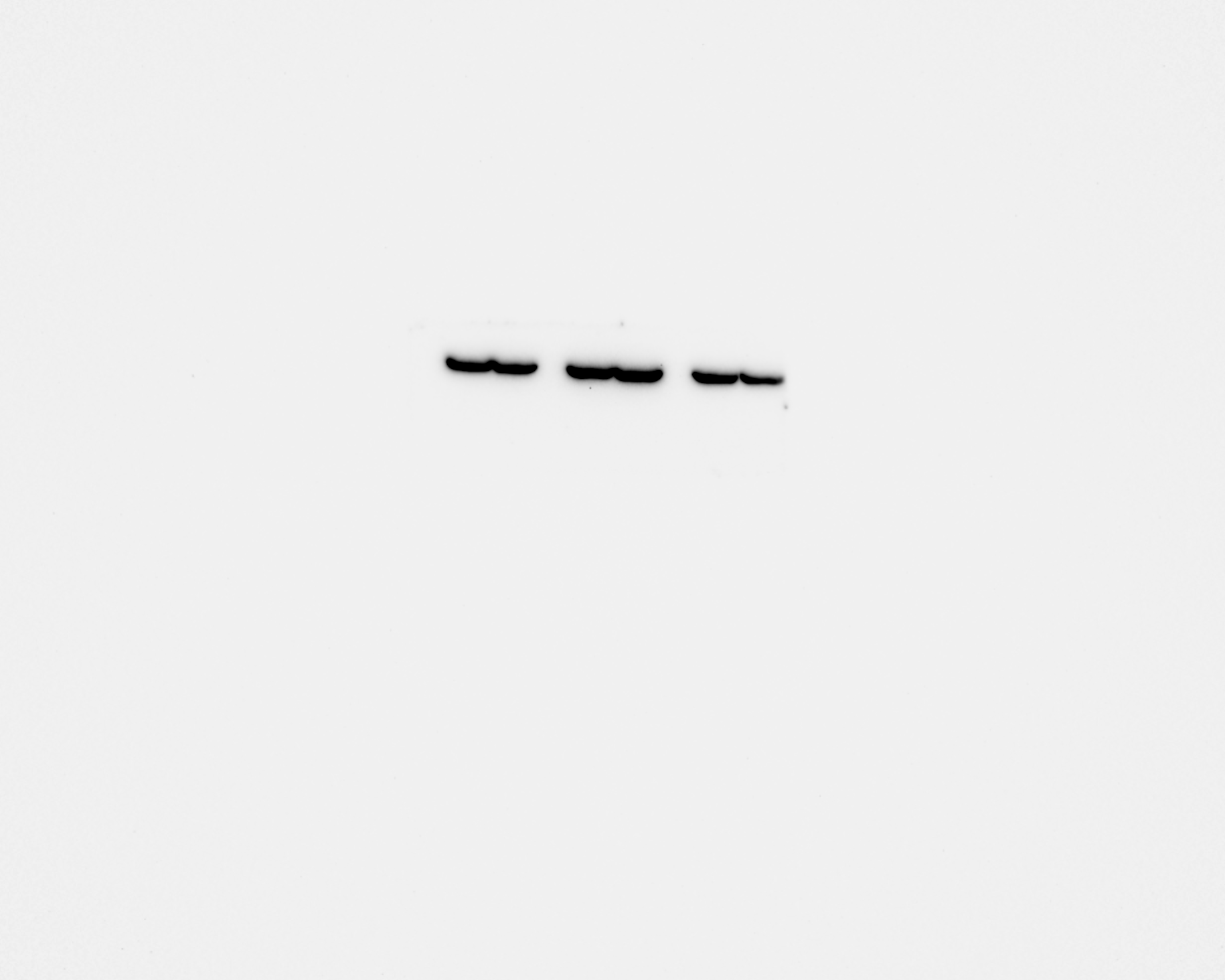

Supplement: Supplementary file 4 [file DataSheet9.ZIP › si-360-2/ZQ123 2022-04-25 17h48m51s(Chemiluminescence).tif]

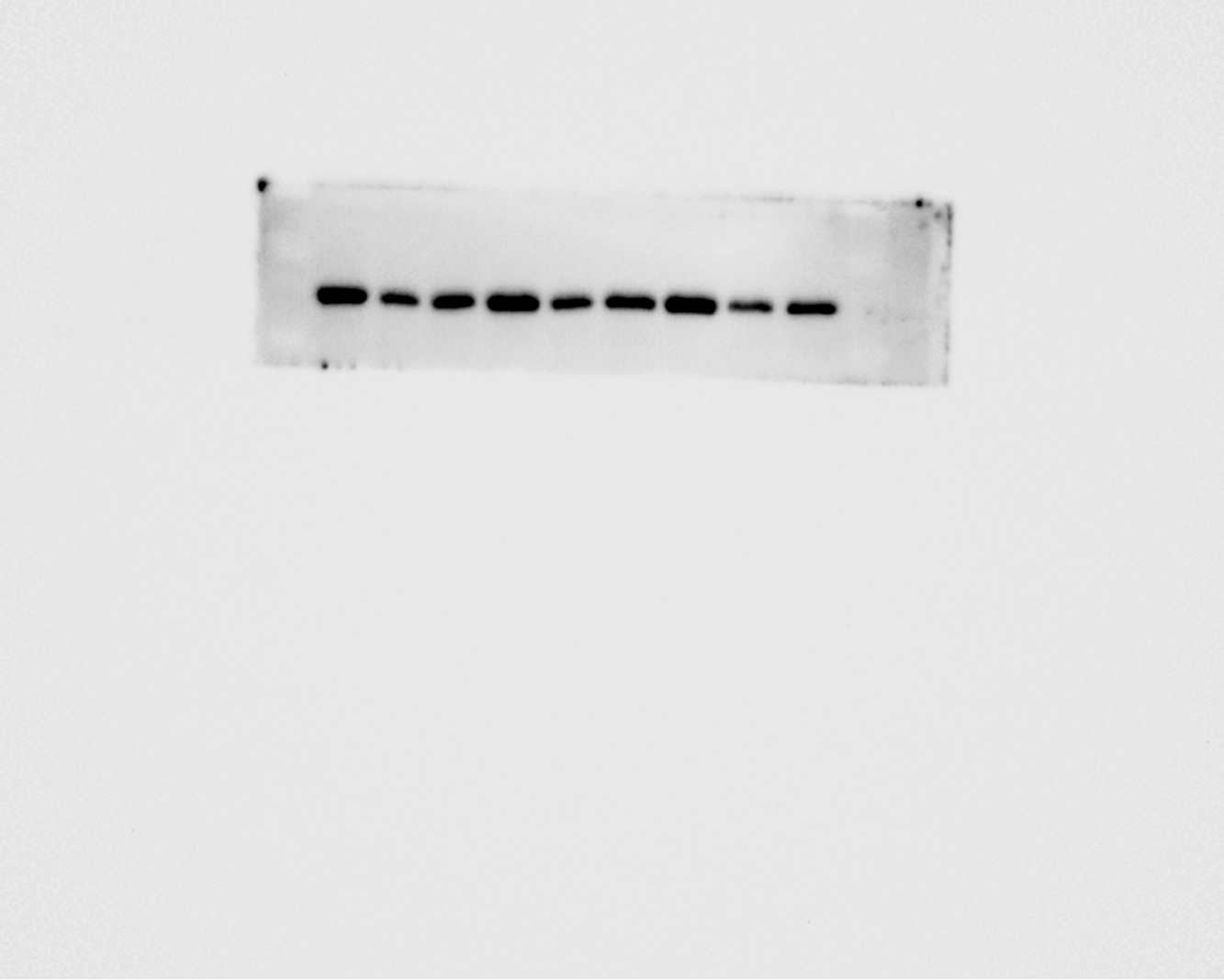

Supplement: Supplementary file 5 [file DataSheet4.ZIP › act_3(Chemiluminescence).tif]

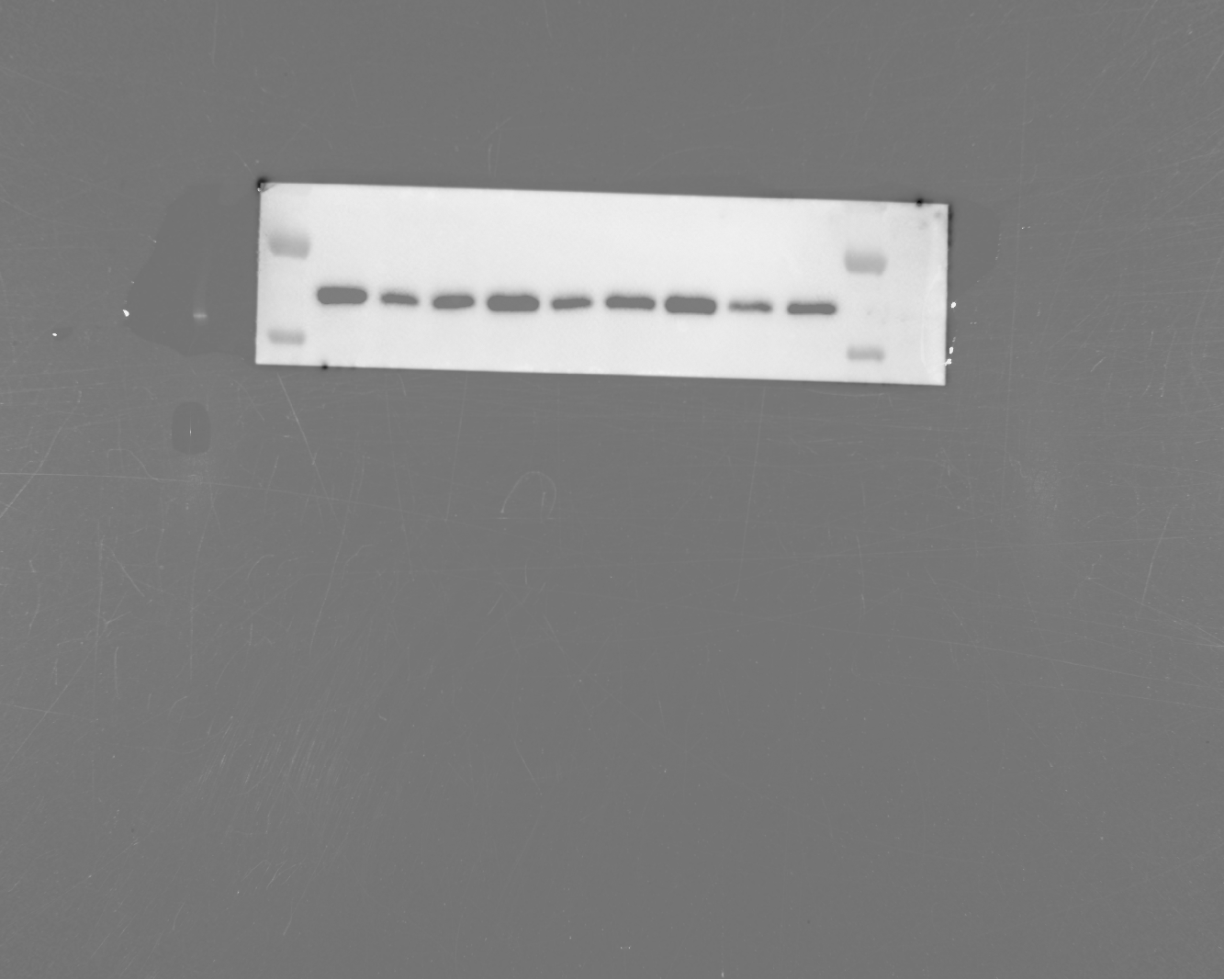

Supplement: Supplementary file 5 [file DataSheet4.ZIP › act_3(Composite).tif]

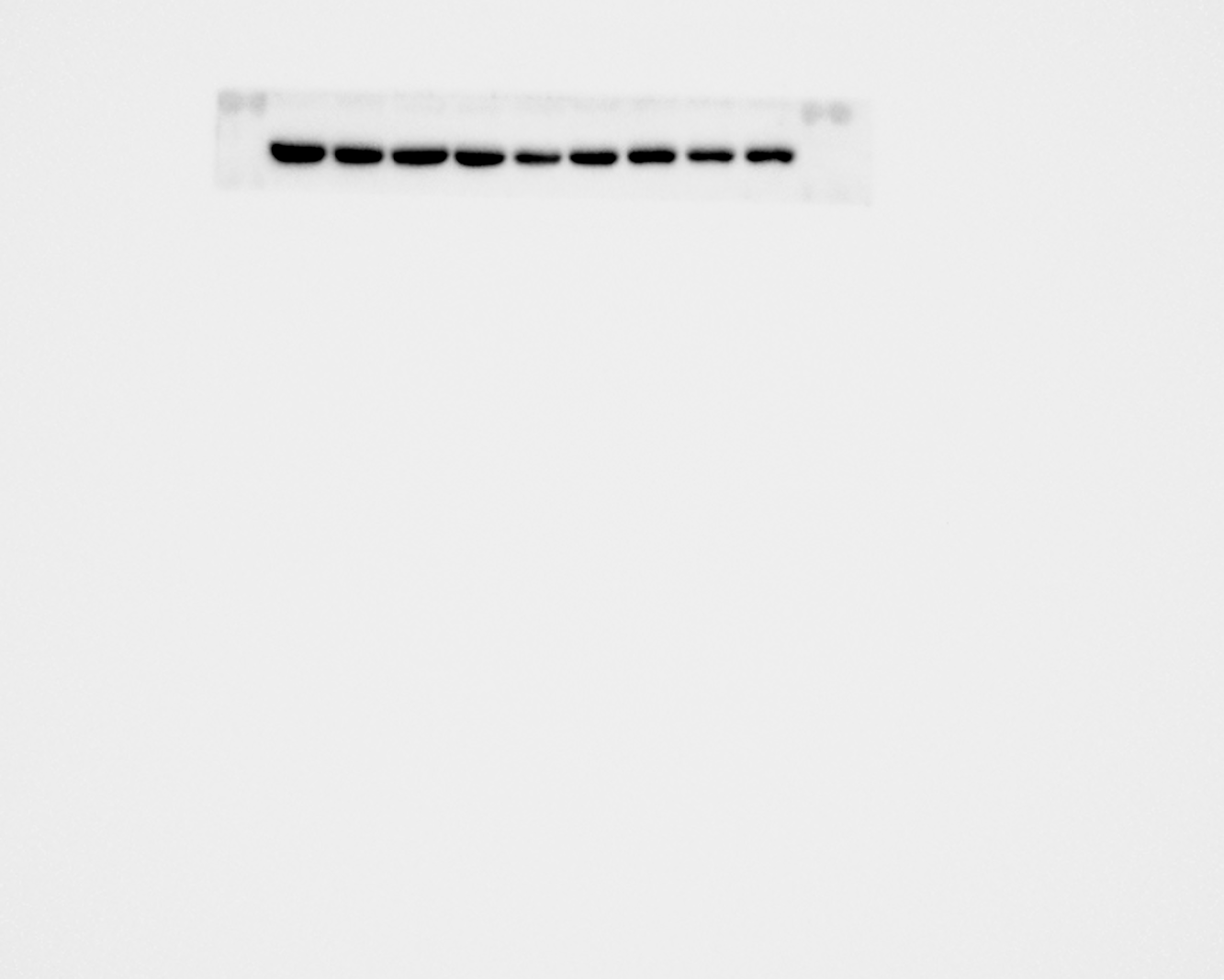

Supplement: Supplementary file 5 [file DataSheet4.ZIP › actin13_4(Chemiluminescence).tif]

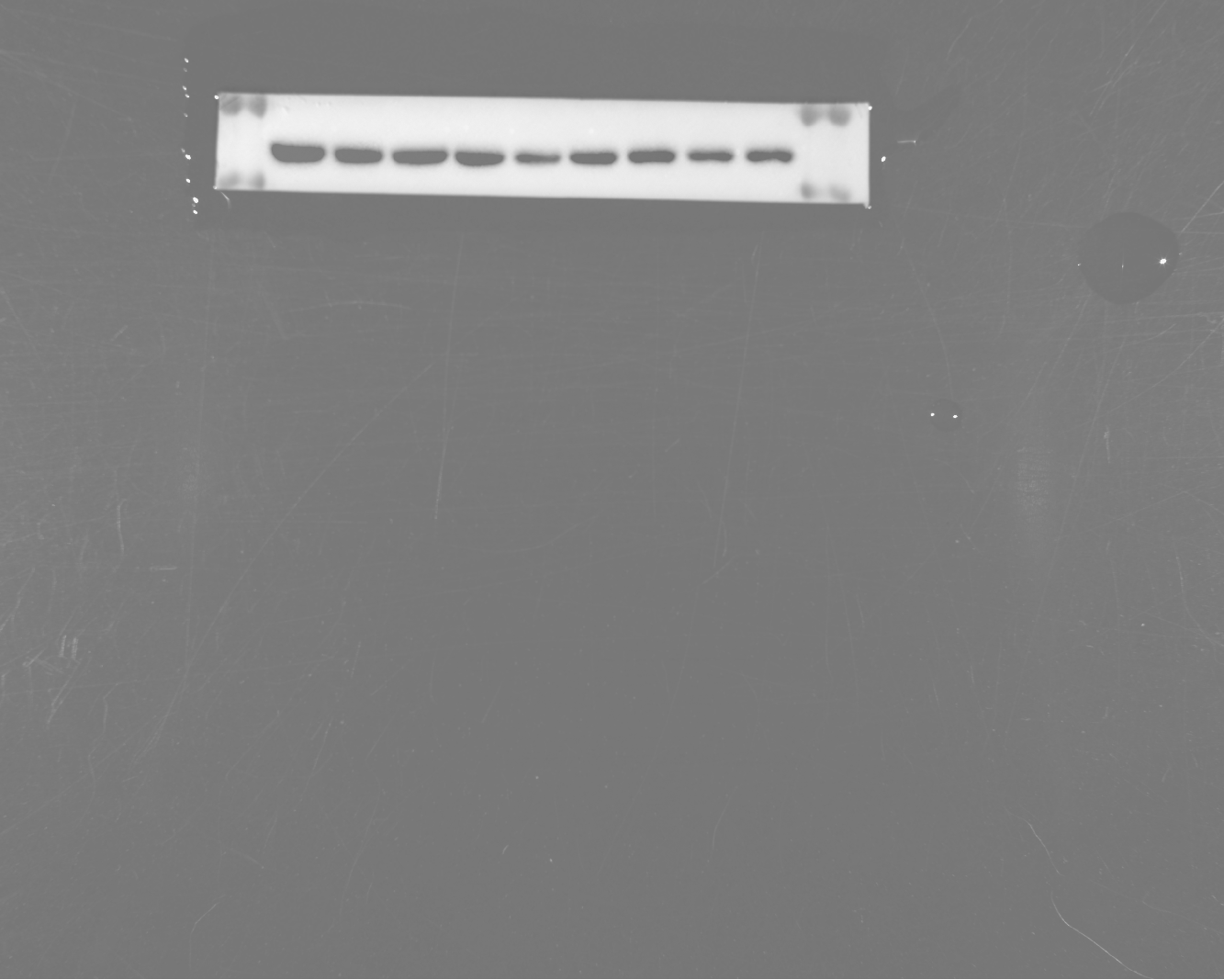

Supplement: Supplementary file 5 [file DataSheet4.ZIP › actin13_4(Composite).tif]

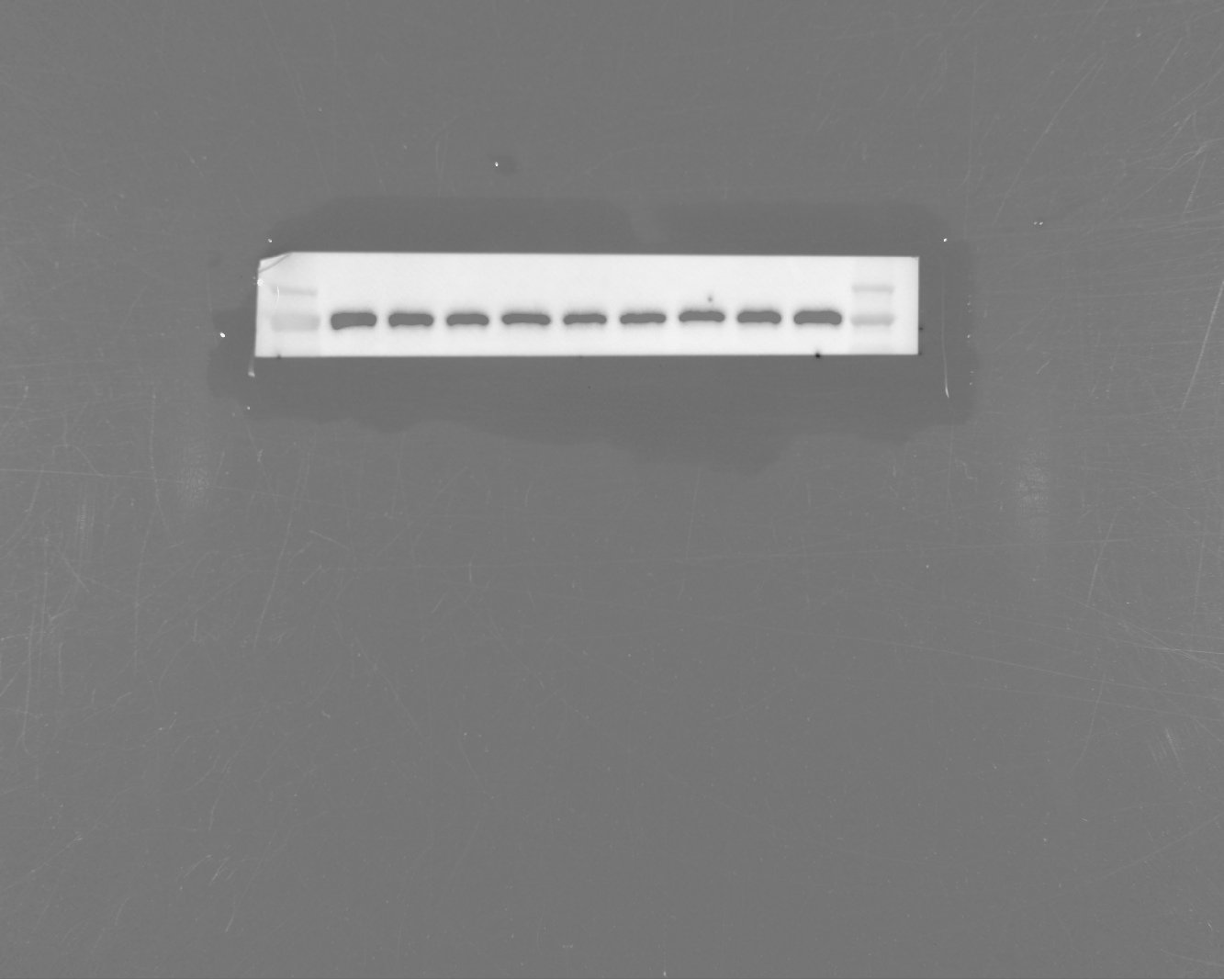

Supplement: Supplementary file 5 [file DataSheet4.ZIP › actin-3 (1).tif]

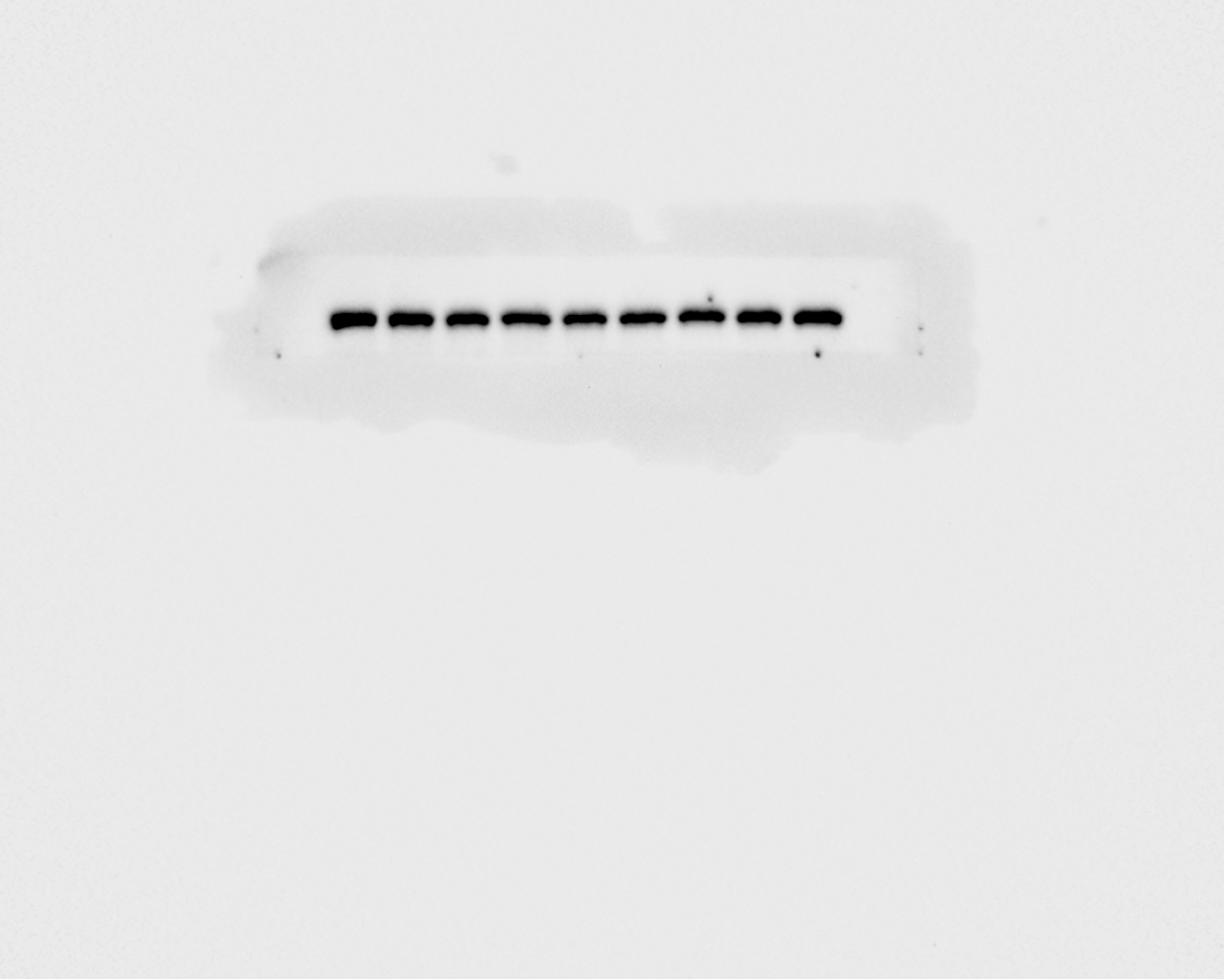

Supplement: Supplementary file 5 [file DataSheet4.ZIP › actin-3 (2).tif]

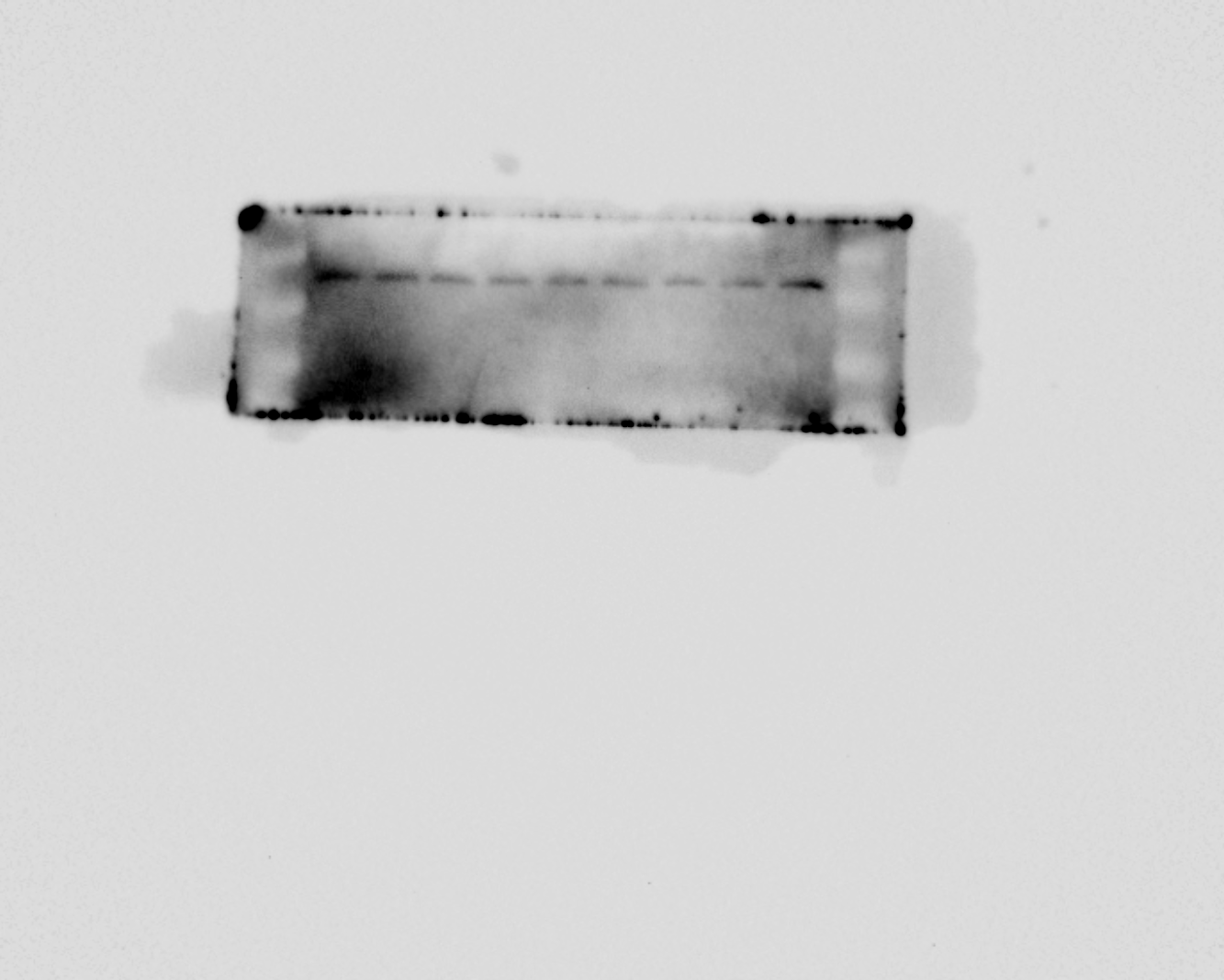

Supplement: Supplementary file 5 [file DataSheet4.ZIP › actin-3_1(Chemiluminescence).tif]

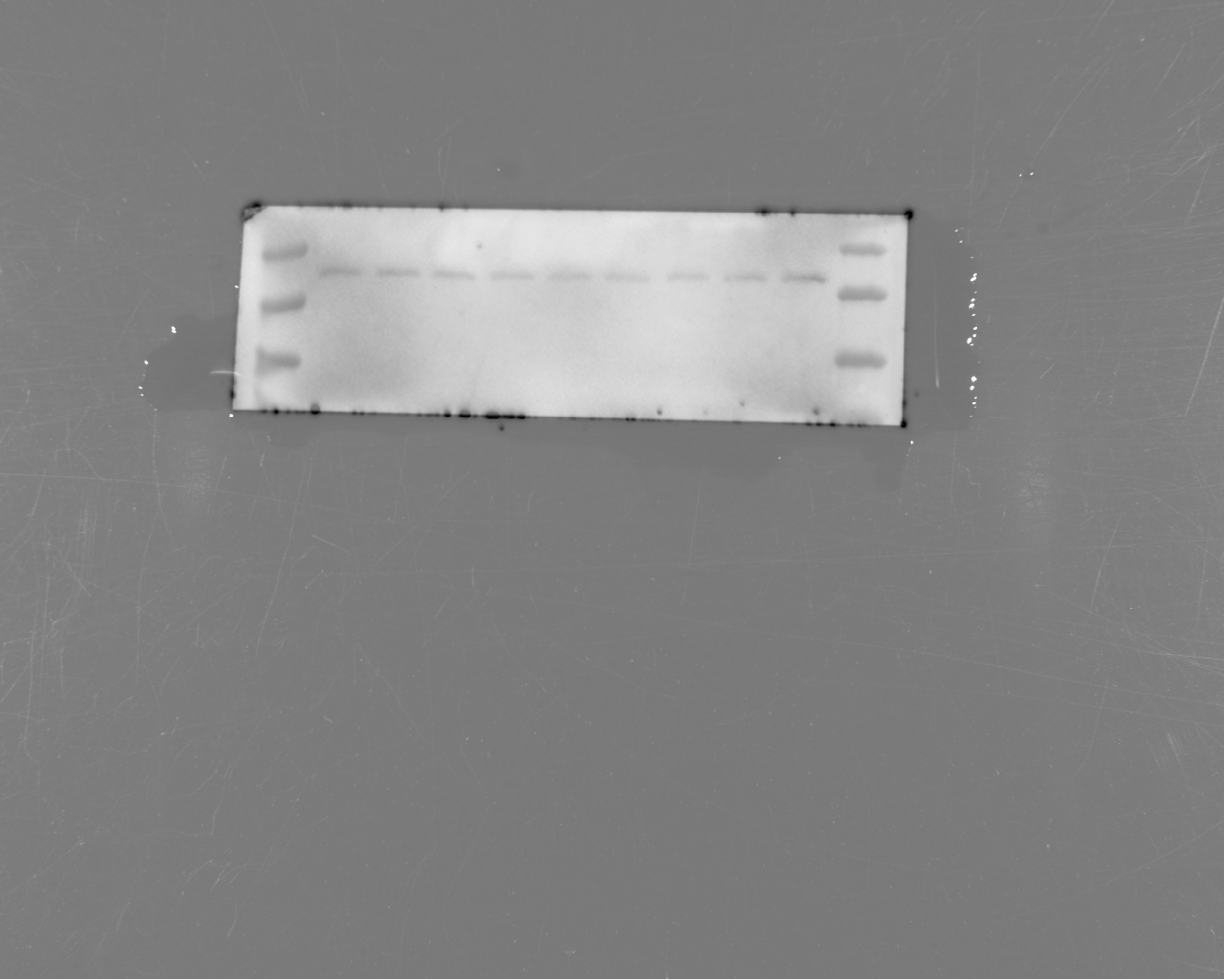

Supplement: Supplementary file 5 [file DataSheet4.ZIP › actin-3_4(Composite).tif]

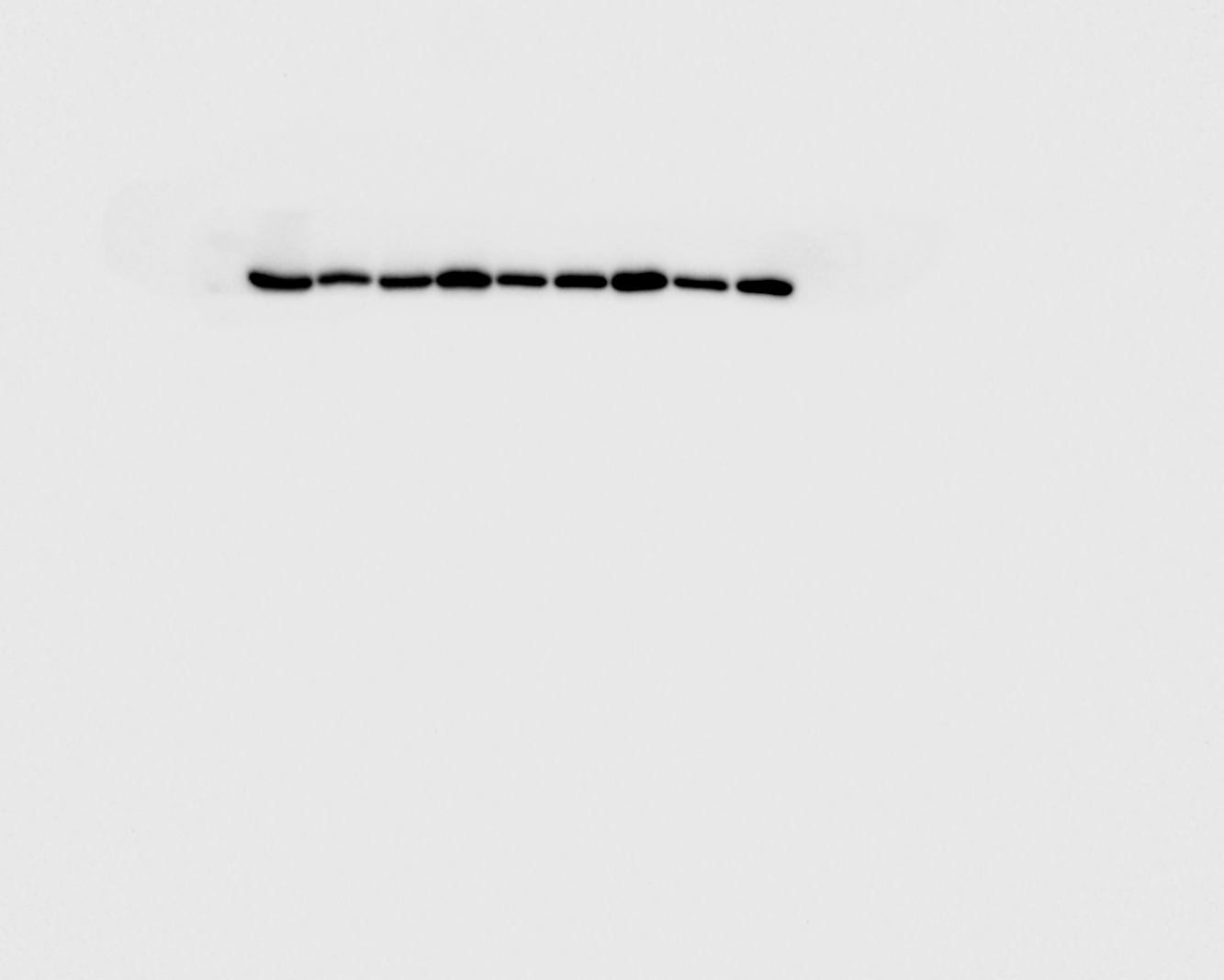

Supplement: Supplementary file 5 [file DataSheet4.ZIP › actin-4_1(Chemiluminescence).tif]

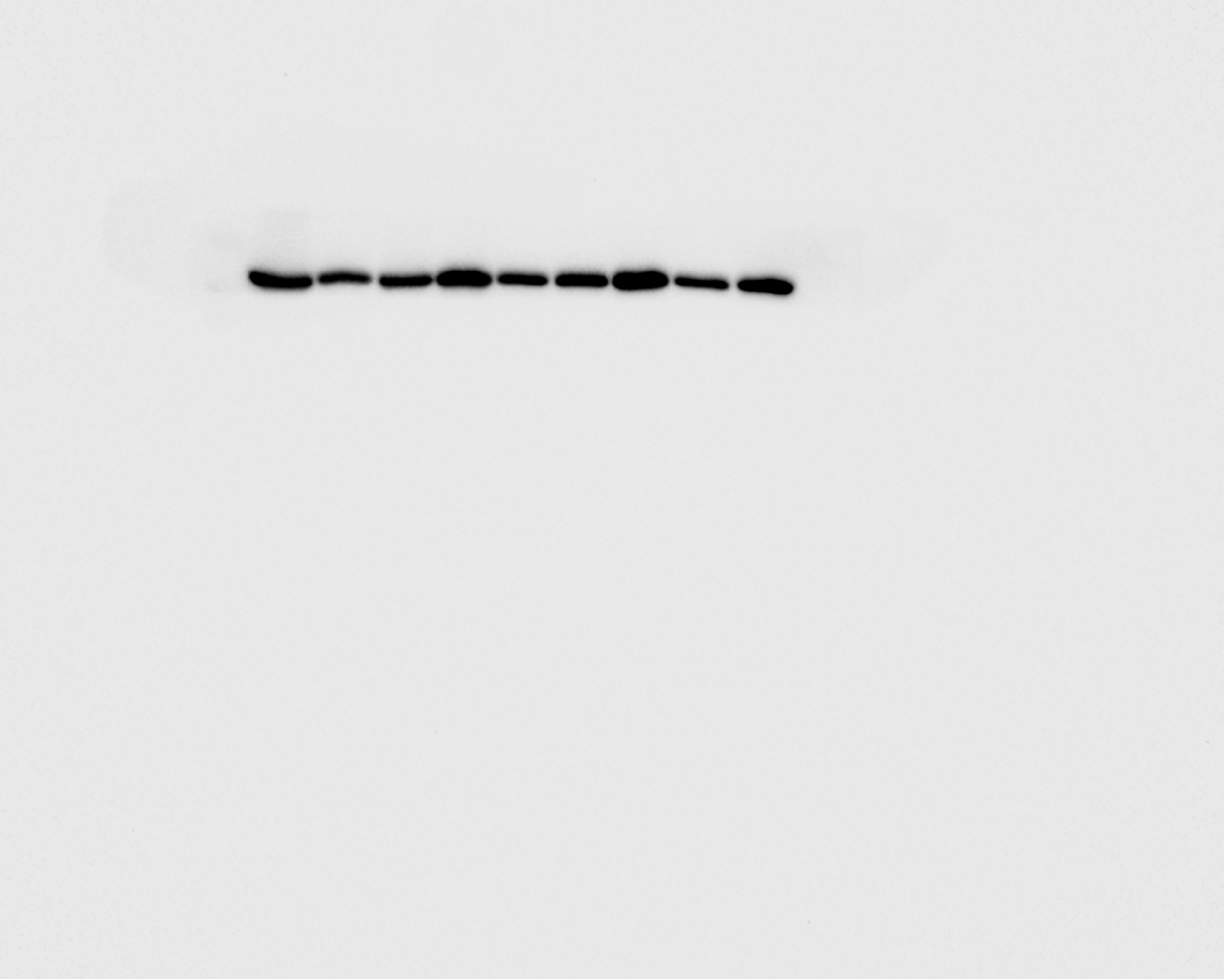

Supplement: Supplementary file 5 [file DataSheet4.ZIP › actin-4_3(Chemiluminescence).tif]

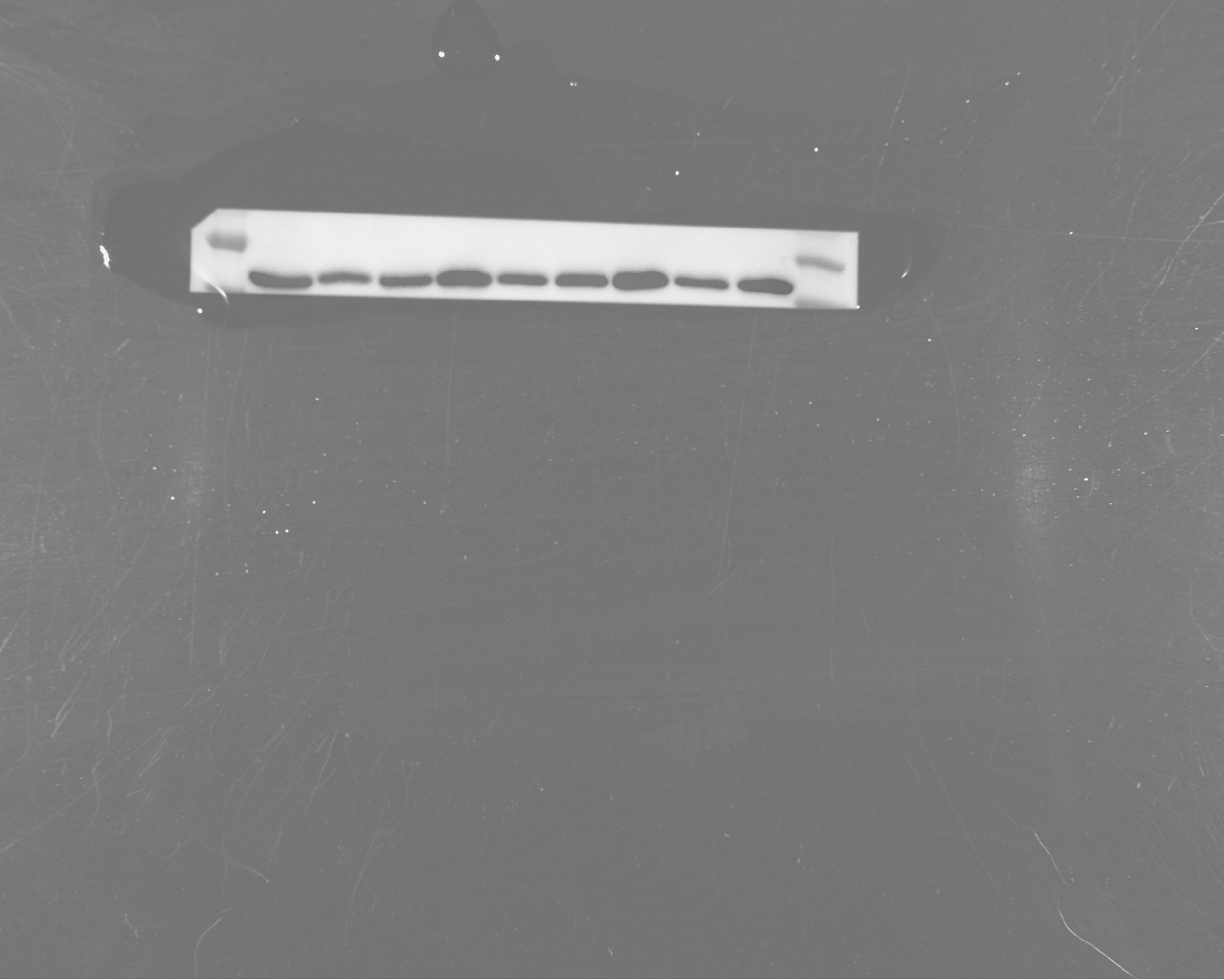

Supplement: Supplementary file 5 [file DataSheet4.ZIP › actin-4_3(Composite).tif]

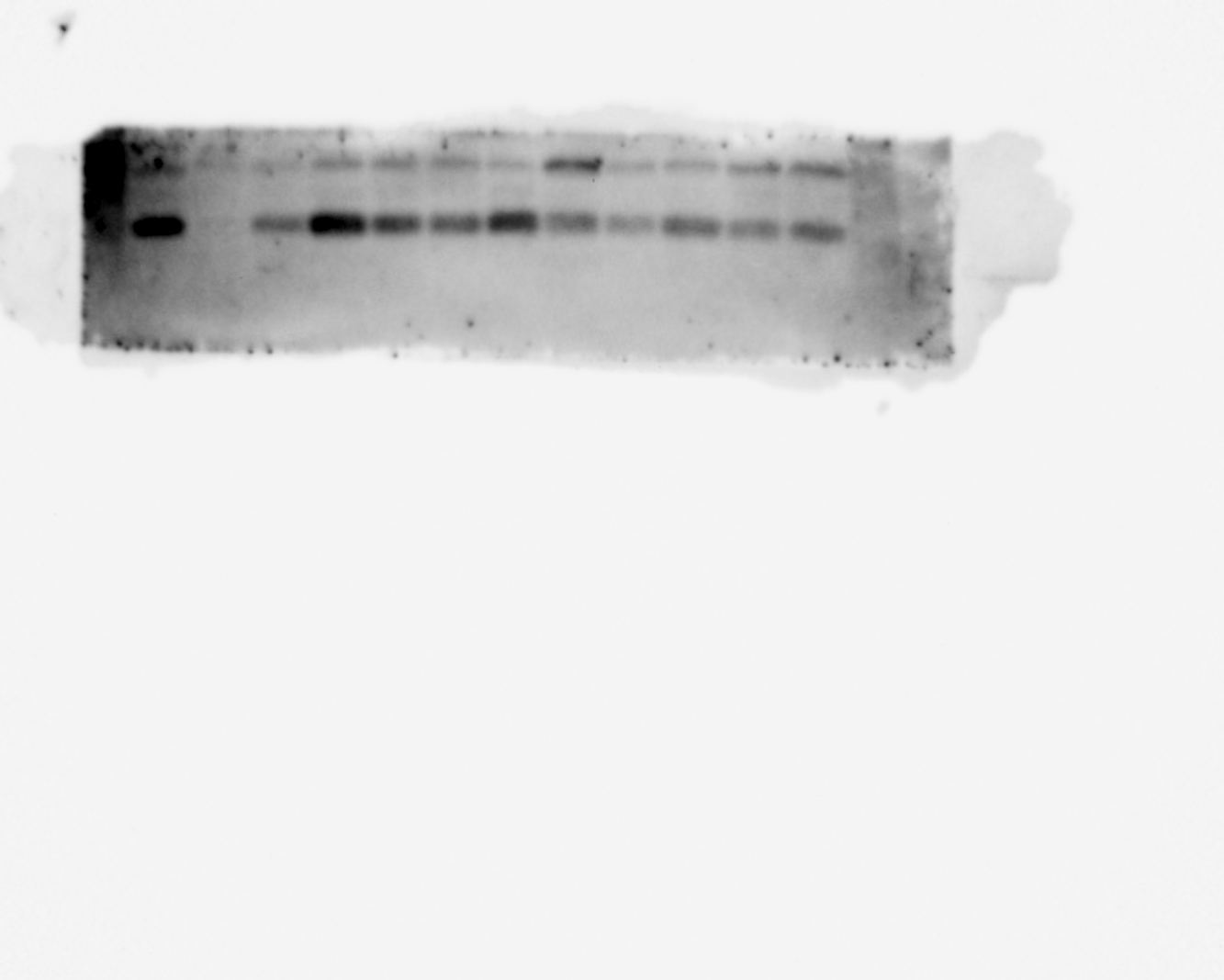

Supplement: Supplementary file 5 [file DataSheet4.ZIP › bax.tif]

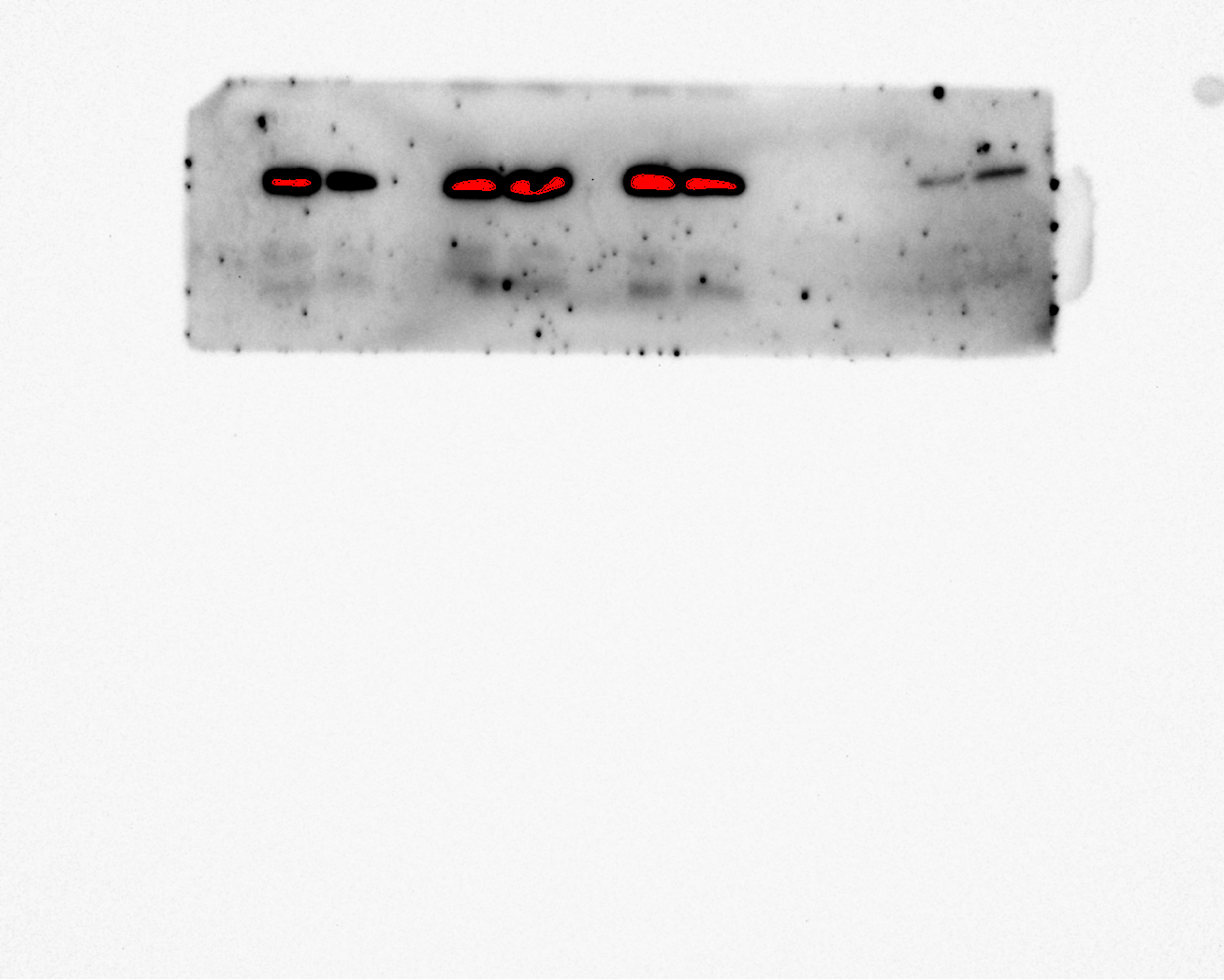

Supplement: Supplementary file 5 [file DataSheet4.ZIP › bax+cc3_6(Chemiluminescence).tif]

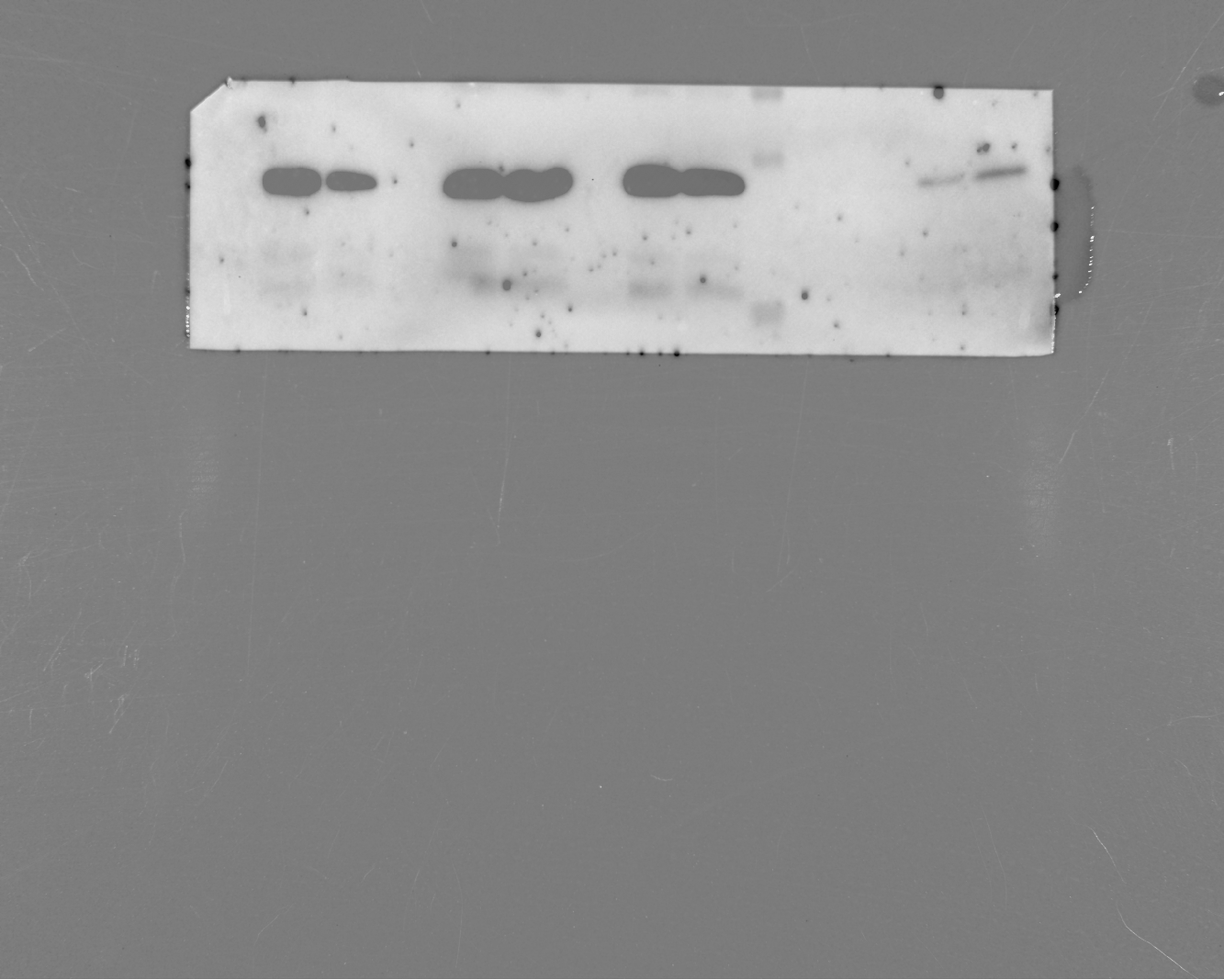

Supplement: Supplementary file 5 [file DataSheet4.ZIP › bax+cc3_6(Composite).tif]

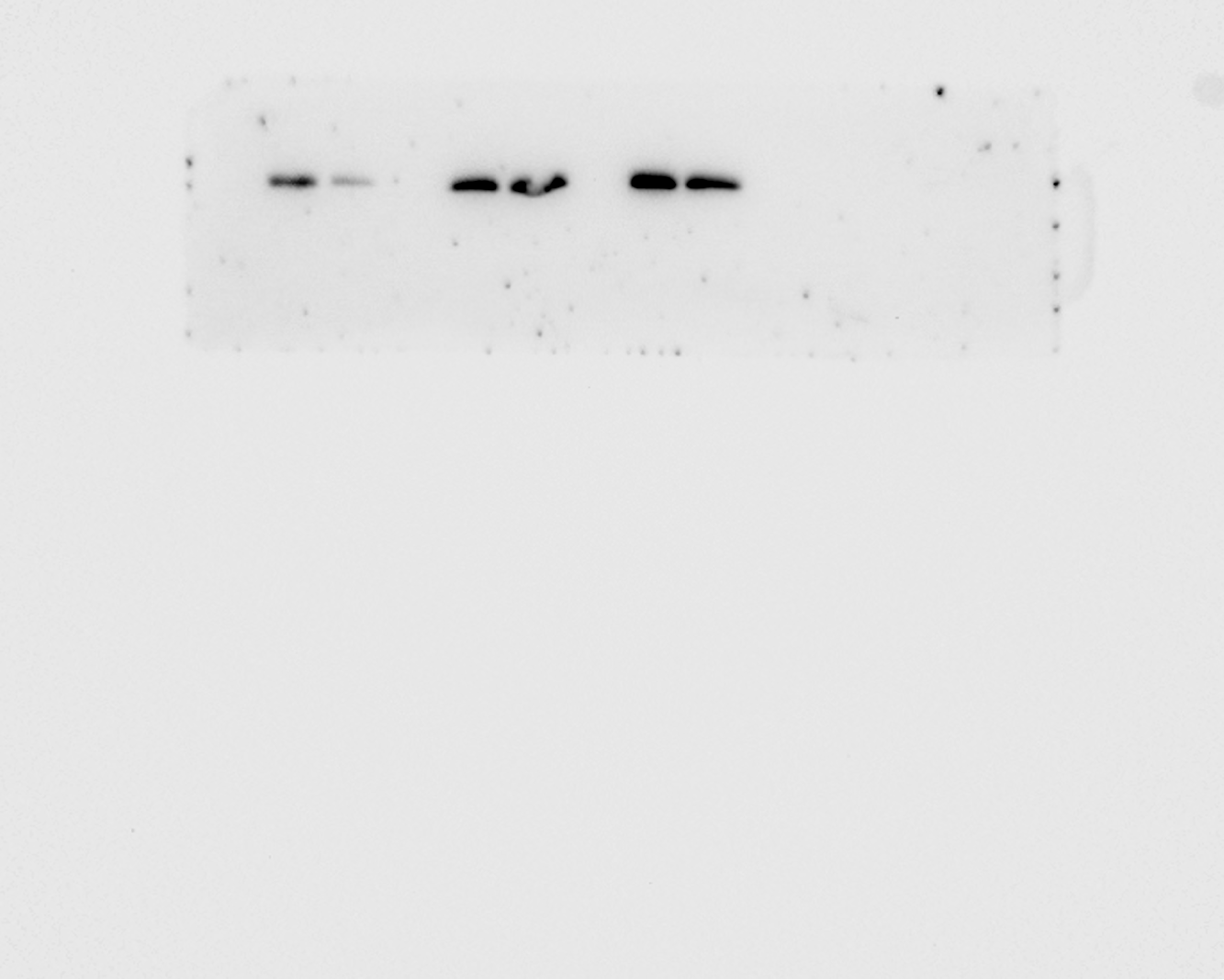

Supplement: Supplementary file 5 [file DataSheet4.ZIP › bax+cc3_7(Chemiluminescence).tif]

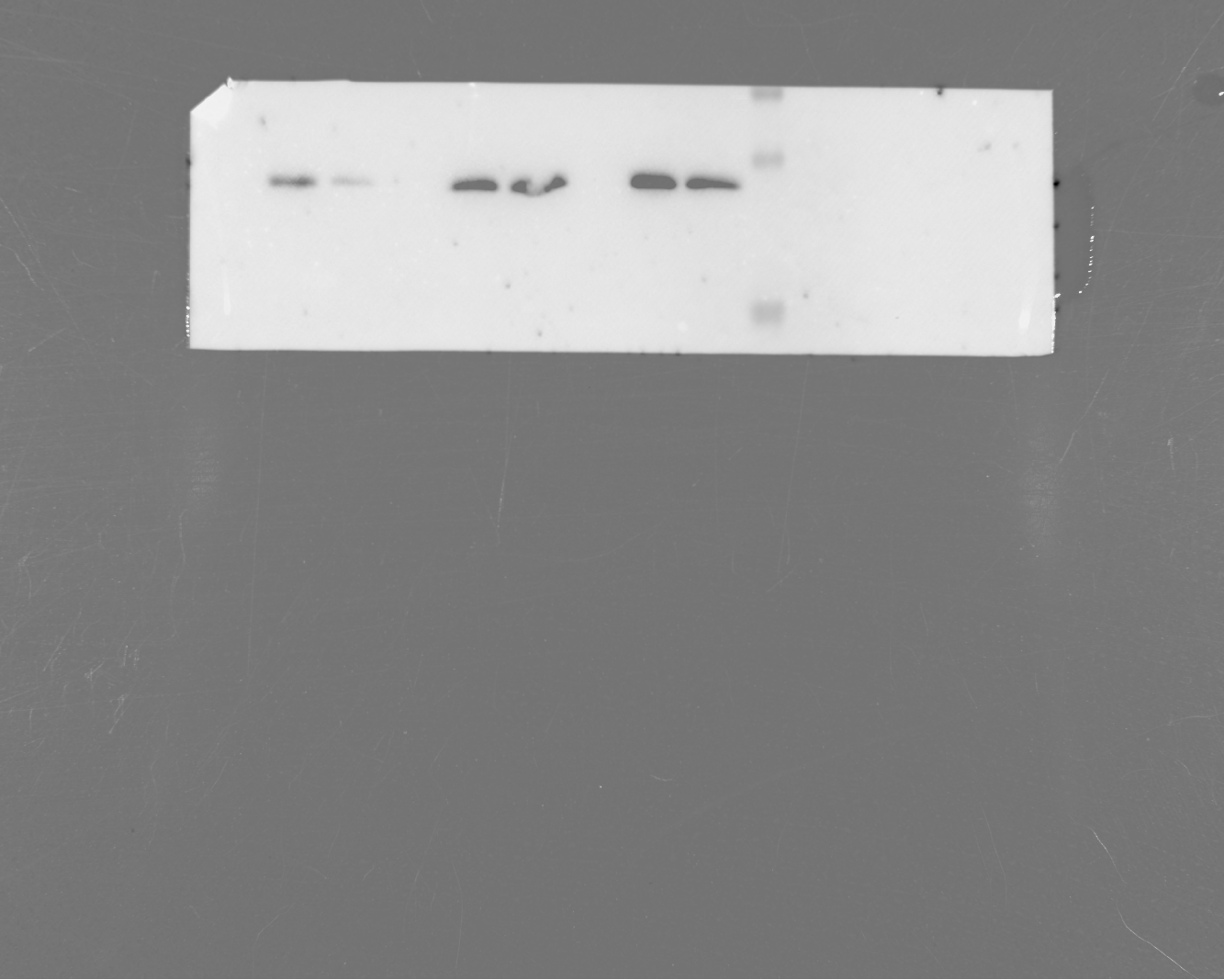

Supplement: Supplementary file 5 [file DataSheet4.ZIP › bax+cc3_7(Composite).tif]

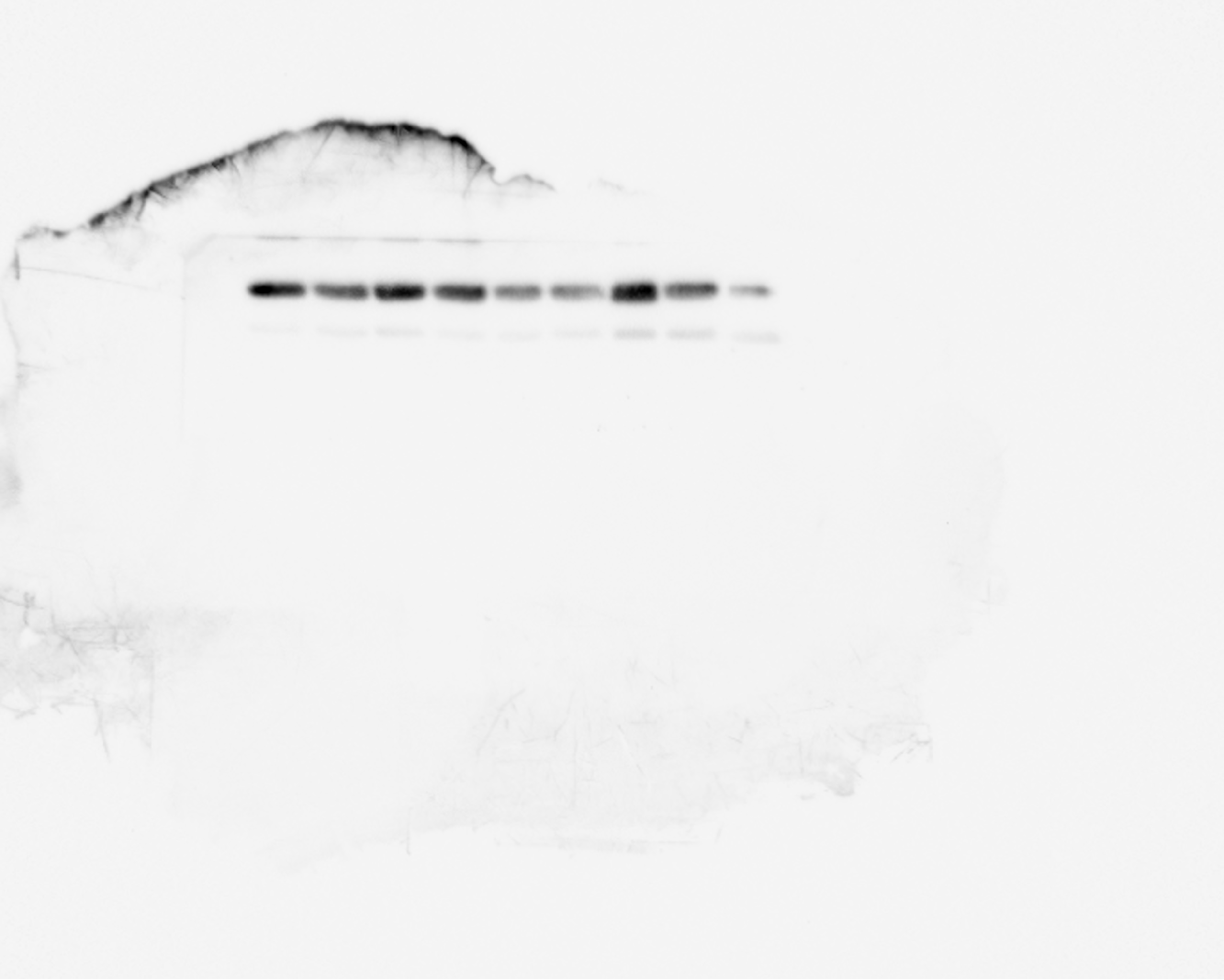

Supplement: Supplementary file 5 [file DataSheet4.ZIP › bcl2-6_3(Chemiluminescence).tif]

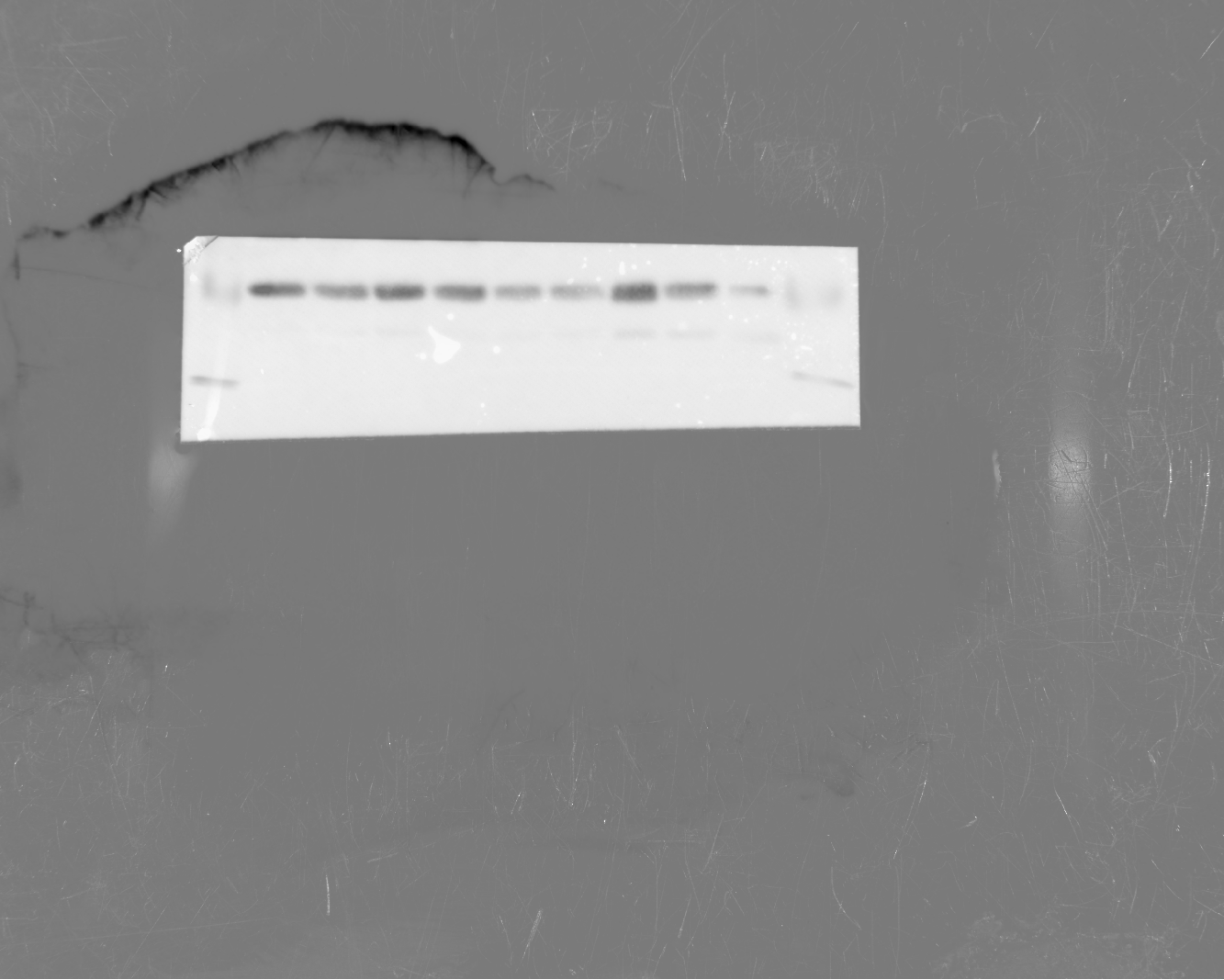

Supplement: Supplementary file 5 [file DataSheet4.ZIP › bcl2-6_3(Composite).tif]
