# Supplementary figures and images for: Effects of Xiao Chengqi Formula on Slow Transit Constipation by Assessing Gut Microbiota and Metabolomics Analysis in vitro and in vivo (part 2 of 3)
Source: Front Pharmacol. 2022 Jun 8;13:864598. doi: 10.3389/fphar.2022.864598 (PMC9237644; doi:10.3389/fphar.2022.864598)

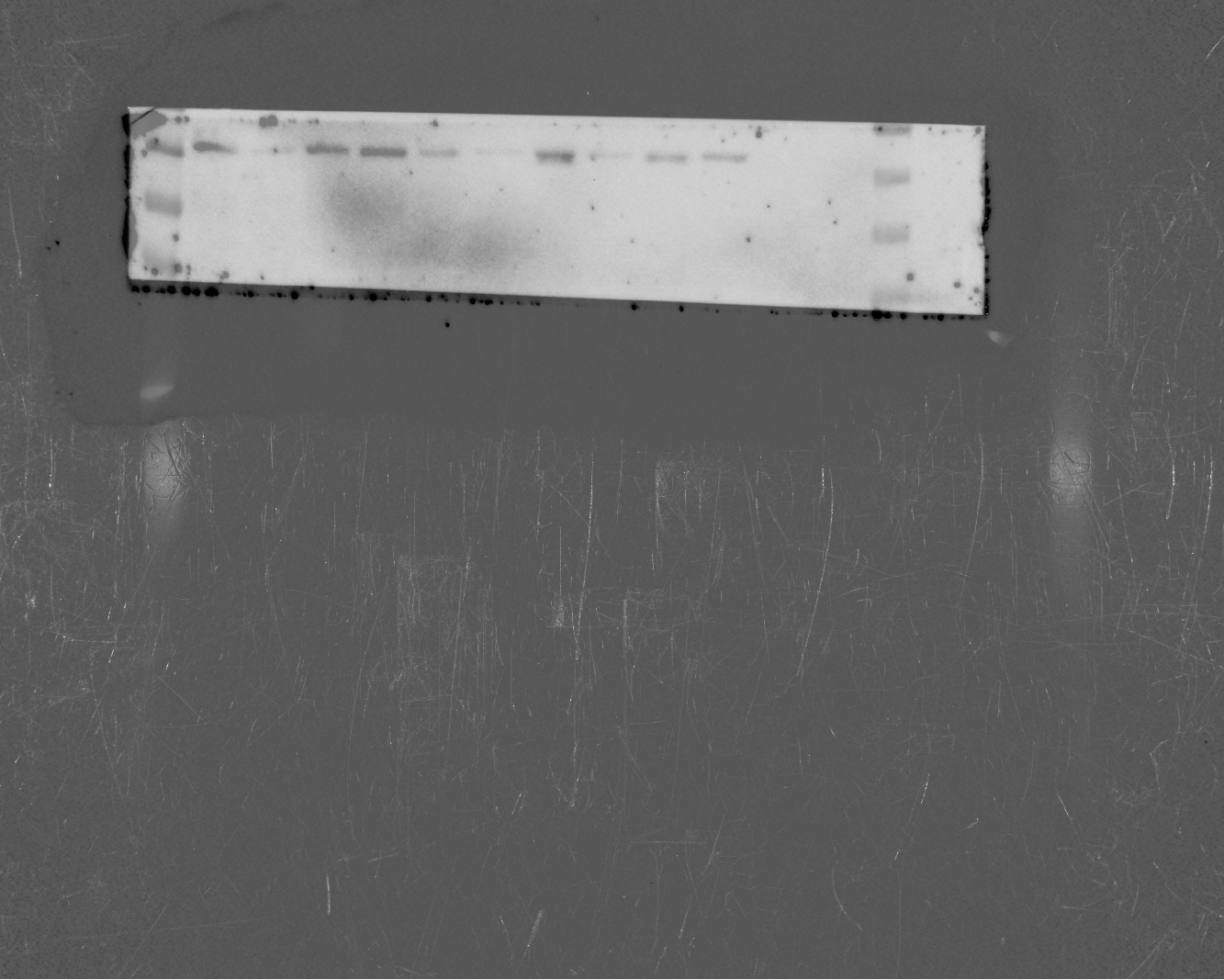

Supplement: Supplementary file 5 [file DataSheet4.ZIP › cas3-2 (1).tif]

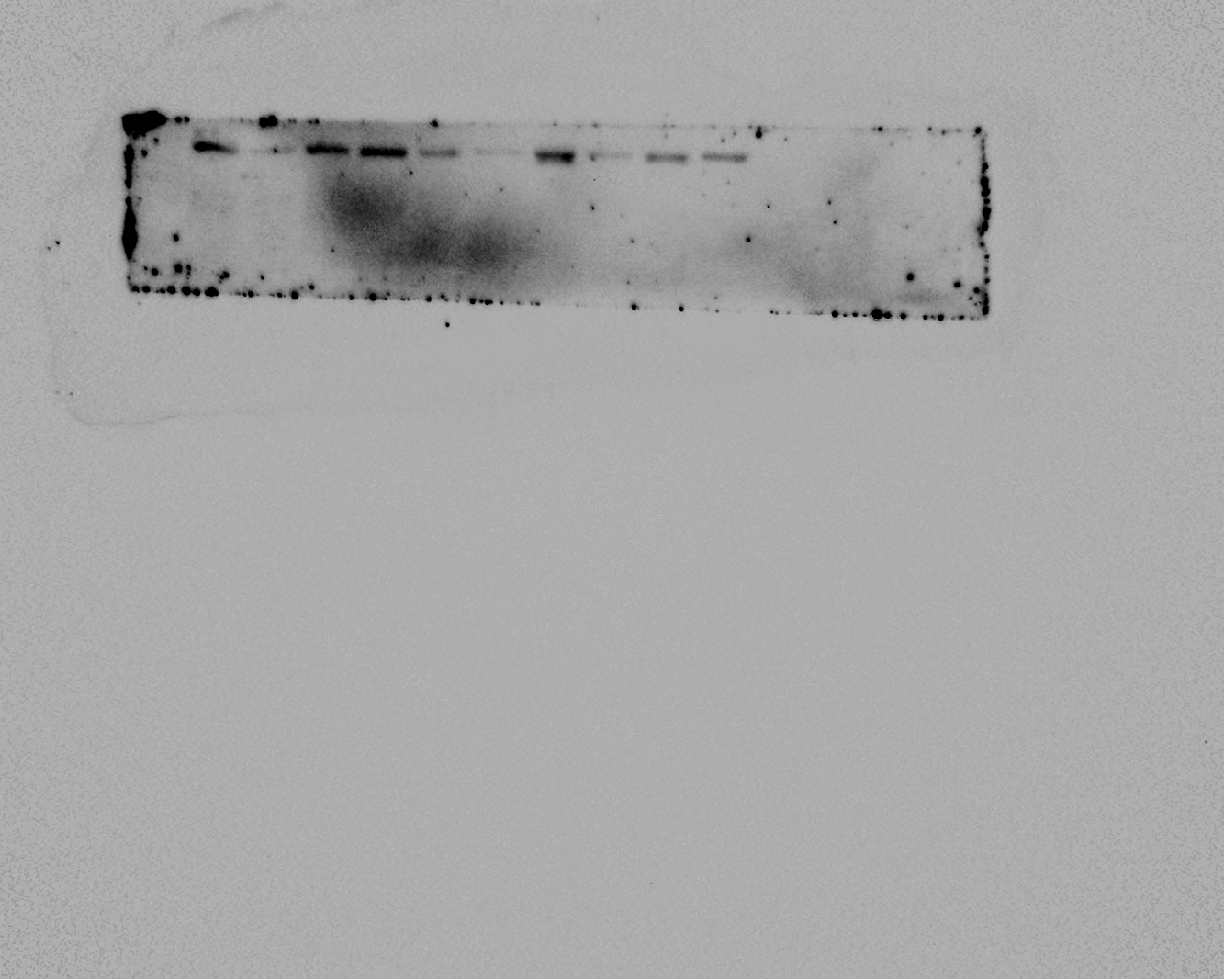

Supplement: Supplementary file 5 [file DataSheet4.ZIP › cas3-2 (2).tif]

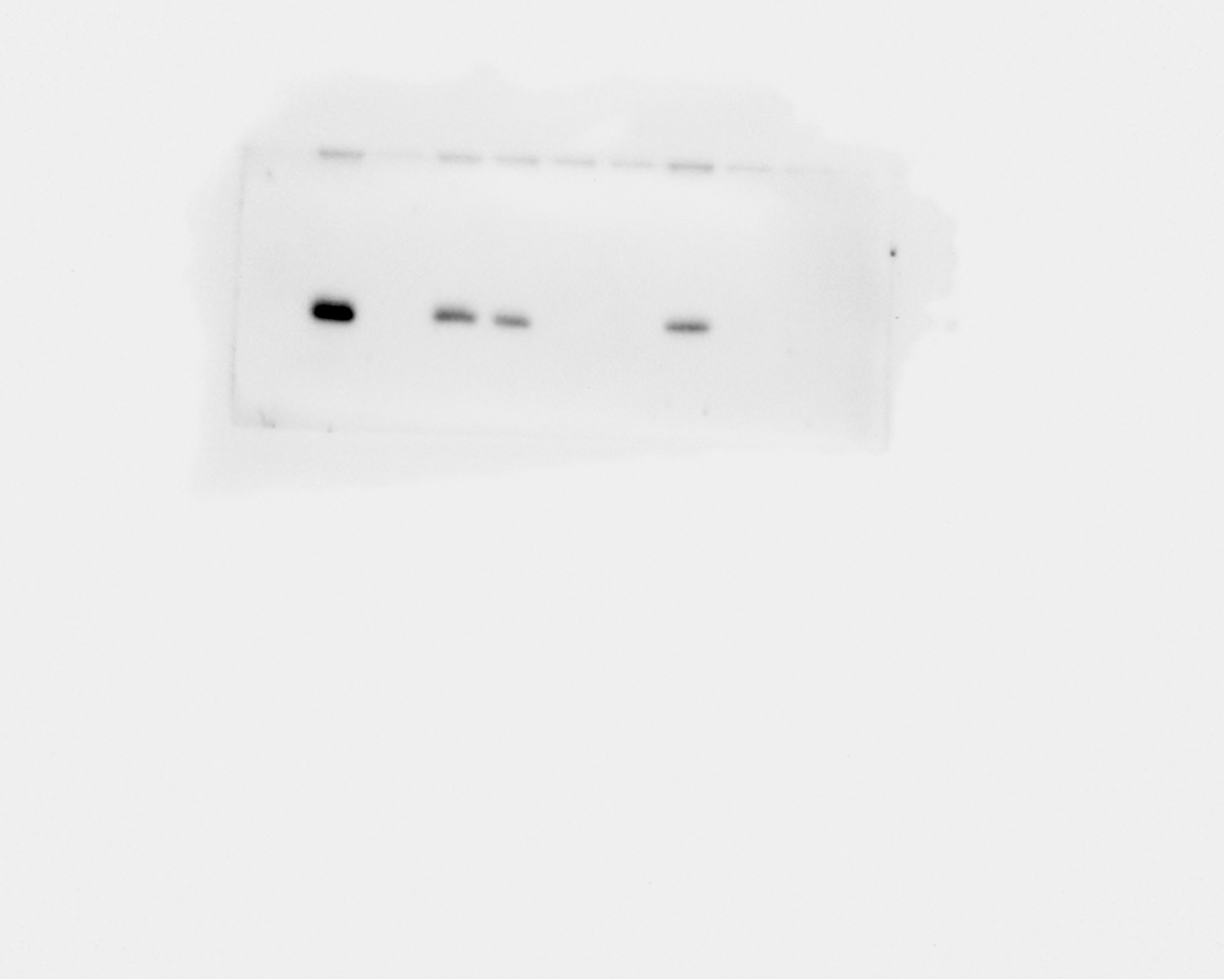

Supplement: Supplementary file 5 [file DataSheet4.ZIP › cas3-2-17_3(Chemiluminescence) (1).tif]

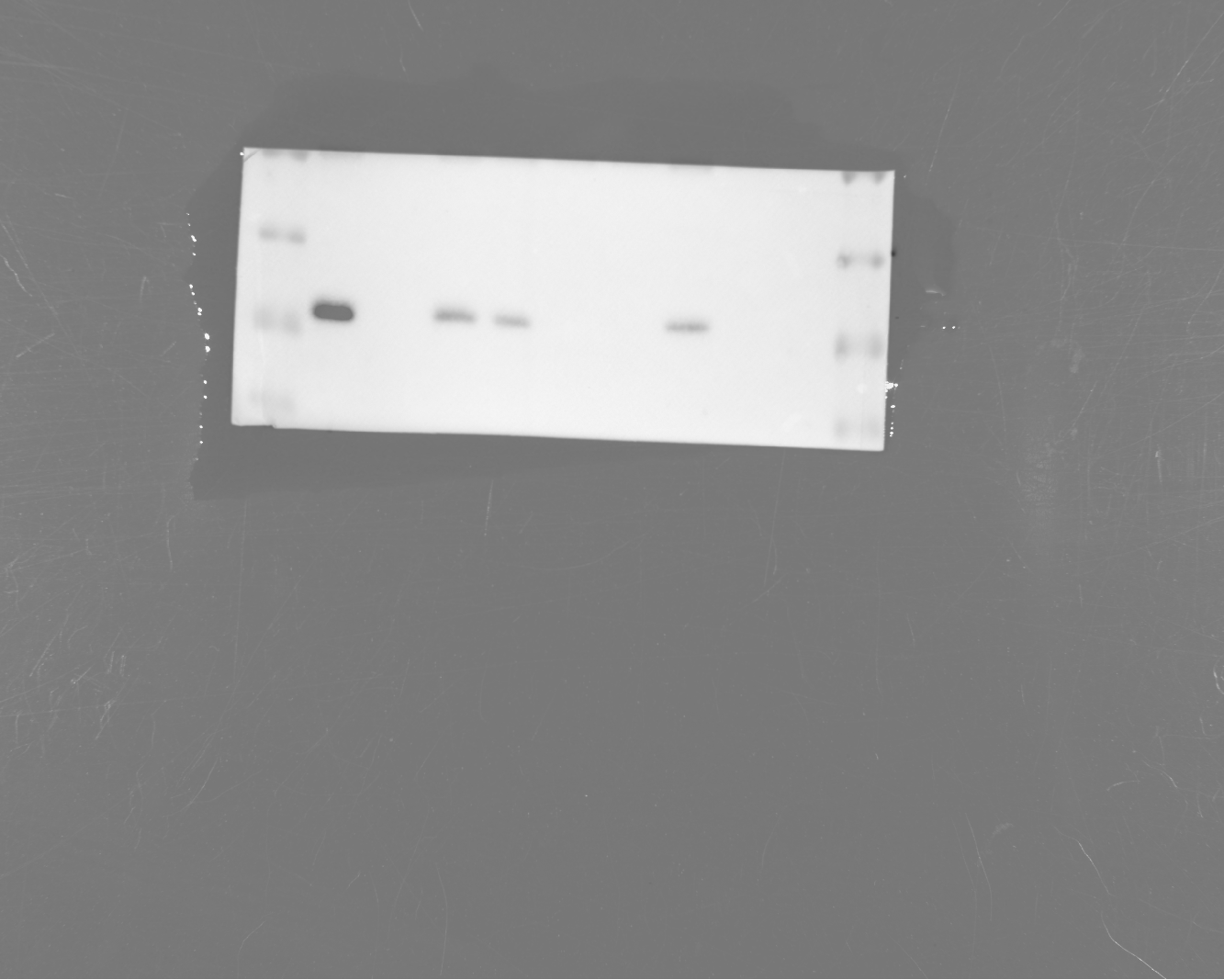

Supplement: Supplementary file 5 [file DataSheet4.ZIP › cas3-2-17_3(Chemiluminescence) (3).tif]

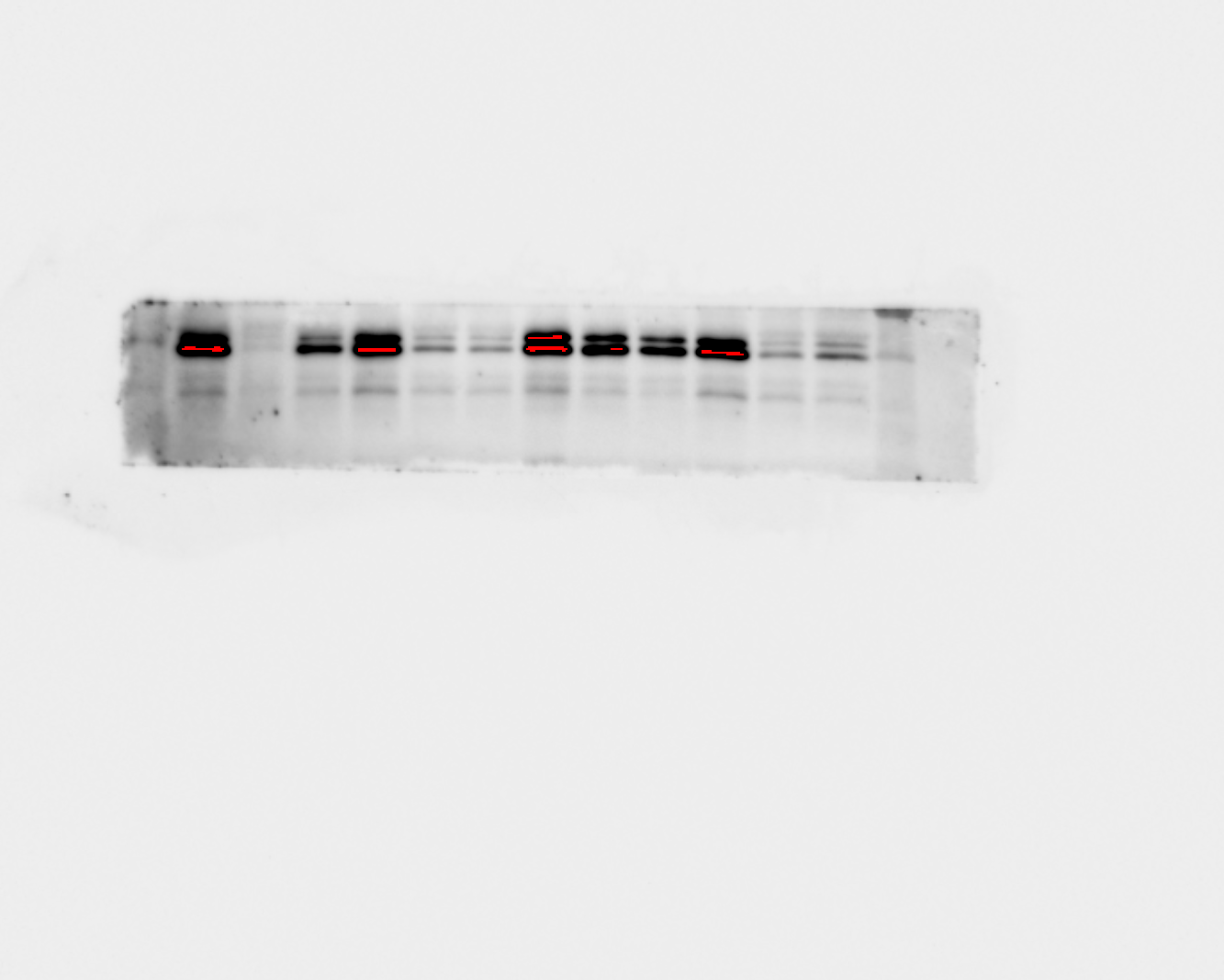

Supplement: Supplementary file 5 [file DataSheet4.ZIP › cas3sea_2(Chemiluminescence).tif]

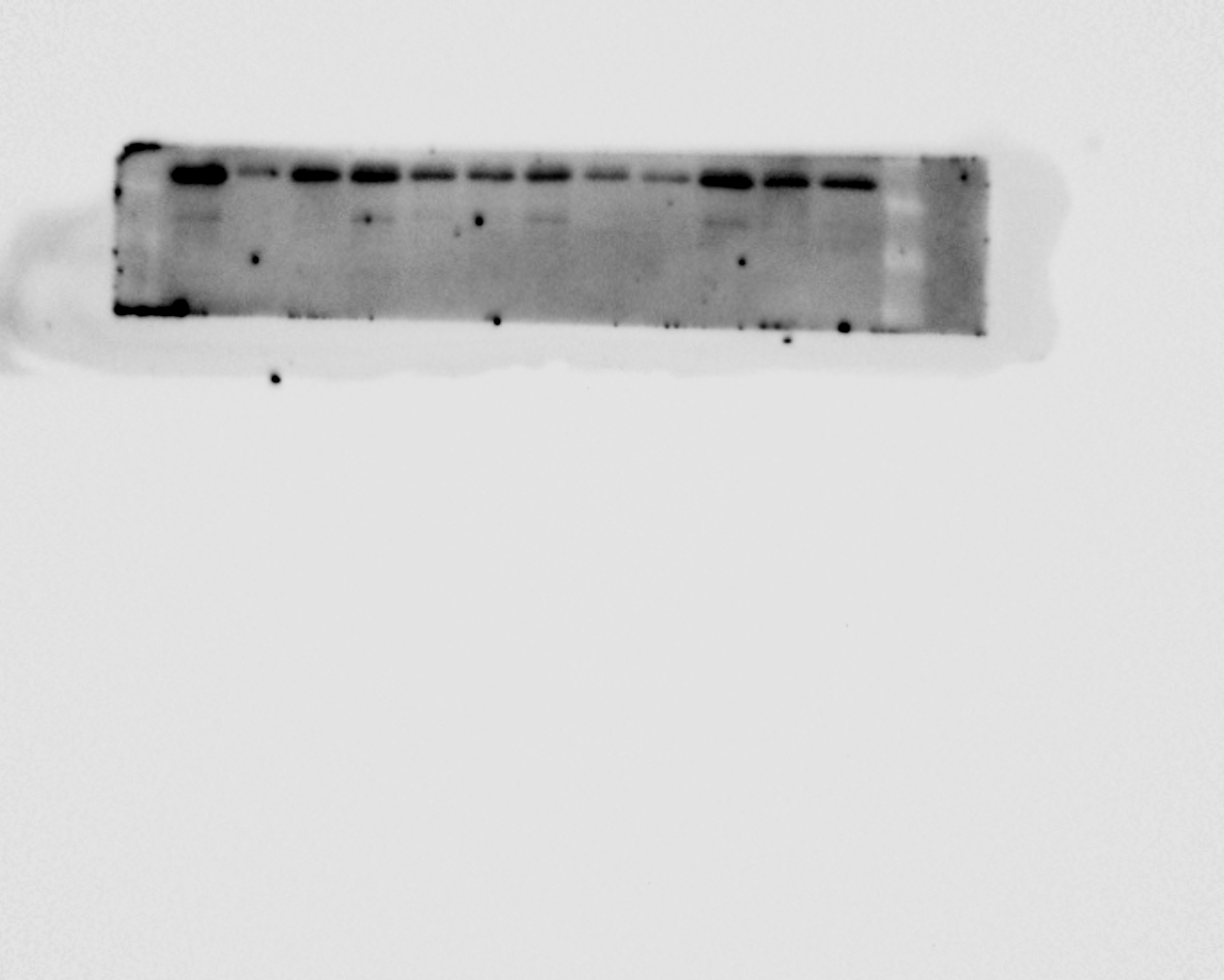

Supplement: Supplementary file 5 [file DataSheet4.ZIP › cas90sea_1(Chemiluminescence).tif]

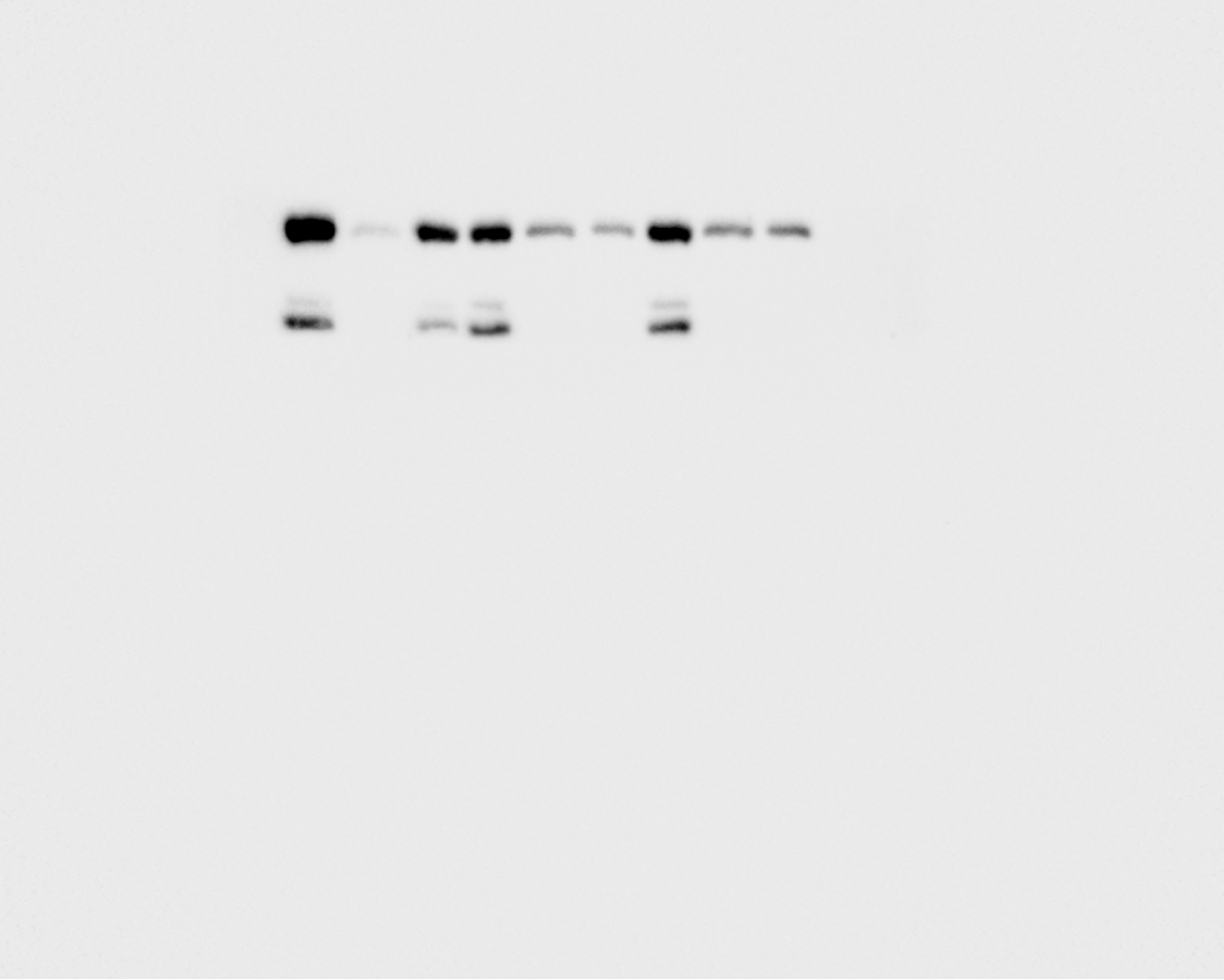

Supplement: Supplementary file 5 [file DataSheet4.ZIP › cas9-16_3(Chemiluminescence).tif]

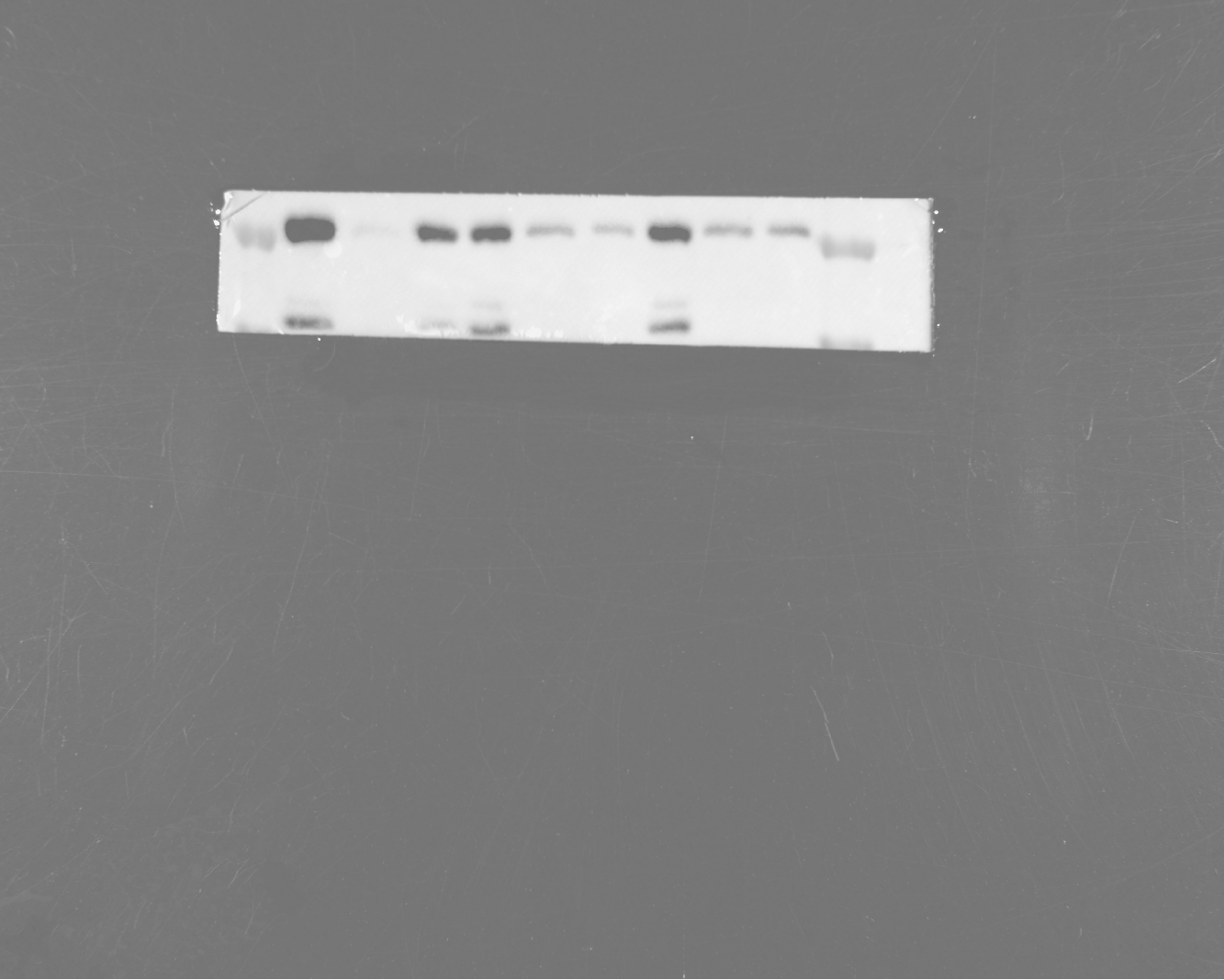

Supplement: Supplementary file 5 [file DataSheet4.ZIP › cas9-16_3(Composite).tif]

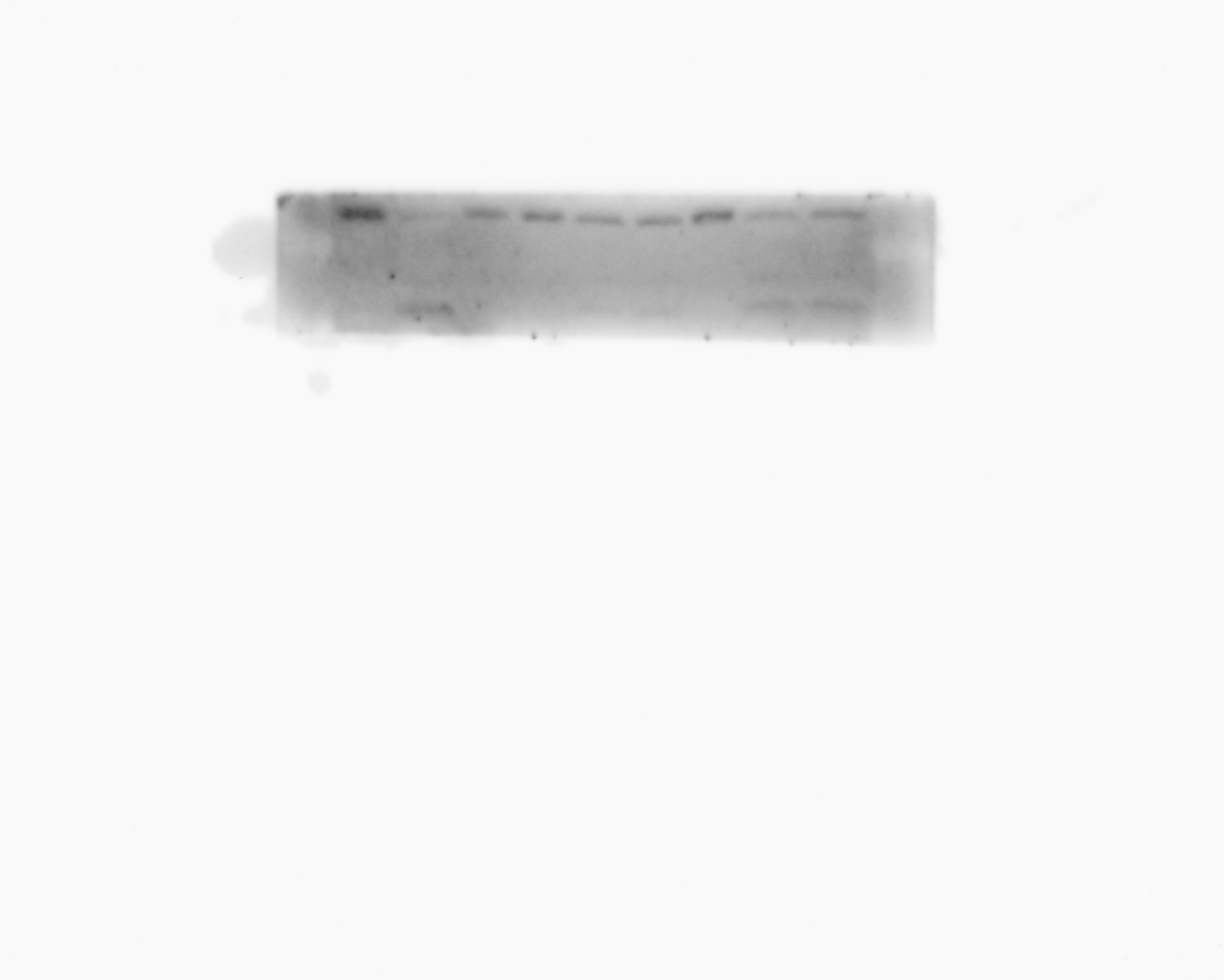

Supplement: Supplementary file 5 [file DataSheet4.ZIP › cas9-3_1(Chemiluminescence).tif]

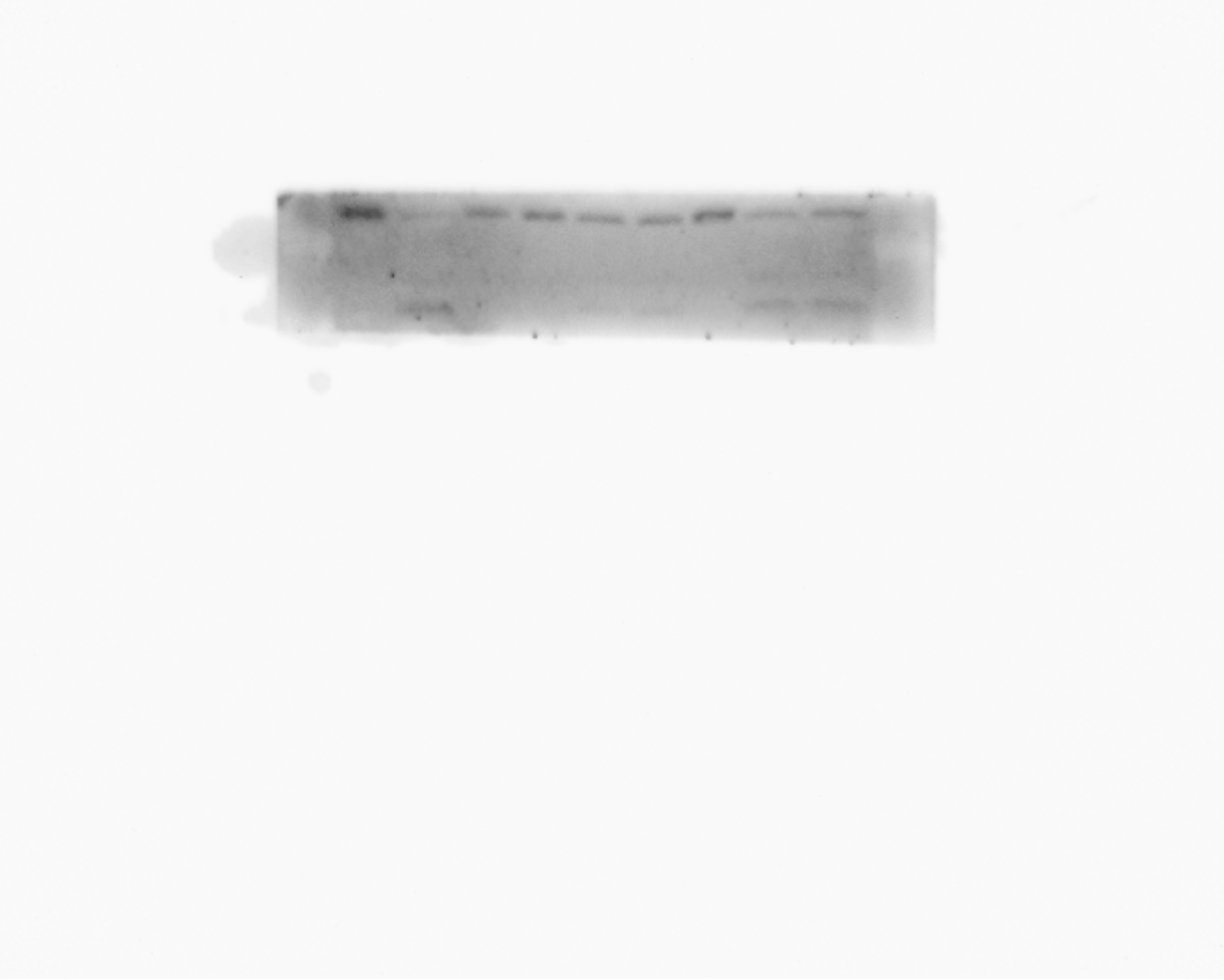

Supplement: Supplementary file 5 [file DataSheet4.ZIP › cas9-3_7(Chemiluminescence).tif]

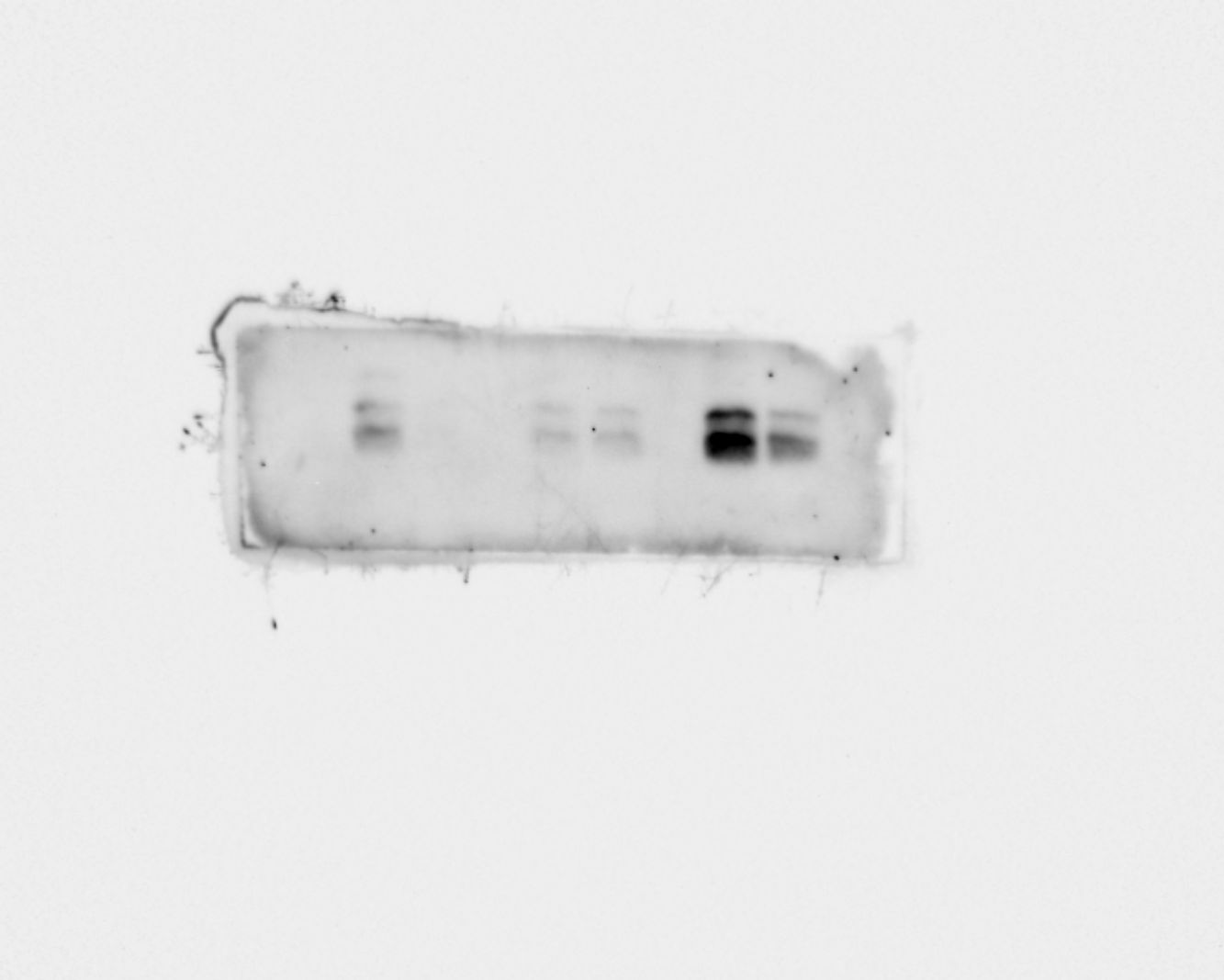

Supplement: Supplementary file 5 [file DataSheet4.ZIP › cc3-1.tif]

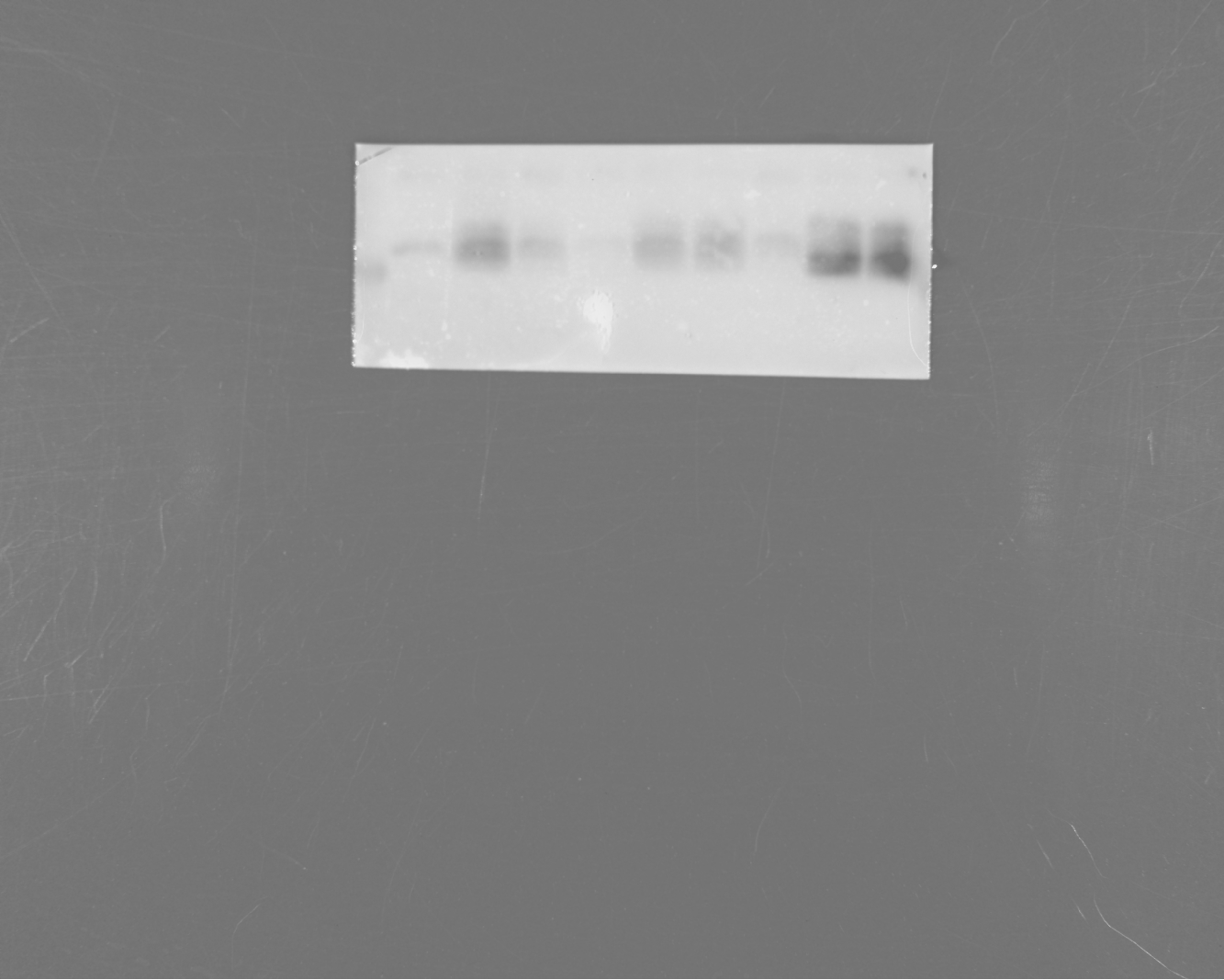

Supplement: Supplementary file 5 [file DataSheet4.ZIP › cc3-2 (1).tif]

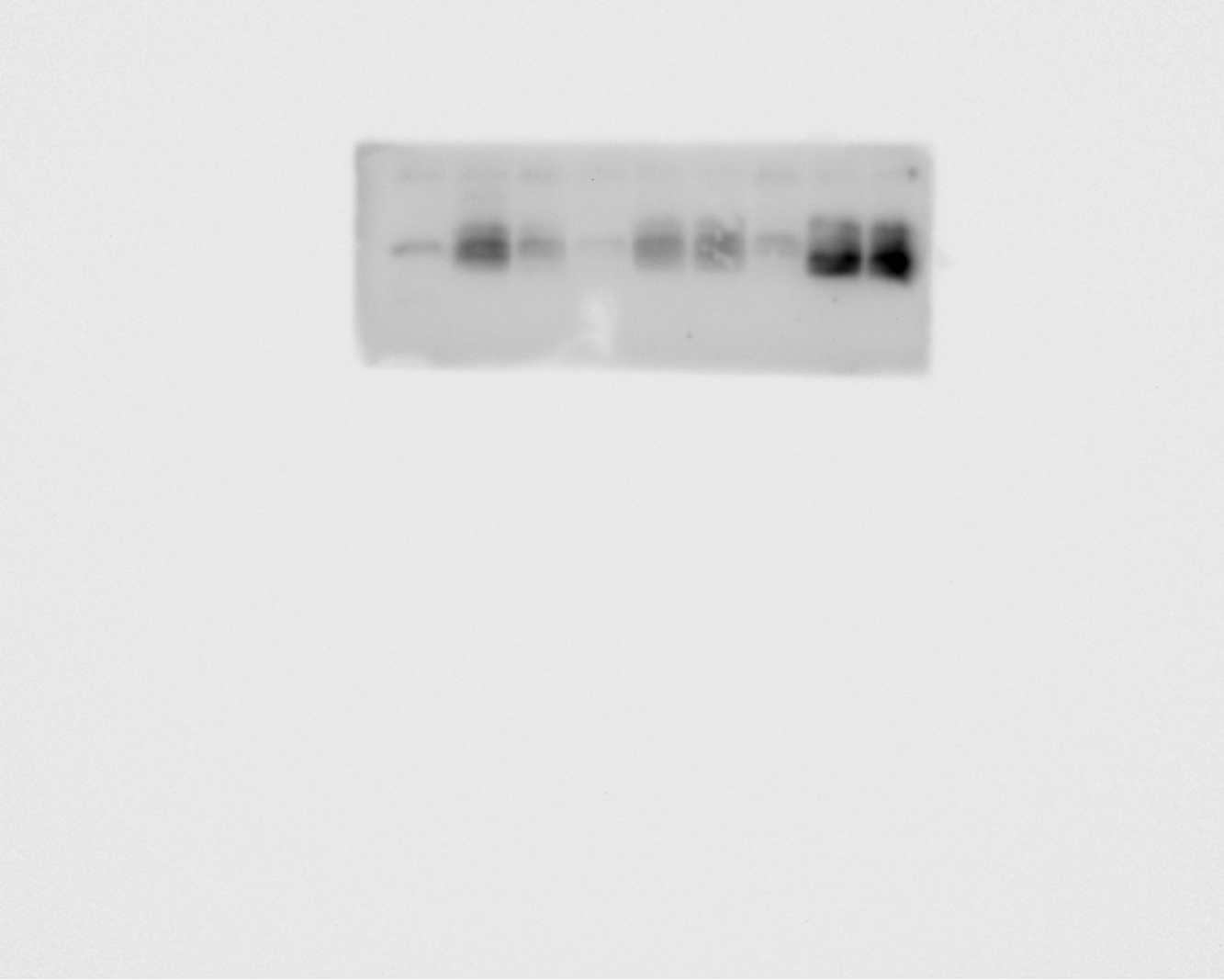

Supplement: Supplementary file 5 [file DataSheet4.ZIP › cc3-2 (2).tif]

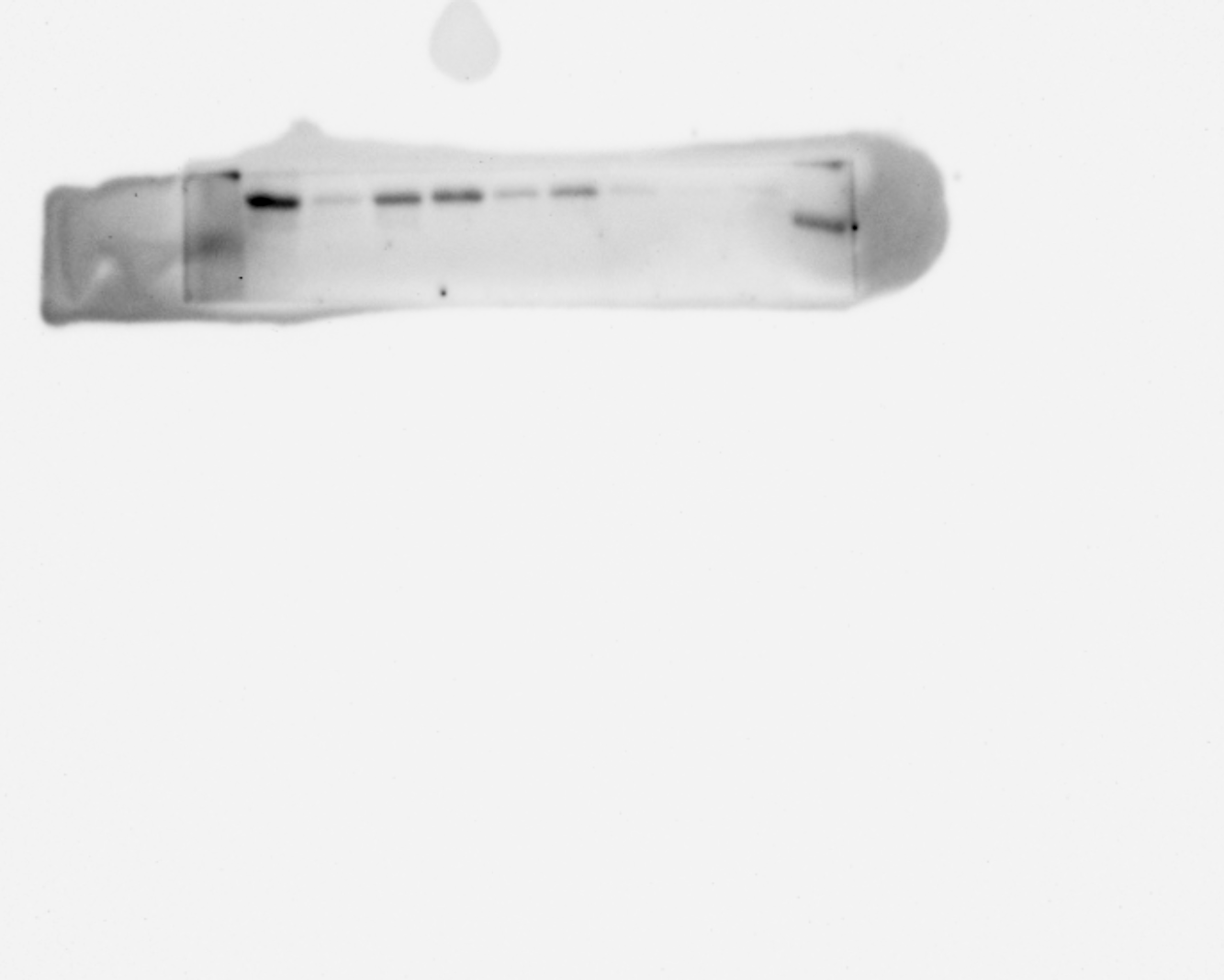

Supplement: Supplementary file 5 [file DataSheet4.ZIP › cycin_1(Chemiluminescence).tif]

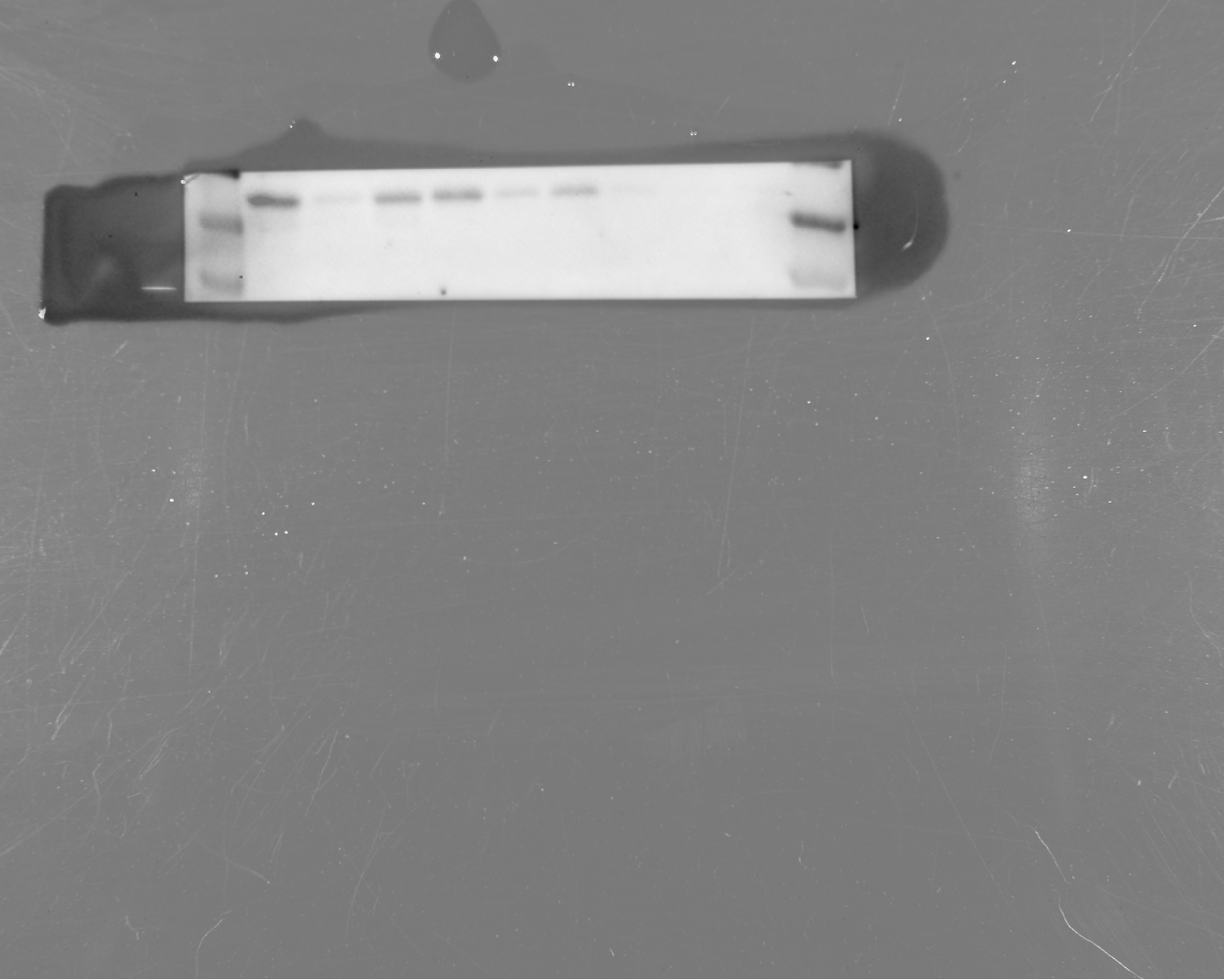

Supplement: Supplementary file 5 [file DataSheet4.ZIP › cycin_3(Composite).tif]

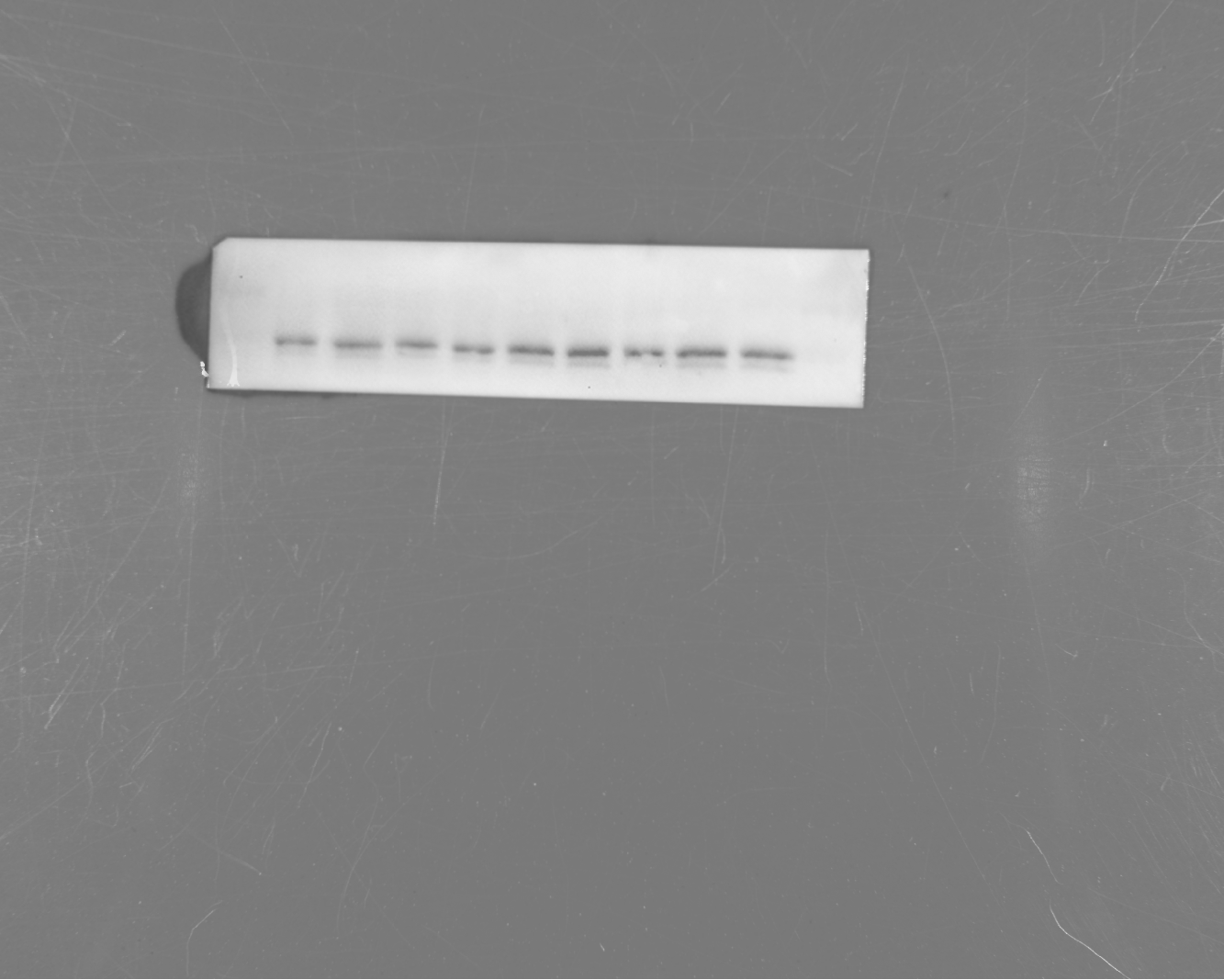

Supplement: Supplementary file 5 [file DataSheet4.ZIP › erk-2 (2).tif]

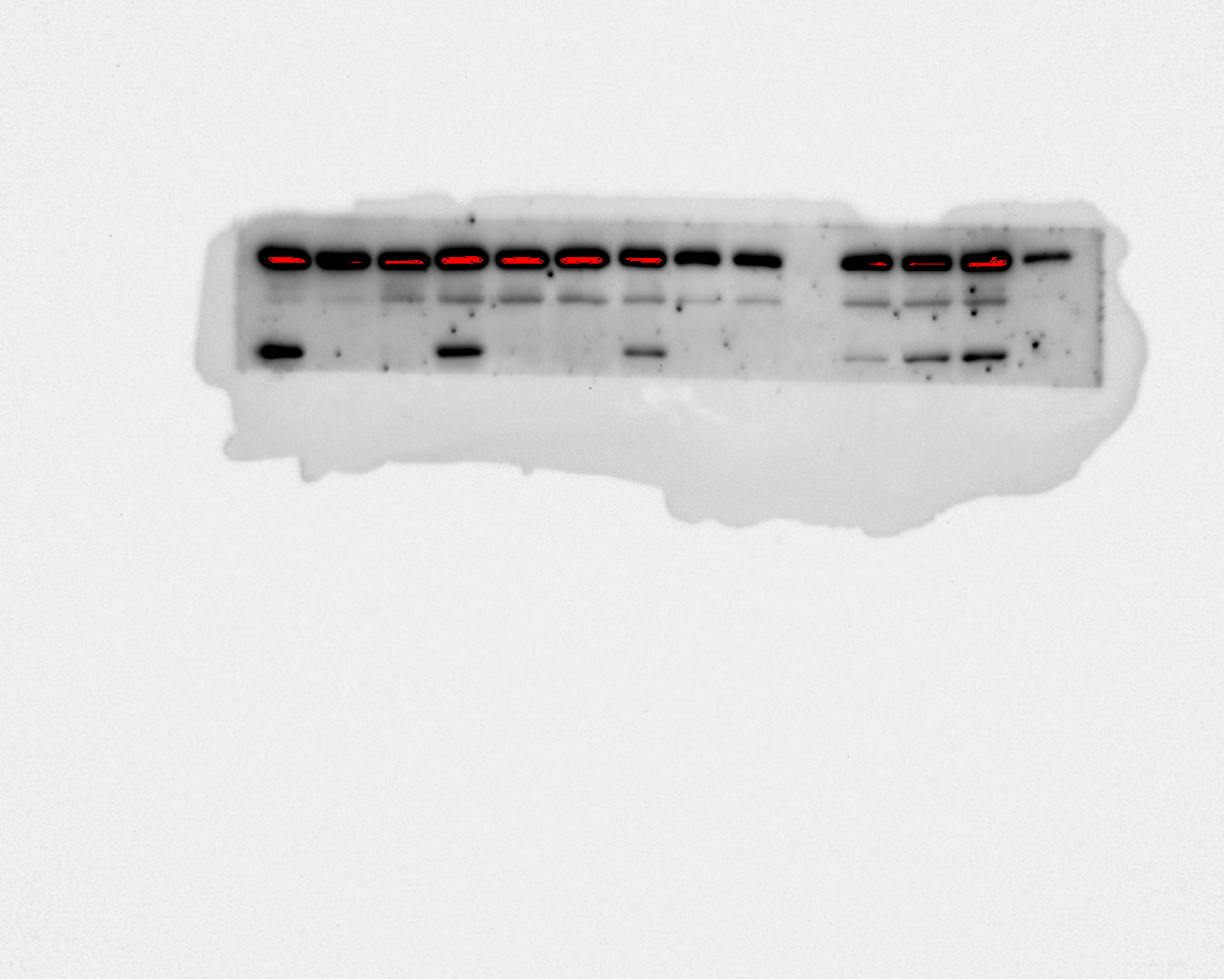

Supplement: Supplementary file 6 [file DataSheet1.ZIP › homo-ST/anorectal 2021-02-07 11h08m42s(Chemiluminescence).tif]

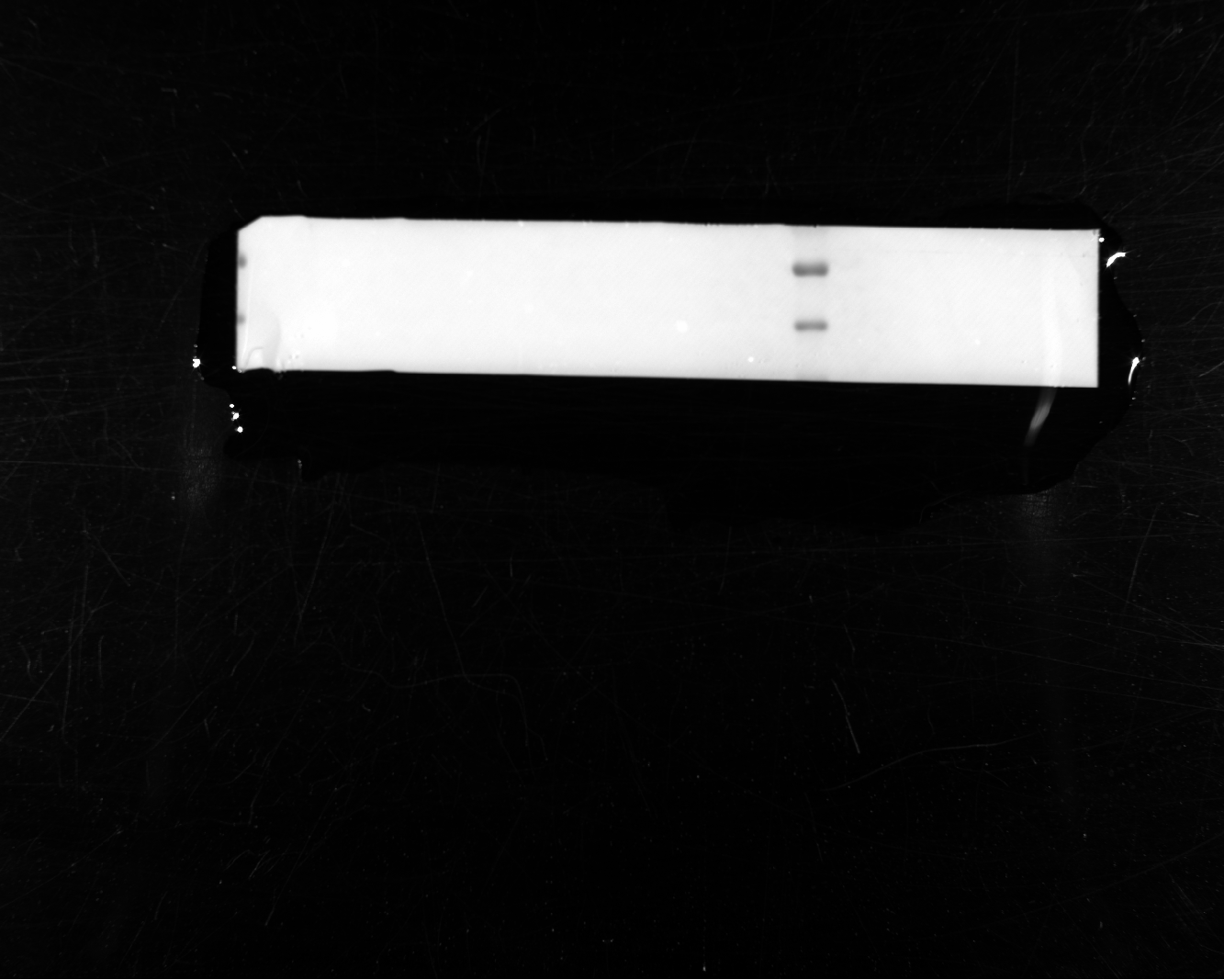

Supplement: Supplementary file 6 [file DataSheet1.ZIP › homo-ST/anorectal 2021-02-07 11h08m42s(Colorimetric).tif]

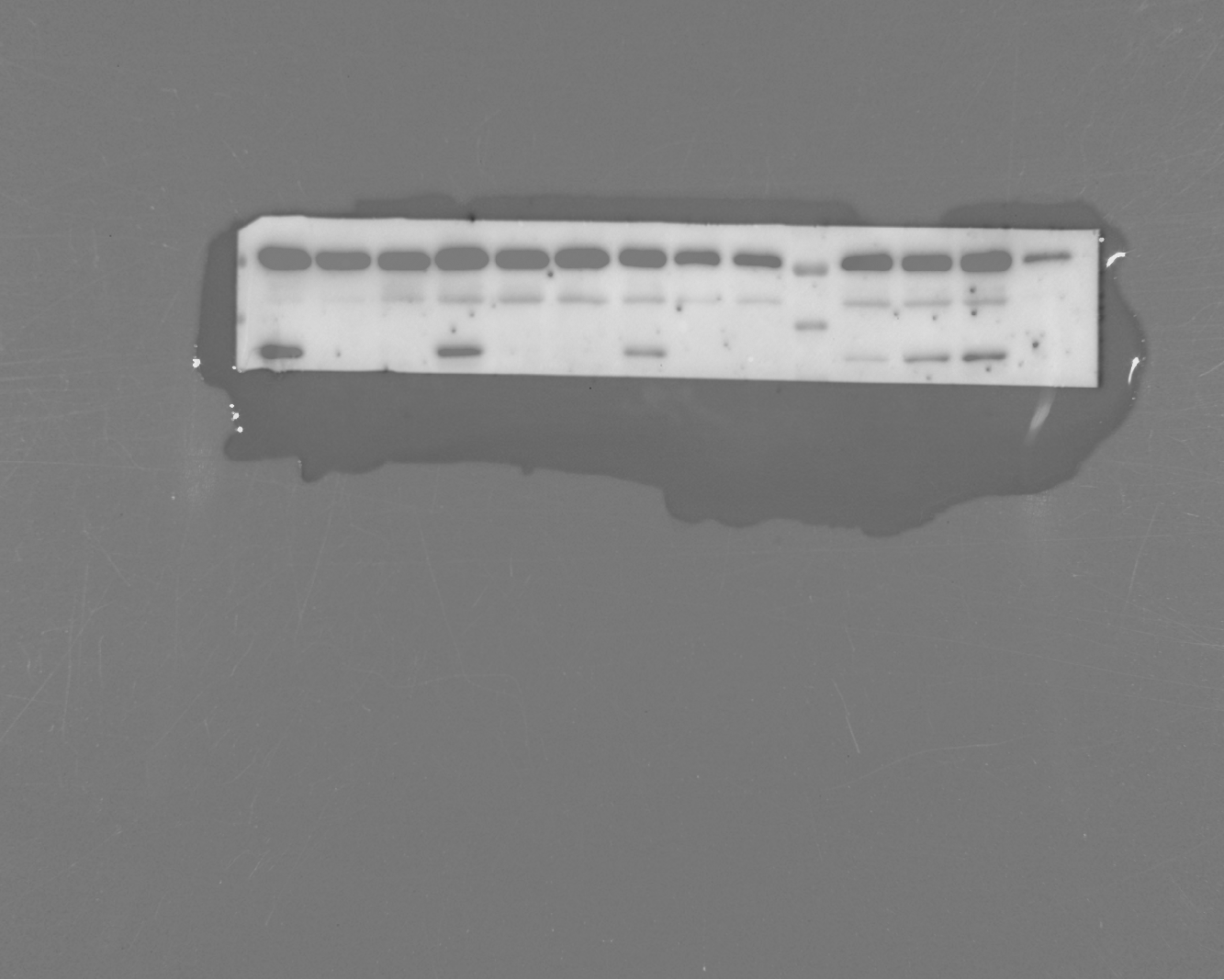

Supplement: Supplementary file 6 [file DataSheet1.ZIP › homo-ST/anorectal 2021-02-07 11h08m42s(Composite).tif]

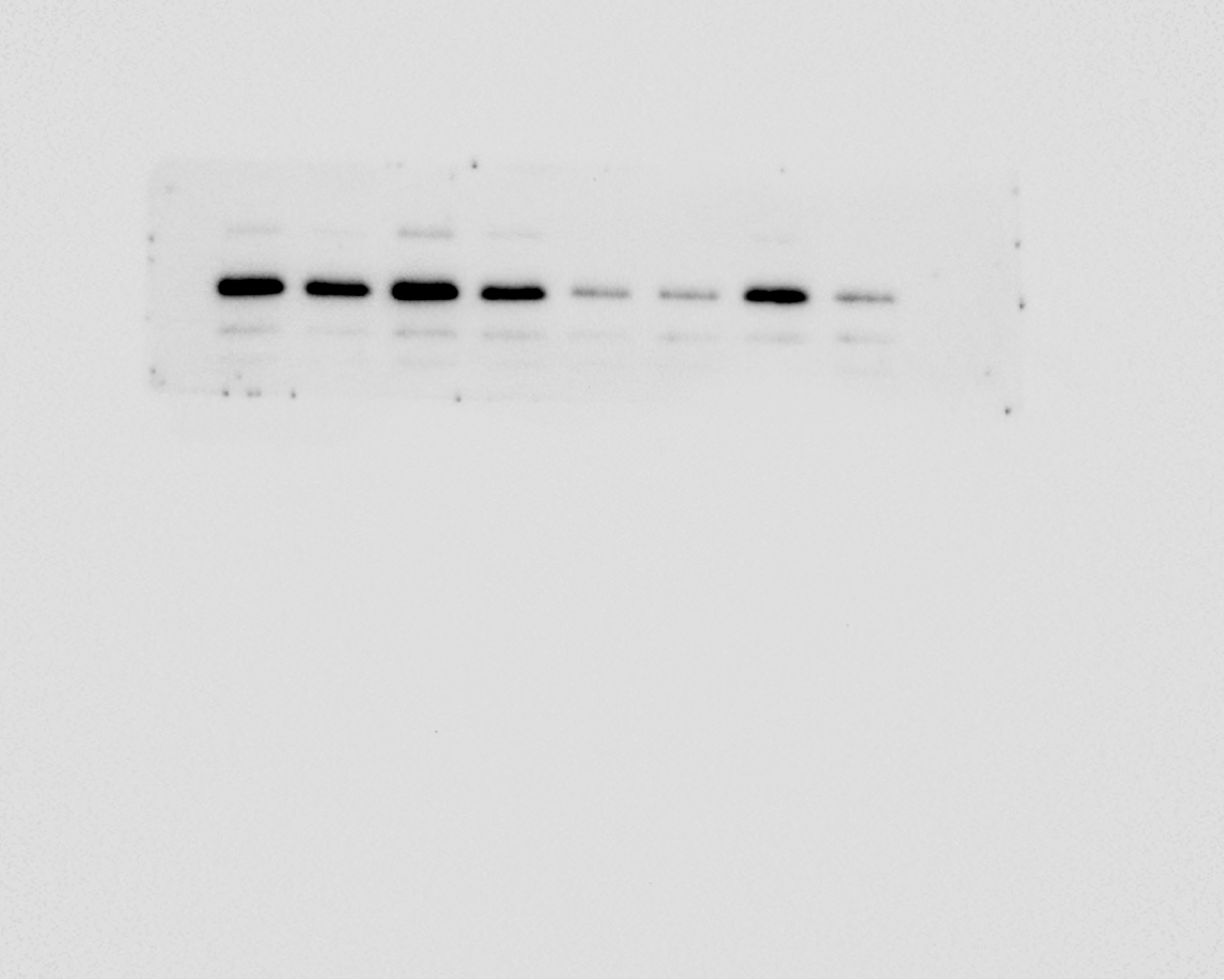

Supplement: Supplementary file 6 [file DataSheet1.ZIP › homo-ST/bcl2stho_3(Chemiluminescence).tif]

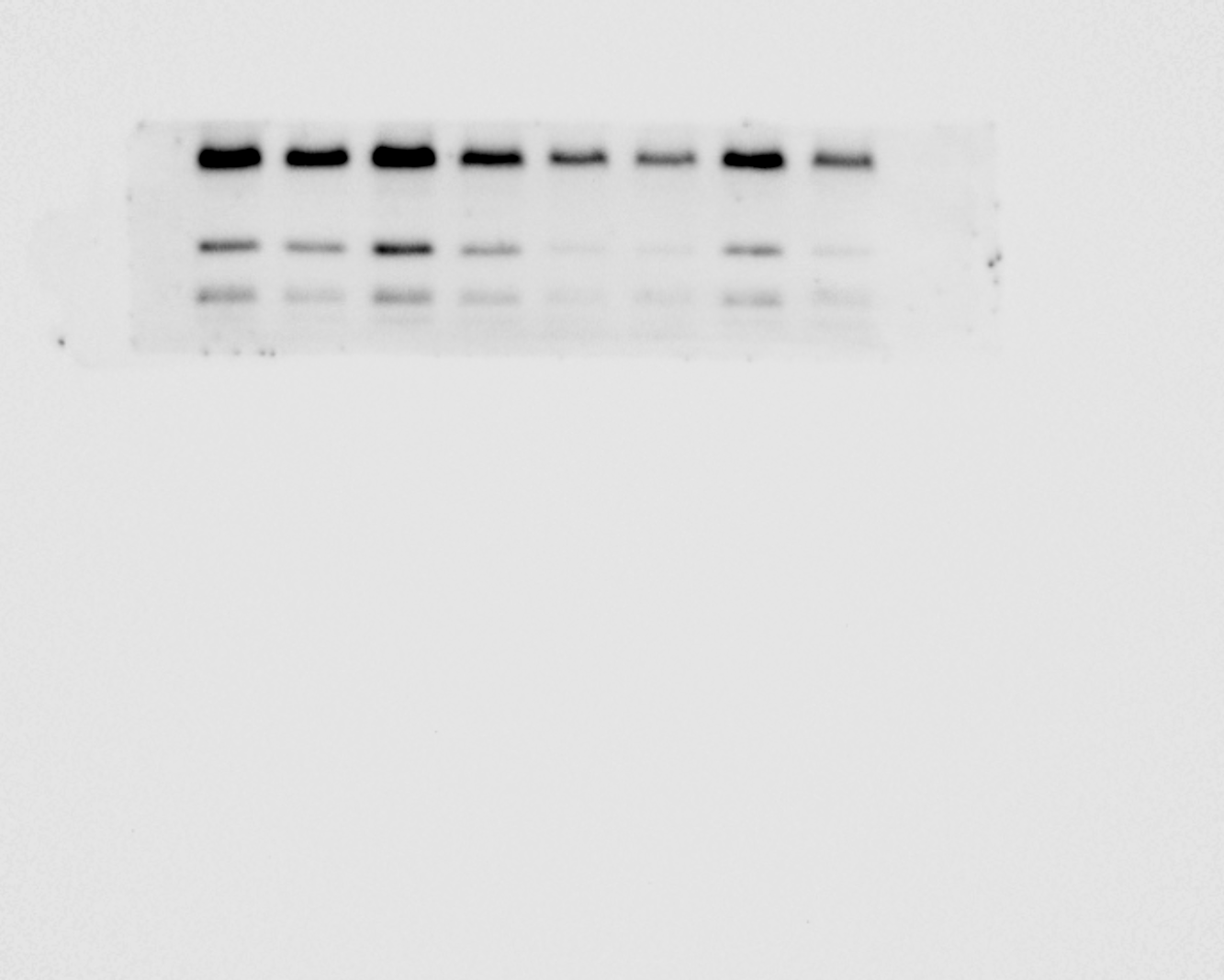

Supplement: Supplementary file 6 [file DataSheet1.ZIP › homo-ST/cas3stho-2_1(Chemiluminescence).tif]

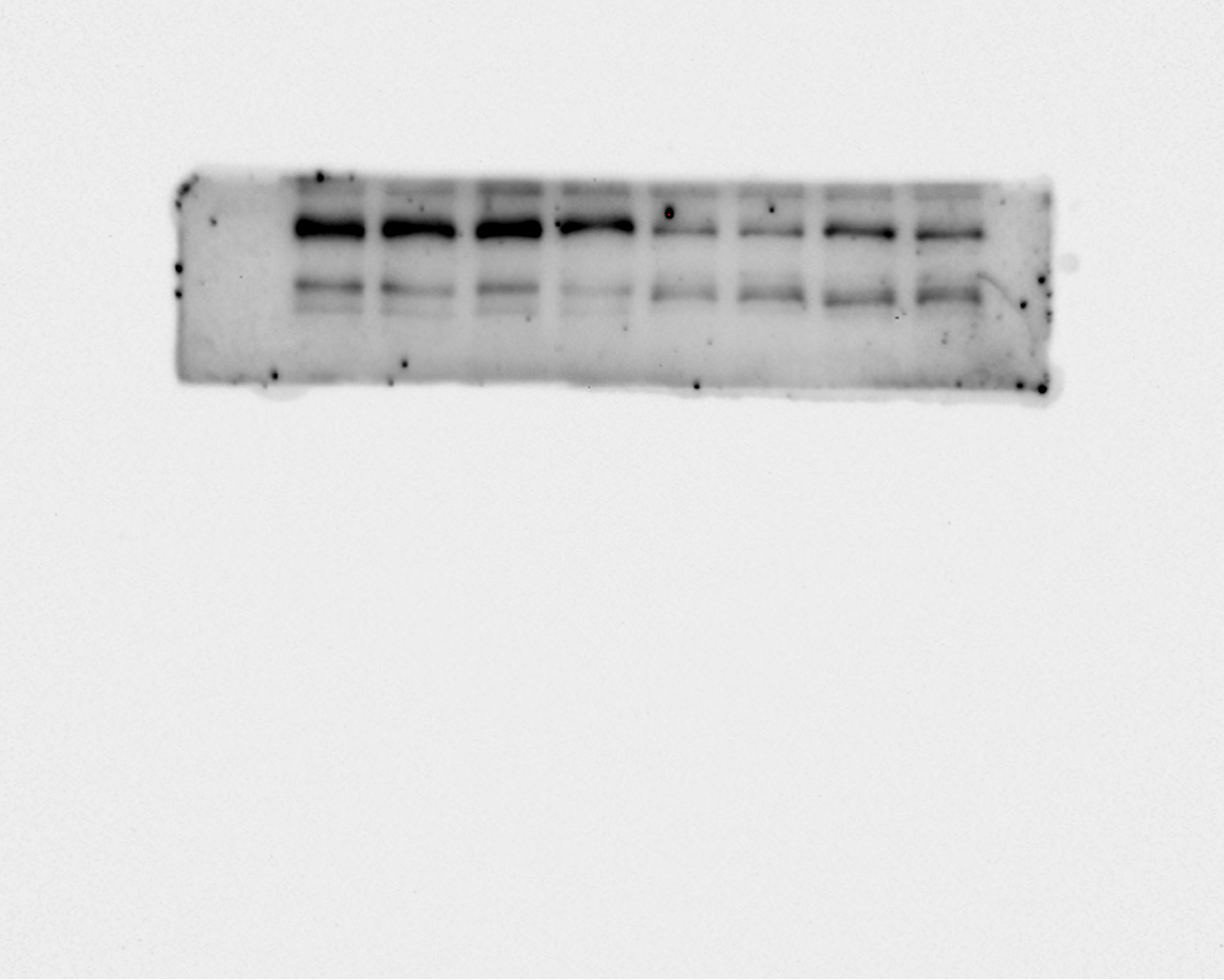

Supplement: Supplementary file 6 [file DataSheet1.ZIP › homo-ST/cas9stho-3_7(Chemiluminescence).tif]

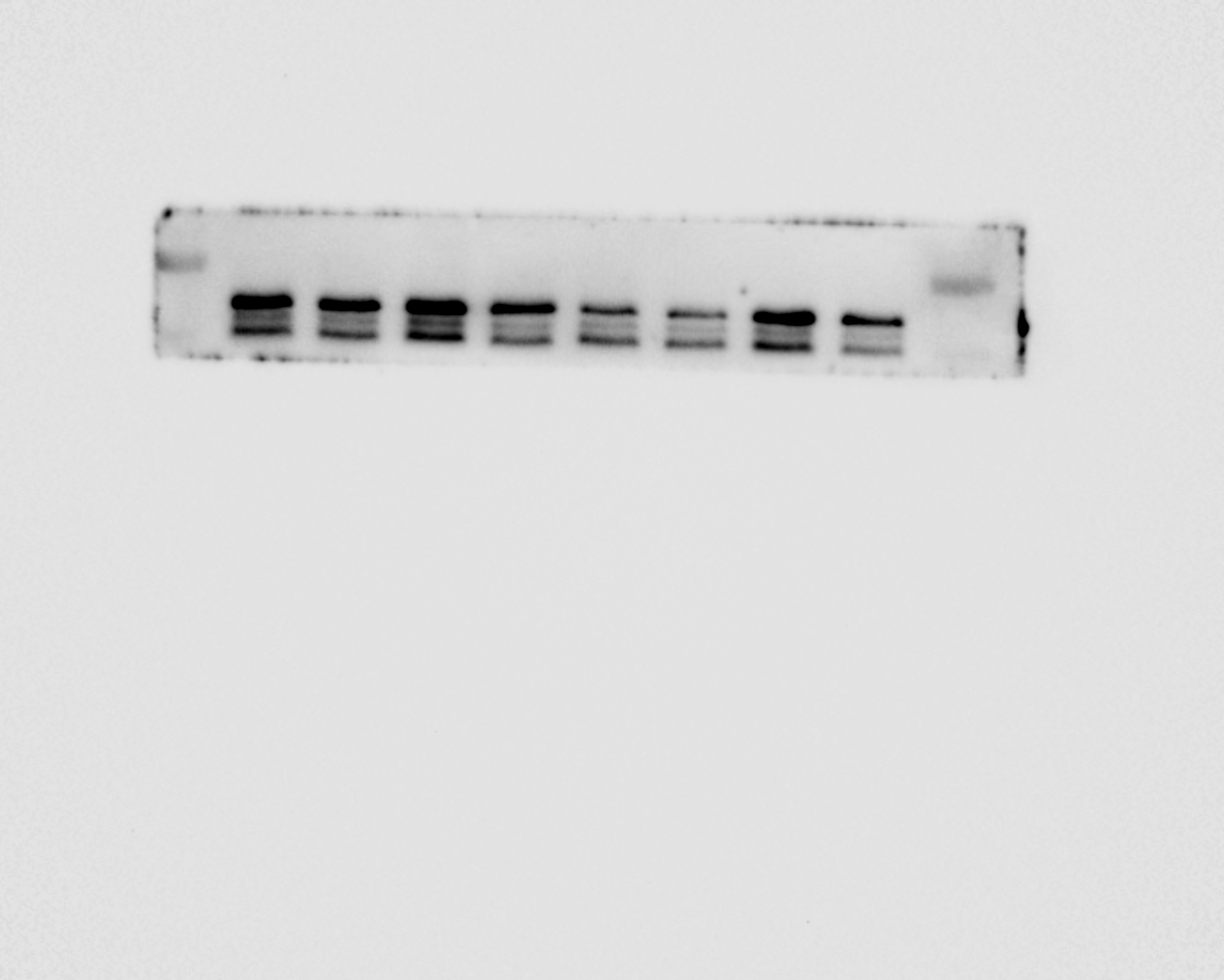

Supplement: Supplementary file 6 [file DataSheet1.ZIP › homo-ST/erksthomo_2(Chemiluminescence).tif]

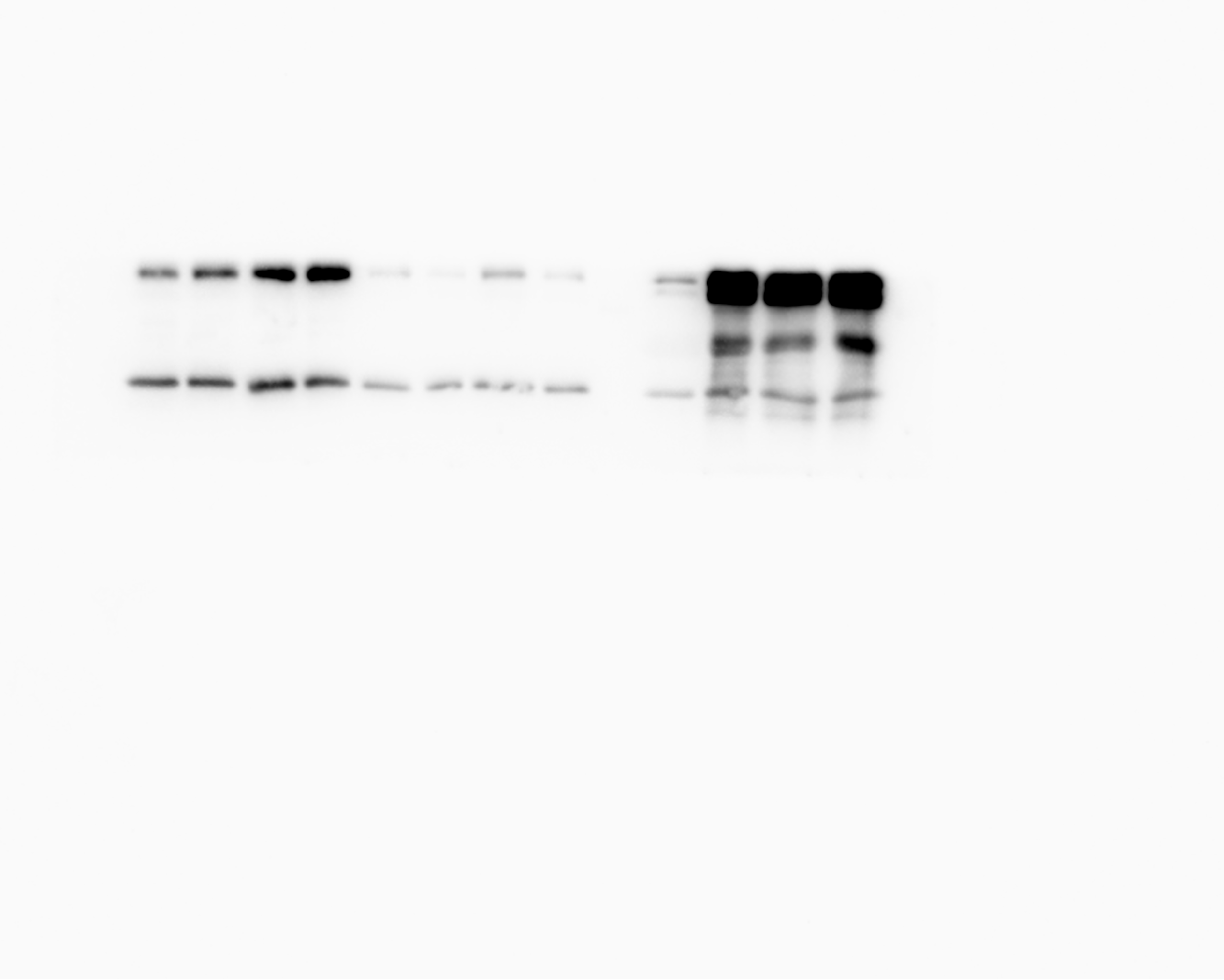

Supplement: Supplementary file 6 [file DataSheet1.ZIP › homo-ST/left-cas9-right-cc9-rat_2(Chemiluminescence).tif]

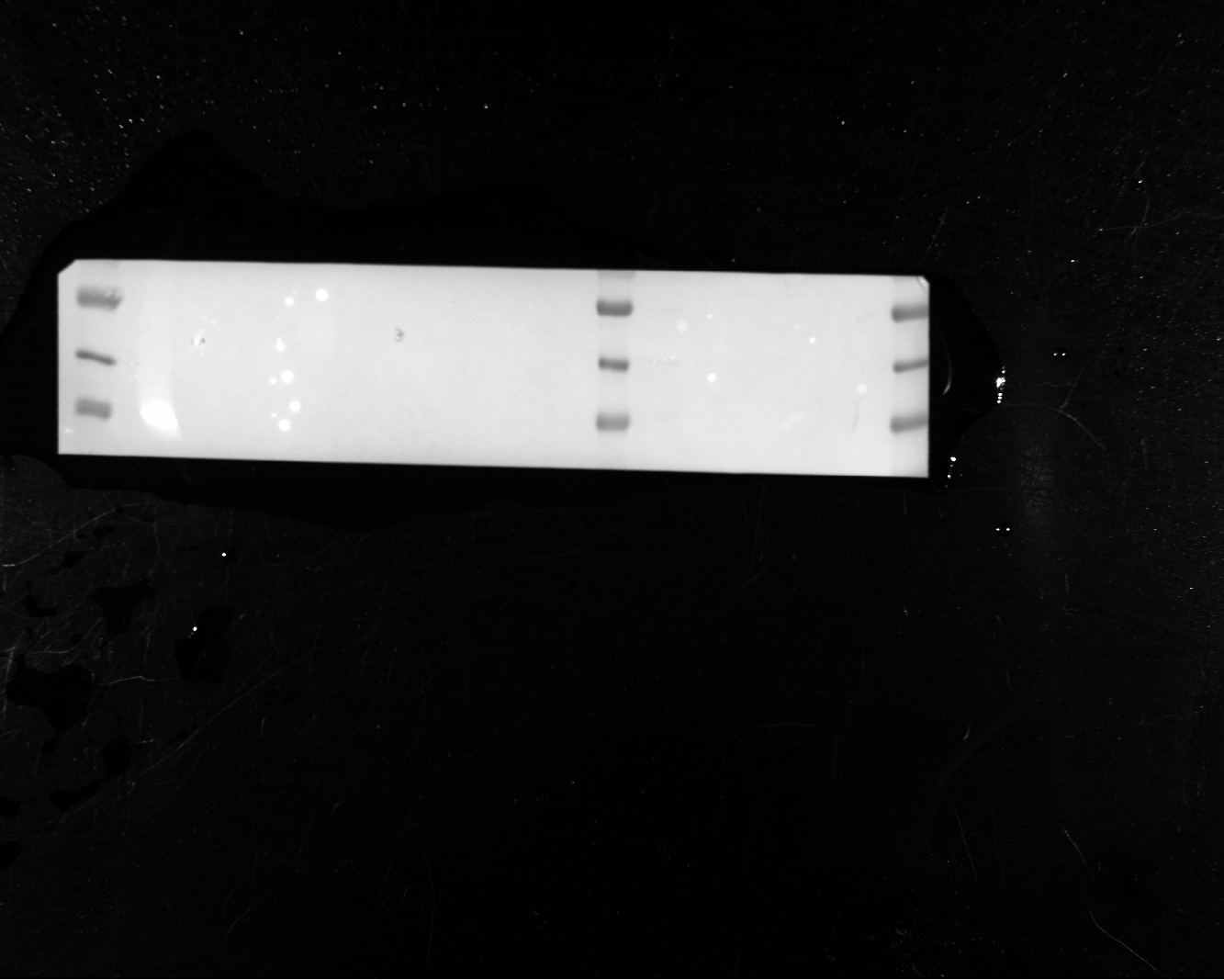

Supplement: Supplementary file 6 [file DataSheet1.ZIP › homo-ST/left-cas9-right-cc9-rat_2(Colorimetric).tif]

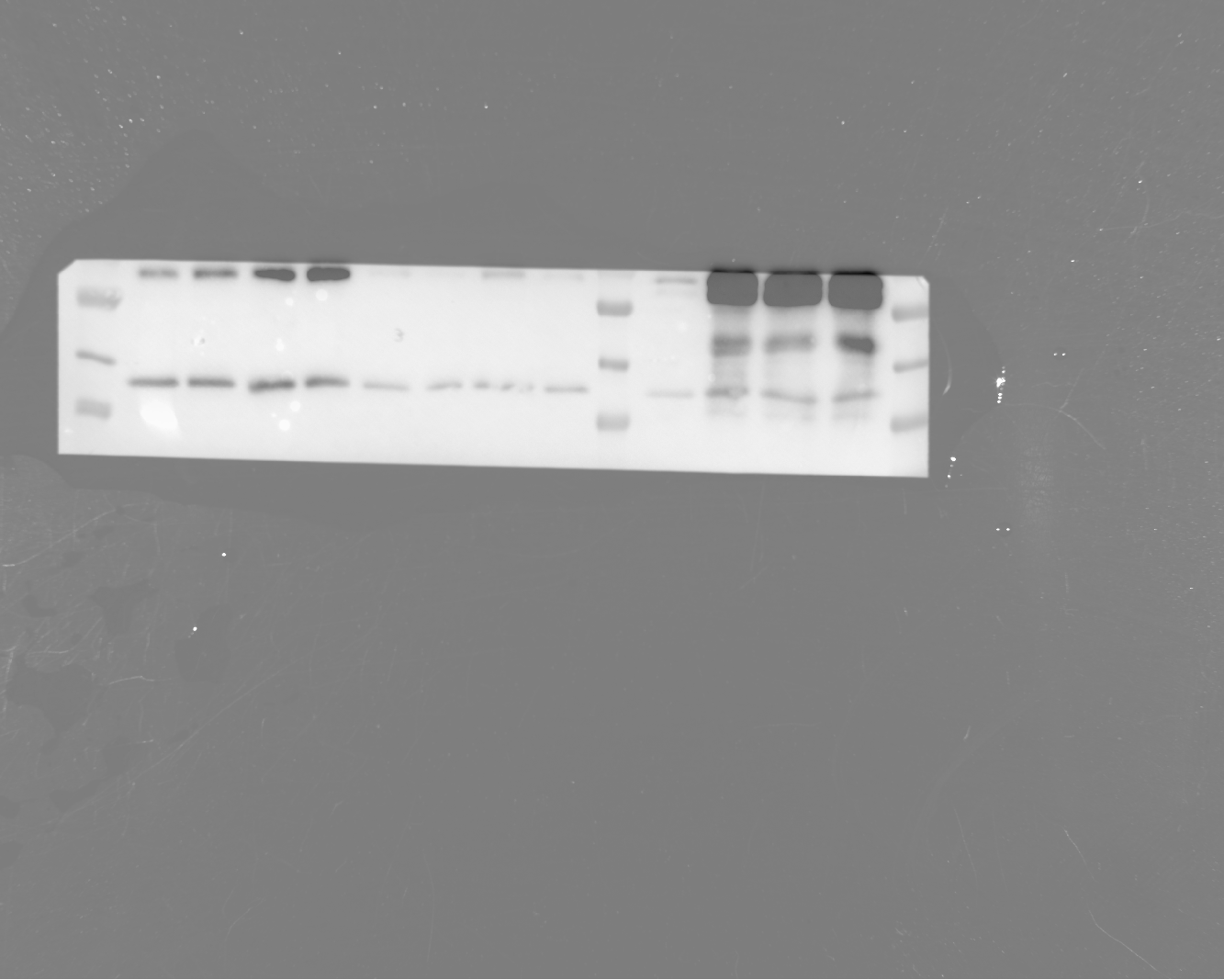

Supplement: Supplementary file 6 [file DataSheet1.ZIP › homo-ST/left-cas9-right-cc9-rat_2(Composite).tif]

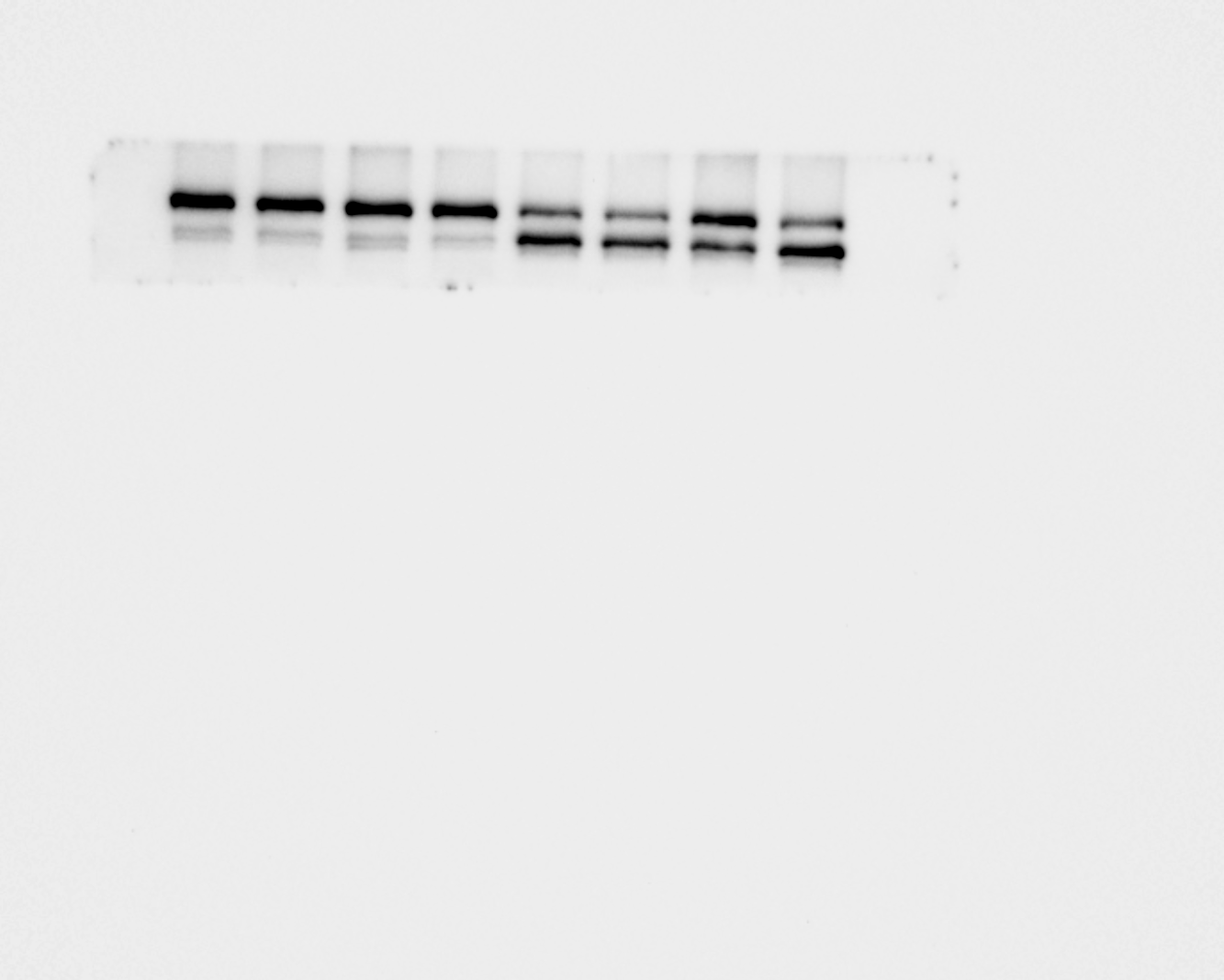

Supplement: Supplementary file 6 [file DataSheet1.ZIP › homo-ST/parp.tif]

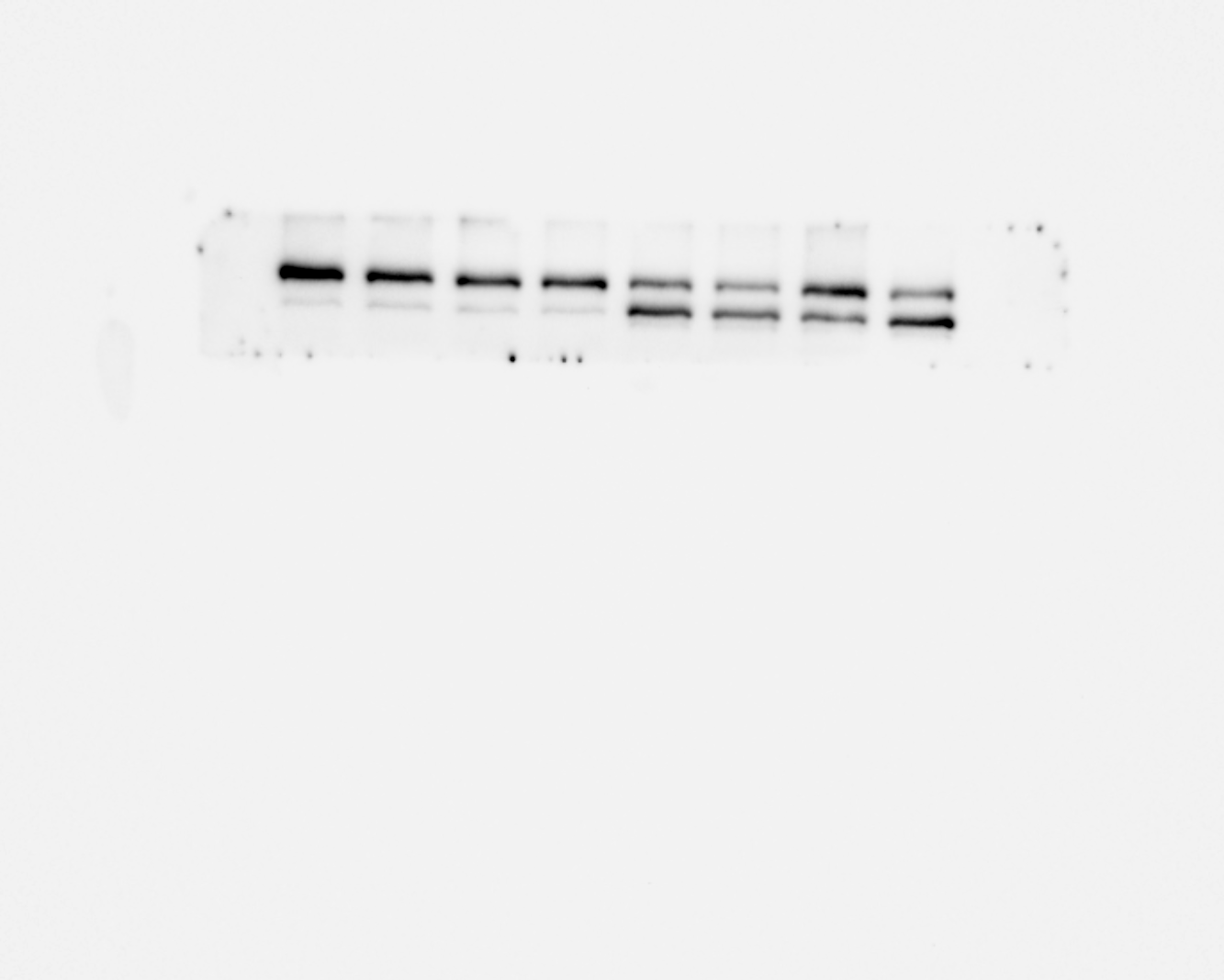

Supplement: Supplementary file 6 [file DataSheet1.ZIP › homo-ST/parpstho_1(Chemiluminescence).tif]

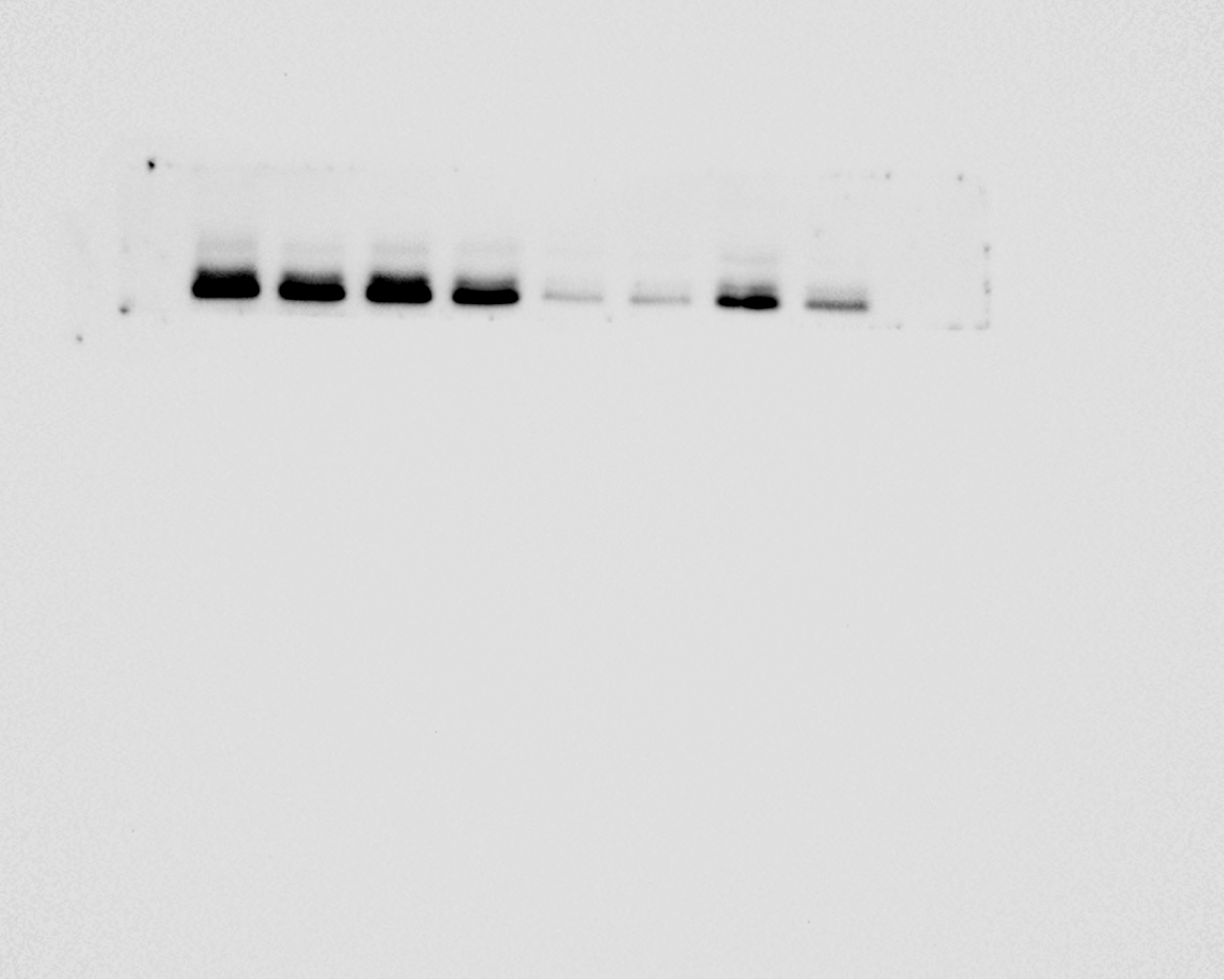

Supplement: Supplementary file 6 [file DataSheet1.ZIP › homo-ST/perkstho_3(Chemiluminescence).tif]

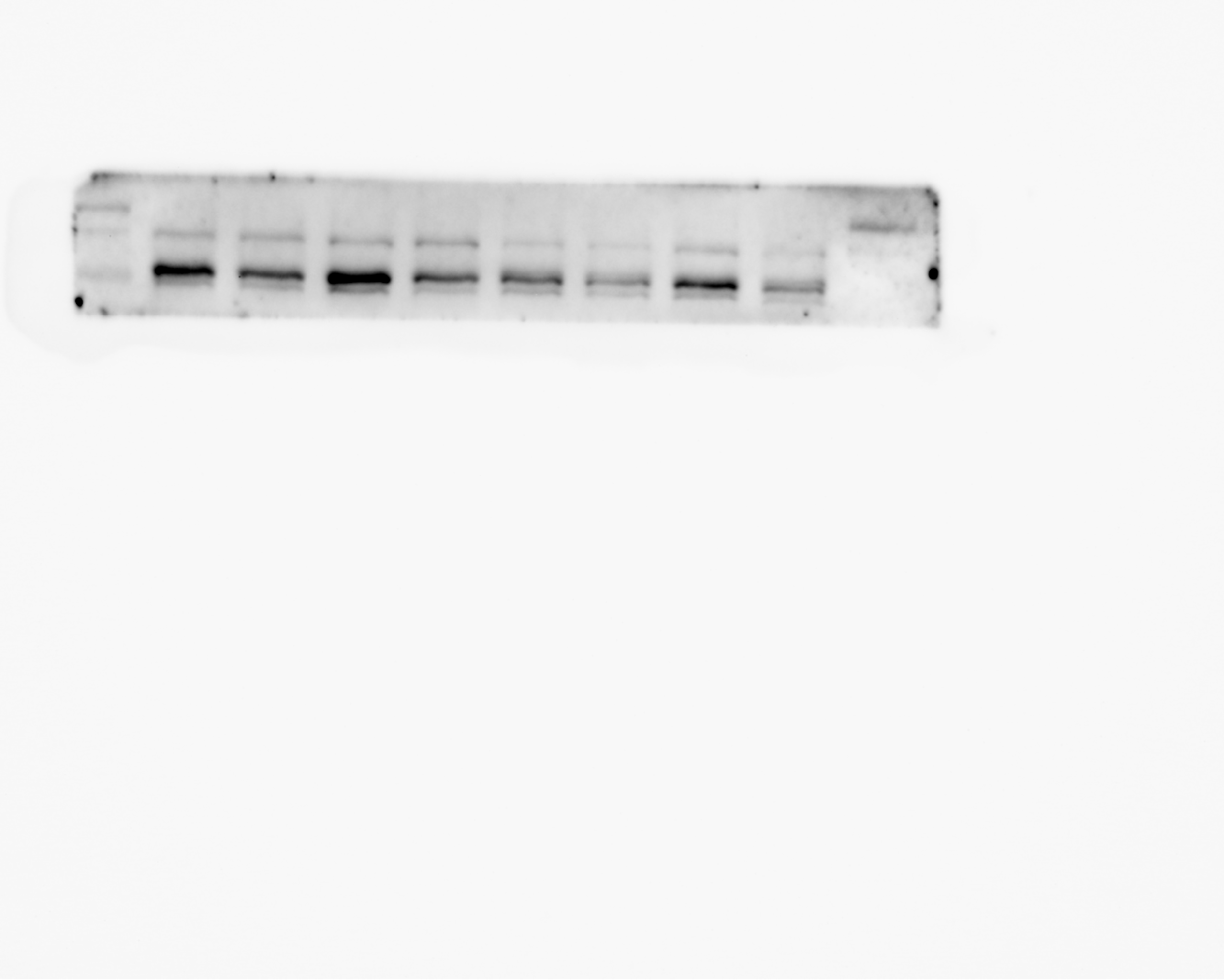

Supplement: Supplementary file 6 [file DataSheet1.ZIP › homo-ST/pstat3stho_3(Chemiluminescence).tif]

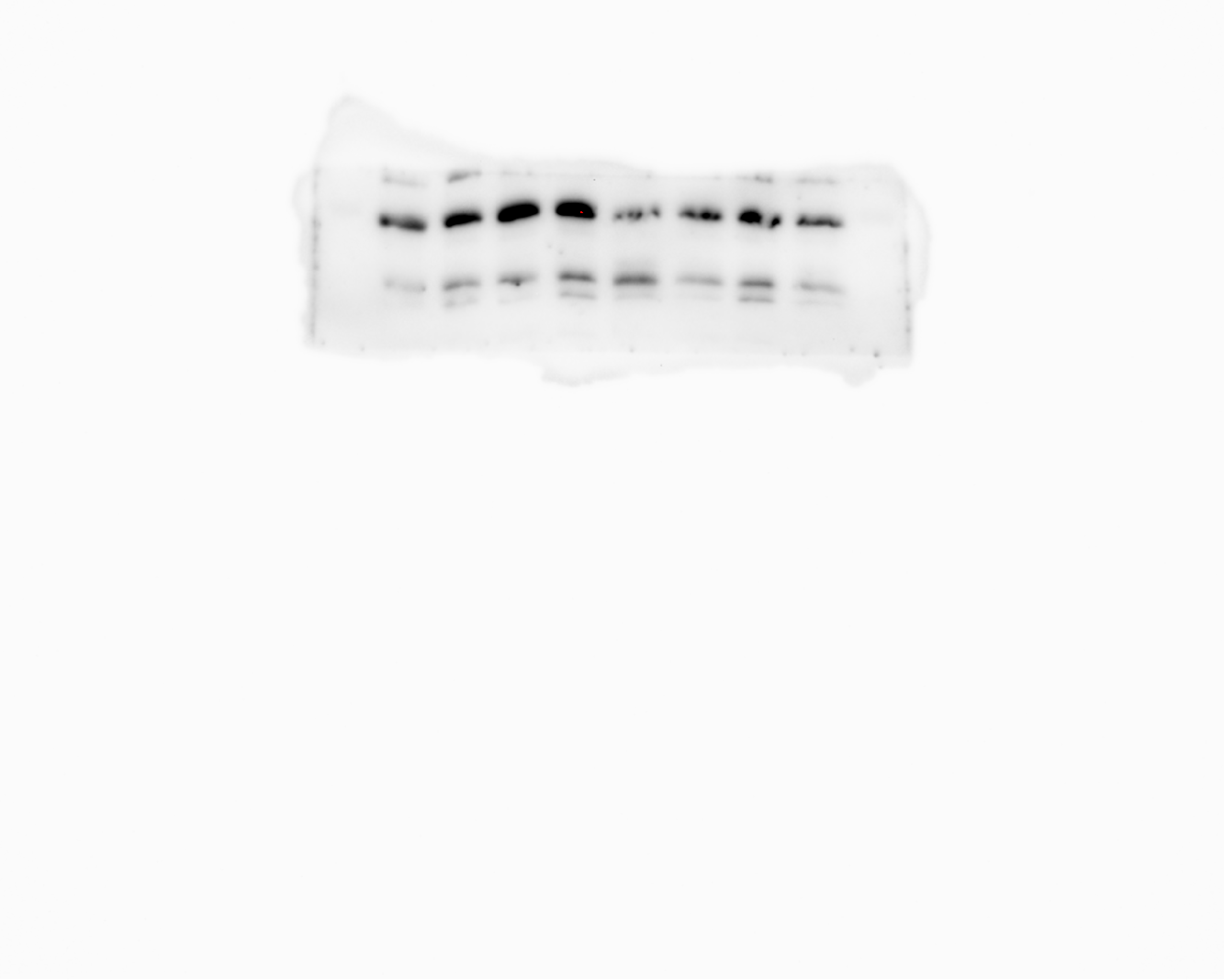

Supplement: Supplementary file 6 [file DataSheet1.ZIP › homo-ST/ratcas9_3(Chemiluminescence).tif]

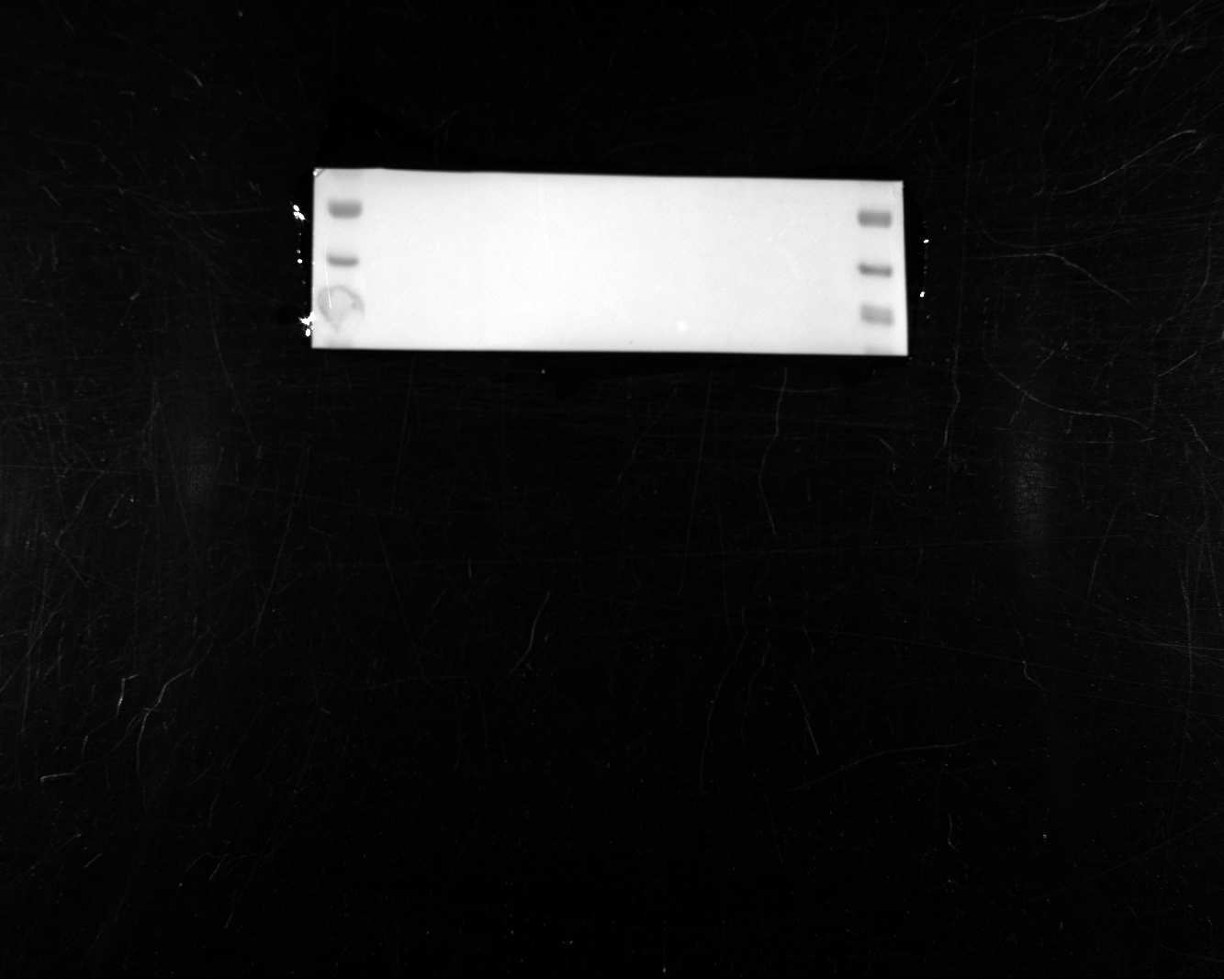

Supplement: Supplementary file 6 [file DataSheet1.ZIP › homo-ST/ratcas9_3(Colorimetric).tif]

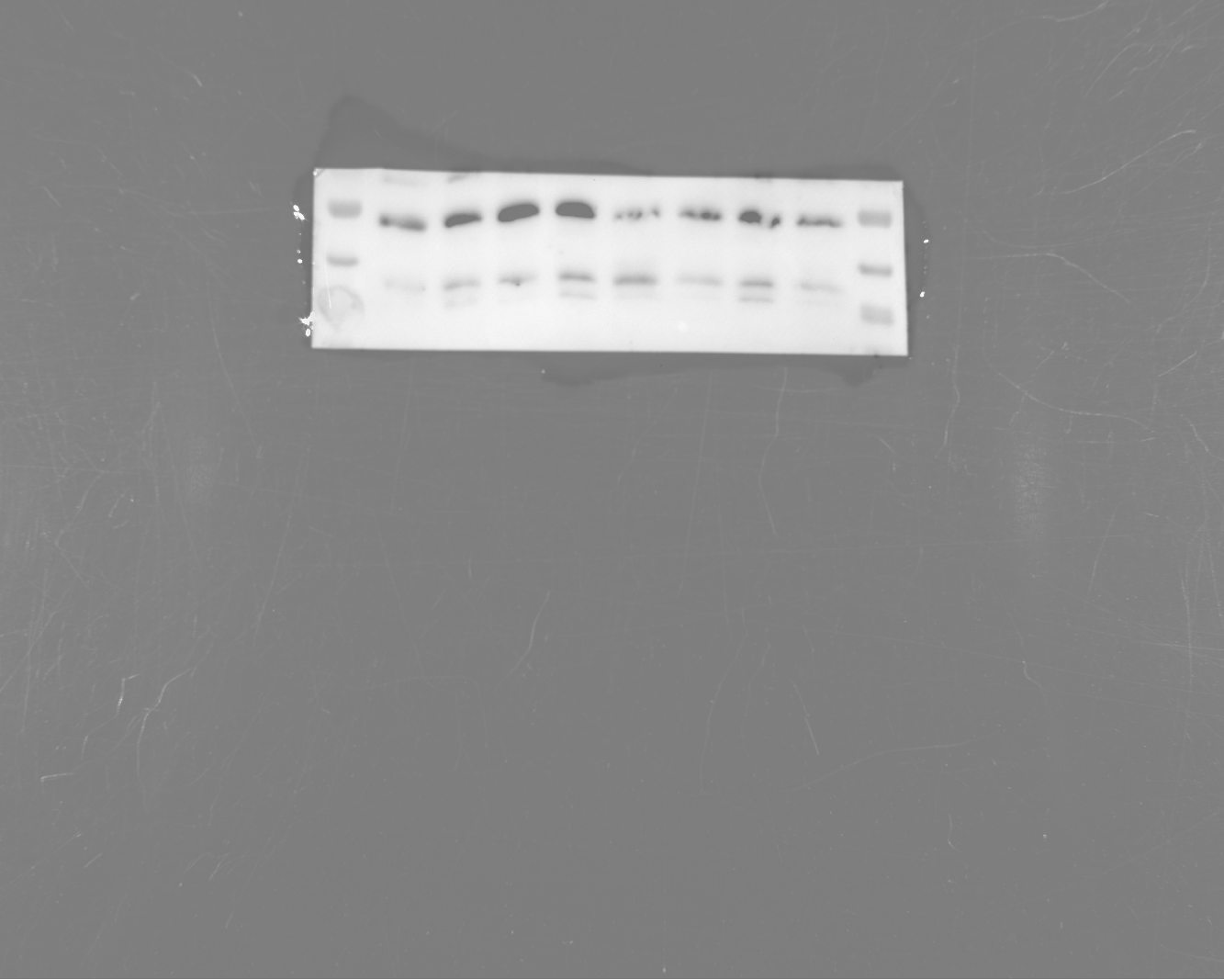

Supplement: Supplementary file 6 [file DataSheet1.ZIP › homo-ST/ratcas9_3(Composite).tif]

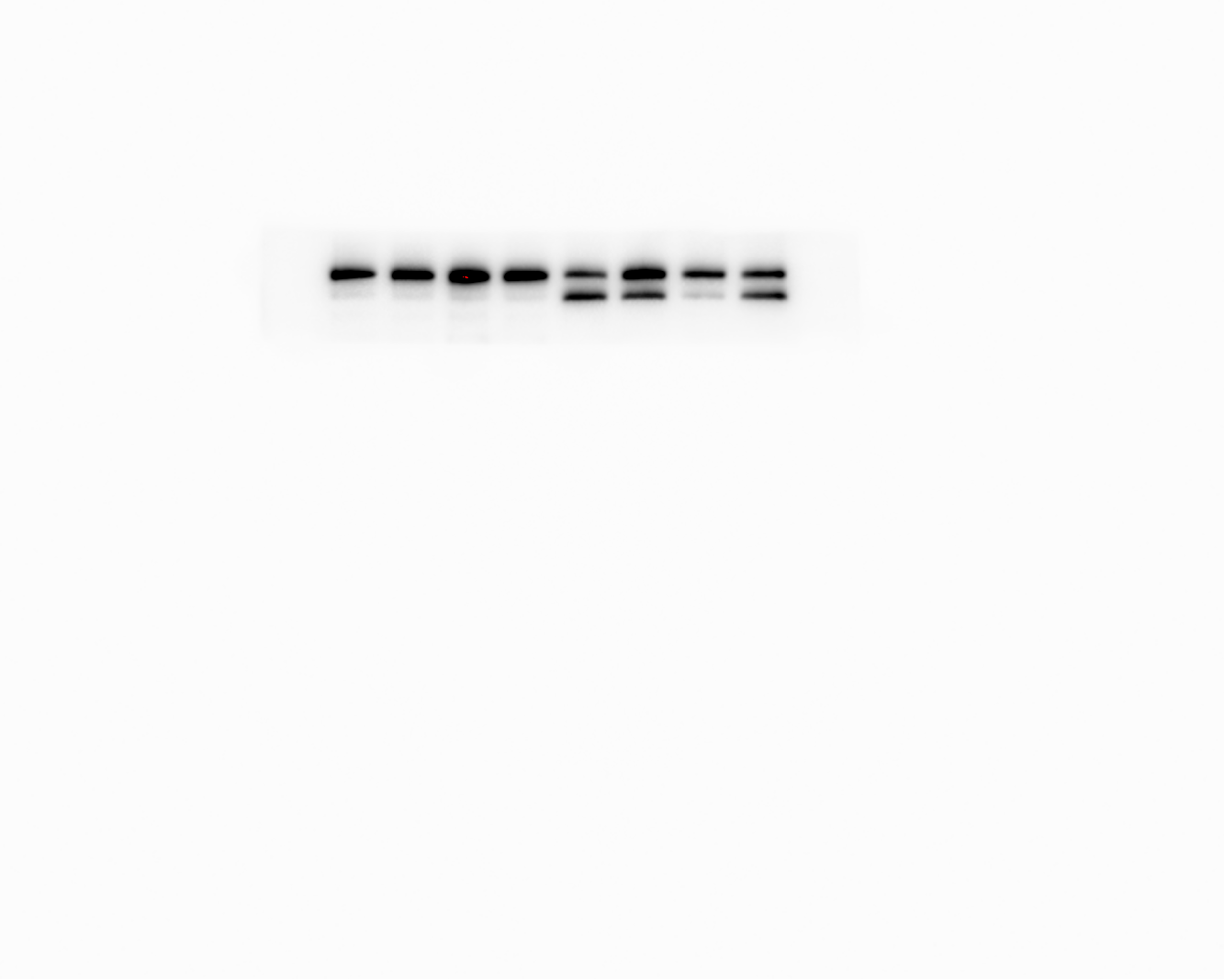

Supplement: Supplementary file 6 [file DataSheet1.ZIP › homo-ST/ratparp-2_4(Chemiluminescence).tif]

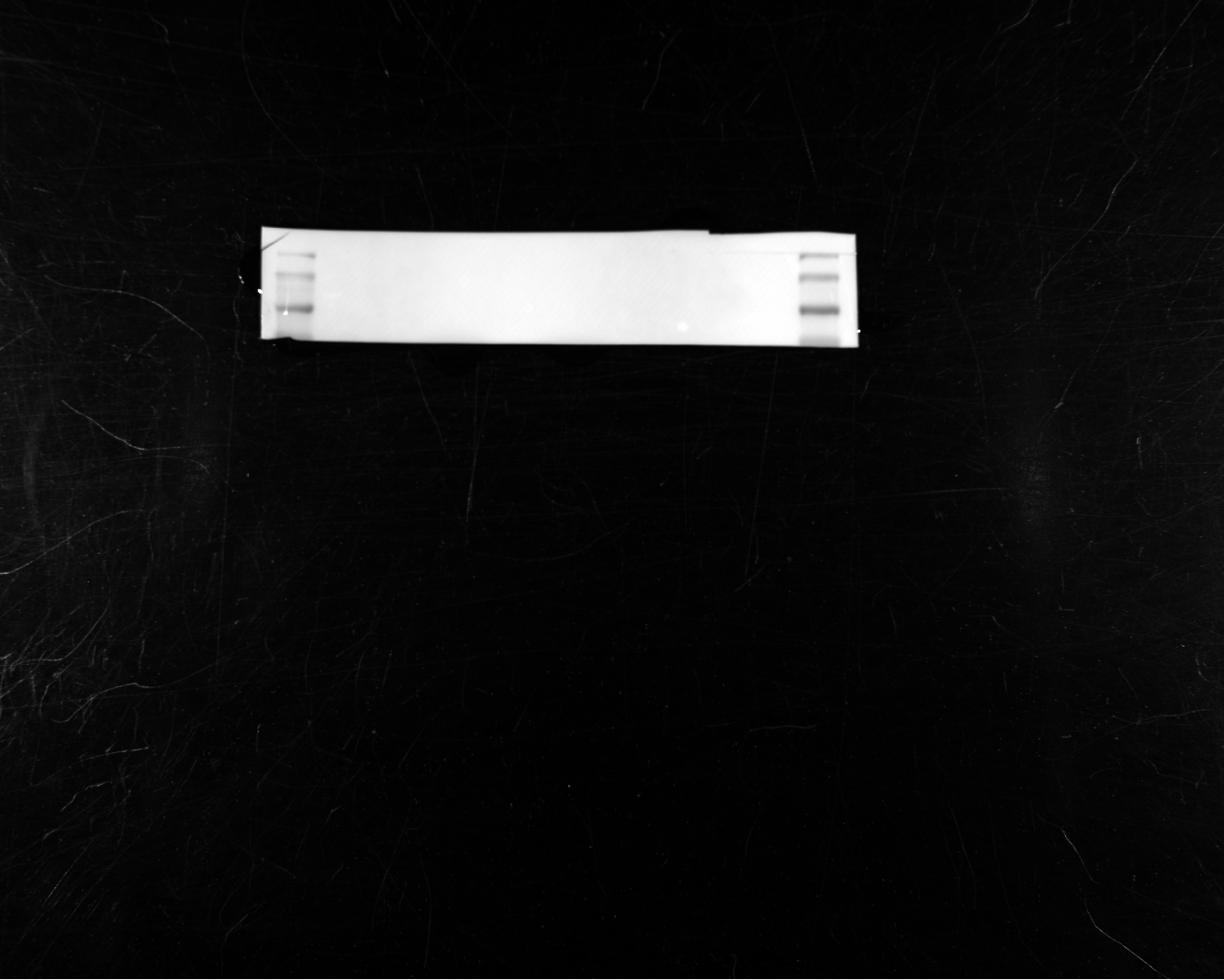

Supplement: Supplementary file 6 [file DataSheet1.ZIP › homo-ST/ratparp-2_4(Colorimetric).tif]

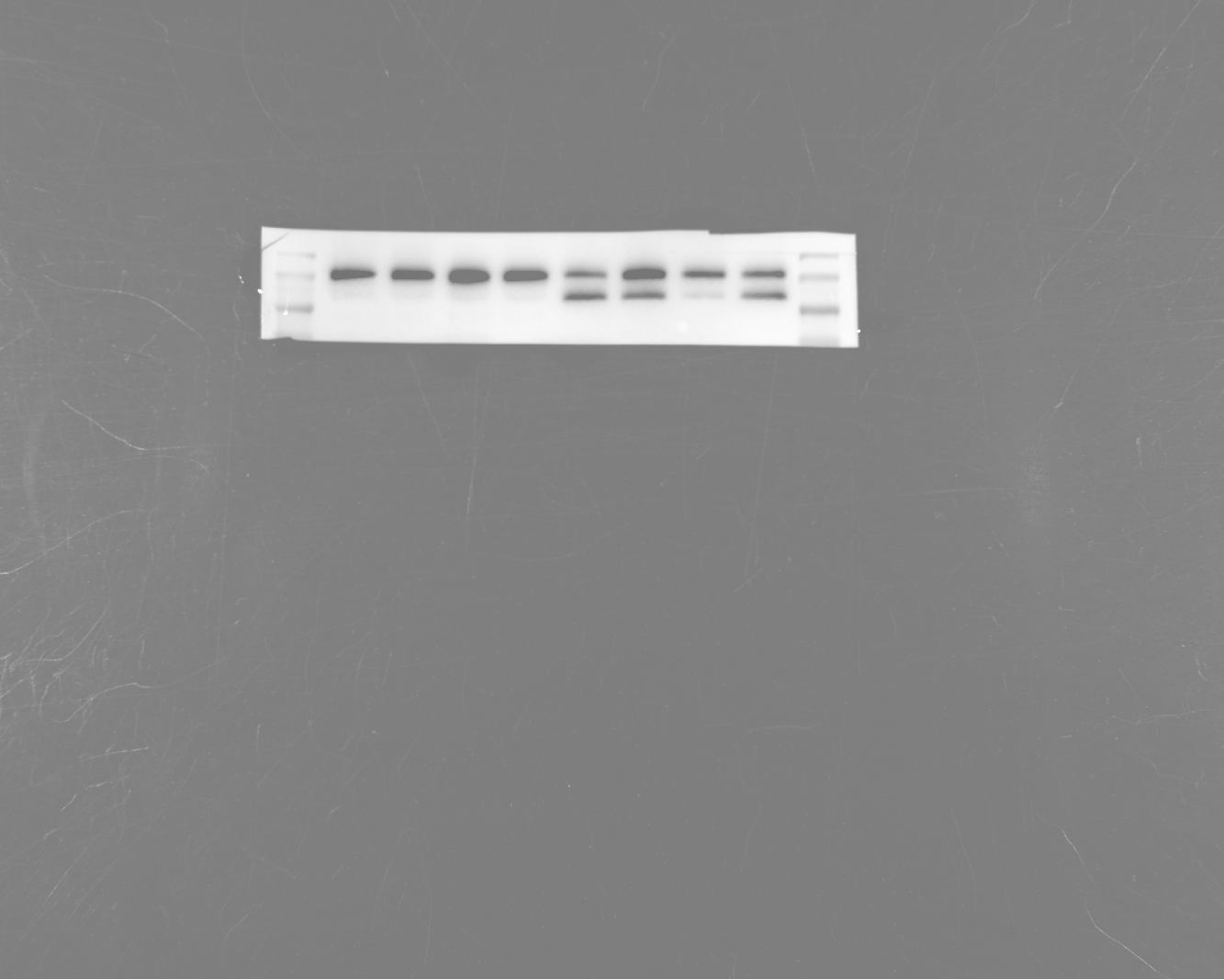

Supplement: Supplementary file 6 [file DataSheet1.ZIP › homo-ST/ratparp-2_4(Composite).tif]

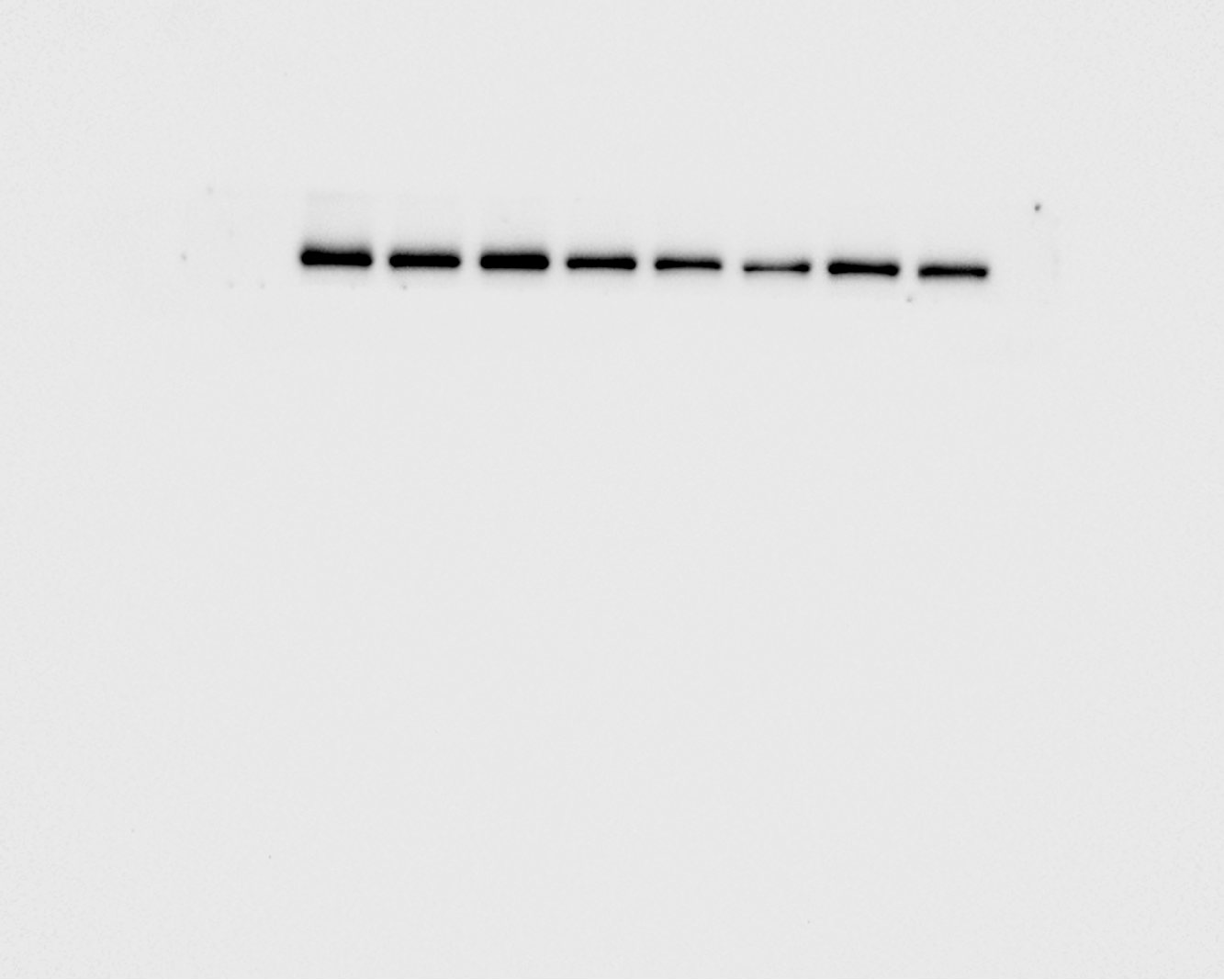

Supplement: Supplementary file 6 [file DataSheet1.ZIP › homo-ST/stat3-1stho_4(Chemiluminescence).tif]

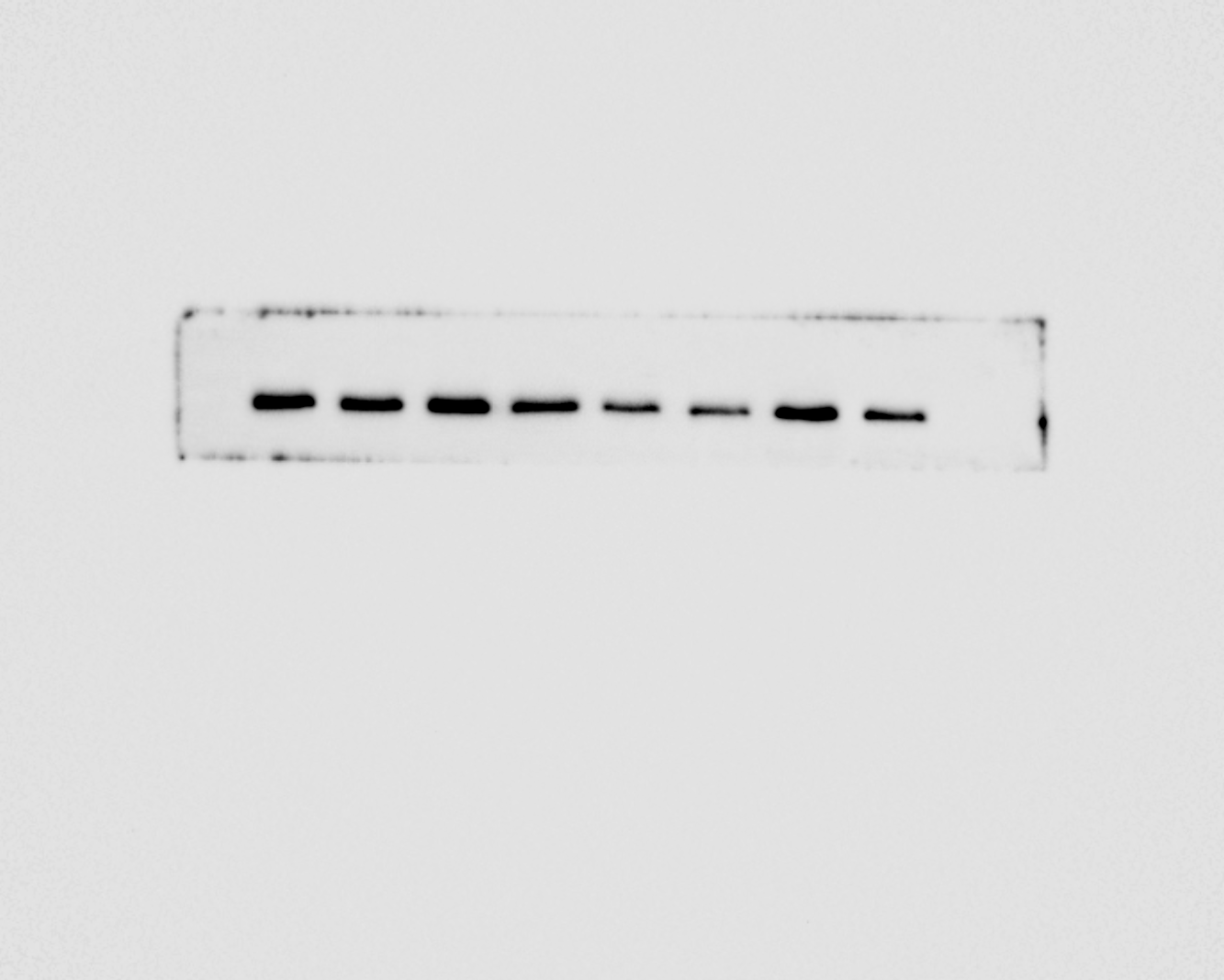

Supplement: Supplementary file 6 [file DataSheet1.ZIP › homo-ST/stat3-2.tif]

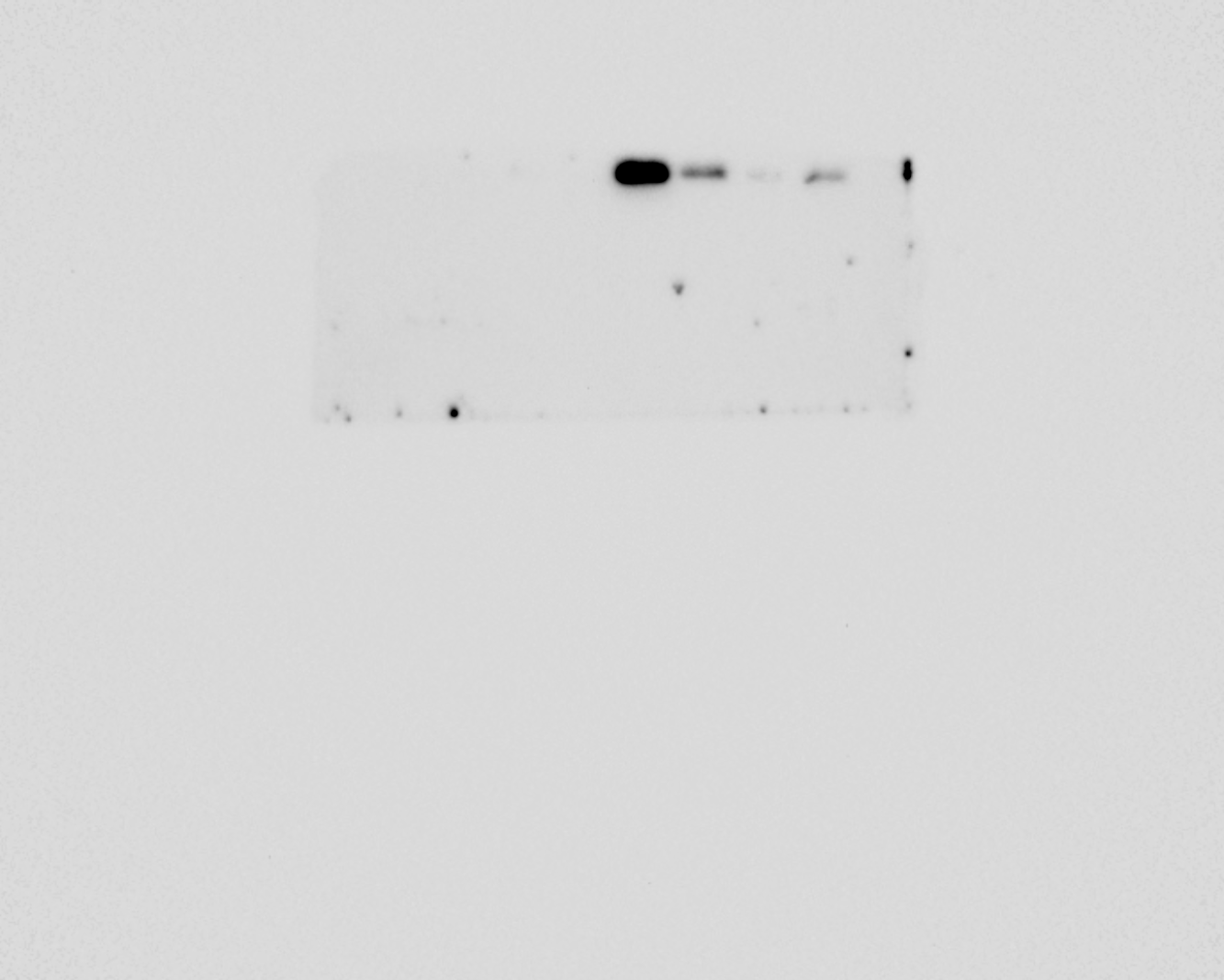

Supplement: Supplementary file 7 [file DataSheet10.ZIP › rat-ICC-ST/cc3-lop-st_1(Chemiluminescence) (1).tif]

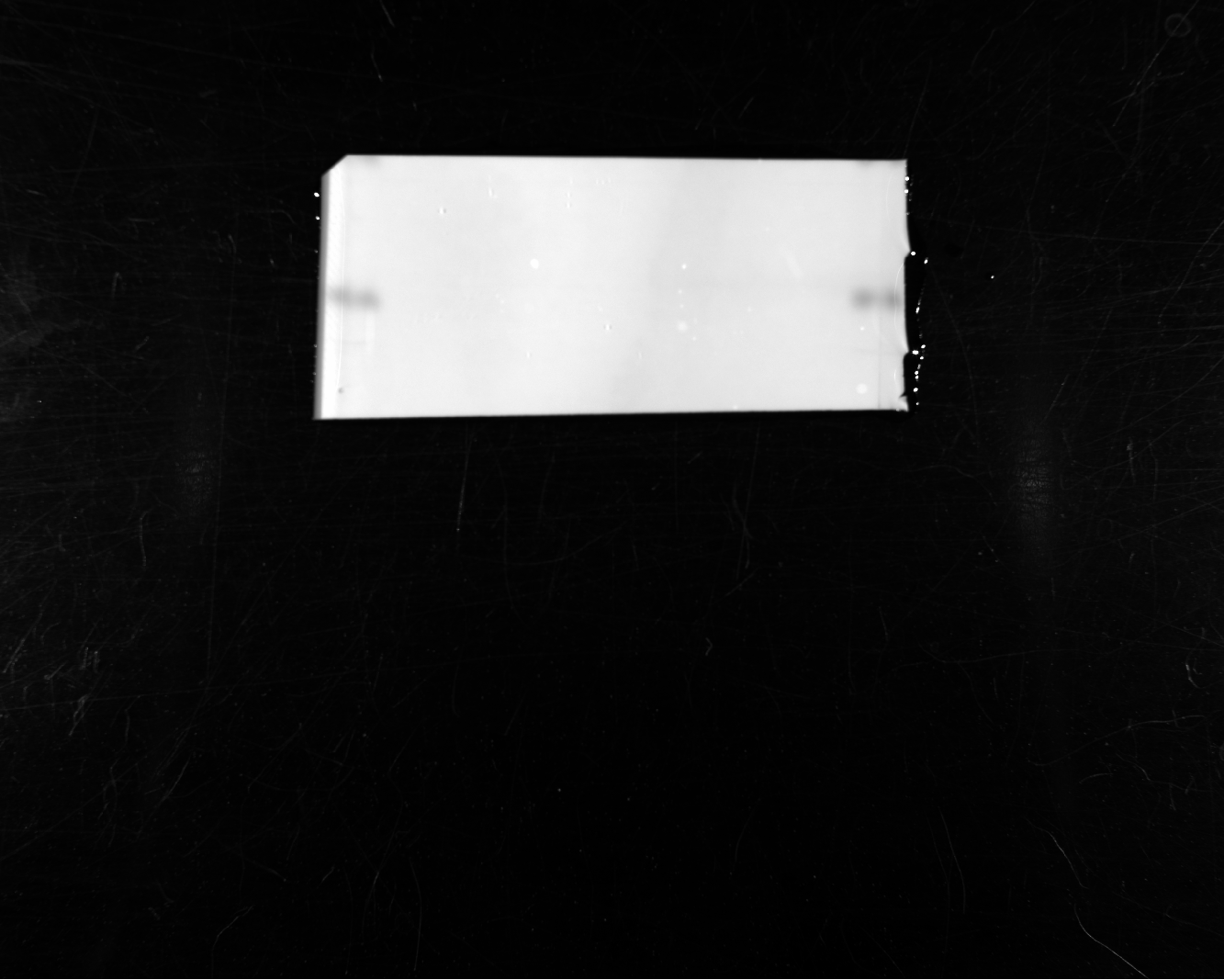

Supplement: Supplementary file 7 [file DataSheet10.ZIP › rat-ICC-ST/cc3-lop-st_1(Chemiluminescence) (2).tif]

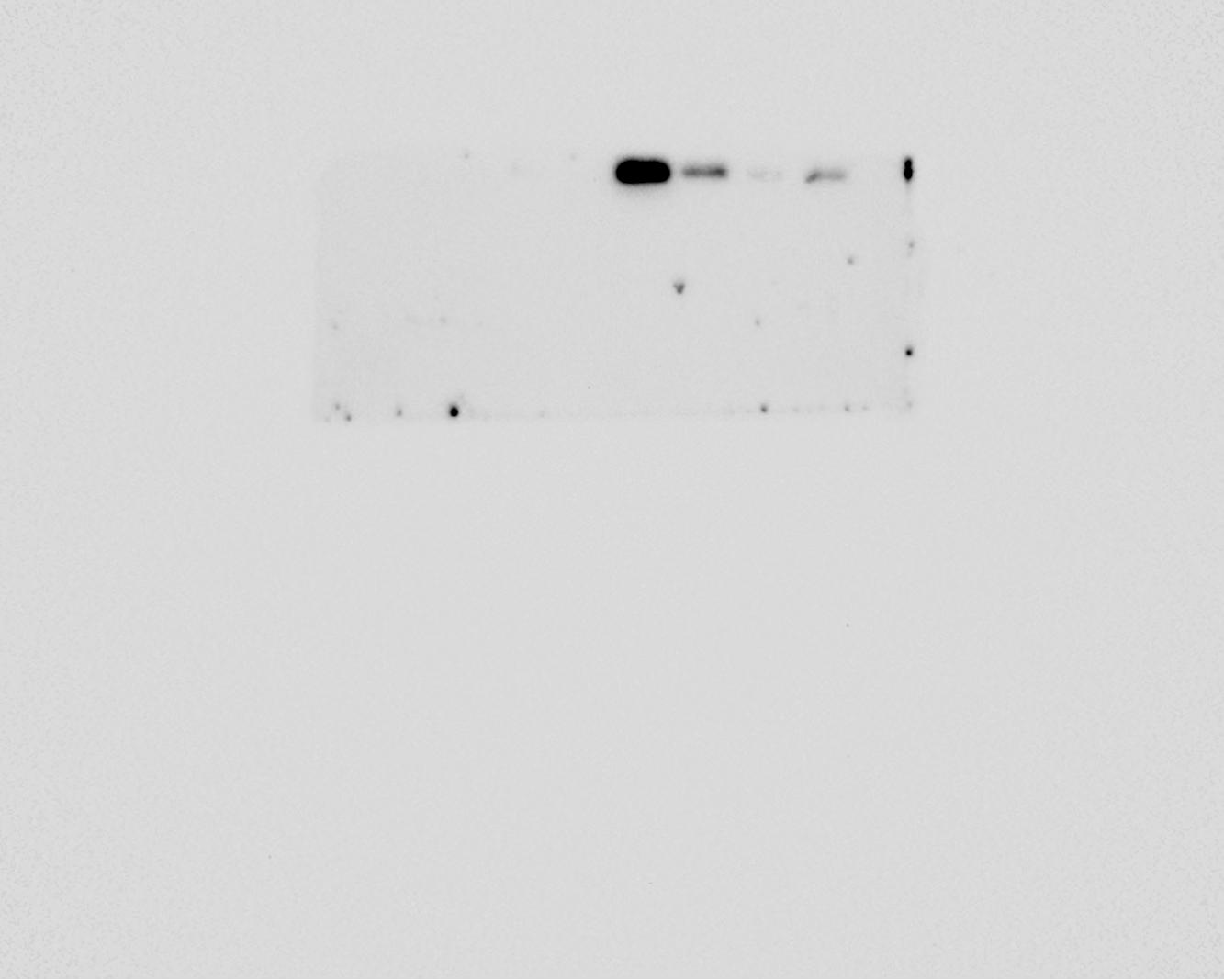

Supplement: Supplementary file 7 [file DataSheet10.ZIP › rat-ICC-ST/cc3-lop-st_1(Chemiluminescence) (3).tif]

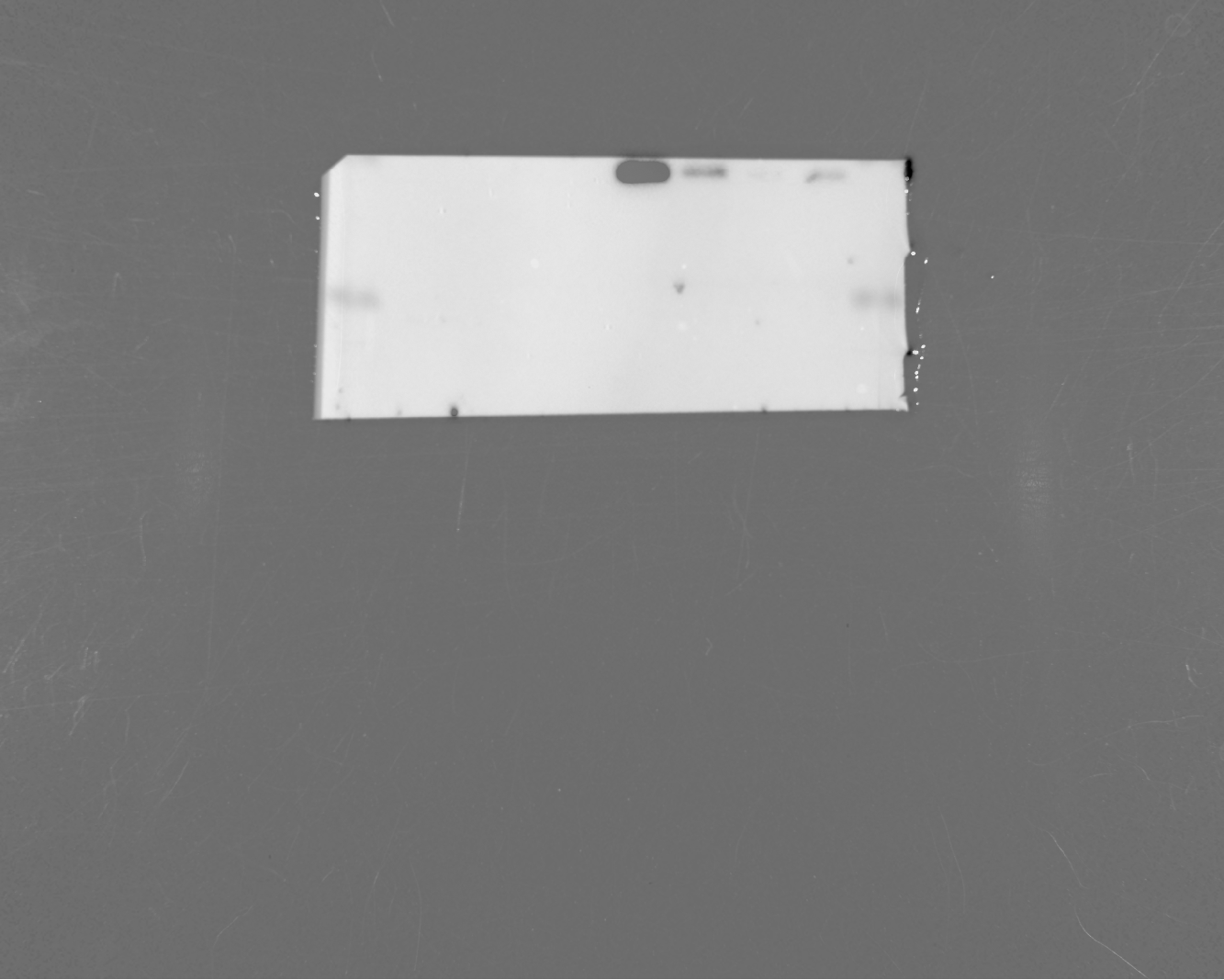

Supplement: Supplementary file 7 [file DataSheet10.ZIP › rat-ICC-ST/cc3-lop-st_1(Chemiluminescence) (5).tif]

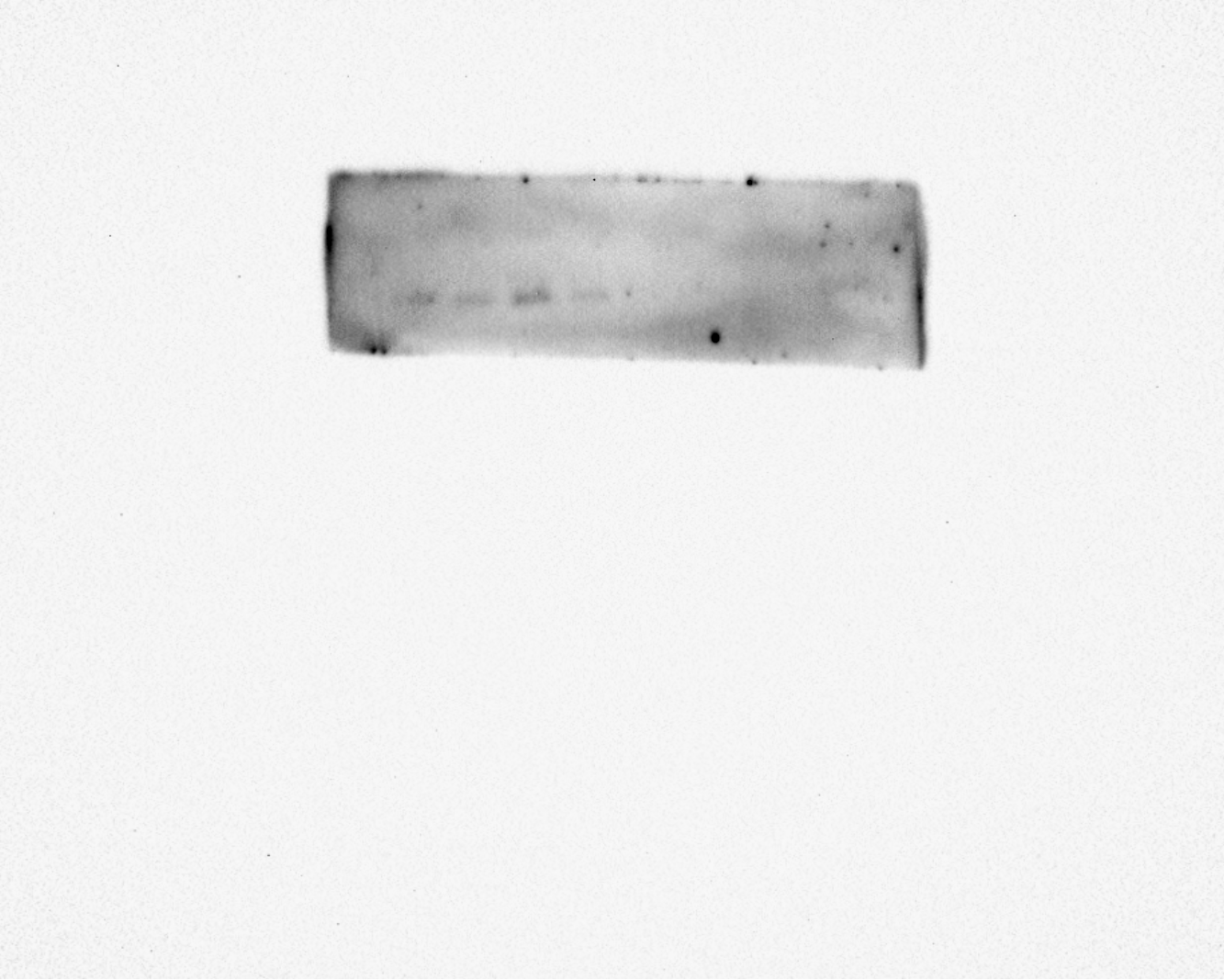

Supplement: Supplementary file 7 [file DataSheet10.ZIP › rat-ICC-ST/cyclind1rat_2(Chemiluminescence).tif]

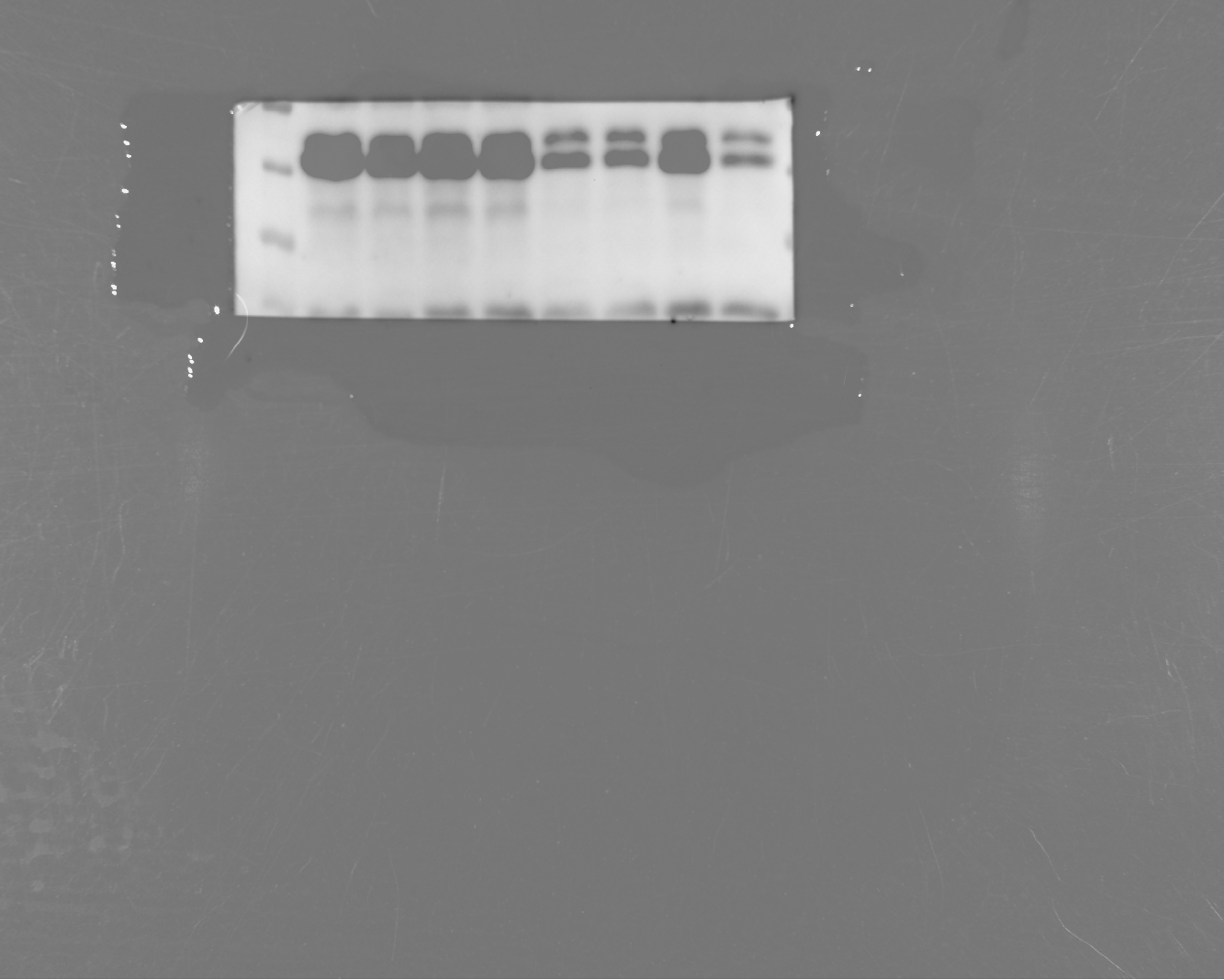

Supplement: Supplementary file 7 [file DataSheet10.ZIP › rat-ICC-ST/perk (1).tif]

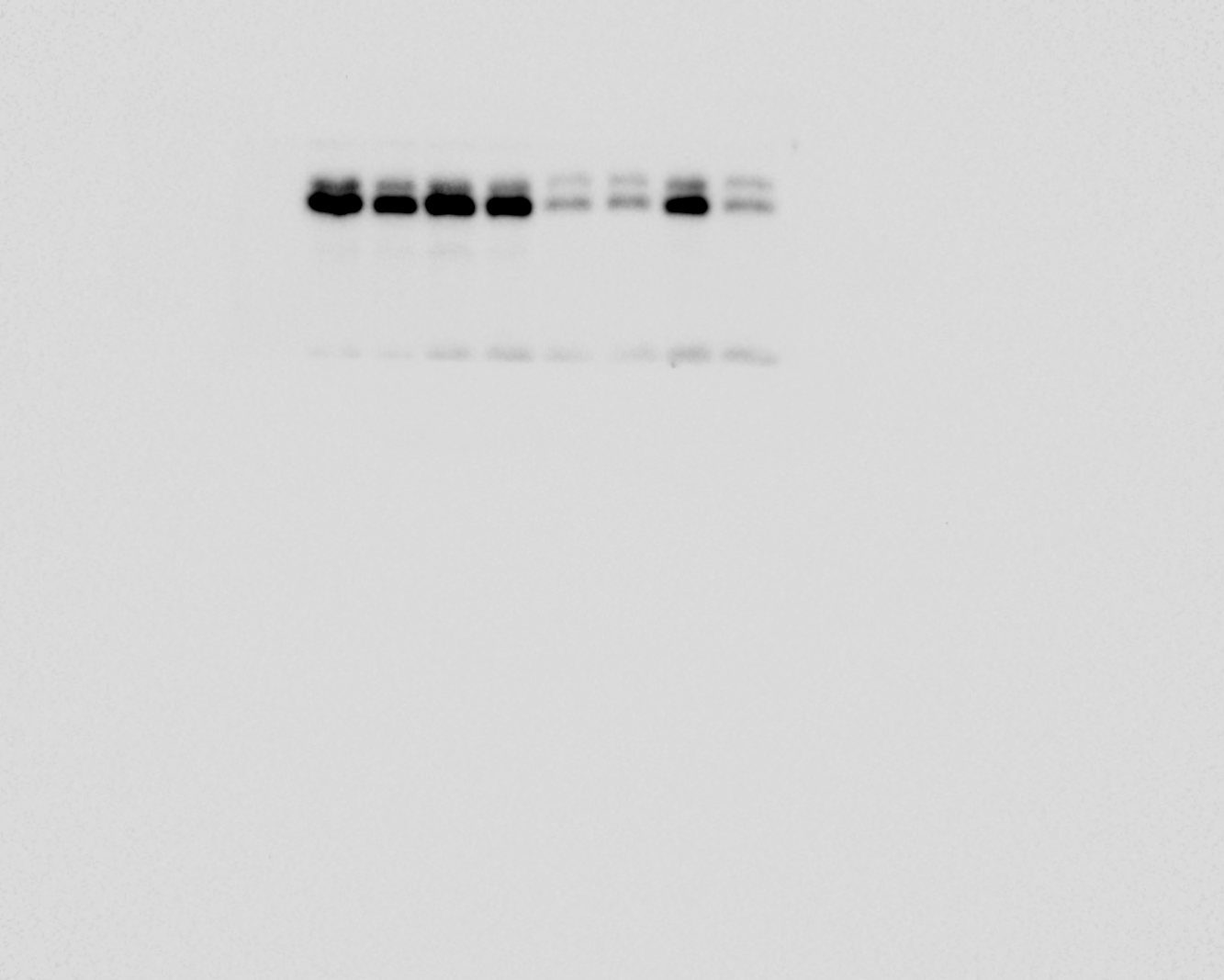

Supplement: Supplementary file 7 [file DataSheet10.ZIP › rat-ICC-ST/perk (2).tif]

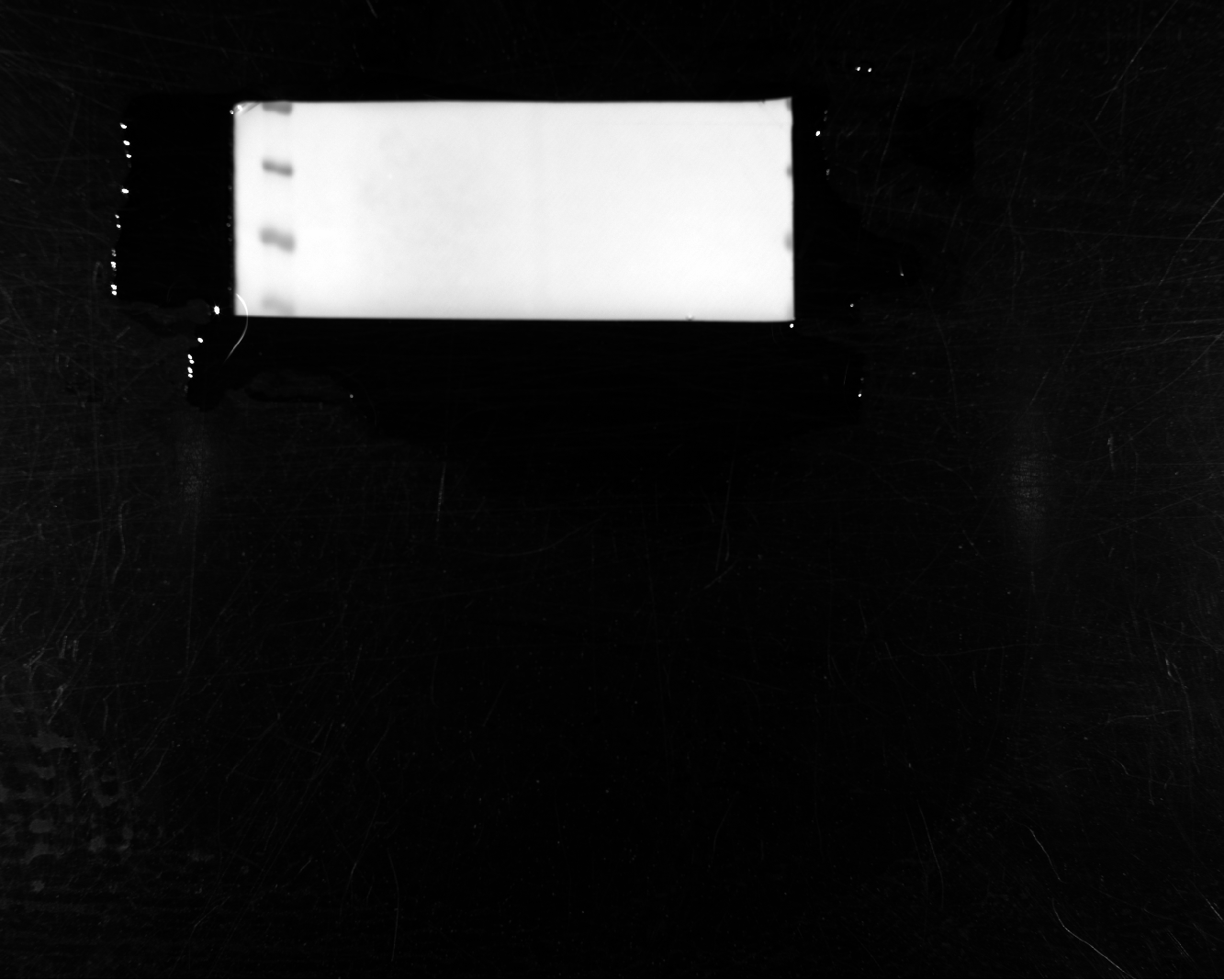

Supplement: Supplementary file 7 [file DataSheet10.ZIP › rat-ICC-ST/perk (3).tif]

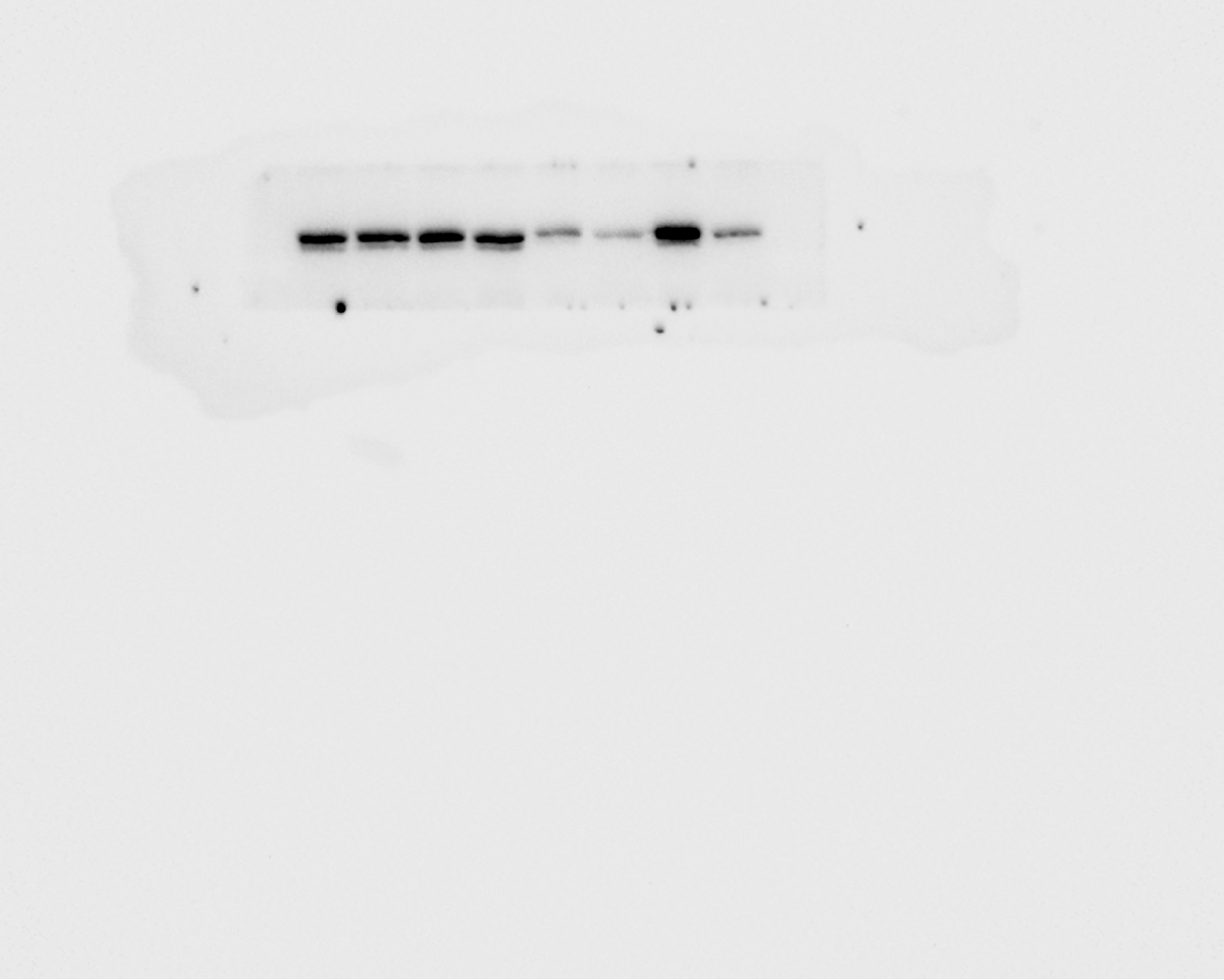

Supplement: Supplementary file 7 [file DataSheet10.ZIP › rat-ICC-ST/pst-lop-st-rat-2_3(Chemiluminescence).tif]

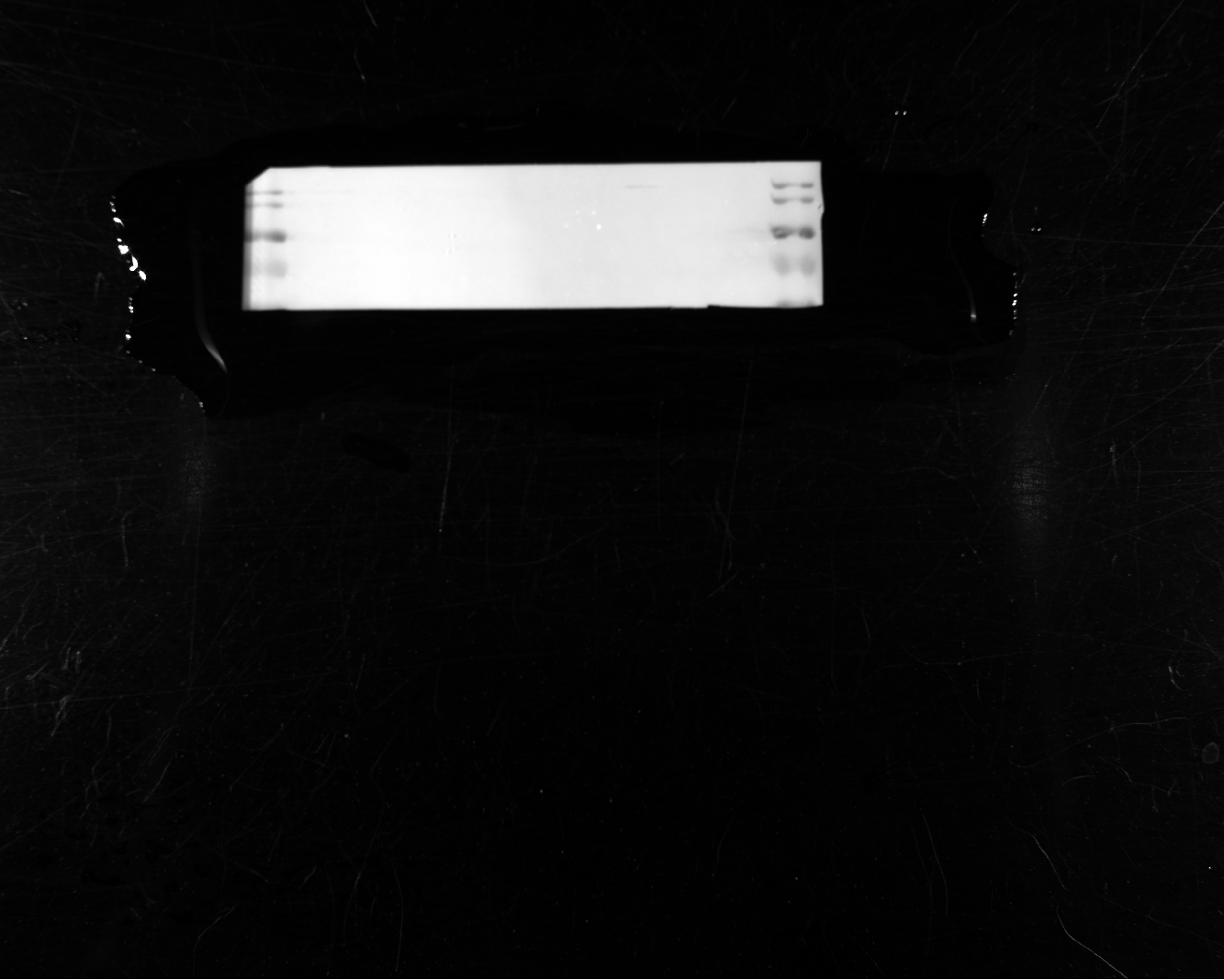

Supplement: Supplementary file 7 [file DataSheet10.ZIP › rat-ICC-ST/pst-lop-st-rat-2_3(Colorimetric).tif]

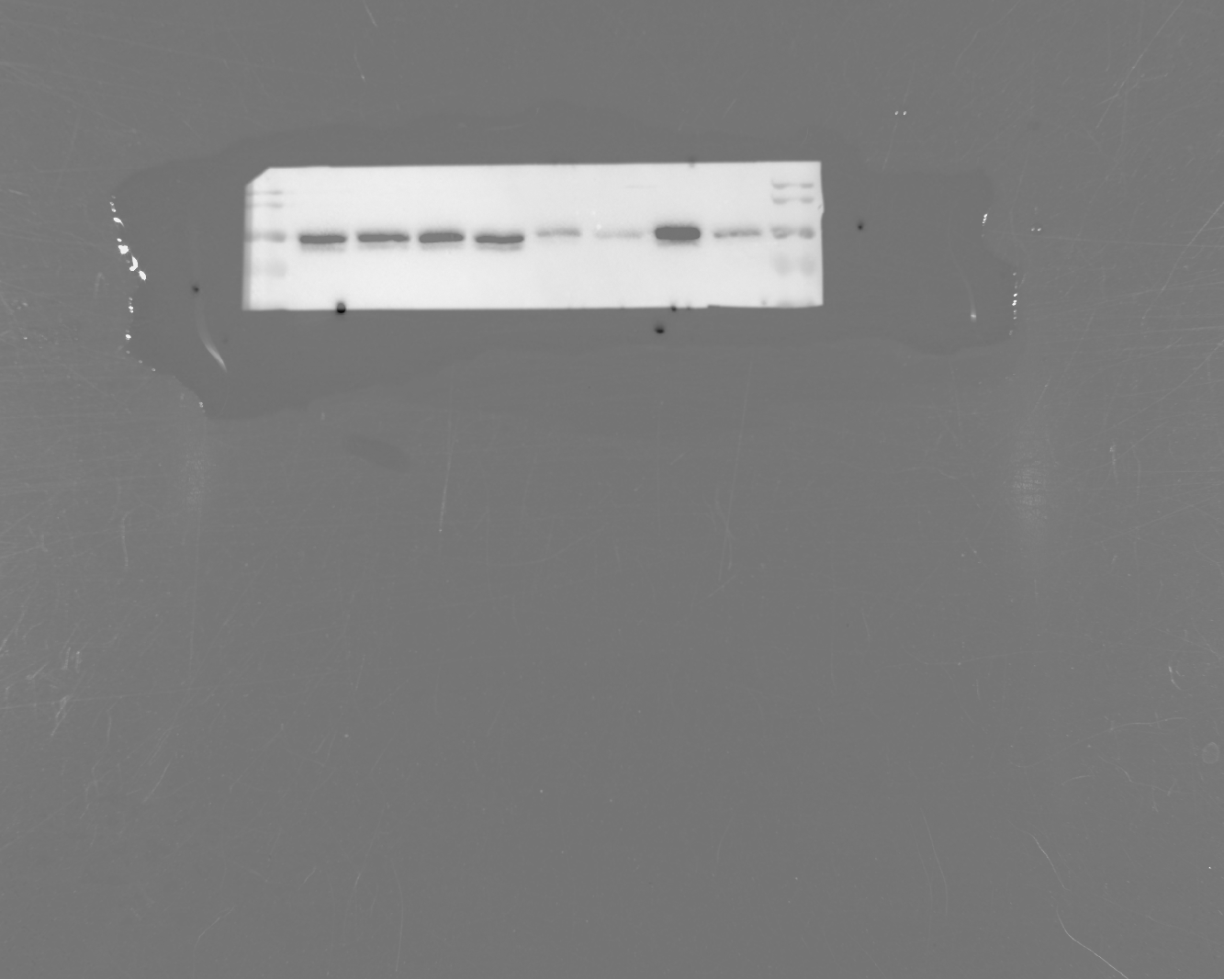

Supplement: Supplementary file 7 [file DataSheet10.ZIP › rat-ICC-ST/pst-lop-st-rat-2_3(Composite).tif]

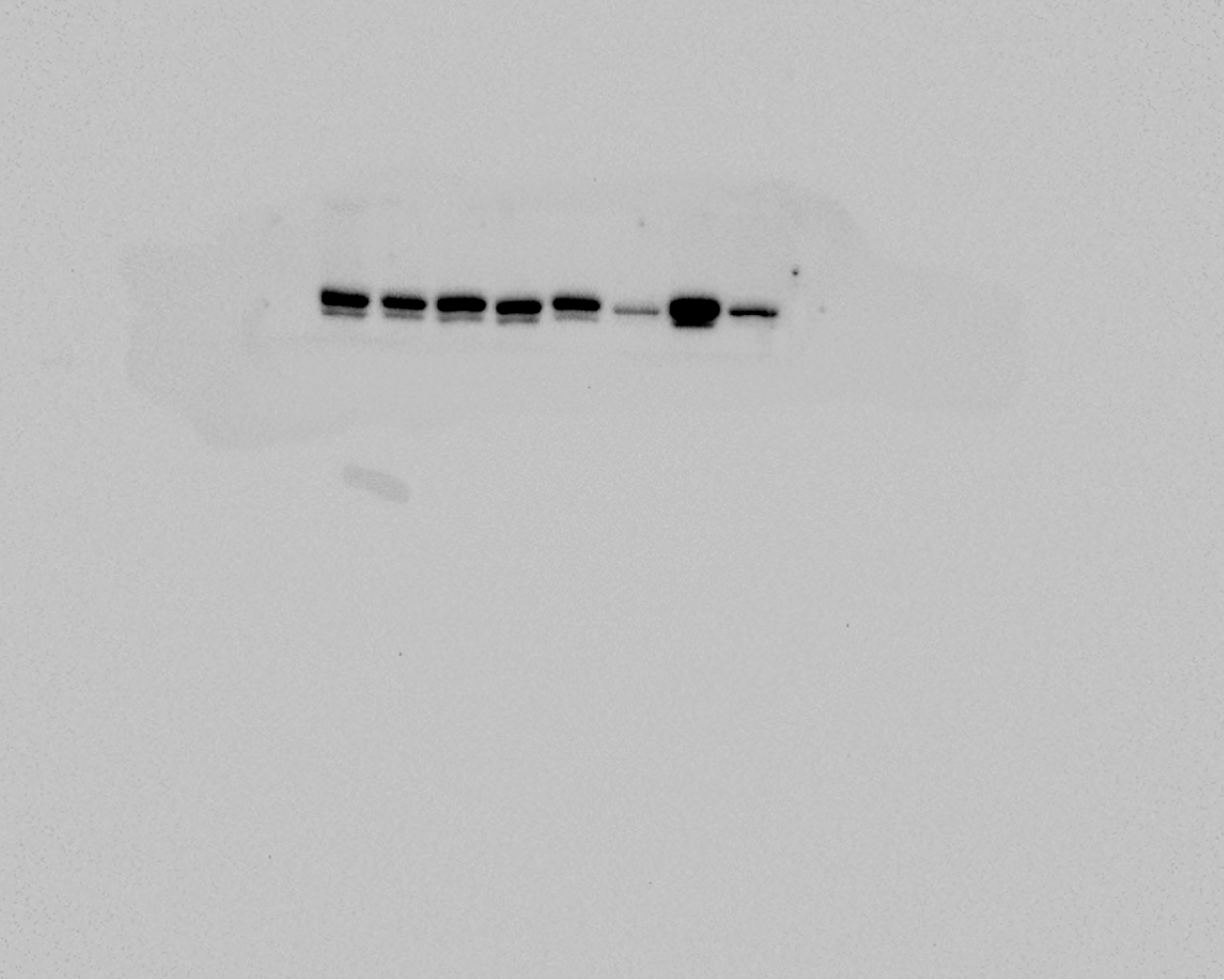

Supplement: Supplementary file 7 [file DataSheet10.ZIP › rat-ICC-ST/pst-rat-loo-st_3(Chemiluminescence).tif]

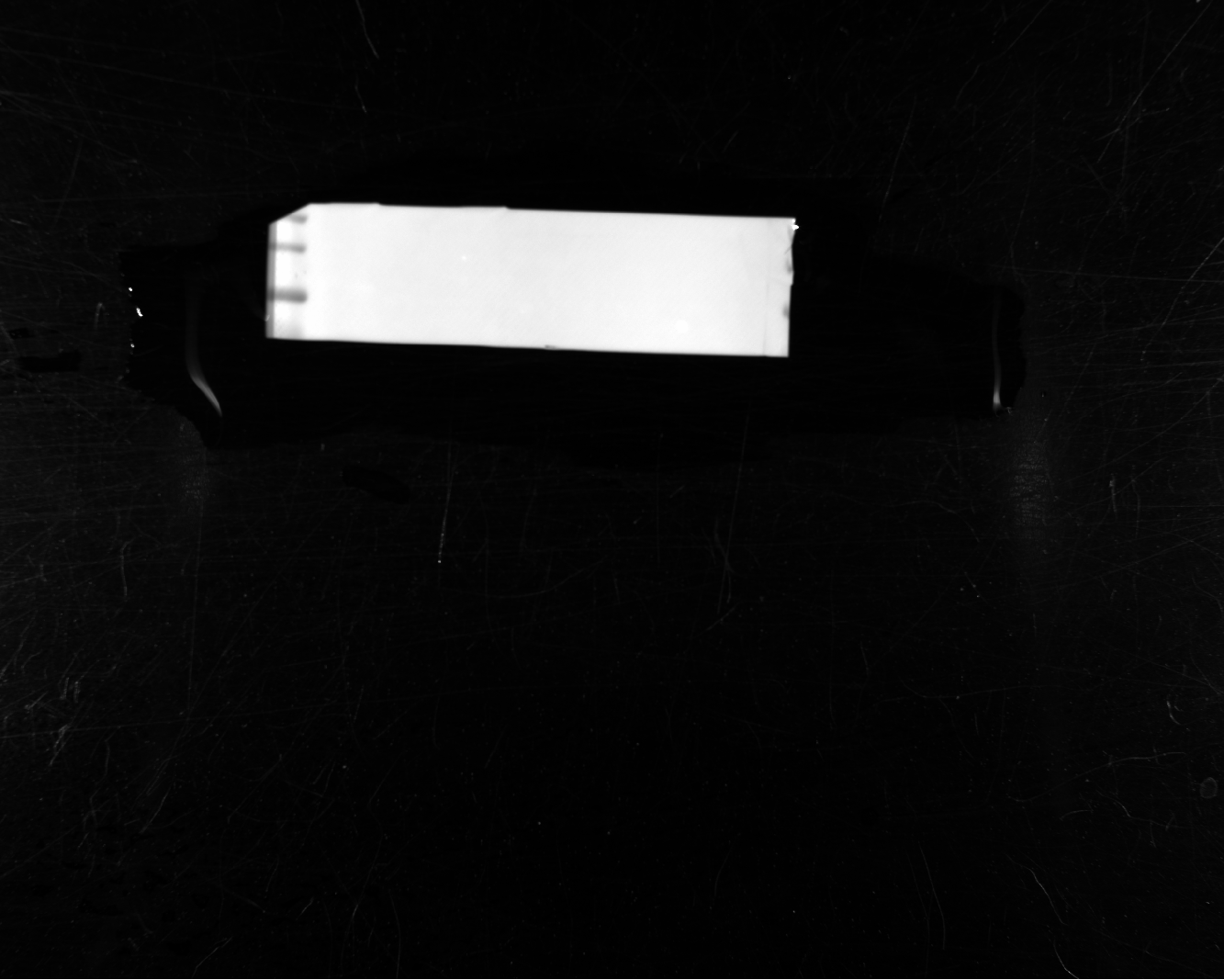

Supplement: Supplementary file 7 [file DataSheet10.ZIP › rat-ICC-ST/pst-rat-loo-st_3(Colorimetric).tif]

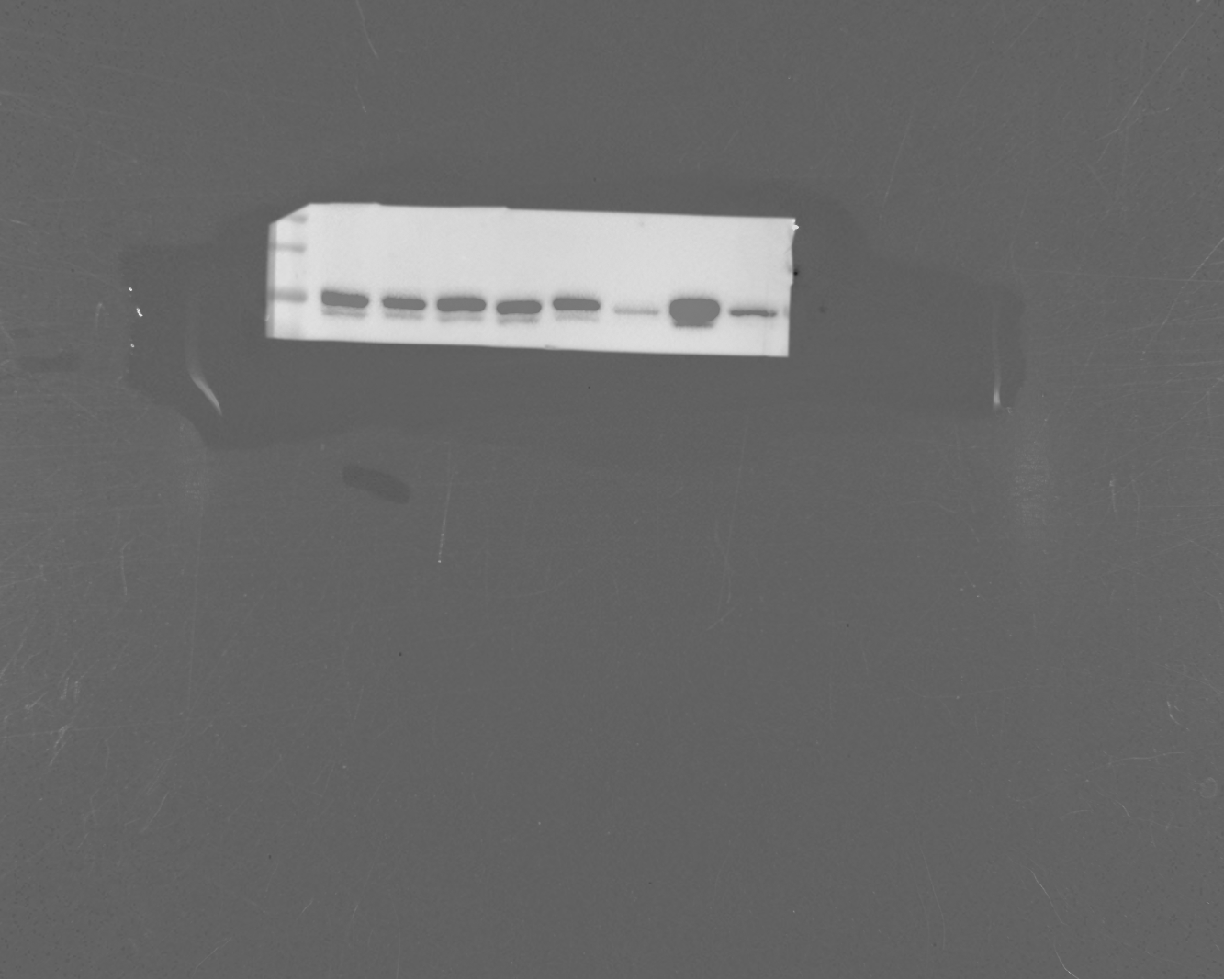

Supplement: Supplementary file 7 [file DataSheet10.ZIP › rat-ICC-ST/pst-rat-loo-st_3(Composite).tif]

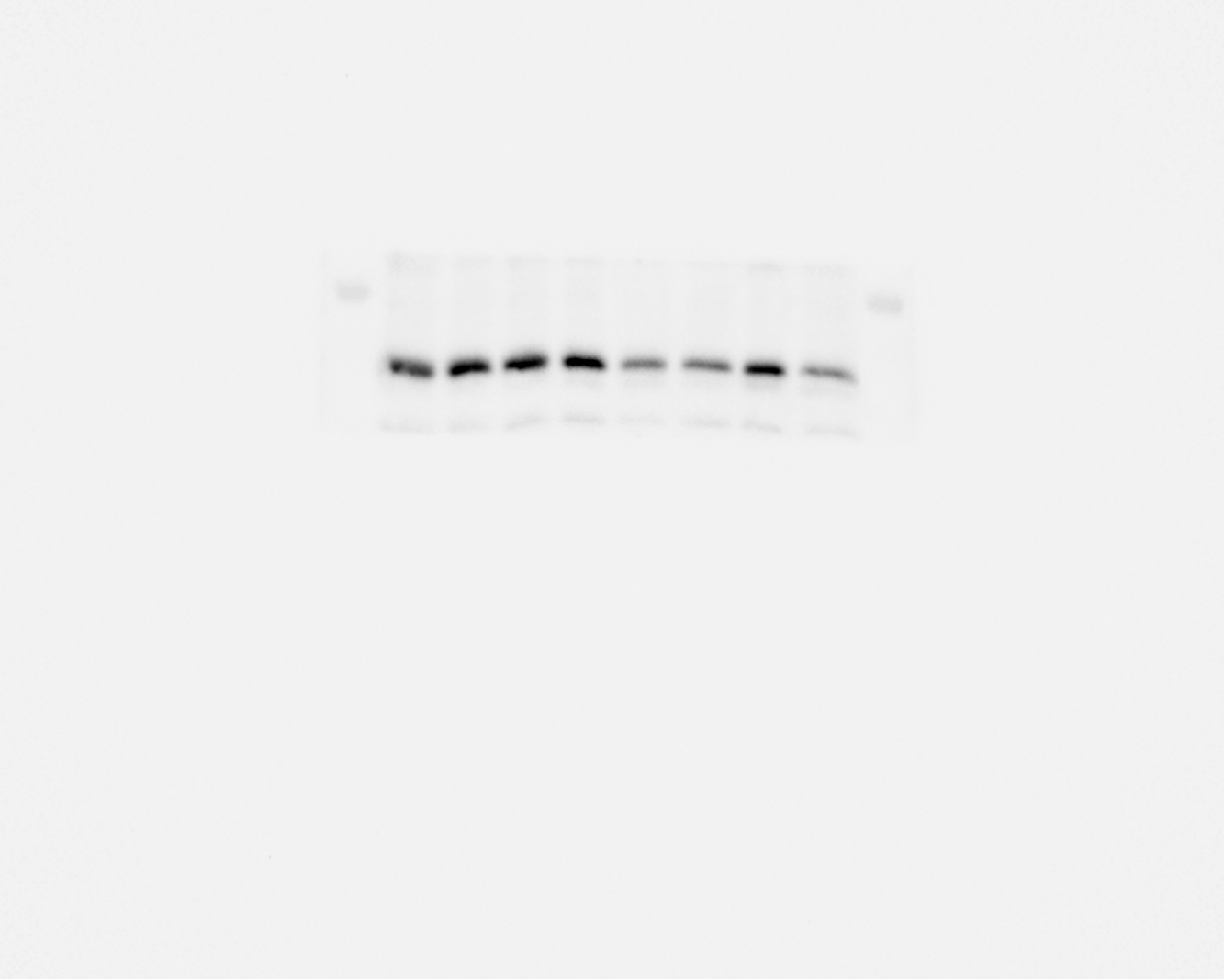

Supplement: Supplementary file 7 [file DataSheet10.ZIP › rat-ICC-ST/rat8-cas3_4(Chemiluminescence).tif]

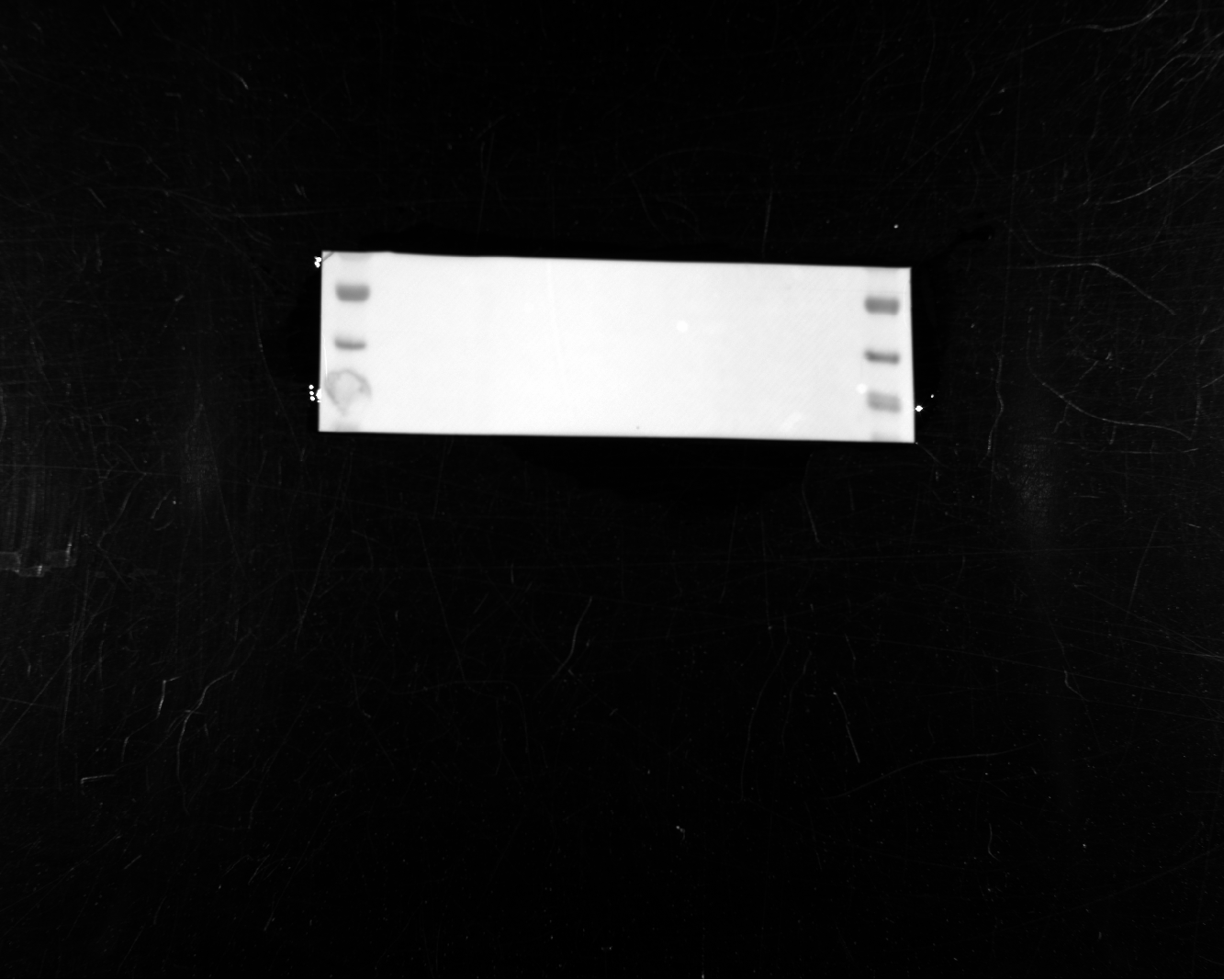

Supplement: Supplementary file 7 [file DataSheet10.ZIP › rat-ICC-ST/rat8-cas3_4(Colorimetric).tif]

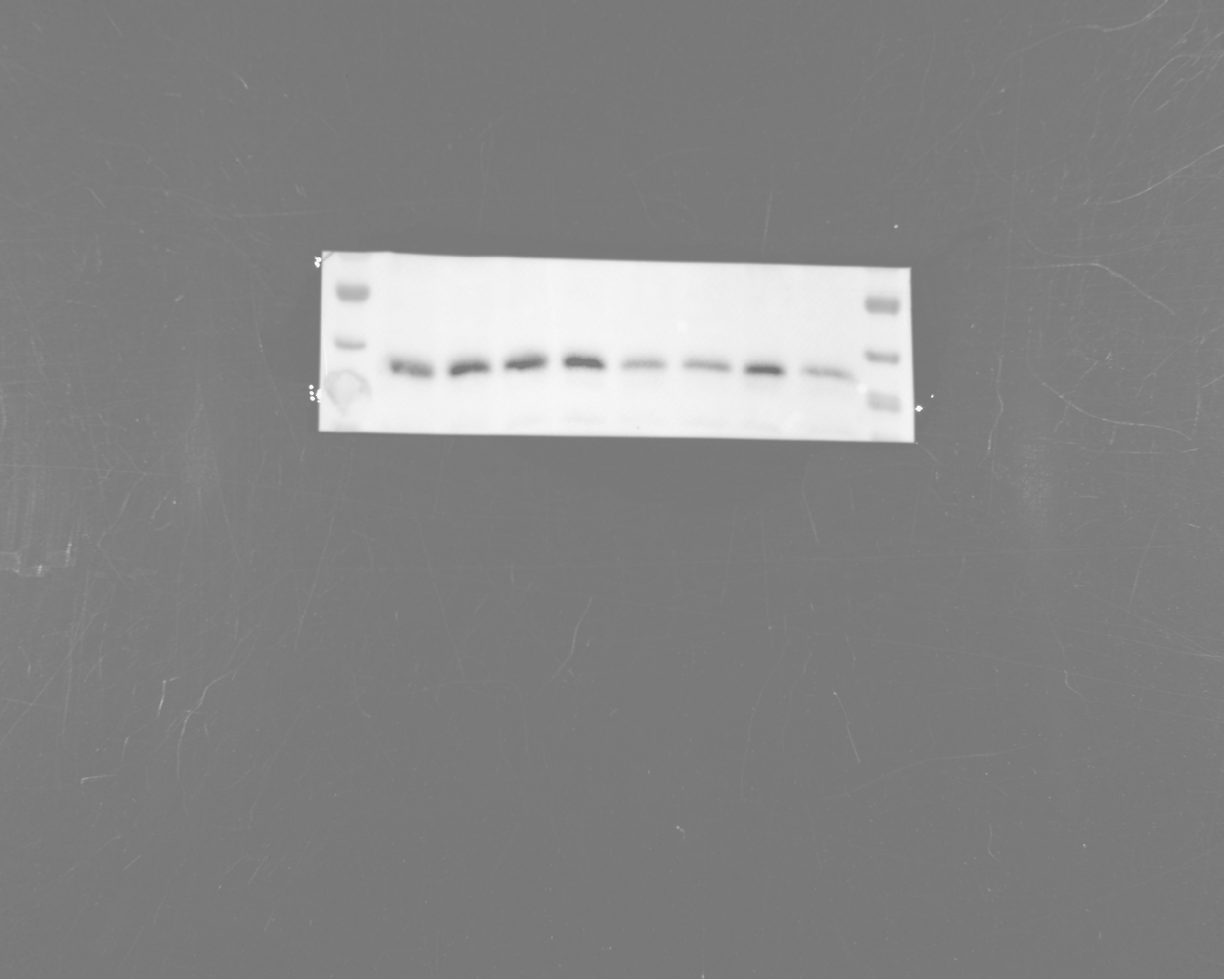

Supplement: Supplementary file 7 [file DataSheet10.ZIP › rat-ICC-ST/rat8-cas3_4(Composite).tif]

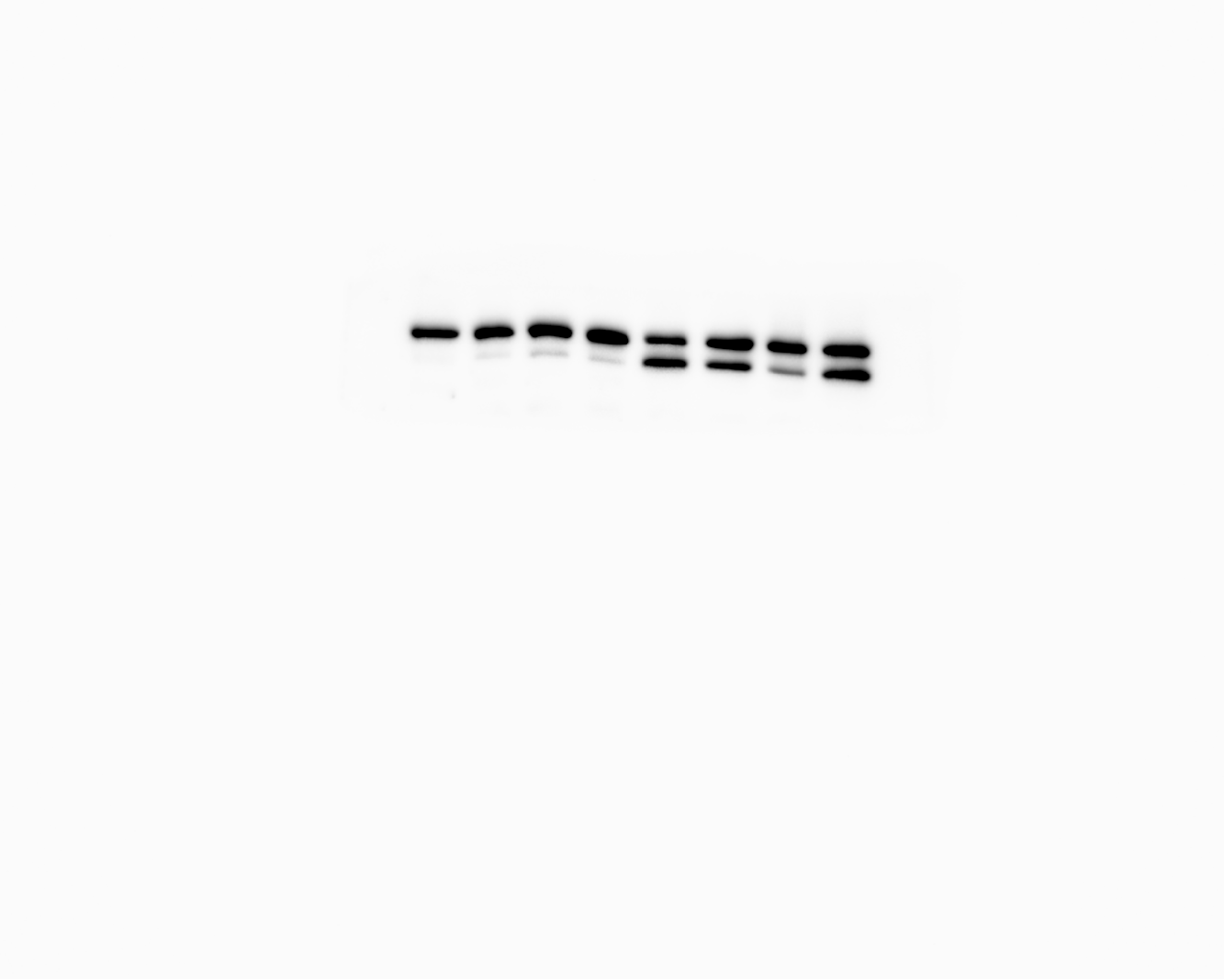

Supplement: Supplementary file 7 [file DataSheet10.ZIP › rat-ICC-ST/rat8-parp_3(Chemiluminescence).tif]

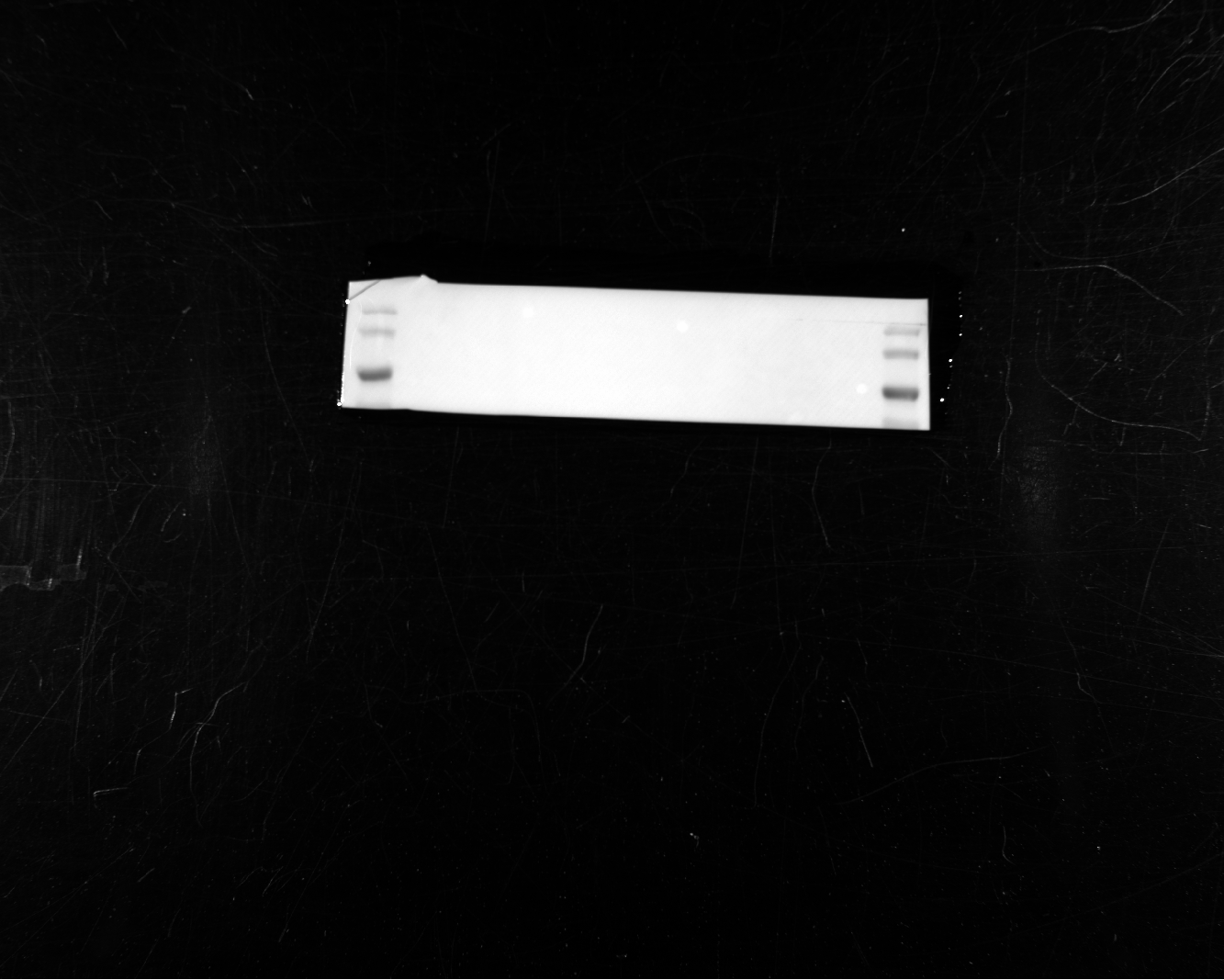

Supplement: Supplementary file 7 [file DataSheet10.ZIP › rat-ICC-ST/rat8-parp_3(Colorimetric).tif]

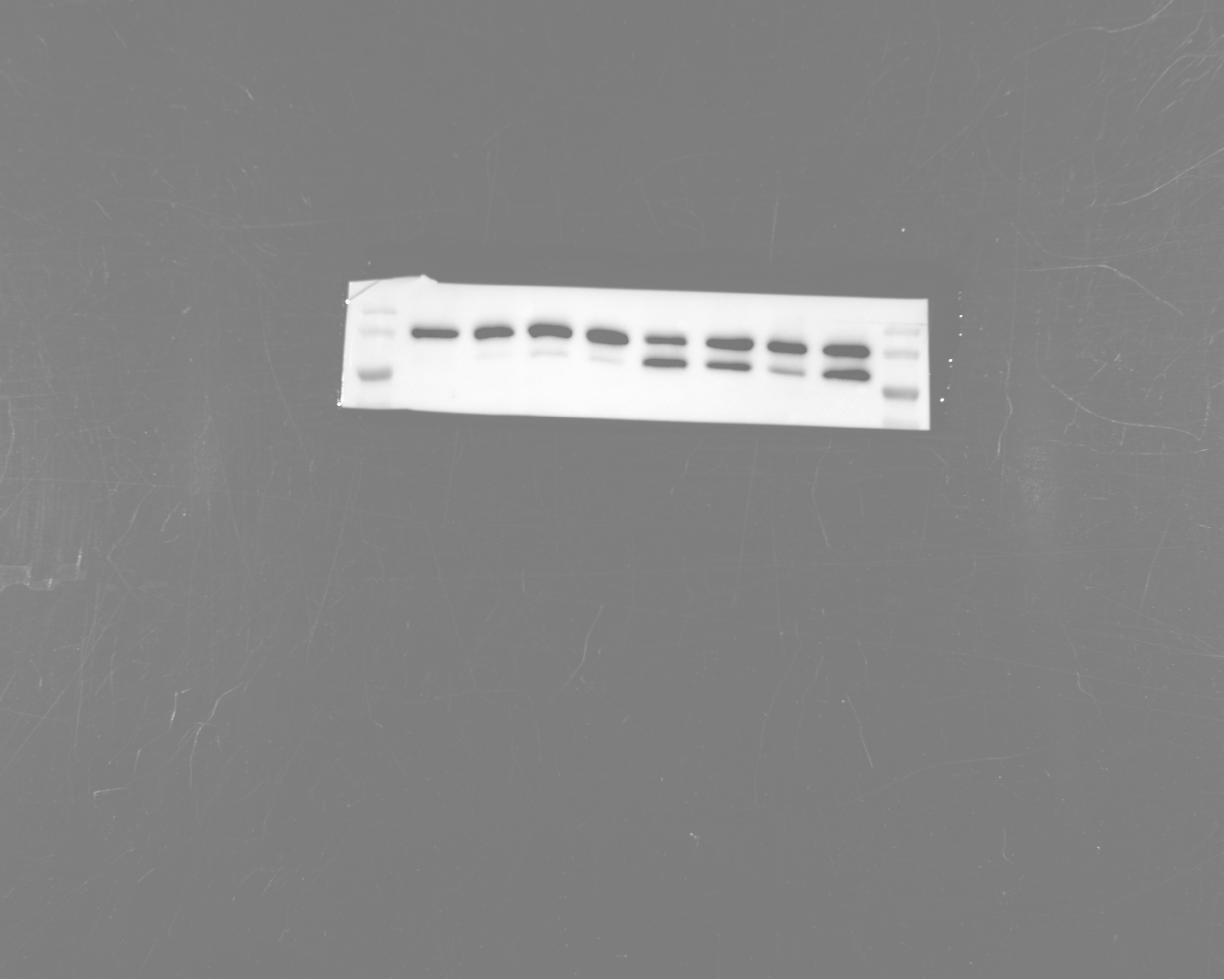

Supplement: Supplementary file 7 [file DataSheet10.ZIP › rat-ICC-ST/rat8-parp_3(Composite).tif]

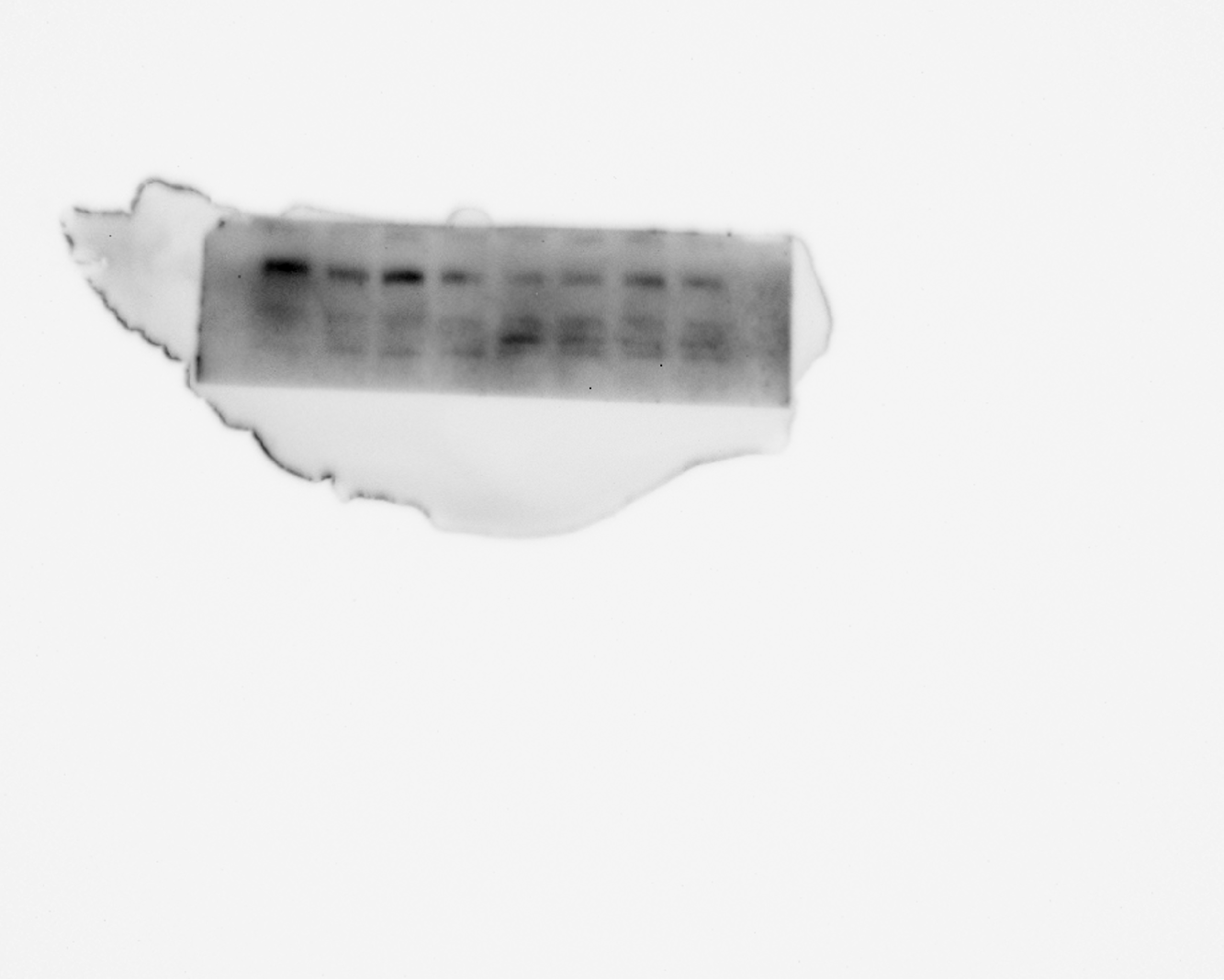

Supplement: Supplementary file 7 [file DataSheet10.ZIP › rat-ICC-ST/ratcas9-2_4(Chemiluminescence).tif]

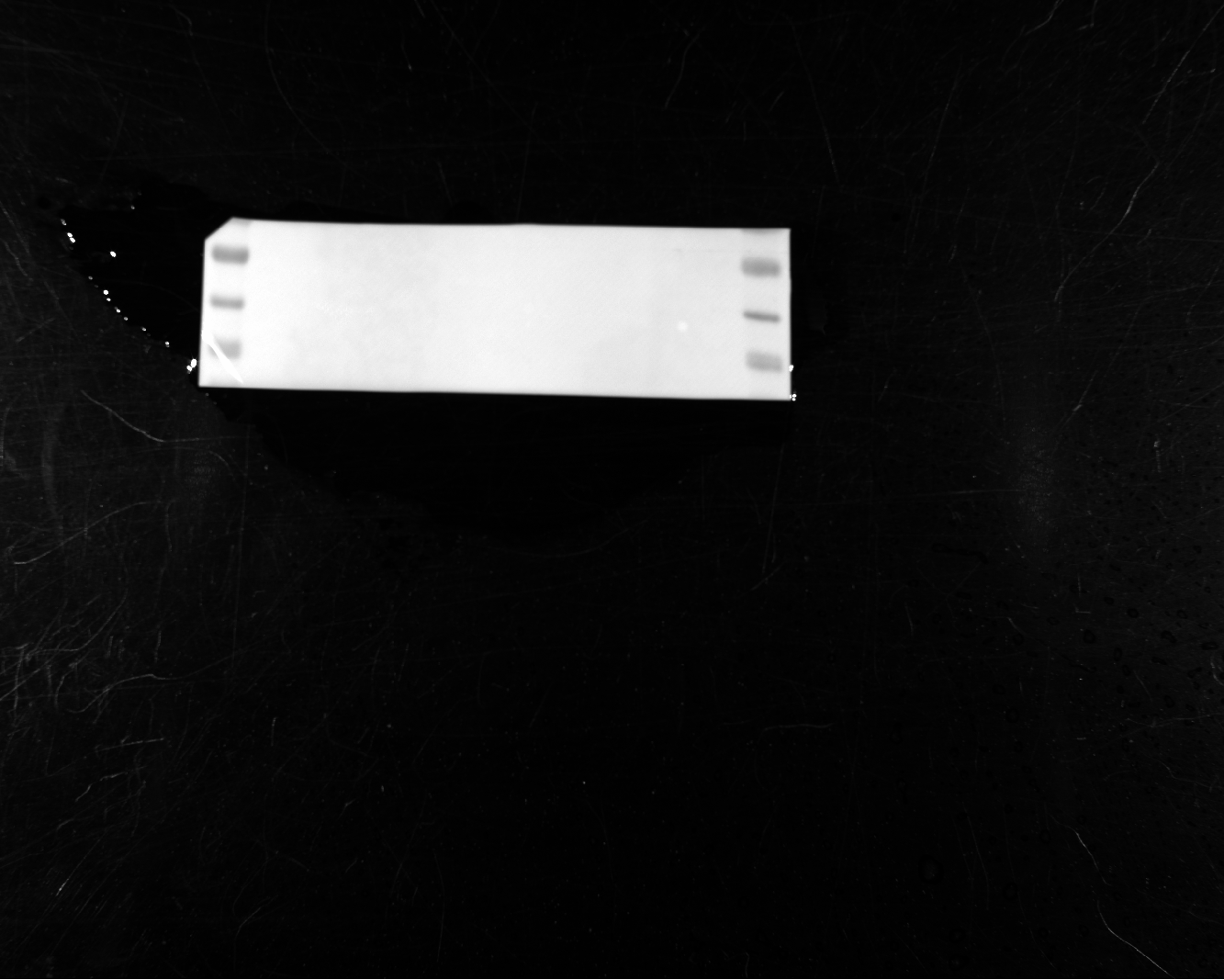

Supplement: Supplementary file 7 [file DataSheet10.ZIP › rat-ICC-ST/ratcas9-2_4(Colorimetric).tif]

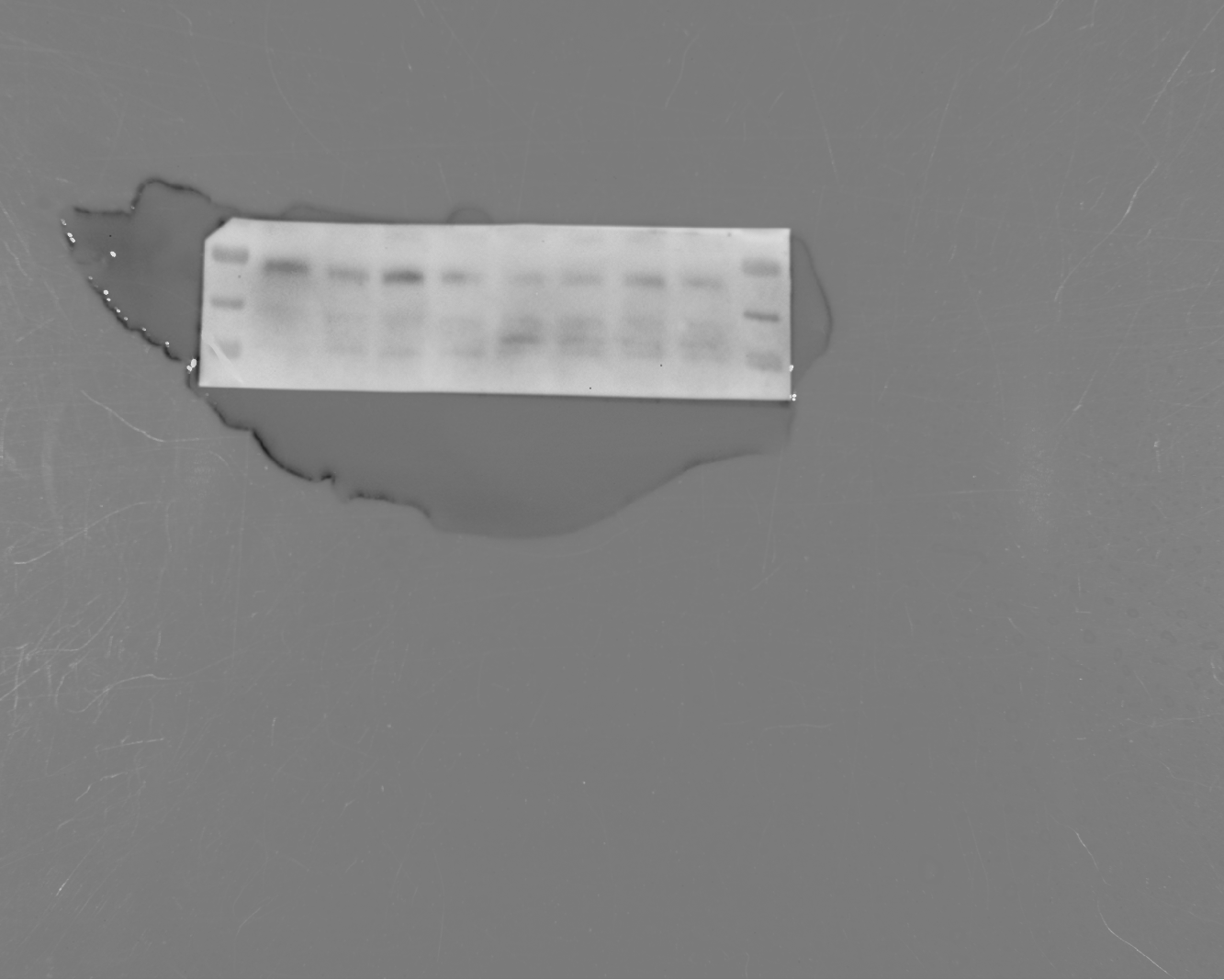

Supplement: Supplementary file 7 [file DataSheet10.ZIP › rat-ICC-ST/ratcas9-2_4(Composite).tif]

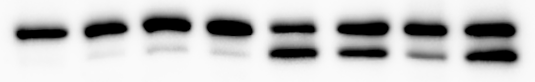

Supplement: Supplementary file 7 [file DataSheet10.ZIP › rat-ICC-ST/╝⌠▓├-rat8-parp_3(Chemiluminescence).tif]

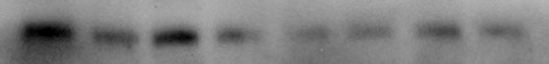

Supplement: Supplementary file 7 [file DataSheet10.ZIP › rat-ICC-ST/╝⌠▓├-ratcas9-2_4(Chemiluminescence).tif]

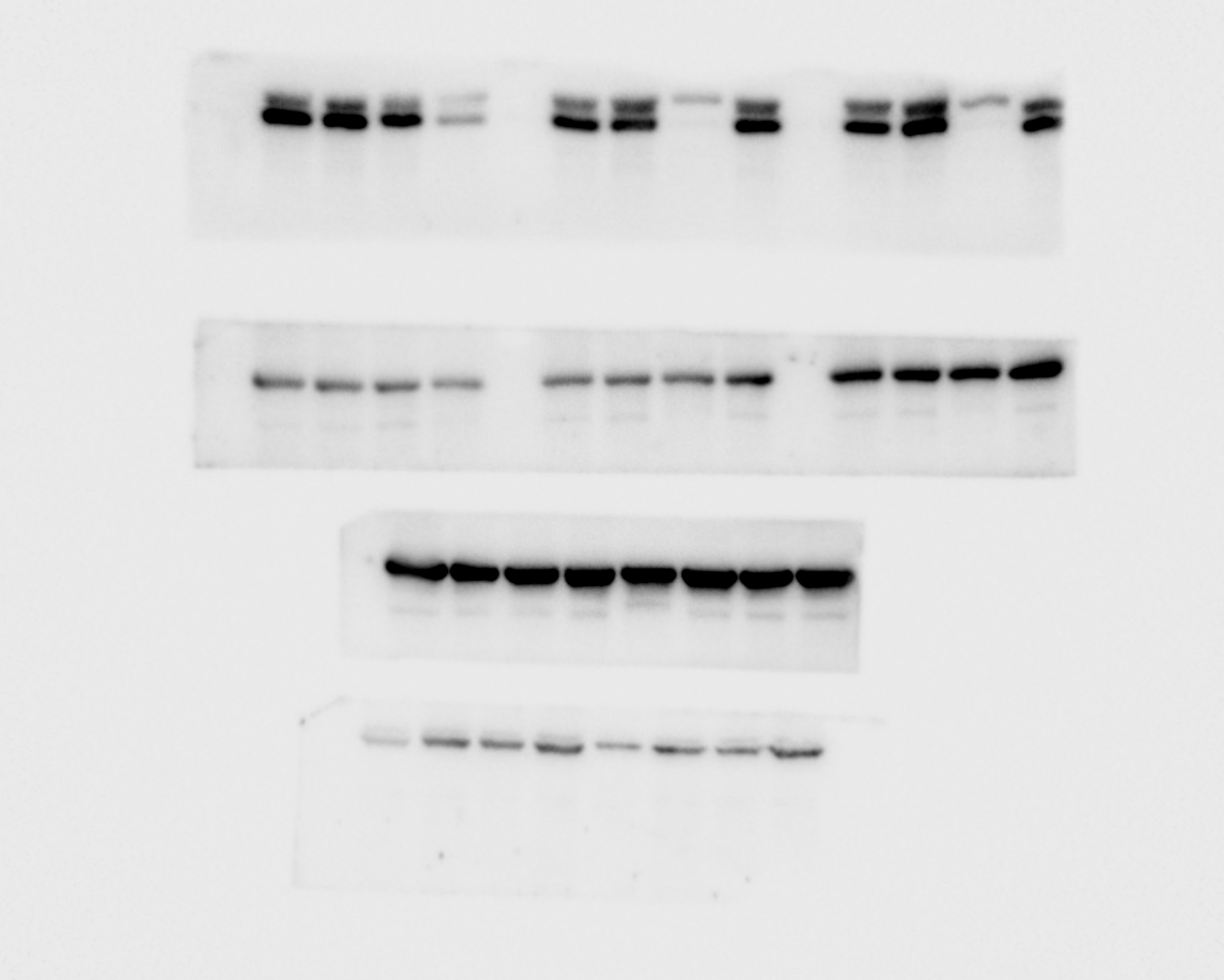

Supplement: Supplementary file 8 [file DataSheet6.ZIP › rat-ICC-L+B/actin-rat_2(Chemiluminescence).tif]

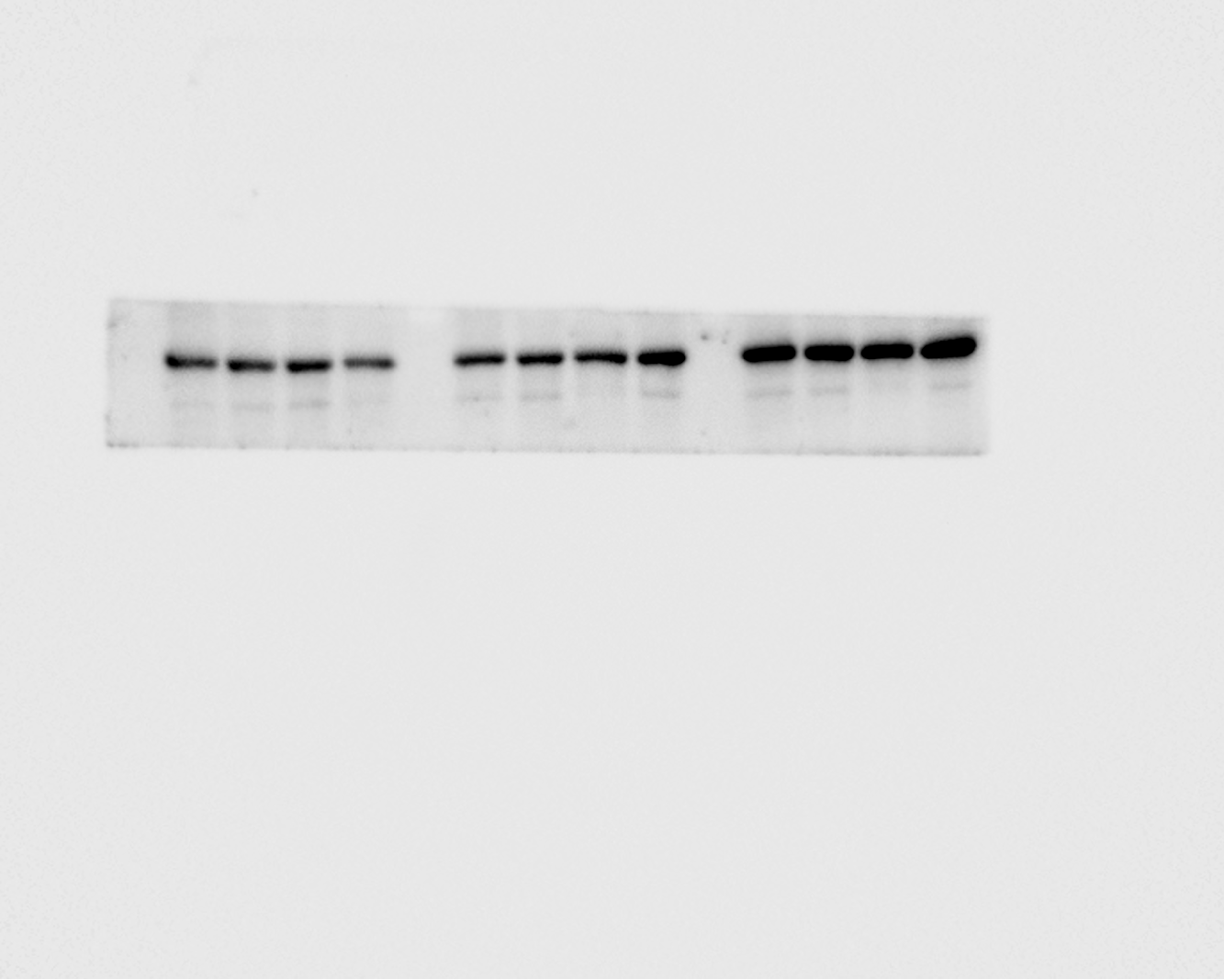

Supplement: Supplementary file 8 [file DataSheet6.ZIP › rat-ICC-L+B/actin-rat_5(Chemiluminescence).tif]

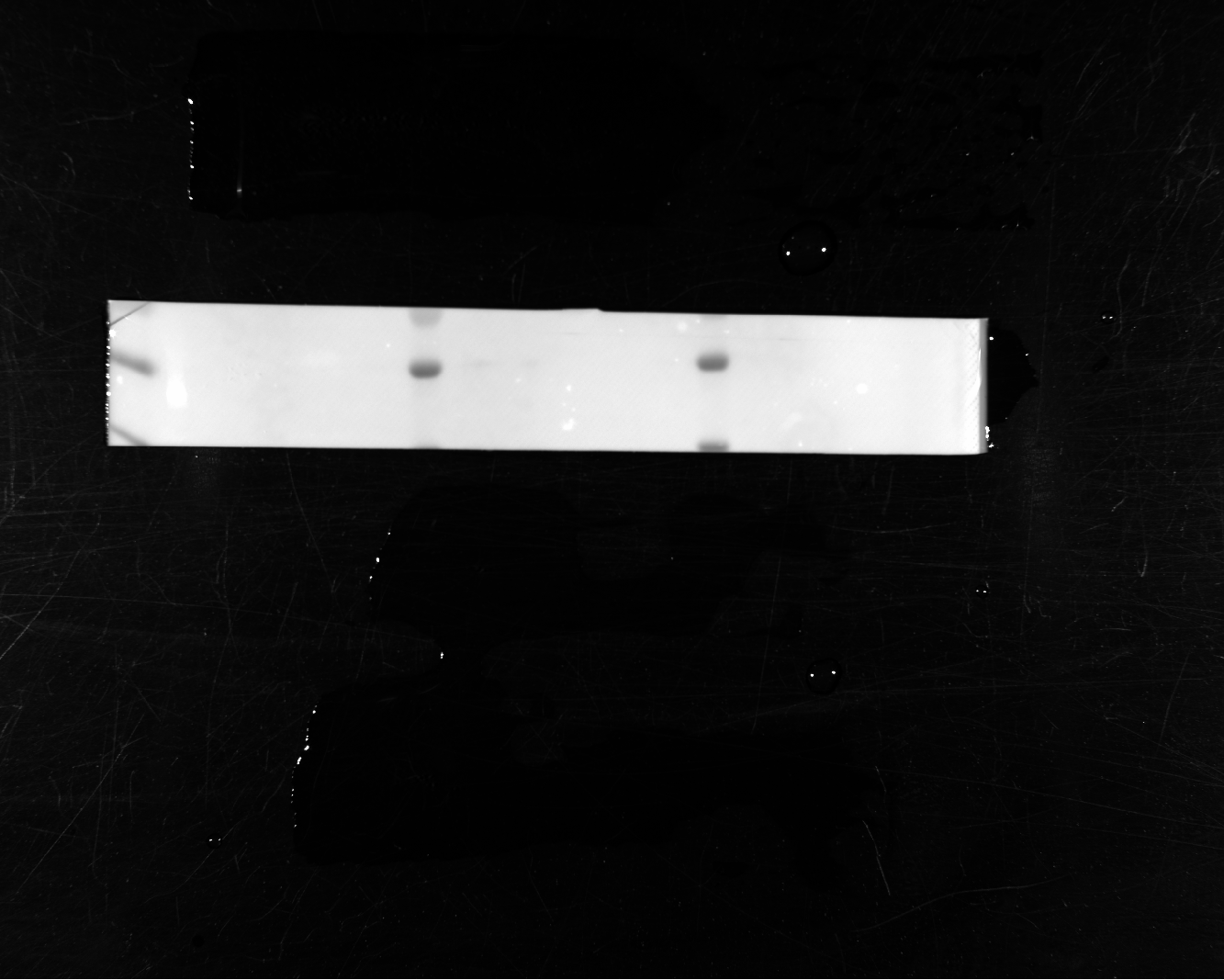

Supplement: Supplementary file 8 [file DataSheet6.ZIP › rat-ICC-L+B/actin-rat_5(Colorimetric).tif]

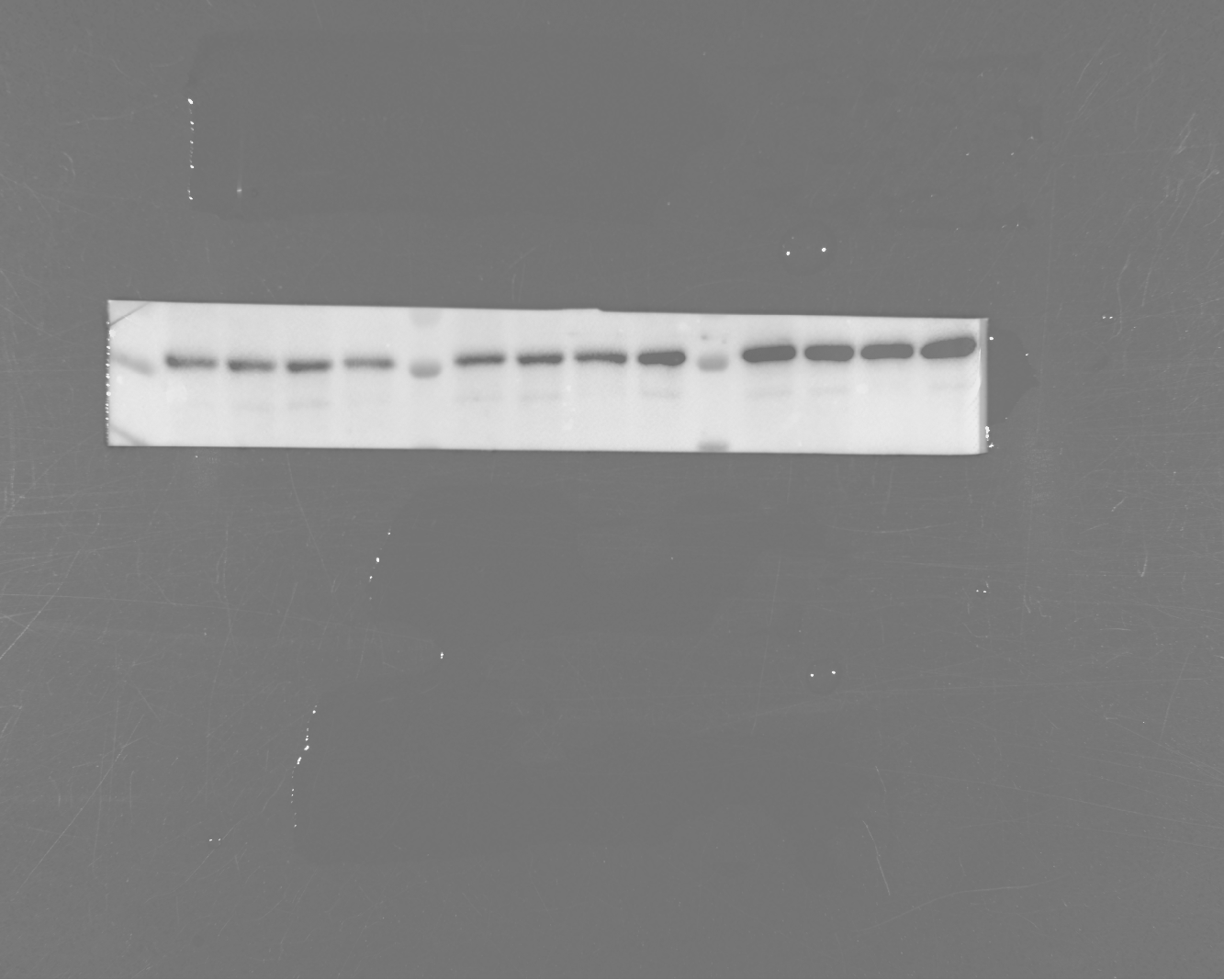

Supplement: Supplementary file 8 [file DataSheet6.ZIP › rat-ICC-L+B/actin-rat_5(Composite).tif]

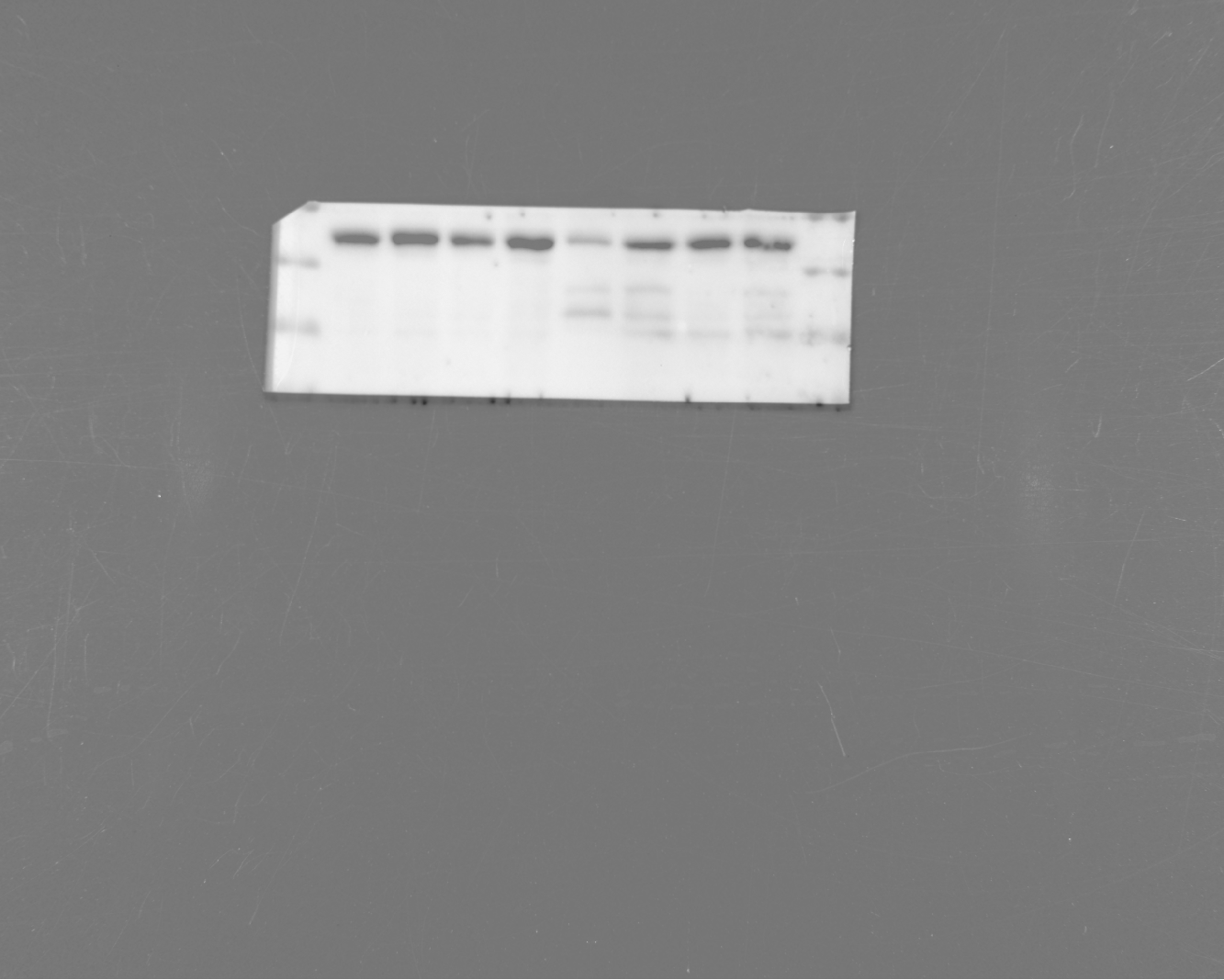

Supplement: Supplementary file 8 [file DataSheet6.ZIP › rat-ICC-L+B/anorectal 2021-01-27 10h16m31s(Composite).tif]

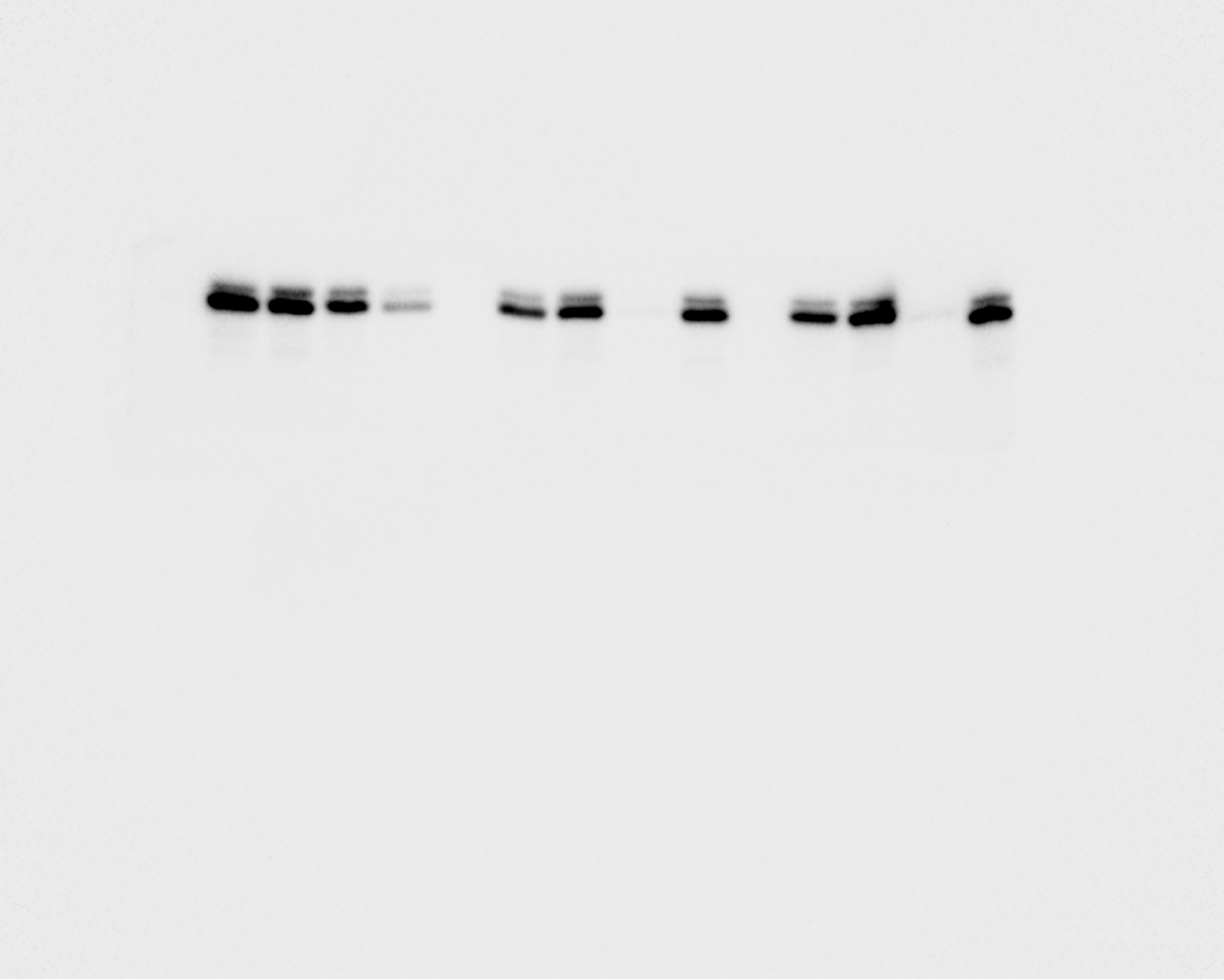

Supplement: Supplementary file 8 [file DataSheet6.ZIP › rat-ICC-L+B/anorectal 2021-01-27 10h41m04s(Chemiluminescence).tif]

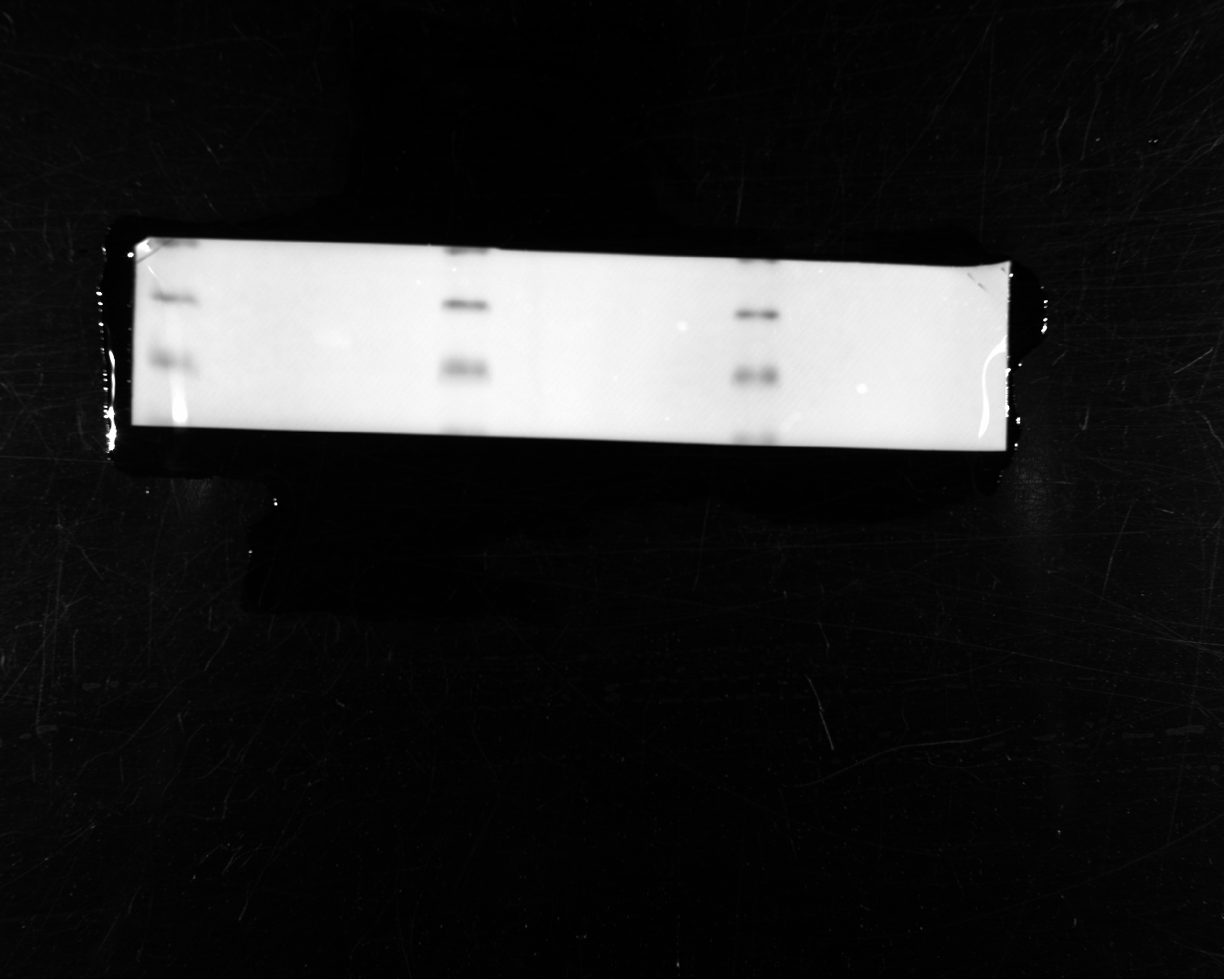

Supplement: Supplementary file 8 [file DataSheet6.ZIP › rat-ICC-L+B/anorectal 2021-01-27 10h41m04s(Colorimetric).tif]

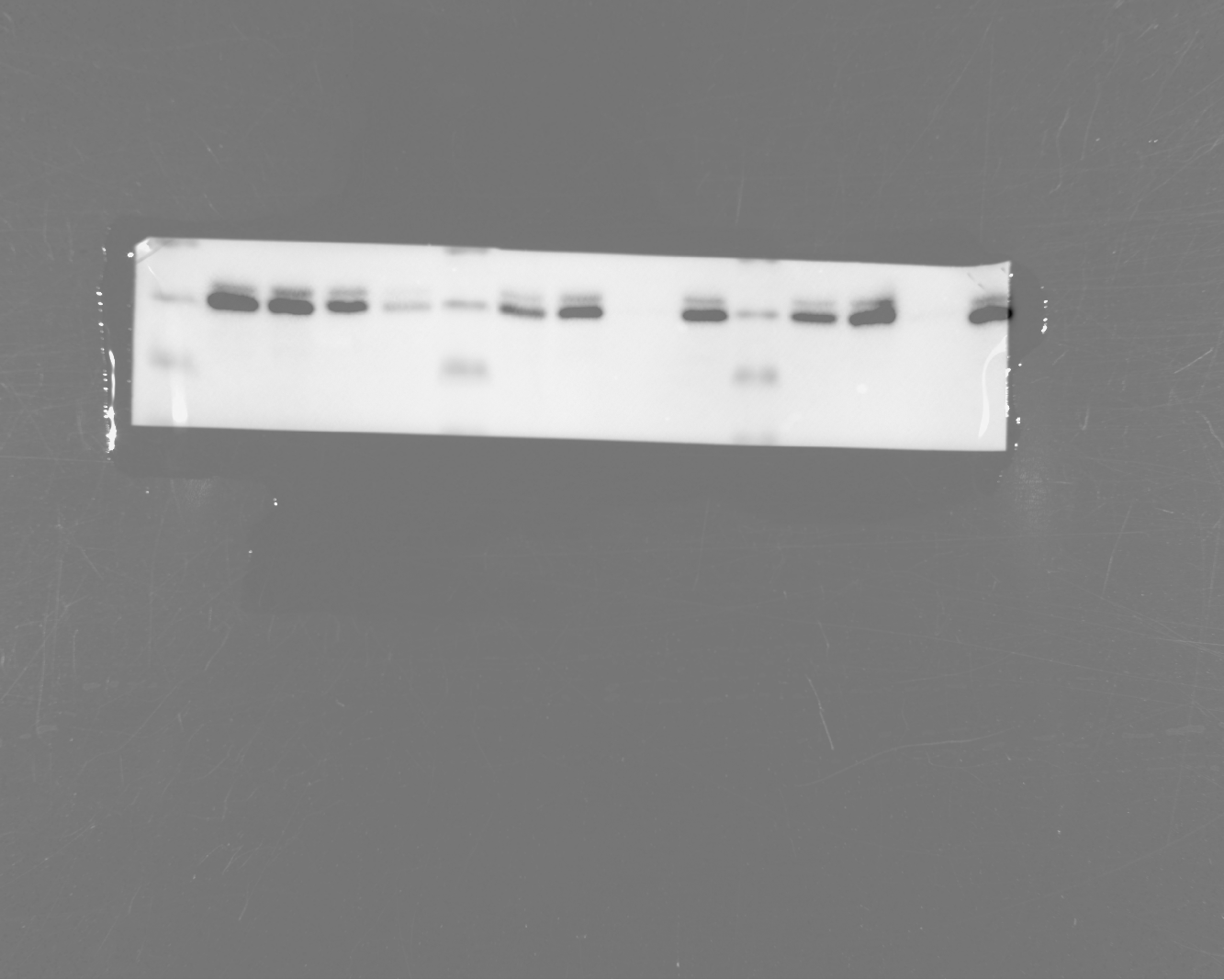

Supplement: Supplementary file 8 [file DataSheet6.ZIP › rat-ICC-L+B/anorectal 2021-01-27 10h41m04s(Composite).tif]

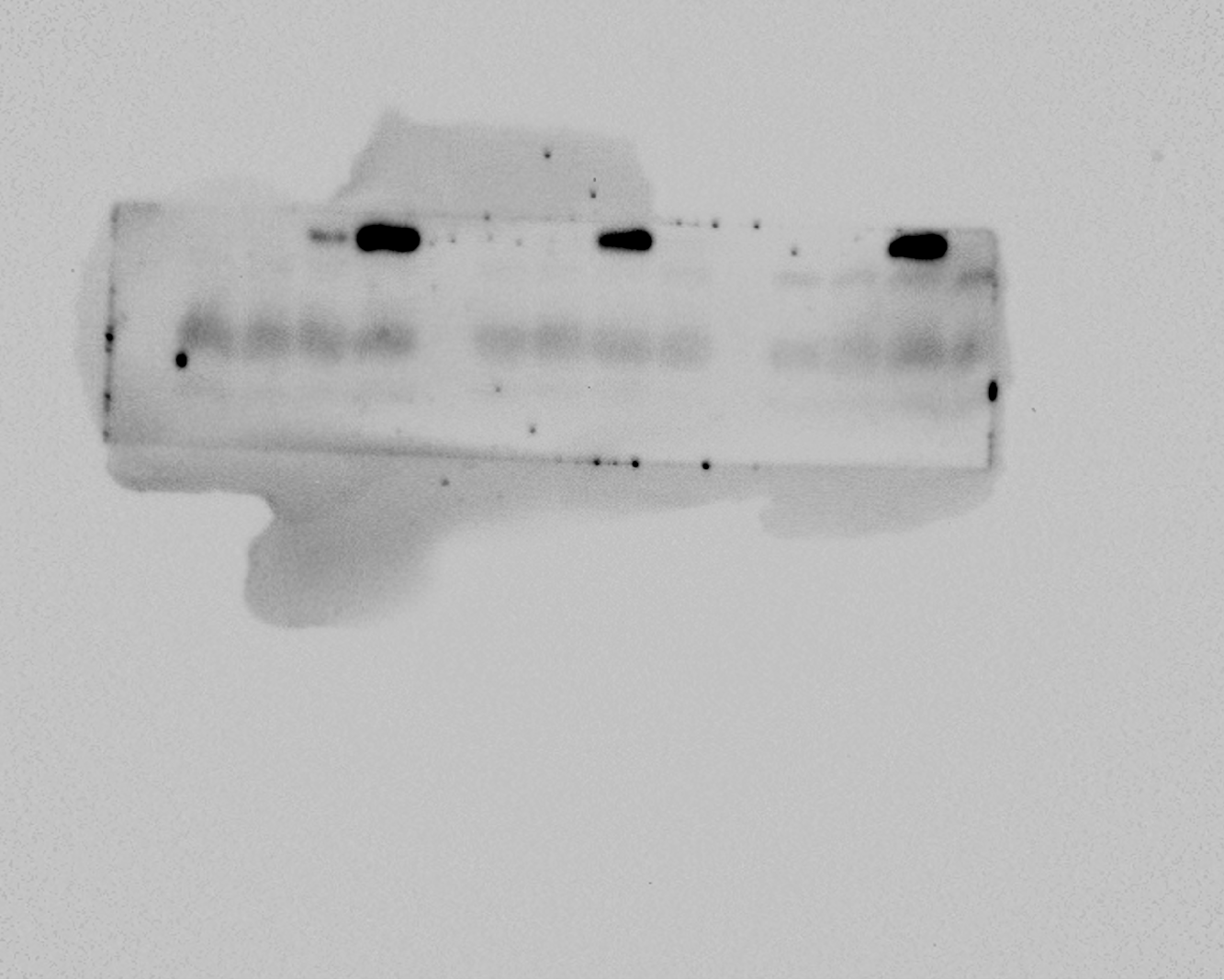

Supplement: Supplementary file 8 [file DataSheet6.ZIP › rat-ICC-L+B/anorectal 2021-01-27 10h45m27s(Chemiluminescence).tif]

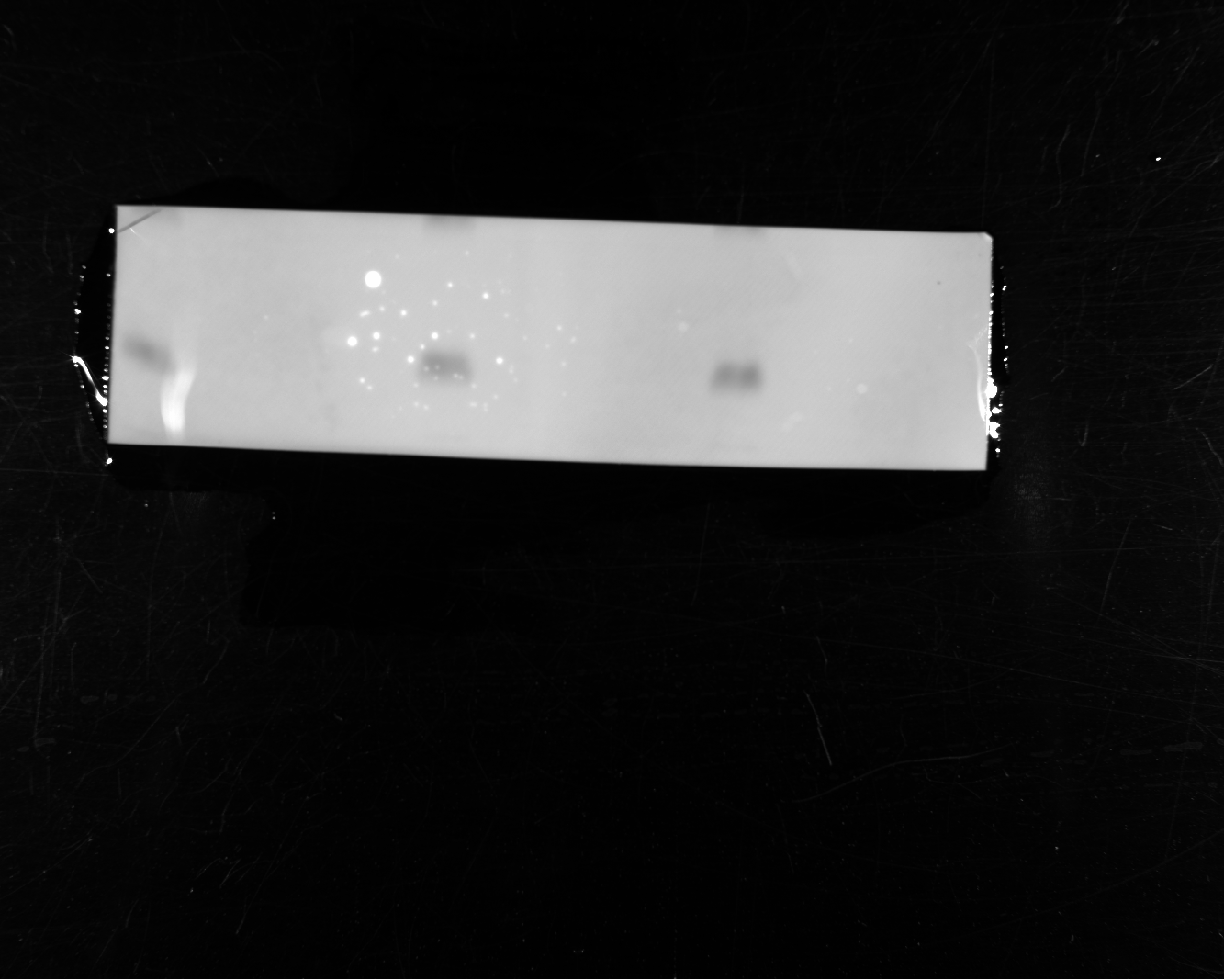

Supplement: Supplementary file 8 [file DataSheet6.ZIP › rat-ICC-L+B/anorectal 2021-01-27 10h45m27s(Colorimetric).tif]

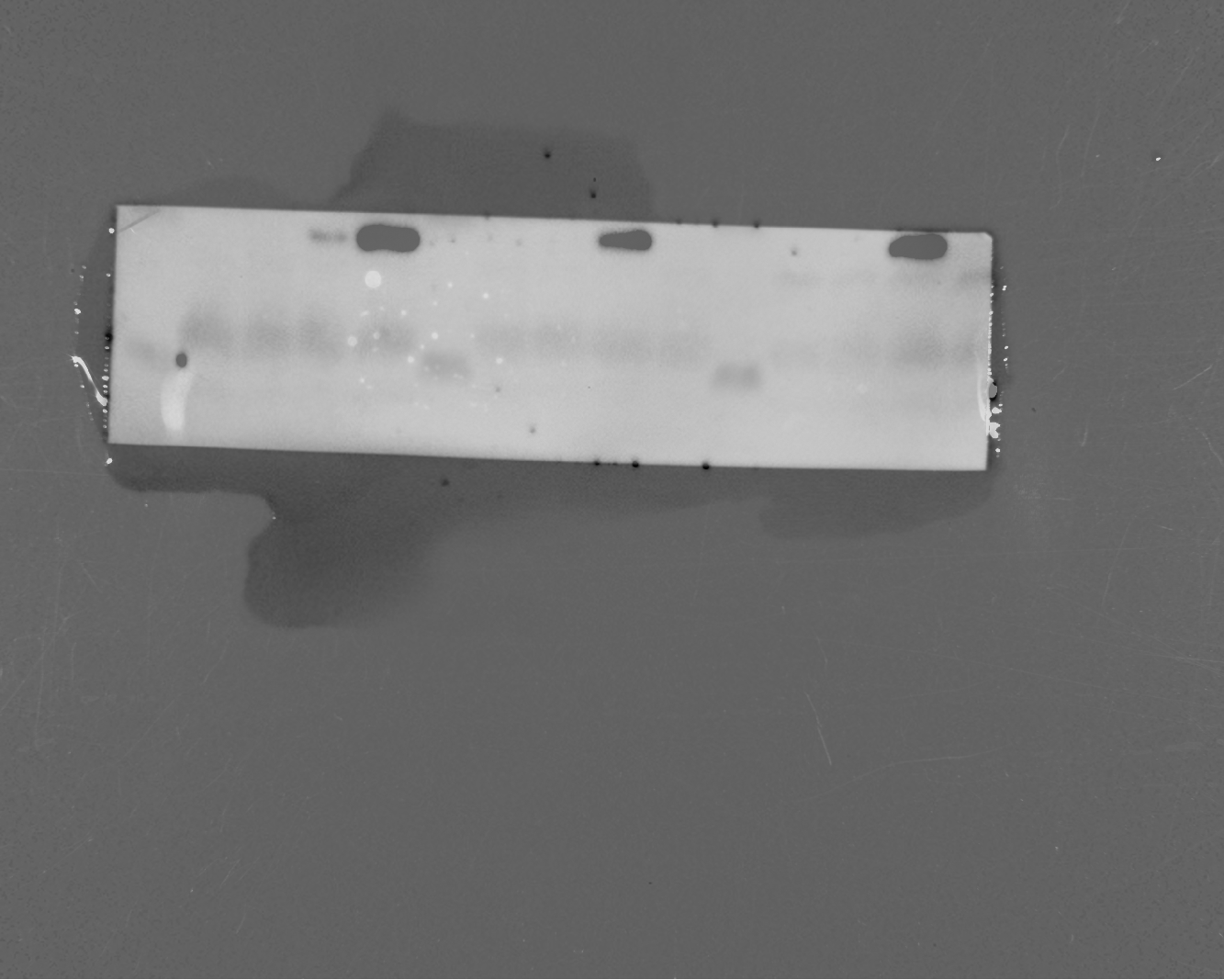

Supplement: Supplementary file 8 [file DataSheet6.ZIP › rat-ICC-L+B/anorectal 2021-01-27 10h45m27s(Composite).tif]

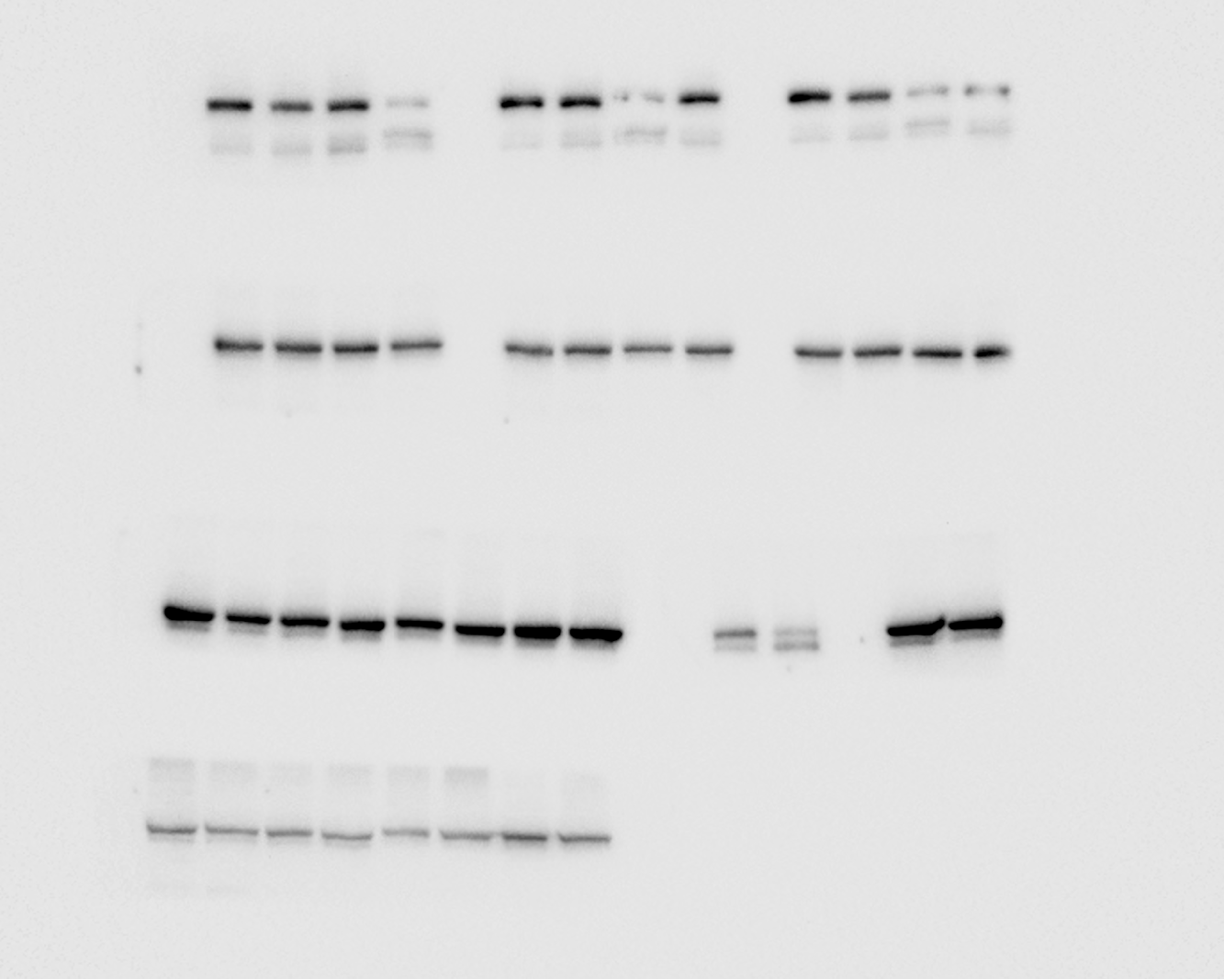

Supplement: Supplementary file 8 [file DataSheet6.ZIP › rat-ICC-L+B/anorectal 2021-01-27 10h58m11s(Chemiluminescence).tif]

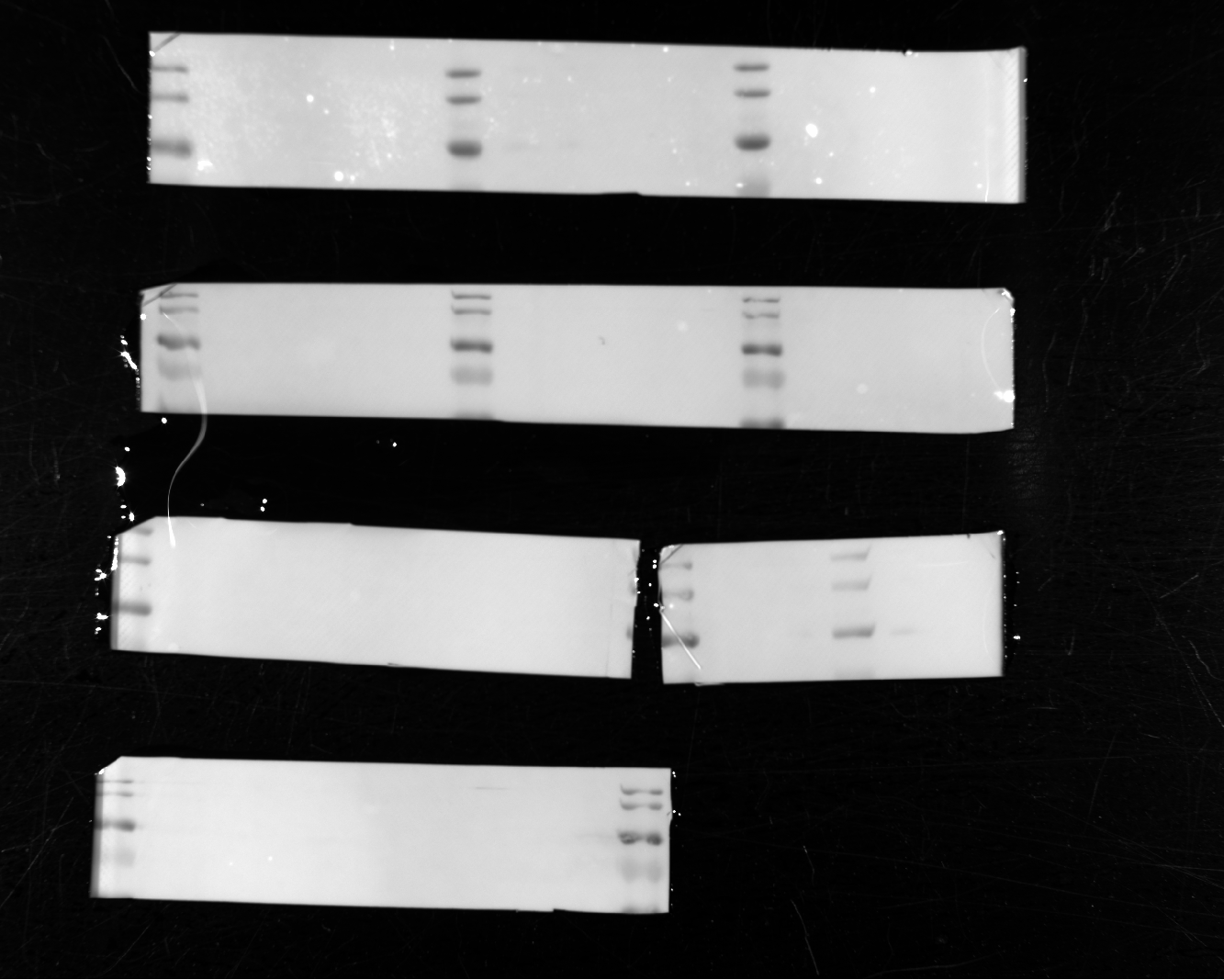

Supplement: Supplementary file 8 [file DataSheet6.ZIP › rat-ICC-L+B/anorectal 2021-01-27 10h58m11s(Colorimetric).tif]

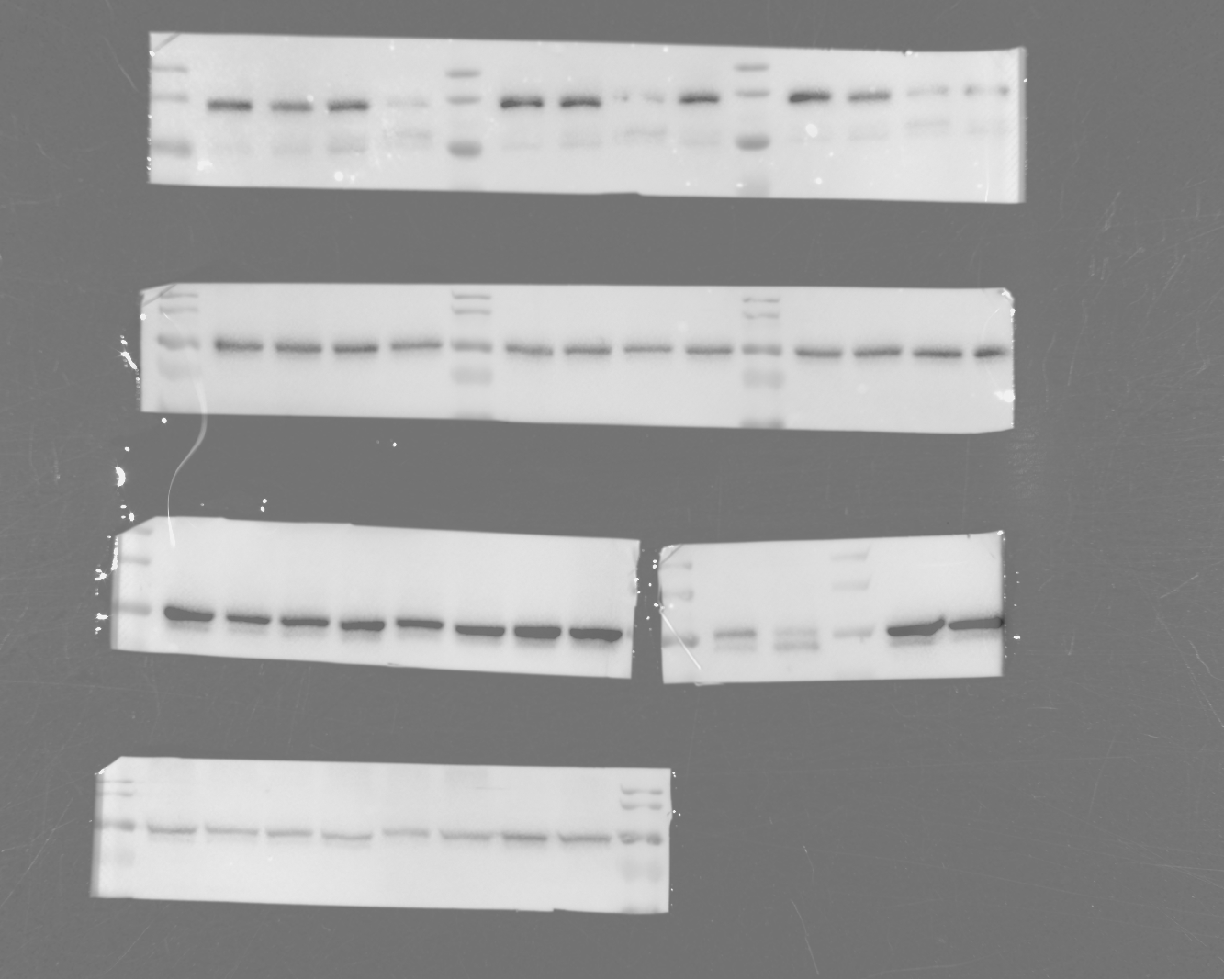

Supplement: Supplementary file 8 [file DataSheet6.ZIP › rat-ICC-L+B/anorectal 2021-01-27 10h58m11s(Composite).tif]

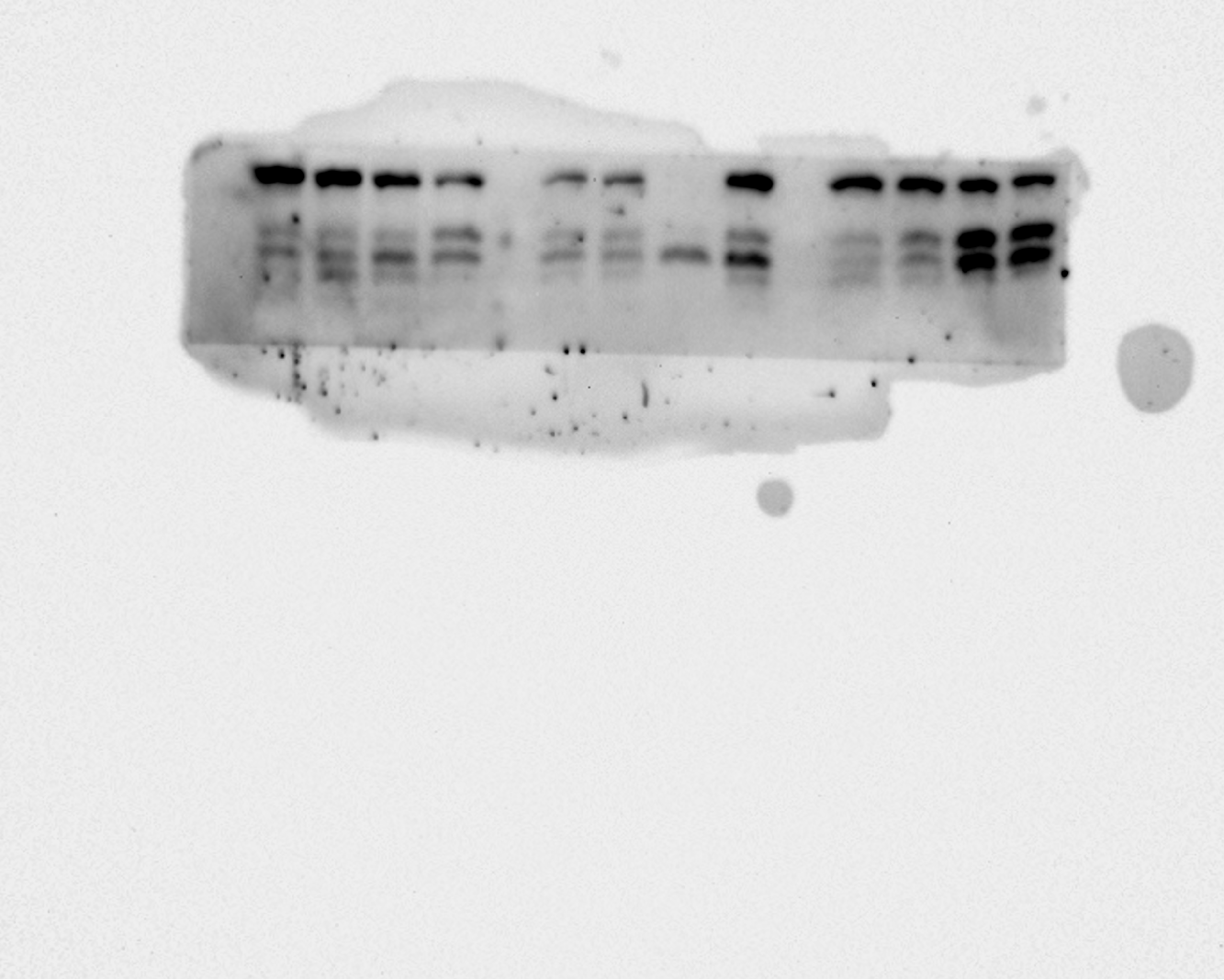

Supplement: Supplementary file 8 [file DataSheet6.ZIP › rat-ICC-L+B/anorectal 2021-01-28 14h33m19s(Chemiluminescence).tif]

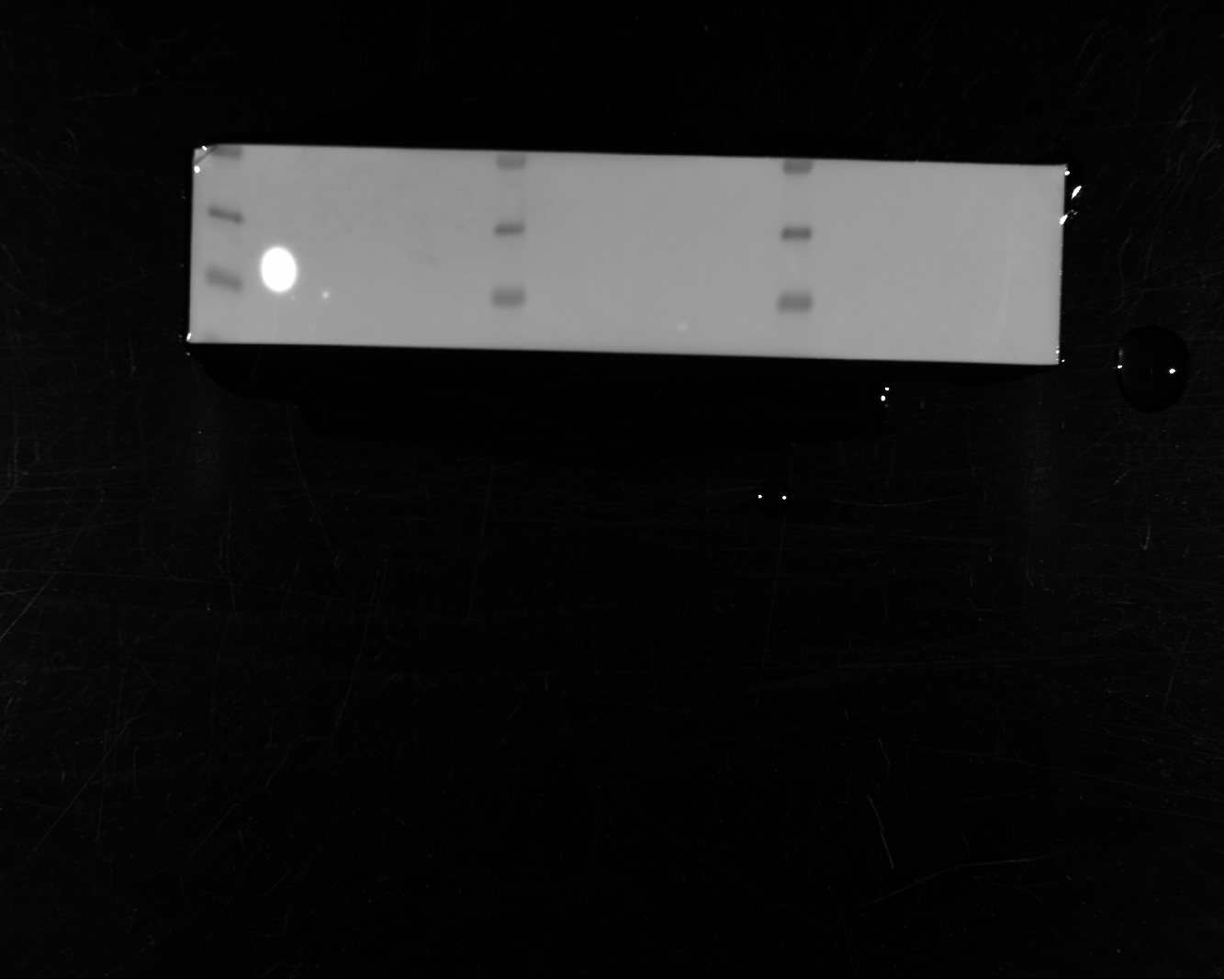

Supplement: Supplementary file 8 [file DataSheet6.ZIP › rat-ICC-L+B/anorectal 2021-01-28 14h33m19s(Colorimetric).tif]

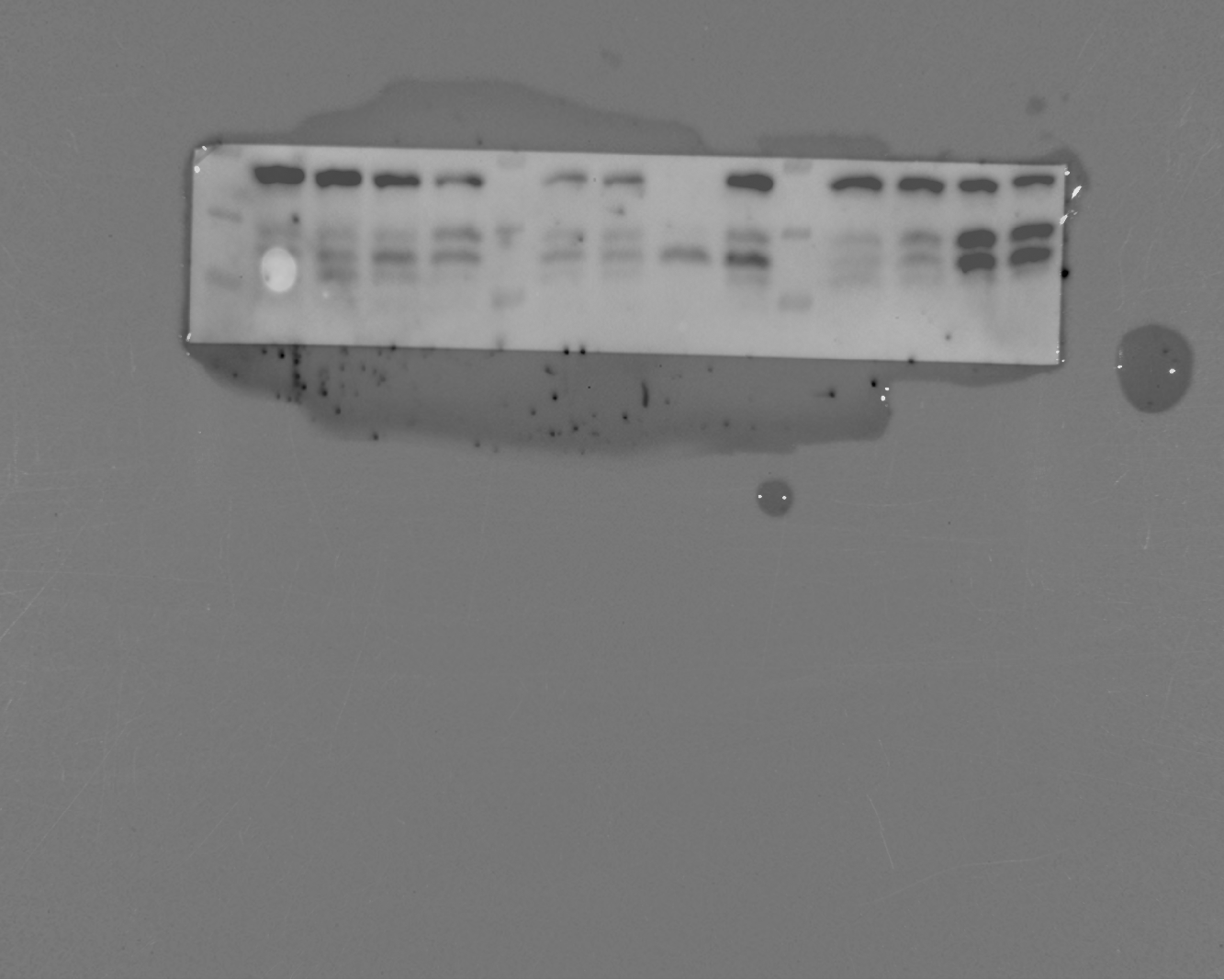

Supplement: Supplementary file 8 [file DataSheet6.ZIP › rat-ICC-L+B/anorectal 2021-01-28 14h33m19s(Composite).tif]

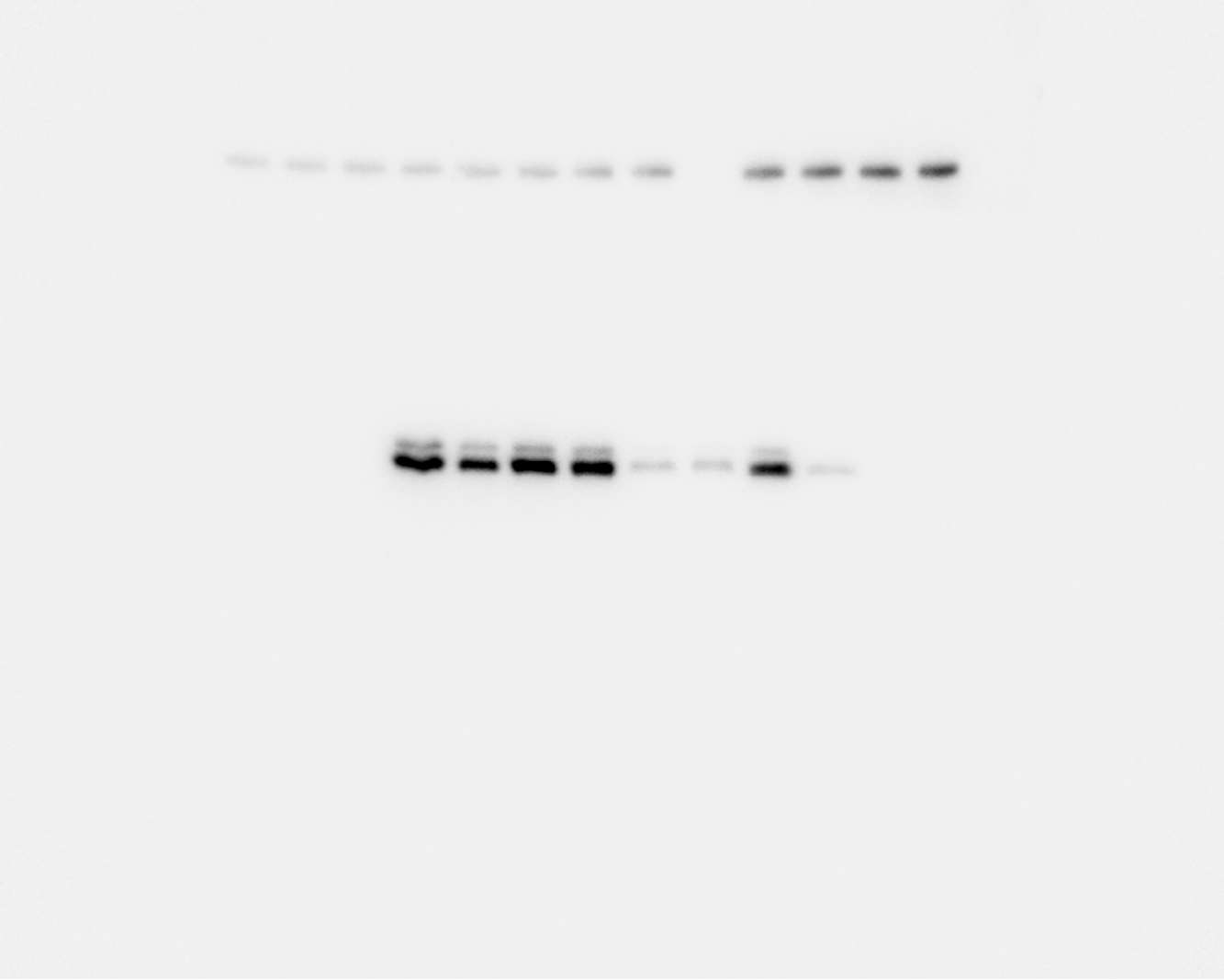

Supplement: Supplementary file 8 [file DataSheet6.ZIP › rat-ICC-L+B/anorectal 2021-01-28 15h06m08s(Chemiluminescence).tif]

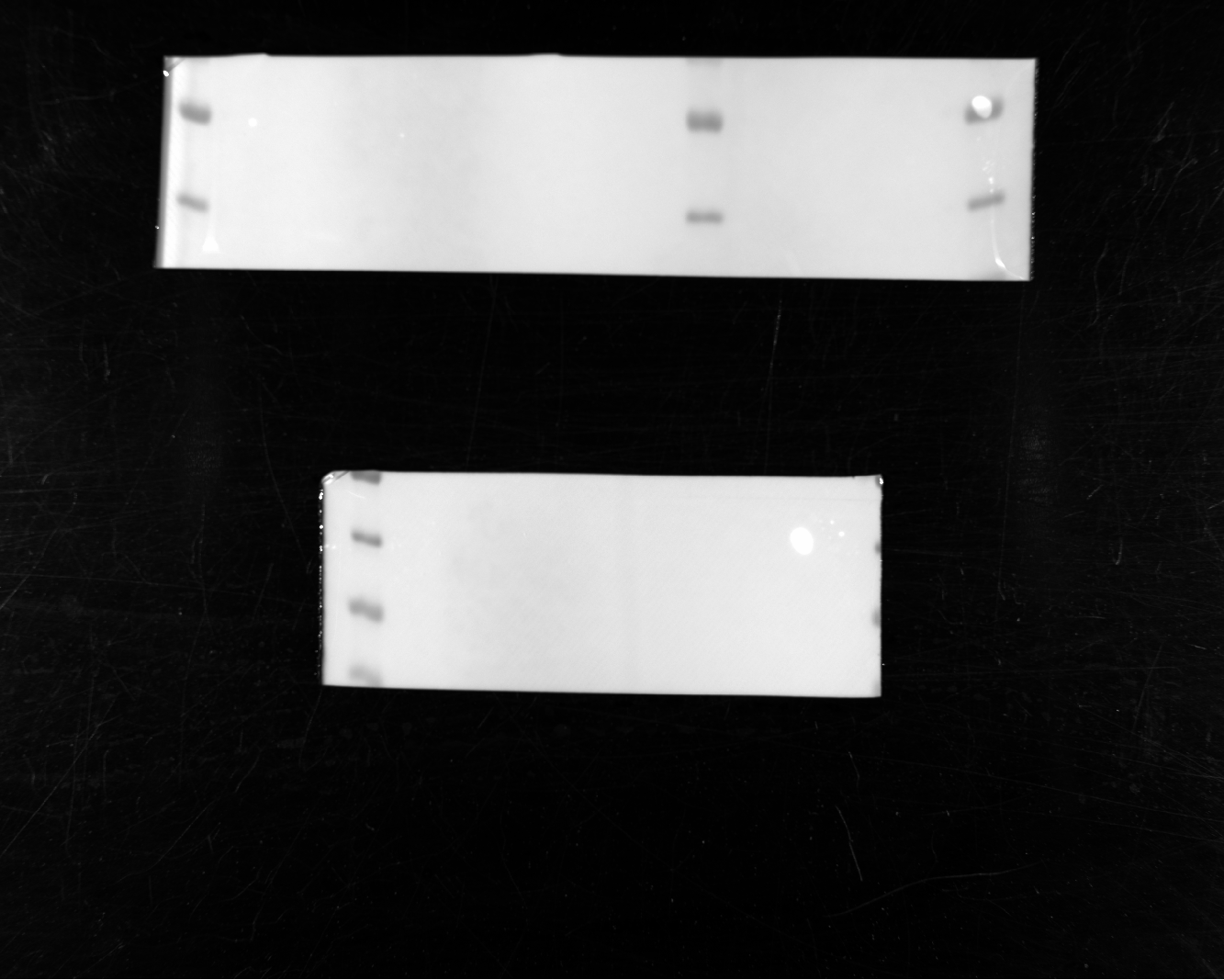

Supplement: Supplementary file 8 [file DataSheet6.ZIP › rat-ICC-L+B/anorectal 2021-01-28 15h06m08s(Colorimetric).tif]

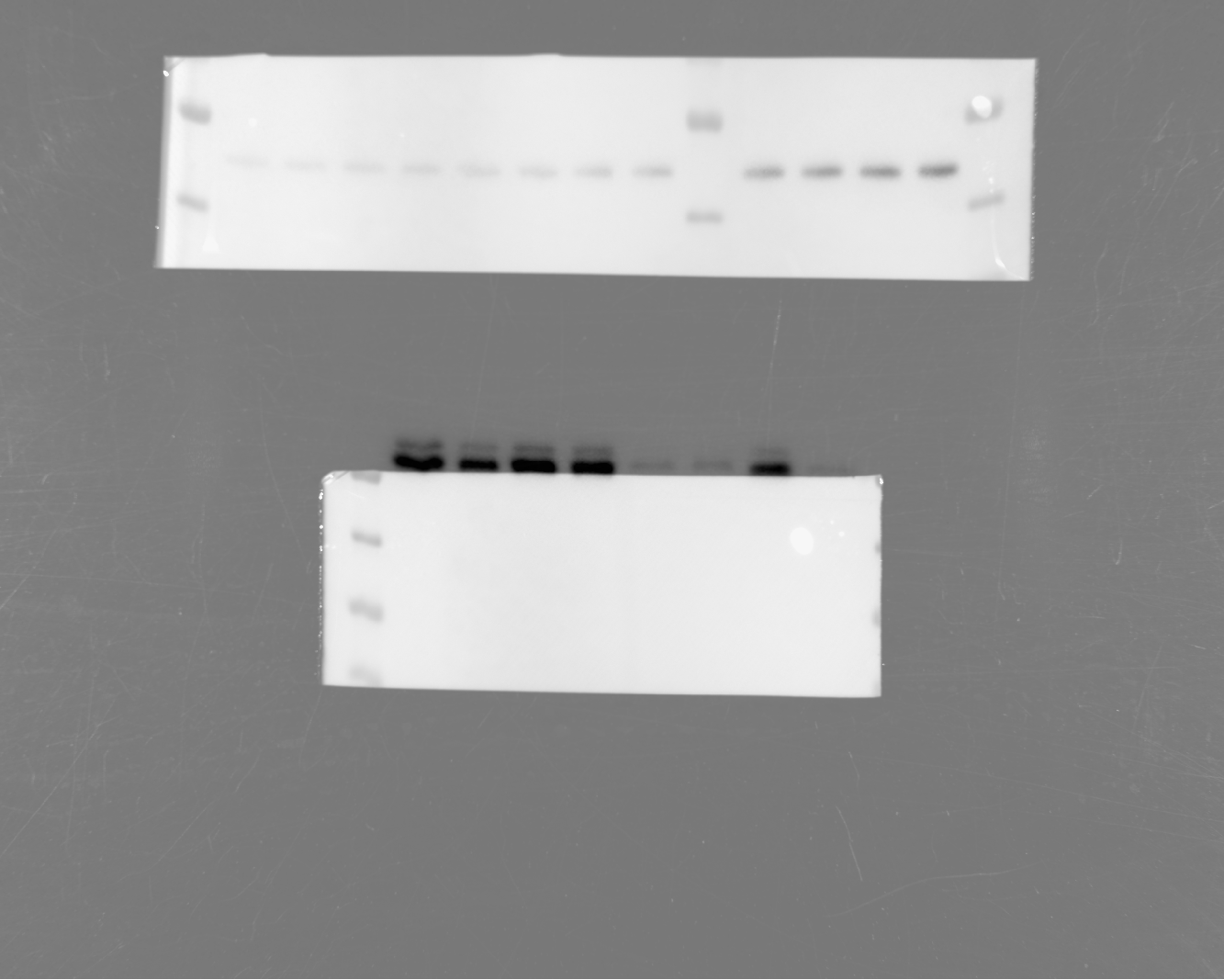

Supplement: Supplementary file 8 [file DataSheet6.ZIP › rat-ICC-L+B/anorectal 2021-01-28 15h06m08s(Composite).tif]

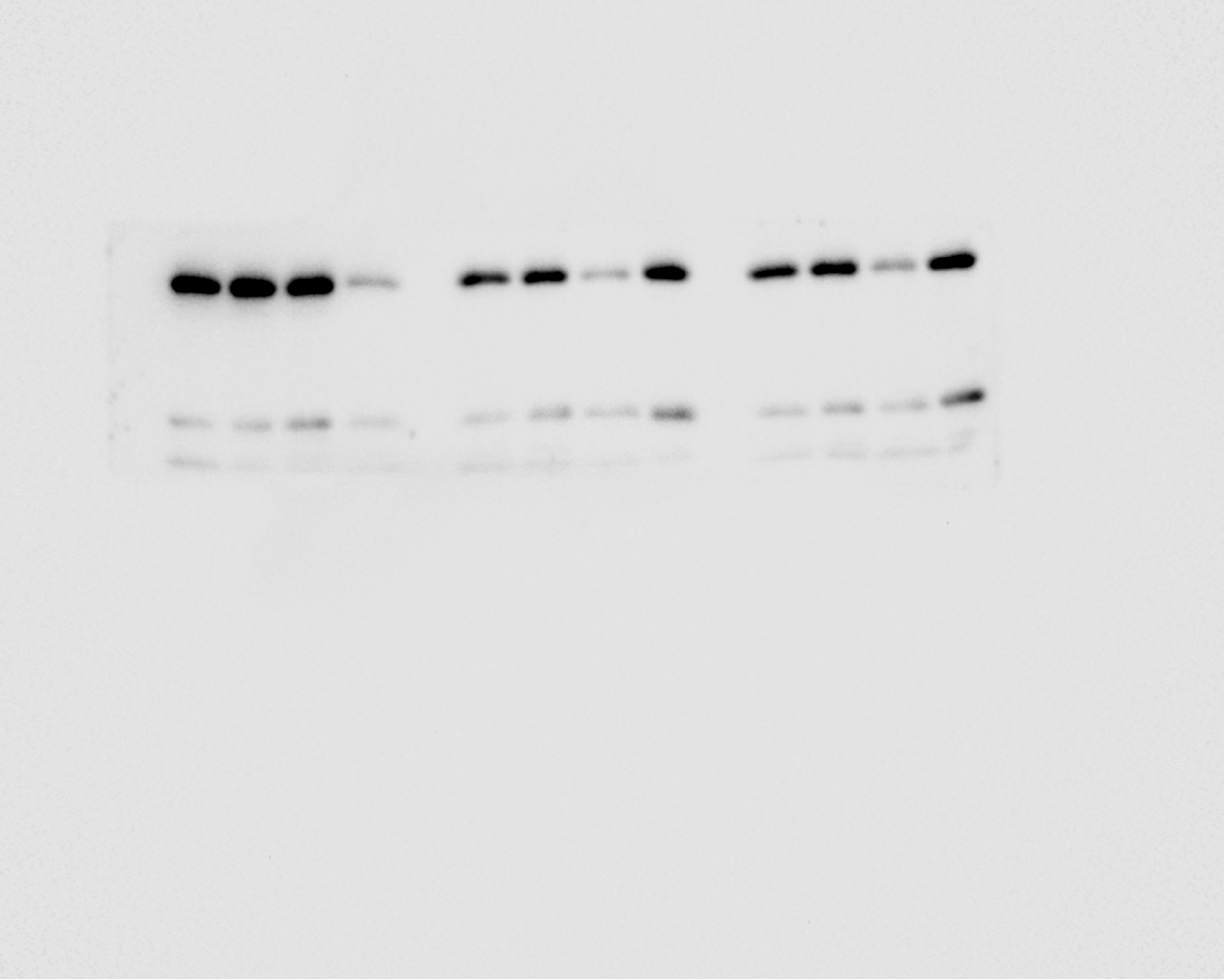

Supplement: Supplementary file 8 [file DataSheet6.ZIP › rat-ICC-L+B/bcl2_3(Chemiluminescence).tif]

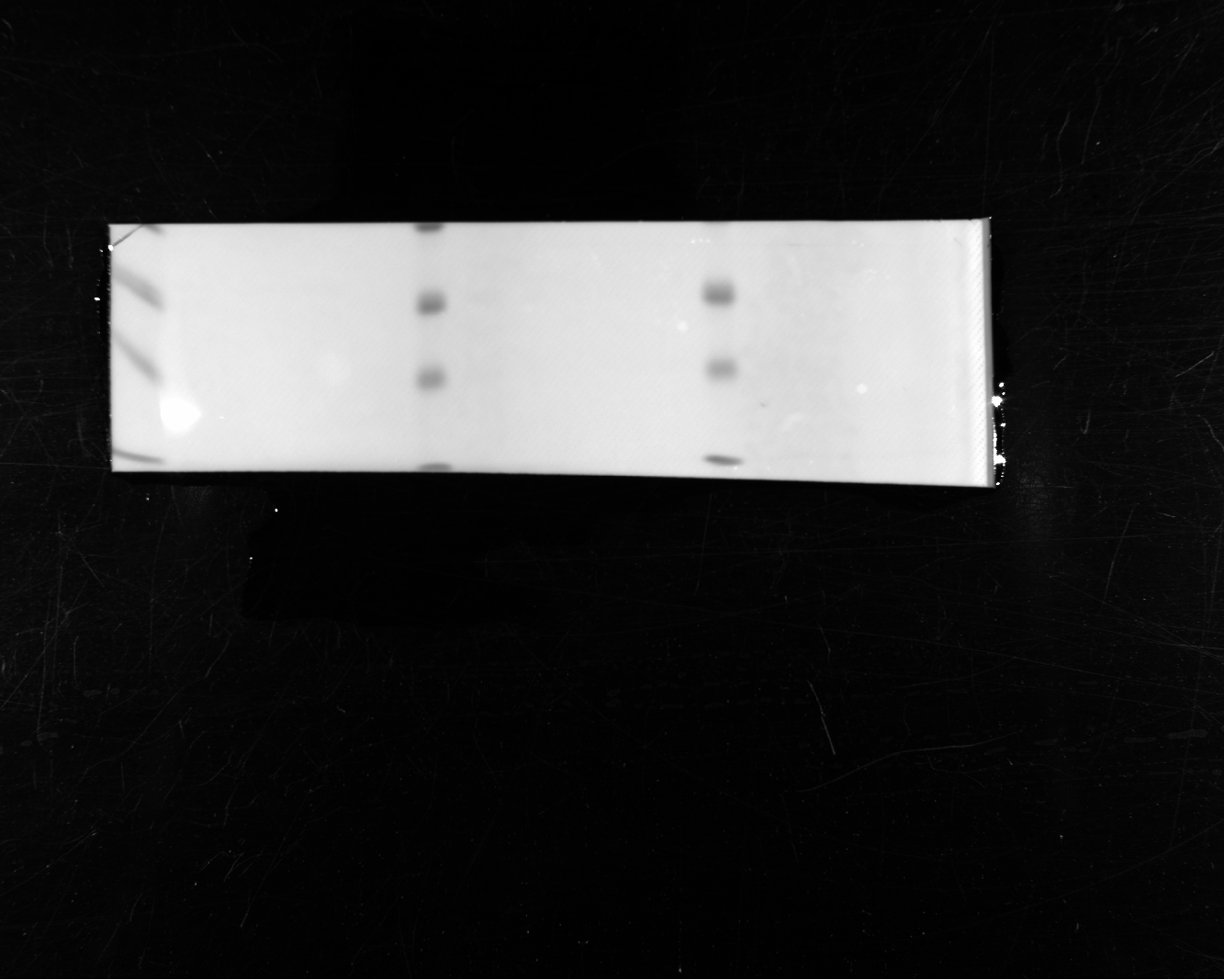

Supplement: Supplementary file 8 [file DataSheet6.ZIP › rat-ICC-L+B/bcl2_3(Colorimetric).tif]

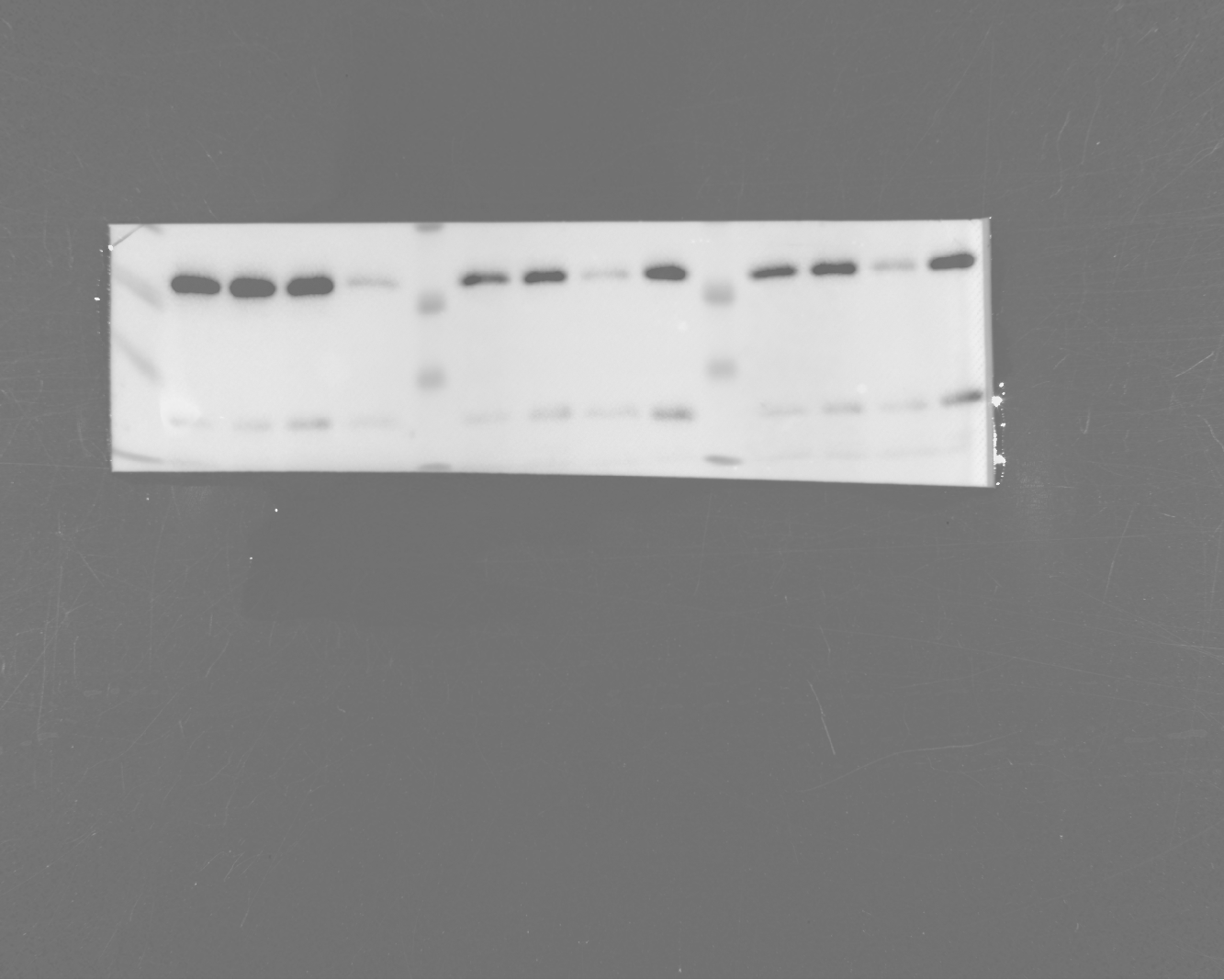

Supplement: Supplementary file 8 [file DataSheet6.ZIP › rat-ICC-L+B/bcl2_3(Composite).tif]

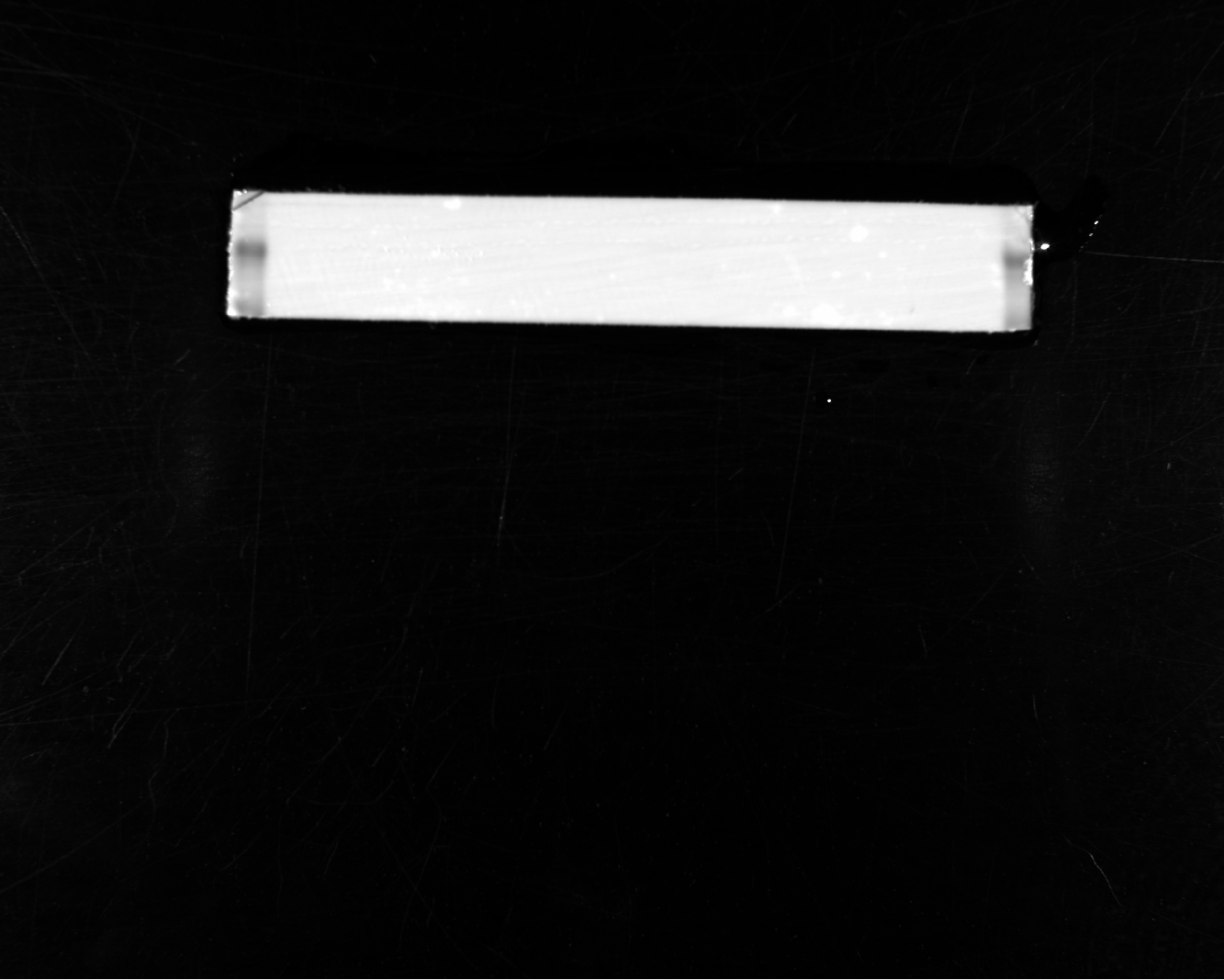

Supplement: Supplementary file 8 [file DataSheet6.ZIP › rat-ICC-L+B/cas3 (1).tif]

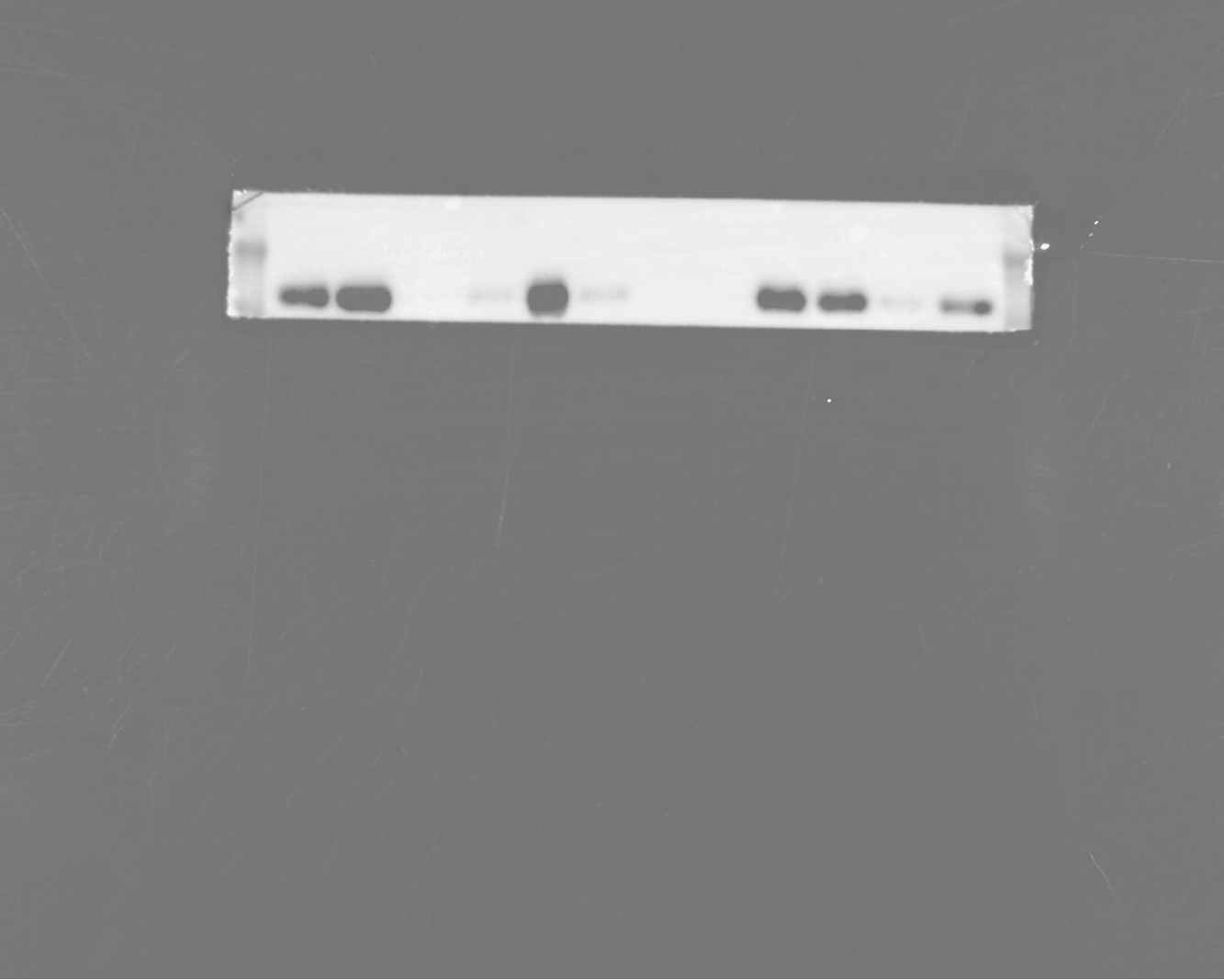

Supplement: Supplementary file 8 [file DataSheet6.ZIP › rat-ICC-L+B/cas3 (2).tif]

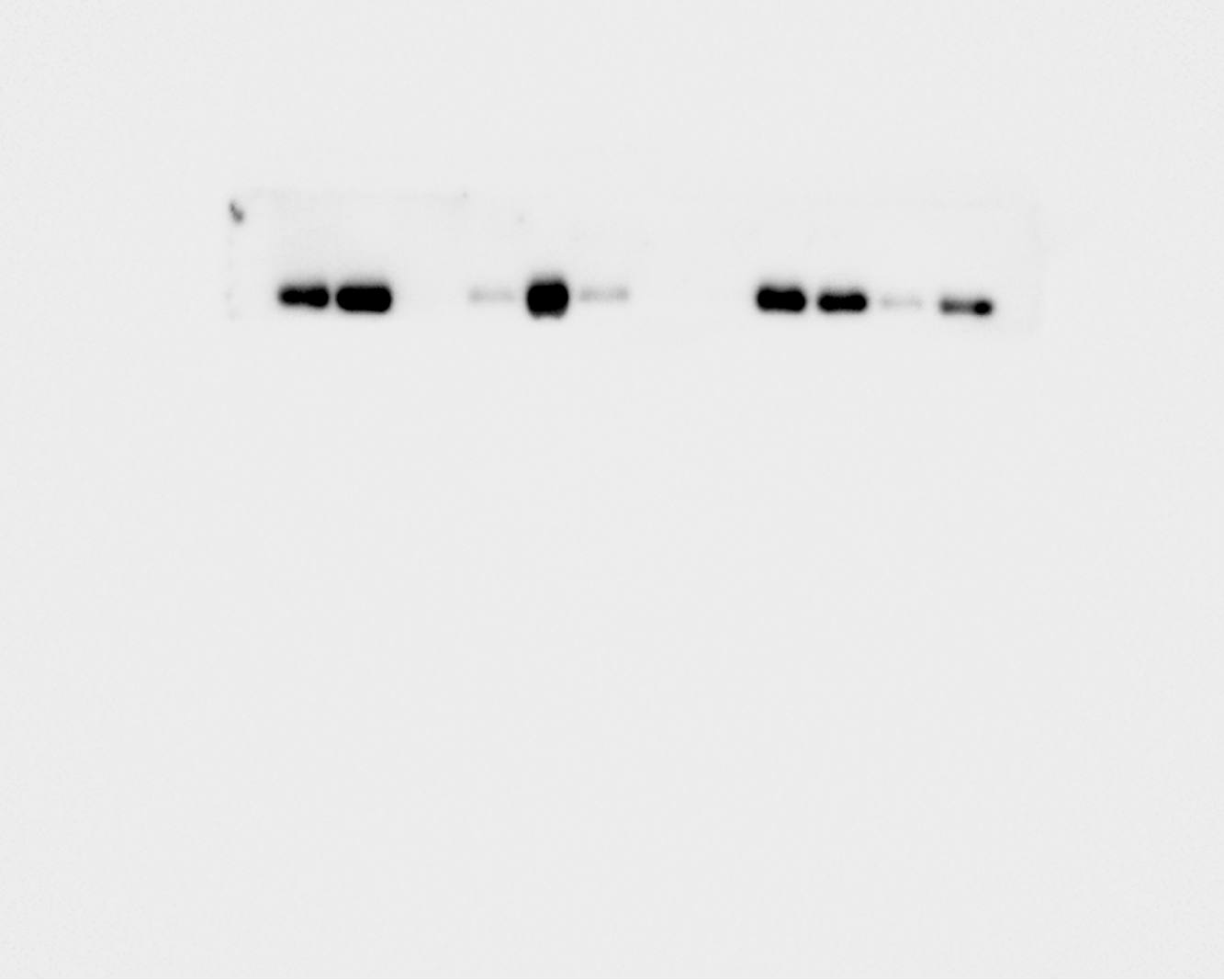

Supplement: Supplementary file 8 [file DataSheet6.ZIP › rat-ICC-L+B/cas3 (3).tif]

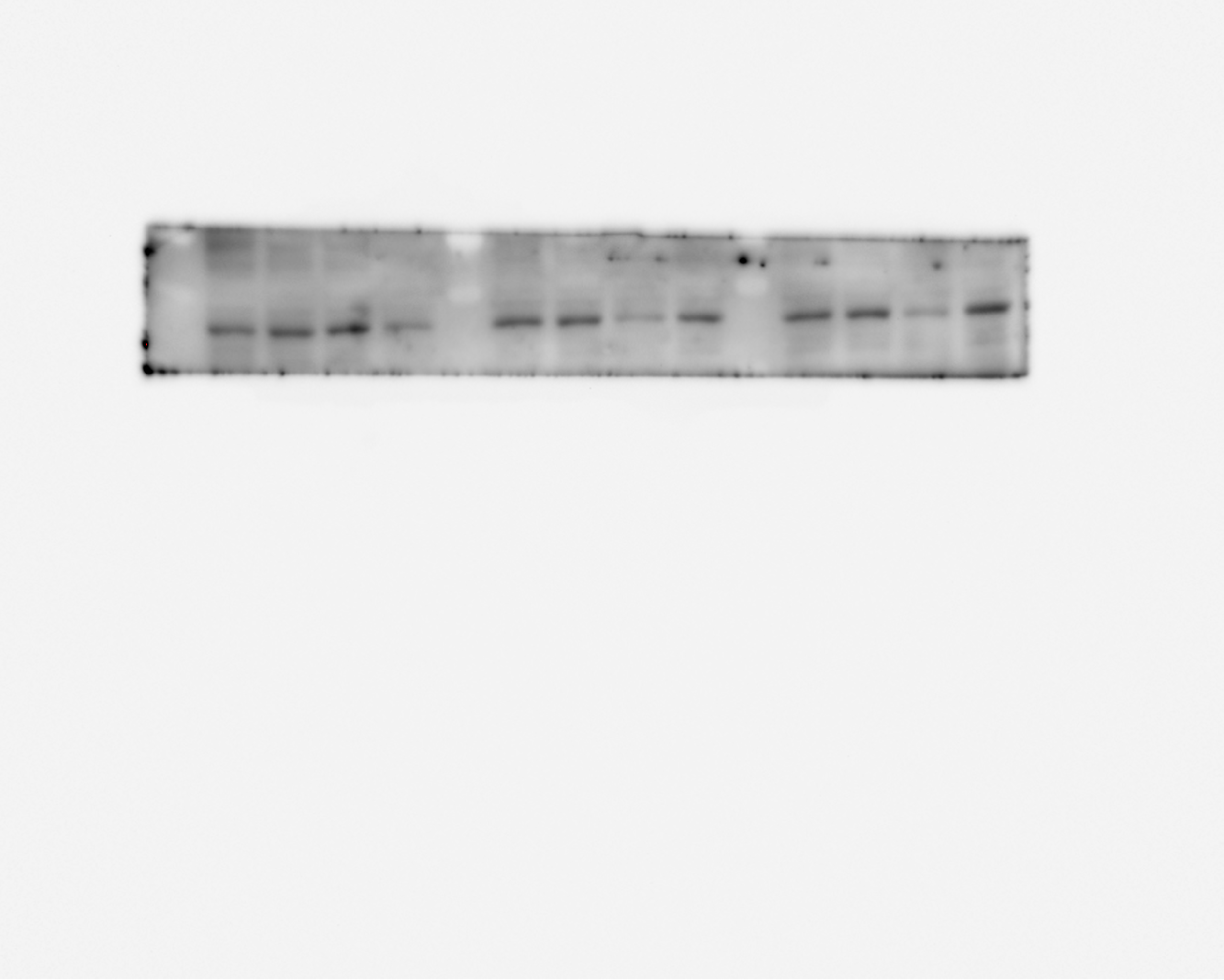

Supplement: Supplementary file 8 [file DataSheet6.ZIP › rat-ICC-L+B/cas9_5(Chemiluminescence).tif]

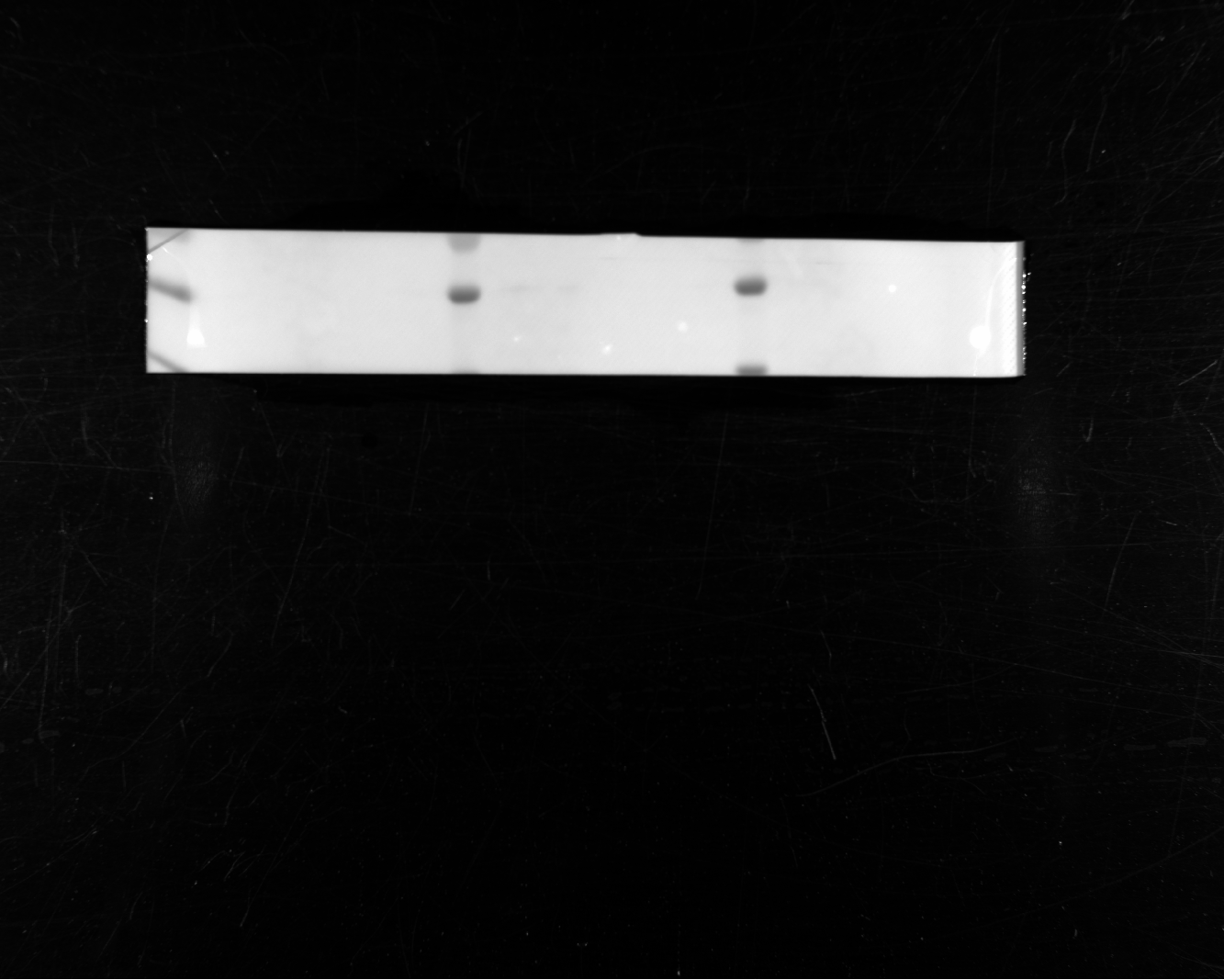

Supplement: Supplementary file 8 [file DataSheet6.ZIP › rat-ICC-L+B/cas9_5(Colorimetric).tif]

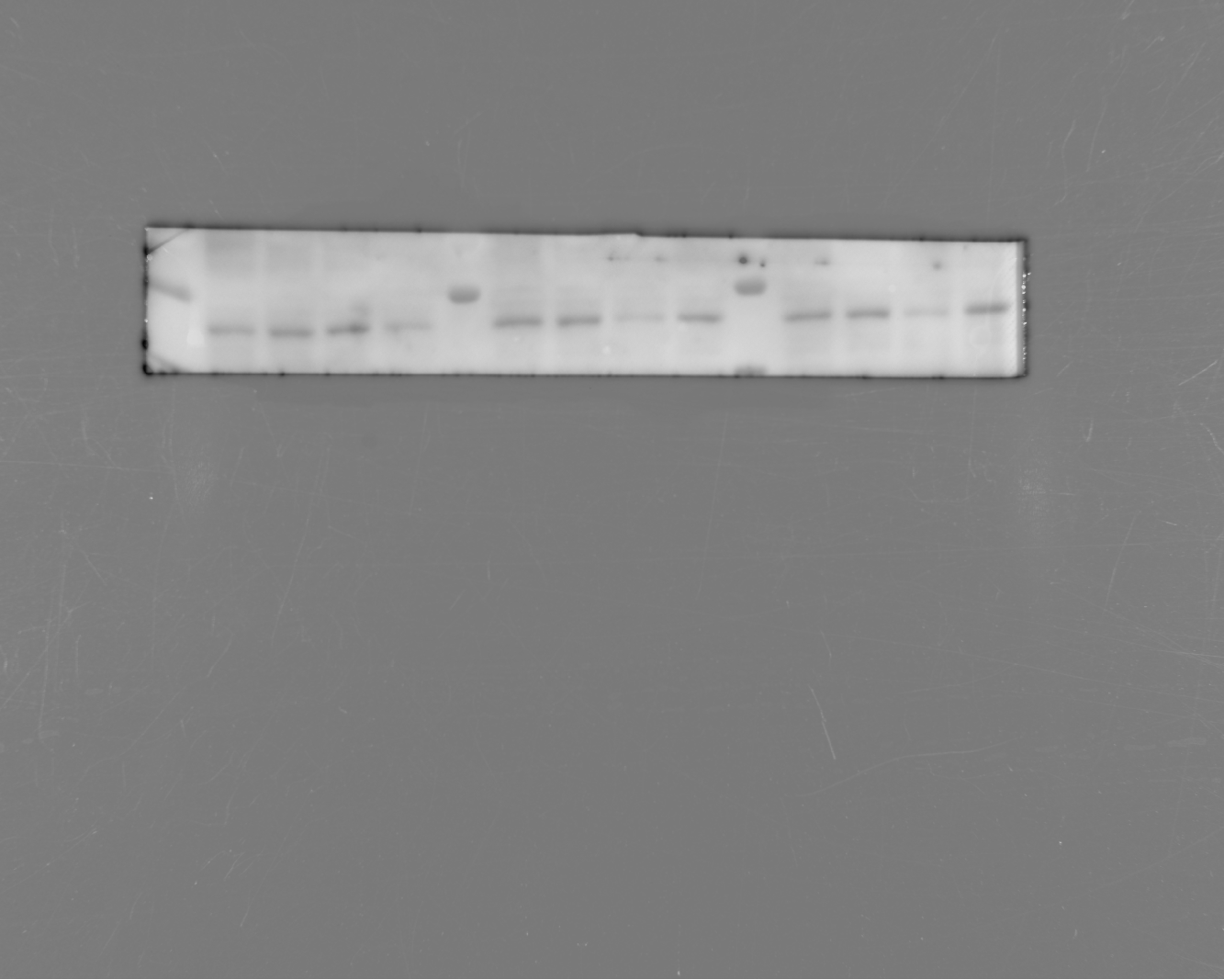

Supplement: Supplementary file 8 [file DataSheet6.ZIP › rat-ICC-L+B/cas9_5(Composite).tif]

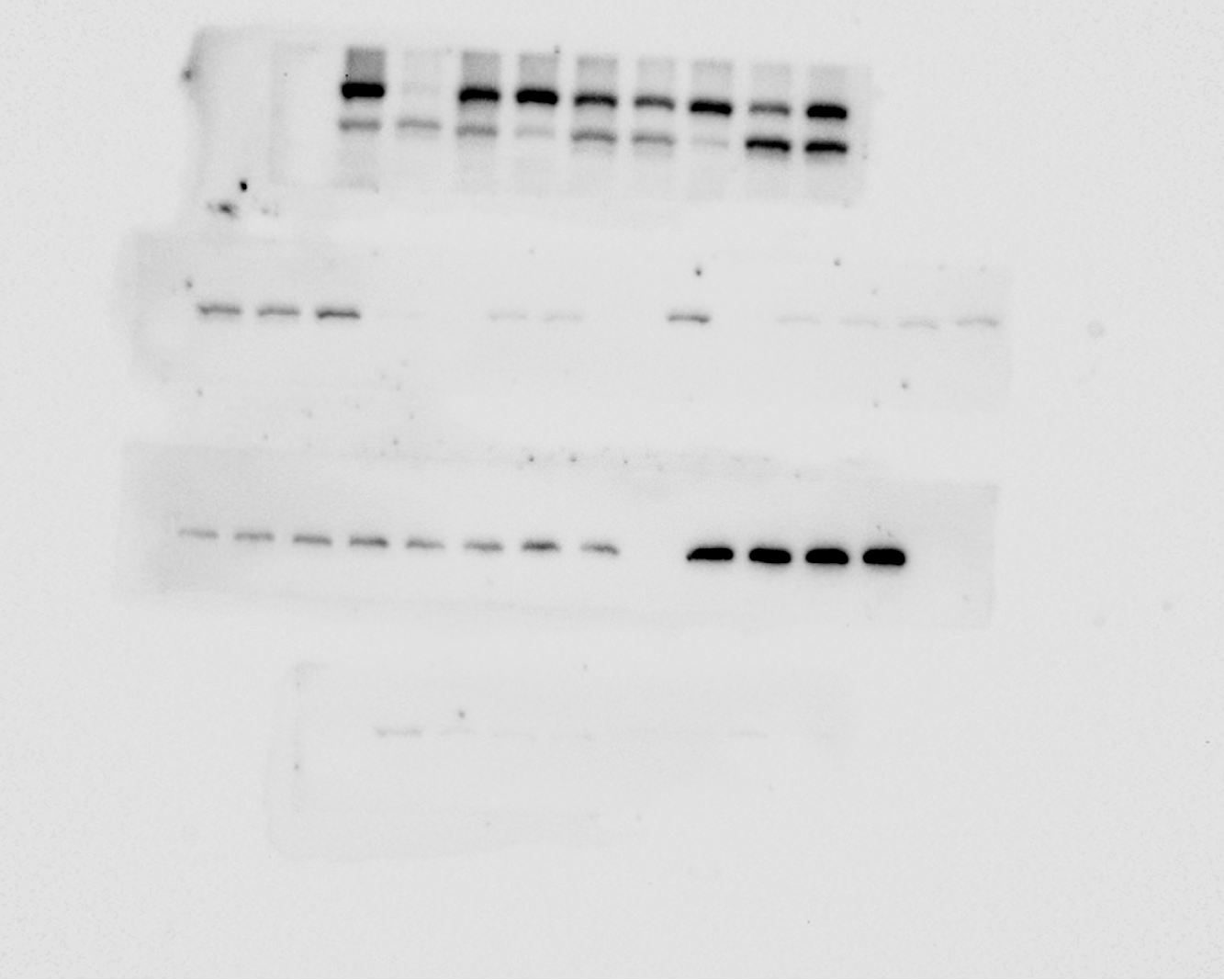

Supplement: Supplementary file 8 [file DataSheet6.ZIP › rat-ICC-L+B/par-pst-st-pst_9(Chemiluminescence).tif]

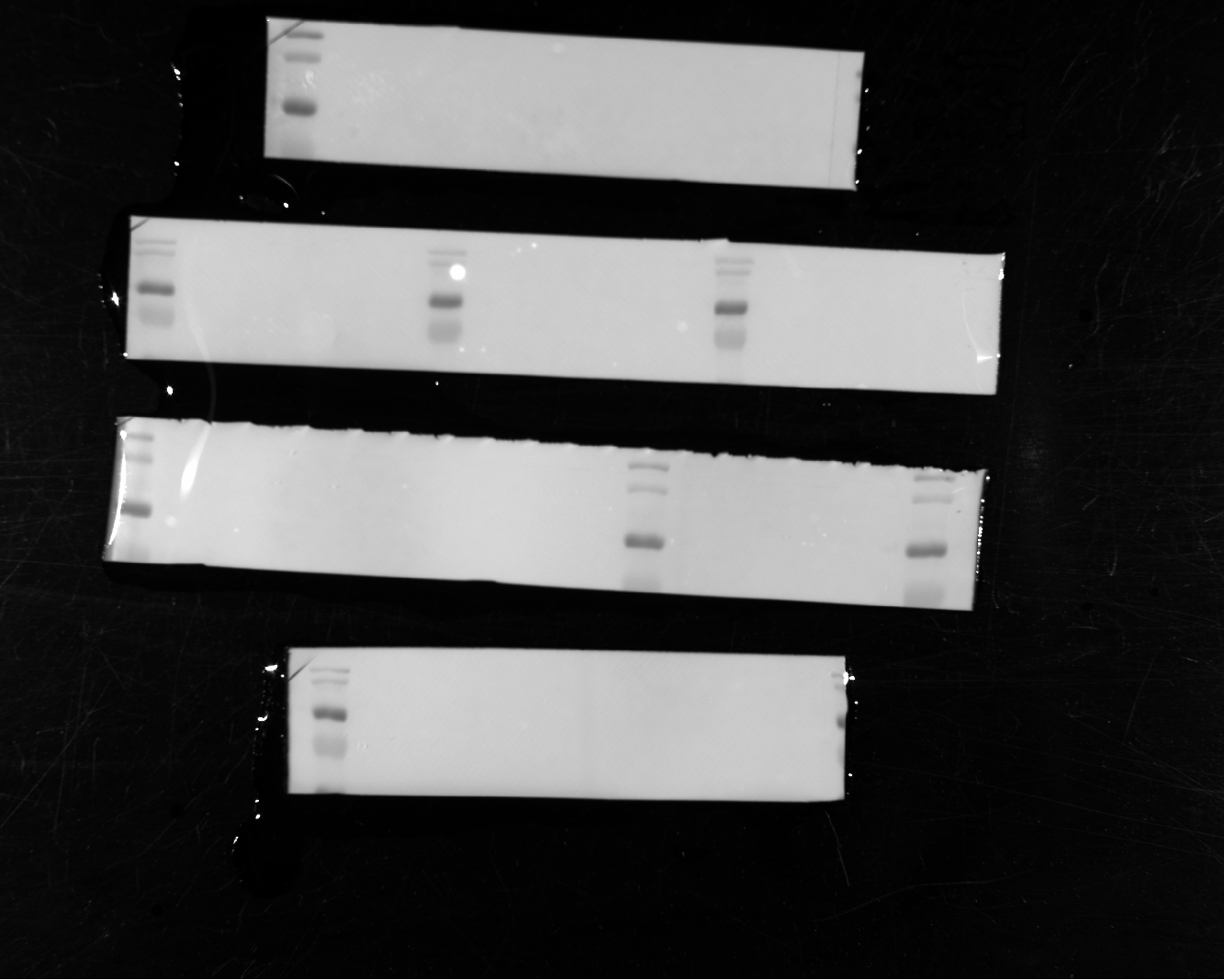

Supplement: Supplementary file 8 [file DataSheet6.ZIP › rat-ICC-L+B/par-pst-st-pst_9(Colorimetric).tif]

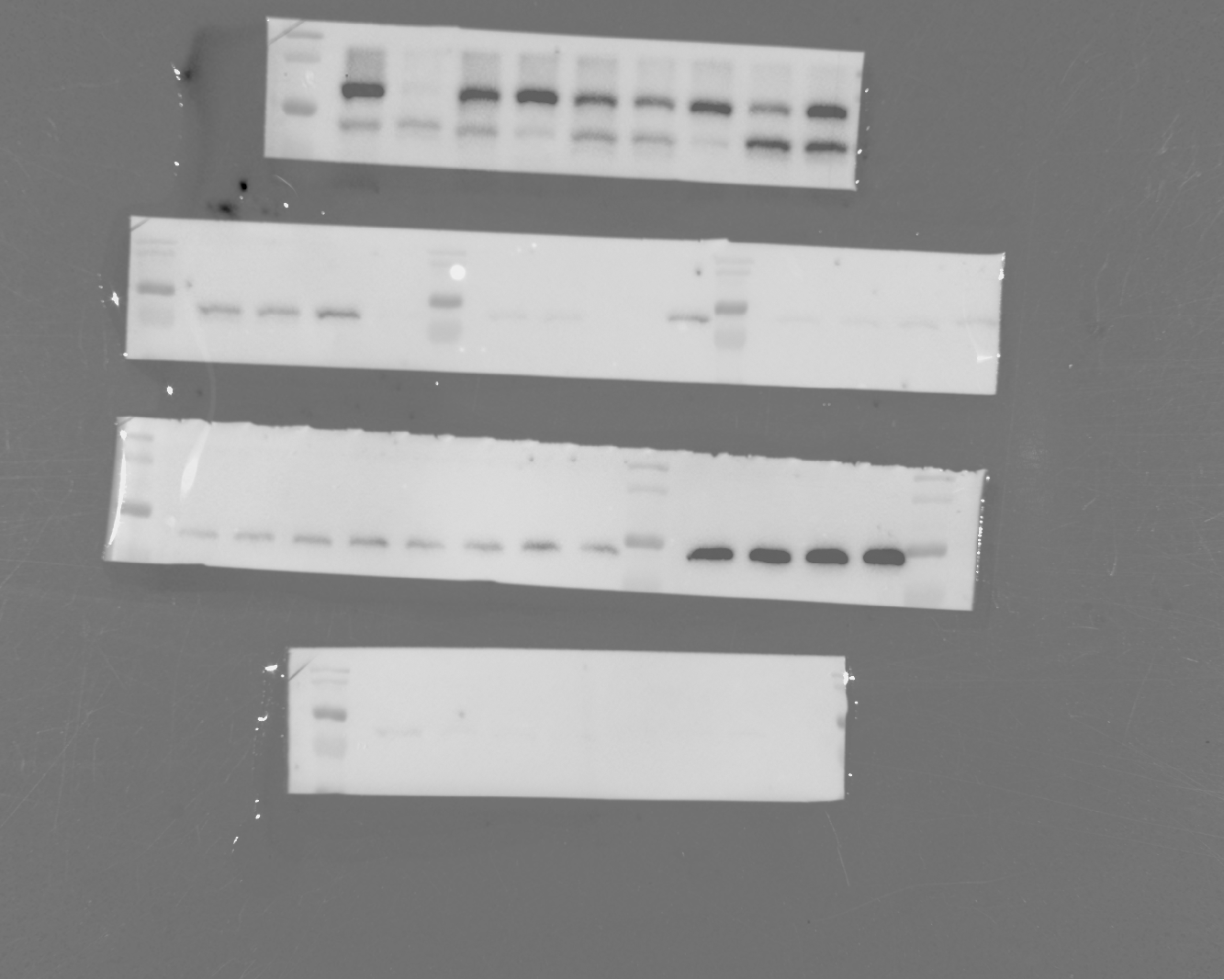

Supplement: Supplementary file 8 [file DataSheet6.ZIP › rat-ICC-L+B/par-pst-st-pst_9(Composite).tif]

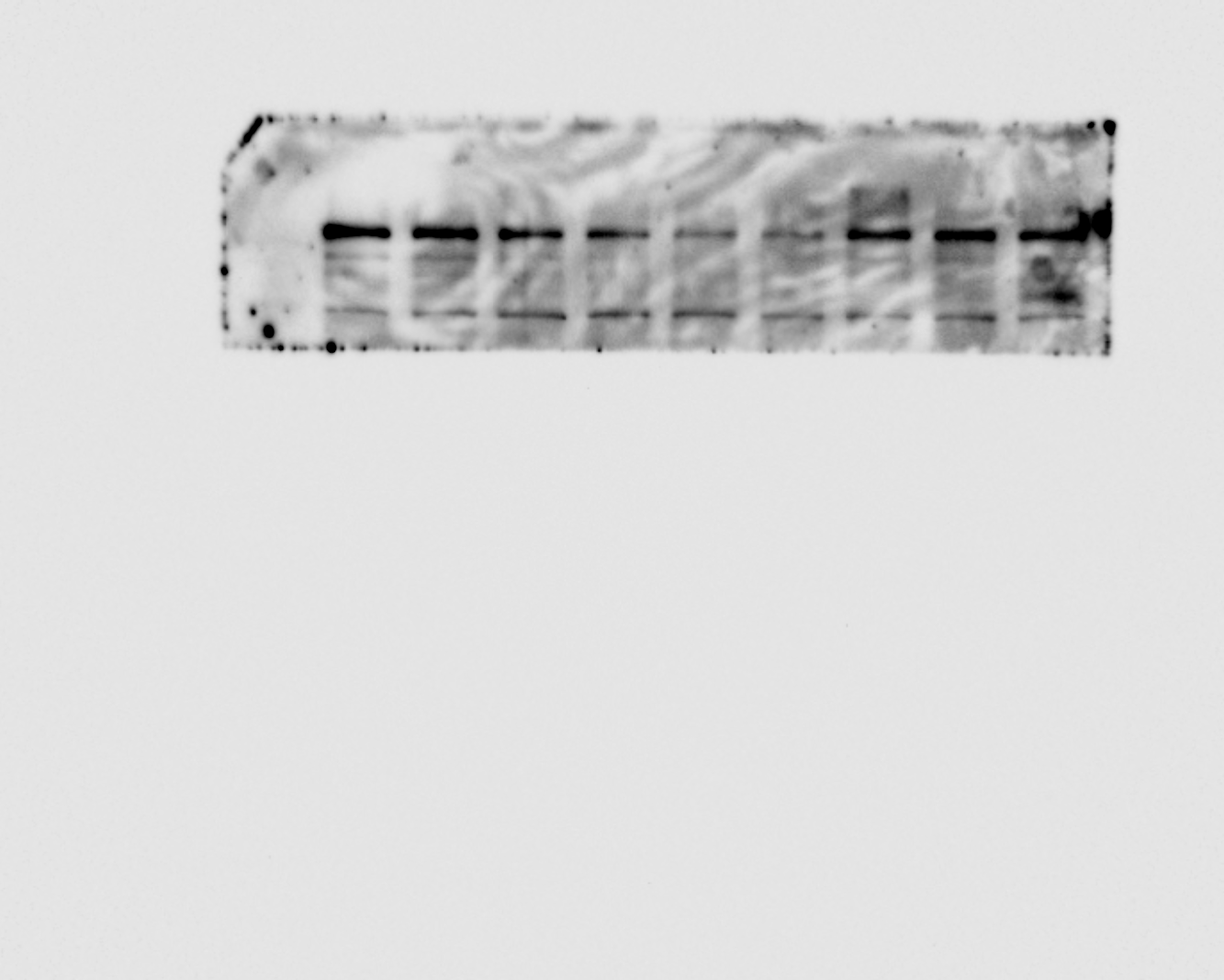

Supplement: Supplementary file 9 [file DataSheet2.ZIP › animal model/117rat_1(Chemiluminescence).tif]

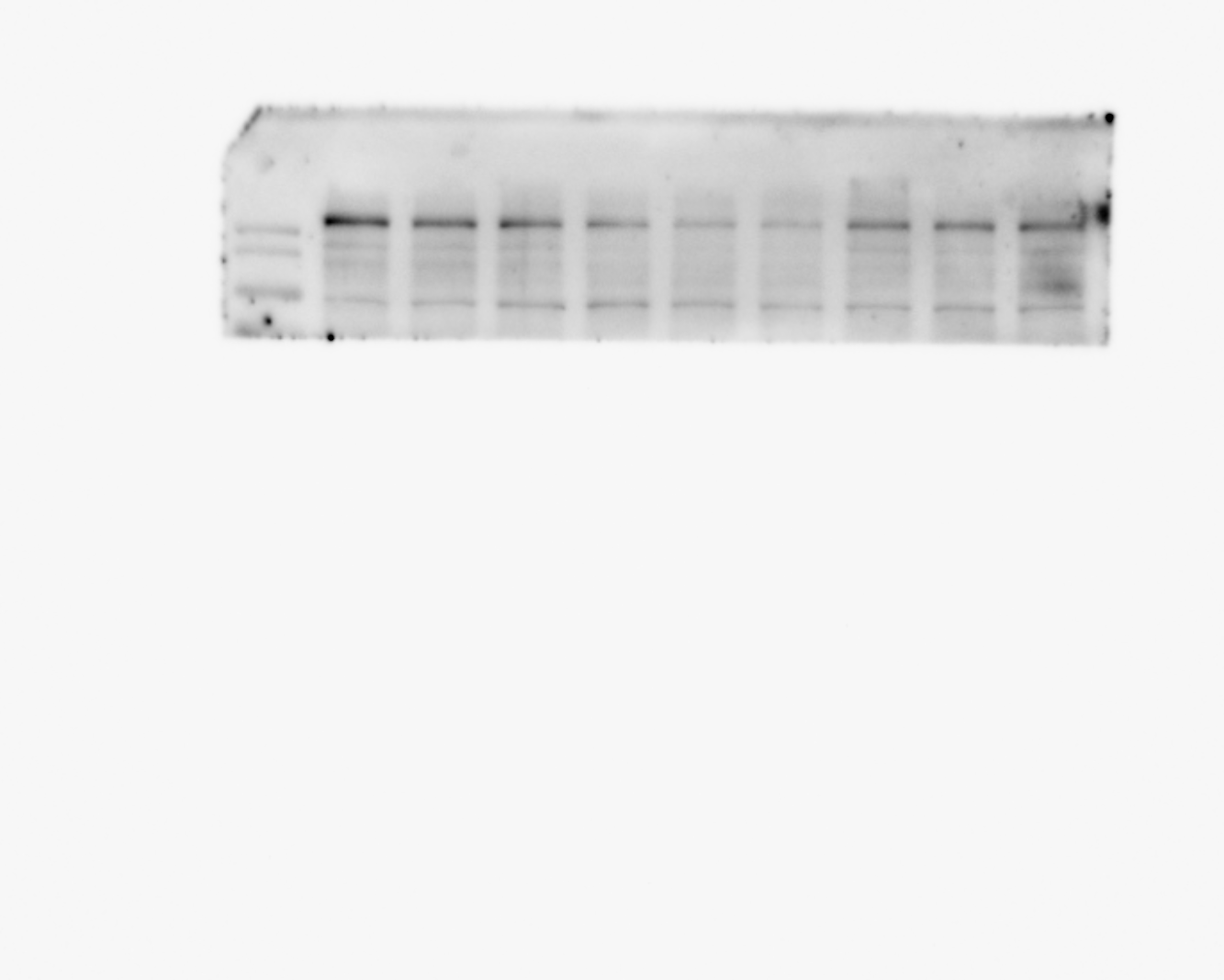

Supplement: Supplementary file 9 [file DataSheet2.ZIP › animal model/117rat_2(Chemiluminescence).tif]

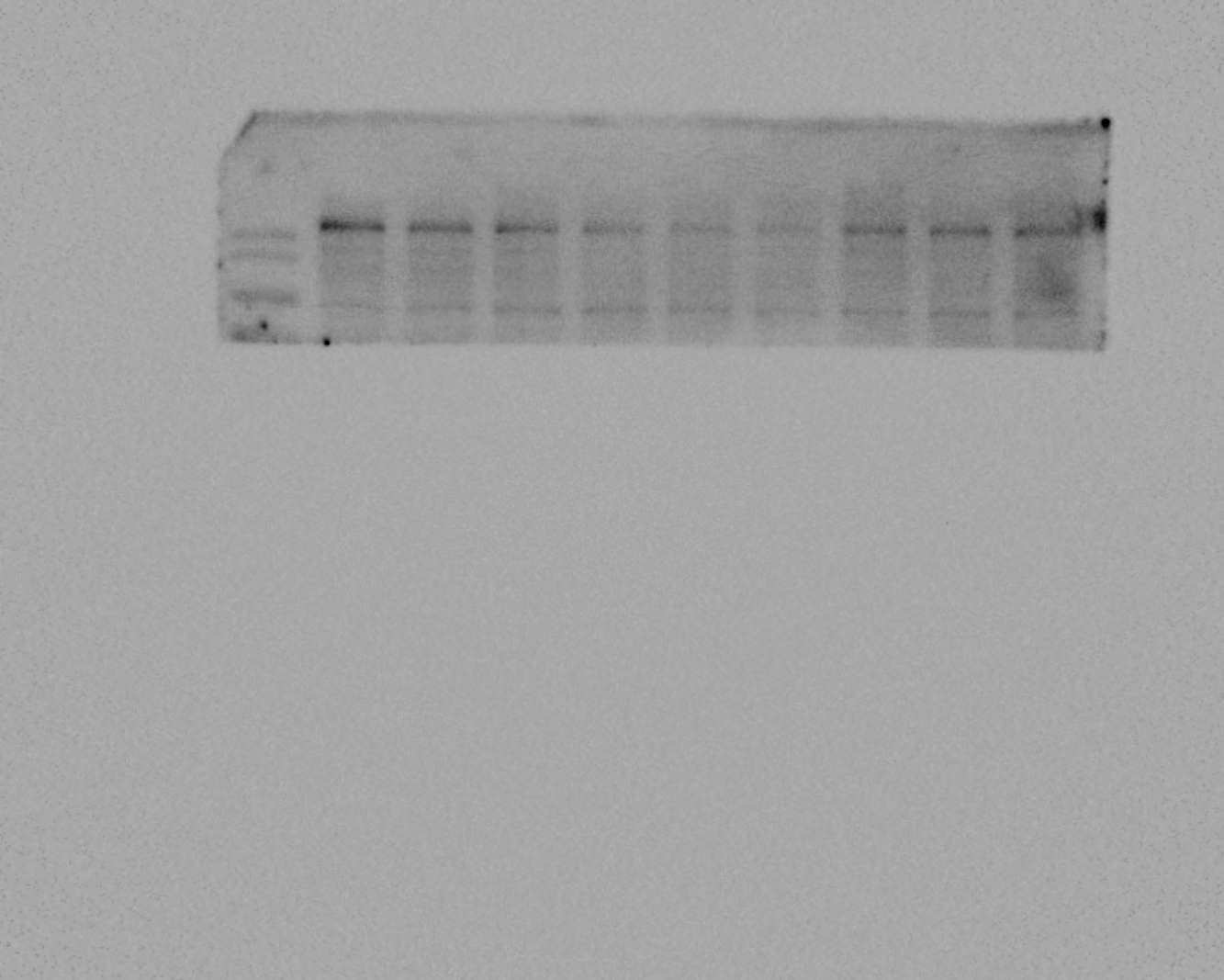

Supplement: Supplementary file 9 [file DataSheet2.ZIP › animal model/117rat_3(Chemiluminescence).tif]

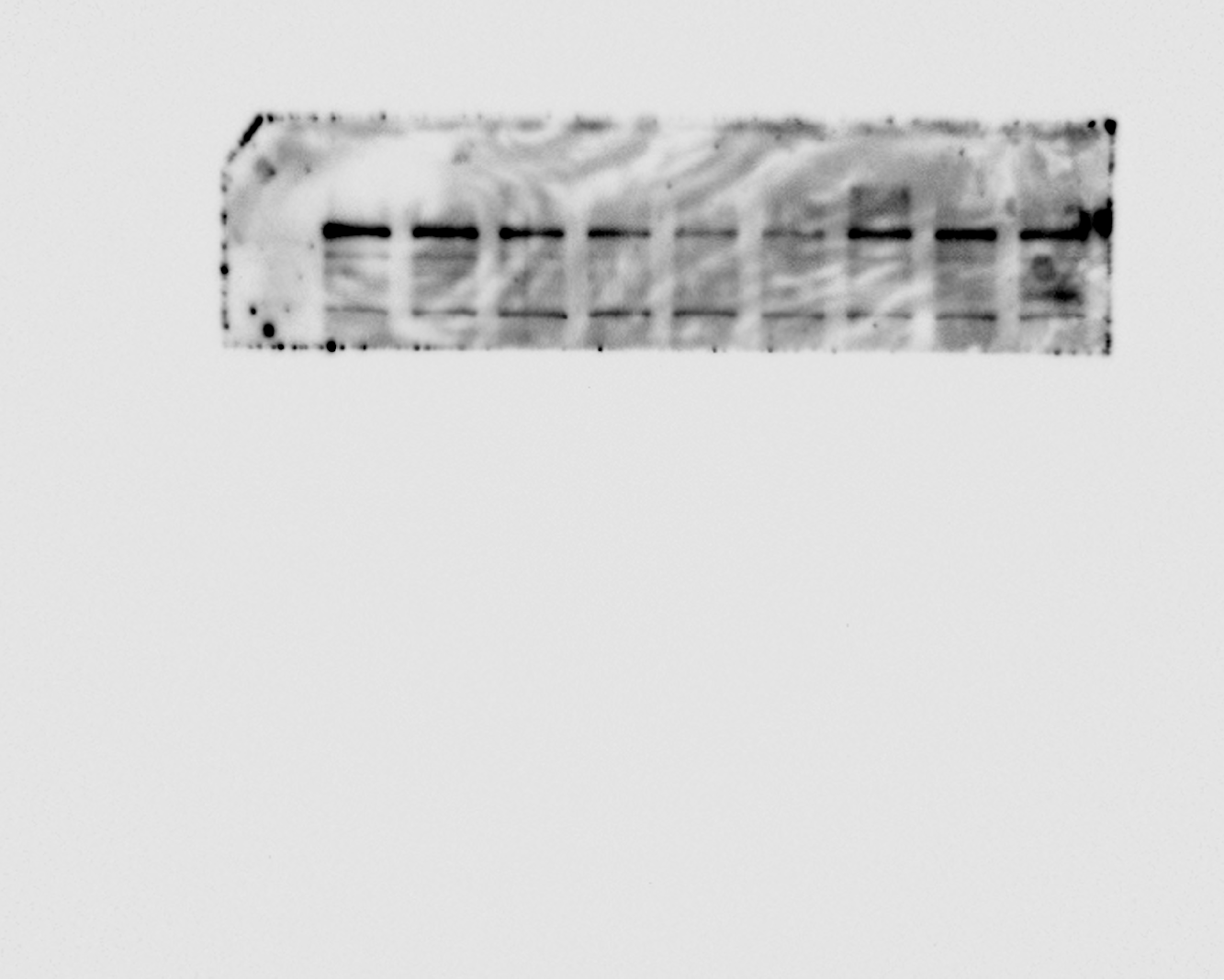

Supplement: Supplementary file 9 [file DataSheet2.ZIP › animal model/117rat_5(Chemiluminescence).tif]

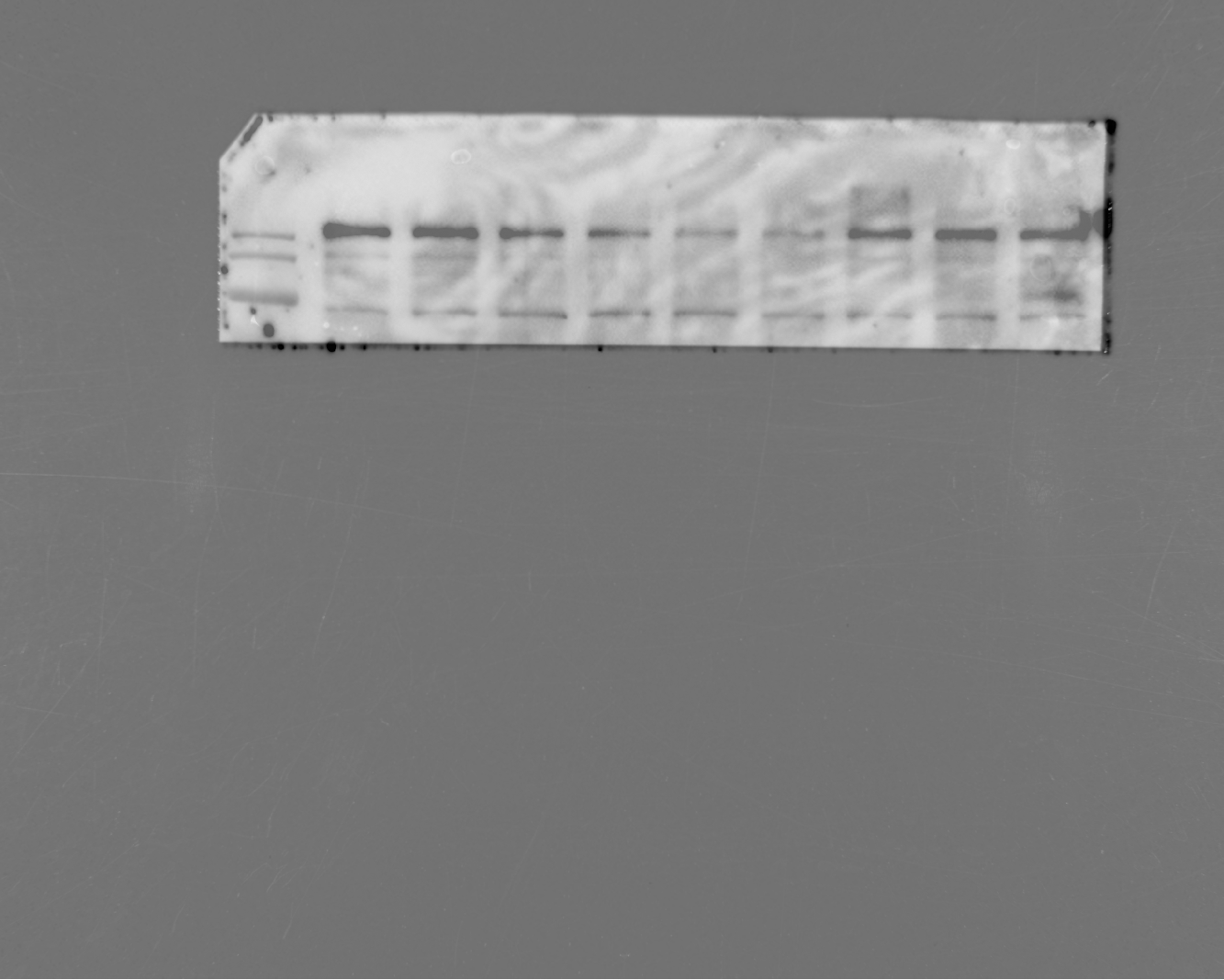

Supplement: Supplementary file 9 [file DataSheet2.ZIP › animal model/117rat_5(Composite).tif]
