# Supplementary figures and images for: Effects of Xiao Chengqi Formula on Slow Transit Constipation by Assessing Gut Microbiota and Metabolomics Analysis in vitro and in vivo (part 3 of 3)
Source: Front Pharmacol. 2022 Jun 8;13:864598. doi: 10.3389/fphar.2022.864598 (PMC9237644; doi:10.3389/fphar.2022.864598)

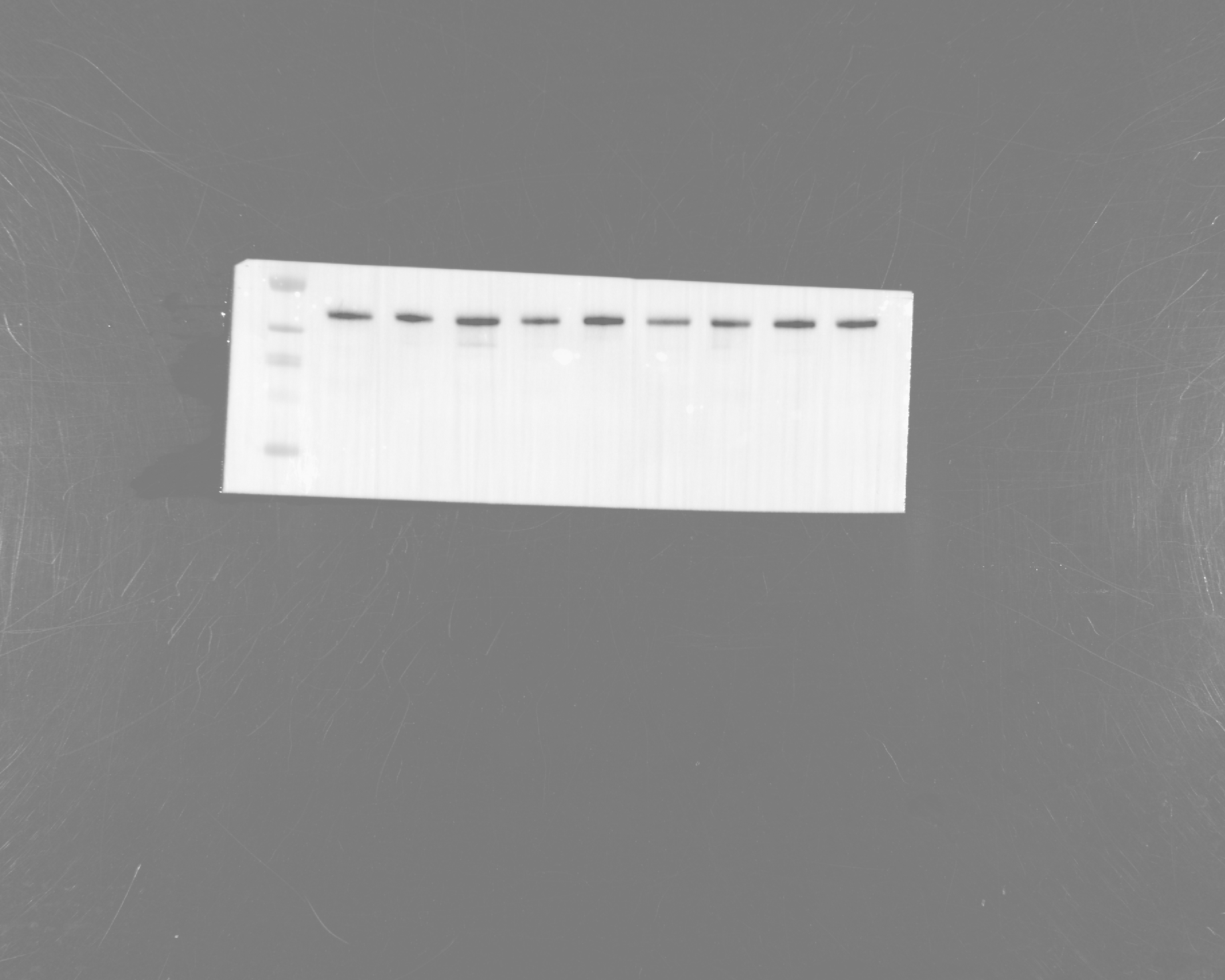

Supplement: Supplementary file 9 [file DataSheet2.ZIP › animal model/ACTIN (1).tif]

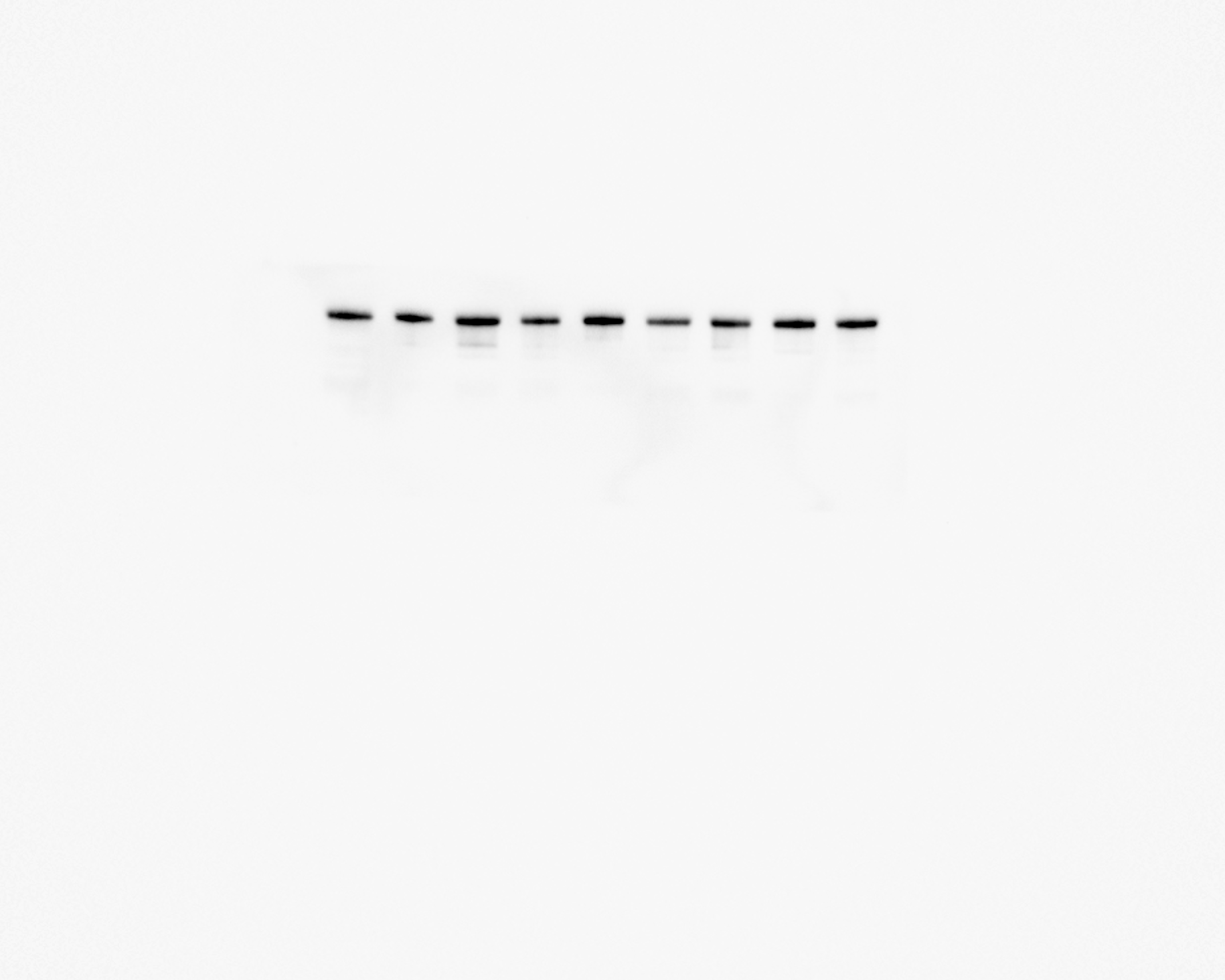

Supplement: Supplementary file 9 [file DataSheet2.ZIP › animal model/ACTIN (2).tif]

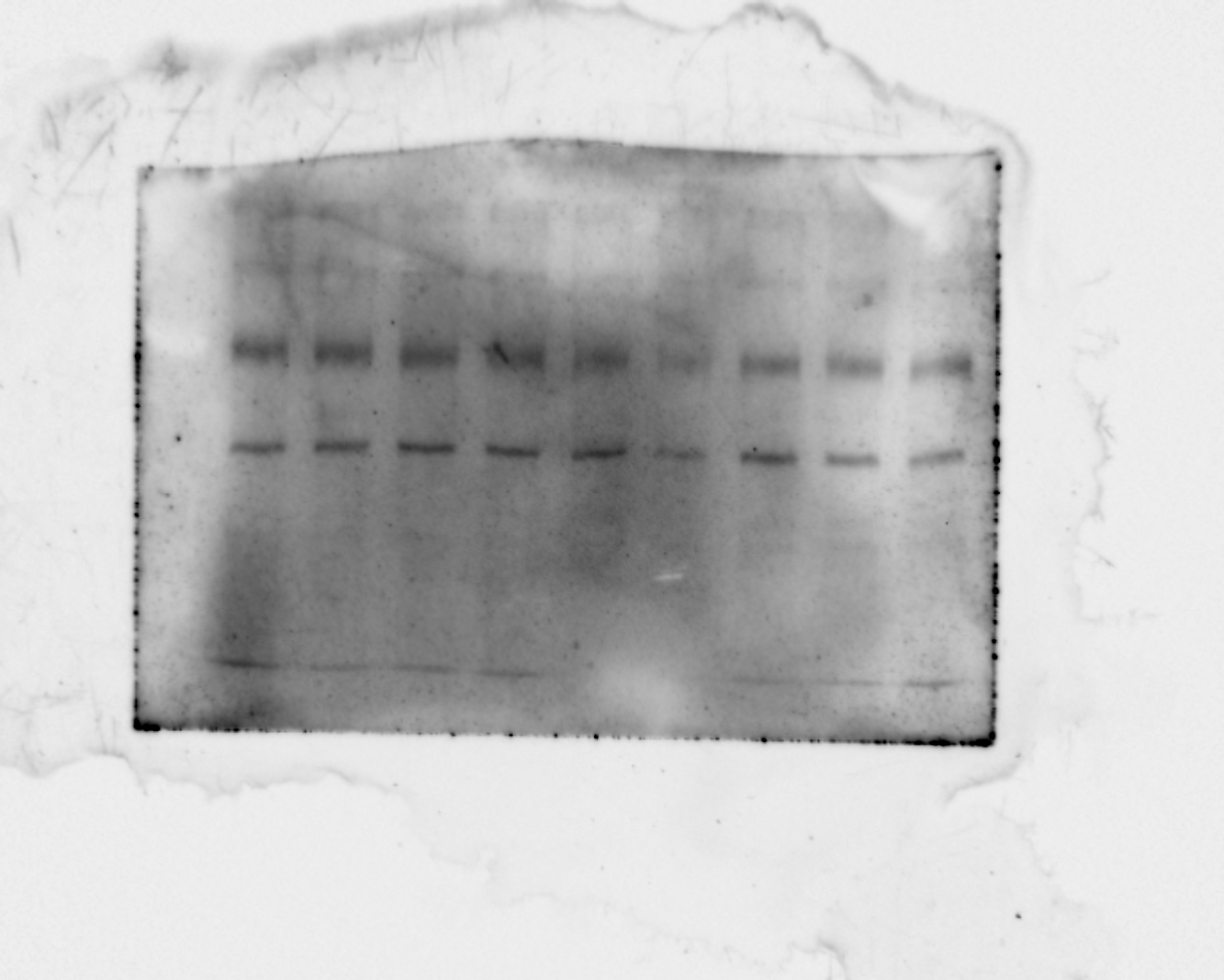

Supplement: Supplementary file 9 [file DataSheet2.ZIP › animal model/actin_3(Chemiluminescence).tif]

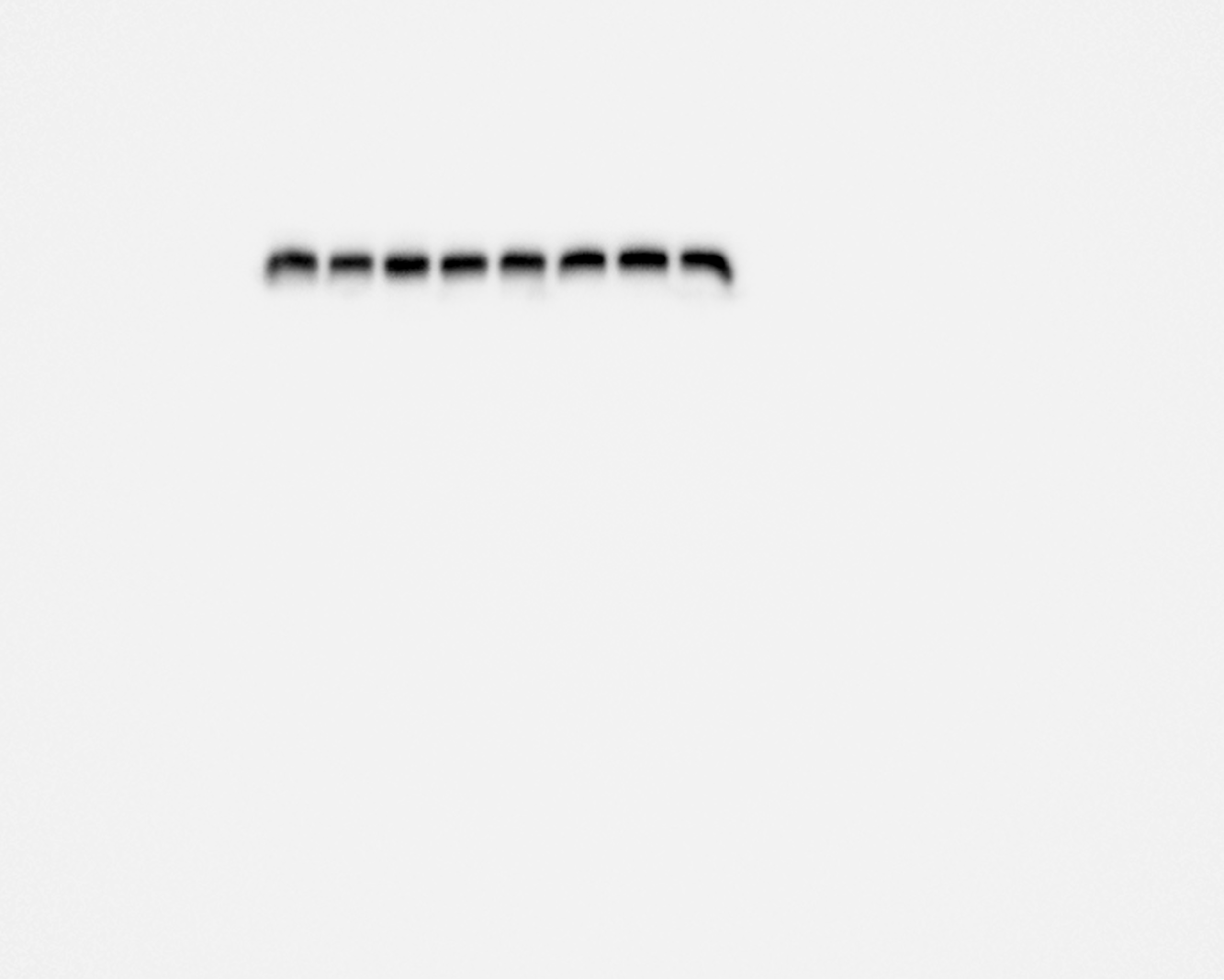

Supplement: Supplementary file 9 [file DataSheet2.ZIP › animal model/actinrat2_3(Chemiluminescence).tif]

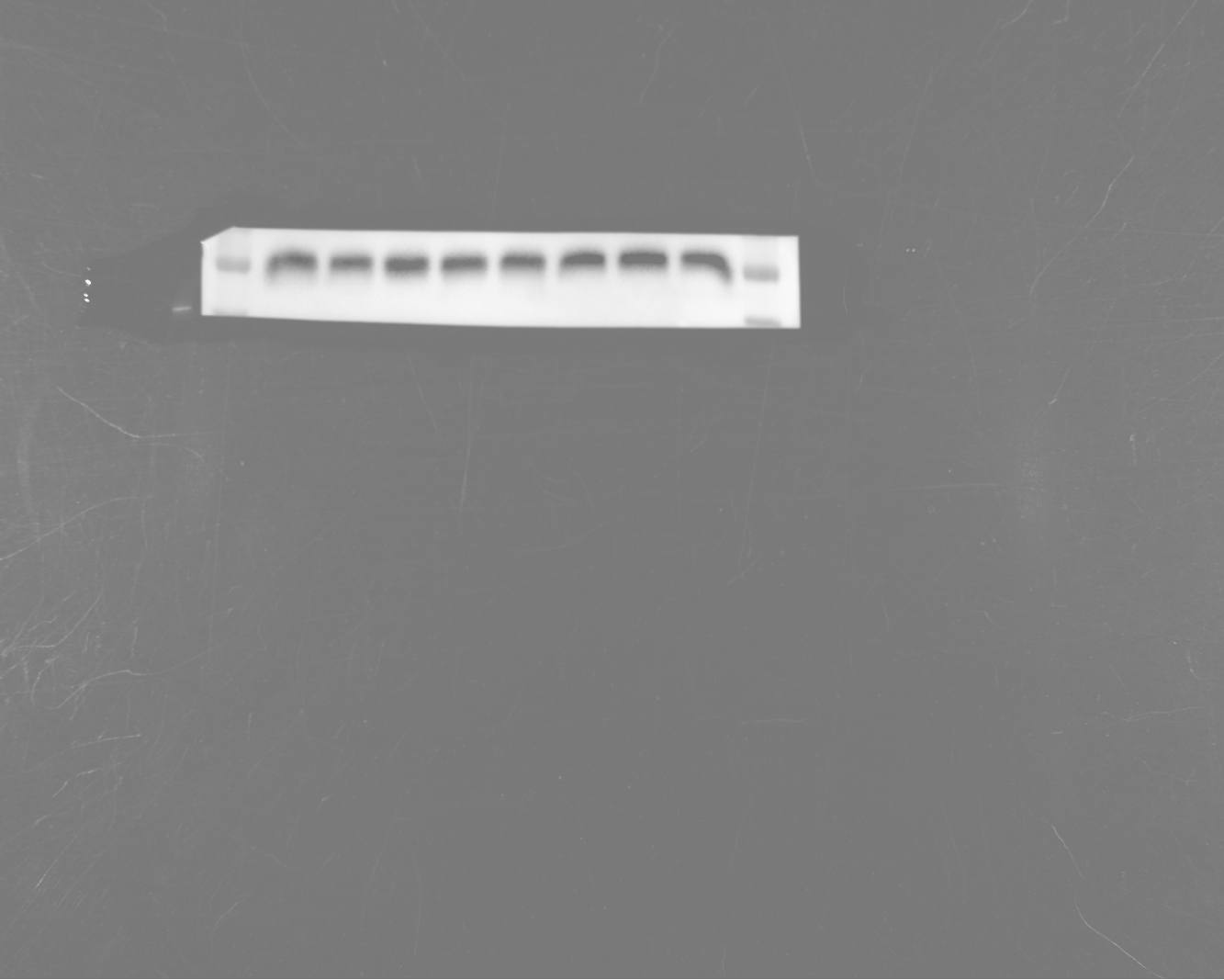

Supplement: Supplementary file 9 [file DataSheet2.ZIP › animal model/actinrat2_3(Composite).tif]

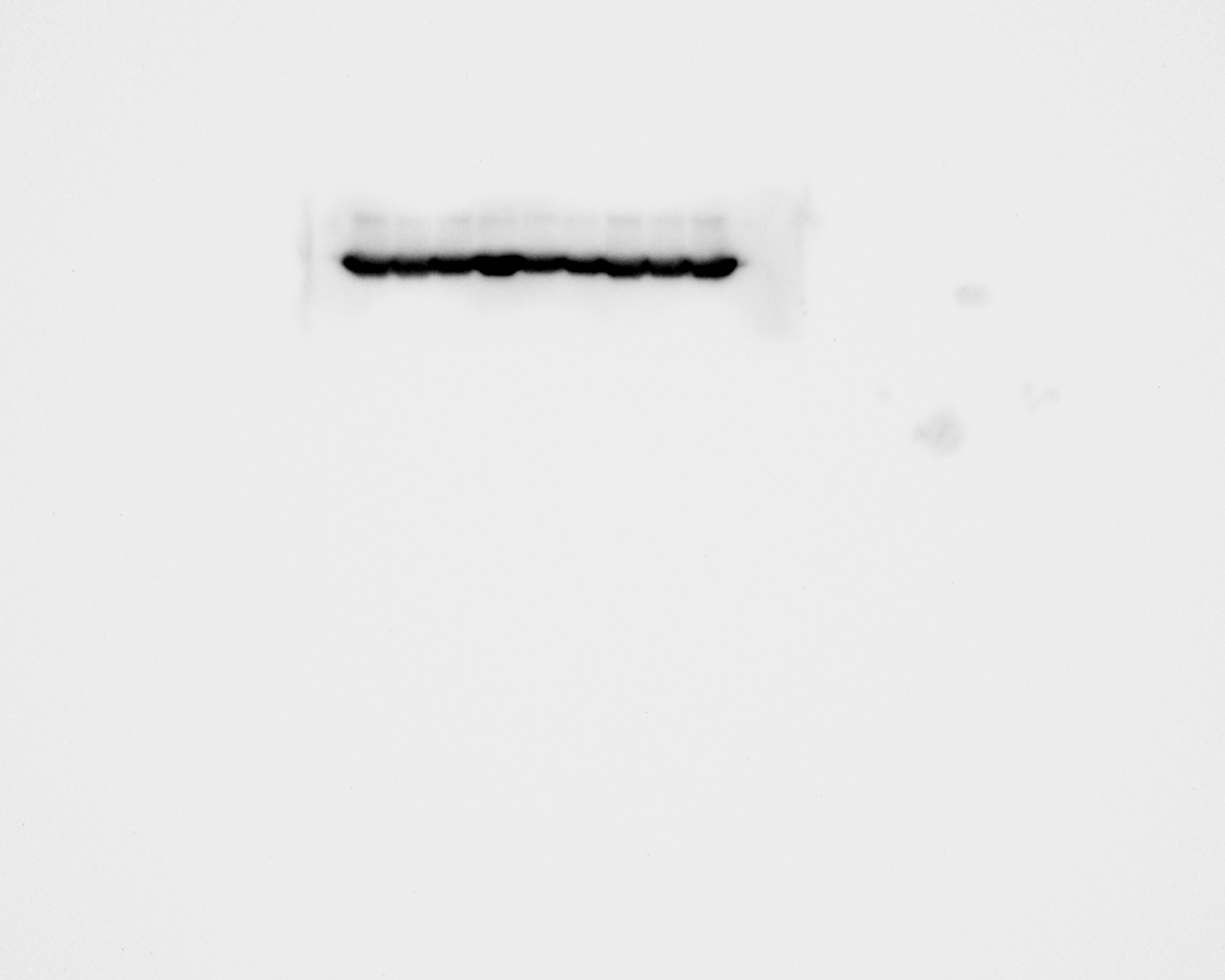

Supplement: Supplementary file 9 [file DataSheet2.ZIP › animal model/actin-ratcolon-117_3(Chemiluminescence).tif]

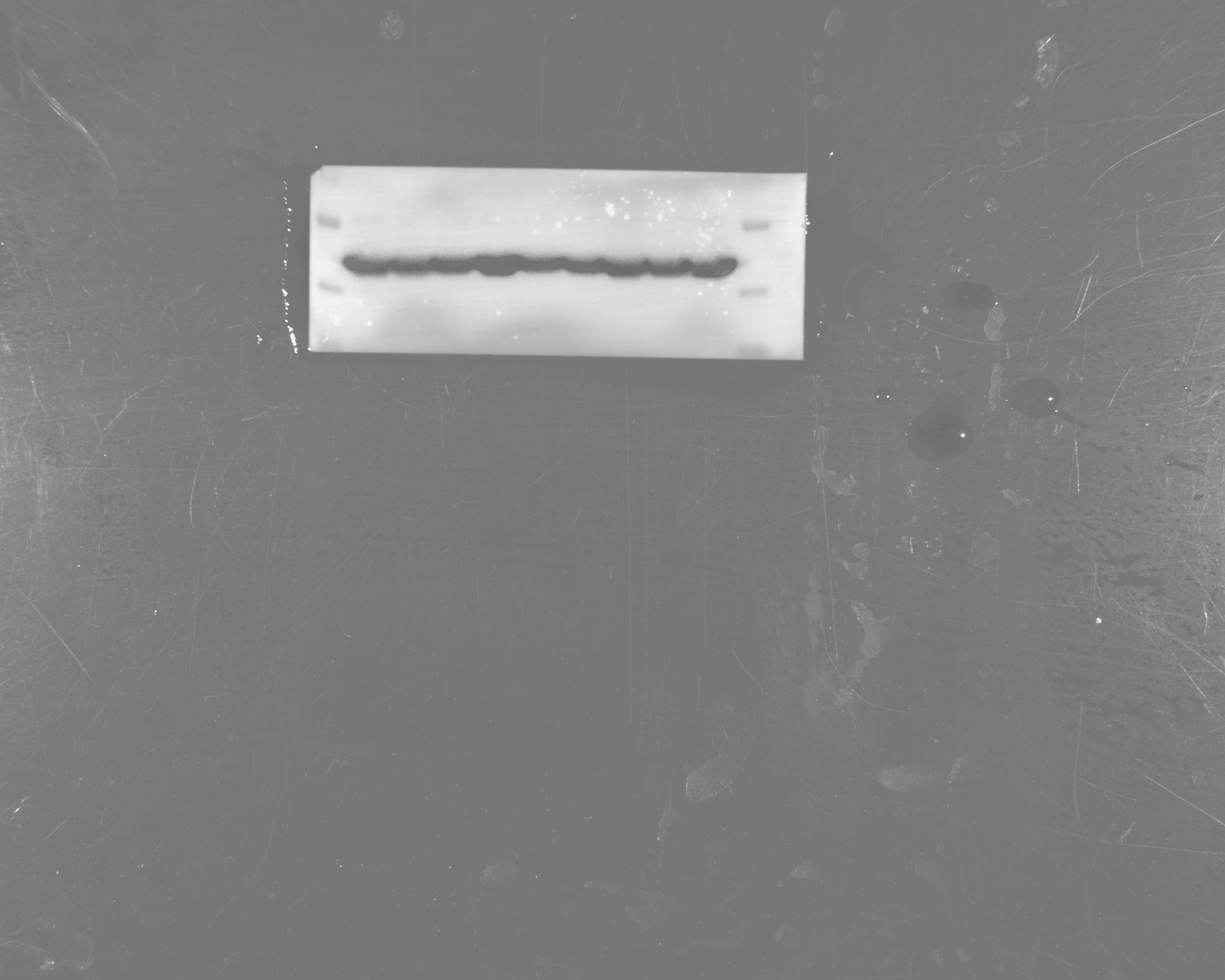

Supplement: Supplementary file 9 [file DataSheet2.ZIP › animal model/actin-ratcolon-117_3(Composite).tif]

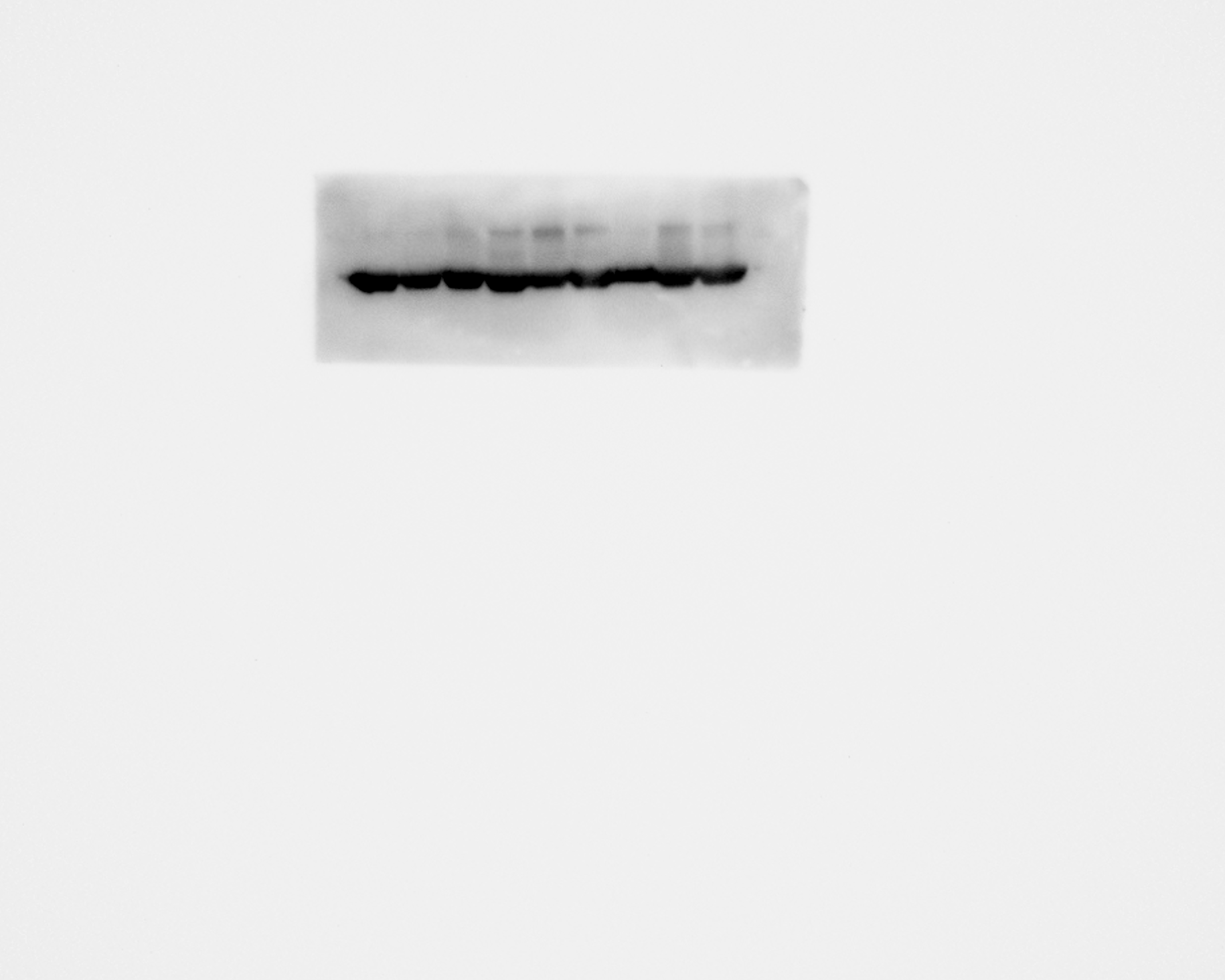

Supplement: Supplementary file 9 [file DataSheet2.ZIP › animal model/actin-ratcolon-cd360_5(Chemiluminescence).tif]

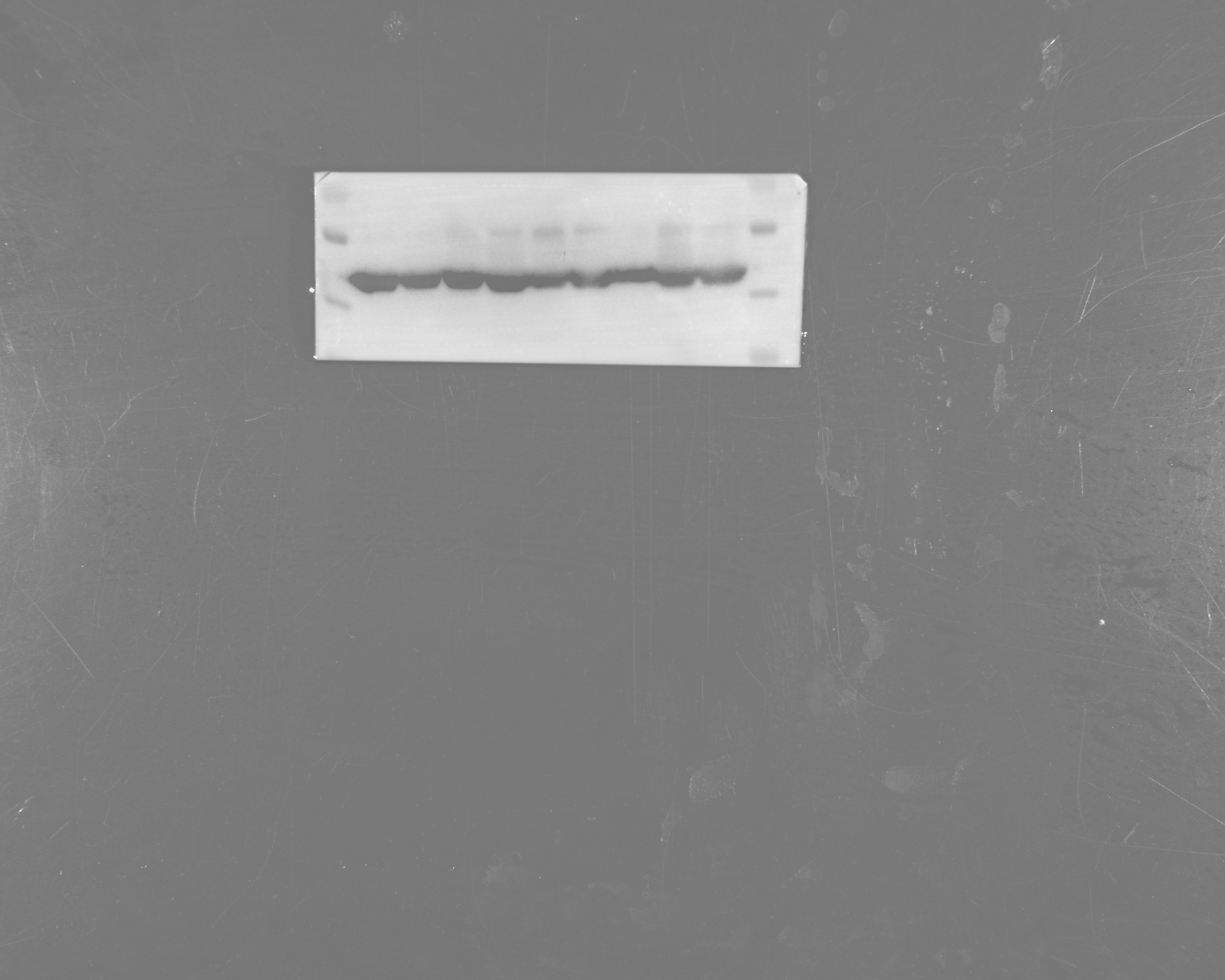

Supplement: Supplementary file 9 [file DataSheet2.ZIP › animal model/actin-ratcolon-cd360_5(Composite).tif]

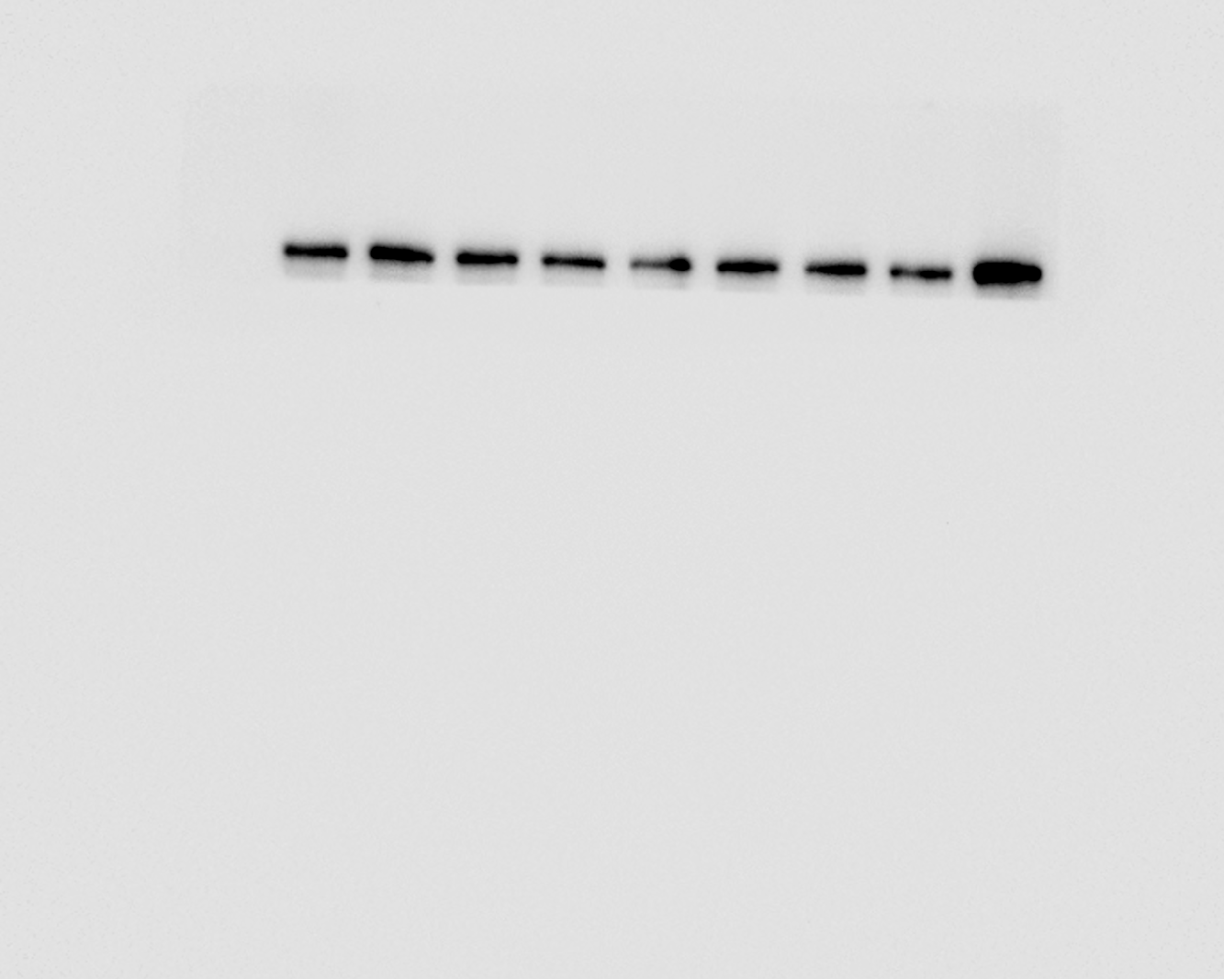

Supplement: Supplementary file 9 [file DataSheet2.ZIP › animal model/anorectal 2020-11-18 17h19m47s(Chemiluminescence).tif]

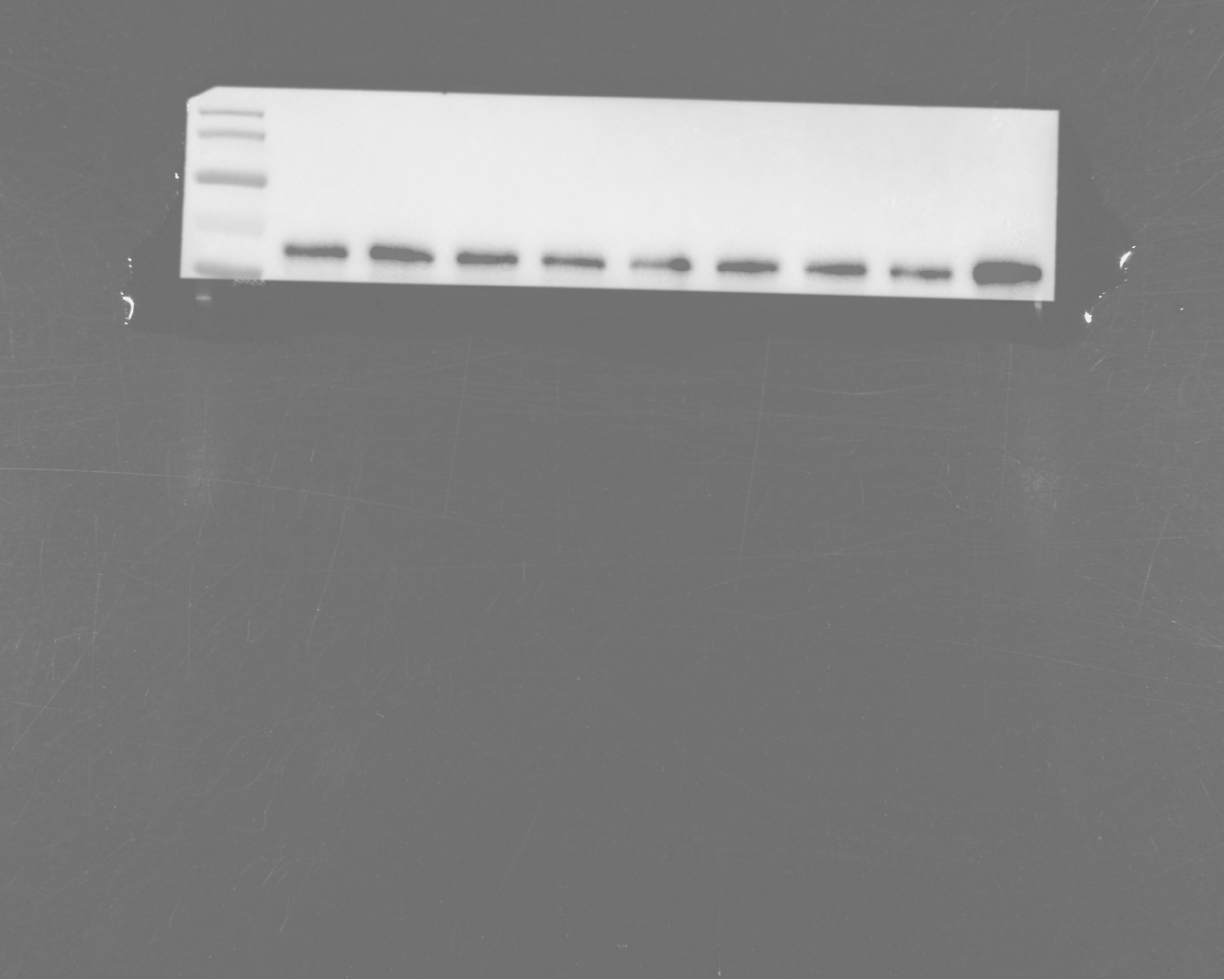

Supplement: Supplementary file 9 [file DataSheet2.ZIP › animal model/anorectal 2020-11-18 17h19m47s(Composite).tif]

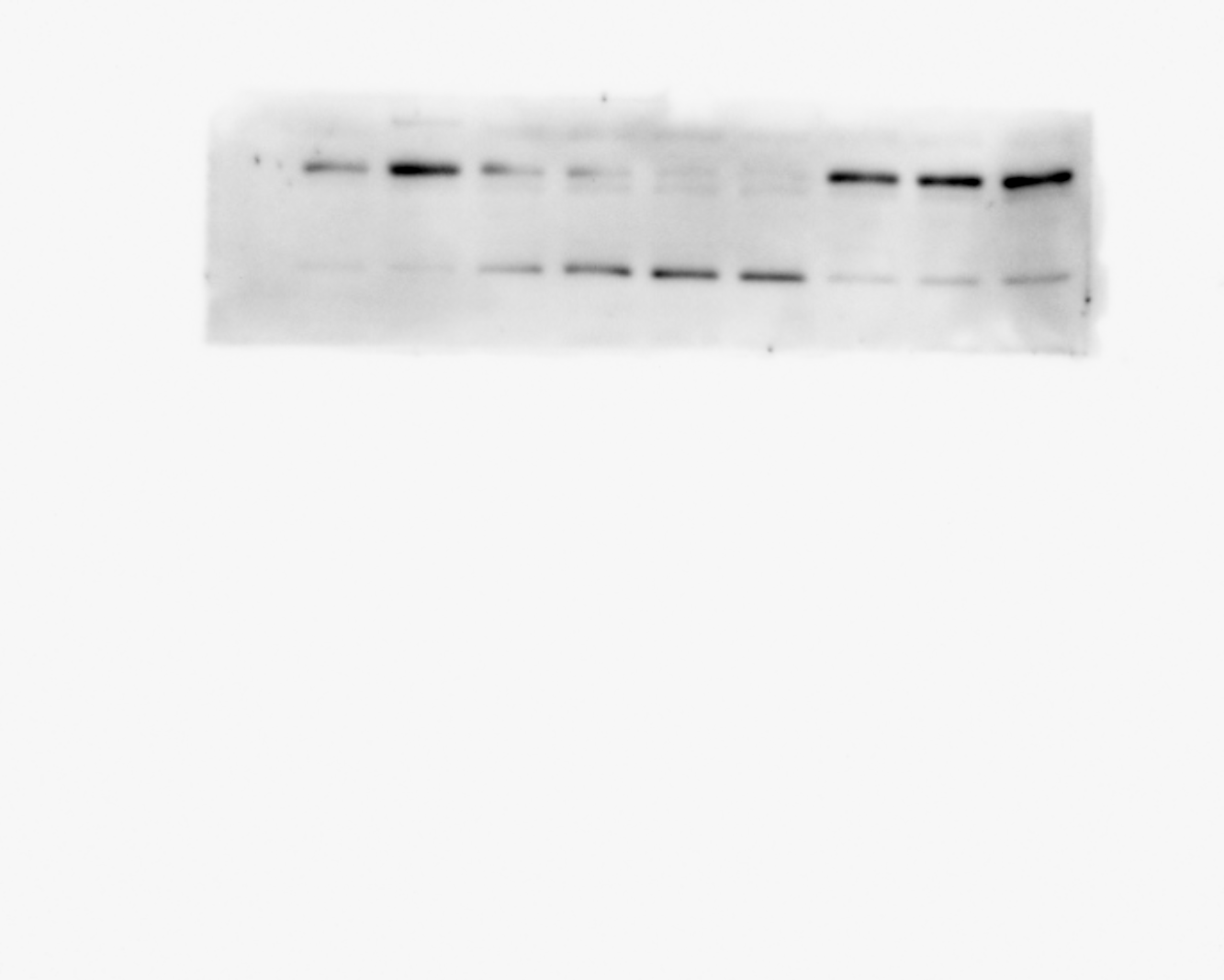

Supplement: Supplementary file 9 [file DataSheet2.ZIP › animal model/IL-21R (2).tif]

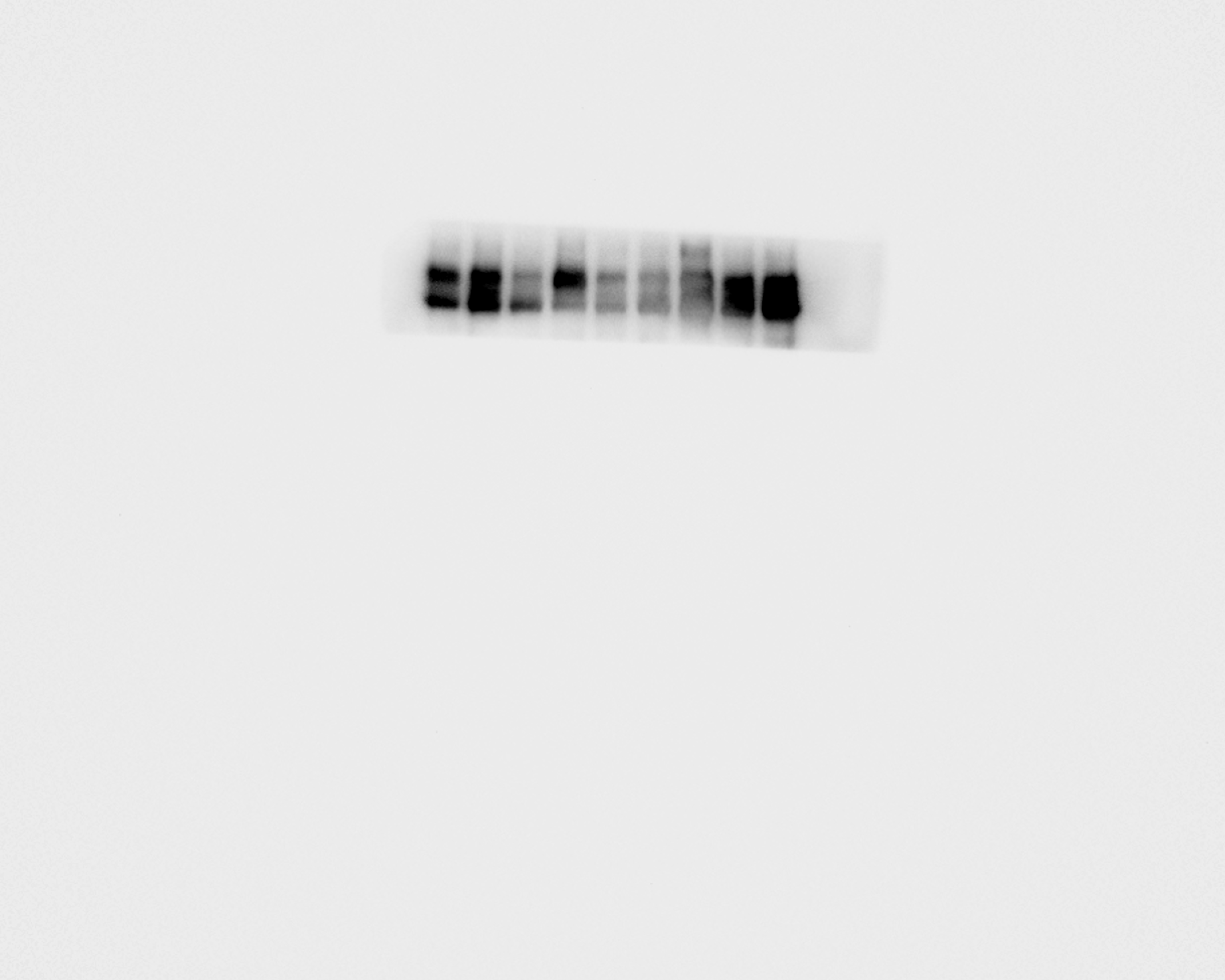

Supplement: Supplementary file 9 [file DataSheet2.ZIP › animal model/ratcolon117-3_3(Chemiluminescence).tif]

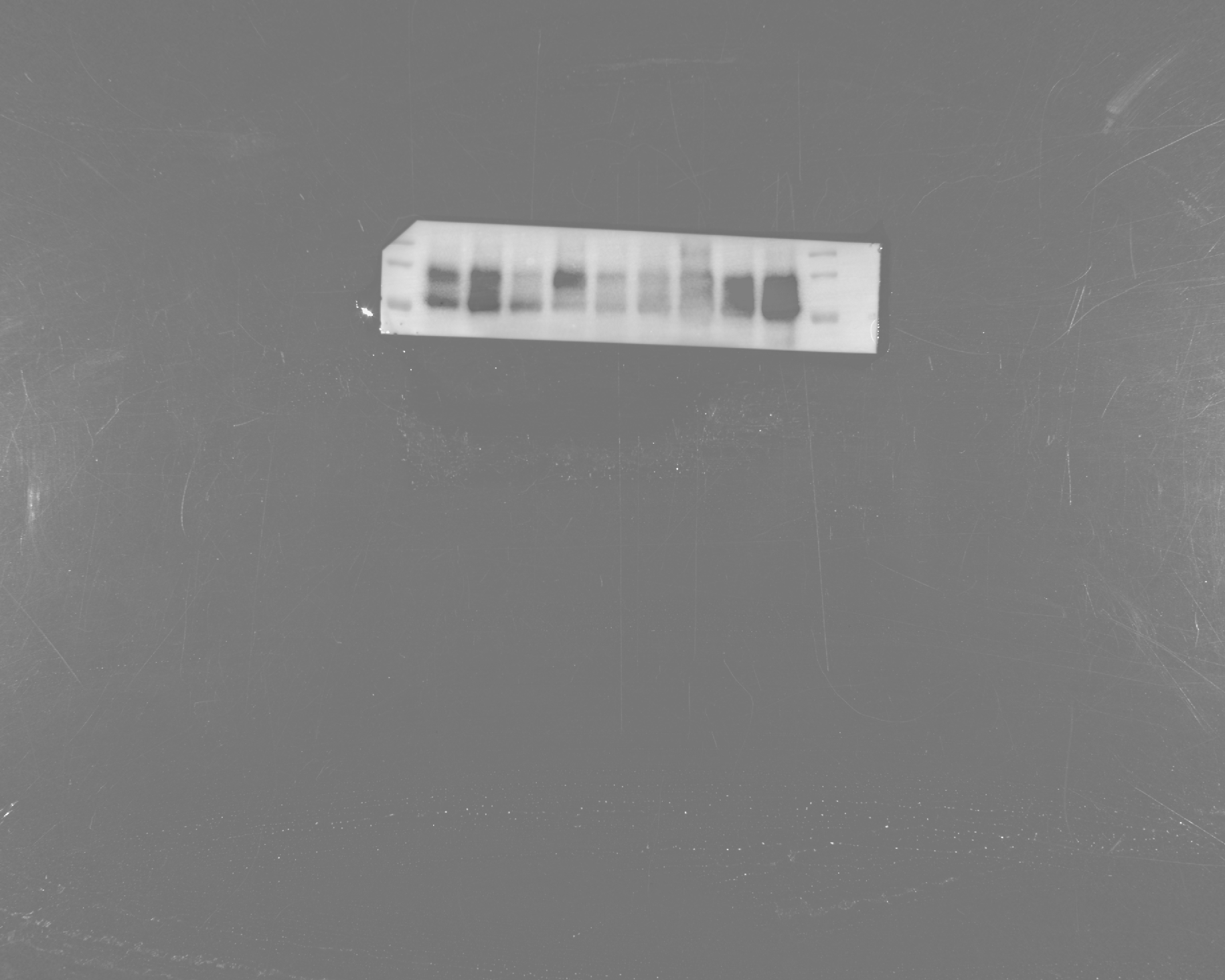

Supplement: Supplementary file 9 [file DataSheet2.ZIP › animal model/ratcolon117-3_3(Composite).tif]

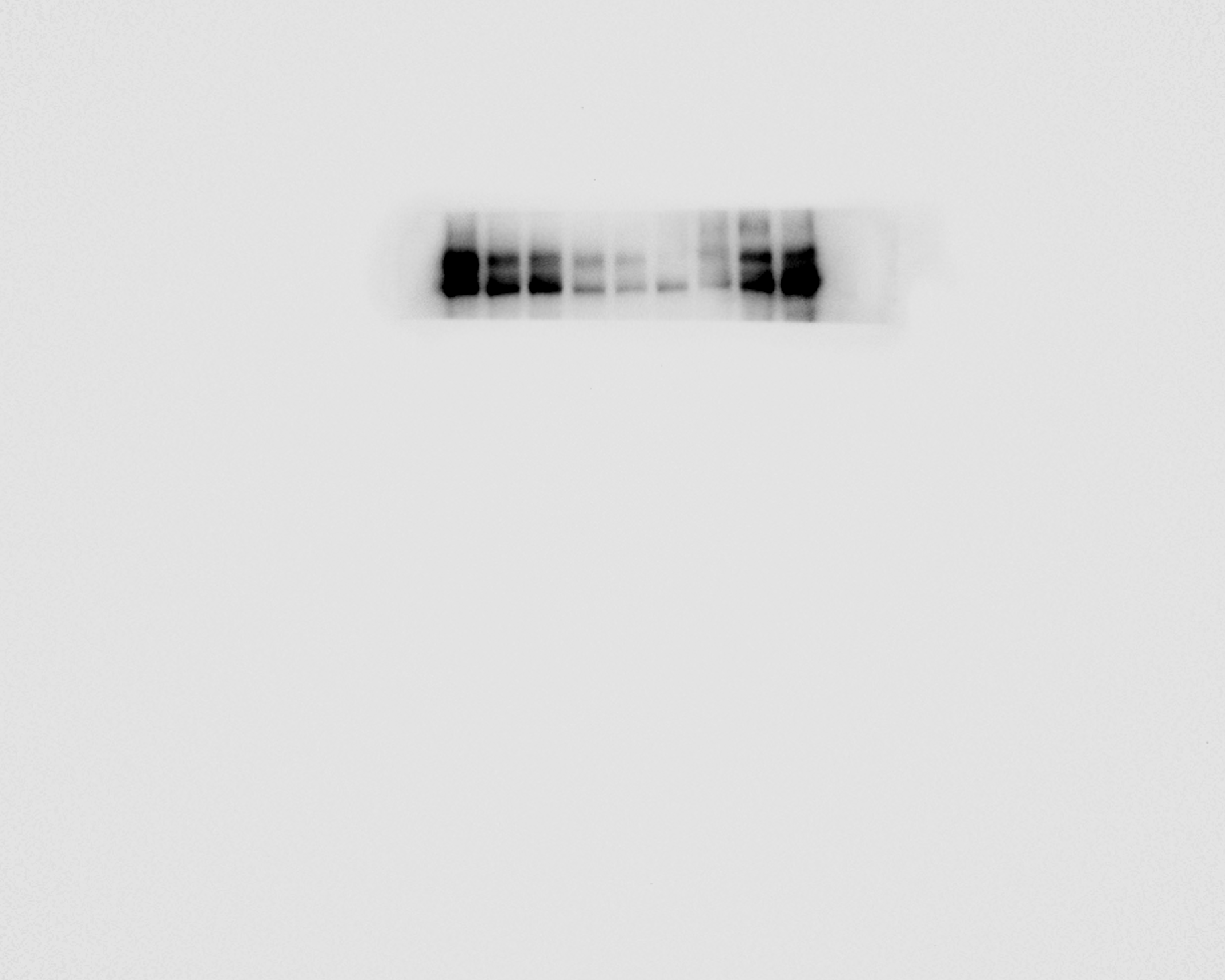

Supplement: Supplementary file 9 [file DataSheet2.ZIP › animal model/ratcolon117-4_3(Chemiluminescence).tif]

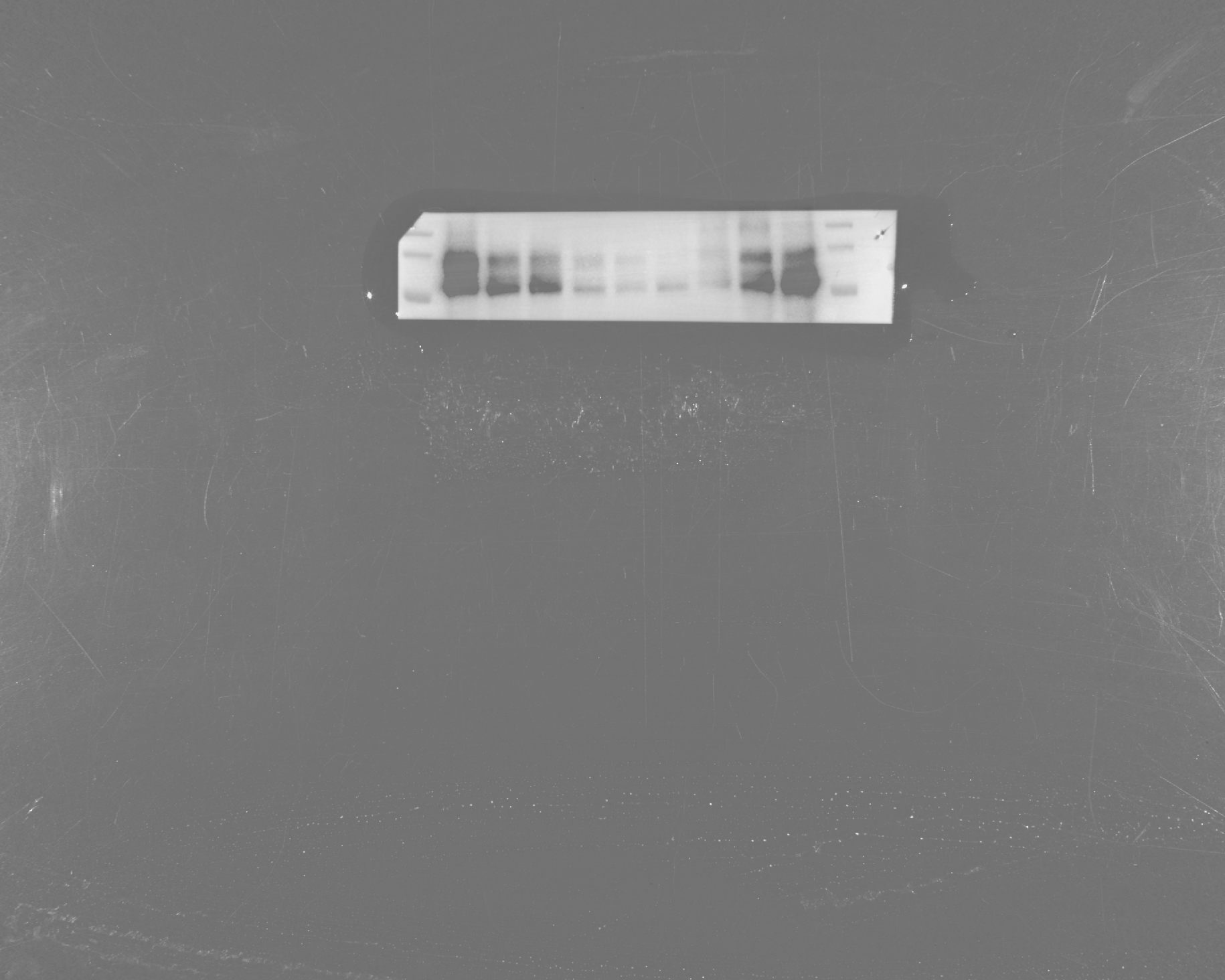

Supplement: Supplementary file 9 [file DataSheet2.ZIP › animal model/ratcolon117-4_3(Composite).tif]

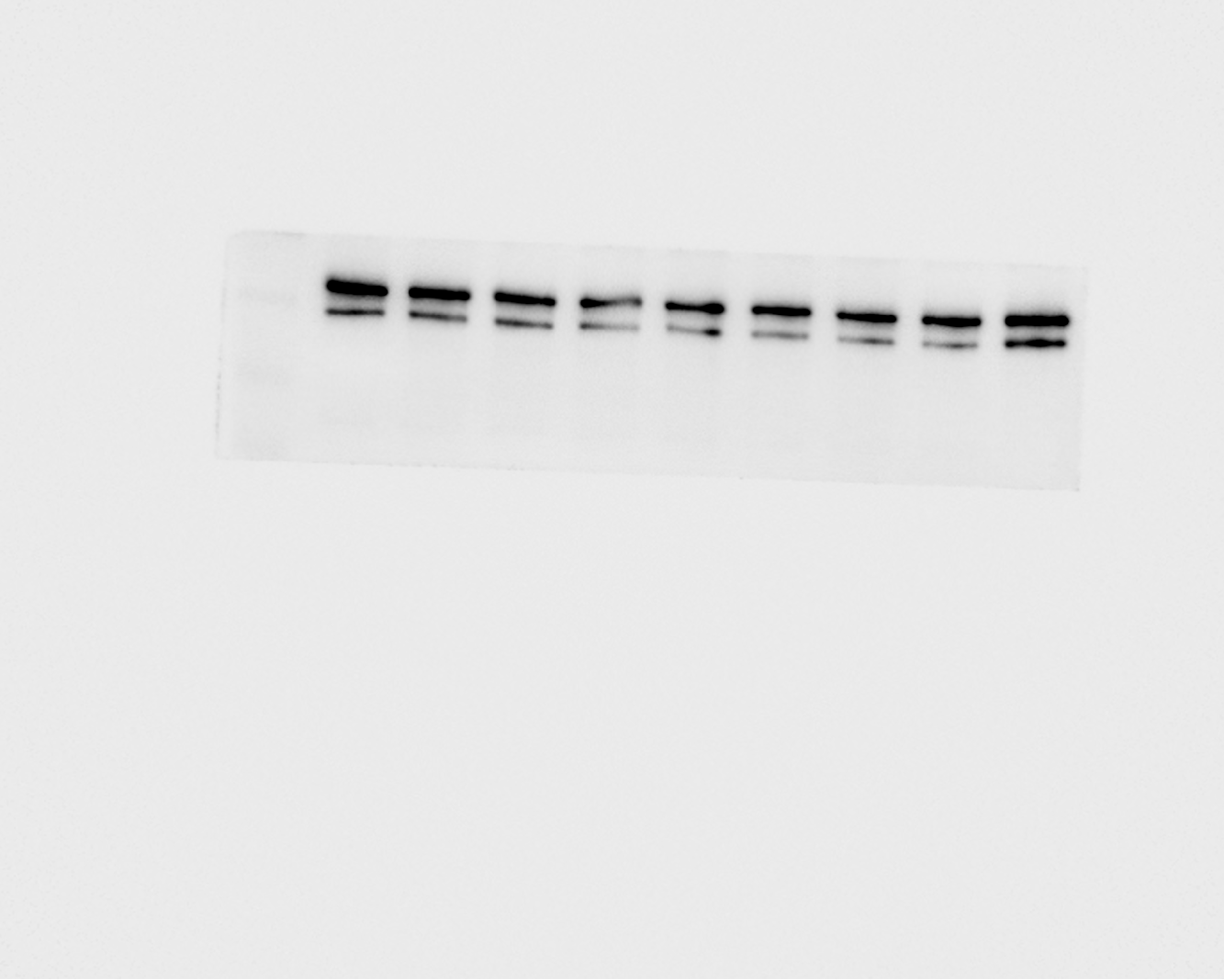

Supplement: Supplementary file 9 [file DataSheet2.ZIP › animal model/raterk_4(Chemiluminescence).tif]

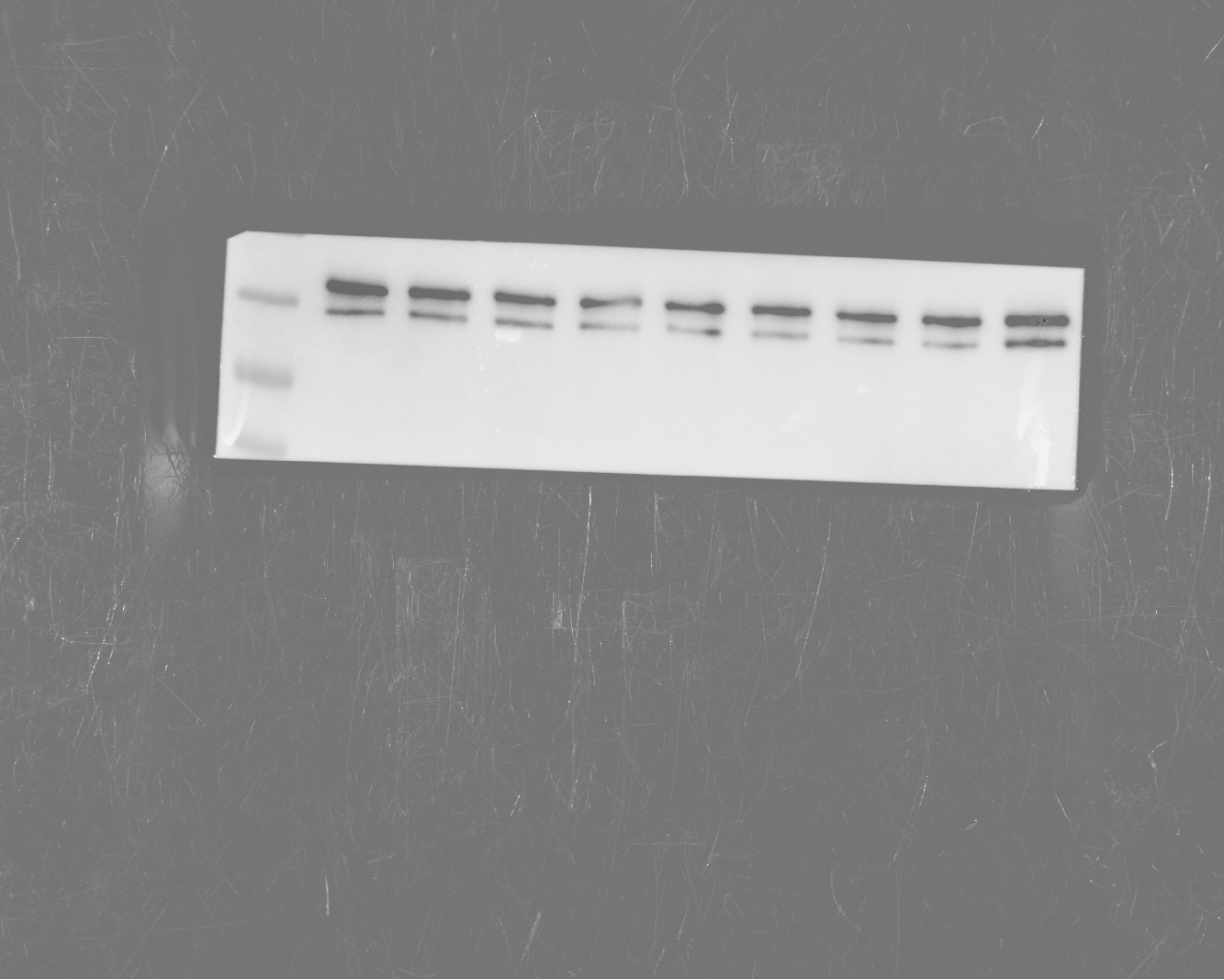

Supplement: Supplementary file 9 [file DataSheet2.ZIP › animal model/raterk_4(Composite).tif]

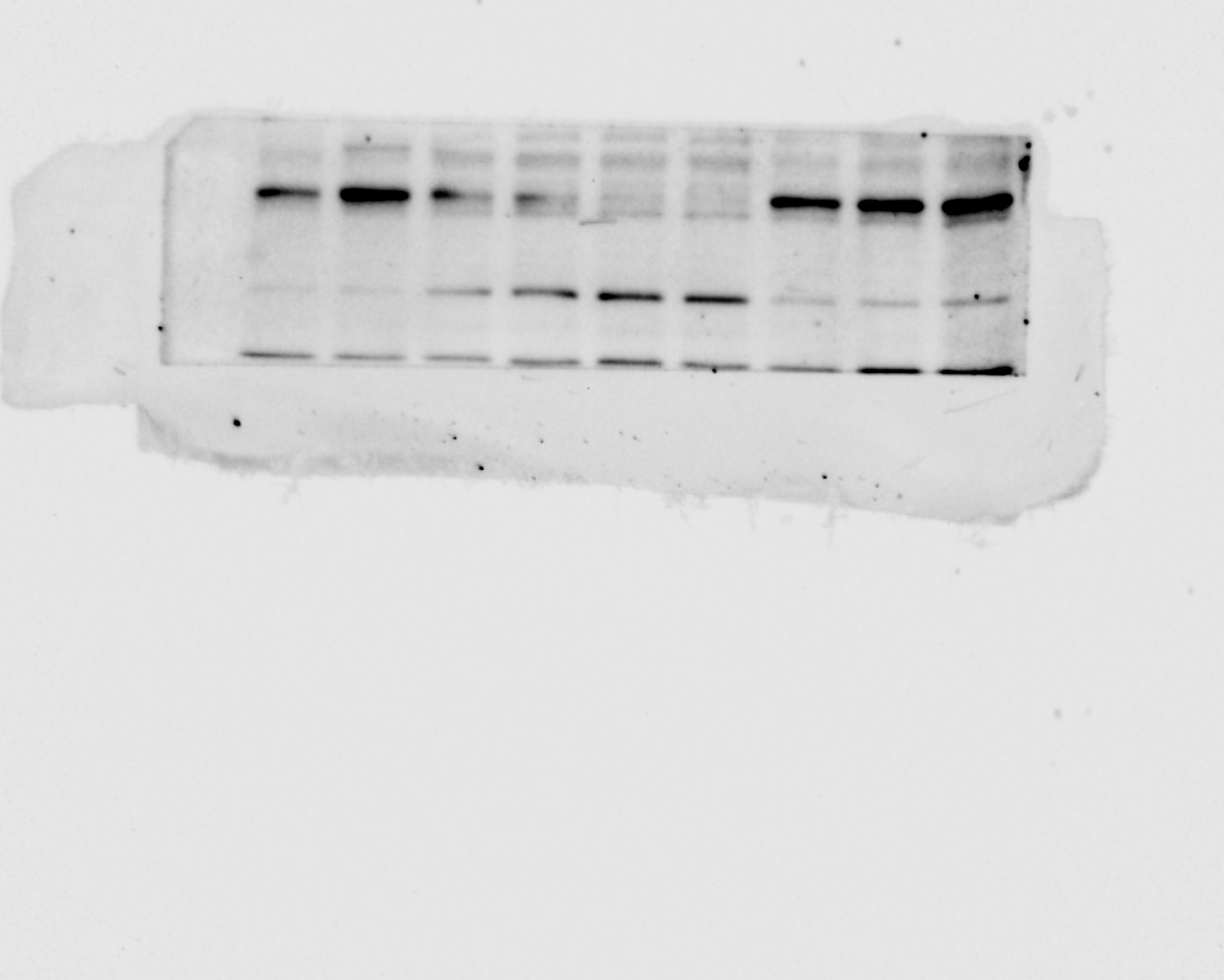

Supplement: Supplementary file 9 [file DataSheet2.ZIP › animal model/ratp21(Chemiluminescence).tif]

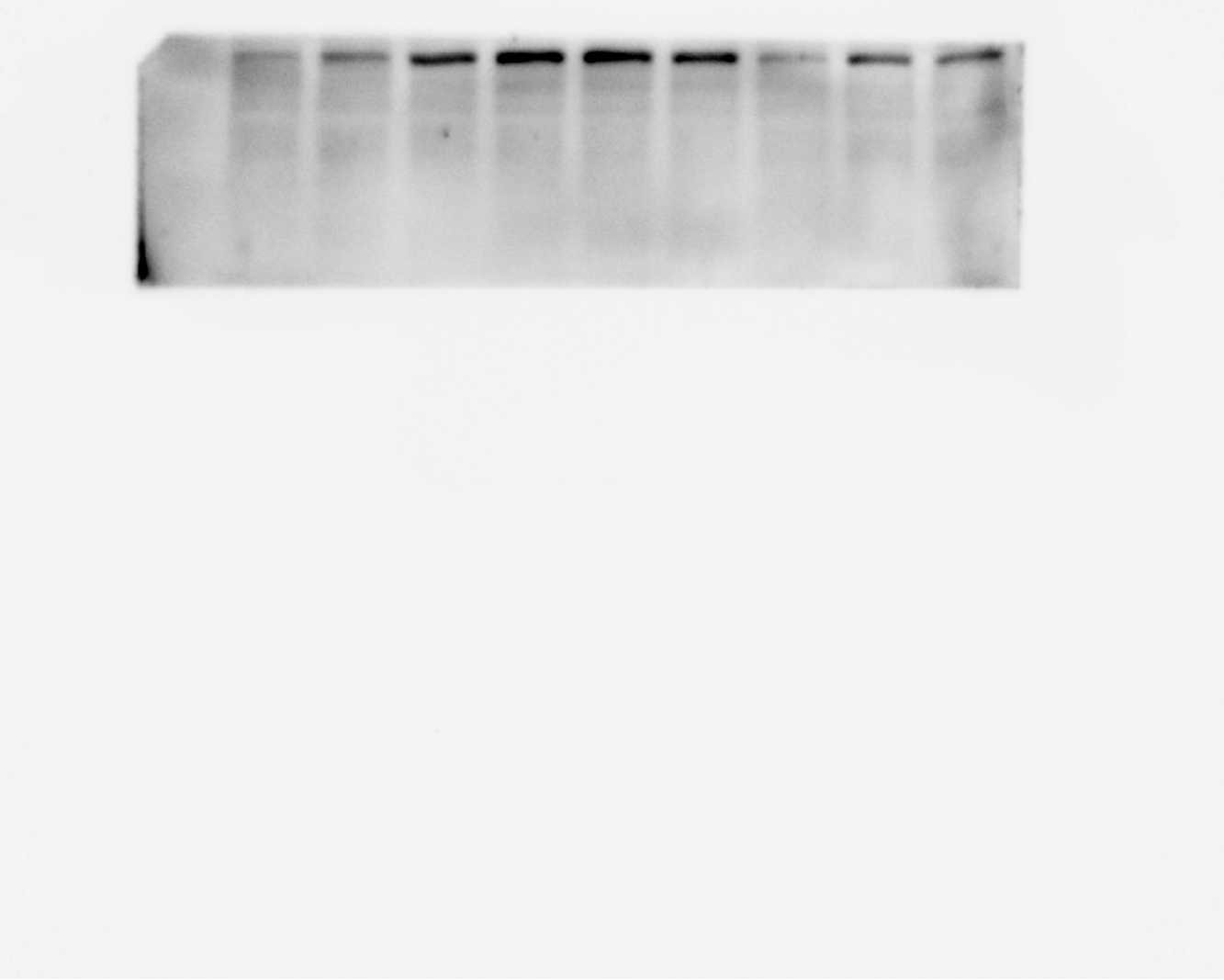

Supplement: Supplementary file 9 [file DataSheet2.ZIP › animal model/ratpcna_3(Chemiluminescence).tif]

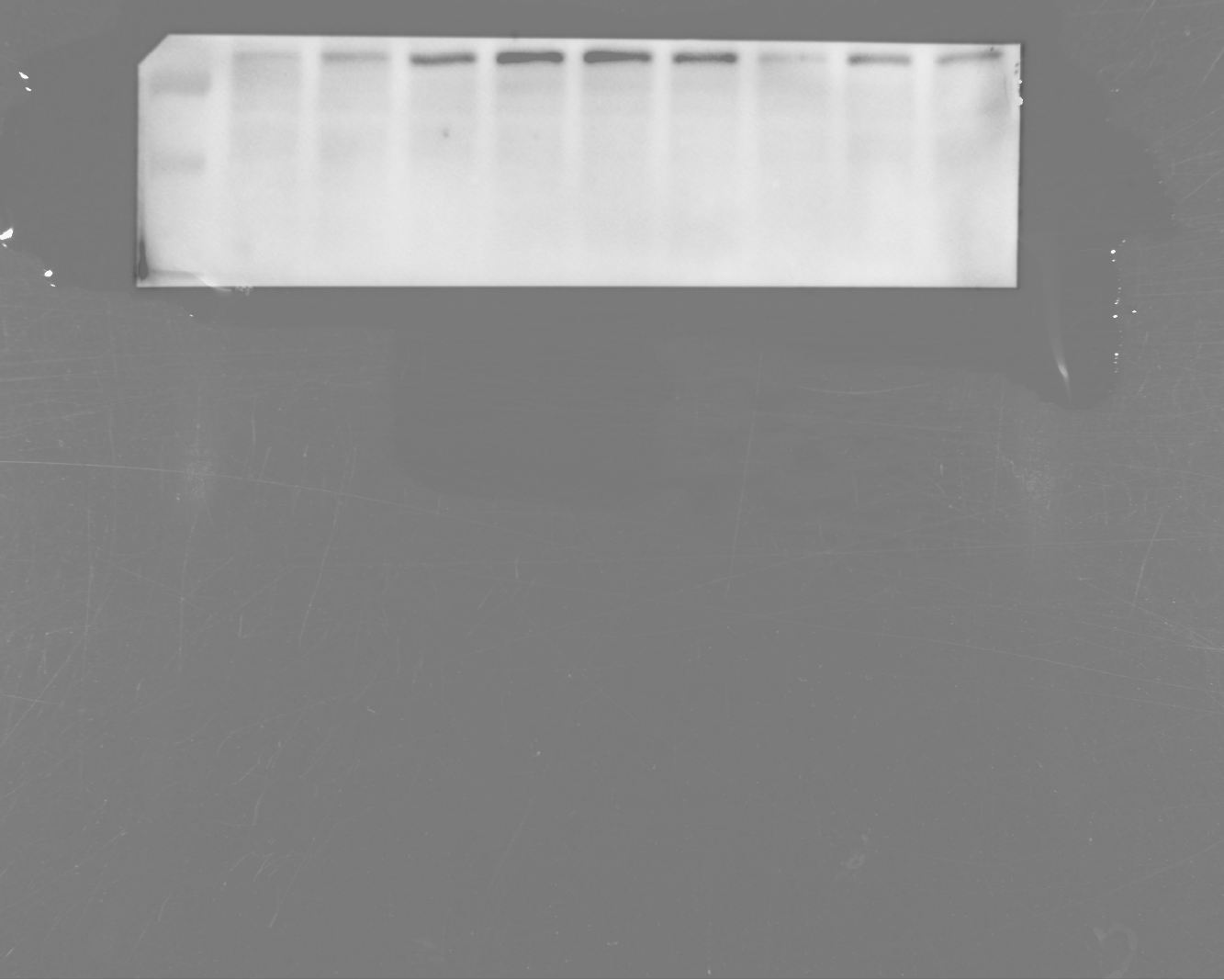

Supplement: Supplementary file 9 [file DataSheet2.ZIP › animal model/ratpcna_3(Composite).tif]

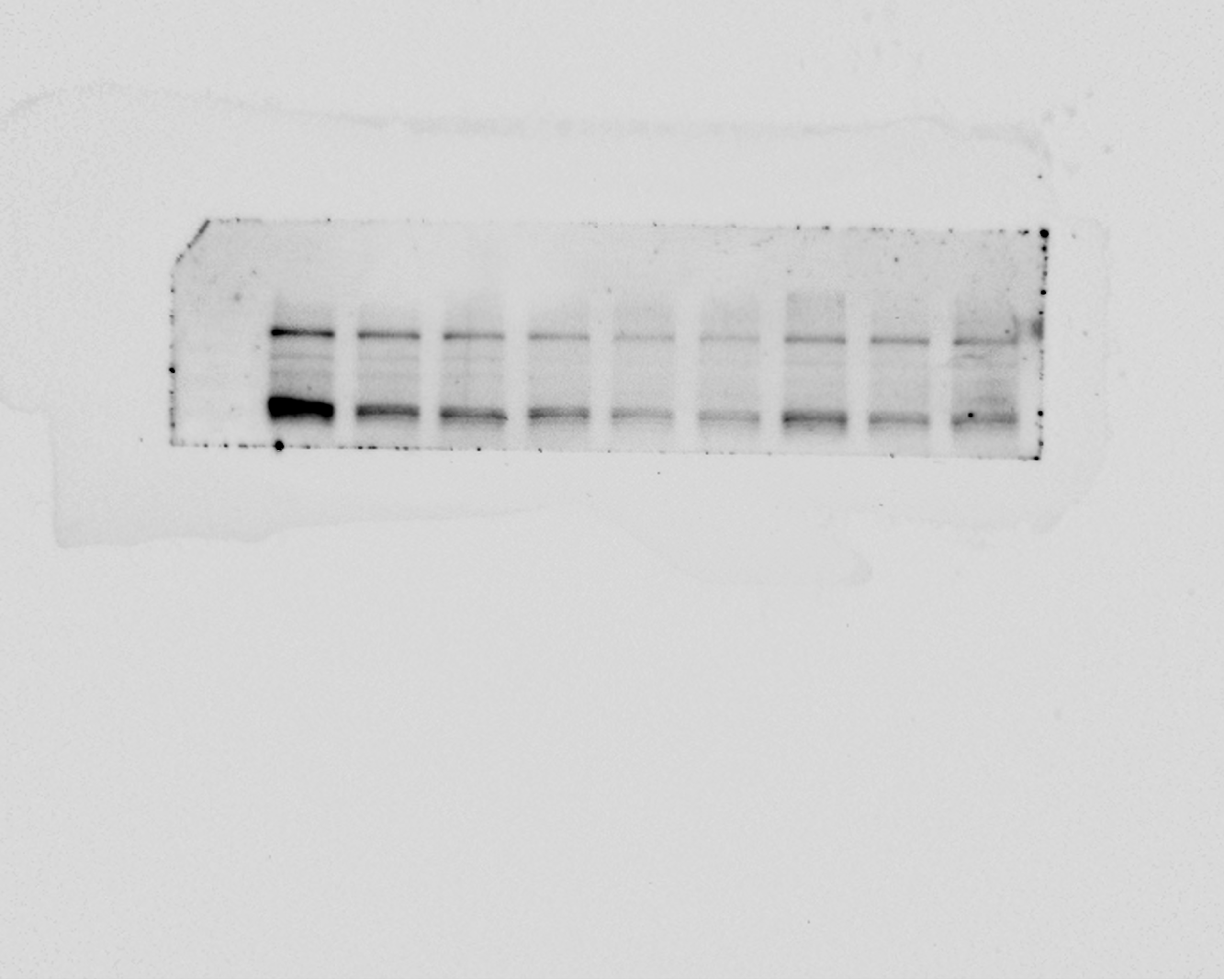

Supplement: Supplementary file 9 [file DataSheet2.ZIP › animal model/ratpstat3_3(Chemiluminescence).tif]

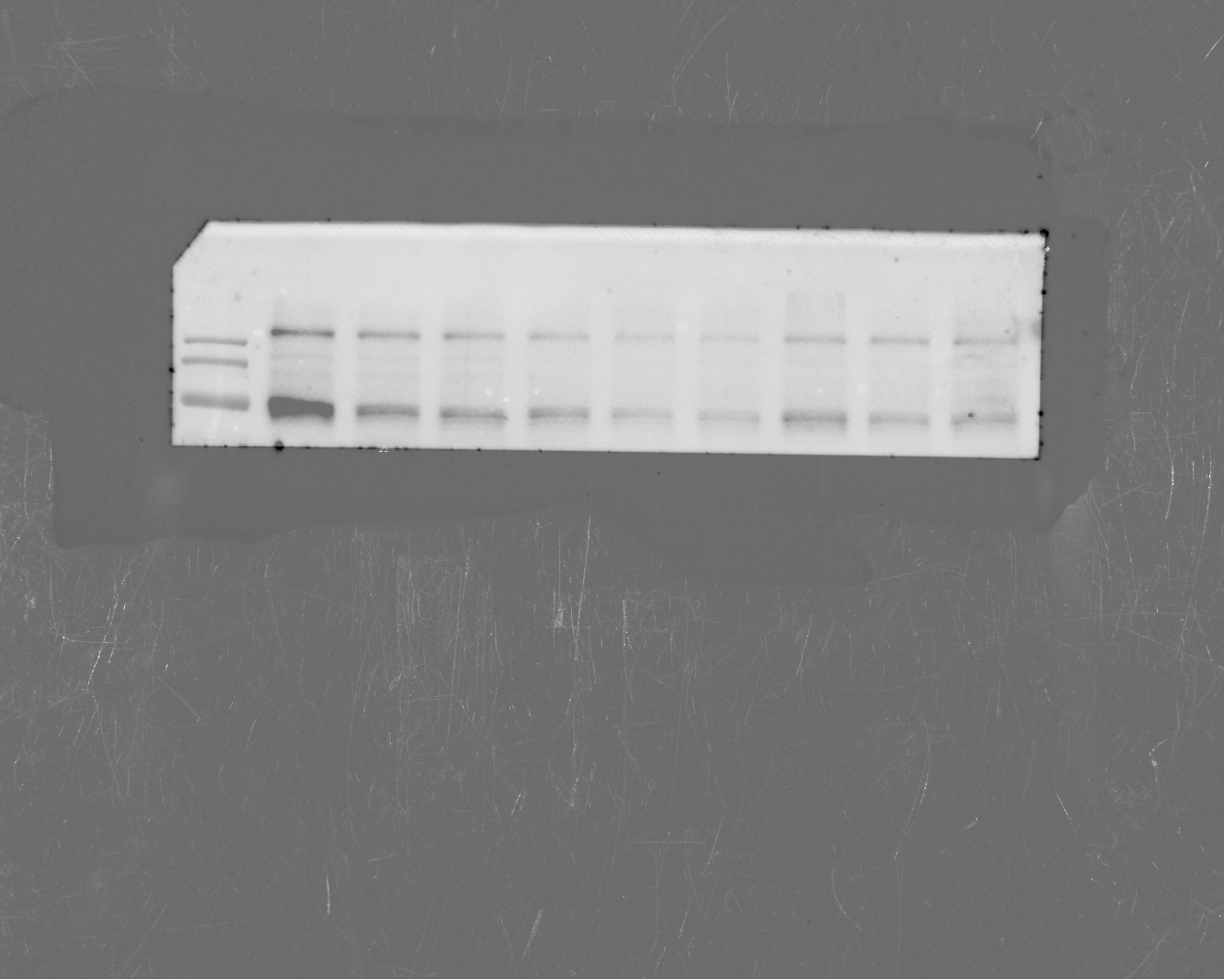

Supplement: Supplementary file 9 [file DataSheet2.ZIP › animal model/ratpstat3_3(Composite).tif]

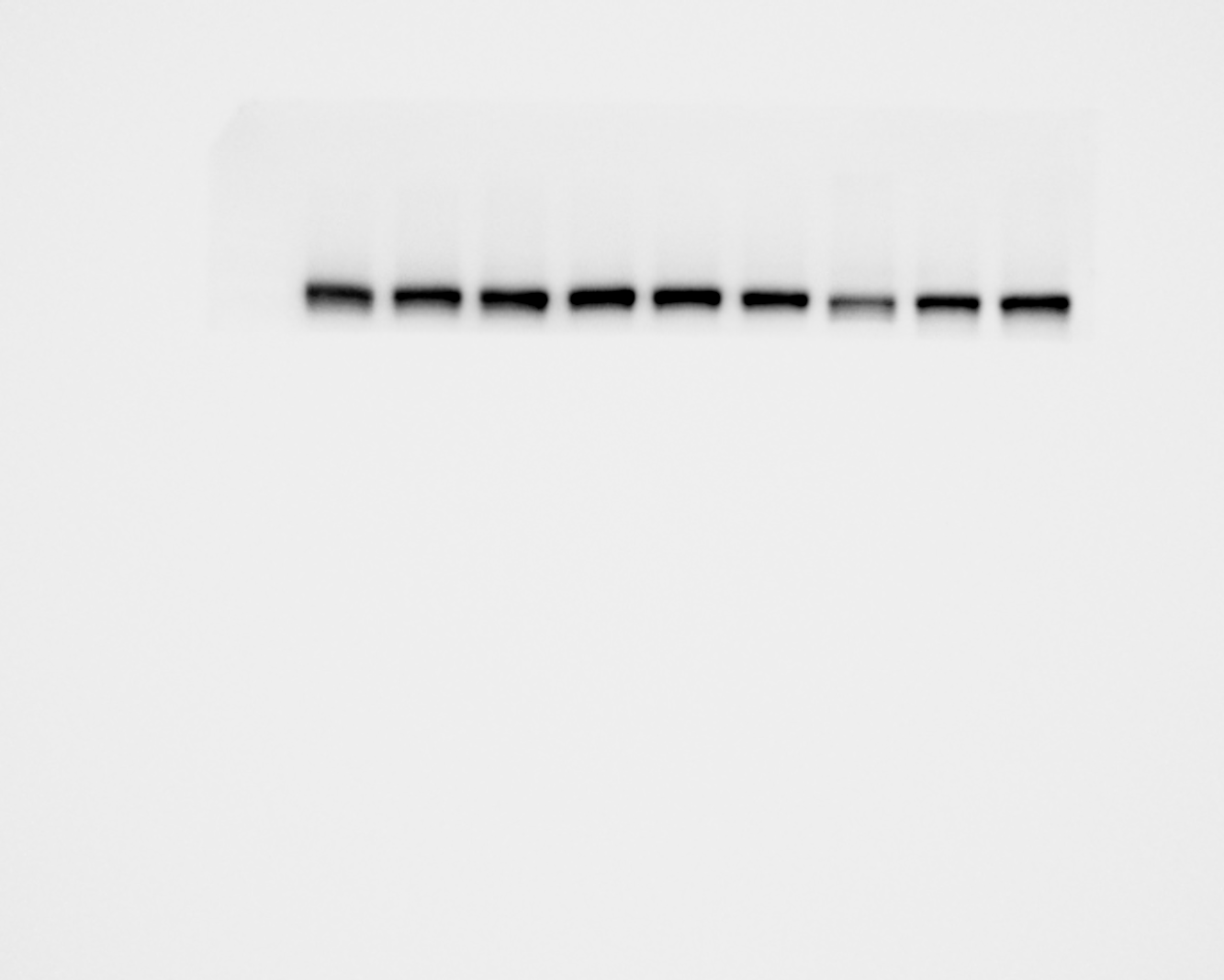

Supplement: Supplementary file 9 [file DataSheet2.ZIP › animal model/ratstat3_1(Chemiluminescence).tif]

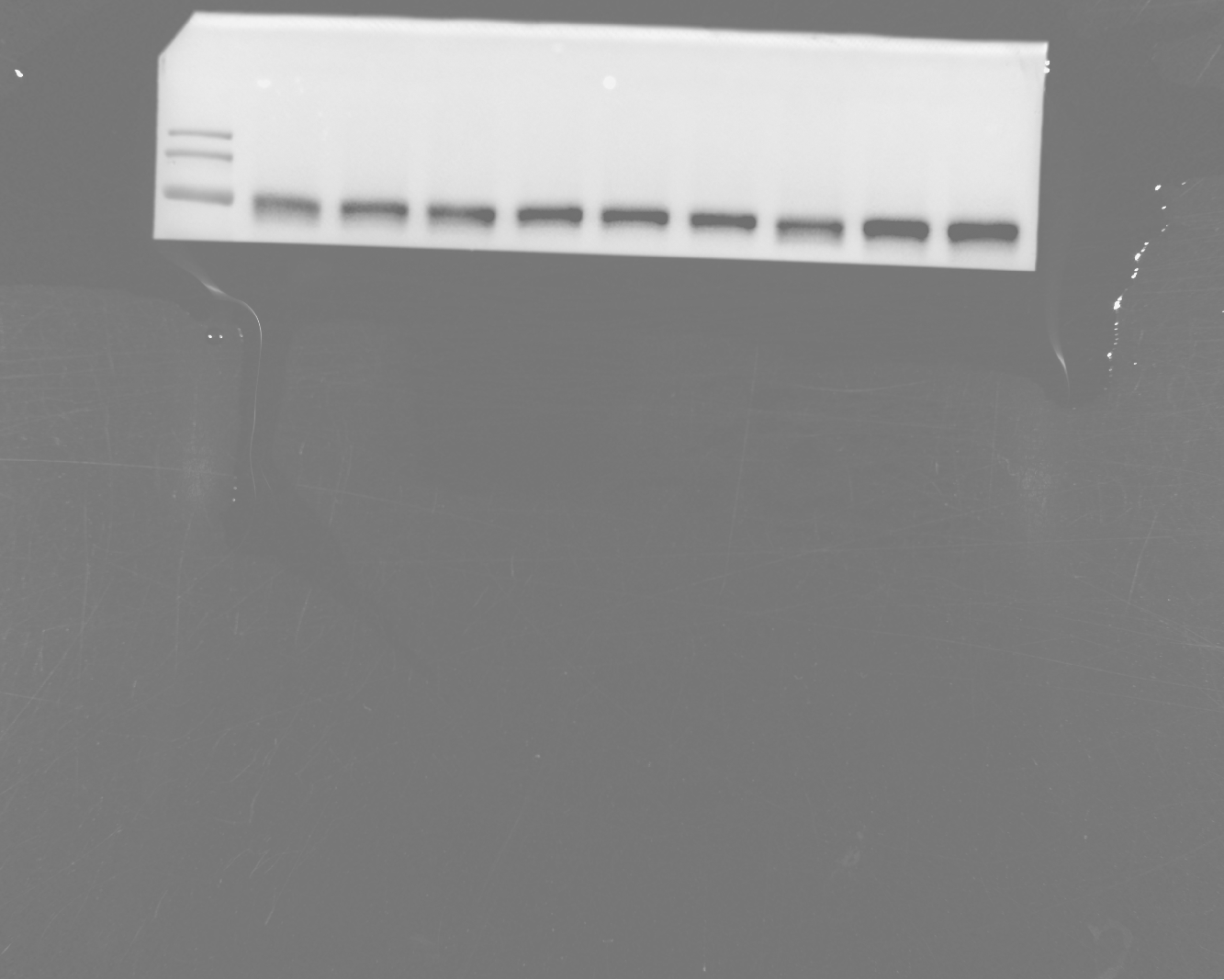

Supplement: Supplementary file 9 [file DataSheet2.ZIP › animal model/ratstat3-2_3(Composite).tif]

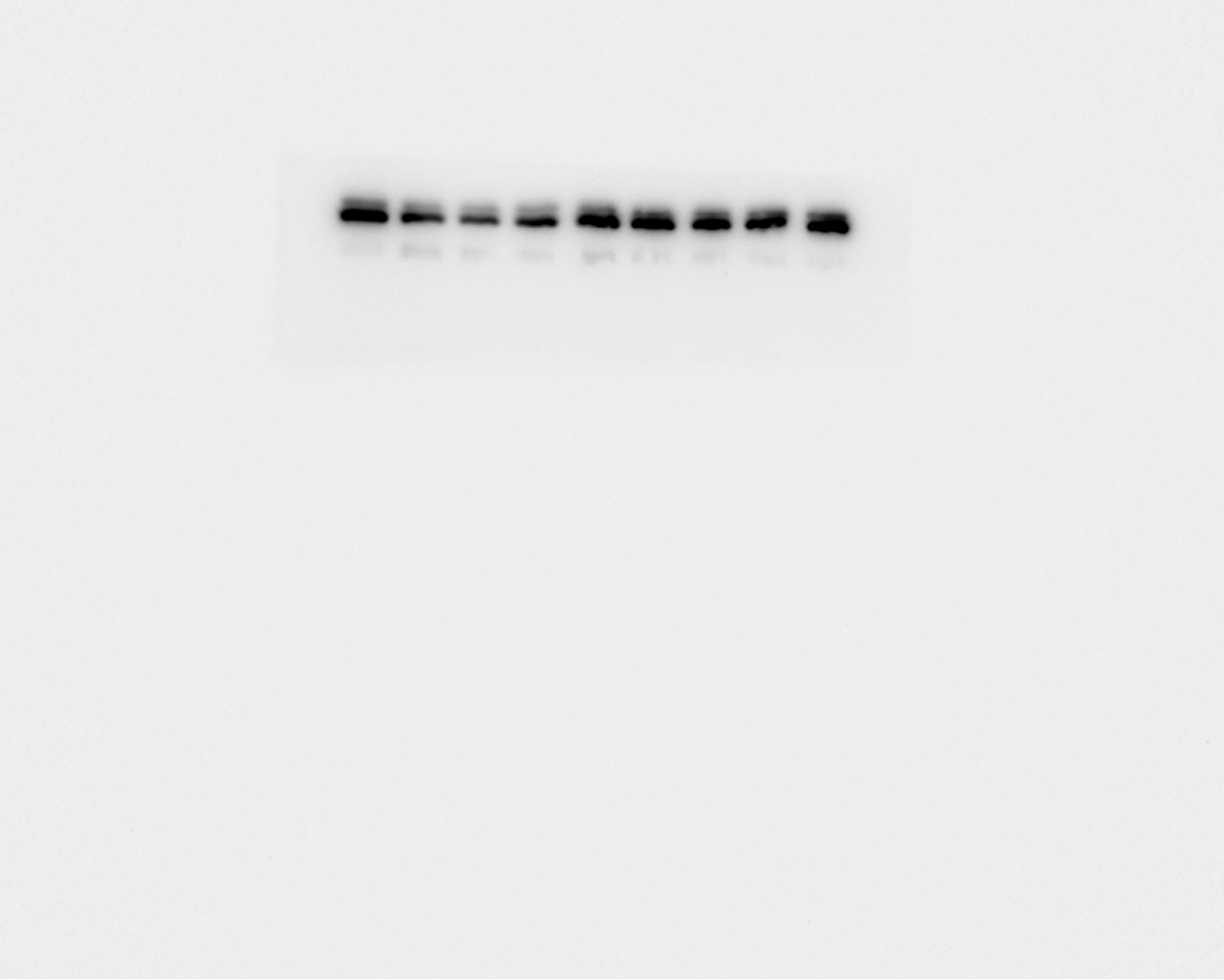

Supplement: Supplementary file 10 [file DataSheet5.ZIP › erk-13_5(Chemiluminescence).tif]

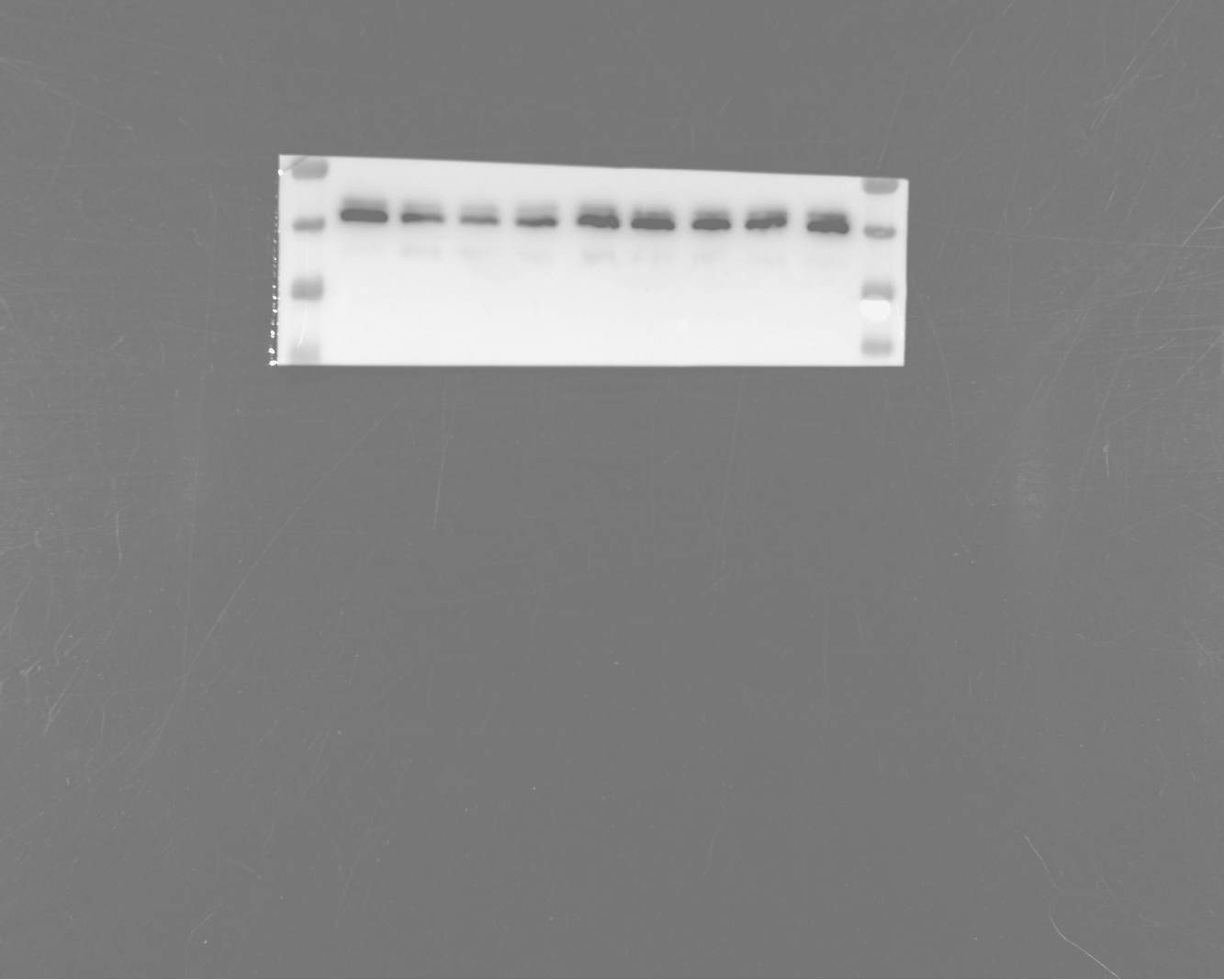

Supplement: Supplementary file 10 [file DataSheet5.ZIP › erk-13_5(Composite).tif]

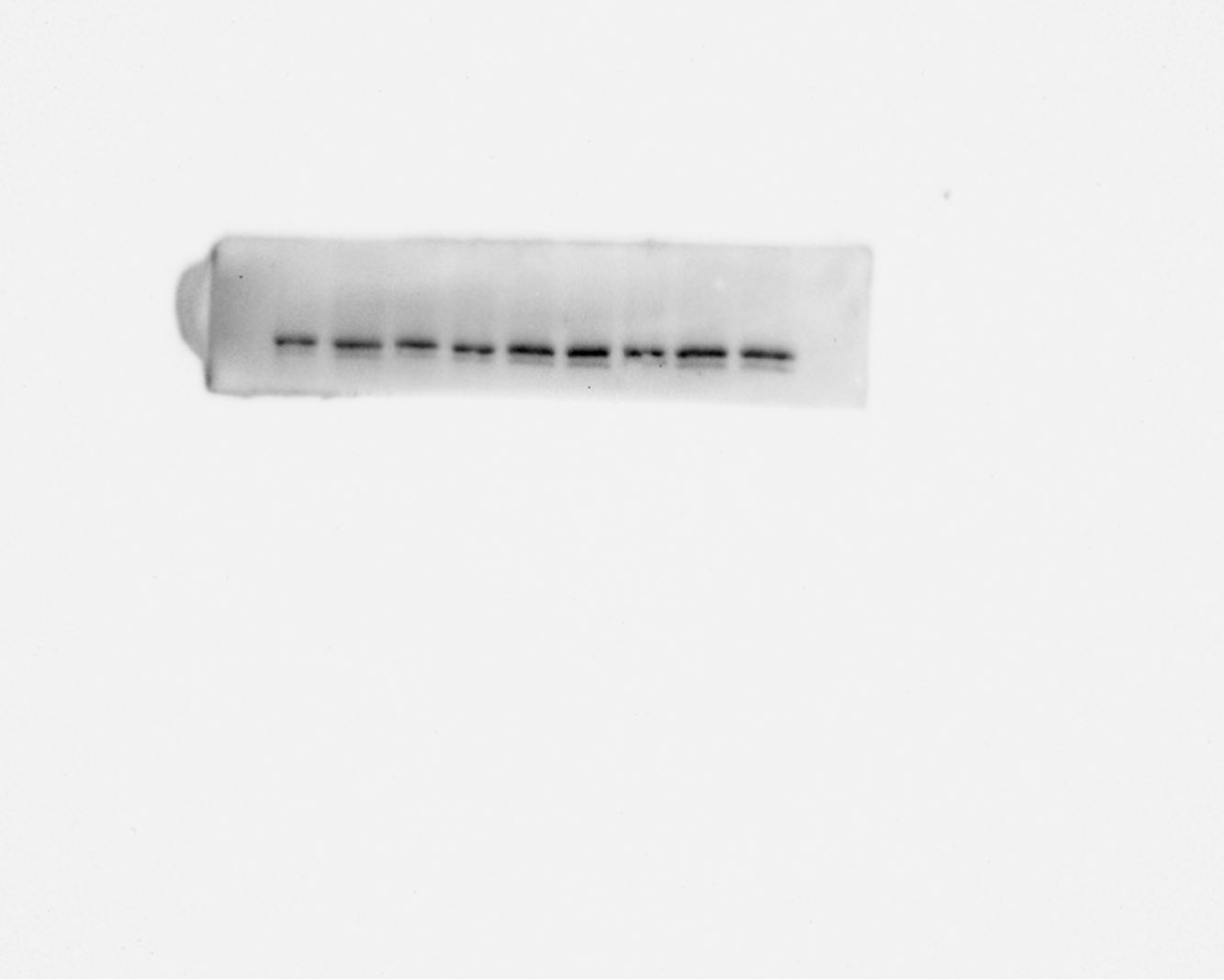

Supplement: Supplementary file 10 [file DataSheet5.ZIP › erk-2 (3).tif]

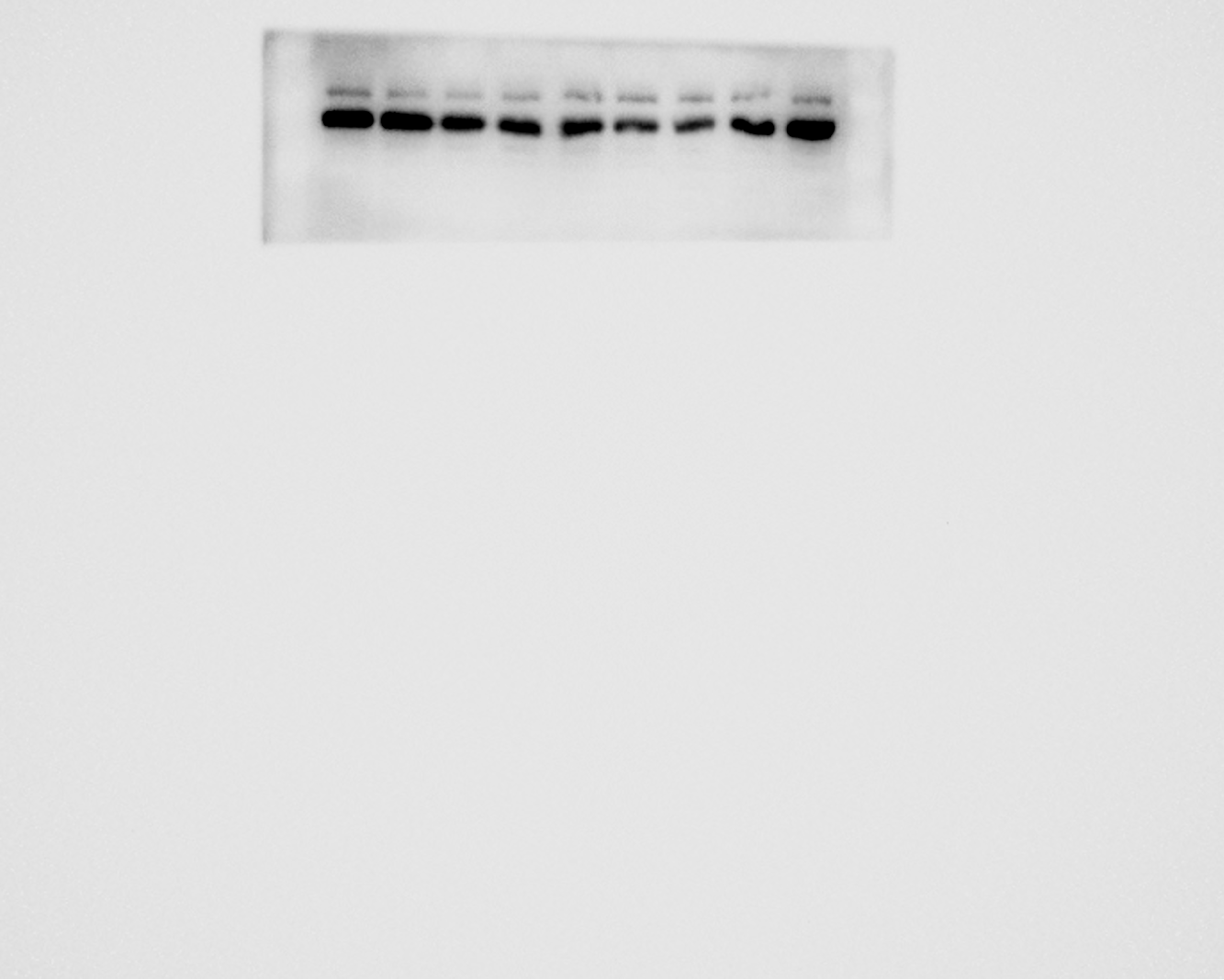

Supplement: Supplementary file 10 [file DataSheet5.ZIP › gapdh13_5(Chemiluminescence).tif]

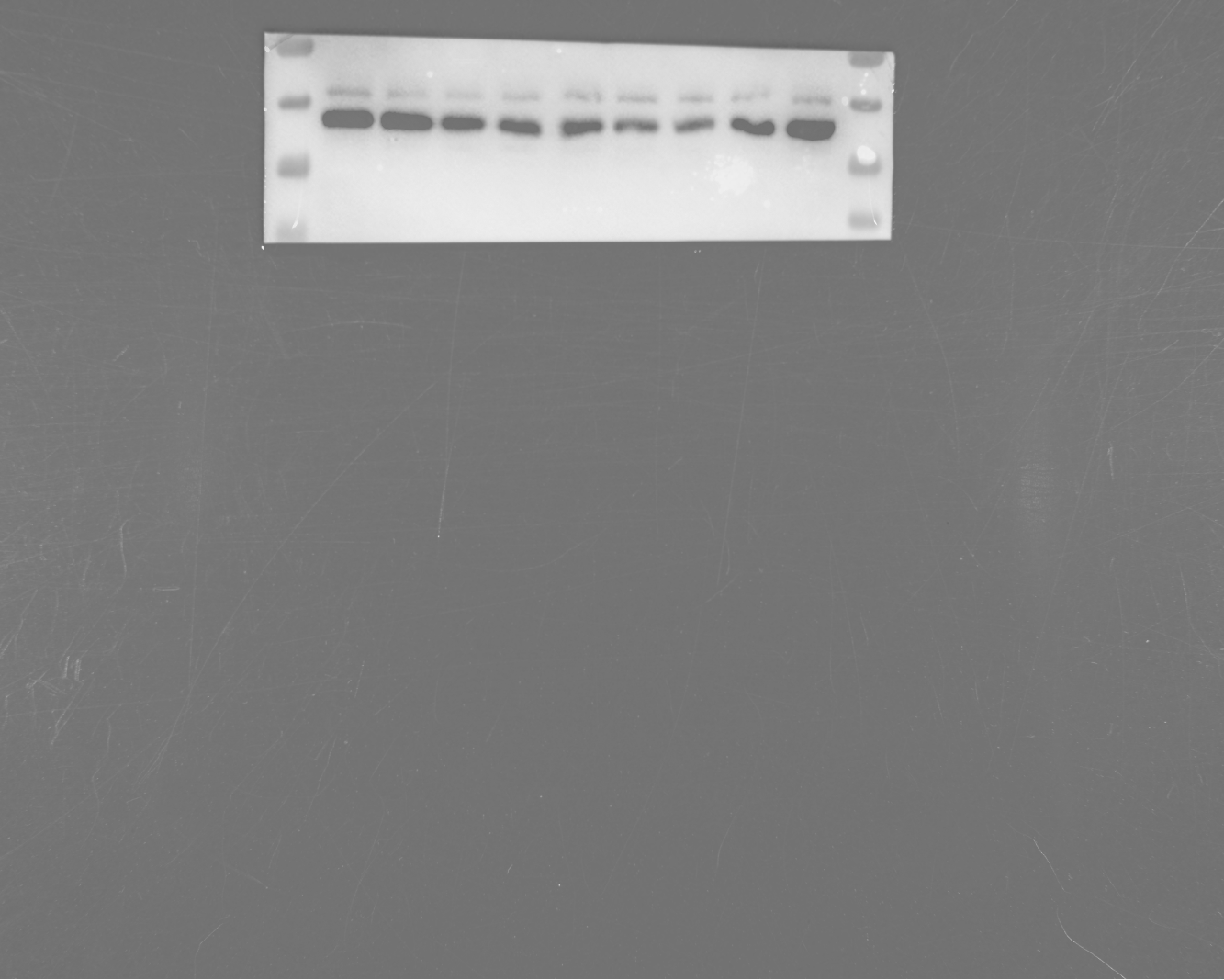

Supplement: Supplementary file 10 [file DataSheet5.ZIP › gapdh13_5(Composite).tif]

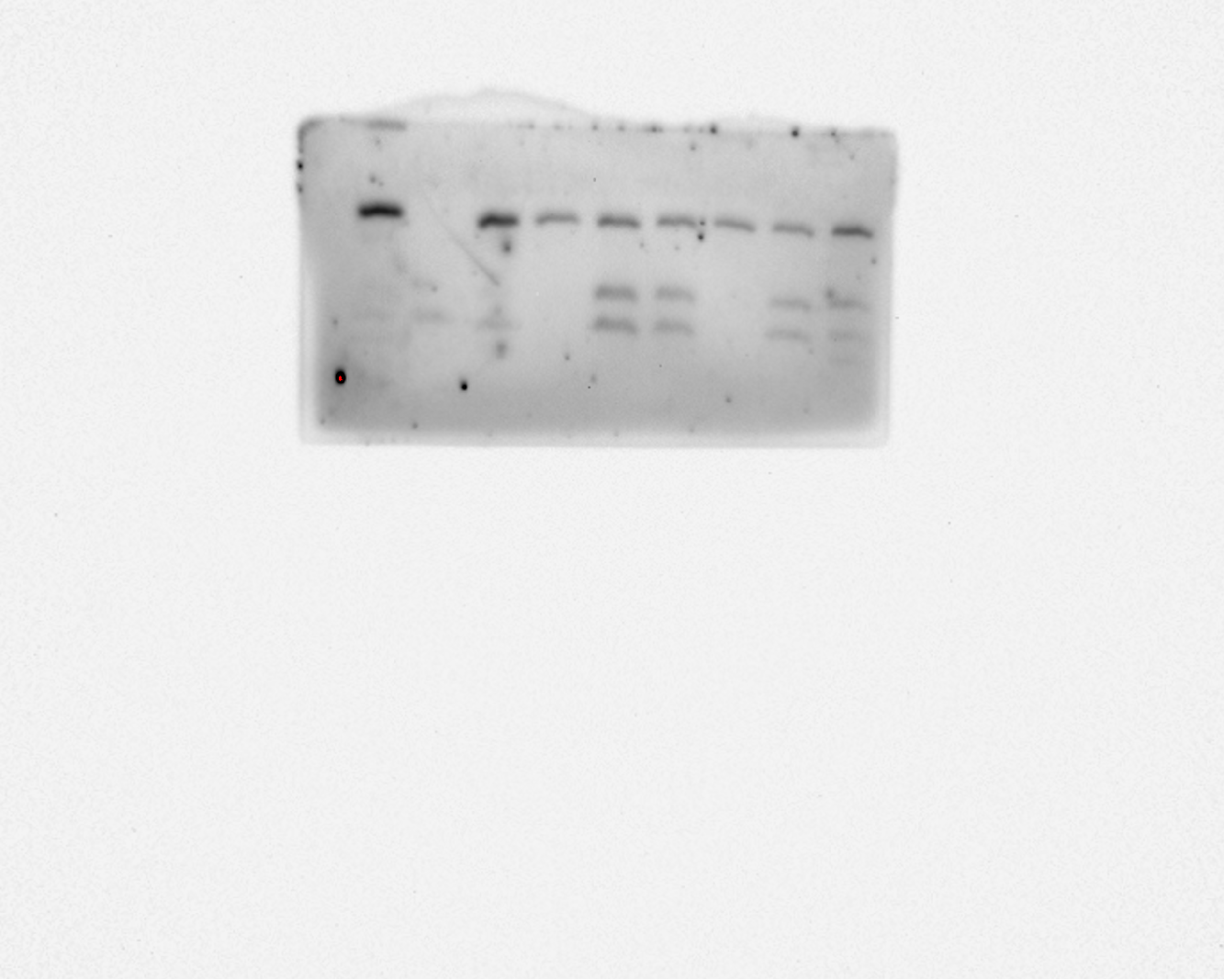

Supplement: Supplementary file 10 [file DataSheet5.ZIP › homo-cas9_12(Chemiluminescence).tif]

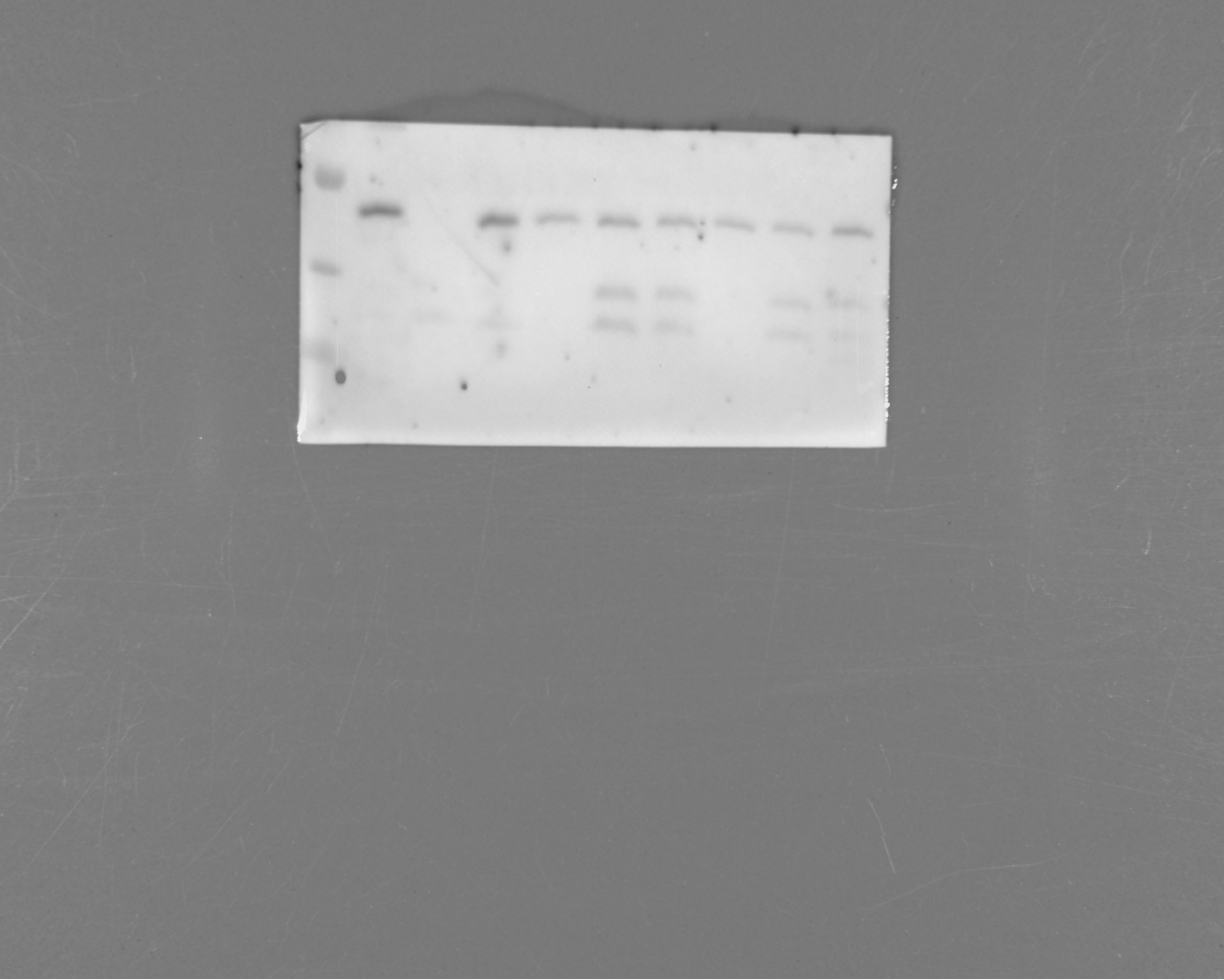

Supplement: Supplementary file 10 [file DataSheet5.ZIP › homo-cas9_12(Composite).tif]

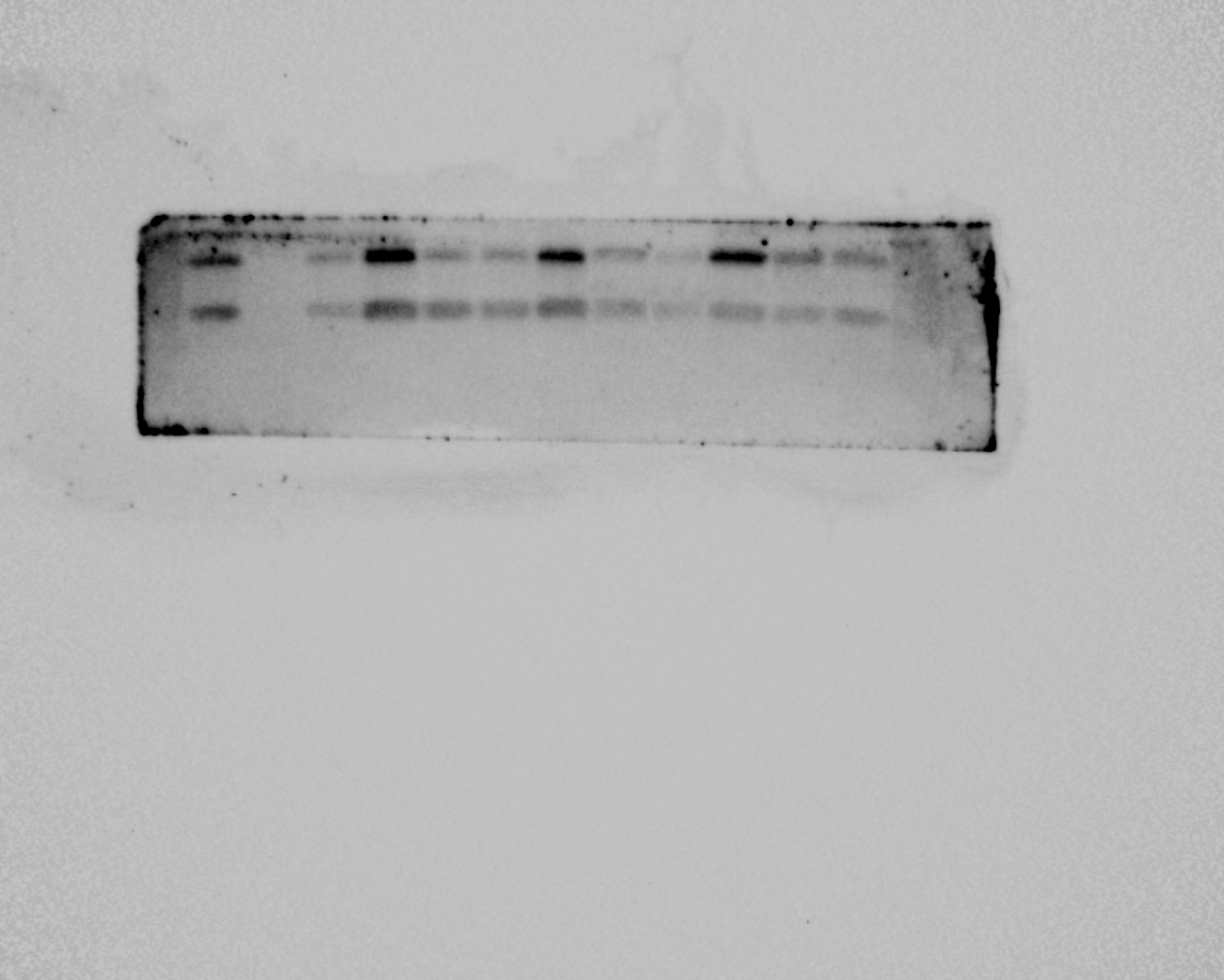

Supplement: Supplementary file 10 [file DataSheet5.ZIP › p21sea_1(Chemiluminescence).tif]

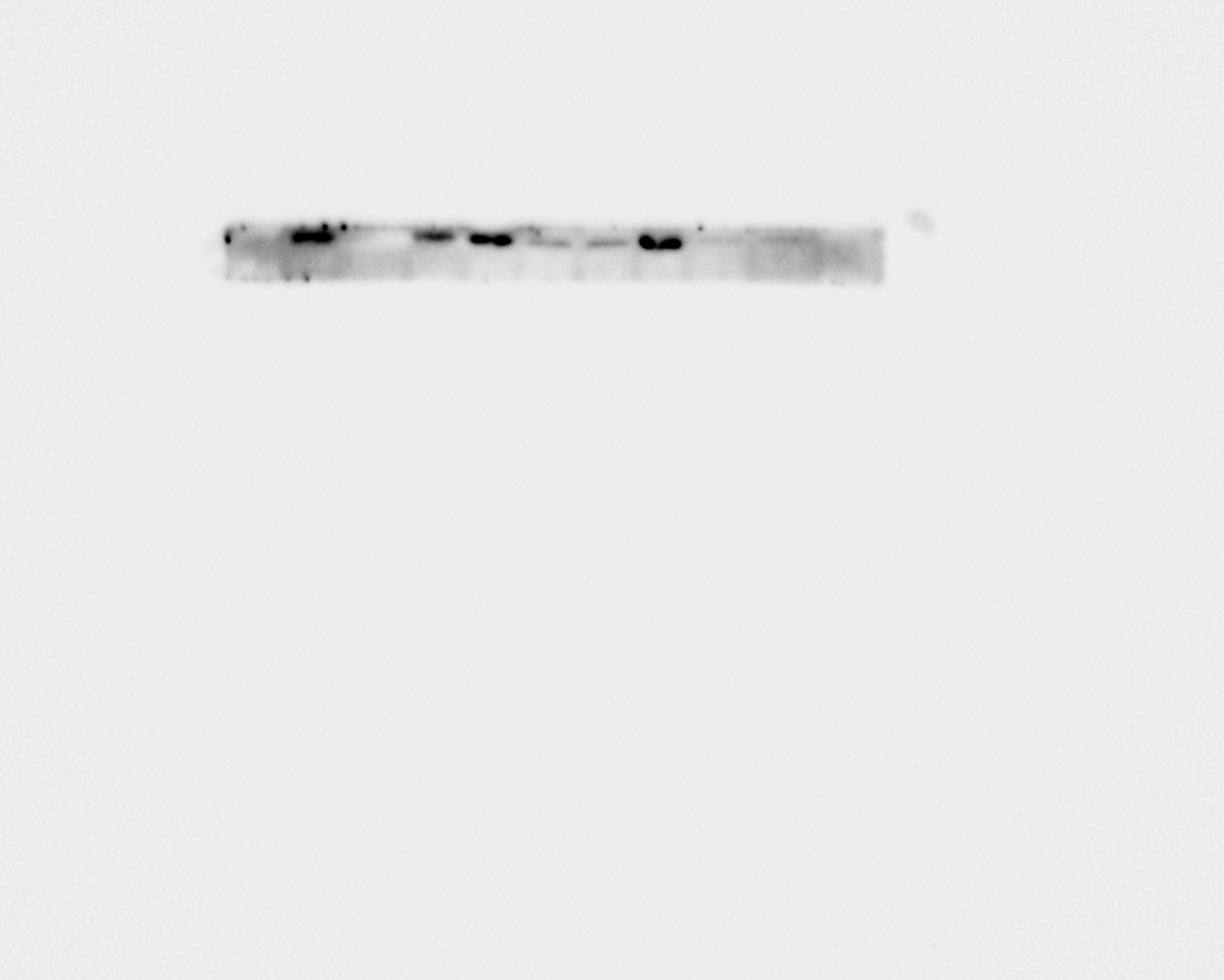

Supplement: Supplementary file 10 [file DataSheet5.ZIP › pakt-3_1(Chemiluminescence).tif]

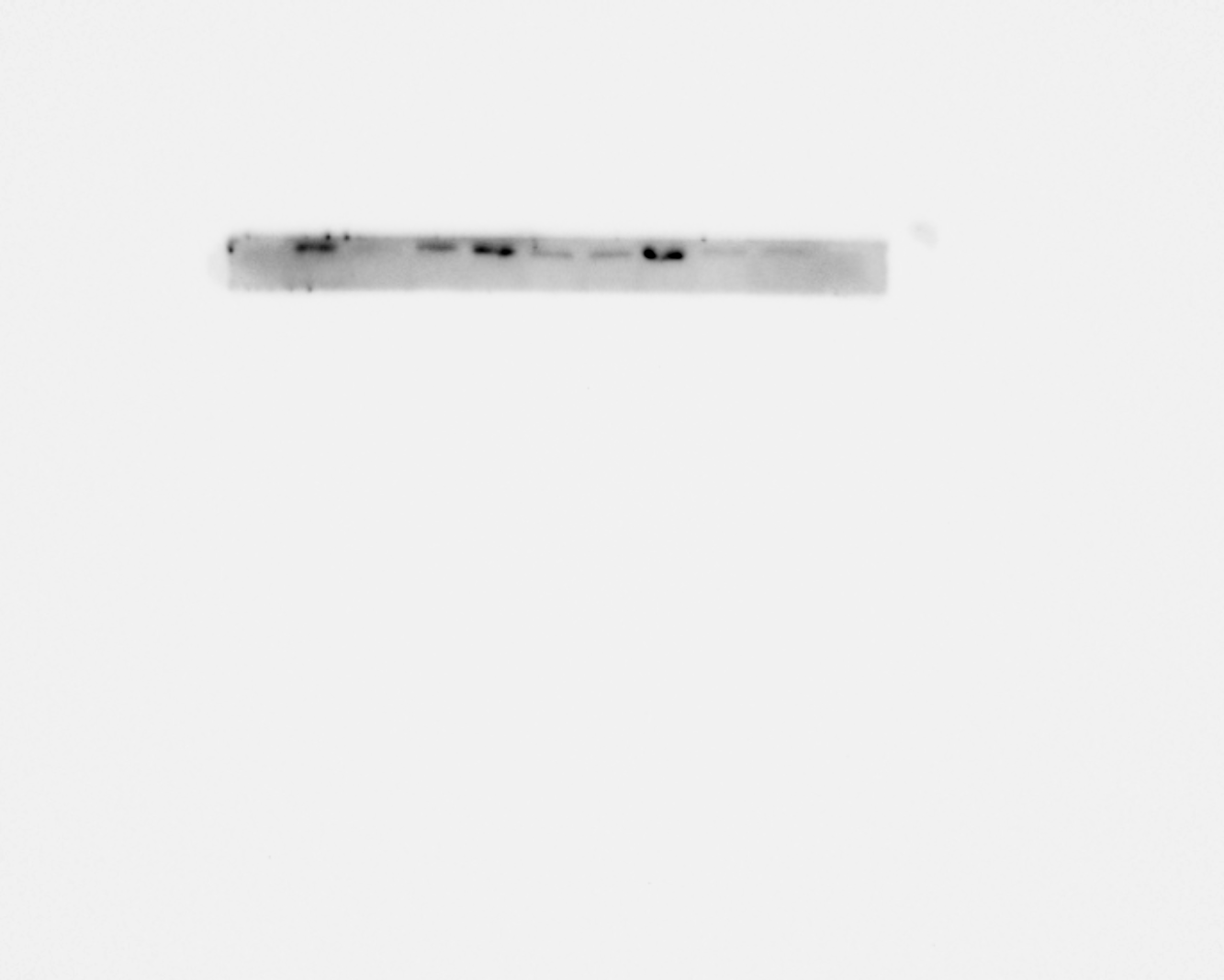

Supplement: Supplementary file 10 [file DataSheet5.ZIP › pakt-3_2(Chemiluminescence).tif]

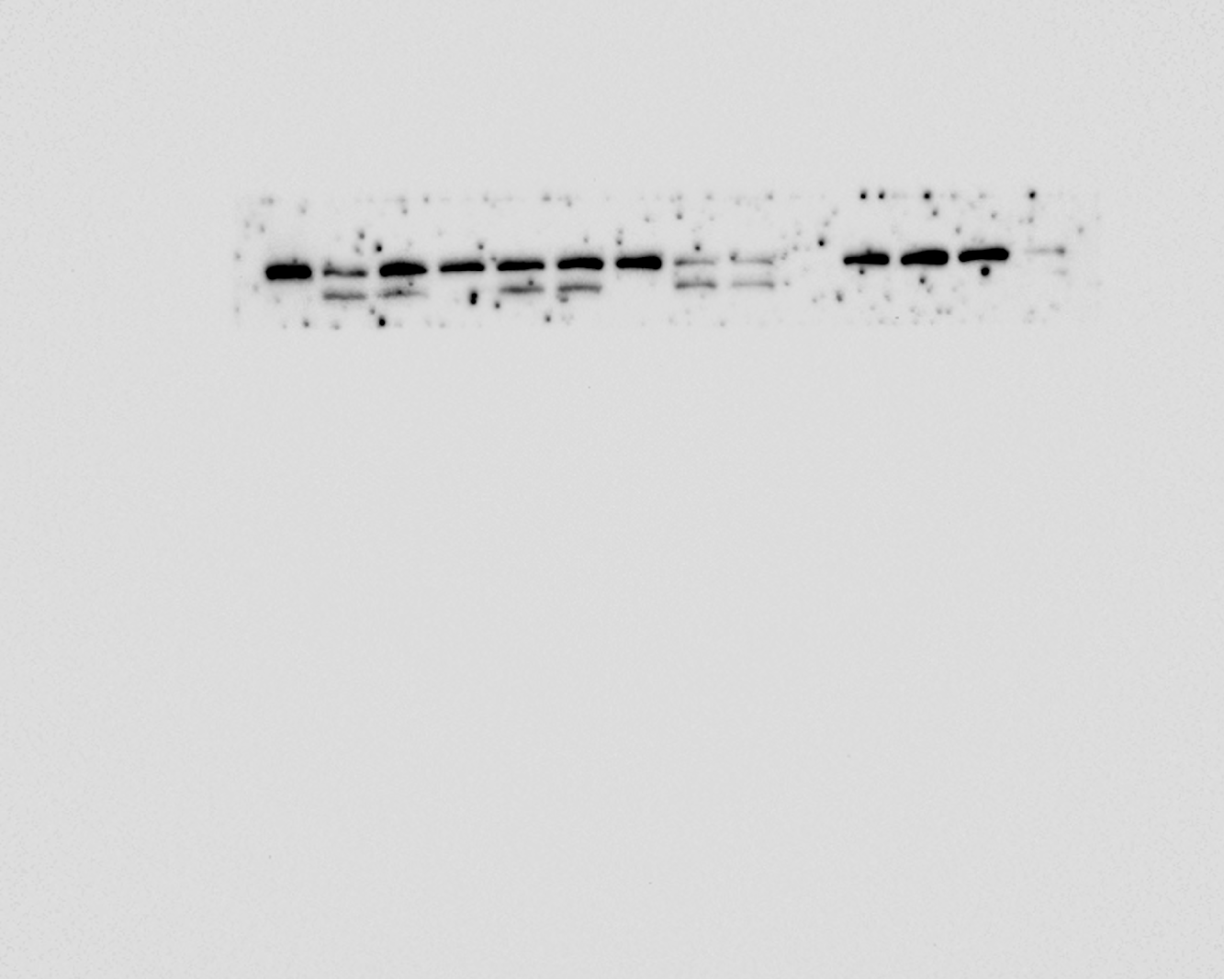

Supplement: Supplementary file 10 [file DataSheet5.ZIP › par-9_3(Chemiluminescence).tif]

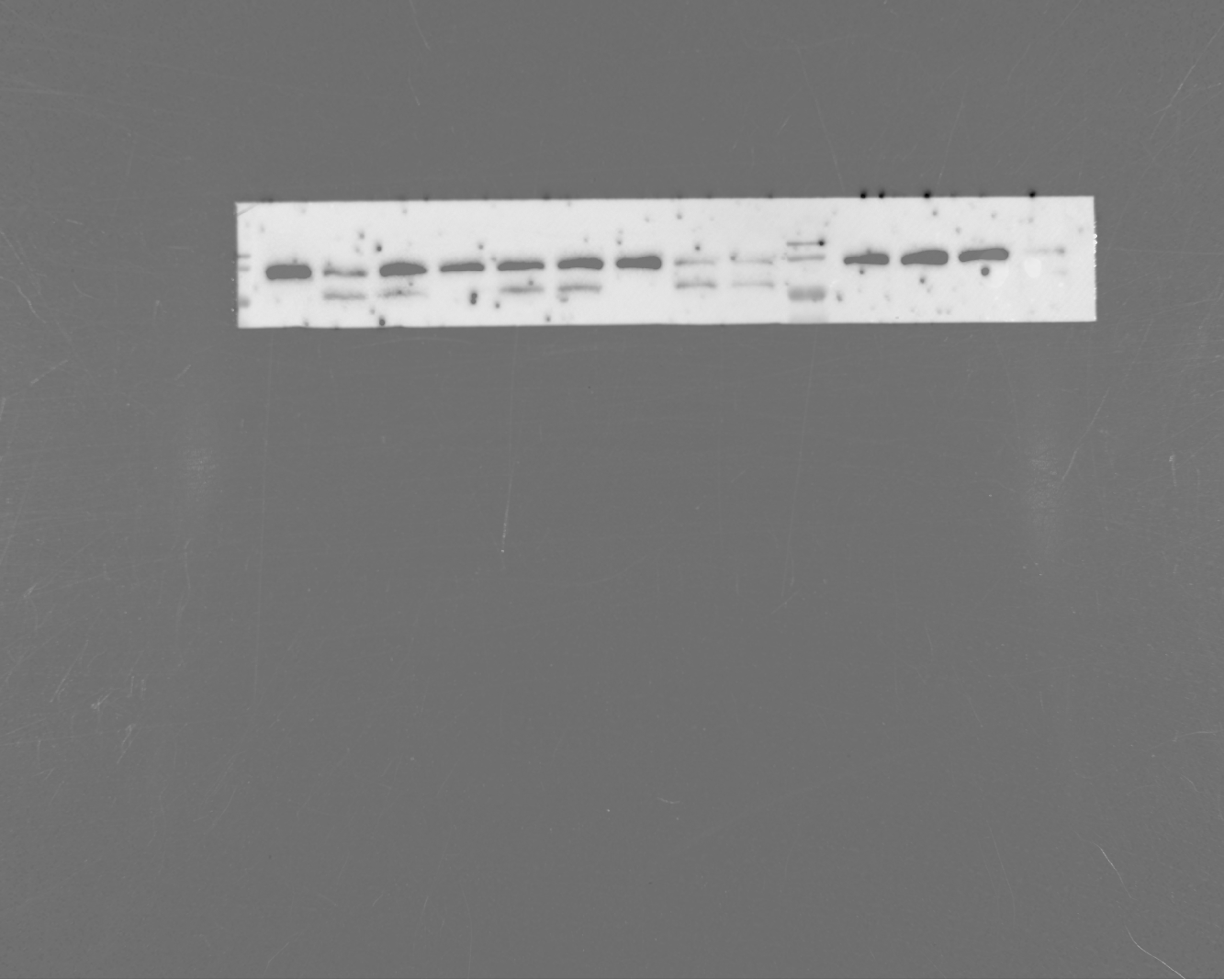

Supplement: Supplementary file 10 [file DataSheet5.ZIP › par-9_3(Composite).tif]

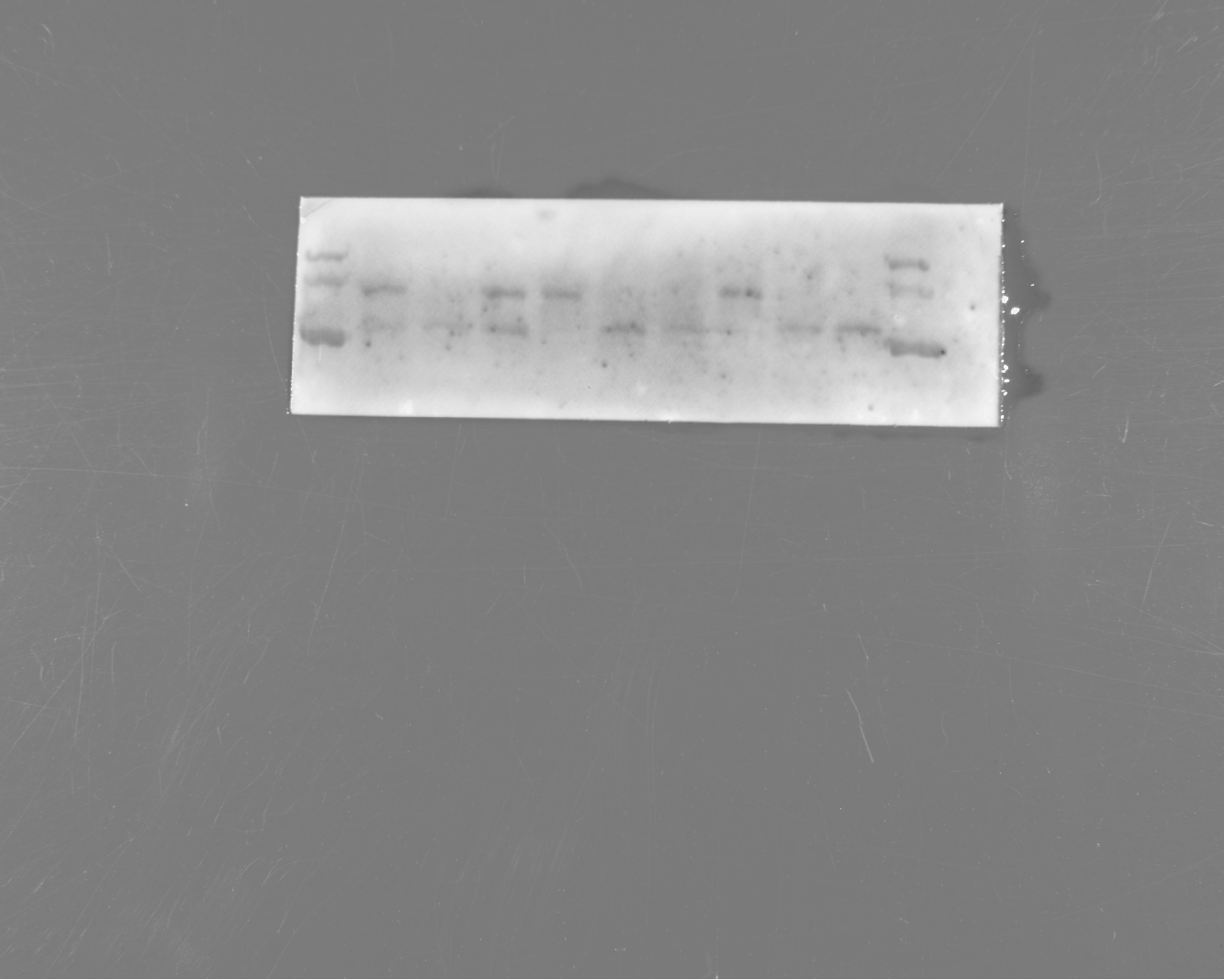

Supplement: Supplementary file 10 [file DataSheet5.ZIP › parp (1).tif]

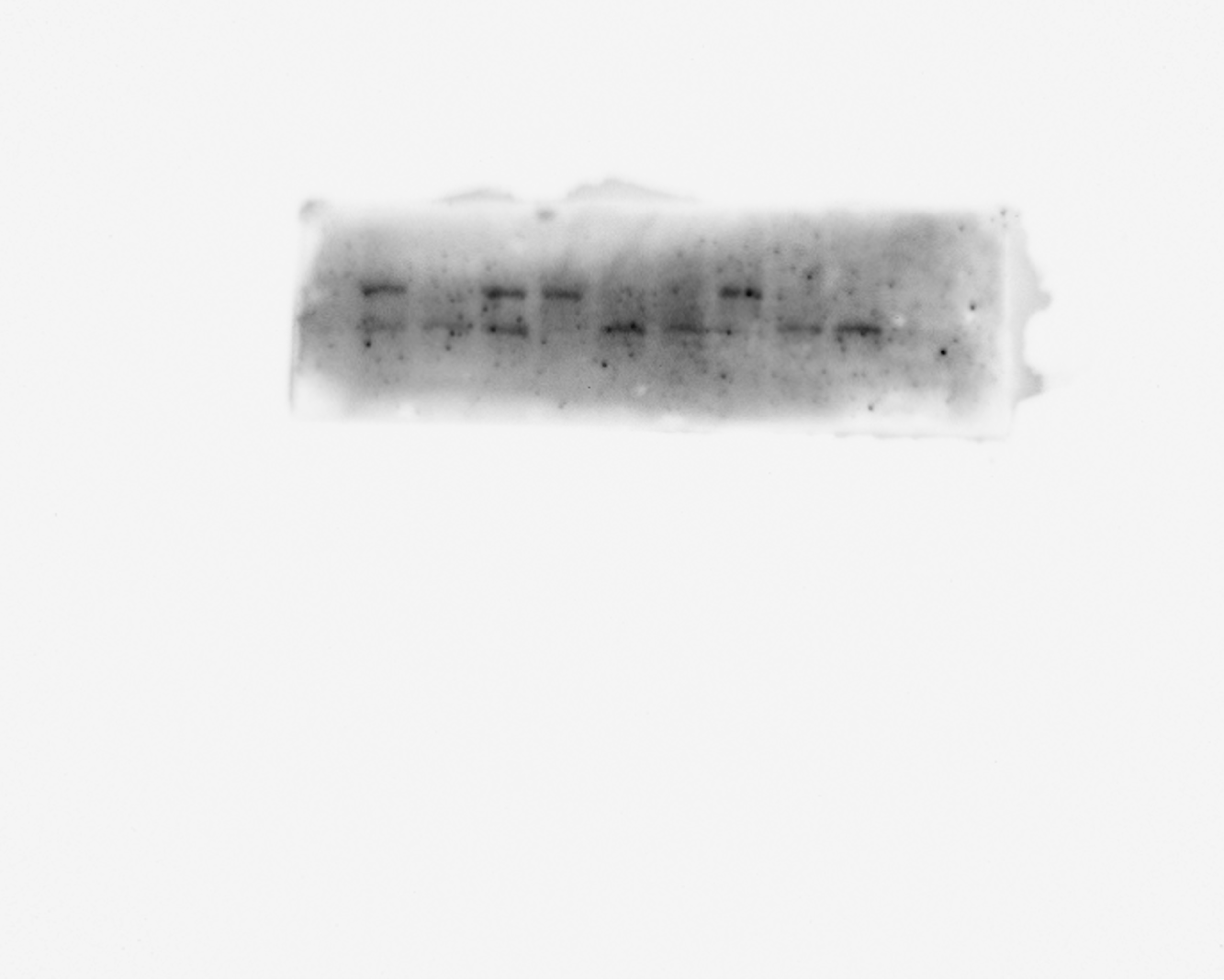

Supplement: Supplementary file 10 [file DataSheet5.ZIP › parp (11).tif]

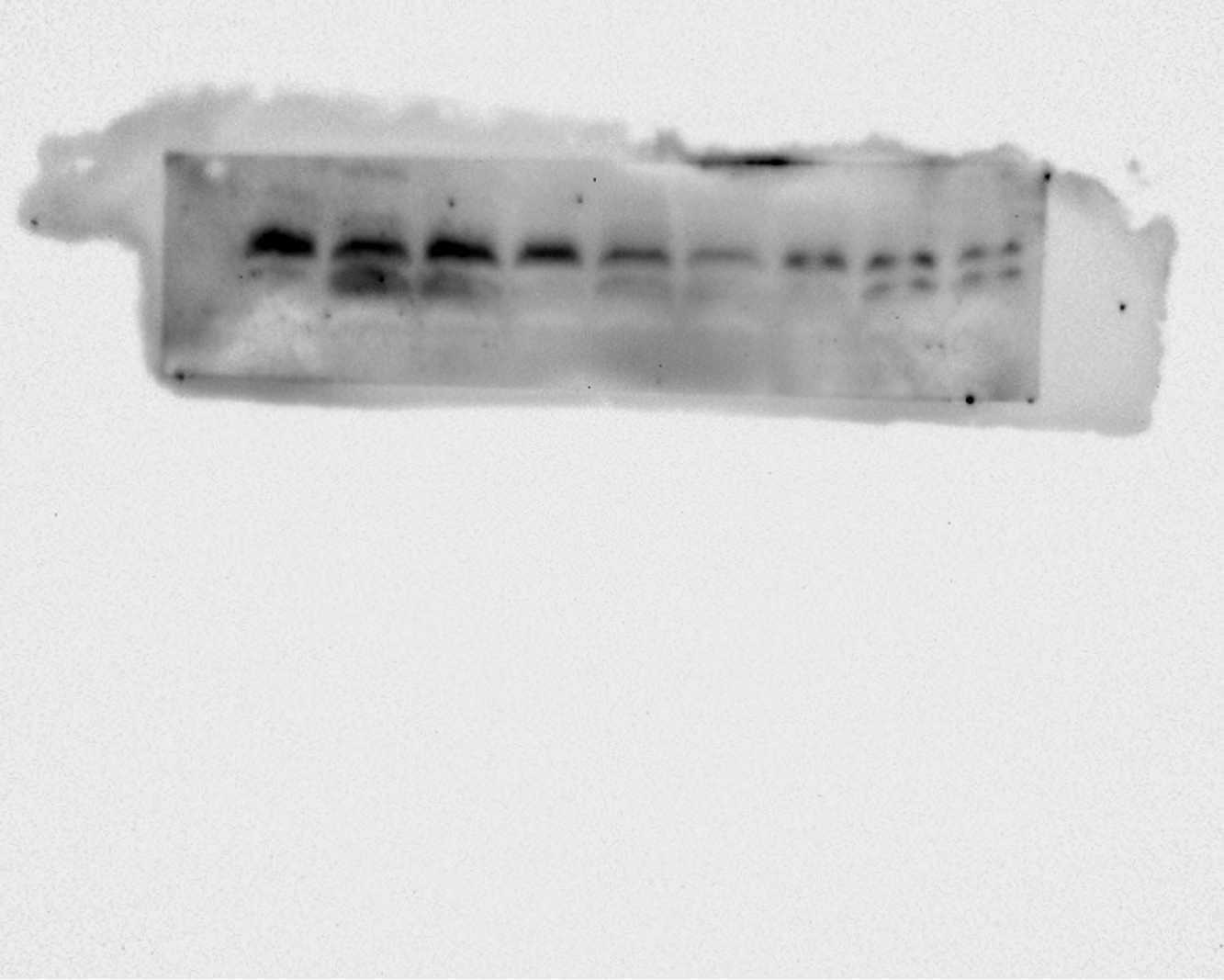

Supplement: Supplementary file 10 [file DataSheet5.ZIP › parp (2).tif]

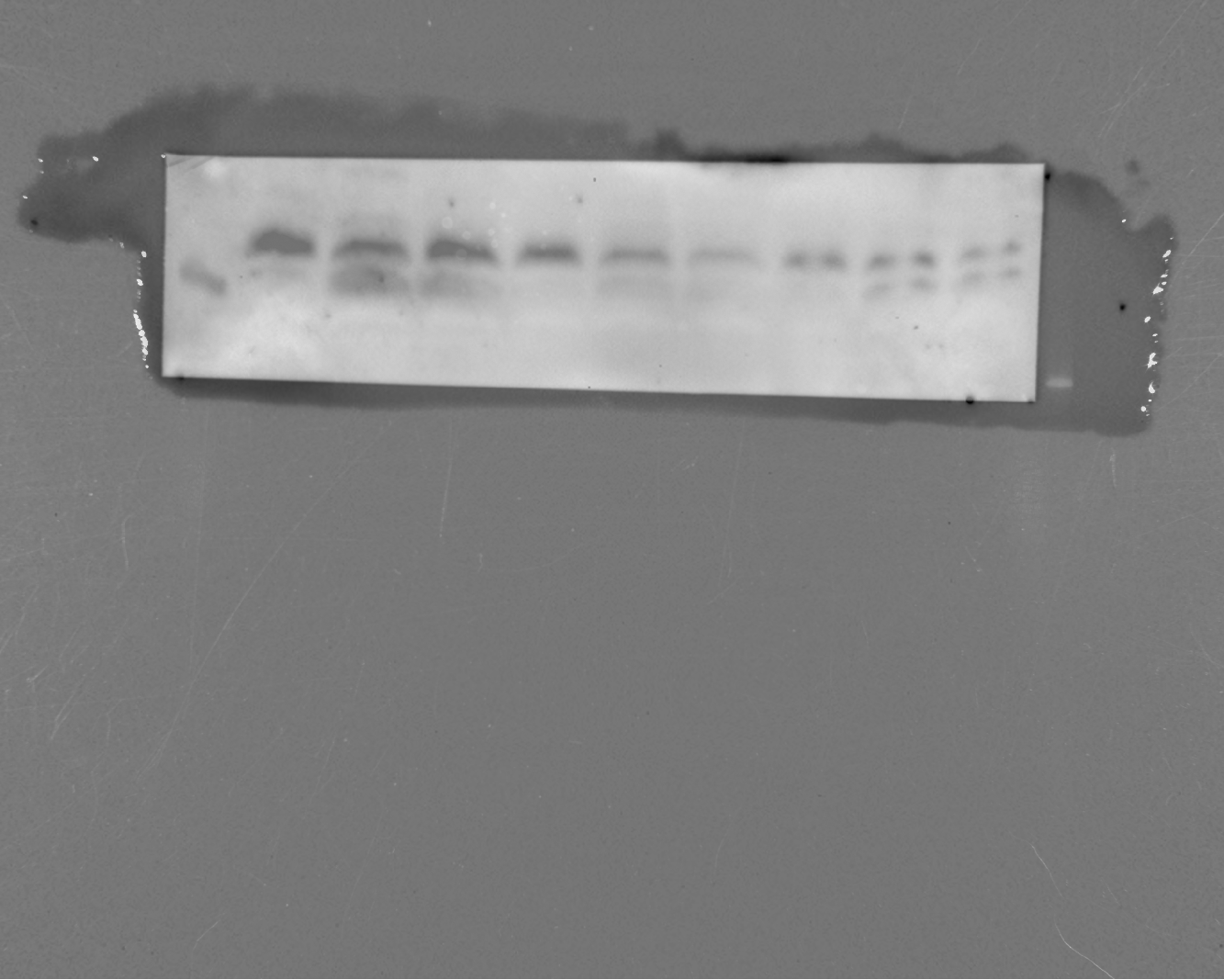

Supplement: Supplementary file 10 [file DataSheet5.ZIP › parp (4).tif]

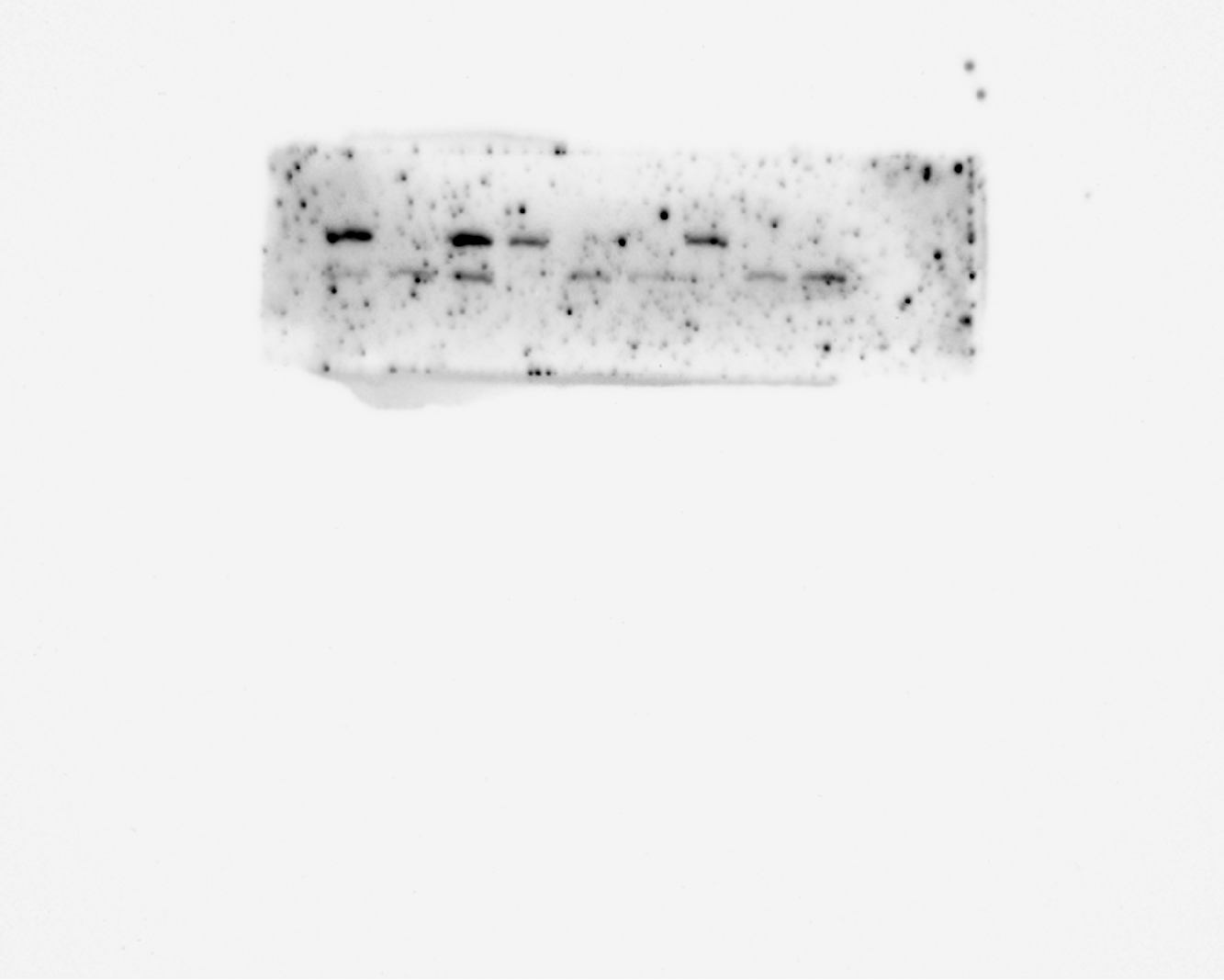

Supplement: Supplementary file 10 [file DataSheet5.ZIP › parp (5).tif]

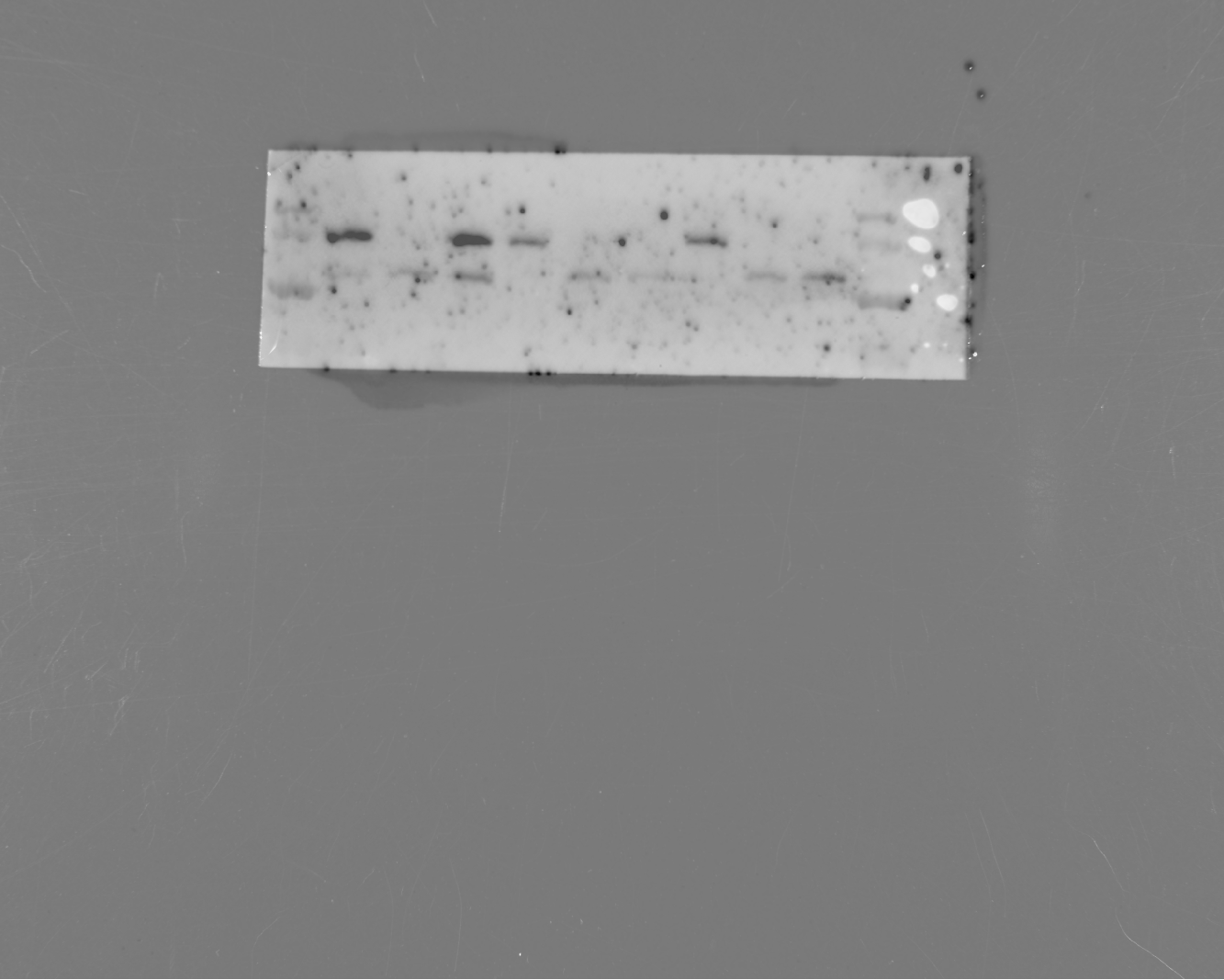

Supplement: Supplementary file 10 [file DataSheet5.ZIP › parp (7).tif]

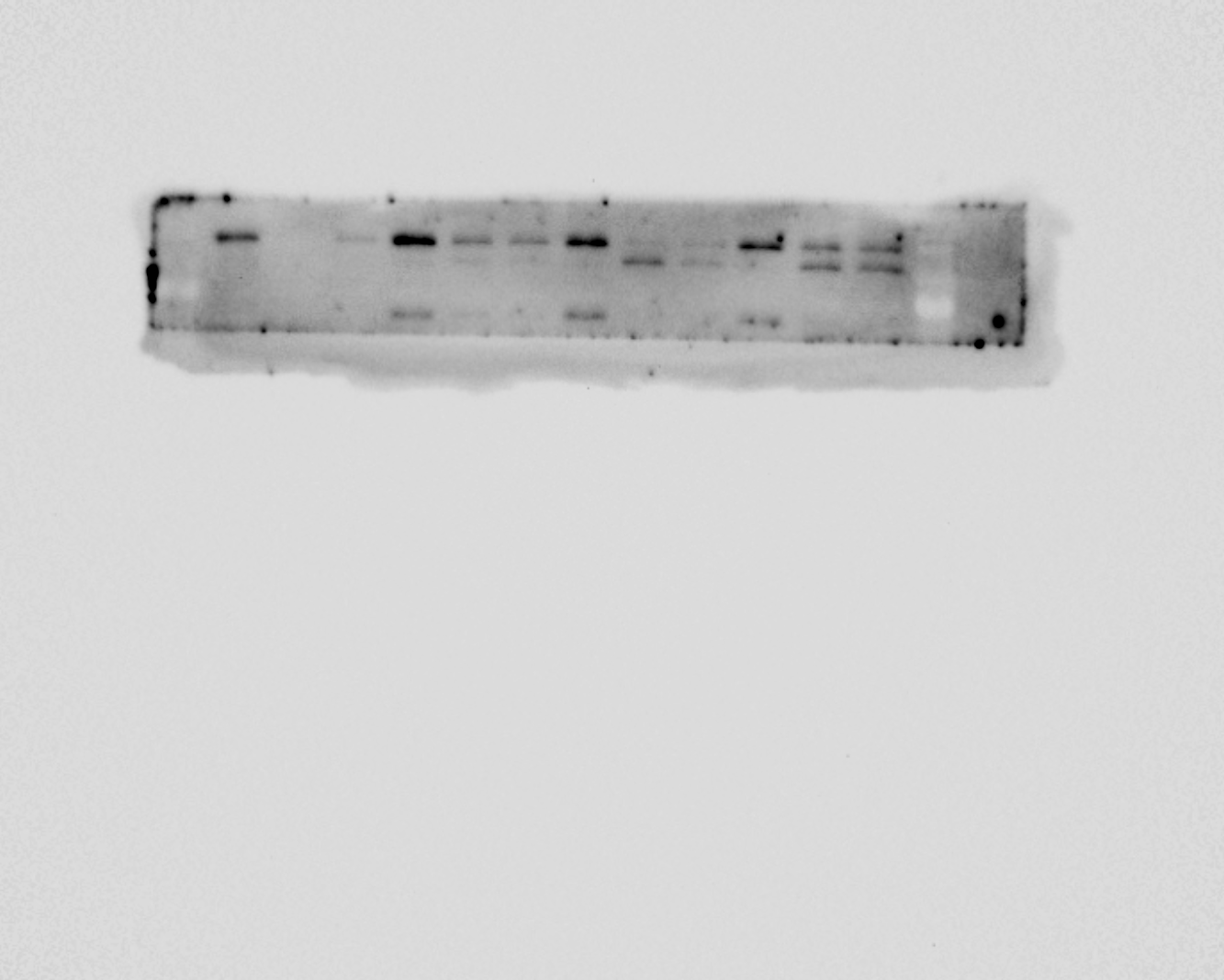

Supplement: Supplementary file 10 [file DataSheet5.ZIP › parp0sea_2(Chemiluminescence).tif]

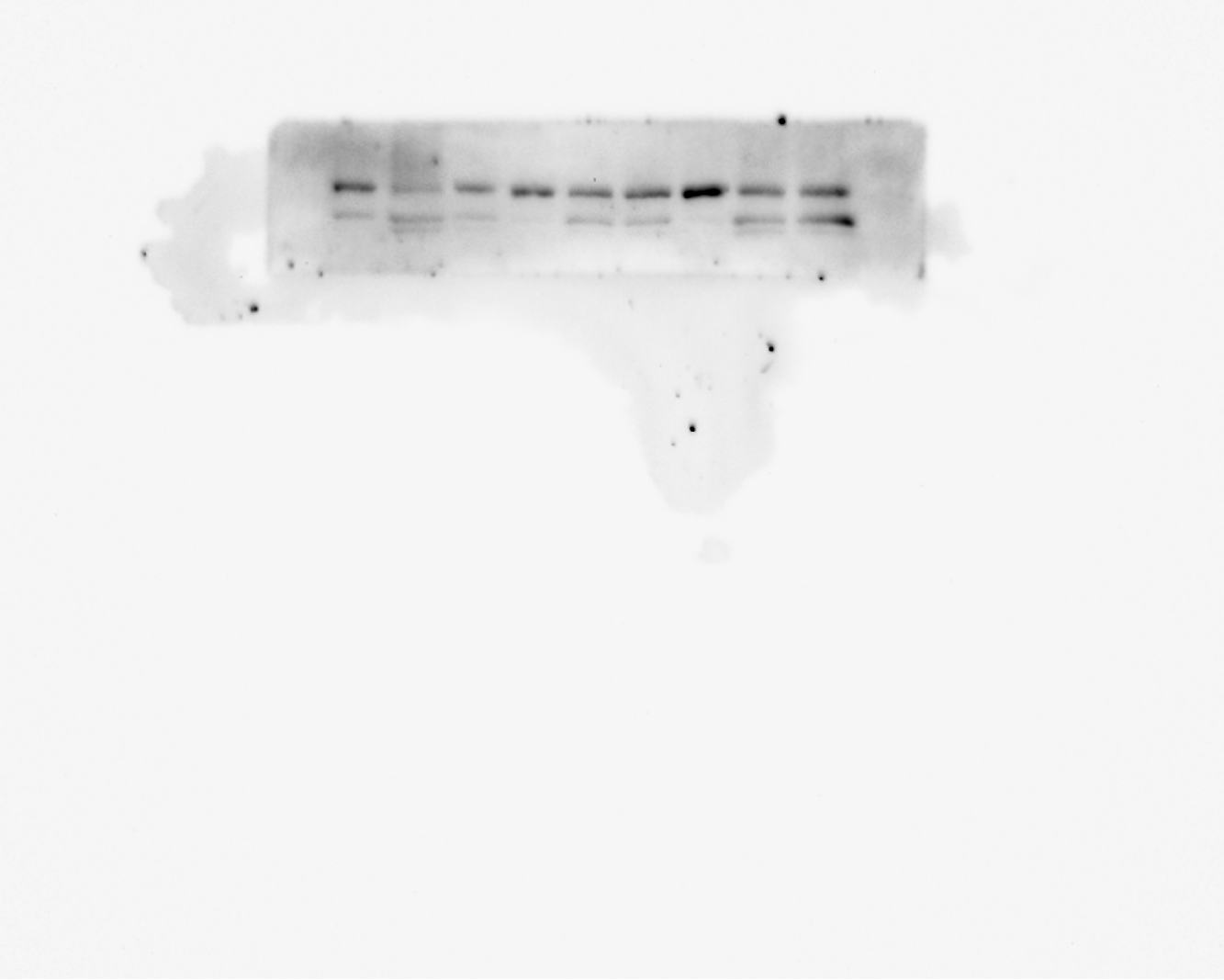

Supplement: Supplementary file 10 [file DataSheet5.ZIP › parp-3_3(Chemiluminescence).tif]

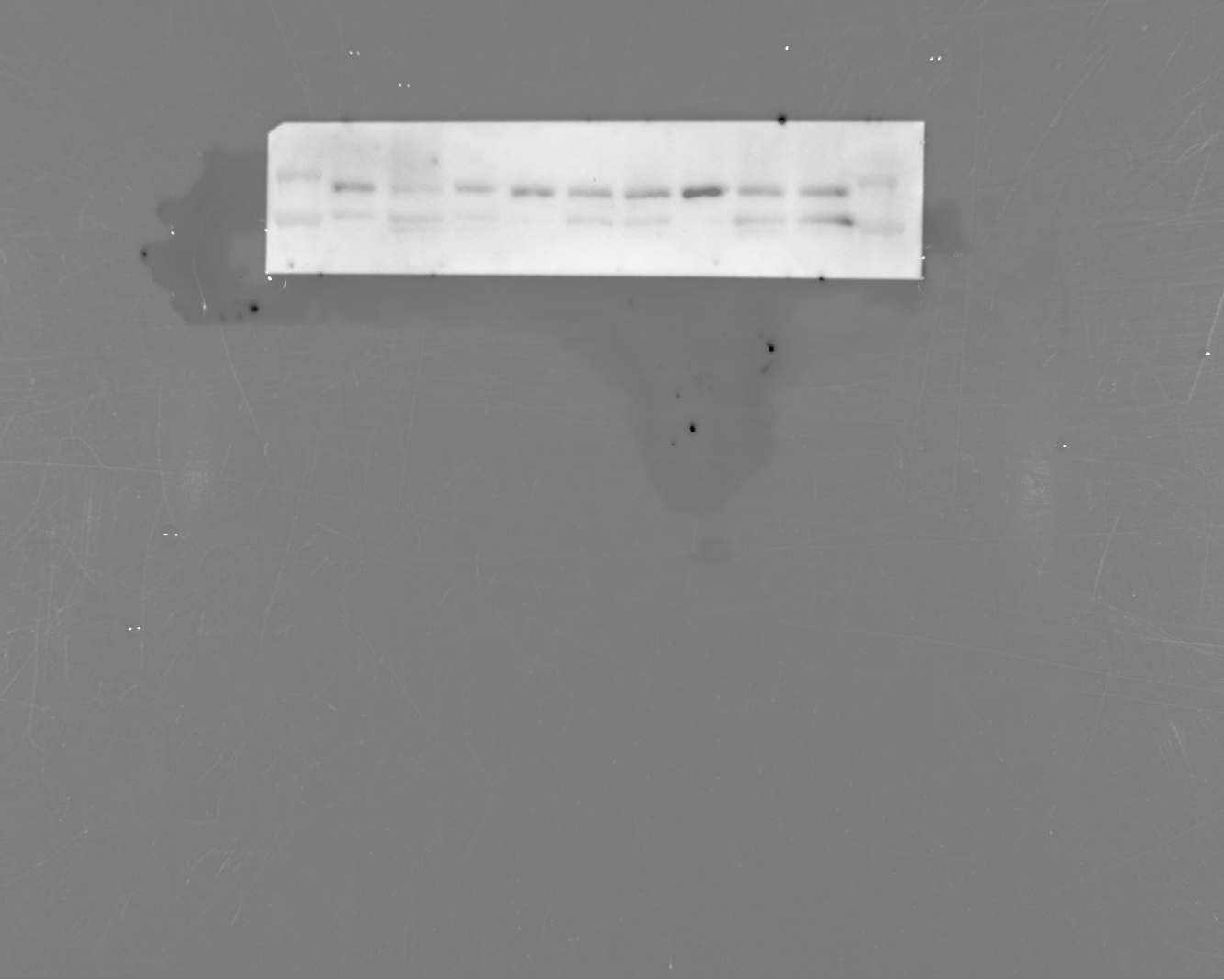

Supplement: Supplementary file 10 [file DataSheet5.ZIP › parp-3_3(Composite).tif]

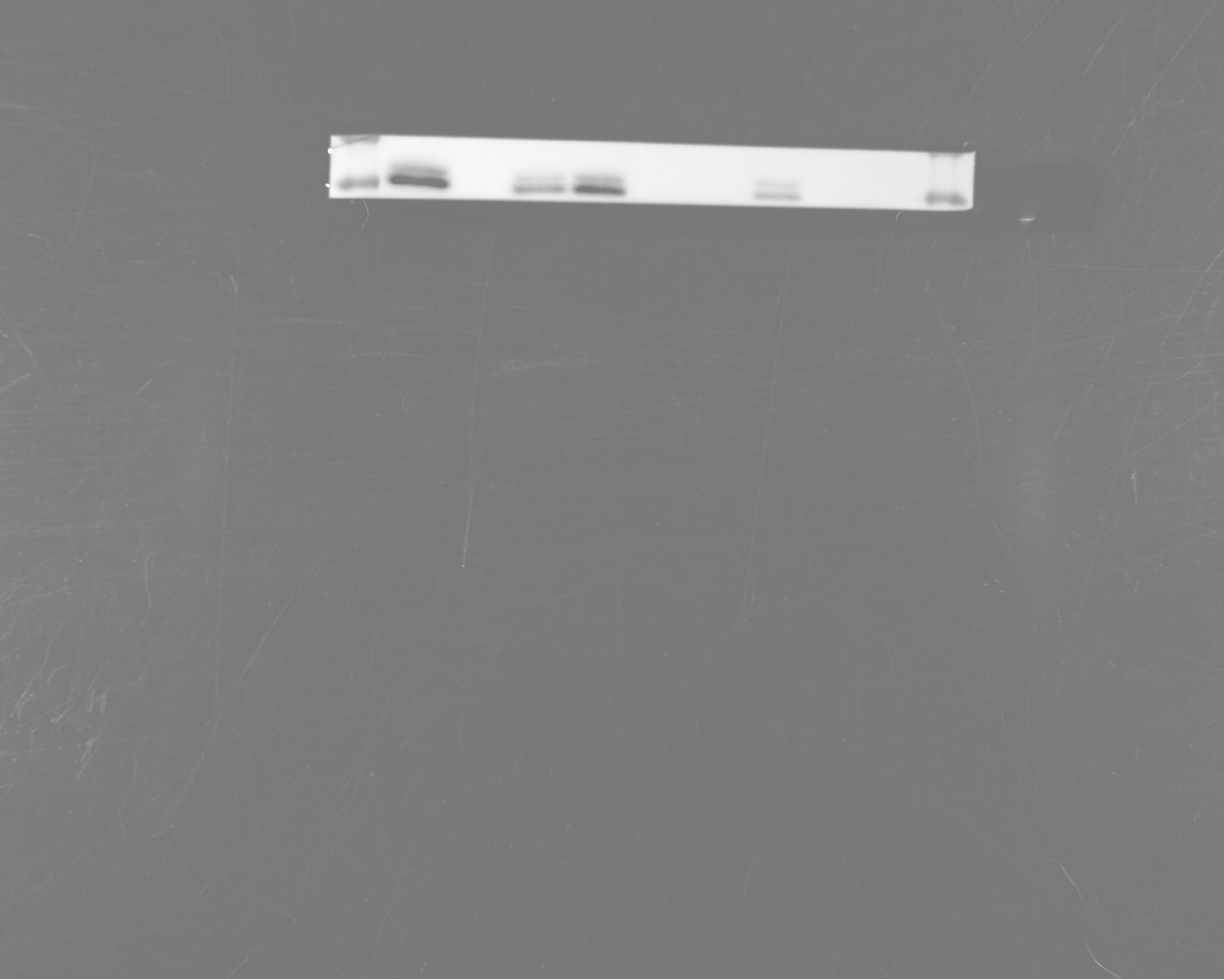

Supplement: Supplementary file 10 [file DataSheet5.ZIP › perk (1).tif]

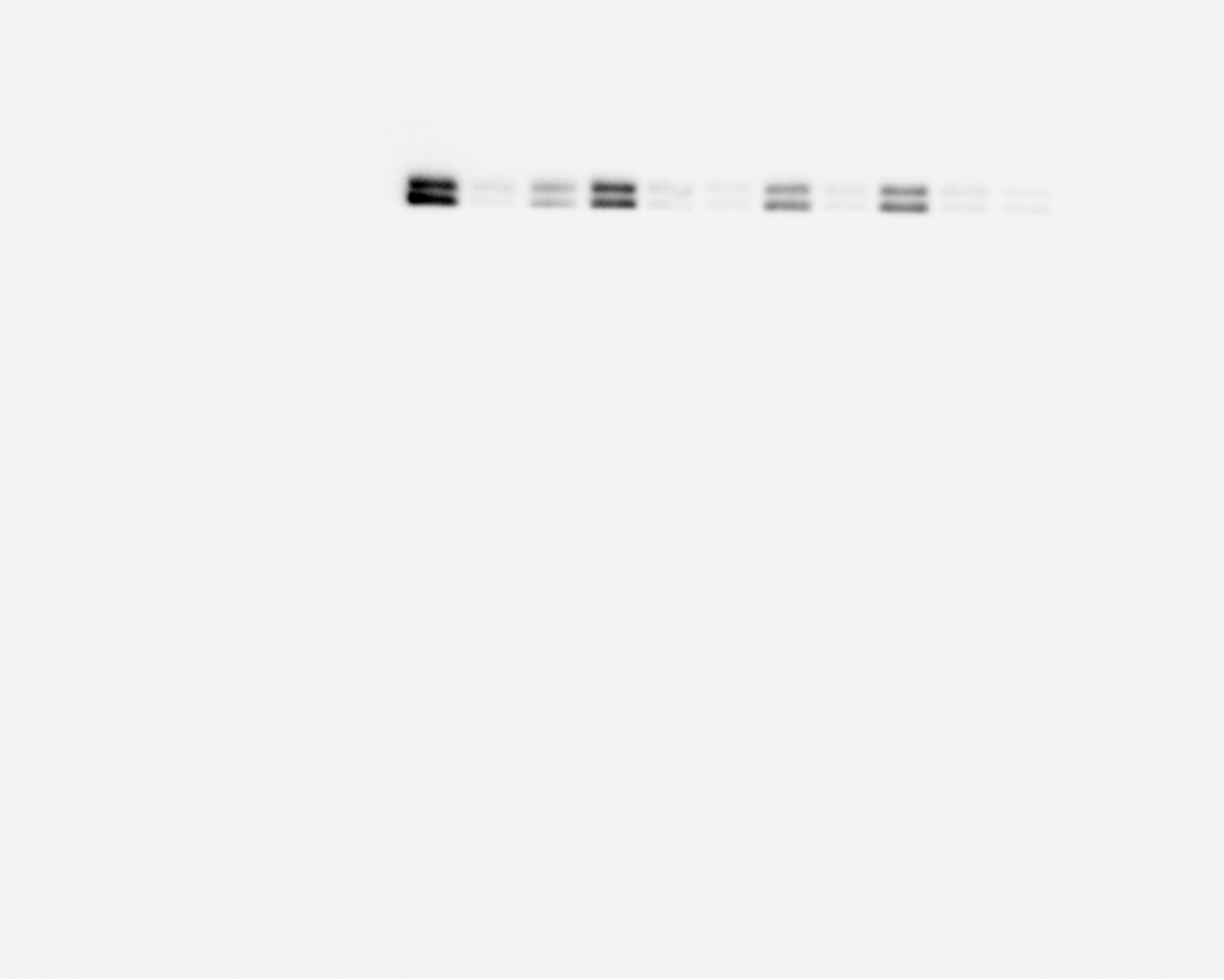

Supplement: Supplementary file 10 [file DataSheet5.ZIP › perk (2).tif]

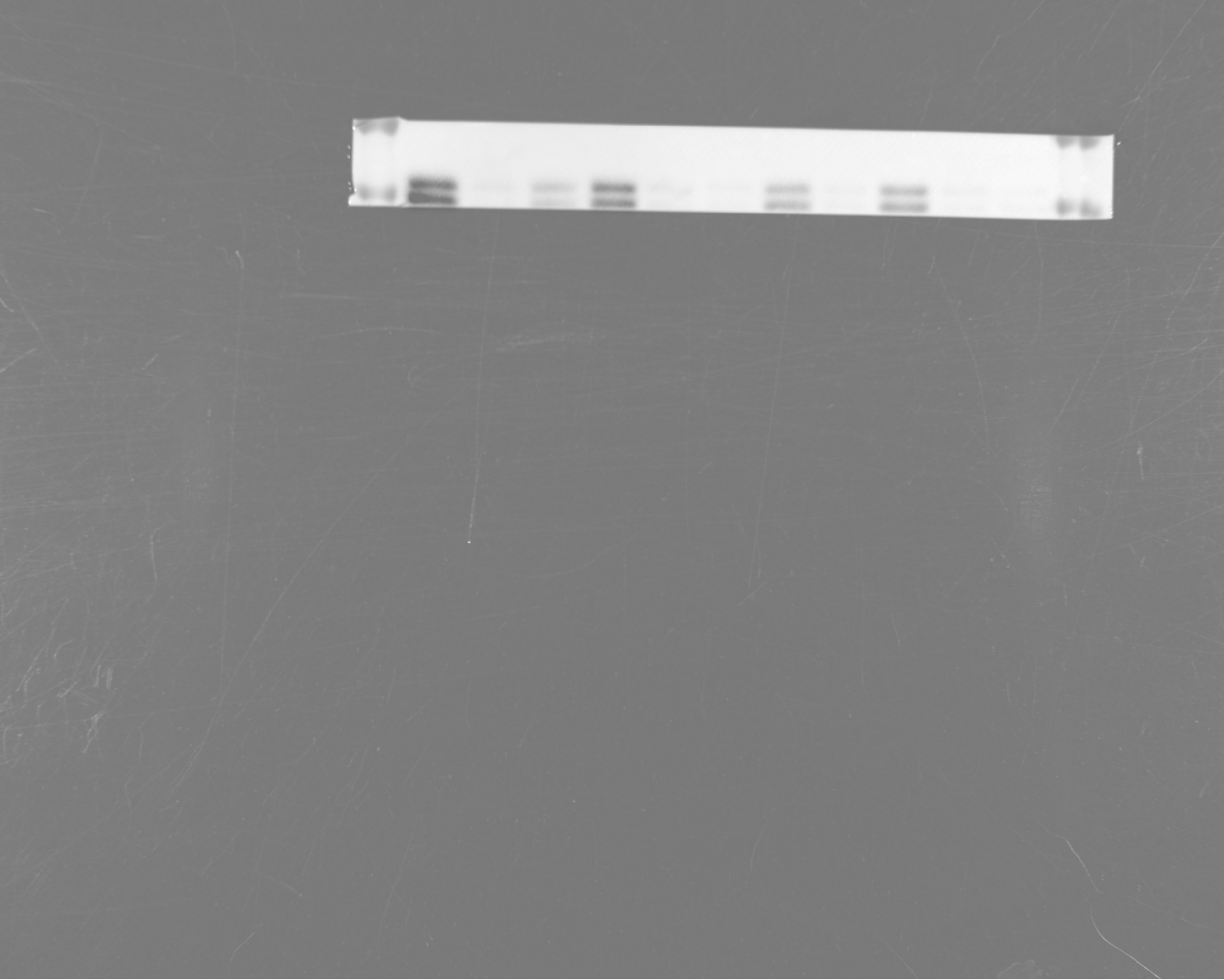

Supplement: Supplementary file 10 [file DataSheet5.ZIP › perk (4).tif]

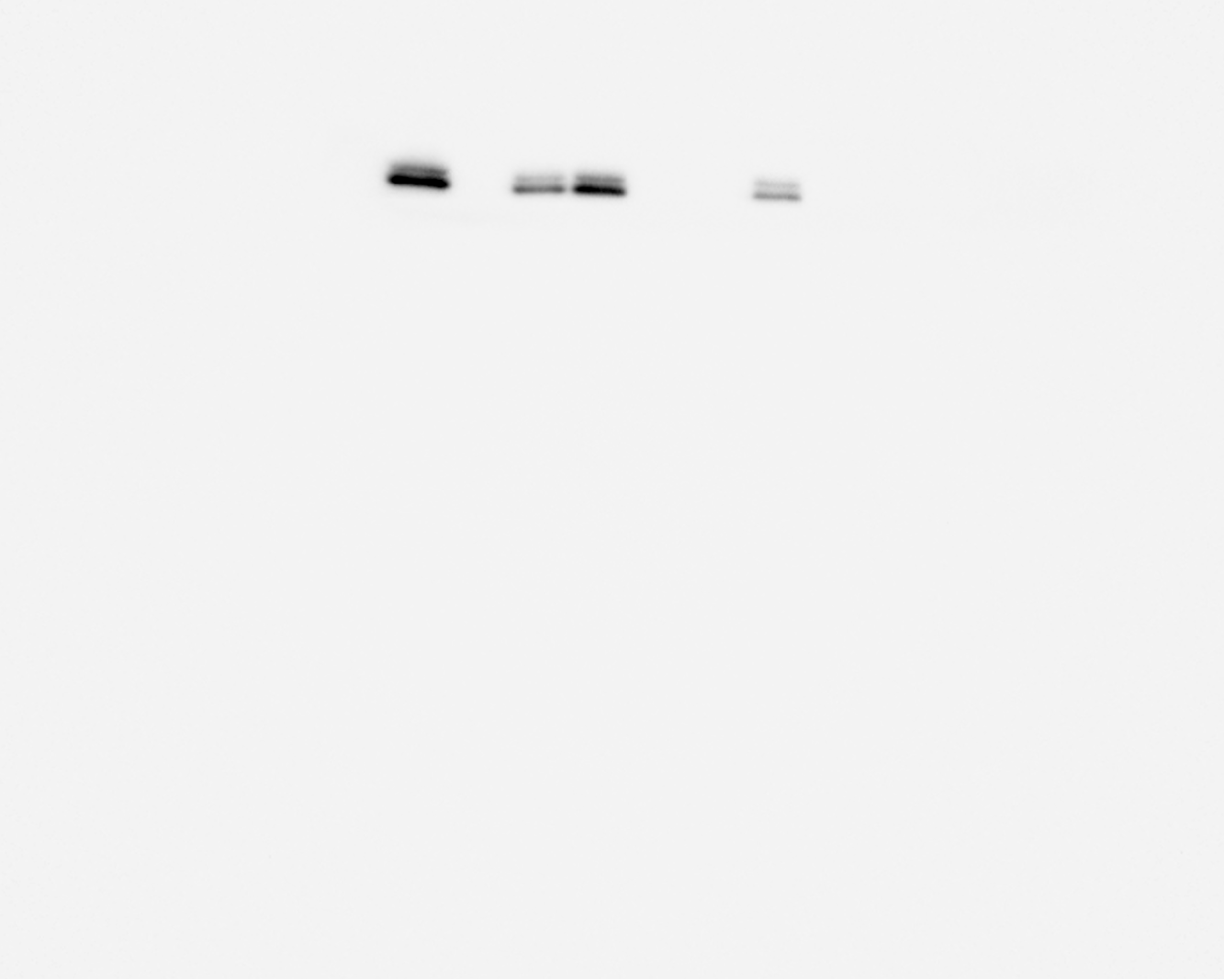

Supplement: Supplementary file 10 [file DataSheet5.ZIP › perk (5).tif]

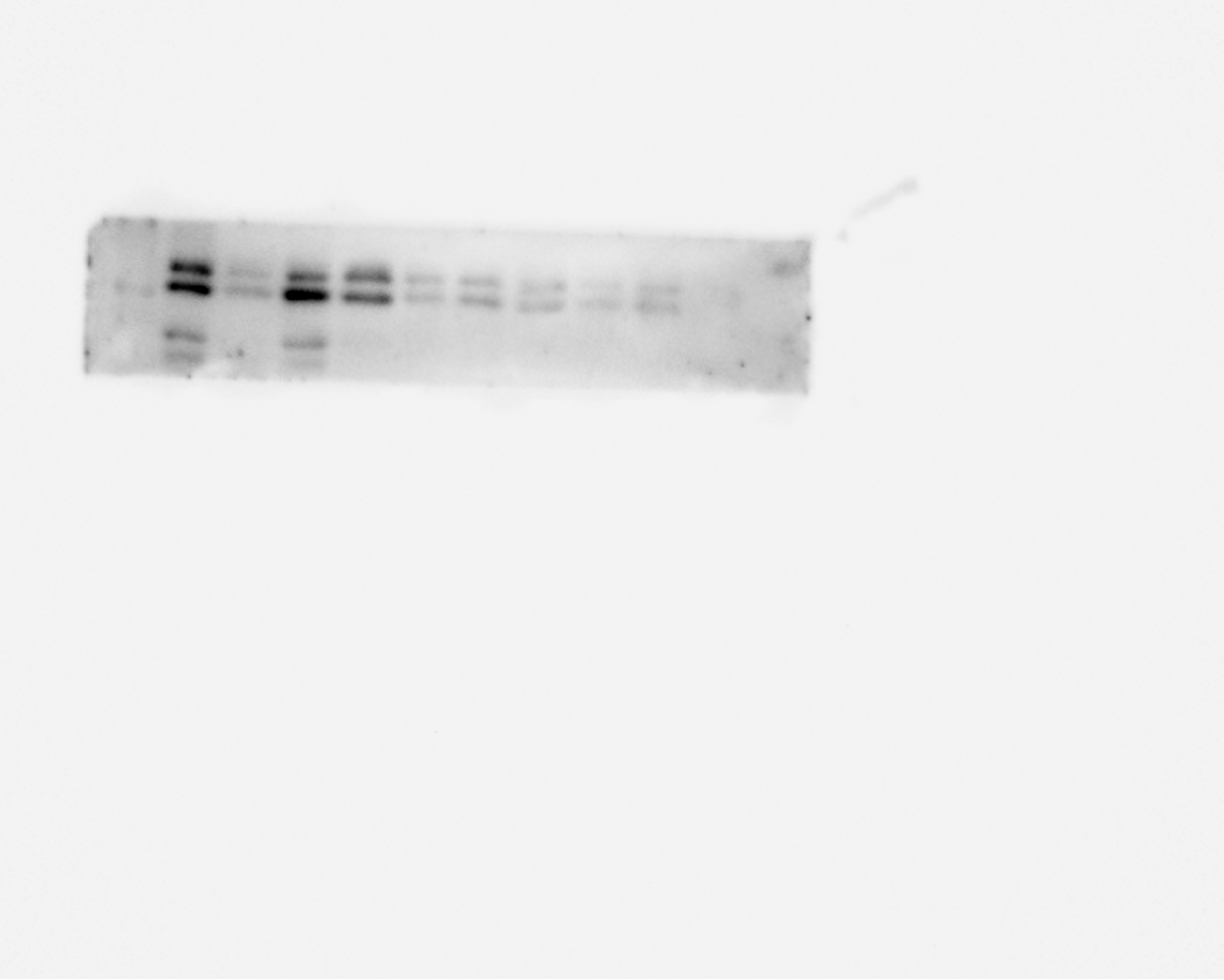

Supplement: Supplementary file 10 [file DataSheet5.ZIP › perk5_3(Chemiluminescence).tif]

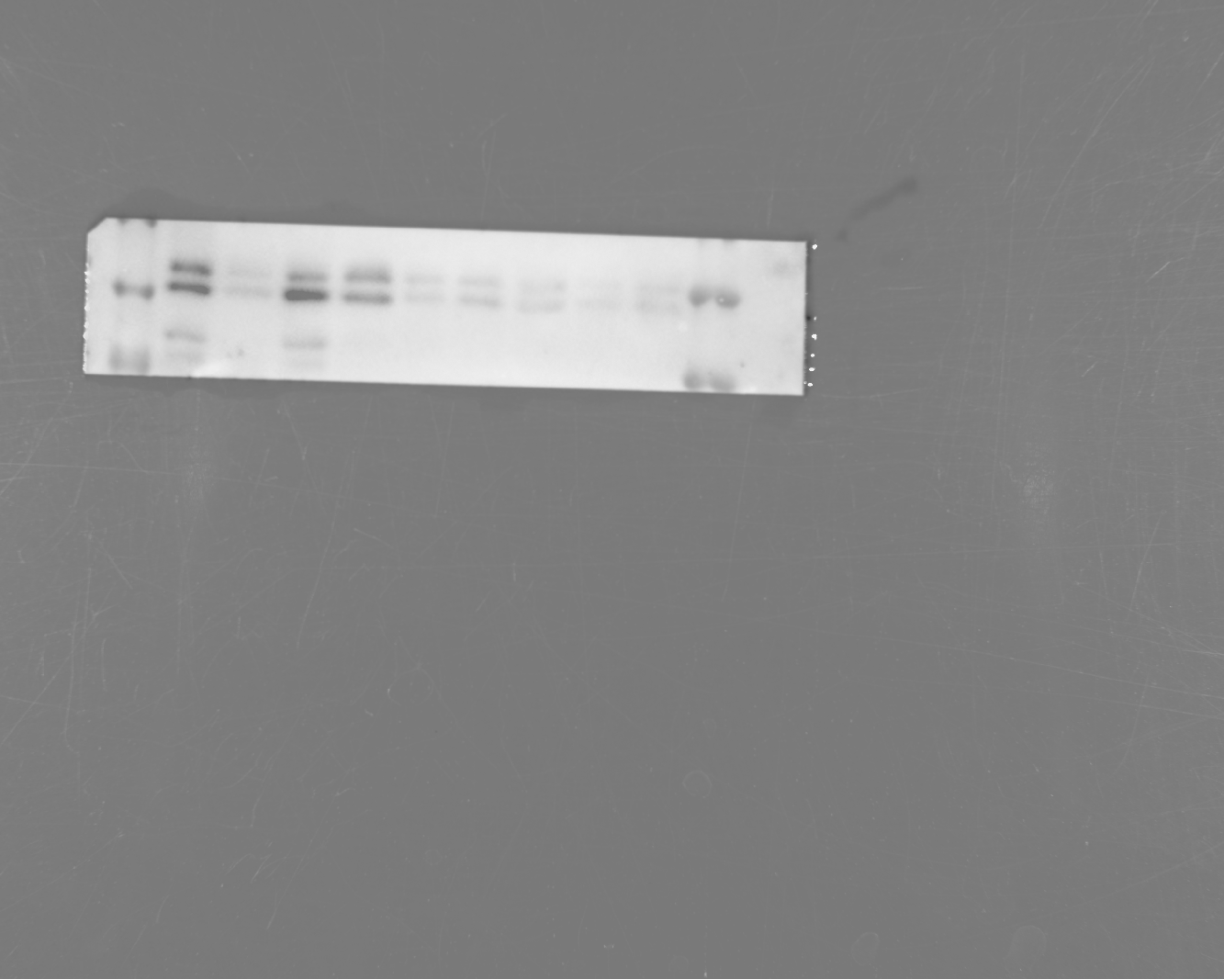

Supplement: Supplementary file 10 [file DataSheet5.ZIP › perk5_3(Composite).tif]

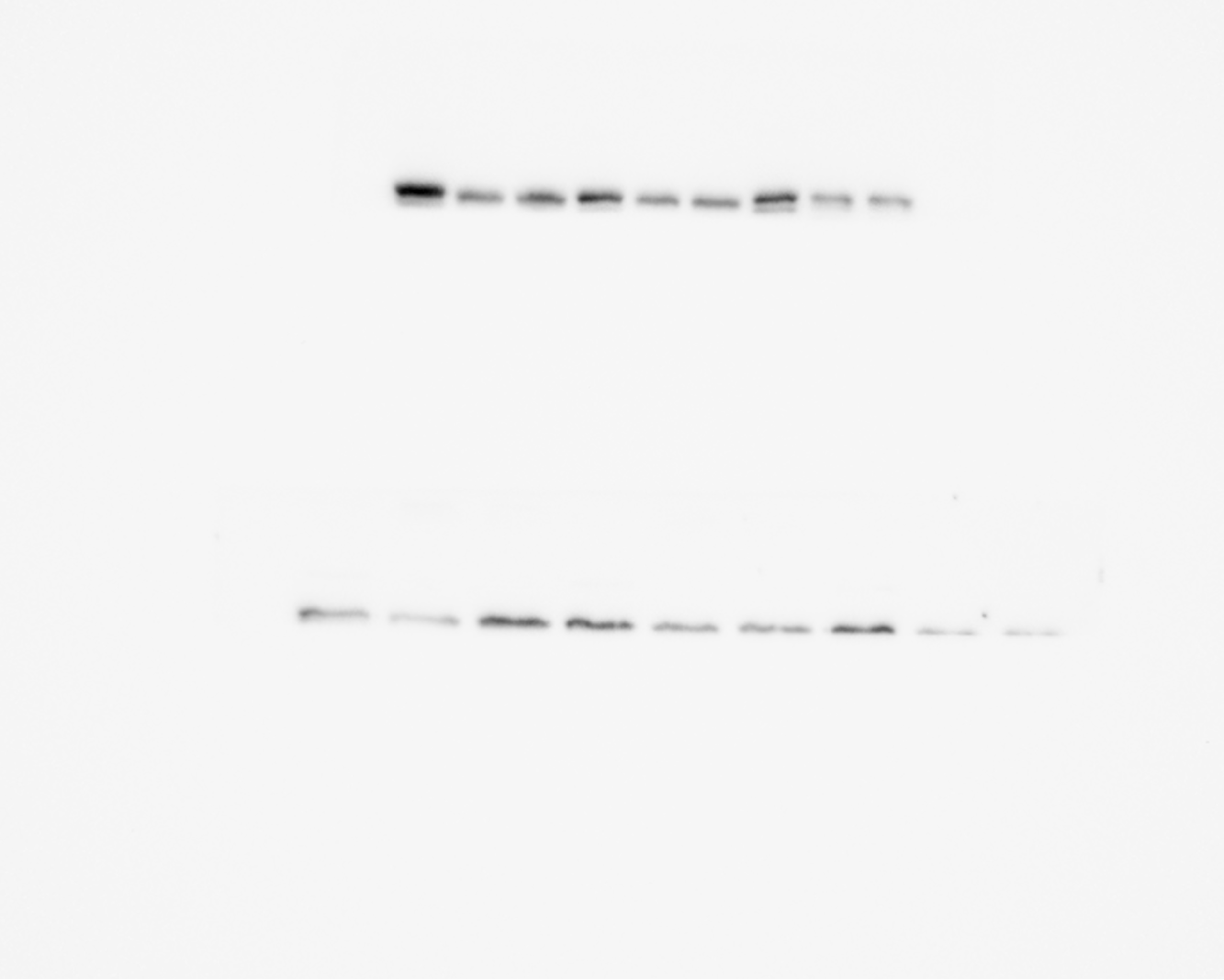

Supplement: Supplementary file 10 [file DataSheet5.ZIP › pst-13_4(Chemiluminescence).tif]

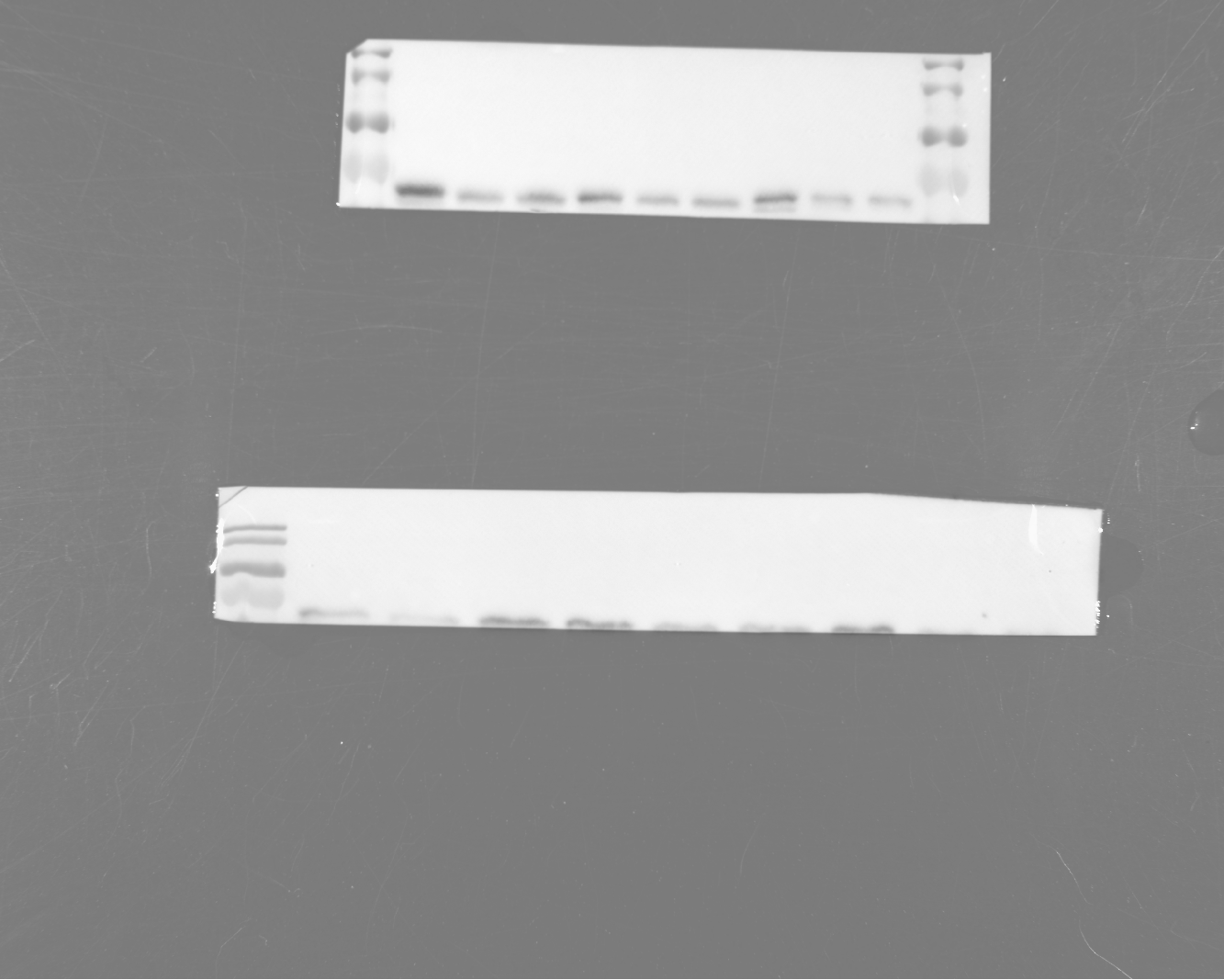

Supplement: Supplementary file 10 [file DataSheet5.ZIP › pst-13_4(Composite).tif]

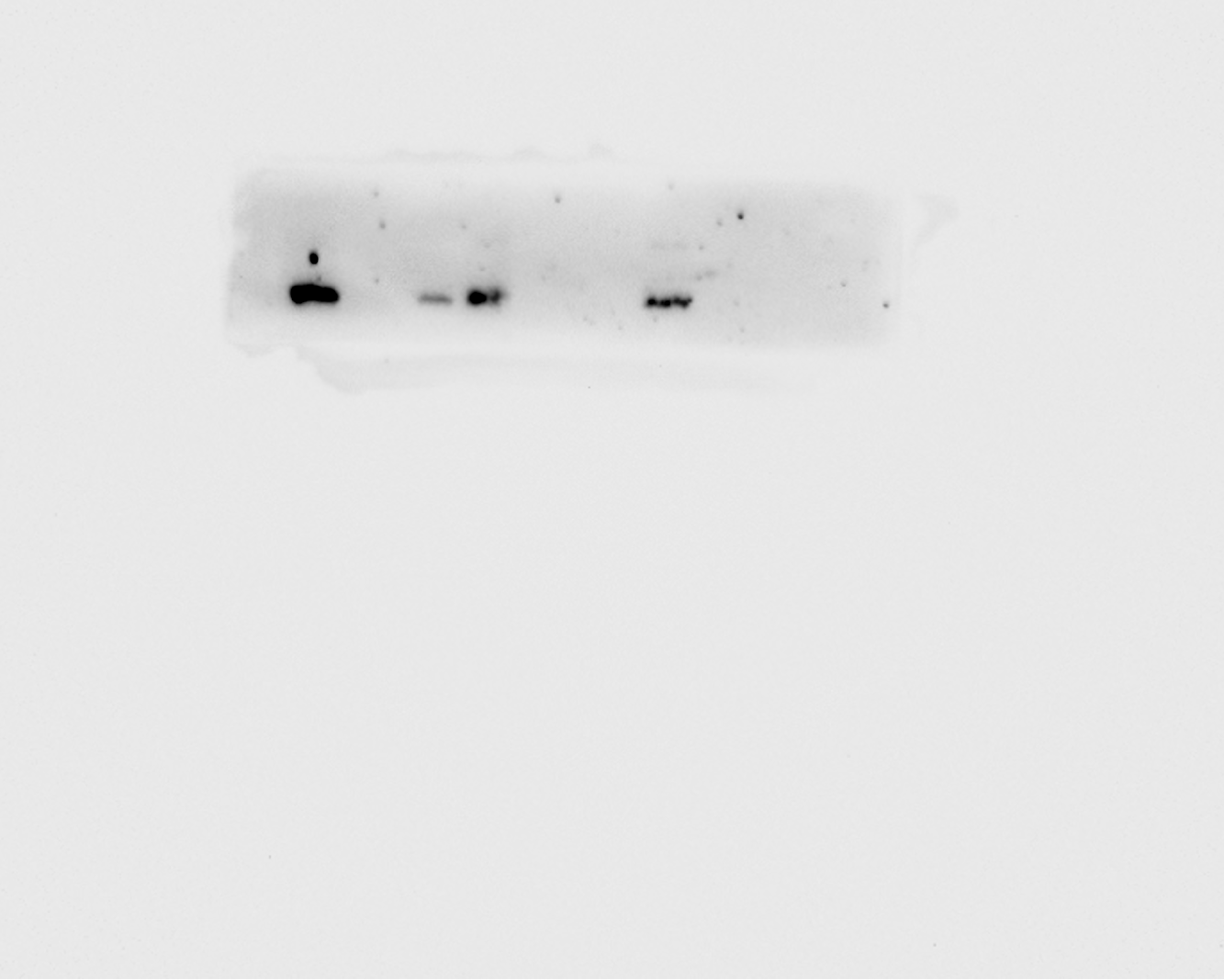

Supplement: Supplementary file 10 [file DataSheet5.ZIP › pst-16_3(Chemiluminescence).tif]

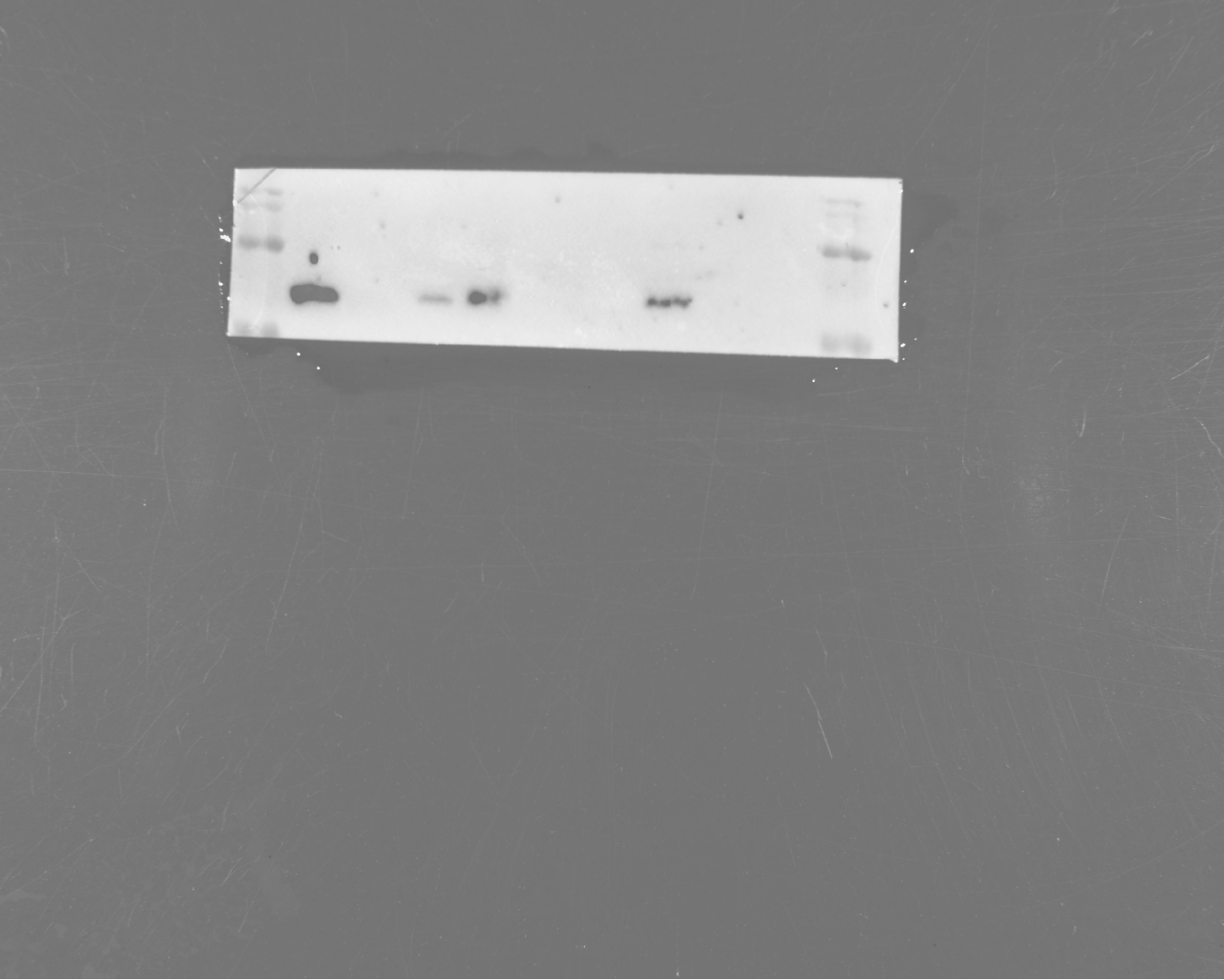

Supplement: Supplementary file 10 [file DataSheet5.ZIP › pst-16_3(Composite).tif]

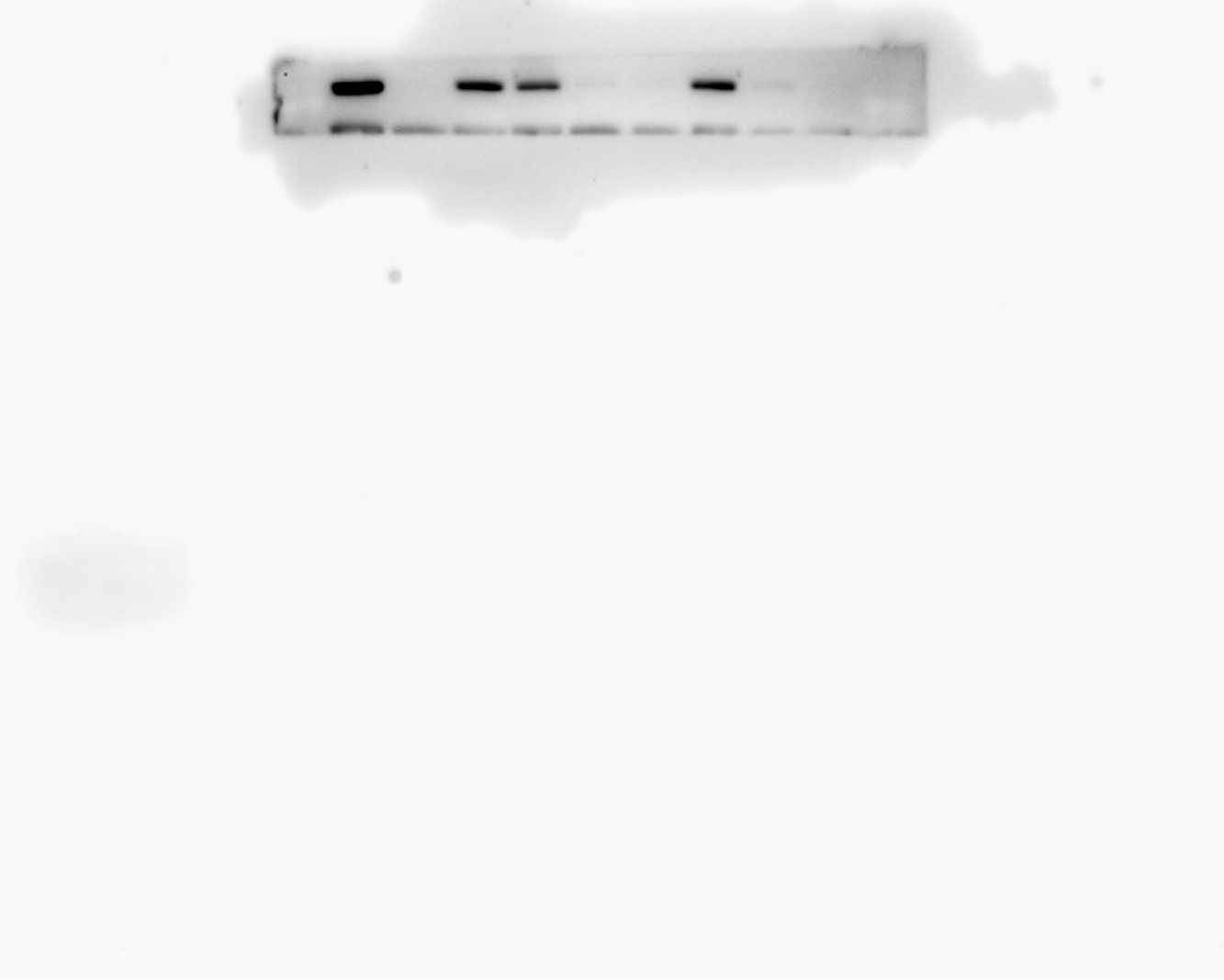

Supplement: Supplementary file 10 [file DataSheet5.ZIP › pst-17_3(Chemiluminescence).tif]

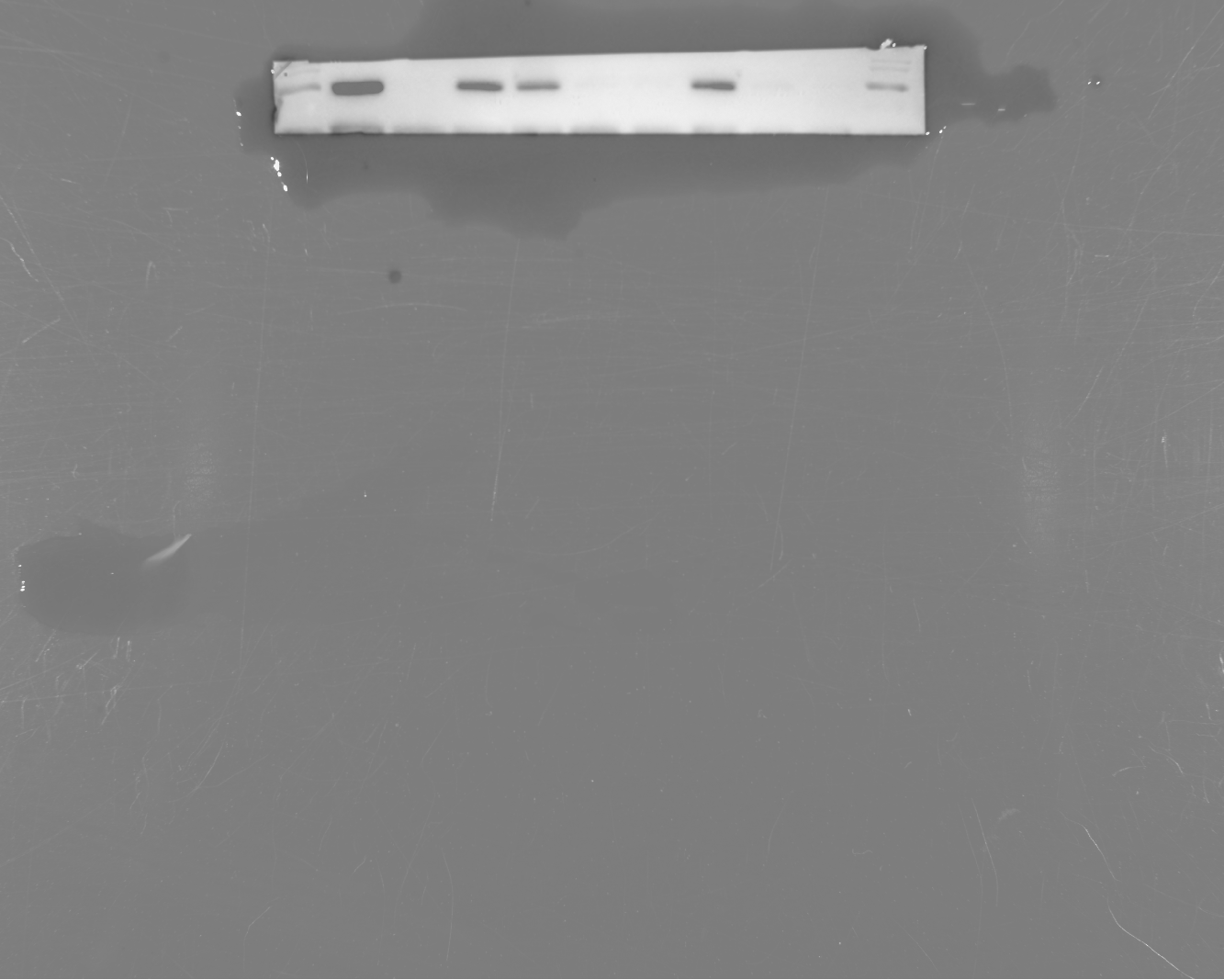

Supplement: Supplementary file 10 [file DataSheet5.ZIP › pst-17_3(Composite).tif]

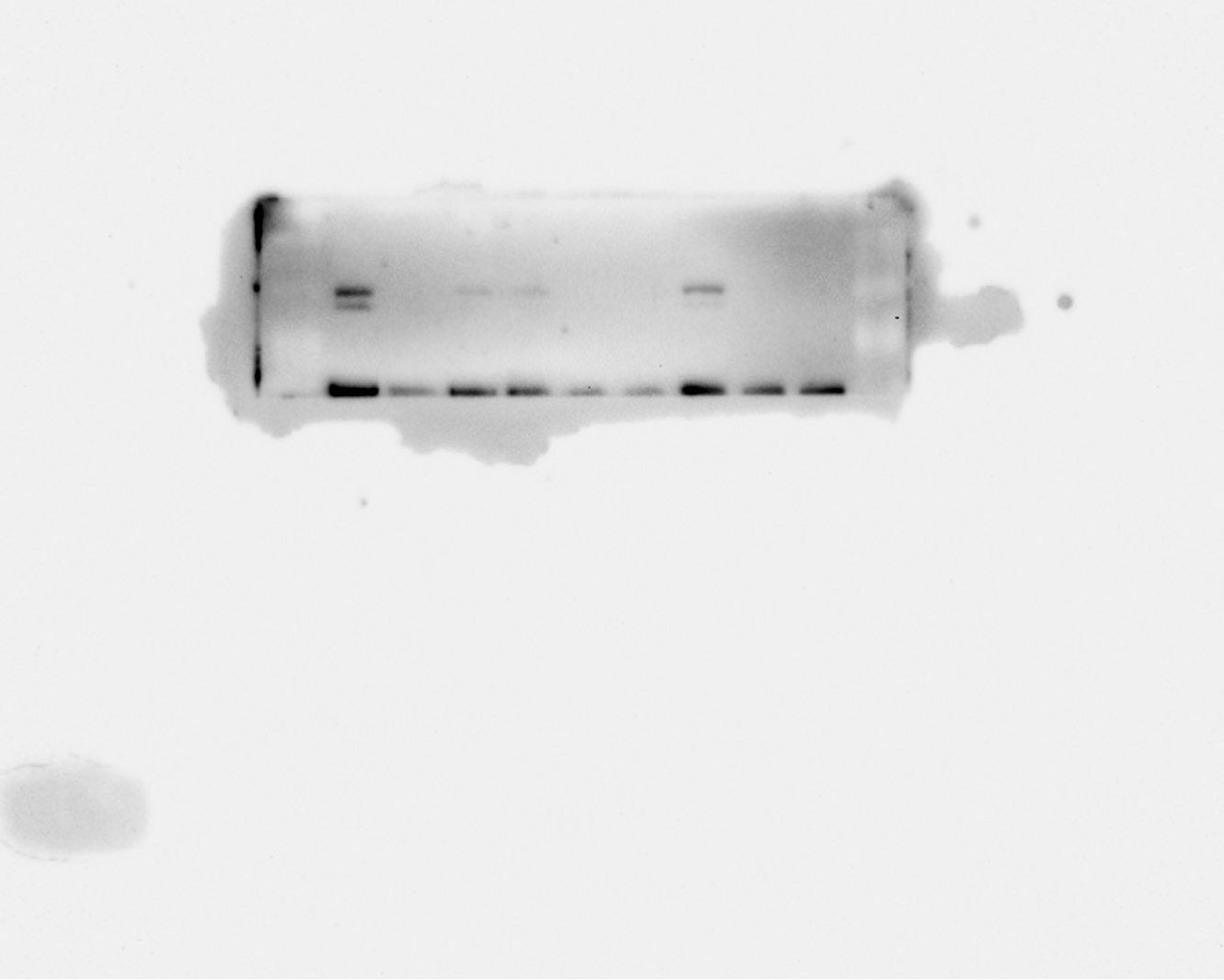

Supplement: Supplementary file 10 [file DataSheet5.ZIP › pst-17-2_3(Chemiluminescence).tif]

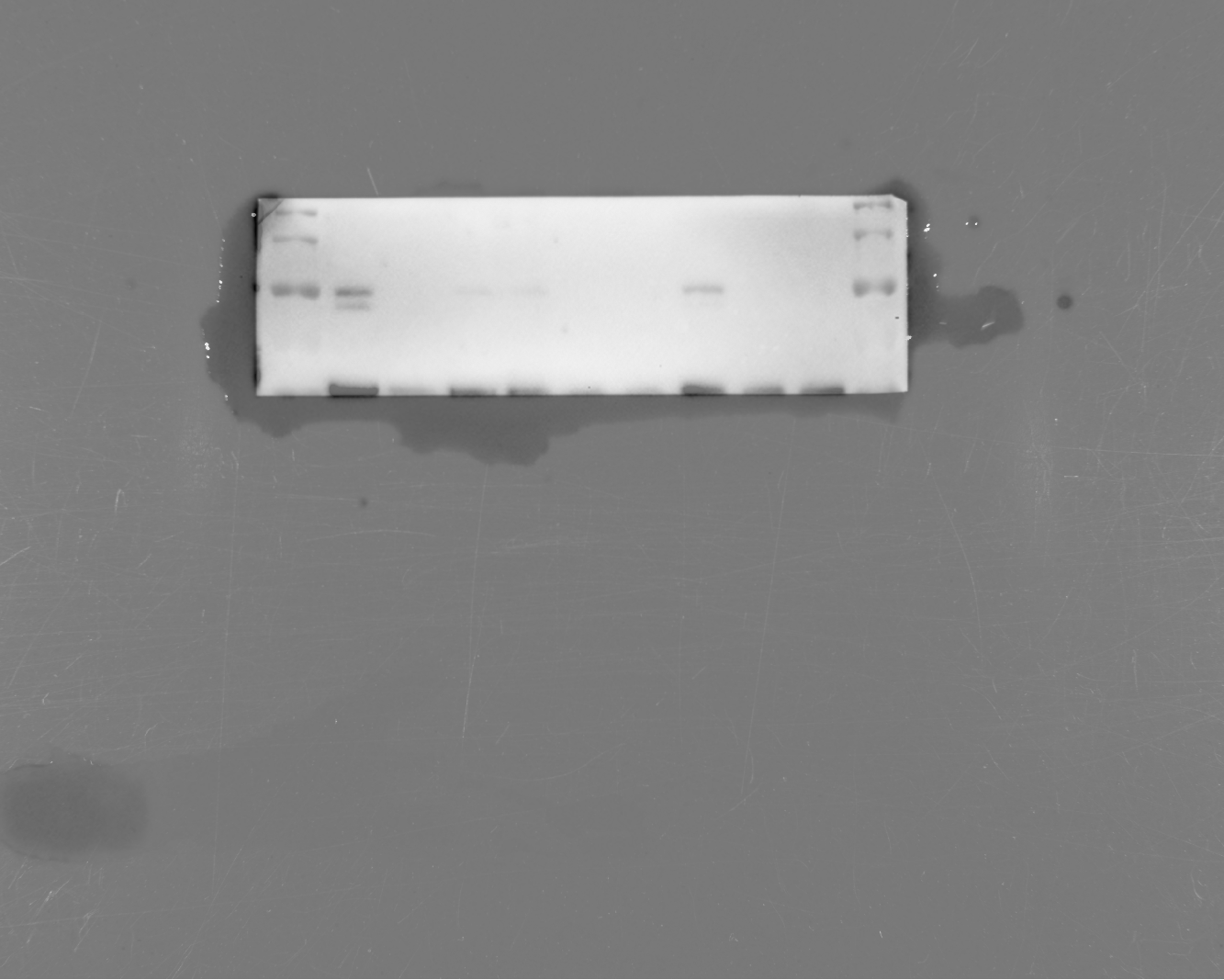

Supplement: Supplementary file 10 [file DataSheet5.ZIP › pst-17-2_3(Composite).tif]

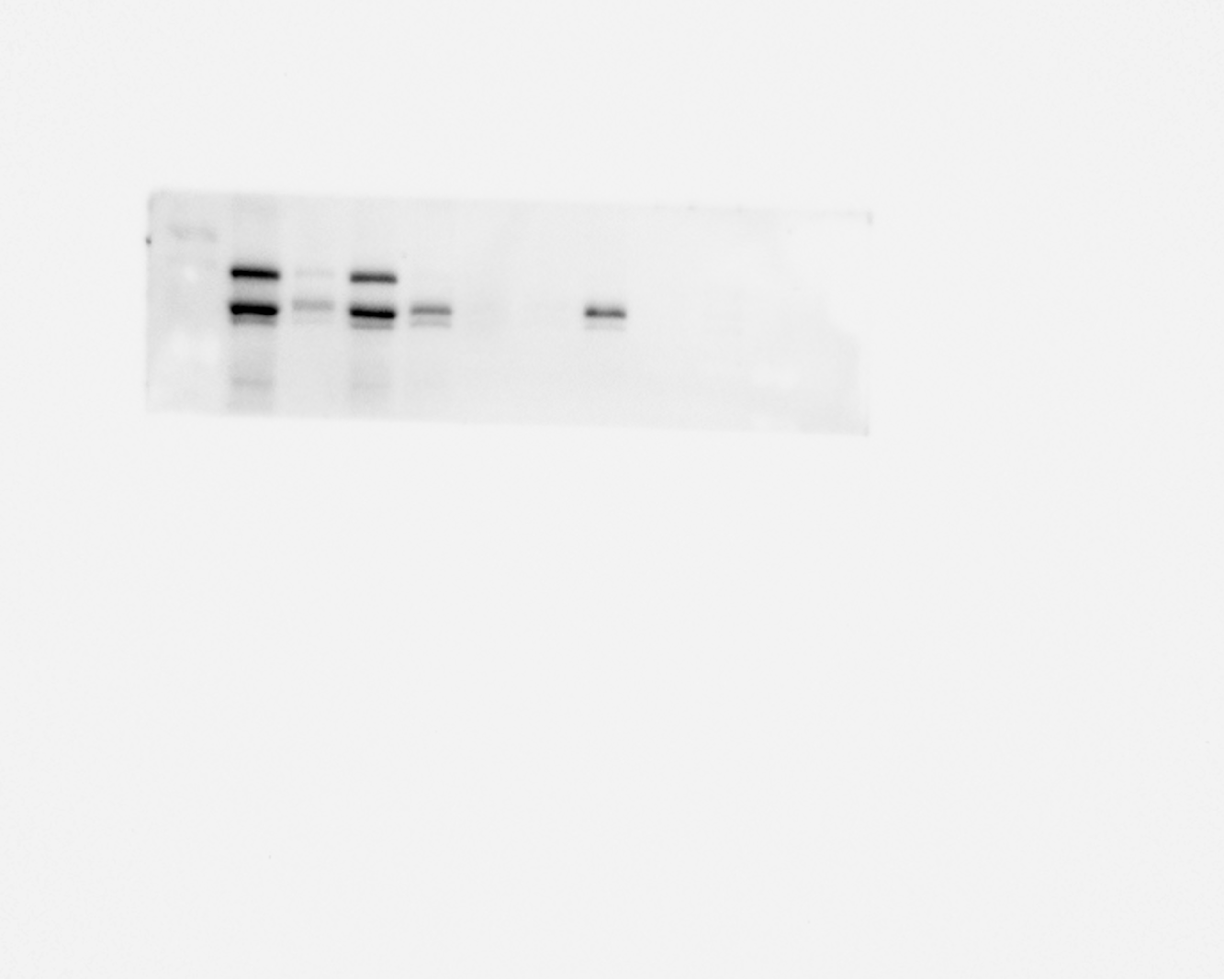

Supplement: Supplementary file 10 [file DataSheet5.ZIP › pst-5_3(Chemiluminescence).tif]

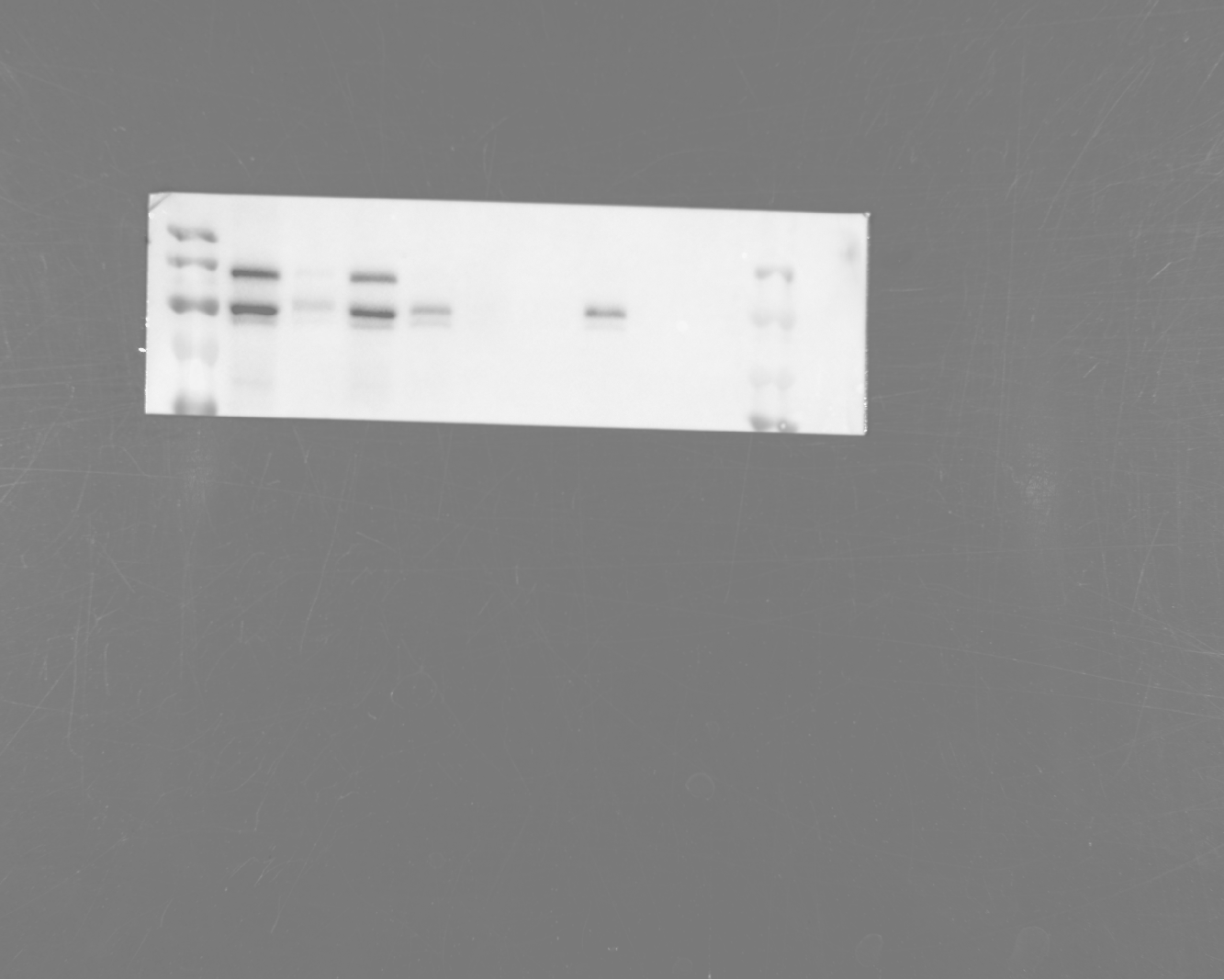

Supplement: Supplementary file 10 [file DataSheet5.ZIP › pst-5_3(Composite).tif]

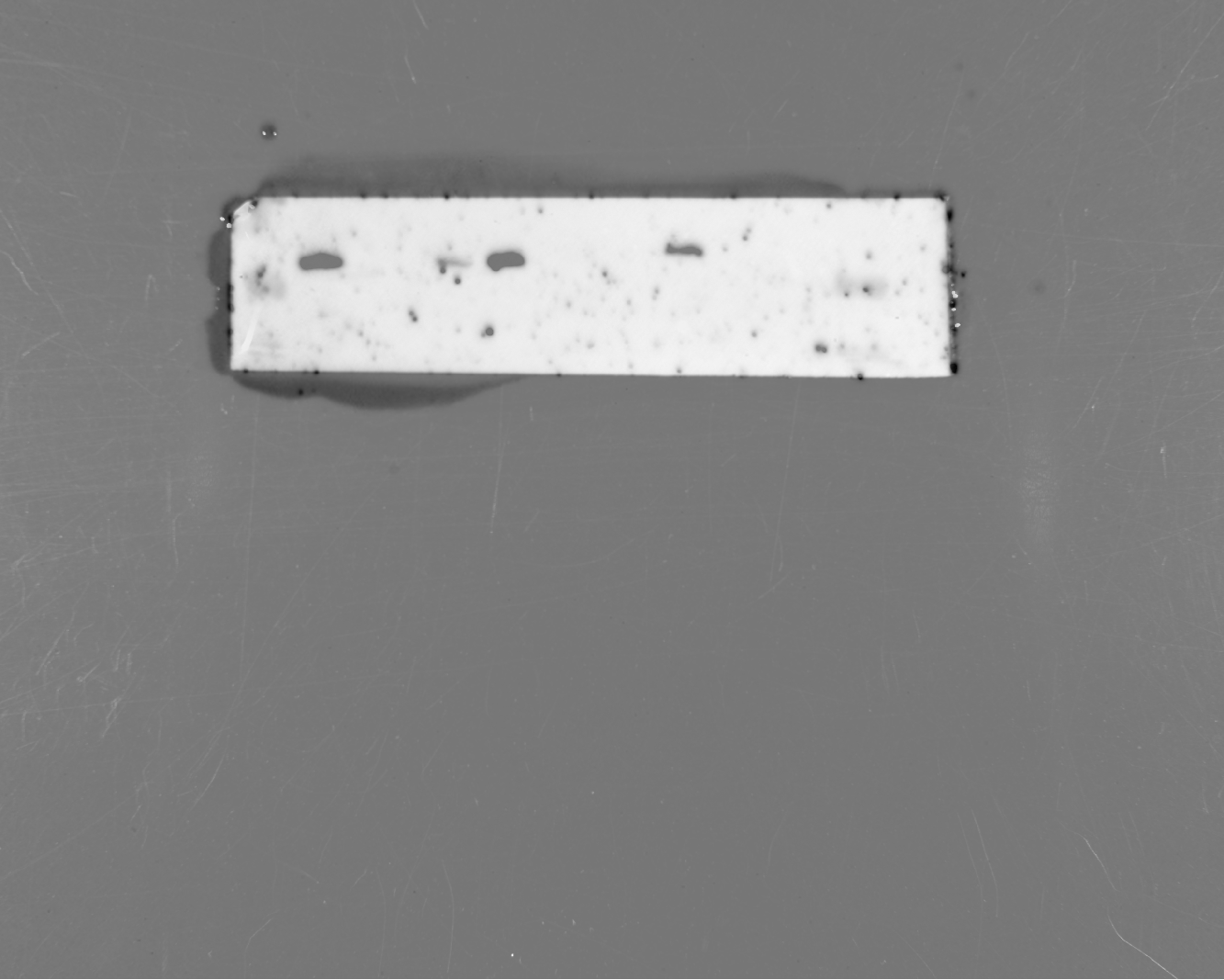

Supplement: Supplementary file 10 [file DataSheet5.ZIP › pst-6 (2).tif]

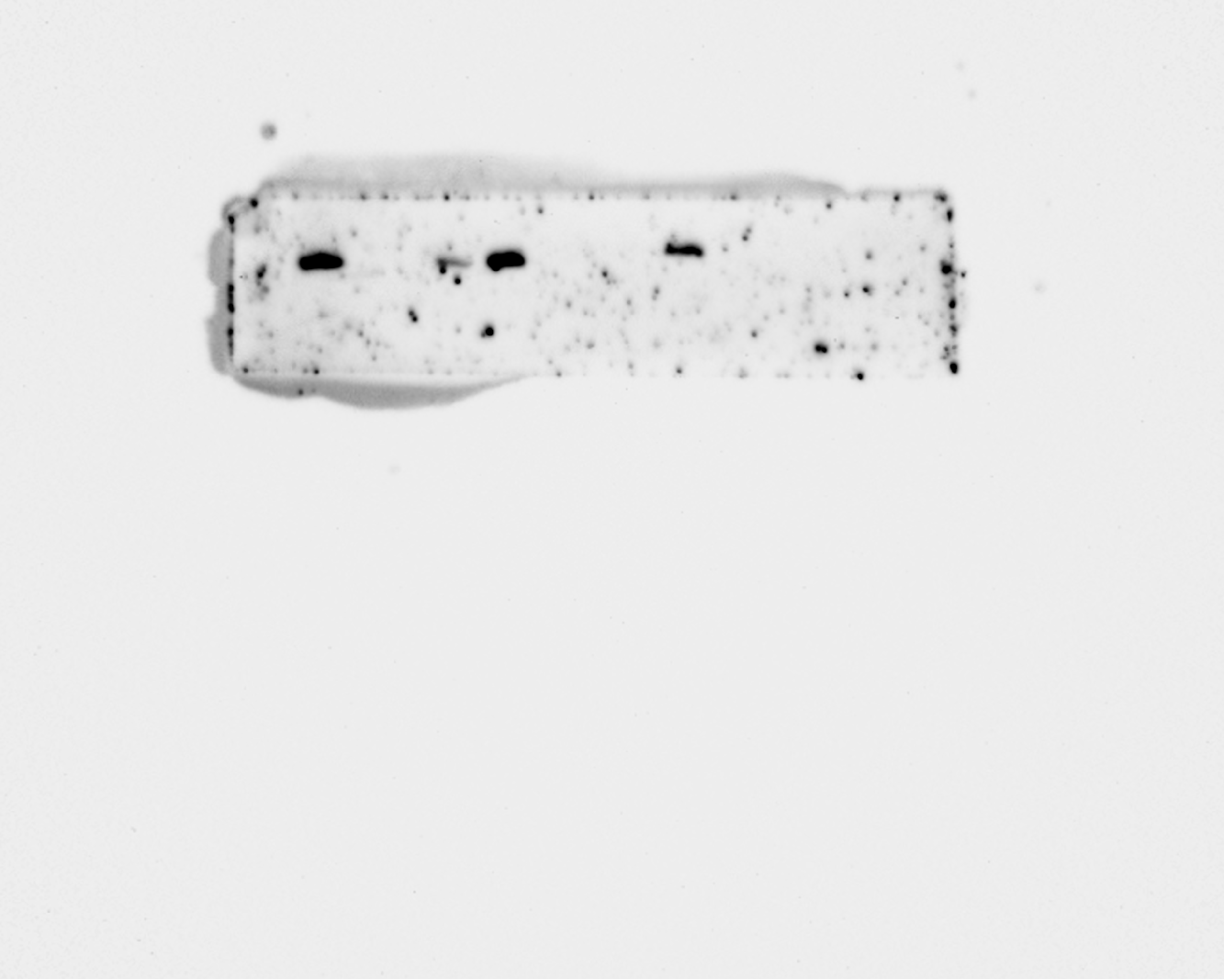

Supplement: Supplementary file 10 [file DataSheet5.ZIP › pst-6 (3).tif]

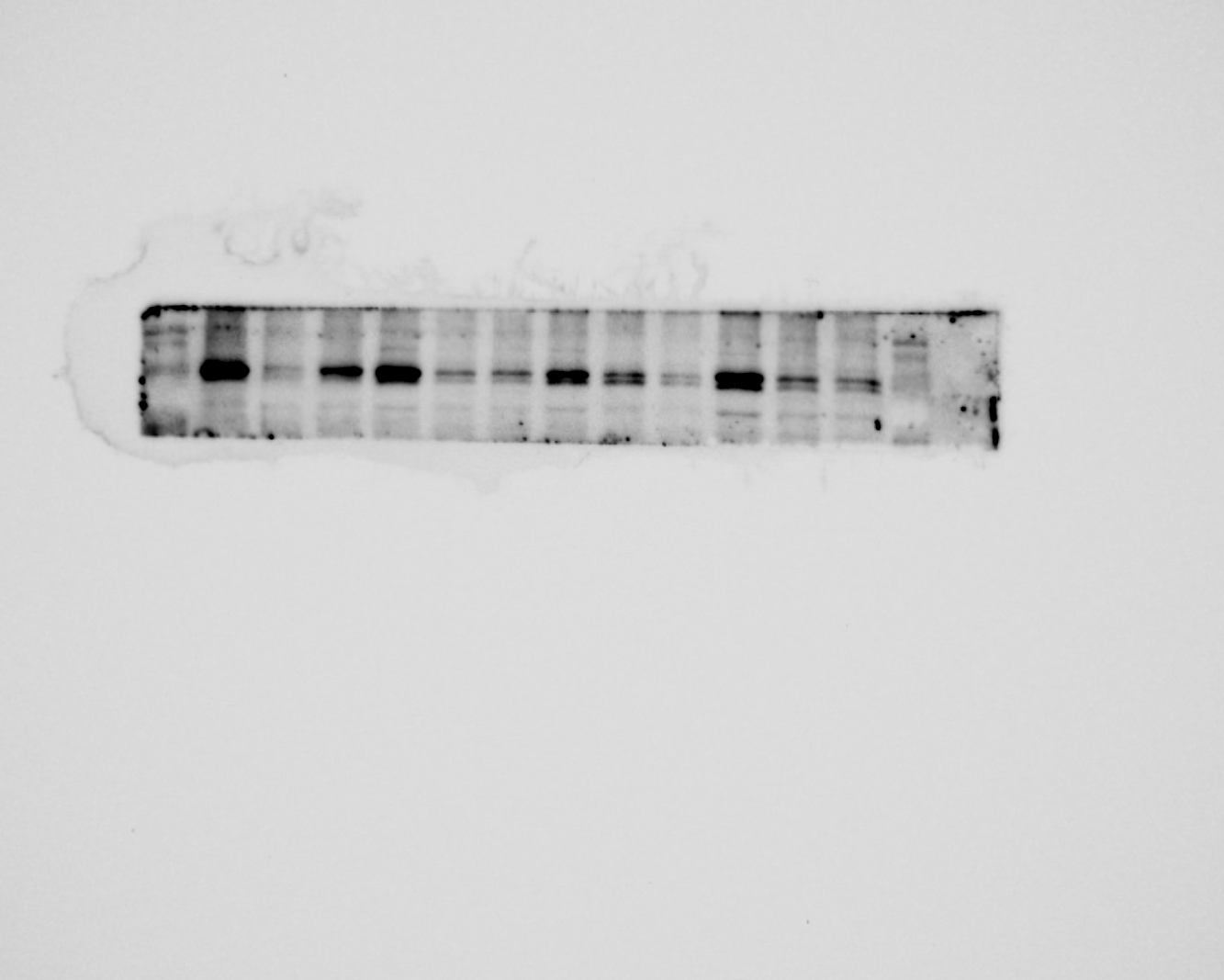

Supplement: Supplementary file 10 [file DataSheet5.ZIP › pstat3sea_1(Chemiluminescence).tif]

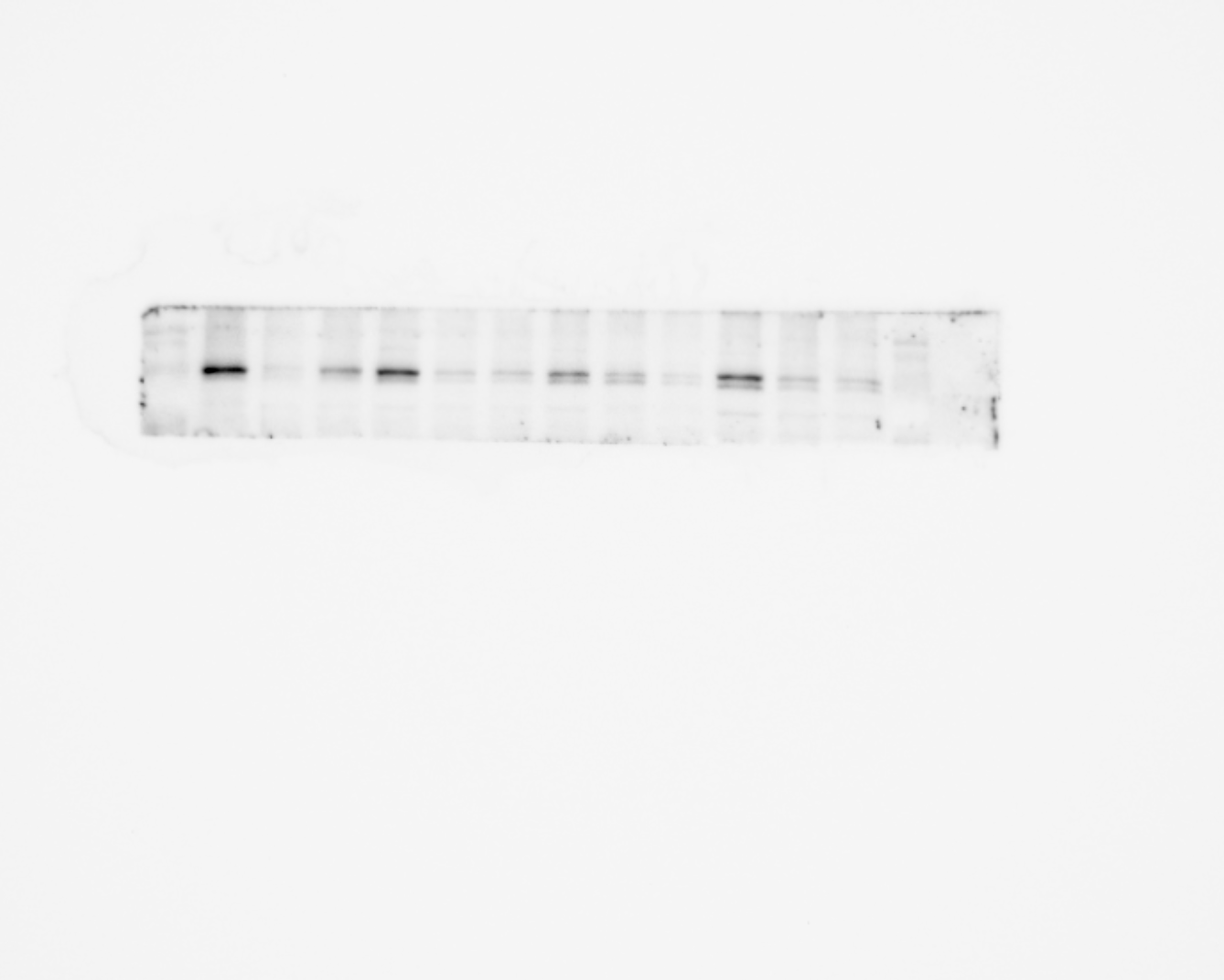

Supplement: Supplementary file 10 [file DataSheet5.ZIP › pstat3sea_1.tif]

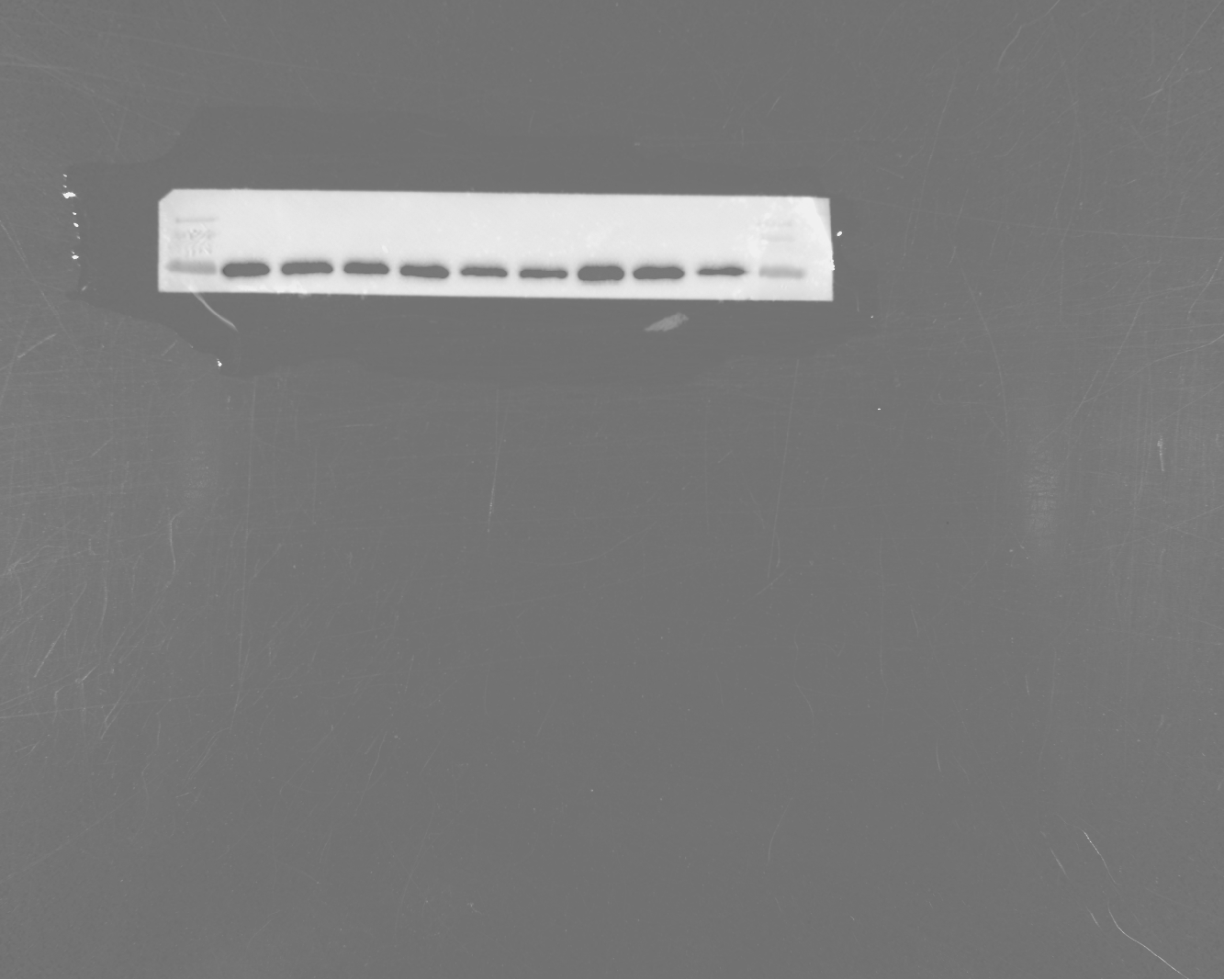

Supplement: Supplementary file 10 [file DataSheet5.ZIP › ST (1).tif]

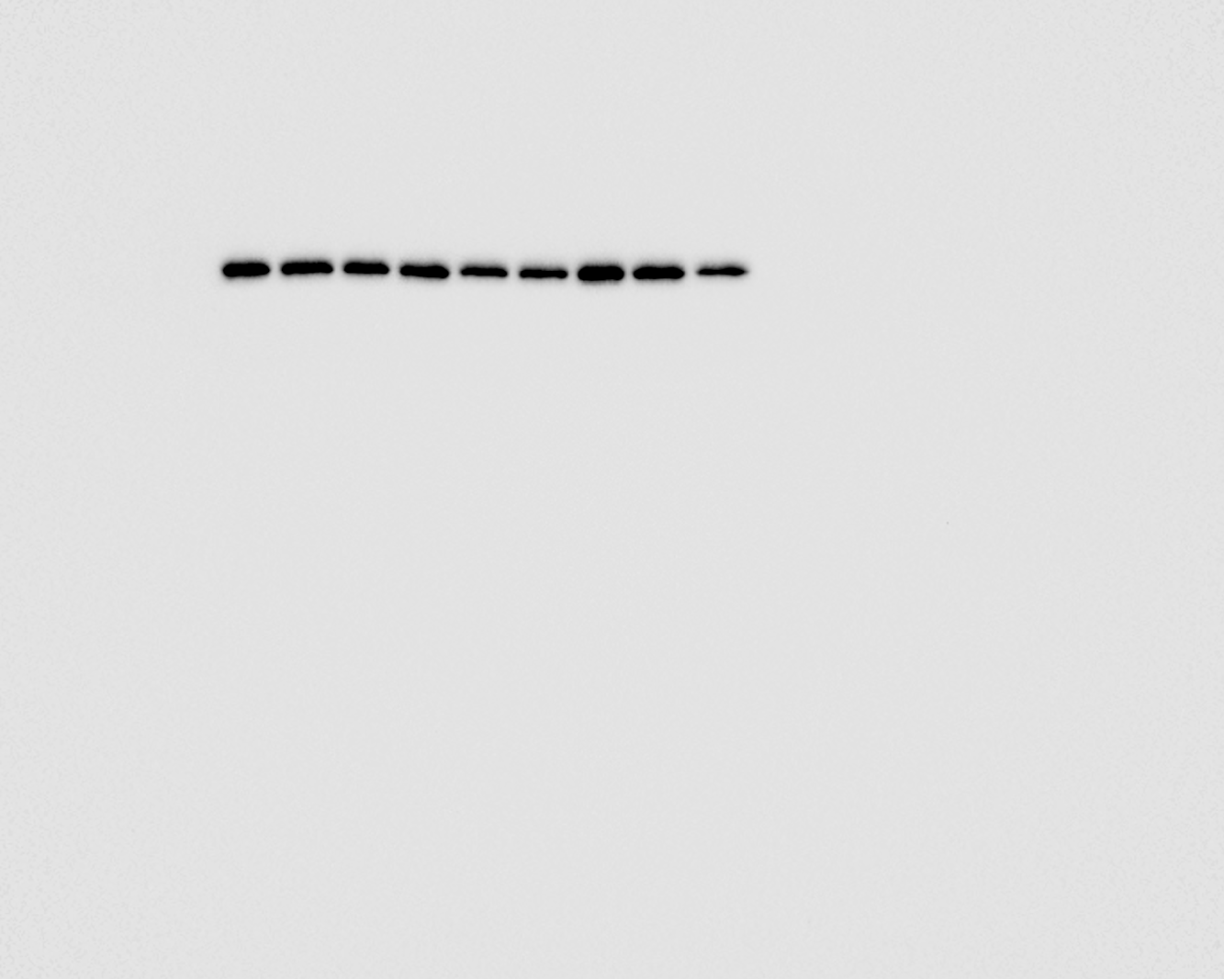

Supplement: Supplementary file 10 [file DataSheet5.ZIP › ST (2).tif]

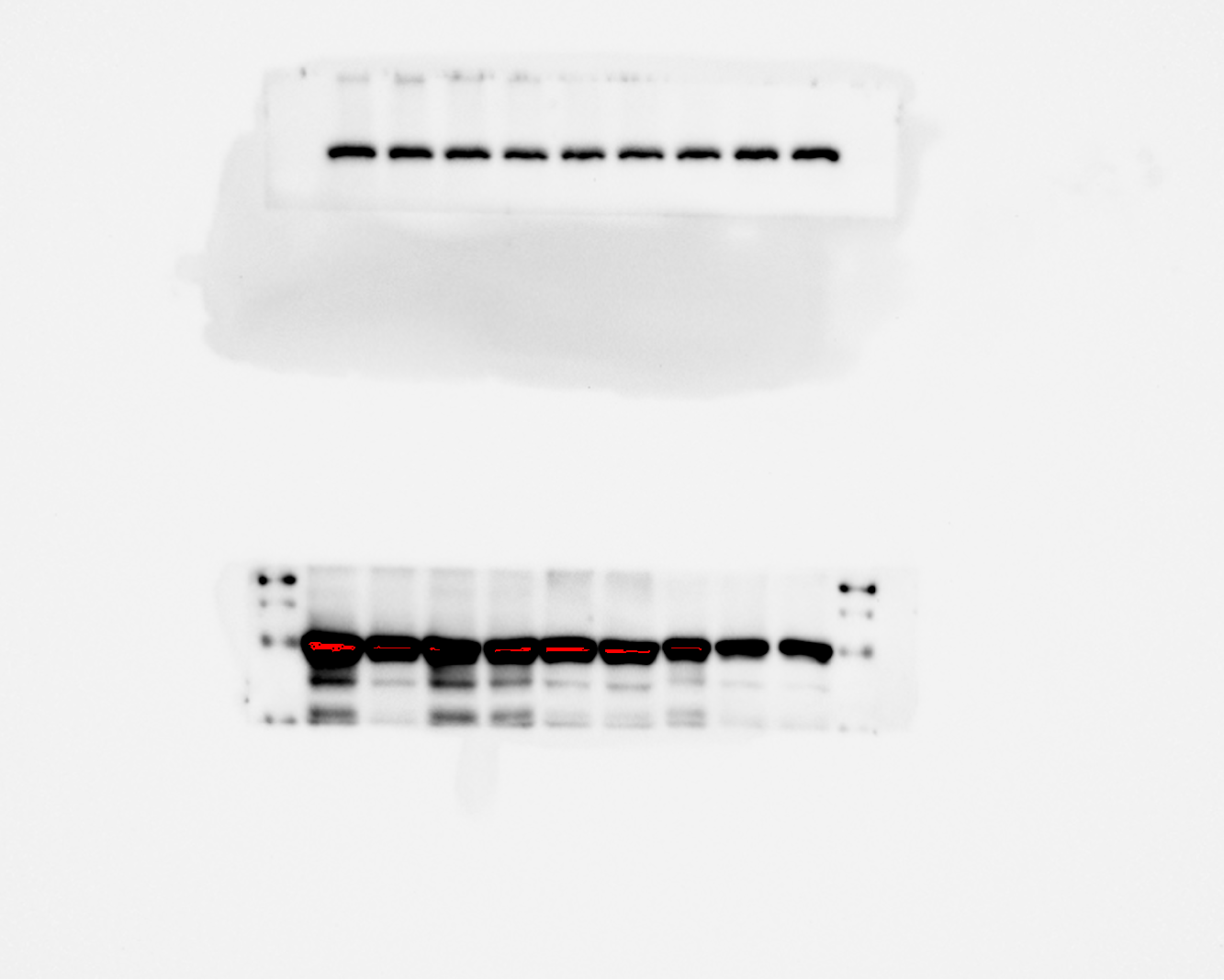

Supplement: Supplementary file 10 [file DataSheet5.ZIP › st-13_4(Chemiluminescence).tif]

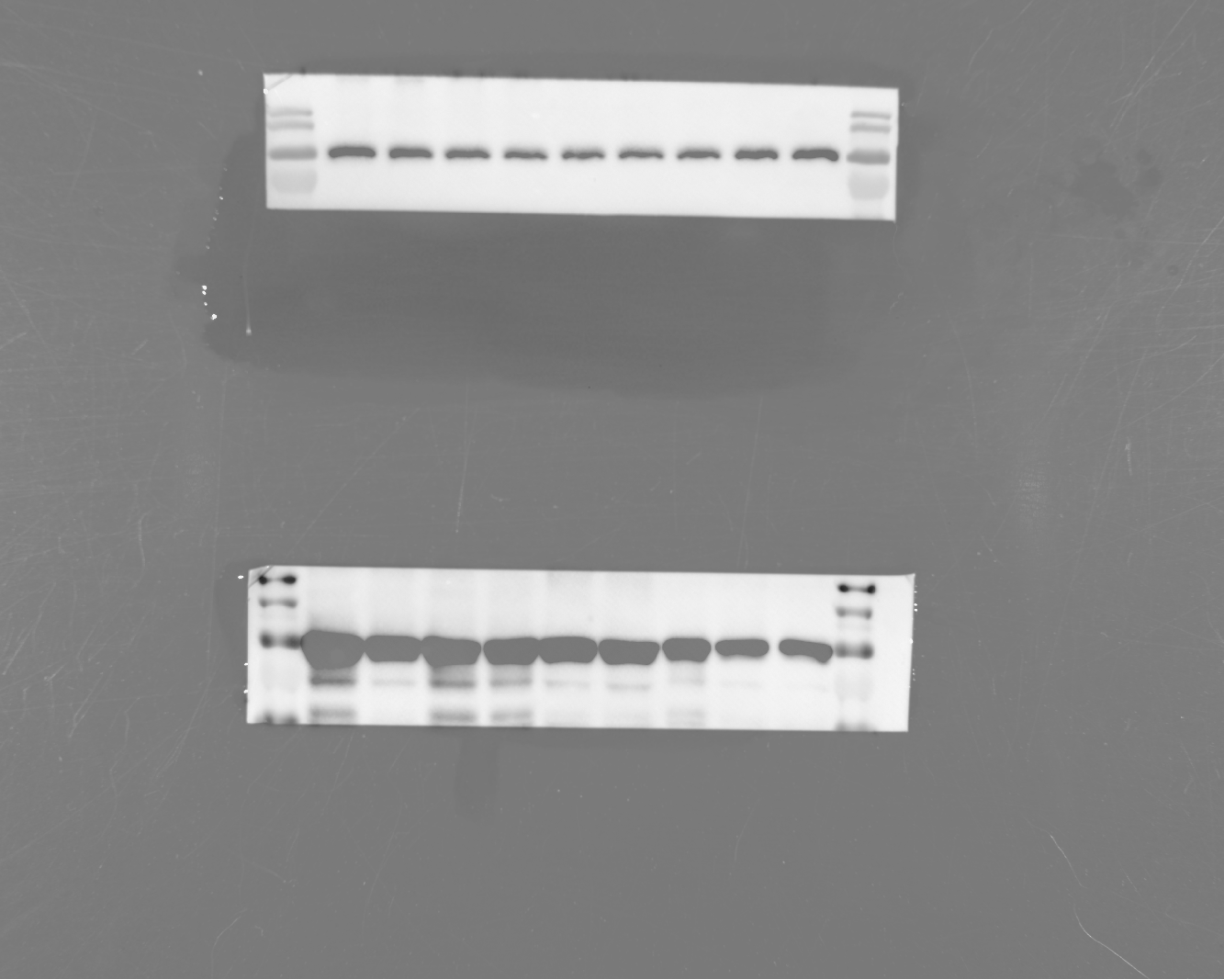

Supplement: Supplementary file 10 [file DataSheet5.ZIP › st-13_4(Composite).tif]

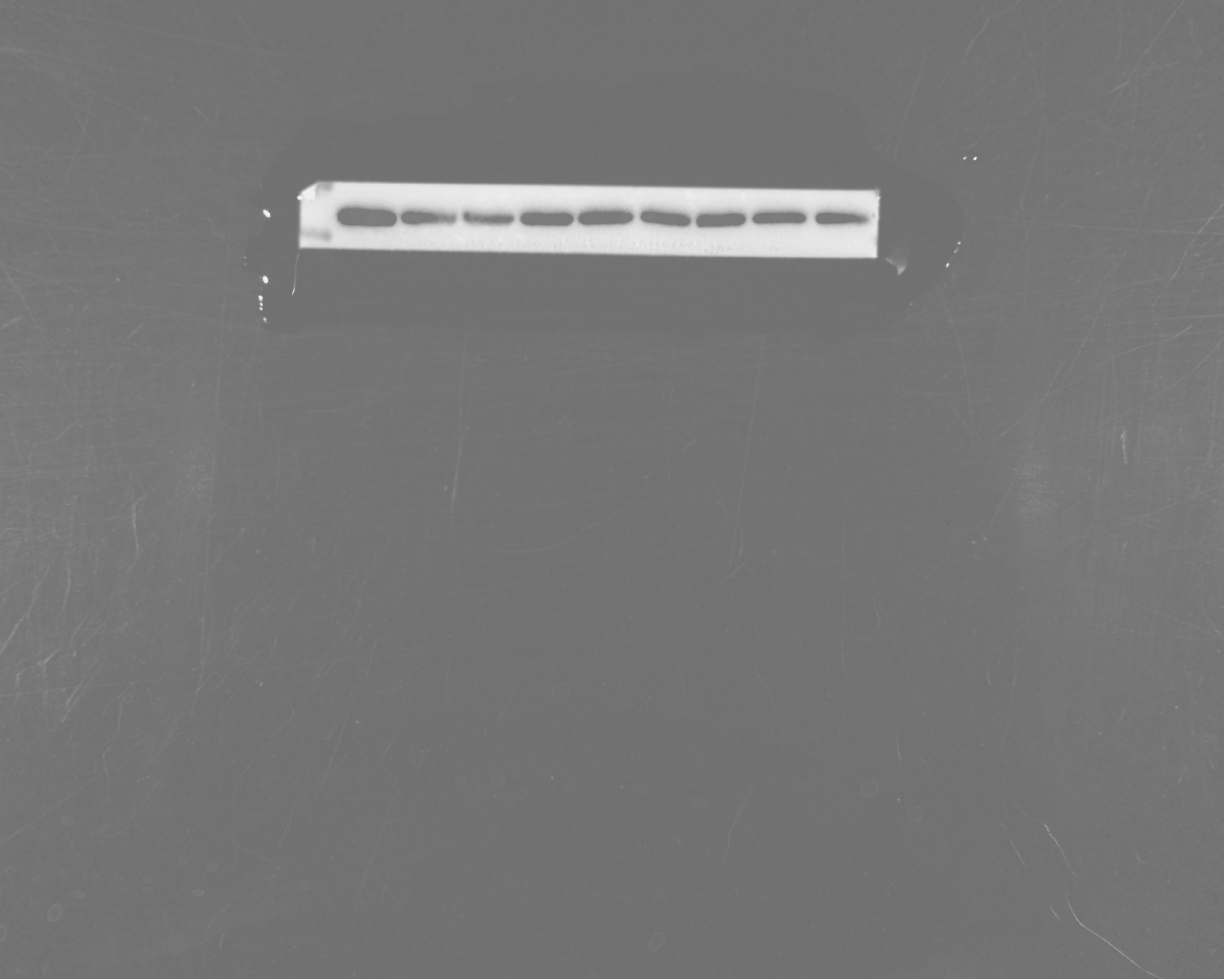

Supplement: Supplementary file 10 [file DataSheet5.ZIP › ST-2 (1).tif]

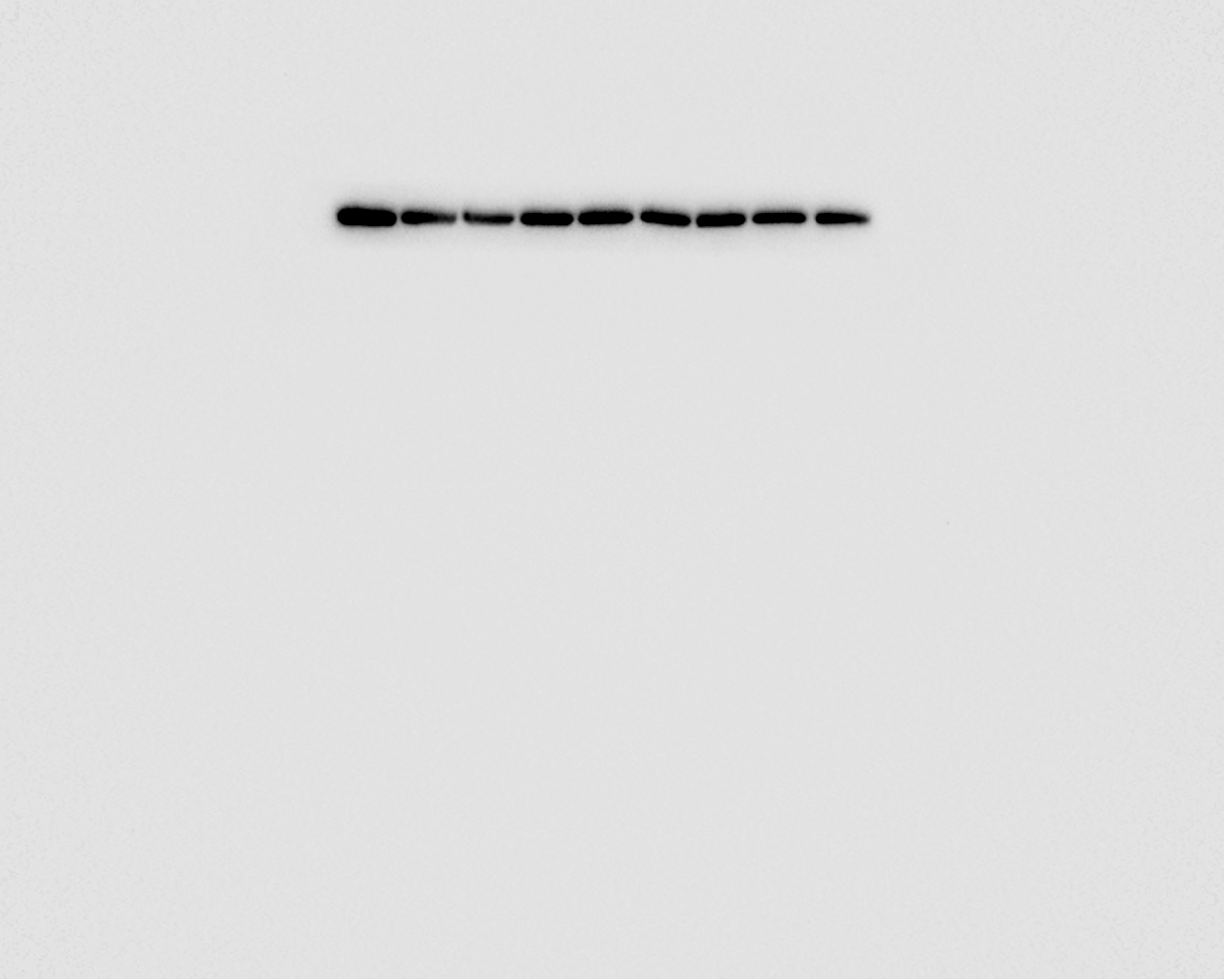

Supplement: Supplementary file 10 [file DataSheet5.ZIP › ST-2 (2).tif]

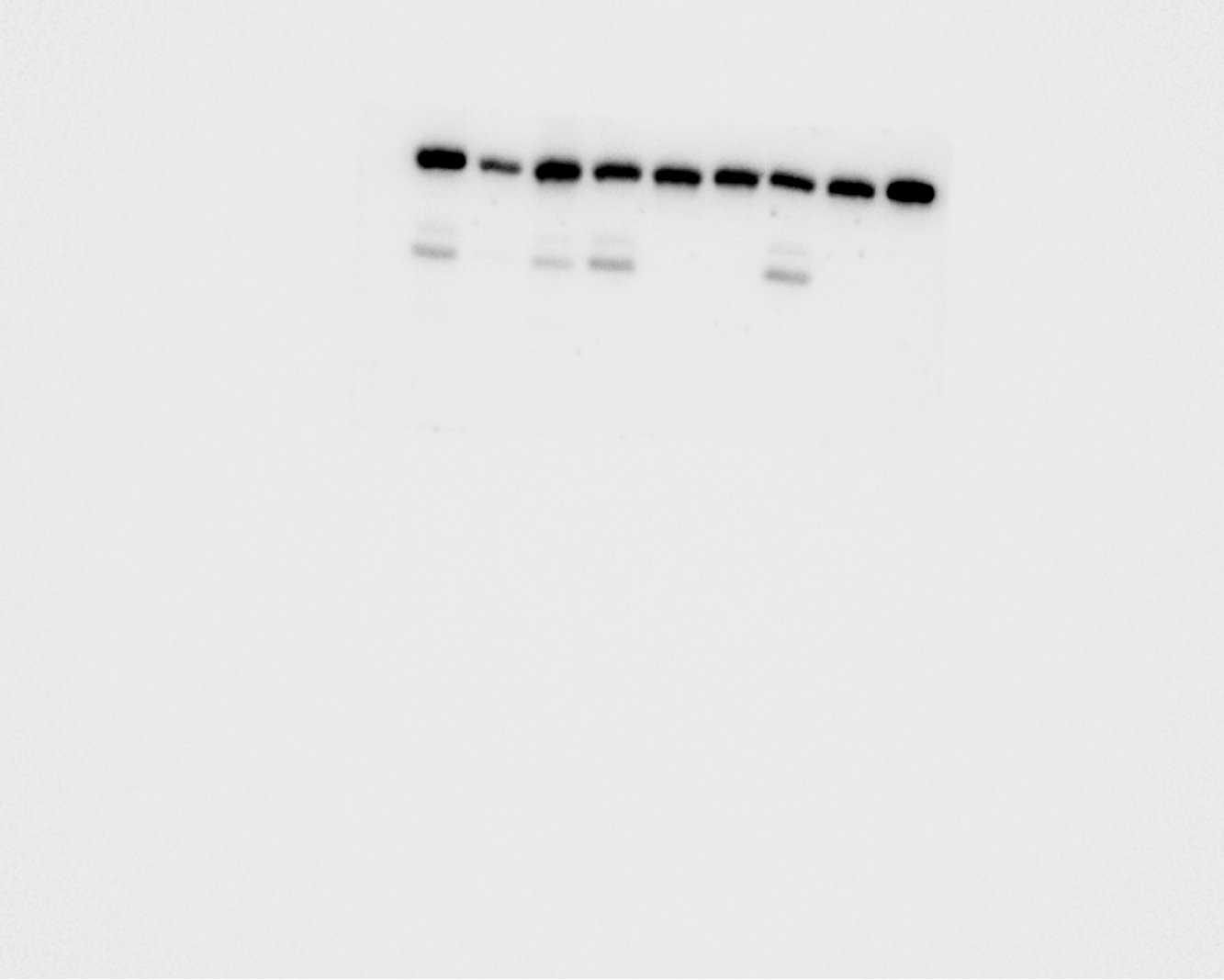

Supplement: Supplementary file 10 [file DataSheet5.ZIP › tublin-17_4(Chemiluminescence).tif]

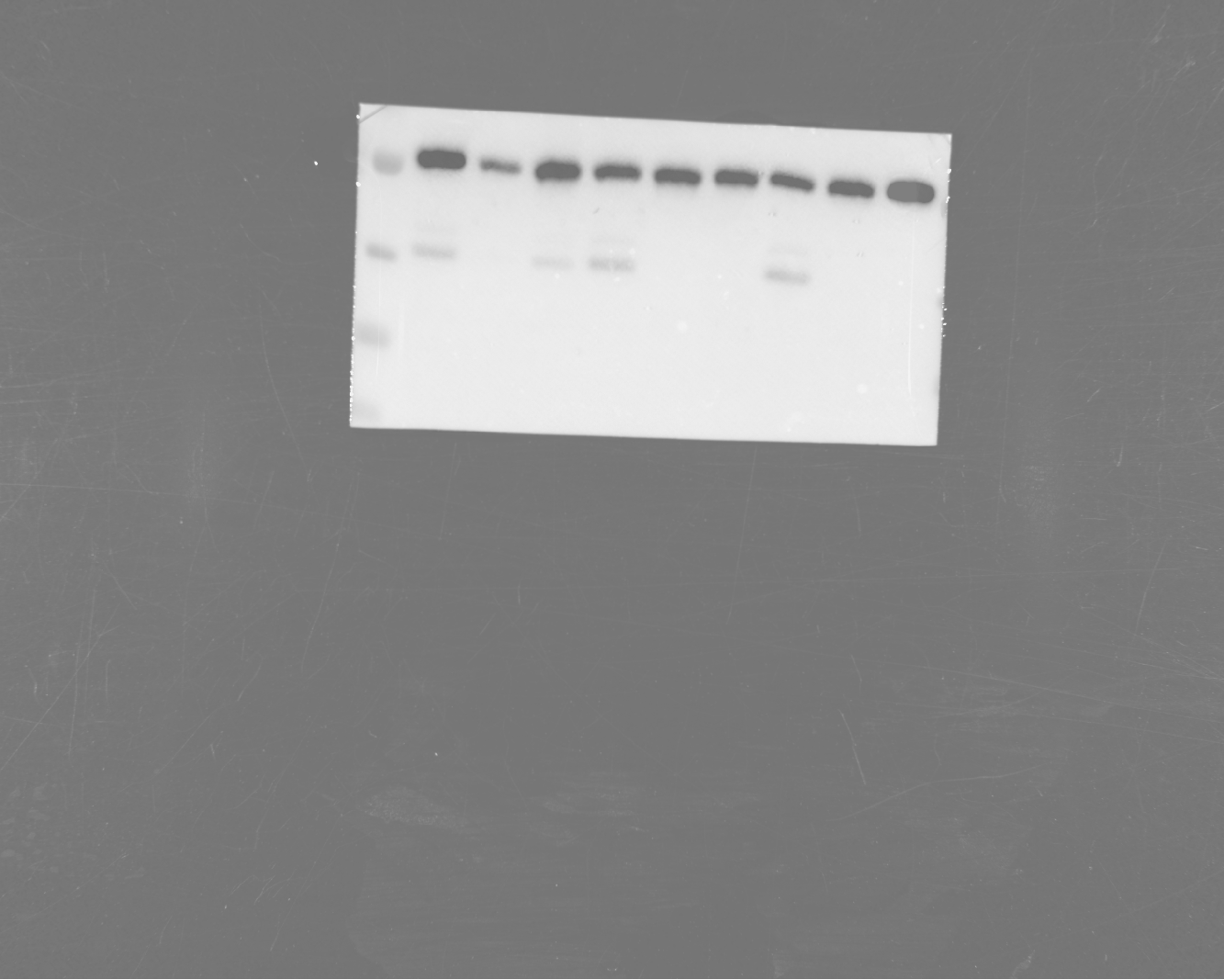

Supplement: Supplementary file 10 [file DataSheet5.ZIP › tublin-17_4(Composite).tif]

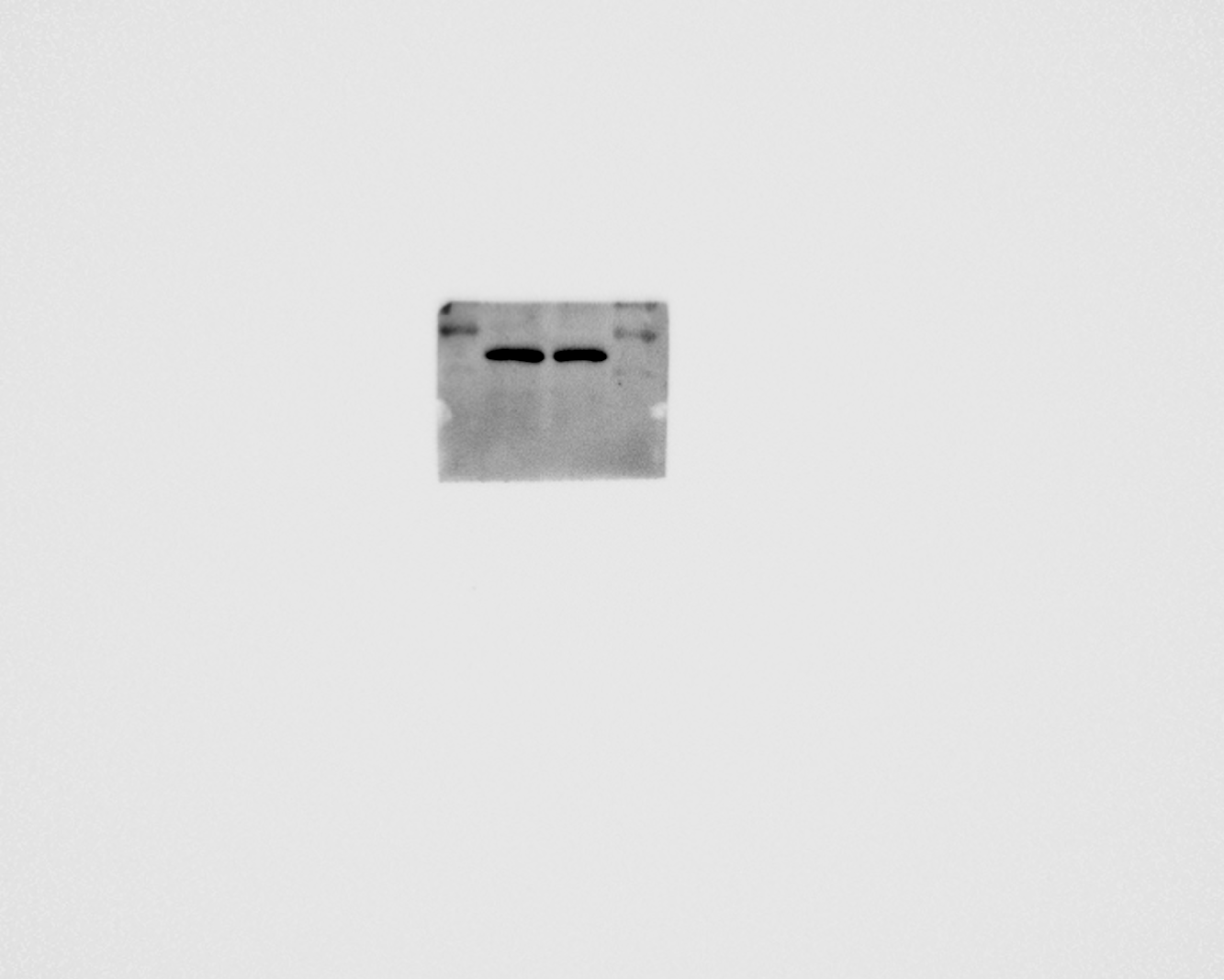

Supplement: Supplementary file 11 [file DataSheet7.ZIP › actin (1).tif]

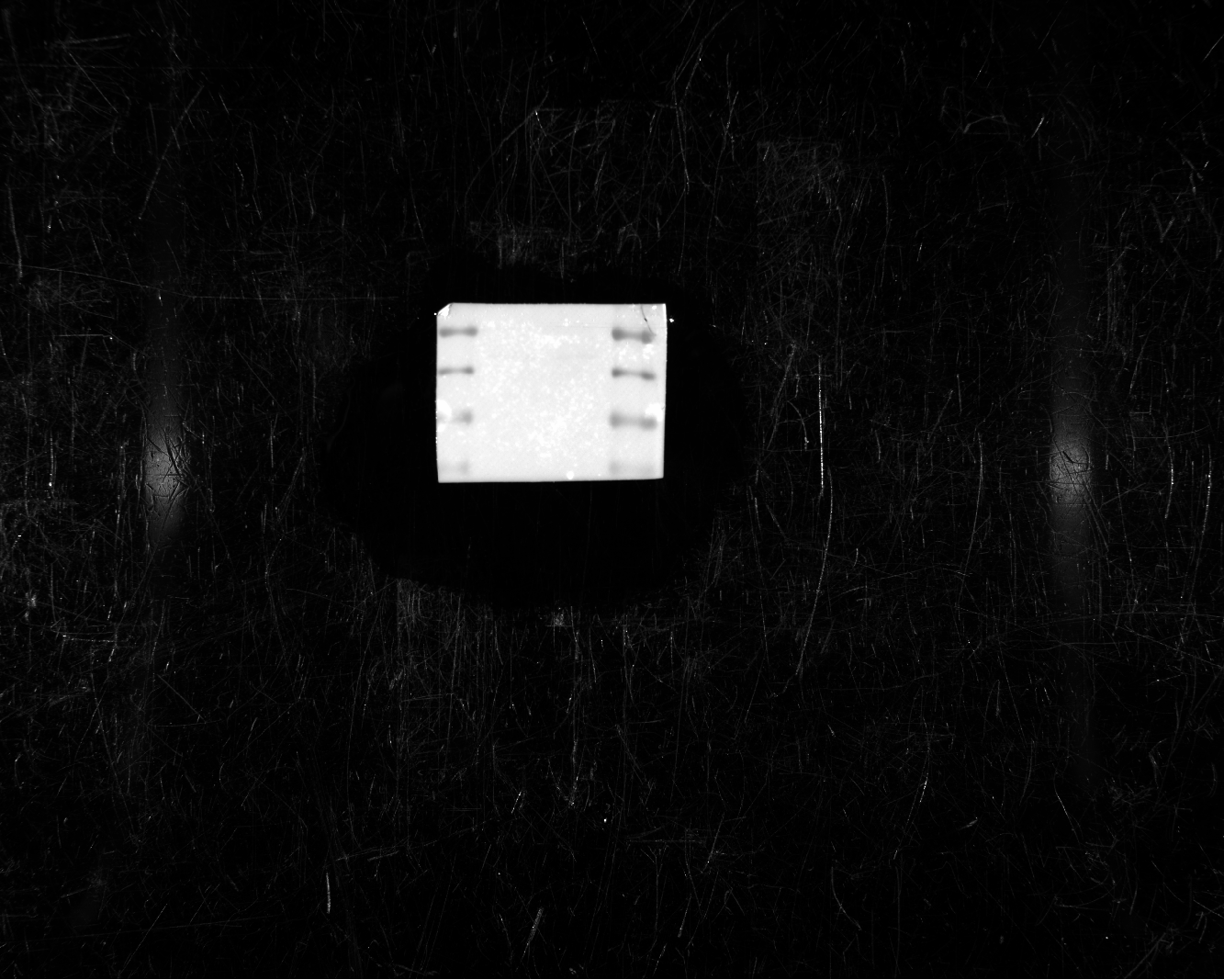

Supplement: Supplementary file 11 [file DataSheet7.ZIP › actin (2).tif]

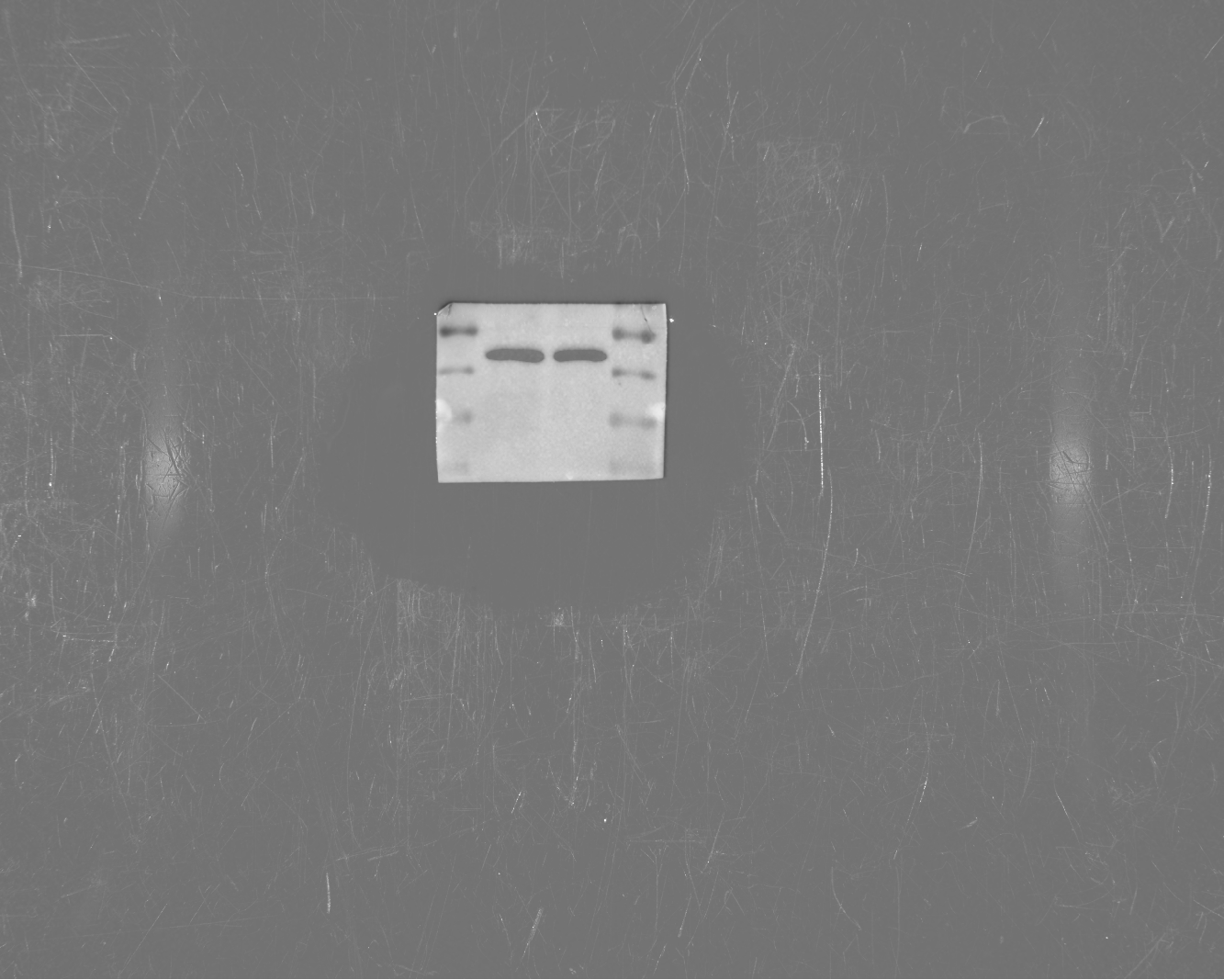

Supplement: Supplementary file 11 [file DataSheet7.ZIP › actin (3).tif]

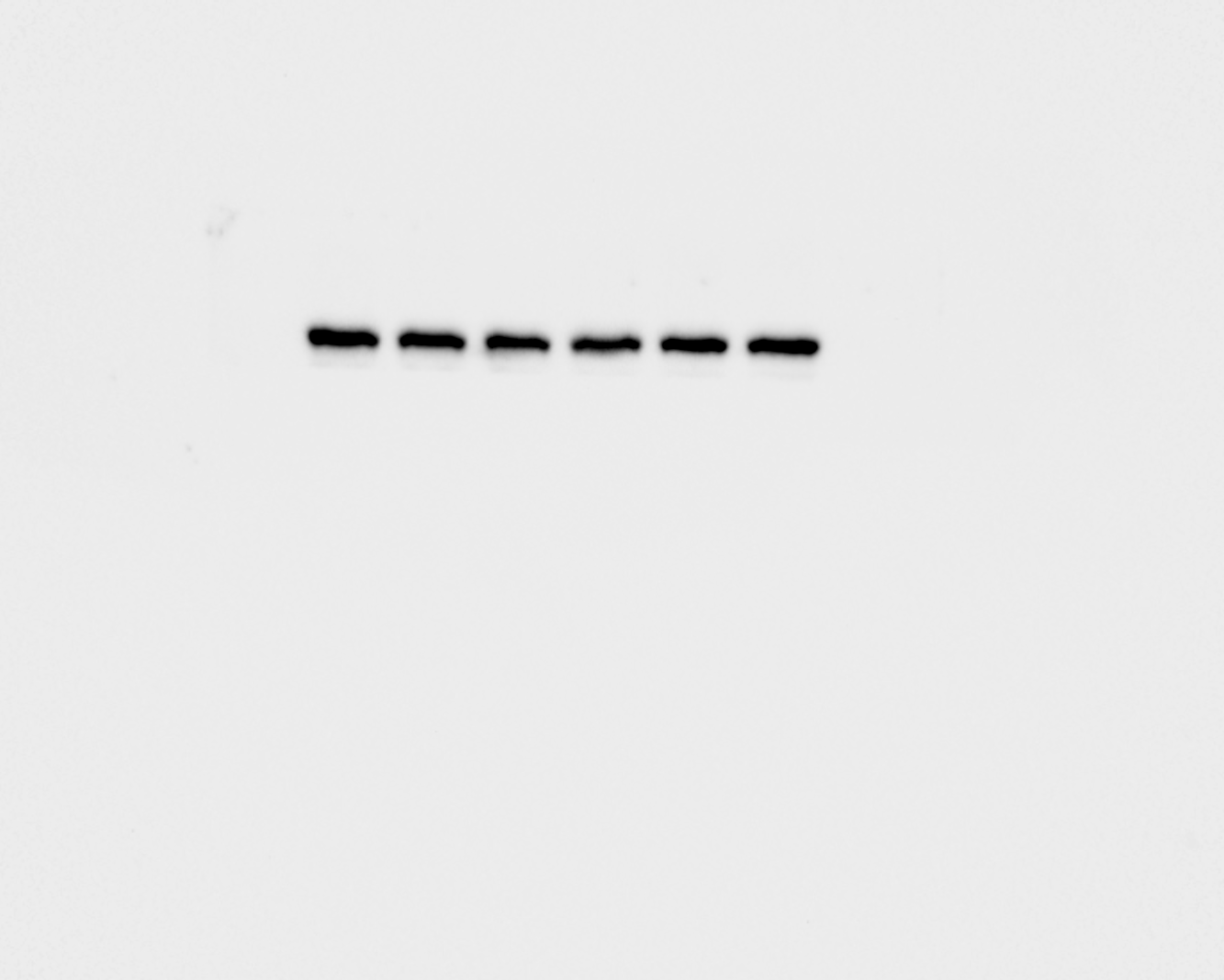

Supplement: Supplementary file 11 [file DataSheet7.ZIP › actin(Chemiluminescence) (2).tif]

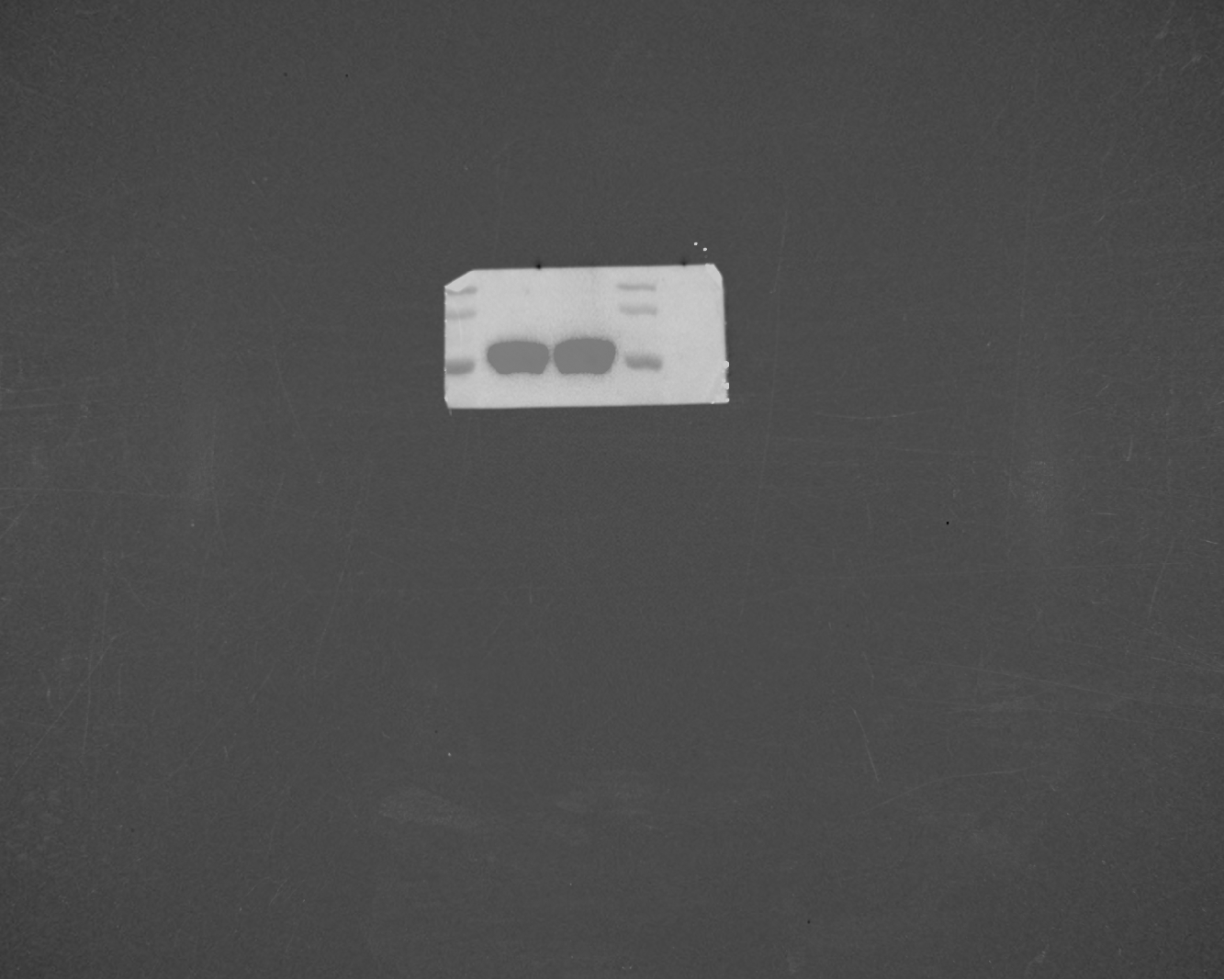

Supplement: Supplementary file 11 [file DataSheet7.ZIP › actin-3 (1).tif]

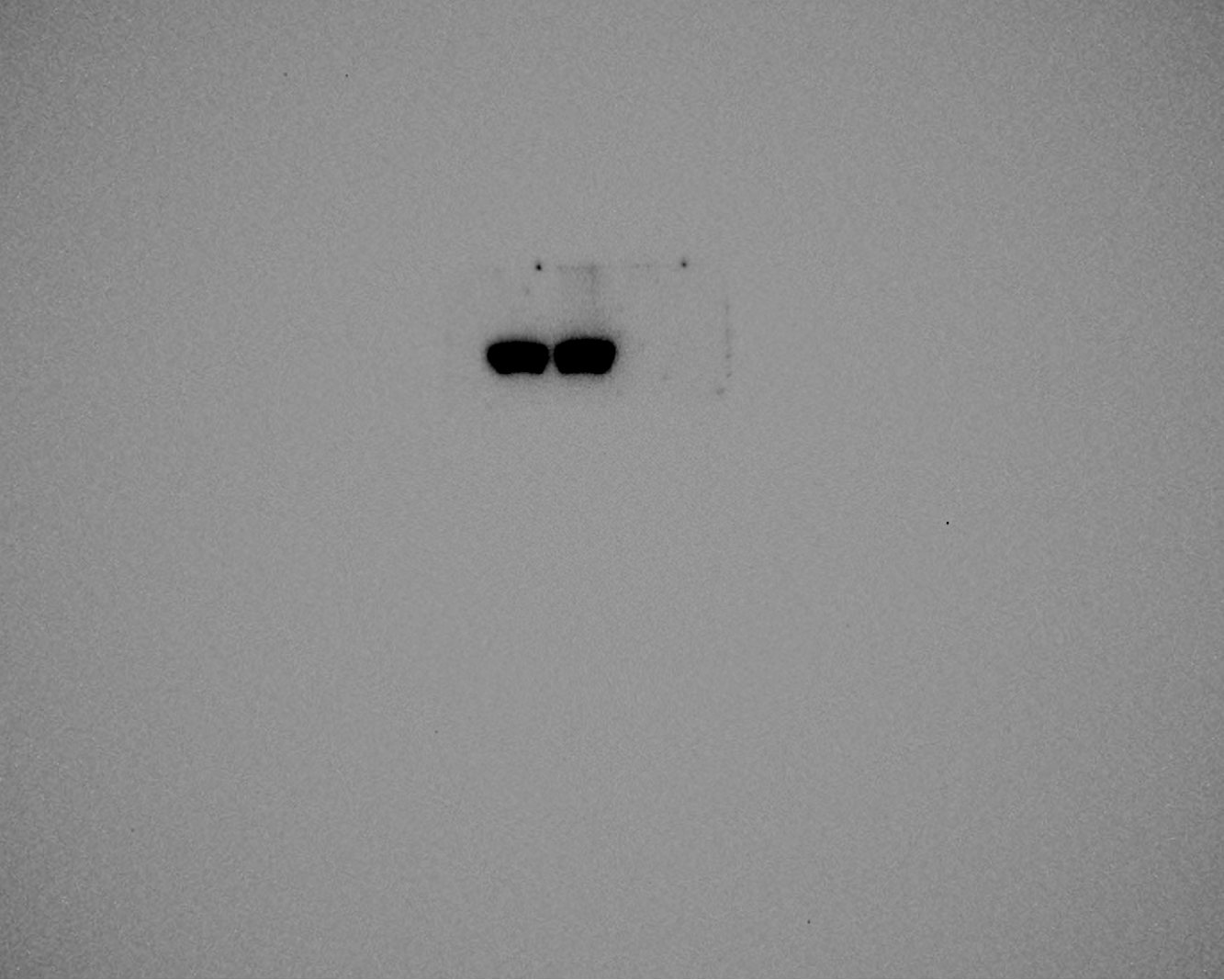

Supplement: Supplementary file 11 [file DataSheet7.ZIP › actin-3 (2).tif]

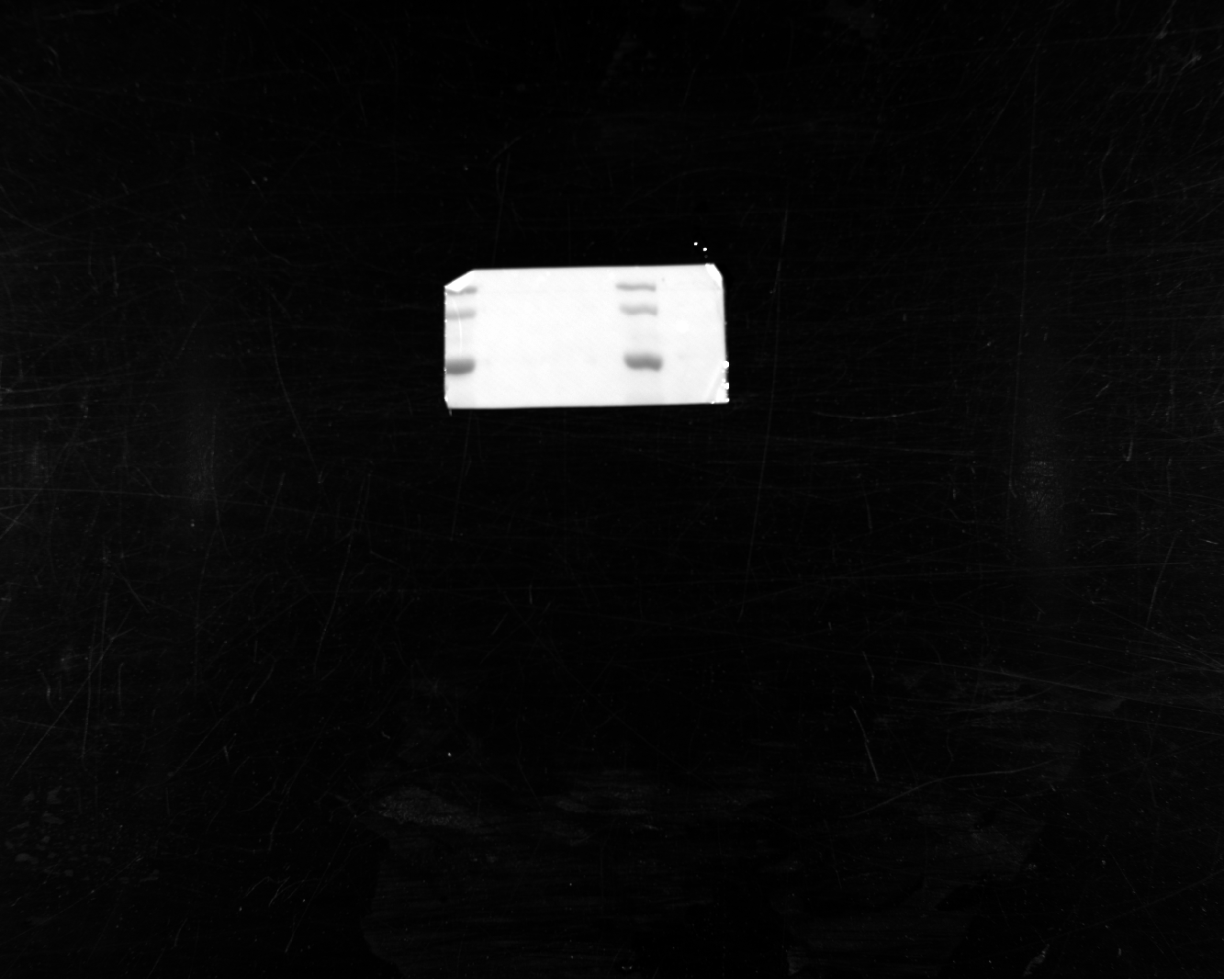

Supplement: Supplementary file 11 [file DataSheet7.ZIP › actin-3 (3).tif]

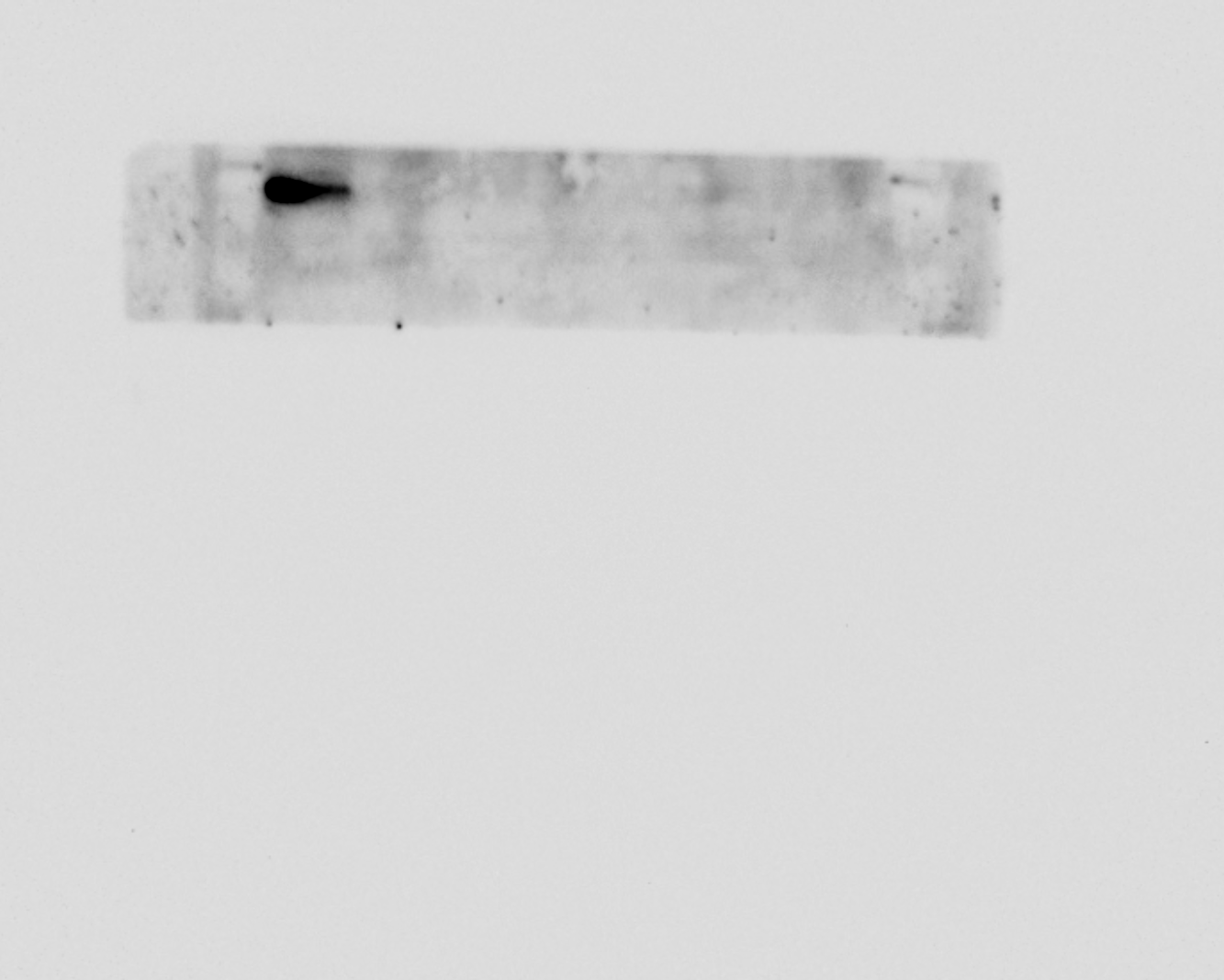

Supplement: Supplementary file 11 [file DataSheet7.ZIP › bcl2ncsi_1(Chemiluminescence).tif]

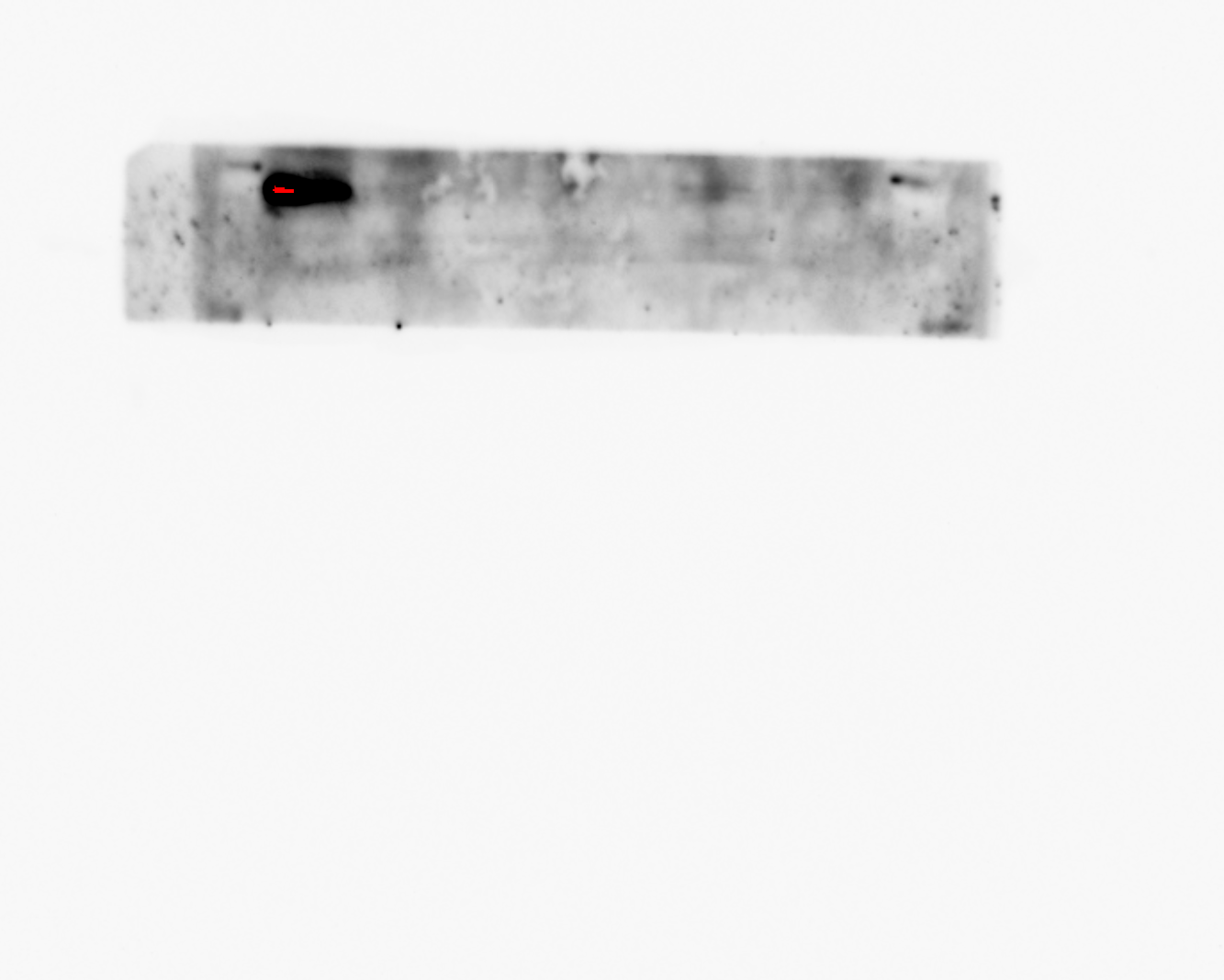

Supplement: Supplementary file 11 [file DataSheet7.ZIP › bcl2ncsi_2(Chemiluminescence).tif]

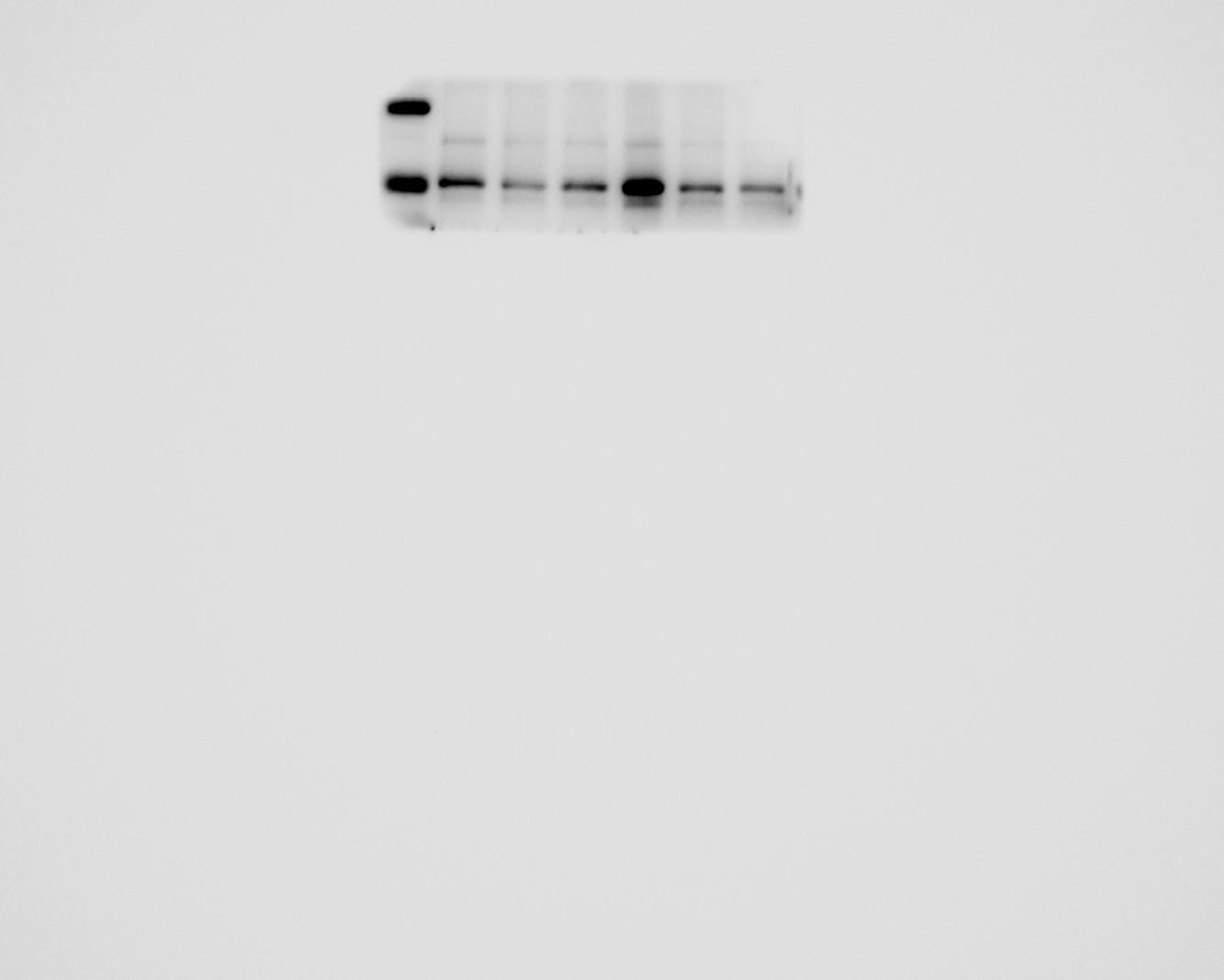

Supplement: Supplementary file 11 [file DataSheet7.ZIP › cas3.tif]

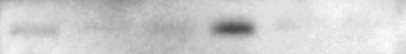

Supplement: Supplementary file 11 [file DataSheet7.ZIP › cas-3.tif]

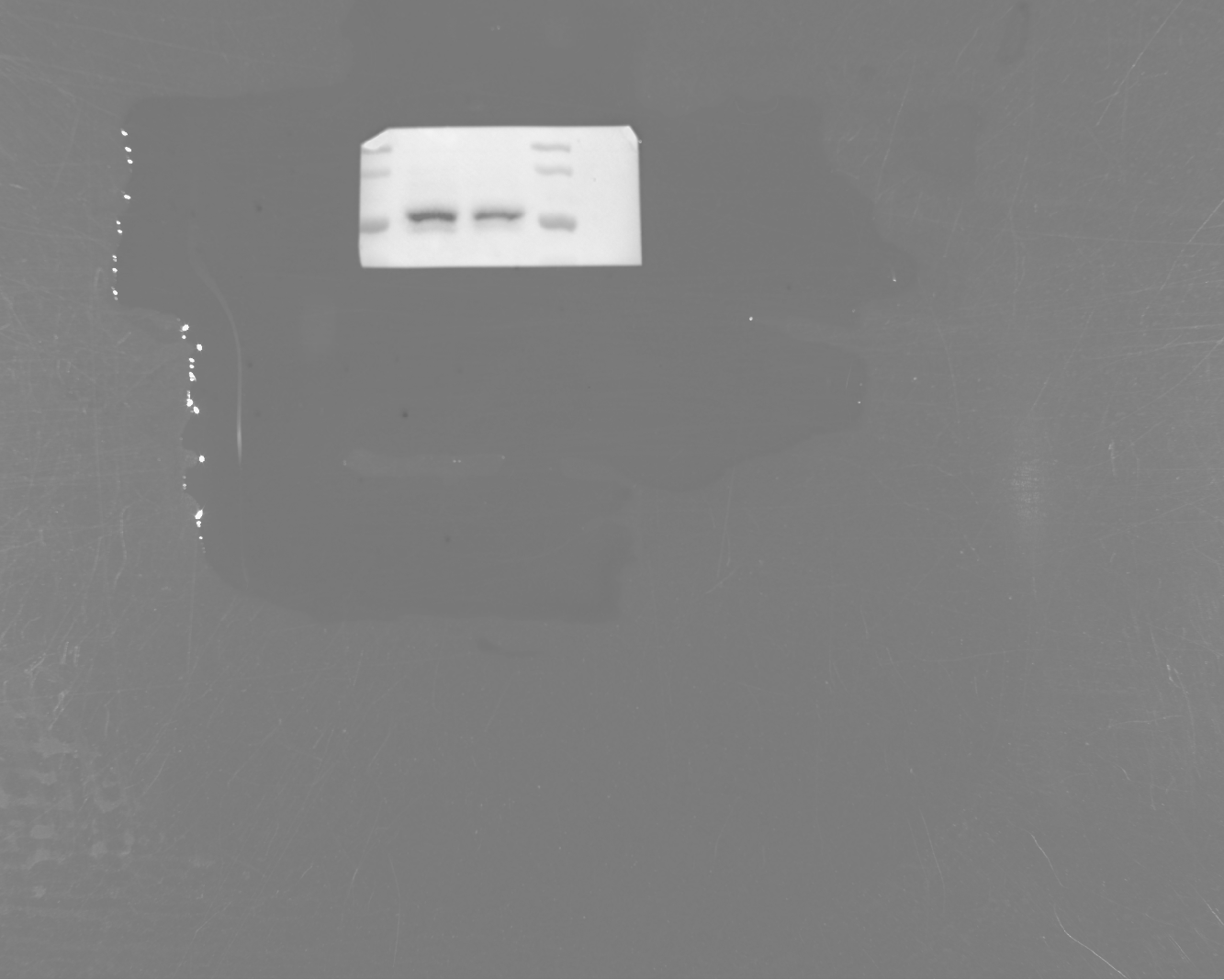

Supplement: Supplementary file 11 [file DataSheet7.ZIP › cd360-2 (1).tif]

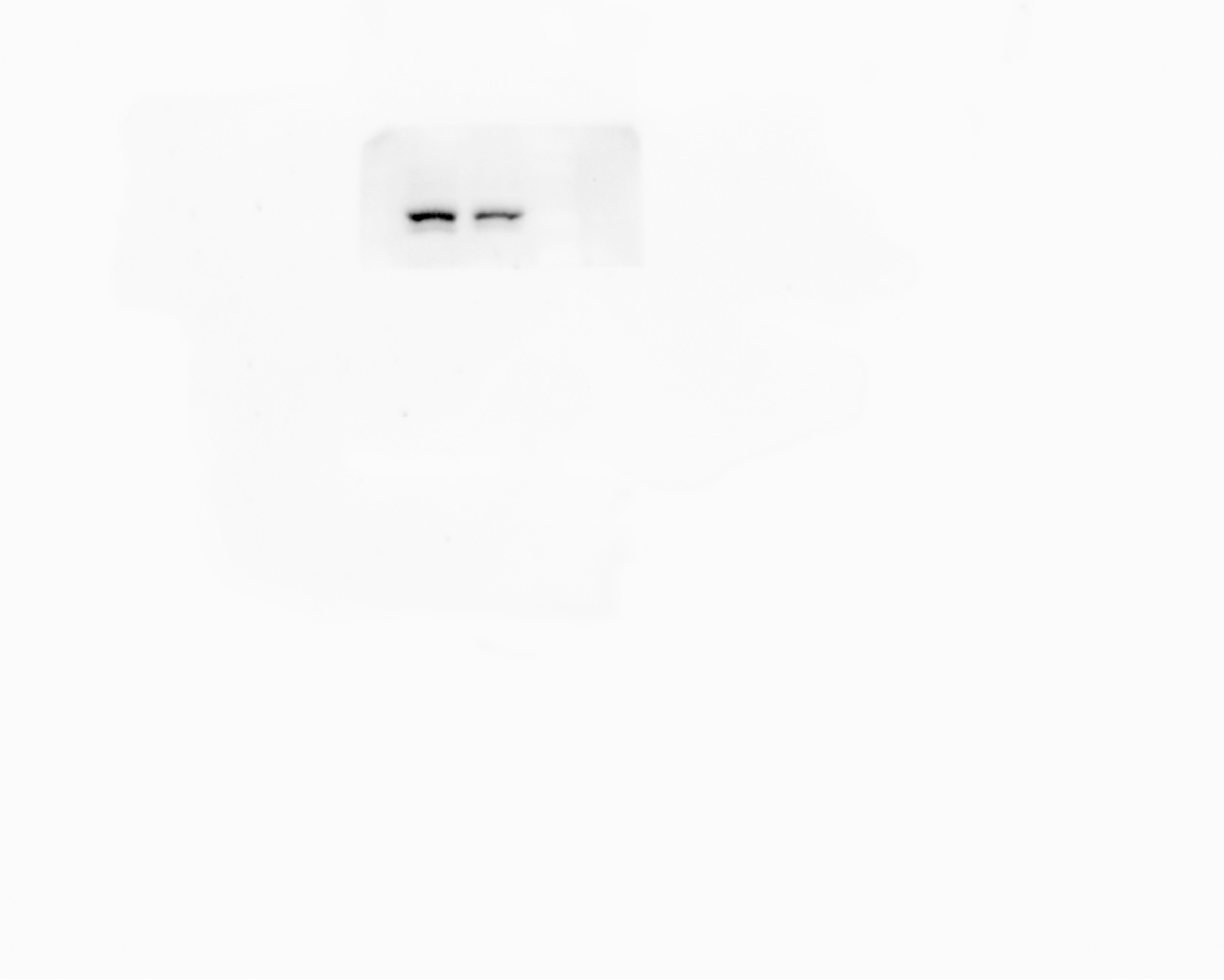

Supplement: Supplementary file 11 [file DataSheet7.ZIP › cd360-2 (2).tif]

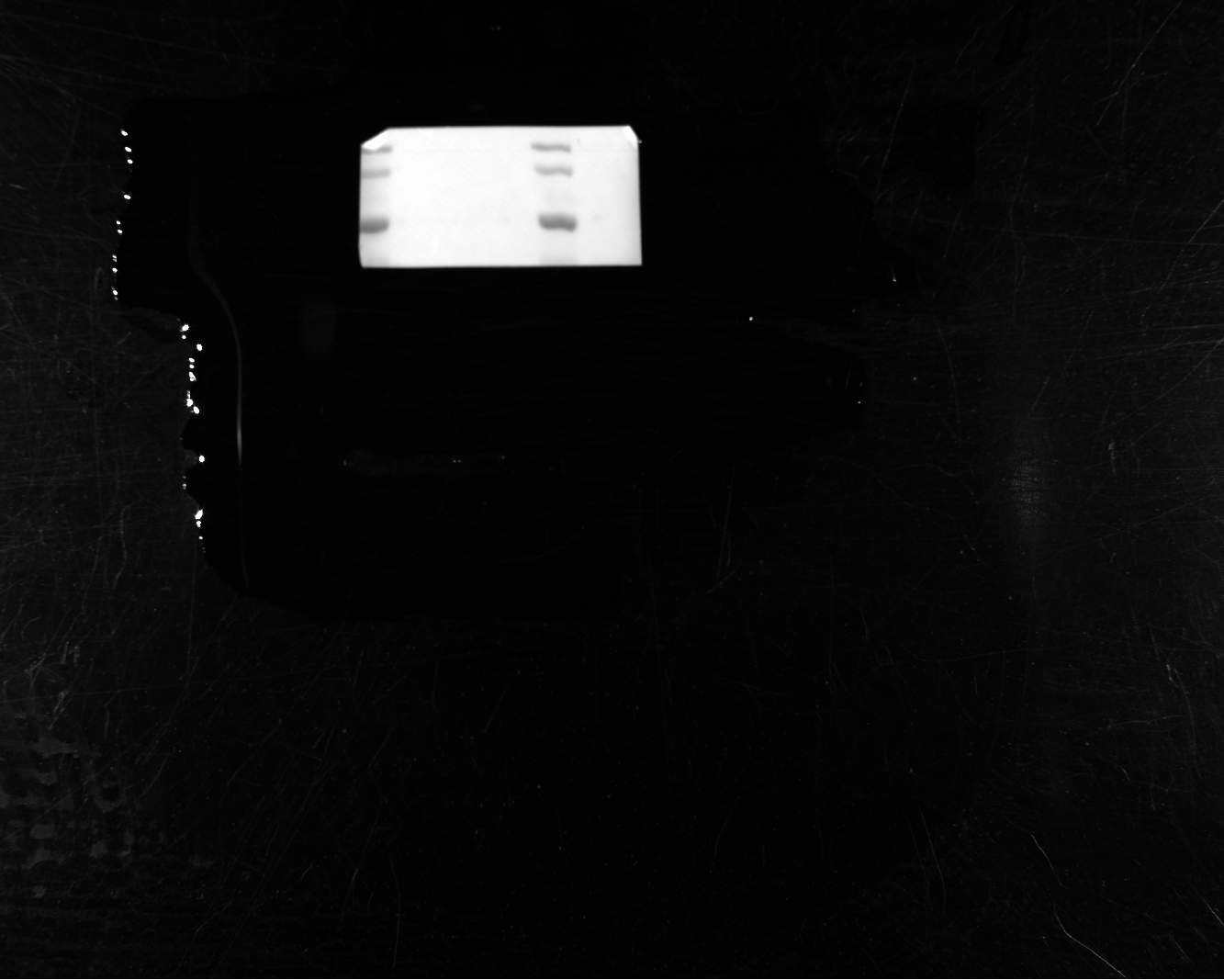

Supplement: Supplementary file 11 [file DataSheet7.ZIP › cd360-2 (3).tif]

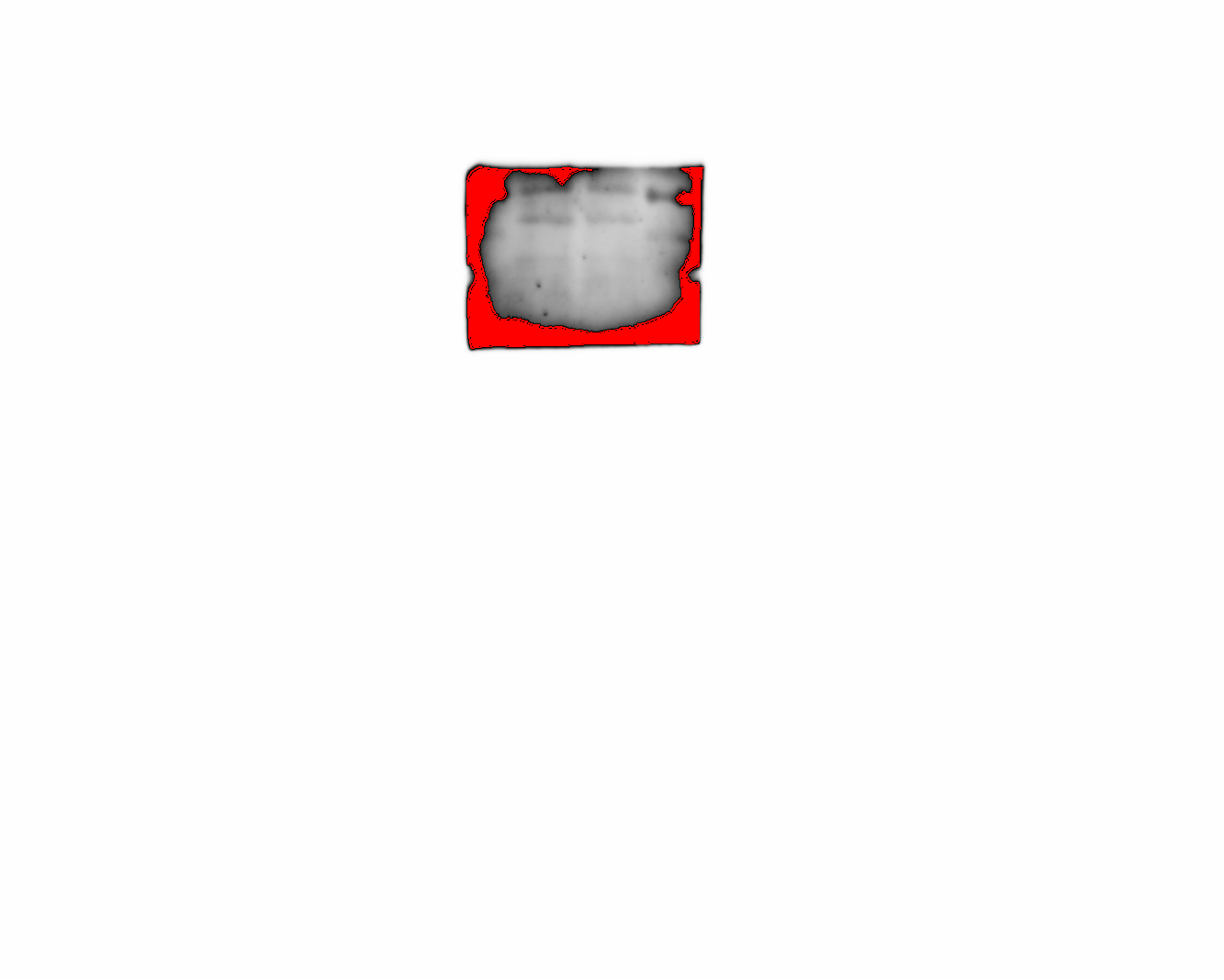

Supplement: Supplementary file 11 [file DataSheet7.ZIP › cd360-2_7(Chemiluminescence).tif]

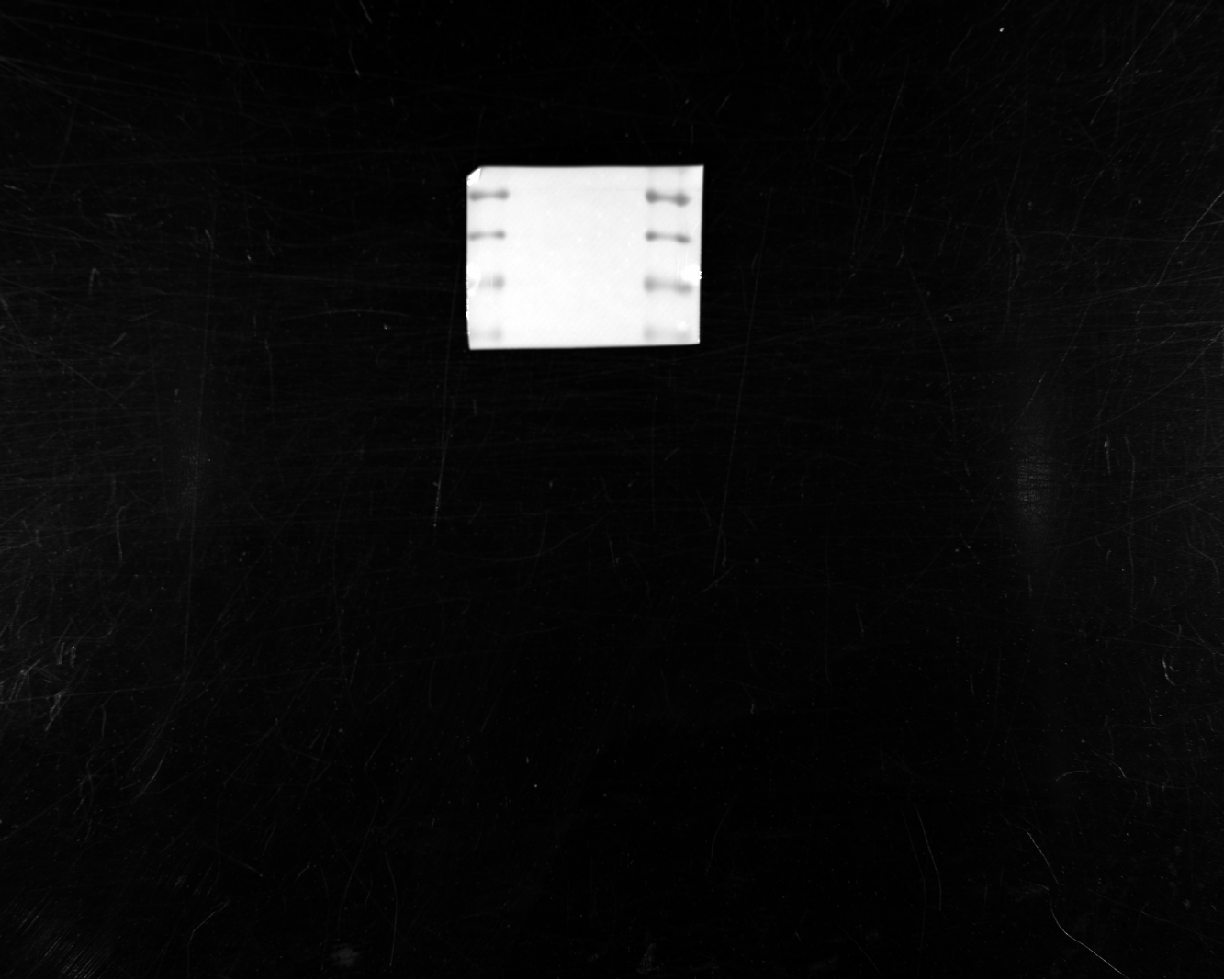

Supplement: Supplementary file 11 [file DataSheet7.ZIP › cd360-2_7(Colorimetric).tif]

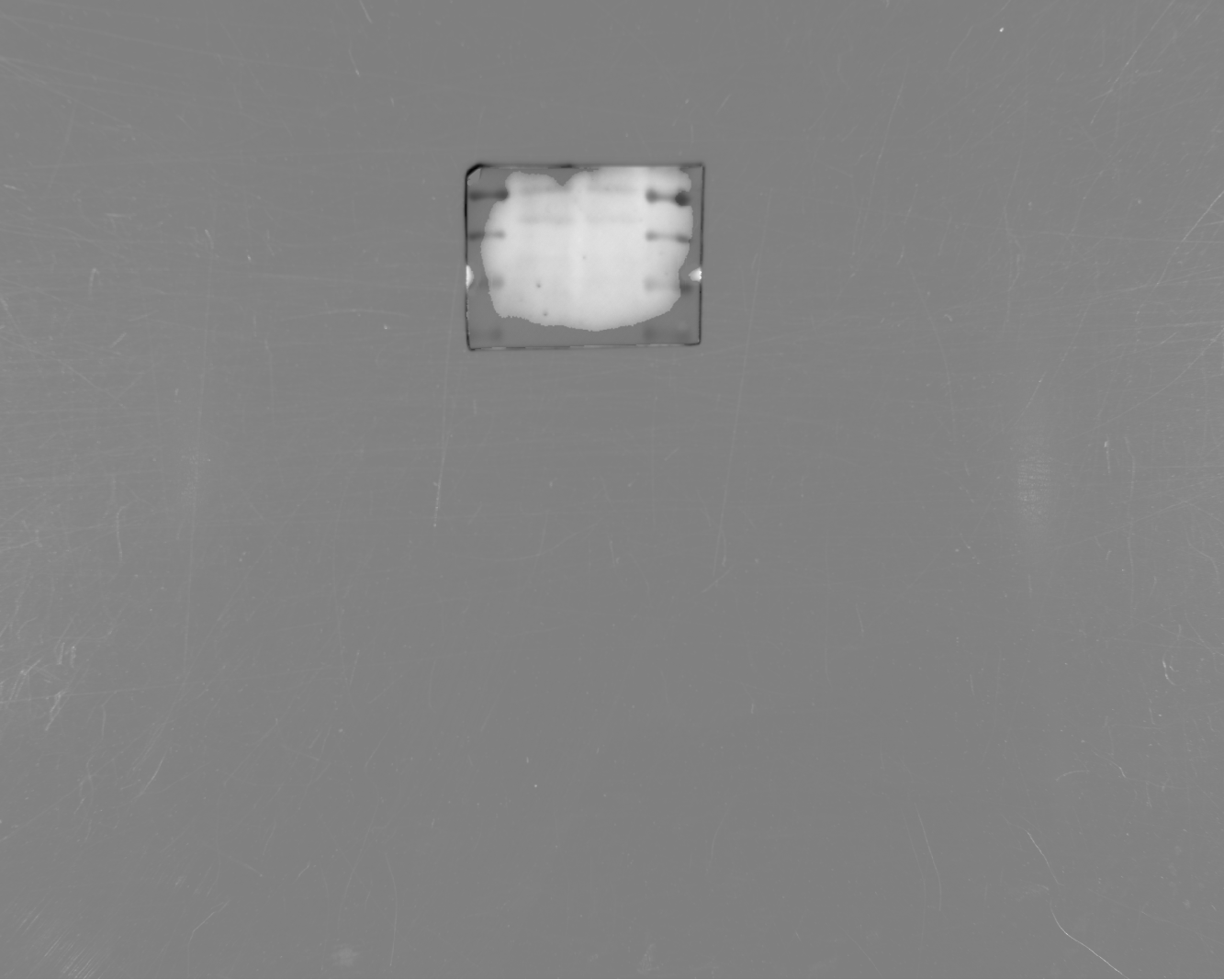

Supplement: Supplementary file 11 [file DataSheet7.ZIP › cd360-2_7(Composite).tif]

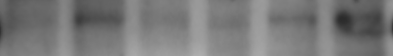

Supplement: Supplementary file 11 [file DataSheet7.ZIP › c-parprat_6(Chemiluminescence).tif]

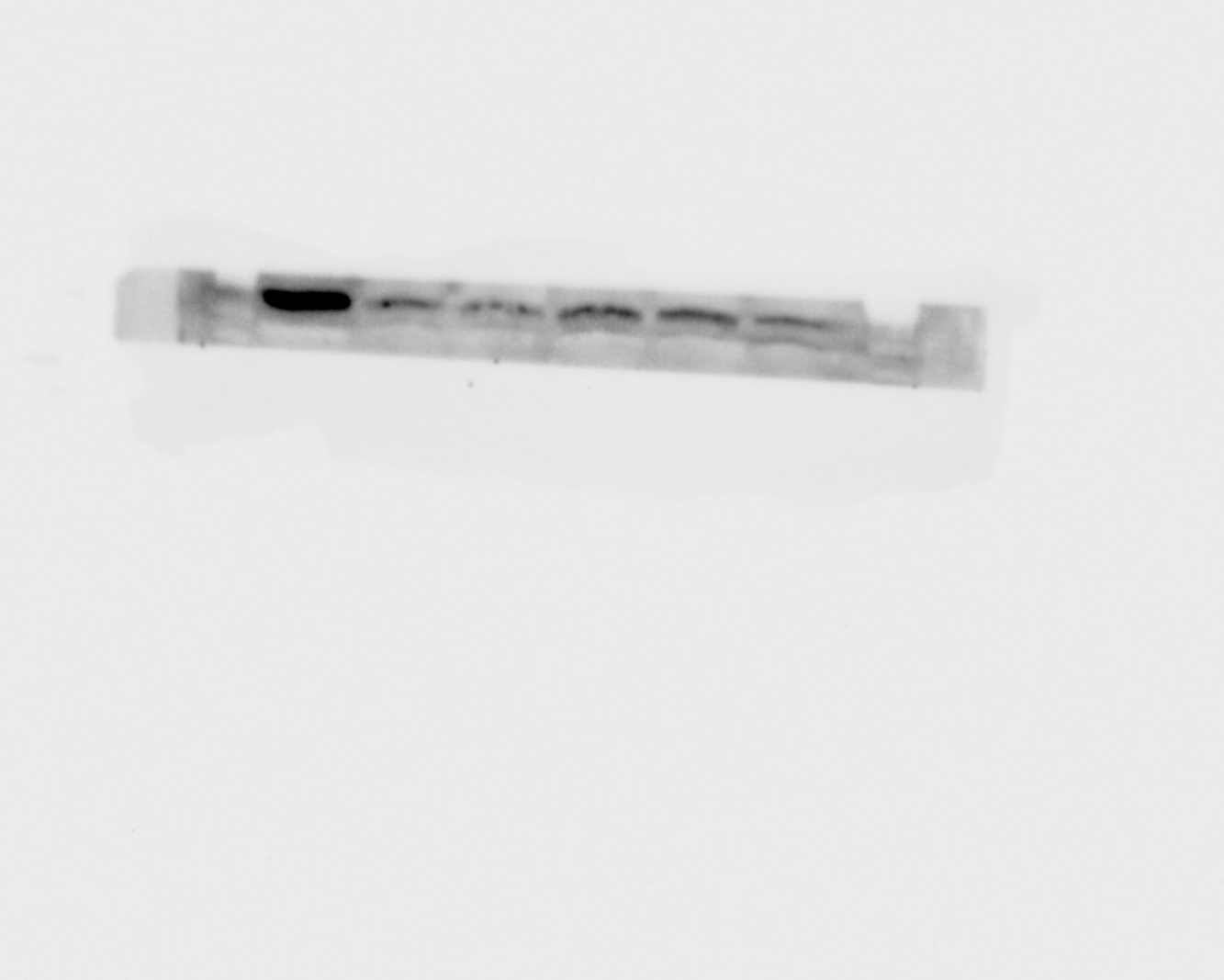

Supplement: Supplementary file 11 [file DataSheet7.ZIP › p53_1(Chemiluminescence).tif]

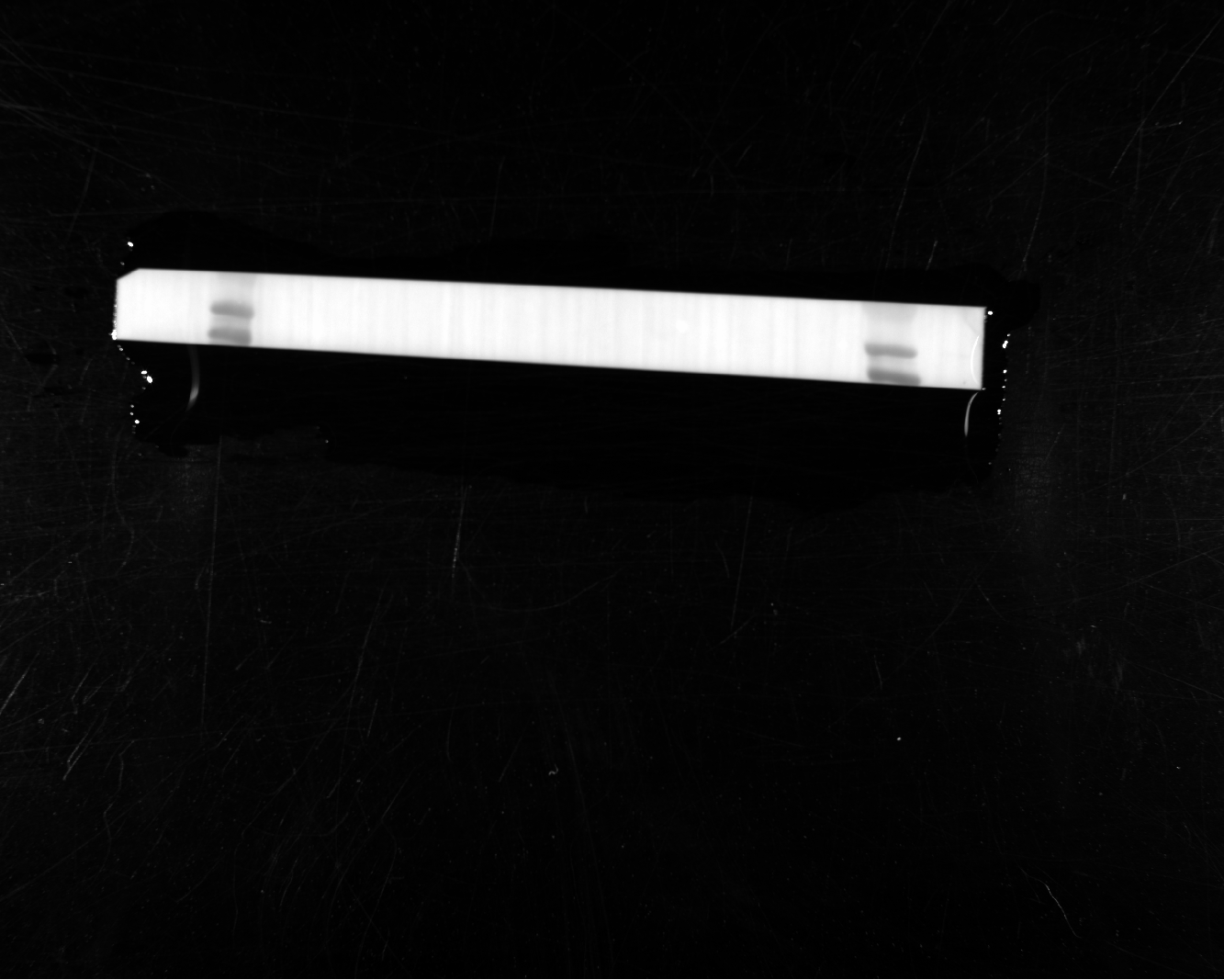

Supplement: Supplementary file 11 [file DataSheet7.ZIP › p53_2(Colorimetric).tif]

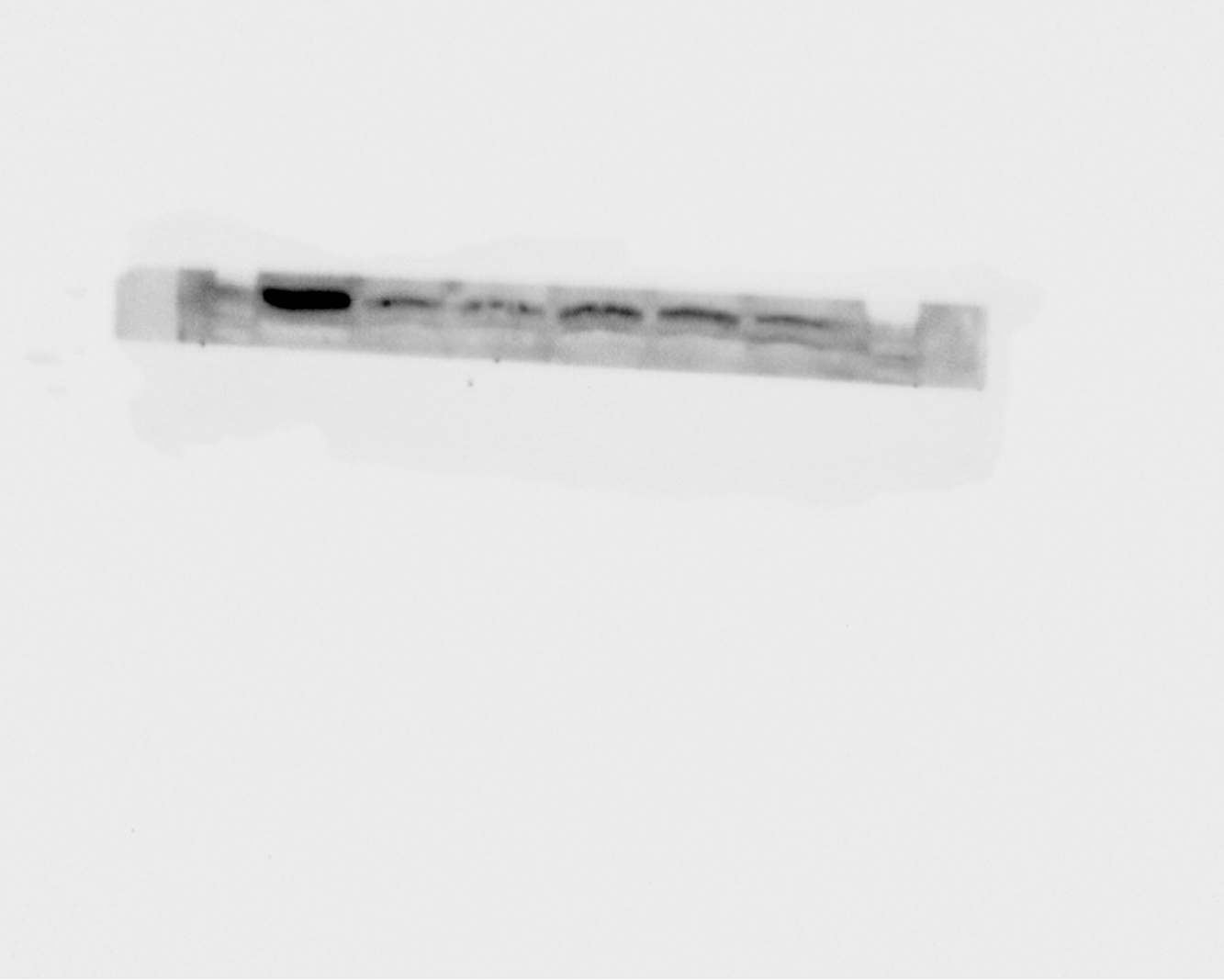

Supplement: Supplementary file 11 [file DataSheet7.ZIP › p53_3(Chemiluminescence).tif]

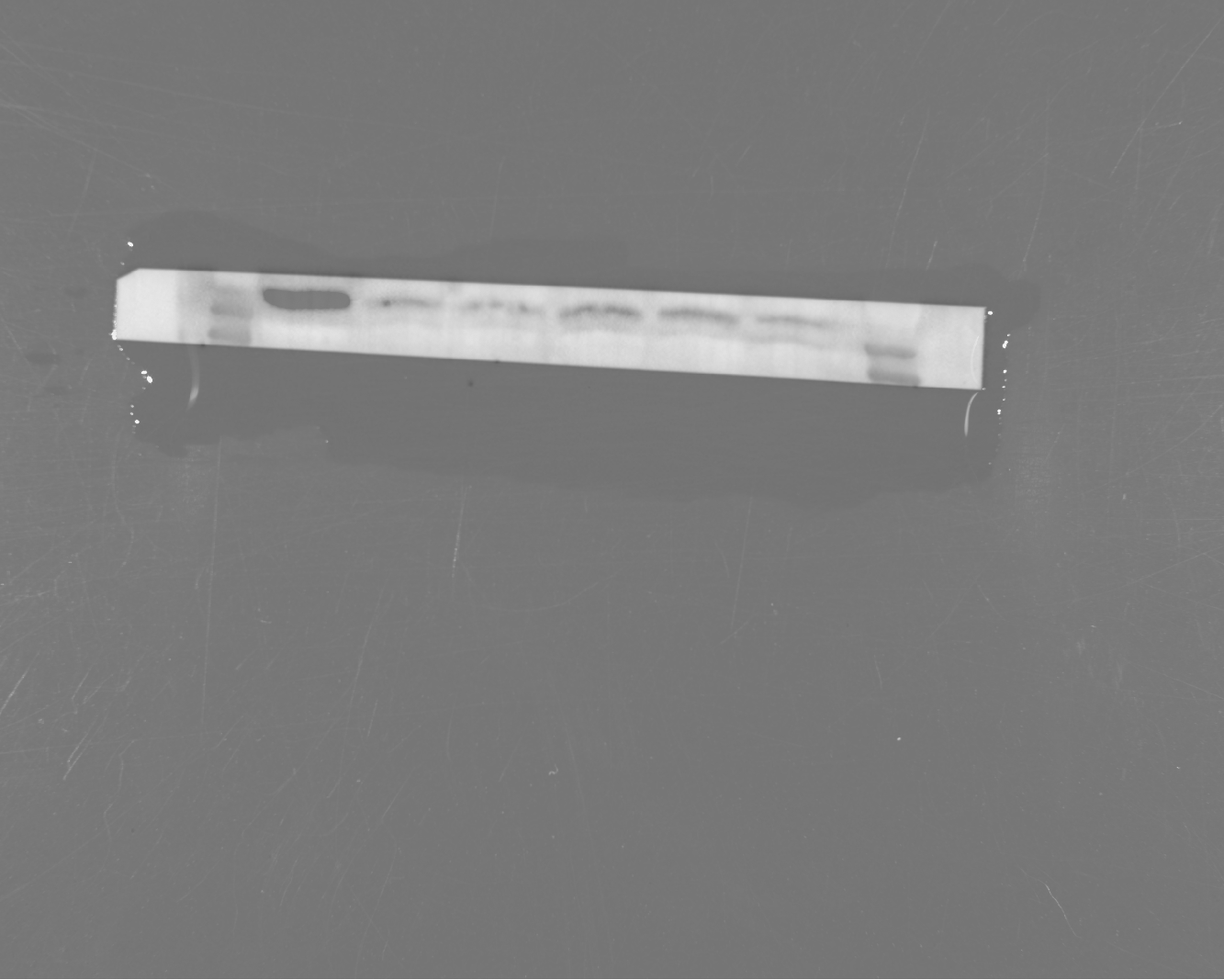

Supplement: Supplementary file 11 [file DataSheet7.ZIP › p53_3(Composite).tif]
